# HeH^+^ Collisions with H_2_: Rotationally Inelastic Cross Sections and Rate Coefficients from Quantum Dynamics at Interstellar Temperatures

**DOI:** 10.1021/acs.jpca.1c10309

**Published:** 2022-04-01

**Authors:** K. Giri, L. González-Sánchez, Rupayan Biswas, E. Yurtsever, F. A. Gianturco, N. Sathyamurthy, U. Lourderaj, R. Wester

**Affiliations:** † Department of Computational Sciences, Central University of Punjab, Bathinda, Punjab 151401, India; ‡ Departamento de Química Física, University of Salamanca Plaza de los Caídos sn, 37008 Salamanca, Spain; § School of Chemical Sciences, National Institute of Science Education and Research (NISER) Bhubaneswar, An OCC of Homi Bhabha National Institute, P.O. Jatni, Khurda, Odisha 752050, India; ∥ Department of Chemistry, Koc University Rumelifeneriyolu, Sariyer TR 34450 Istanbul, Turkey; ⊥ Institut für Ionenphysik und Angewandte Physik, Universität Innsbruck Technikerstaße 25, A-6020 Innsbruck, Austria; # Indian Institute of Science Education and Research Mohali, SAS Nagar, Manauli, Punjab 140306, India

## Abstract

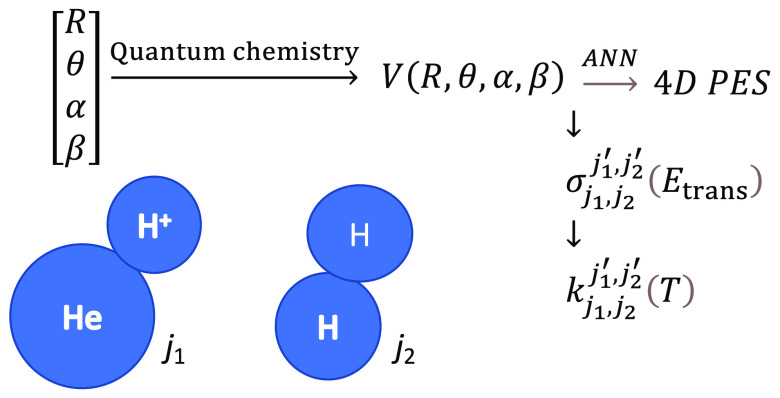

We report for the first time an accurate ab initio potential energy surface for the HeH^+^–H_2_ system in four dimensions (4D) treating both diatomic species as rigid rotors. The computed ab initio potential energy point values are fitted using an artificial neural network method and used in quantum close coupling calculations for different initial states of both rotors, in their ground electronic states, over a range of collision energies. The state-to-state cross section results are used to compute the rate coefficients over a range of temperatures relevant to interstellar conditions. By comparing the four dimensional quantum results with those obtained by a reduced-dimensions approach that treats the H_2_ molecule as an averaged, nonrotating target, it is shown that the reduced dimensionality results are in good accord with the four dimensional results as long as the HeH^+^ molecule is not initially rotationally excited. By further comparing the present rate coefficients with those for HeH^+^–H and for HeH^+^–He, we demonstrate that H_2_ molecules are the most effective collision partners in inducing rotational excitation in HeH^+^ cation at interstellar temperatures. The rotationally inelastic rates involving *o*-H_2_ and *p*-H_2_ excitations are also obtained and they turn out to be, as in previous systems, orders of magnitude smaller than those involving the cation. The results for the H_2_ molecular partner clearly indicate its large energy-transfer efficiency to the HeH^+^ system, thereby confirming its expected importance within the kinetics networks involving HeH^+^ in interstellar environments.

## 1 Introduction

Since the detection of HeH^+^ in the planetary nebula NGC 7027 by Güsten et al.,^1^ further followed by its confirmation by Neufeld et al.,^2^ the interest in the mechanisms of possible formation and destruction of HeH^+^ in stellar and interstellar conditions has clearly increased within the astrochemical community. Novotný et al.^3^ have recently measured the recombination rate for HeH^+^ using an ion storage ring and found that such rates, leading to the destruction of HeH^+^, are much smaller than expected from earlier investigations. These new findings, therefore, suggest that this cation should be more abundant than previously expected in astrochemical environments: from molecular clouds and circumstellar envelopes to the stage of the recombination era in the early universe modelings. Forrey et al.^4^ and Courtney et al.^5^ have taken stock of the factors involved in the formation of HeH^+^ in the planetary nebula as well as in the early universe. They conclude that the abundance of HeH^+^ is at least 3 orders of magnitude larger than what was predicted earlier for redshifts near *z* = 20. Its inclusion within enlarged chemical networks of such modeling should then be considered and therefore a revival of the dynamical analysis used to assess its collision efficiency when operating as an energy dissipation partner with other chemical species like He, H, and H_2_ has become more relevant. One should note that all these partners are in fact considered by several studies to be present in relatively large abundances within interstellar medium (ISM) environments.^6,7^ Hence, the present study will be focused on the study of the inelastic collisions between the title cation and He, H, and H_2_ as interstellar partners of the latter.

It is well-known that the interaction of HeH^+^ with H_2_ is also a reactive interaction and therefore its relative importance in the presence of subreactive, energy-transfer collisions should be considered. For example, using the flowing afterglow method, Adams et al.^8^ had estimated the rate coefficient (*k*) for the reaction,
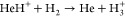
1to be ≥3.5 × 10^–11^ cm^3^ molecule^–1^ s^–1^ for the reactants at 200 K (∼0. 017 eV). Using an ion trap in an ion source mass spectrometer, Ryan and Graham^9^ measured it to be (1.4 ± 0.2) × 10^–9^ cm^3^ molecule^–1^ s^–1^ at a mean ion energy of 0.1 eV. By investigating crossed ion beam-neutral gas collisions, Rutherford and Vroom^10^ estimated the *k* value to be 2.3 × 10^–9^ cm^3^ molecule^–1^ s^–1^ at a mean energy of 0.3 eV. They reported the reaction cross section to be 38 Å^2^ at a relative translational energy (*E*_trans_) of 0.3 eV, decreasing to ∼1 Å^2^ around *E*_trans_ = 6 eV. They also found the decay in the reaction cross section to be inversely proportional to the relative velocity (*v*_rel_) of the reactants for *E*_trans_ in the range 0.4–2 eV, as predicted by Gioumousis and Stevenson.^11^

Using a drift tube mass spectrometer, Johnsen and Biondi^12^ determined *k* to be ≥10^–9^ cm^3^ molecule^–1^ s^–1^ at 300 K. Subsequently, Orient^13^ measured the *k* value to be (1.26 ± 0.16) × 10^–9^ cm^3^ molecule^–1^ s^–1^ at 300 K and independent of the mean kinetic energy in the range 0.04–0.3 eV. This was somewhat less than the value of 1.8 × 10^–9^ cm^3^ molecule^–1^ s^–1^ predicted by the Langevin model.^13^

It will be shown by the present work (see below) that the rate coefficients for the subreactive, rotationally inelastic processes involving HeH^+^···H_2_ collisions under interstellar conditions, which are the processes of interest in the present study, are of comparable magnitude to that of the rate coefficients for the reaction of eq 1 discussed in the previous paragraph. We can therefore argue that the concurrent collision energy-transfer processes are still occurring with a sufficient flux distribution into their inelastic channels to make it significant to be studied alone for a quantitative evaluation of the inelastic rates.

With the same token, the work of Desrousseaux and Lique^14^ on the reactive system HeH^+^ + H found that the purely inelastic channels of rotational excitations became as important as the reactive proton-exchange process once the temperature was above about 50 K. That work also showed that to evaluate the inelastic process alone did not yield rate coefficients which were much different in size from those obtained in the presence of the reactive channels. In the present example our data will be presented well above that temperature and therefore are still expected to remain of significant size within the network of energy-transfer processes involving the present partners even when reactive channels were to be included.

It is also important to note here that in much later work on the present system, while discussing the stability of various HeH_3_^+^ species, Zicler et al.^15^ further estimated the radiative association rate coefficient (*k*_RA_) for the process
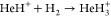
2finding them to rise from 3.10 × 10^–19^ cm^3^ molecule^–1^ s^–1^ at 10 K to 2.01 × 10^–18^ cm^3^ molecule^–1^ s^–1^ at 100 K and to decline to 7.10 × 10^–19^ cm^3^ molecule^–1^ s^–1^ at 500 K. Thus, we can safely assume that this destruction channel is occurring with a much lower probability in comparison with the inelastic processes which we shall discuss below.

The interaction of HeH^+^ with H_2_ to give HeH_3_^+^ and possibly He and H_3_^+^ have also been considered by Zicler et al.^15^ in the paper cited above, but the corresponding inelastic collisions leading to rotational or rovibrational energy transfer have not been included explicitly. It is therefore the main scope of the present work to investigate such inelastic collisions between the title cation and neutral H_2_ as another significant energy-transfer step which needs to be made available from accurate calculations. We shall therefore omit discussing here any further the chemical processes that could competitively interfere with either of the collision partners, since their rates are found to be smaller than those we have obtained in the present study reported below.

More specifically, in the present work we intend to investigate in some detail, and to our knowledge for the first time, how efficiently HeH^+^ could change its rotational energy content when it interacts with the neutral ortho- and para-H_2_ molecules that are known to be extensively present in the same environments. We will therefore show how this new channel for energy flow could enter the dissipation networks by undergoing purely inelastic (rotational) collisions involving either of the molecular partners:
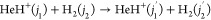
3where we shall consider the neutral partner as being either *p*- or *o*-H_2_.

Very early work on the collision of two rigid-rotor molecules was carried out by Green et al.^16^ for the H_2_···H_2_ system, where they found that para-H_2_(*j* = 0) was comparable in efficiency with He as the neutral partner. Their reported rate coefficients for Δ*j*_1_ = 2 transitions in H_2_ were in the range from 10^–13^ to 10^–10^ cm^3^ molecule^–1^ s^–1^ for temperatures from 500 to 6000 K. Subsequently, Quéméner and Balakrishnan^17^ found from their quantum mechanical calculations on the accurate 4D-PES Boothroyd et al.^18^ that the corresponding rate coefficients for rotational excitations of H_2_ in collision with H_2_(*j* = 0) were indeed extremely small (e.g., in the range from 10^–18^ to 10^–14^cm^3^ molecule^–1^s^–1^) in the *T* range from 40K to 120 K. More recently Klos and Lique^19^ have analyzed the CN^–^···H_2_ ionic system and found that both *p*- and *o*-H_2_ were equivalent in exciting rotational transition in the CN^–^ anion. Further, very recent work on a positively charged system like NH_2_^+^ interacting with H_2_ Balanca et al.^20^ also found the two ortho- and para- variants of the latter partner to exhibit similar efficiency in their collisional excitation of the rotational levels of the cation.

The best available theoretical value for the dipole moment (μ) of HeH^+^ is 1.66 D as given by Pavanello et al.,^21^ while the rotational constant *B* = 33.526 cm^–1^ as quoted in Mueller et al.^22^ These are the values employed in the present calculations for the interaction with the H_2_ molecule kept at its equilibrium geometry of 0.74415 Å.

Funke et al.^23^ carried out perhaps the first SCF calculation for the reaction in eq 1 and showed it to be exothermic by 2.3 eV. However, they did not discuss the presence of any minimum for HeH_3_^+^. Using a larger basis set and a limited configuration interaction calculation, Benson and McLaughlin^24^ obtained an exothermicity of 2.6 eV for the reaction 1. However, they also did not specifically discuss any minimum energy configuration for HeH_3_^+^. Using valence bond configuration interaction functions, Poshusta et al.^25^ reported HeH_3_^+^ to be stable by 0.44 eV (without specifying the reference species). In a subsequent calculation, Poshusta and Agrawal^26^ reported the stability of HeH_3_^+^ to be about 0.02 eV, with respect to well separated He and H_3_^+^. McLaughlin and Thompson^27^ carried out some exploratory trajectory calculations on the potential energy surface (PES) for *C*_2*v*_ geometries computed by Benson and McLaughlin^24^ and found that the larger proportion of trajectories led also to internal rovibrational excitation of product H_3_^+^. Recently, Zicler et al.^15^ have carried out complete active space-second order perturbation theory calculations and shown that HeH_3_^+^ is stable by 2.68 eV relative to the well separated HeH^+^ and H_2_ in their equilibrium geometries and stable by 0.05 eV relative to the well separated He and H_3_^+^.

Unfortunately, none of these studies examined the anisotropy of interaction between HeH^+^ and H_2_ at distances large enough for a realistic computational treatment of rotational energy transfer dynamics to take place. We have therefore carried out new calculations of an extensive set of ab initio points for the relevant interaction, thereby generating a new PES which is focused on the purely inelastic collisions involving either of the molecular partners, without considering in this study the vibrational or reactive channels.

The colliding molecules were therefore taken to be at their fixed internuclear distances given by their equilibrium values (see below) and we have computed the ensuing 4-dimensional (4D), rigid-rotor (RR) PES that describes the molecule–molecule interaction.

In order to have quantitative information on the relative efficiency of a variety of energy-changing processes involving the internal level structure of HeH^+^ when it interacts with other “chemical” partners, it is important to find out how one like the neutral hydrogen molecule can affect internal energy redistributions in the cation within the general cooling paths that followed the recombination era.^6^ To this end, our present results will be compared with those already available for (HeH^+^–H)^14^ and for (HeH^+^–He)^28^ collisions leading to rotational excitations/de-excitations of the same cation. As we shall show below, one of the important findings of our present study is that the neutral hydrogen molecule turns out to be the most efficient collision partner in causing rotational excitations/de-excitations in HeH^+^ and therefore the ensuing inelastic rate coefficients should be included in kinetic networks which model chemical evolution in general ISM environments.

The newly constructed ab initio PES for the rigid rotor HeH^+^–H_2_ interaction is described in Section 2.1 and compared with the one involving neutral He atoms in Section 3.2. The method adopted for computing inelastic cross sections and the present results are described in Section 3.1. The possibility of reducing the dimensionality of the system is discussed in Section 3.2 and the results obtained using a reduced dimensional PES are presented in Section 3.2 as well, while Section 3.3 presents and discusses the behavior of the computed inelastic rate coefficients. The following Section 3.5 compares the present findings with earlier results for H and He. The differences found in the dynamics involving either *p*- or *o*-H_2_ will be discussed in the next section while a summary of our findings and their implications for chemical network modelings will finally be presented in Section 4.

## 2 Computational Methods

### 2.1 HeH^+^–H_2_ 4-D PES between two Rigid Rotors

Extensive ab initio calculations were carried out using the MOLPRO suite of quantum chemistry codes: see refs (29,30). The full dimensionality of the two-molecular partners interaction energy surface is given by six coordinates in a Body-Fixed (BF) representation. However, since the HeH^+^ bond distance, as well as that for H_2_, are kept fixed at their equilibrium values of 0.774 Å and 0.74415 Å, respectively, one reduces the dimensions to 4 coordinates in the same BF frame. The post-Hartree–Fock treatment was carried out using the CCSD(T) method^31,32^ and complete basis set (CBS) extrapolation was attained using the aug-cc-pVTZ, aug-cc-pVQZ, and aug-cc-pV5Z basis sets^33,34^ in four dimensions. The basis-set-superposition-error (BSSE)^35^ was corrected for all the calculated points so that the full interaction was obtained with the inclusion of the BSSE correction.

**Figure 1:**
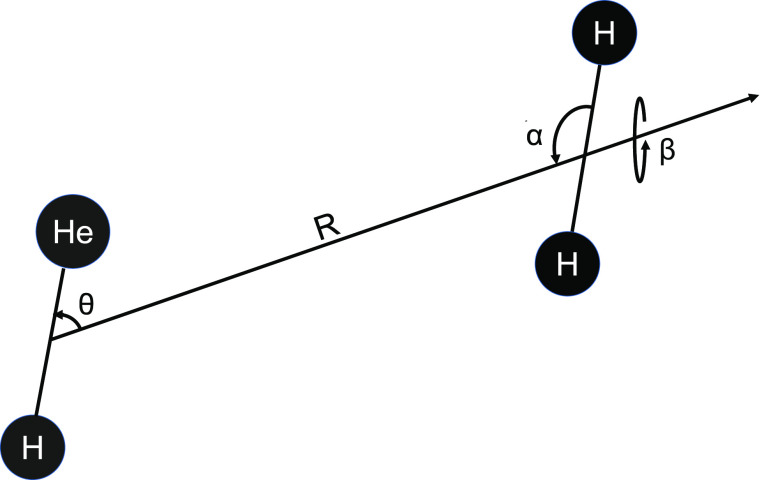
4D set of Body-Fixed coordinates for the HeH^+^–H_2_ system. The two bond distances are given on a comparable scale. See main text for the definition of the coordinates.

The HeH^+^–H_2_ system is now spatially defined by the knowledge of three angles: θ, α, β, and of *R*, the distance between the HeH^+^ and H_2_ centers of mass, all shown in Figure 1, where we report a pictorial representation of the set of coordinates employed for the 4D-RR-PES.

The θ and α angles are polar angles with respect to *R* while β is the dihedral angle. The 4D-RR-PES (*R*, θ, α, β) was calculated using relative distance radial points from 1.0 to 12.0 Å along *R*. For the polar angle θ, we calculated a range of values between 0° and 180° at intervals of 10°. The values for the α rotational angle were chosen to be 0°, 30°, 60°, 90°, 120°, and 150° for the previous selections of *R* and θ values. Note that α = 180° is equivalent to 0°. The values for β were then chosen to be 0°, 30°, 60°, and 90°. The total number of points computed for the whole surface was 16 086.

It is interesting and instructive to look at a representation of some specific “cuts” of the 4D-RR-PES to assess more directly the relative energy effects of changing the dihedral and the polar angles. This analysis will later help us in guiding the choices for the dimensionality reduction of this PES to 2D.

**Figure 2:**
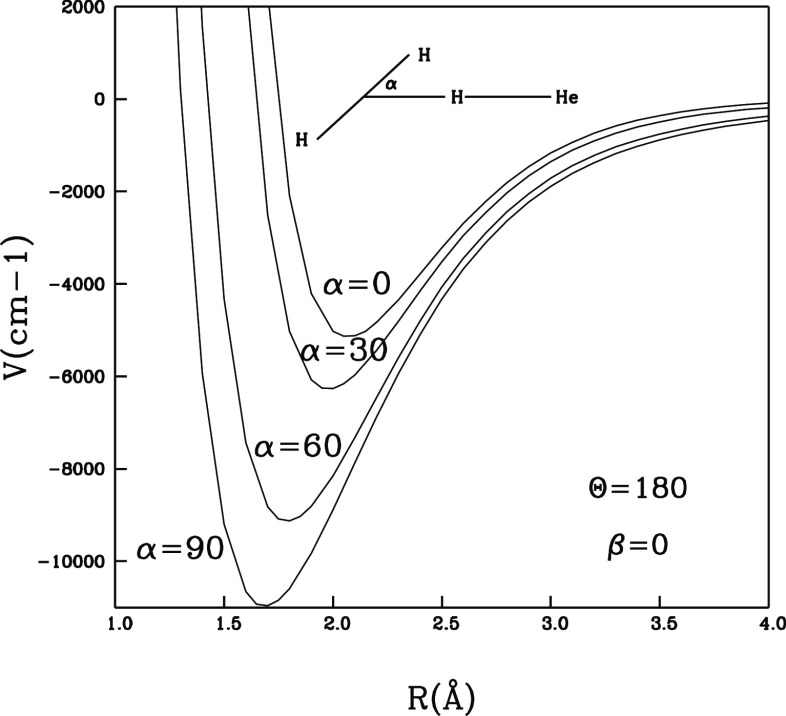
Interaction energy variations as a function of the polar angle α for fixed values of the θ and β angles as shown in the inset.

**Figure 3:**
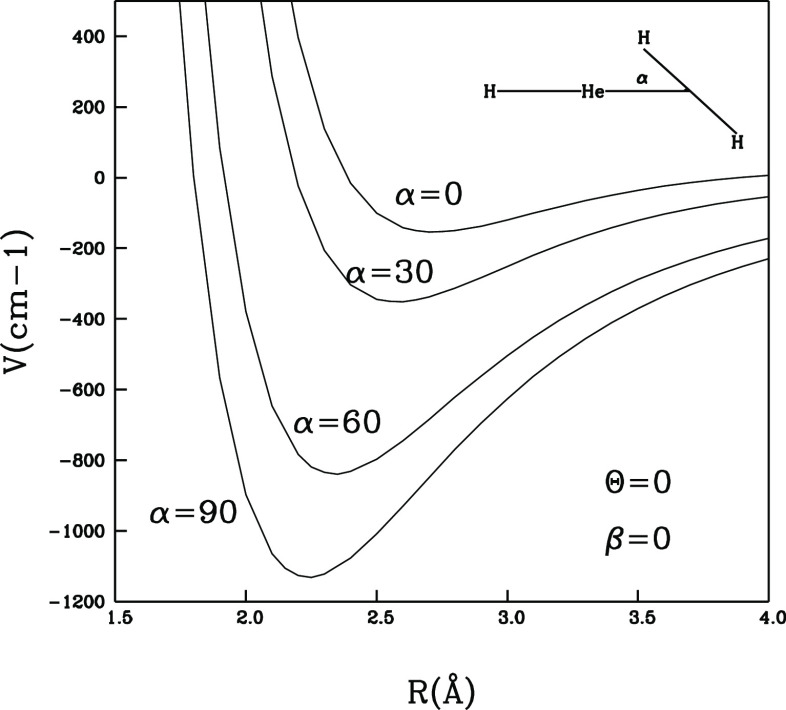
Interaction energy variations as a function of the polar angle α for fixed values of the θ and β angles as shown in the inset. The approach of the H_2_ partner is now at the He-end of the cation.

**Figure 4:**
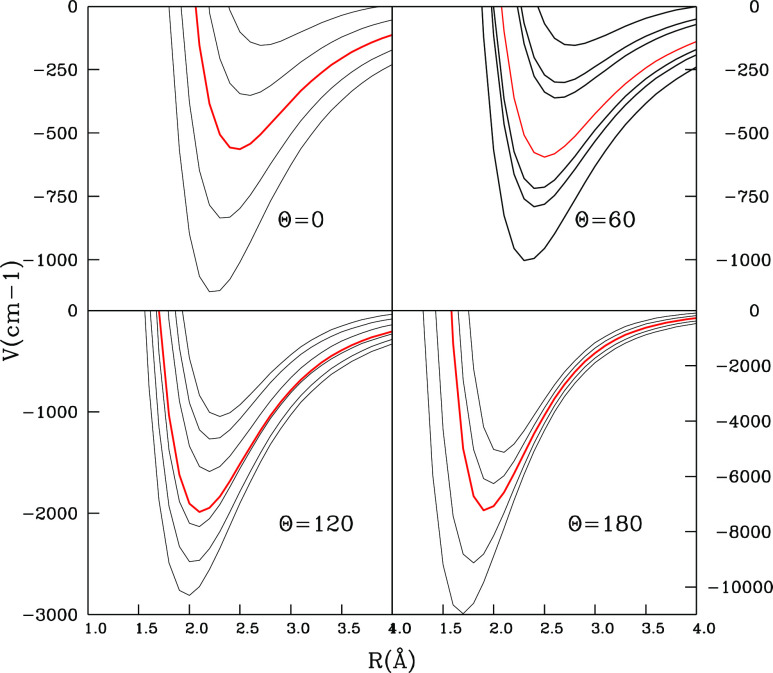
Comparison of the interaction energy variations for four different selections of the polar angle θ while the dihedral angle β is kept fixed at 0°. The different black curves within each panel report variations of the polar angle α while the red curves show the weighted average of all the calculated α variations within each panel. For θ = 0° and 180° four α values, are shown by the curves from top to bottom in each panel: 0°, 30°, 60°, and 90° so there are 5 plots in each frame. In the panels with θ = 60° and 120°, (β = 0°) the six α values now run from 0°(top) to 150°, 30°, 120°, 60°, and 90° (bottom). Note that the configuration with α = 180° is the same as that with α = 0°.

A pictorial selection of such views is given in the three figures, i.e., in Figures 2, 3, and 4, reported below. The following can be said about the results we obtained:

(i) by looking at the data in Figure 2, we see that the chosen in-plane configuration of the H_2_ molecule approaching the H-end of the cation generates large interaction energy changes as the polar angle α is varied. Thus, we expect that this angle variation plays an important role in altering the overall interaction potential;
(ii) on the other hand, the same in-plane approach as before, but this time on the He-end of the cation as reported by Figure 3, indicates that varying the polar angle α has a markedly smaller effect on the variation of the interaction potential on that side of the ionic target;
(iii) when the dihedral angle is varied and several out-of-plane arrangements are analyzed by changing β for the same range of α values as those presented in Figures 2 and 3, essentially marginal differences in the range of energy variations are found by the calculations. In other words, changes of the dihedral angle β have very little effect on the energy variations already seen in the previous two figures;
(iv) the data in the four panels of Figure 4 give us different views of the energy changes along different cuts of the 4D-RR-PES of this study. For the polar angle θ = 0° and 180° there are 4 curves in each of the two panels presented in Figure 4 while those marked in red report in all the panels the corresponding energy averages of all (α, β) pairs considered in the calculations. For the two panels reporting θ = 60° and 120°, we have chosen β = 0° and six different α values. From top to bottom they are α = 0°, 150°, 30°, 120°, 60°, and 90°, which is always the lowest energy configuration in both those panels. It may be noted that the configuration with α = 180° is the same as that with α = 0°.
(v) from the analysis of the large variety of cuts which we evaluated, we see that the minimum energy structure of the complex in 4D is given by the lowest-energy set of orientations reported in Figure 2 for the polar angle α = 90° and the radial distance *R* around 1.72 Å. This result is in line with previous calculations involving the present system.^15^

### 2.2 Fitting the 4D PES with ML-ANN Methods

In the present study, machine learning (ML) methods were used to interpolate the computed ab initio potential energy values for the system. The ML methods involve training an algorithm to learn from input data, consisting of a set of values of a function for a pregiven set of input data, and predicting the outcome of the function for a (new) set of input values for which the function values are not (necessarily) known. Two types of ML methods—Gaussian process for regression (GPR) and artificial neural networks (ANN) were explored initially to represent the HeH^+^–H_2_ 4D PES. What is reported here is the successful use of the ANN approach.

ANNs were inspired by the connections of neurons in brains, and their ability to do complex networking and to recognize patterns. They are constructed using nodes (analogous to neurons) as an input layer, hidden layers and an output layer.^36,37^

**Figure 5:**
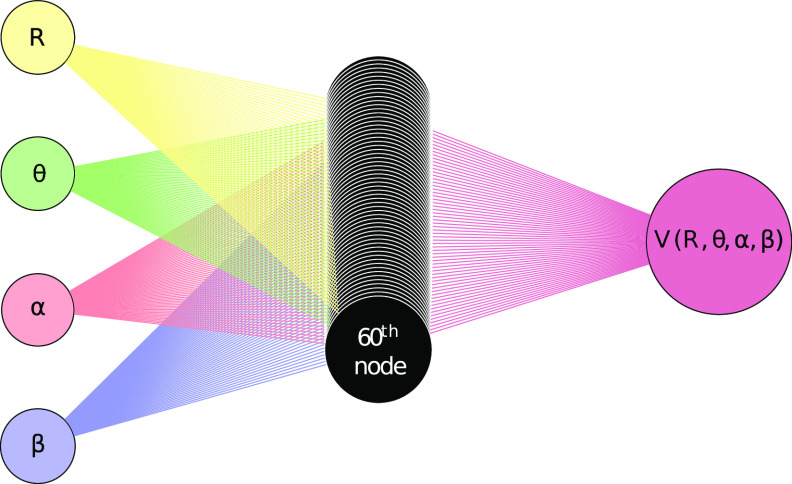
Neural Network design for the four dimensional PES function.

The 4D PES for HeH^+^ + H_2_ was then mapped using the ANN method. MATLAB^38^ software was used to train the ANN, and also to generate the subroutine for the given network. We used a shallow network consisting of one hidden layer with 60 nodes as illustrated in Figure 5. A modified logistic sigmoid function of the form,
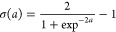
4where *a* is the sum of inputs to any given node, and σ(*a*) is the output of that node, was used as the transfer function of choice. Training was carried out until the root-mean-square deviation (RMSD) values reached acceptably low levels, after which the fits were tested for the removal of overfitting by using the data points not included in the initial training set.

Initially, low energy data points (a total of 14 741 points) were used for training. Five types of fitting were performed with different choices of data sets. In data sets 1 and 2, the ab initio points were sampled randomly using 70% and 98% of the data, respectively. In set 3, the points were chosen using a uniform grid consisting of 50% of the data and including boundary data points. Sets 4 and 5 were chosen using Latin Hypercube Sampling (LHS) Schemes of different sizes.

The accuracy of the different ANN fits was checked by computing the RMSD values listed in Table 1 and by plotting the residuals for the entire range of the potential.

**Table 1:** Summary of Different Sampling Schemes and Their Corresponding RMSD Values of the Trained ANN^a^

data set	sampling	no. of training points	maximum error (cm^–1^)	RMSD (cm^–1^)
1	random (70%)	10 318	208	11.1
2	random (98%)	14 446	–158	8.7
3	grid	9026	150	10.1
4	LHS1	2124	5462	107.1
5	LHS2	4055	–16 027	244.0
6	random (98%)+	15 610	–934	35.4

a “+” symbol for Set 6 signifies that it included additional higher energy data points.

All fits except Set 6 suffered from overfitting for different combinations of α and β. Overfitting occurs when the RMSD of a fit is low but the fit does not vary smoothly over all the variables, hence yielding erroneous values where the value of the function is not known.

For Set 6 we added higher energy points to the data set, taking the total number of points to 15 928 and obtained another ANN fit by randomly choosing 98% of the data for training.

This fit had an overall RMSD of 35.4 cm^–1^ (which is about 0.1 kcalmol^–1^) and a maximum error of −934 cm^–1^ for *R* = 1.6 Å at θ = 30°, α = 180°, β = 0°, and *V* = 7597.80 cm^–1^. This is in line with similar results found with other types of fitting of initial raw ab initio points.

**Figure 6:**
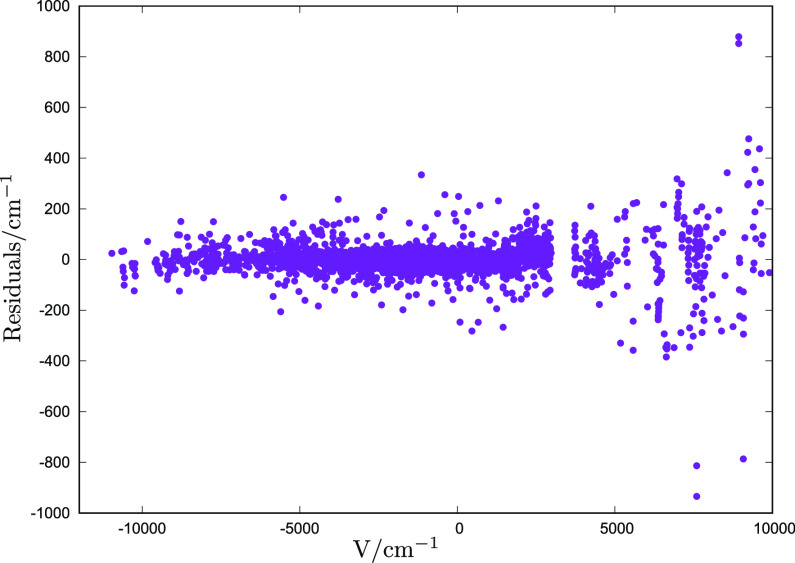
Residuals plotted as a function of *V* for the entire fit using the data set 6.

**Figure 7:**
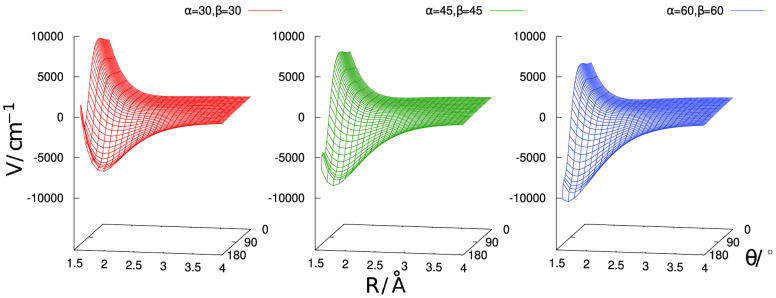
*V* as a function of *R* and θ for different combinations of α and β. The red plot (left) and the blue plot (right) are for values of α and β where data are available, and the green plot(center) is for α = 45° and β = 45°, where no data were available.

The residuals near the minimum energy region of the PES are less than 200 cm^–1^, when compared to the well depth of ∼10 000 cm^–1^, as illustrated in Figure 6. This fit also avoided overfitting, i.e., the value of *V* varied smoothly over α and β variables as illustrated in Figure 7.

**Figure 8:**
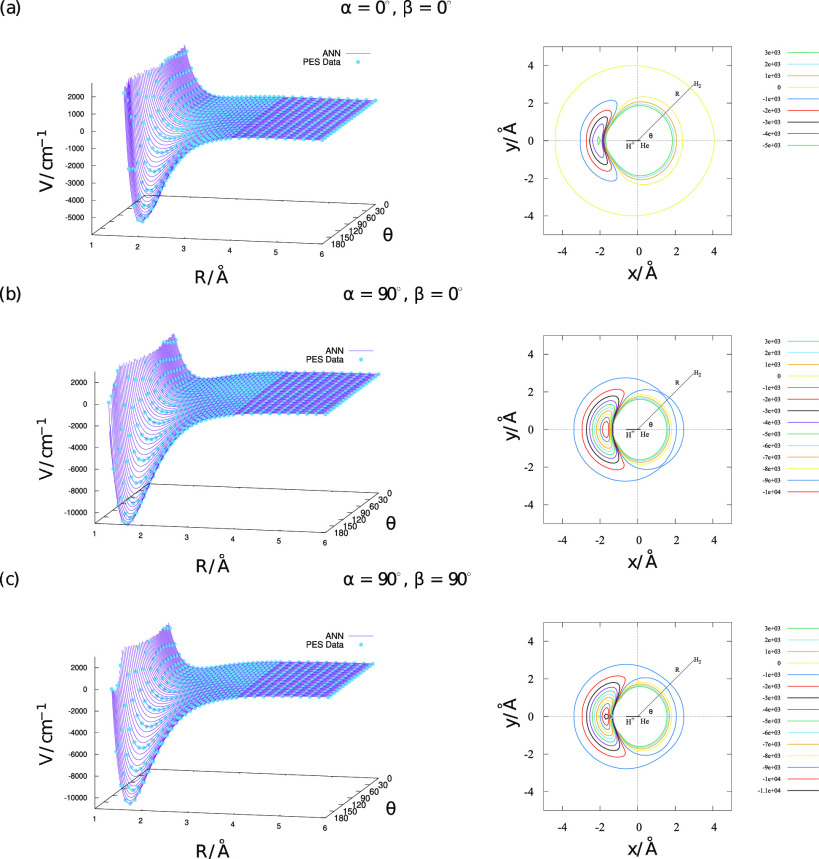
Plot of the PES (left) obtained from ANN predictions and ab initio data (blue dots) plotted against *R*,θ. The respective potential energy contour plots (right), for different sets of α and β.

The PES and the corresponding contours for three different sets of fixed values of α and β are plotted as a function of *R* and θ in Figure 8. It can be seen that the ANN fit reproduces the ab initio data accurately without overfitting.

### 2.3 Fortran Routine for the Final ML-ANN Fit

MATLAB was used to produce a stand-alone C++ code for the ANN fit with Random (98%) sampling (Set 6), which contained all the optimized weights and biases. It was further converted to FORTRAN programming language, so that it could be used within the scattering code MOLSCAT^39^ that was employed for the present calculations. The same routine is included in the Supporting Information (SI).

Since the input data for the 4D PES was available up to *R* ≤ 12 Å, we also added the asymptotically correct long-range potential *V*_LR_ for the needed larger *R* values:
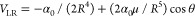
5where α_0_ = (α_∥_ + 2α_⊥_)/3 and α_2_ = α_∥_ – α_⊥_. Using the results of α_∥_ = 6.38049 and α_⊥_ = 4.57769 au for *r* = 1.4 au (0.7408 Å) of H_2_ reported by Kolos and Wolniewicz,^40^ α_0_ = 5.1786 au. It is worth reiterating that θ = 0 corresponds to the He end of HeH^+^ and θ = 180° to the H end. (*r*(HeH^+^) = 0.774 Å, *r*(H–H) = 0.744155 Å).

For HeH^+^–He, μ = 1.66 D.^41^ For a unit (+) and a unit (−) charge separated by 1 Å, dipole moment = 4.8 D. Taking the Coulombic charge to be 1 in au, 1.66 D = [1.66/(4.8 × 0.529167)] = 0.6535 au. Therefore,
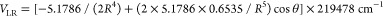
6

The switch between the ML-ANN fitted PES and the asymptotically correct *V*_LR_ was made using the switching function *f*_s_(*R*) = 1/(exp ((*R* – *R*_0_)/*δR*) + 1) such that
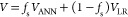
7with *R*_0_ = 11.0 Å and *δR* = 0.5 Å for R = 11–12 Å. We set *V*_ANN_ to zero for *R* > 12 Å.

## 3 Results and Discussion

### 3.1 HeH^+^–H_2_ 4-Dimensional Quantum Dynamics

In the standard procedures employed for solving the Coupled-Channel (CC) scattering equations, it is usually convenient to expand, at each value of *R*, the interaction potential *V* (*R*,θ,α,β) into orthogonal angular functions.^42−45^ In the specific case of the present work involving the scattering of two linear rigid rotors, one can in principle write down a standard uncoupled, double expansion^45^ involving the variables defined in the previous section:
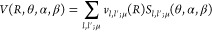
8which we will not discuss here in more detail and where the (*l*,*l*′;μ) indexes run independently of each other (see more below). Indexes *l*, *l*′ are associated, respectively, with the rotational motions of HeH^+^ and H_2_. In eq 8, the homonuclear symmetry of H_2_ forces the index *l*′ to be even.

In the present calculations, the MOLSCAT code starts by employing the ML-ANN fit of the potential to describe the interaction and then expands that intermolecular energy surface in terms of double Legendre polynomials as described in ref (39) which follows instead the coupled representation of the final potential function:
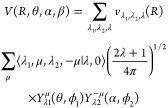
9

Although the expansion is in terms of a product of spherical harmonics involving two azimuthal angles ϕ_1_ and ϕ_2_, the change in sign of μ between the two spherical harmonics terms makes sure that the potential depends only upon ϕ_1_ – ϕ_2_ = β. The angle variables in eq 9 are therefore the same as in eq 8. In this case, as it occurred in the previous equation, the homonuclear symmetry of H_2_ forces the index λ_2_ to be even. The two types of expansions are equivalent and are connected by a unitary transformation. The present calculations have therefore employed the coupled expansion of eq 9, where the index λ is always given by the sum of the other two indexes λ_1_,λ_2_, in contrast with the μ index in the eq 8.

**Figure 9:**
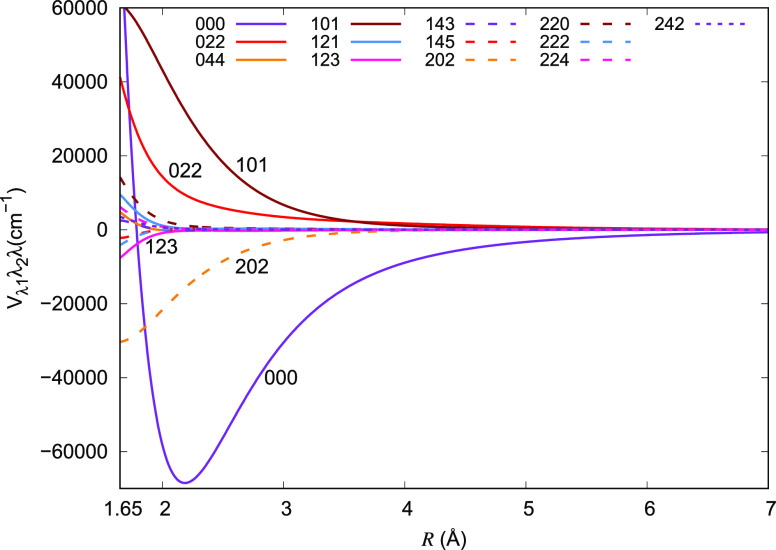
Comparison of the multipolar expansion coefficients generated by the scattering code MOLSCAT for the 4D RR-PES for the HeH^+^···H_2_ system, discussed in this work.

Some of the resulting coupled expansion coefficients *V*_λ_1_λ_2_λ_ which were produced within the MOLSCAT code are plotted as a function of *R* in Figure 9 and show clearly that the isotropic part (000) of the potential is the dominant term, as it is expected for the present interaction. The other significant terms are 101, 202, 121, 123, 022, and 044, following the labeling of the double multipolar notation. The smoothness of the final curves testifies for the good quality of the present ML-ANN fit. It is also worth noting at this point that the second coefficient in each product has only even terms because of the symmetry of the homonuclear partner rotor (H_2_).

Since the standard time-independent formulation of the Coupled Channel method for studying rigid rotor–rigid rotor (RR) quantum collisions is well documented in the literature^39^ and the MOLSCAT software is also readily available^39^ for computing the state-to-state inelastic transition probability values for given initial conditions, we only list some of the input parameters employed when using that code:

JTOTL = 0, JTOTU = 100, BE = 33.526, 60.8, J1MIN = 0, J1MAX = 10, J2MIN = 0, J2MAX = 6, J2STEP = 2, L1MAX = 10, L2MAX = 4, IHOMO2 = 2, NPTS(1) = 21, NPTS(2) = 21, NPTS(3) = 25, for *p*-H_2_ as the collision partner. For *o*-H_2_ (J2MIN = 1, J2MAX = 7) was used.

Briefly, we shall consider below energy-transfer collisions between HeH^+^ and H_2_ molecular partners, taken to be in their defined initial rotational states (*j*_1_ and *j*_2_, respectively), for a range of relative collision energies *E*_trans_ (1 to 2000 cm^–1^) and for total angular momentum (*J*) values up to 100, by which convergence of the inelastic cross sections is achieved within 10% of their values. The chosen interaction potential was obviously the one described in the previous subsection.

**Figure 10:**
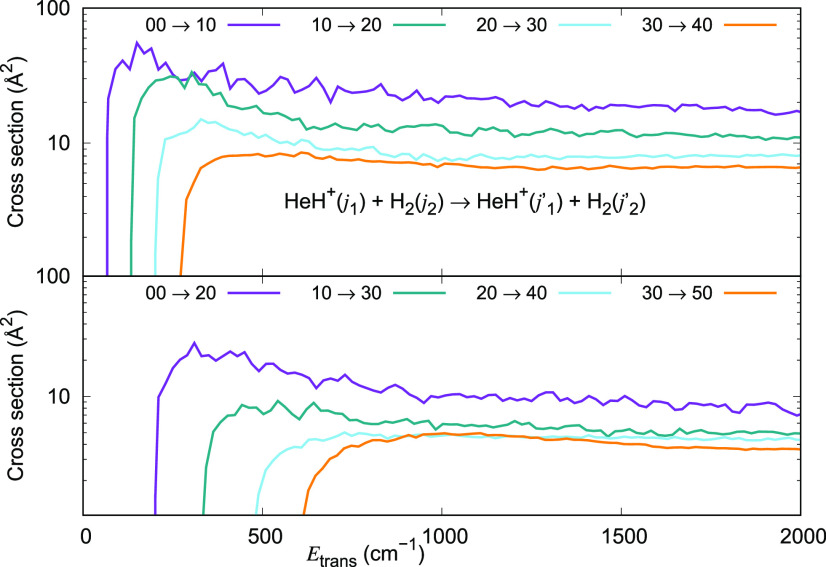
Computed excitation cross sections for a series of inelastic processes generated using the 4D RR-PES for the HeH^+^(*j*_1_)···para-H_2_(*j*_2_ = 0) system, discussed in this work. The upper panel reports excitation transitions with Δ*j*_1_ = +1, while the lower panel indicates excitation processes with Δ*j*_1_ = +2. The value of the rotational index *j*_2_ in the H_2_ partner is kept fixed and equal to 0.

The computed integral inelastic cross section values as a function of relative translational energy (σ(*E*_trans_)) for HeH^+^ (*j*_1_ = 0, 1, 2 and 3) colliding with *p*-H_2_ (*j*_2_ = 0) plotted in Figure 10 (upper panel) show clearly their rise from the energetic threshold at 2*B*, 4*B*, 6*B*, and 8*B*, respectively for Δ*j*_1_ = +1 transitions in HeH^+^ while H_2_ remains unexcited. For *j*_1_ = 0, the initial rise in the excitation function is followed by a maximum around 60 Å^2^ and there are several oscillations in the curve showing clearly the existence of scattering resonances. The cross section declines to a value of 18 Å^2^ at *E*_trans_ = 2000 cm^–1^. Plots for σ (*j*_1_ = 1, 2, and 3) all show a similar behavior. That is, the rise from the threshold is followed by a maximum in the curve and an eventual leveling off around *E*_trans_ = 2000 cm^–1^. The oscillations in the excitation function decrease as *j*_1_ is increased from 0 to 1 to 2 to 3. The significant drop in the inelastic cross section with an increase in *j*_1_ is also evident in this figure, showing clearly that they decrease with an increase in the energy gap (2*B*, 4*B*, 6*B*, and 8*B*) between the initial and final states of HeH^+^, as is usually expected.

The lower panel in Figure 10 shows the excitation cross sections for Δ*j*_1_ = 2 for initial *j*_1_ = 0, 1, 2, and 3 and *j*_2_ = 0 over the same energy range. Since the energy gap in these transitions is larger, the cross section values for Δ*j*_1_ = 2 transitions are consistently smaller than those for the corresponding Δ*j*_1_ = 1 transitions. Otherwise, the excitation functions behave similarly as in the upper panel. Once more, there are noticeable oscillations in the σ(*E*_trans_) curve for *j*_1_ = 0 at lower energies. These oscillations decrease in amplitude on going to rotational excitations from *j*_1_ = 1 and become essentially nonexistent for *j*_1_ = 2 and 3. The decrease in the excitation cross section with an increase in the energy gap between the initial and final states is again evident in the curves plotted in the lower panel, particularly at lower energies.

**Figure 11:**
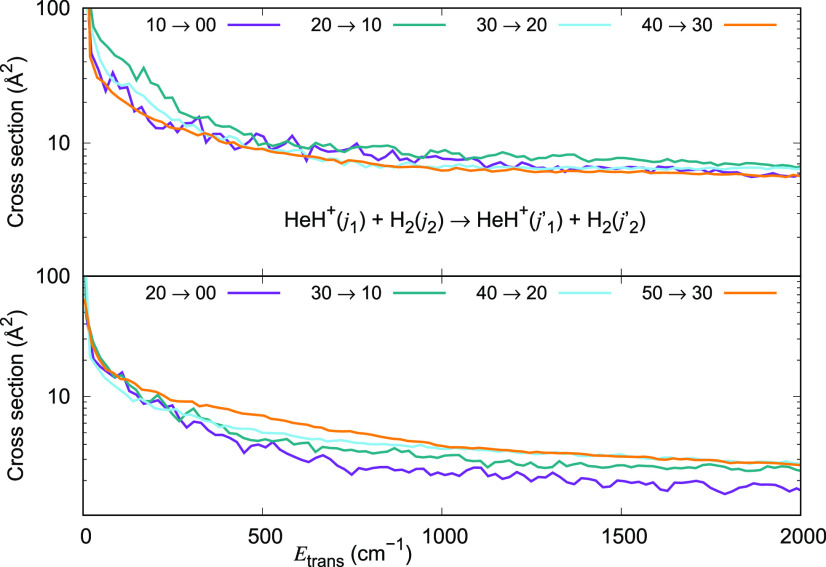
Computed de-excitation cross sections for a series of inelastic processes generated using the 4D RR-PES for the HeH^+^(*j*_1_)···para-H_2_(*j*_2_ = 0) system, discussed in this work. The upper panel reports de-excitation transitions with Δ*j*_1_ = −1, while the lower panel indicates de-excitation processes with Δ*j*_1_ = −2. The value of the rotational index *j*_2_ in the H_2_ partner is kept fixed and equal to 0.

The inelastic cross section values obtained from our calculations for different initial (*j*_1_, *j*_2_) states of the collision partners but for Δ*j*_1_ = −1 and Δ*j*_1_ = −2 transitions are now plotted in Figure 11. They show clearly that the de-excitation cross section values become as large as 100 Å^2^ for Δ*j*_1_ = −1 transitions at low energies. They are a factor of 2 smaller for Δ*j*_1_ = −2 transitions, once again indicating the exponential gap relationship between the transition probability and the energy gap between the initial and the final states of the ionic rotor.

It is important to point out at this stage that the excitation (*j*_1_ → *j*_1_^′^) cross section values beyond the threshold region are consistently larger than the corresponding (*j*_1_ ← *j*_1_^′^) de-excitation cross section values because of the larger degeneracy of the final states in the excitation process. Incidentally, our calculations have also confirmed that their values for excitation and de-excitation paths between the same given pair of initial and final states obey the principle of microscopic reversibility.

**Figure 12:**
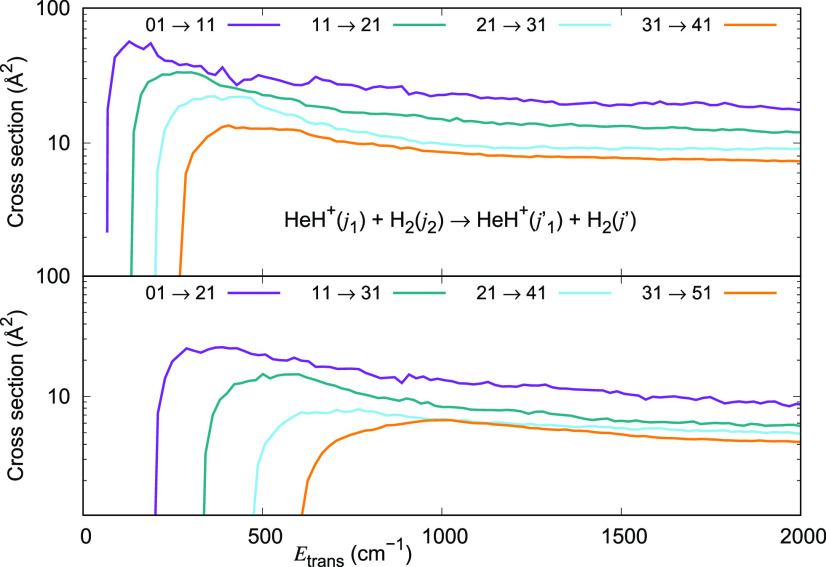
Computed excitation cross sections for a series of inelastic processes generated using the 4D RR-PES for the HeH^+^(*j*_1_)···*o*-H_2_(*j*_2_ = 1) system, discussed in this work. The upper panel reports excitation transitions with Δ*j*_1_ = +1, while the lower panel indicates excitation processes with Δ*j*_1_ = +2. The value of the rotational index *j*_2_ in the H_2_ partner is kept fixed and equal to 1.

To provide more details on the quantum dynamics between the present systems, it is useful to further analyze the different roles of having either the ortho (*j*_2_ = odd) or the para (*j*_2_ = even) forms of the H_2_ partner. We have therefore computed the excitation cross section values for HeH^+^(*j*_1_ = 0, 1, 2 and 3) colliding with *o*-H_2_ (*j*_2_ = 1) in the same energy range as previously discussed in Figure 10 for the *p*-H_2_ case. The results reported in the upper panel of Figure 12 for *o*-H_2_ are remarkably similar in size and energy dependence to those previously reported in the upper panel of Figure 10 for *p*-H_2_. They all show a steep rise in value above the threshold, up to a maximum followed by a reduction in values. The larger the energy gap between the initial and the final states, the smaller the cross section for Δ*j*_1_ = 1 transitions. The results reported in the lower panel of Figure 12 for Δ*j*_1_ = 2 transitions are again remarkably similar in behavior to those reported in the lower panel of Figure 10 for *p*-H_2_.

Although our calculations find that, in general terms, the HeH^+^-*o*-H_2_(*j*_2_ = 1) collisions are comparable to those for HeH^+^-*p*-H_2_(*j*_2_ = 0), a closer comparison of the two sets of results reveals that the values for the former tend to be slightly larger by a few percent than those for the latter, particularly at the higher energies we have considered. This effect will be further discussed when analyzing below the corresponding rate coefficients.

**Figure 13:**
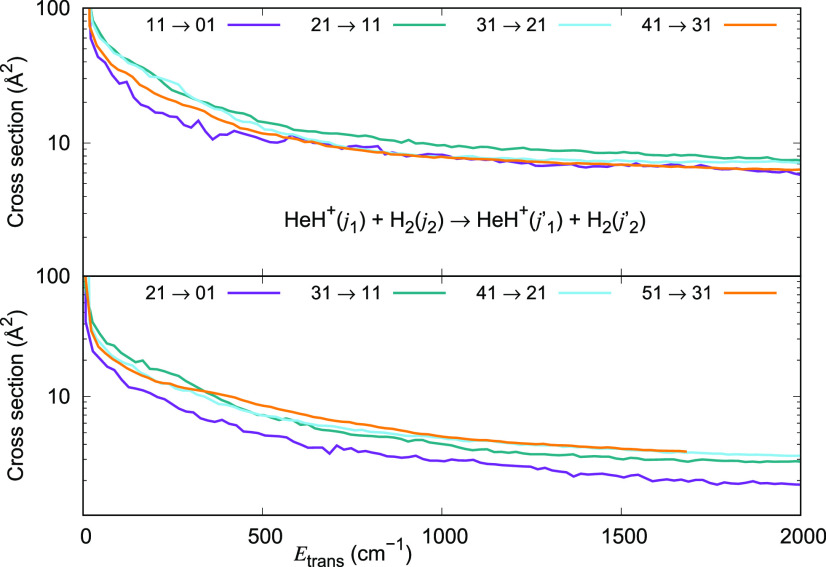
Computed de-excitation cross sections for a series of inelastic processes generated using the 4D RR-PES for the HeH^+^(*j*_1_)···*o*-H_2_(*j*_2_ = 1) system, discussed in this work. The upper panel reports de-excitation transitions with Δ*j*_1_ = −1, while the lower panel indicates de-excitation processes with Δ*j*_1_ = −2.

The de-excitation cross sections for Δ*j*_1_ = −1 transitions in HeH^+^ (*j*_1_ = 1, 2, 3 and 4)-*o*-H_2_ (*j*_2_ = 1) collisions are now reported in the upper panel of Figure 13 and are once more comparable to those for HeH^+^ (*j*_1_ = 1, 2, 3, and 4)-*p*-H_2_ (*j*_2_ = 0) already reported in the upper panel of Figure 11. With the same token, the de-excitation values for Δ*j*_1_ = −2 transitions in HeH^+^ (*j*_1_ = 2, 3, 4, and 5)-*o*-H_2_ (*j*_2_ = 1) collisions reported in the lower panel of Figure 13 are comparable to those for HeH^+^ (*j*_1_ = 2, 3, 4, and 5)-*p*-H_2_ (*j*_2_ = 0) reported in the lower panel of Figure 11.

Cross section values for Δ*j*_1_ = +1 transitions in HeH^+^ (*j*_1_ = 0, 1, 2, and 3) due to collision with *p*-H_2_ (*j*_2_ = 2) are compared with those due to collision with *p*-H_2_ (*j*_2_ = 0) in the Figure S1 reported in the SI. Although the results for *j*_2_ = 0 and 2 are comparable to each other, the differences between the two sets of results become noticeable for higher *j*_1_ states, with an increasing *E*_trans_. Cross section values for *j*_2_ = 2 are consistently higher than those for *j*_2_ = 0, for *j*_1_ = 2 and 3. It is worth reminding the reader that the cross section values are reported in Figure S1 using a log scale along the *y*-axis.

The cross section values for Δ*j*_1_ = +2 transitions in HeH^+^ (*j*_1_ = 0, 1, 2 and 3) in collision with *p*-H_2_ (*j*_2_ = 2) are compared with those from collisions with *p*-H_2_(*j*_2_ = 0) in Figure S2 reported in the SI. Once more, we find the two sets to be fairly similar in size and energy dependence, with the differences becoming more noticeable for higher *j*_1_ states.

To establish quantitatively the relative efficiency of *p*-H_2_ (*j*_2_ = 0) and *o*-H_2_ (*j*_2_ = 1) in causing rotational excitation and de-excitation processes for the title cation is particularly of interest when astrophysical conditions are considered. While Klos and Lique^19^ found no significant difference between ortho and para hydrogen as collision partners for rotational excitations in CN^–^, they pointed out that the trend was opposite to that found for several other interstellar species like CO,^46^ SiS,^47^ HNC,^48^ and H_2_O.^49^ Hence, the strong similarity we found in the present case is in line for what was found before for non-negative partners. This aspect of the dynamics will be further discussed below.

It should be further noticed at this point that the number of systems for which the full 4D PES has been computed and the corresponding full, RR dynamics has been investigated is still fairly limited. There have been, however, several studies where the problem has been simplified to make it computationally more easily amenable to calculation. Especially when *p*-H_2_ (*j*_2_ = 0) is the collision partner, it could be expedient to treat it as a nonrotating partner and make some angular averaging of the full 4D potential to investigate the dynamics under reduced dimensions. This is more reasonable especially when considering that the potentials around H_2_ are usually not strongly anisotropic and *p*-H_2_ does not undergo rotational excitation unless the involved collision energy *E*_trans_ exceeds 120 cm^–1^. We shall therefore discuss below in detail how this dimensionality reduction could be achieved for the present system

### 3.2 Dimensionality Reduction: 2D PES

When we limit our analysis to the para-H_2_ component of the hydrogen molecular partner and take it to be in its ground (*j*_2_ = 0) rotational state, we would be dealing with a nonrotating molecule and focusing only on the rotational state-changing processes involving the cation. It is indeed reasonable to consider the rotational excitation of the latter partner to be the one more likely to occur, since the energy spacing between the *j*_2_ = 0 and 2 levels in para-H_2_ is about 510 K, to be compared with the energy spacing in the HeH^+^ rotor for the *j*_1_ = 0 to *j*_1_ = 1 transition of 96.474 K, i.e., about five times smaller. Under such circumstances, therefore, it would be realistic to consider a simpler interaction potential involving an average over the angles that describe the H_2_ anisotropy within the complex of the two molecular partners. The latter molecule would then be treated as a structureless object with an anisotropic interaction with the cation.

The above simplification corresponds, if starting from the uncoupled, double-multipolar expansion of the 4D potential as given in the previous Section 3.1 via eq 8, to retain only the leading terms of that equation, as discussed in earlier works (e.g., see: Kalugina et al.^50^). This means that only the terms of the double expansion with *l* = μ = 0 are kept to describe the averaged interaction which now only depends on two variables. The resulting potential is now described by only the distance *R* and the polar angle θ, both defined in Figure 1.

Since in the present case the double expansion of the potential was given by the coupled expansion reported by eq 9, we decided instead to carry out the dimension-averaging directly on the original 4D potential values for the four variables in Figure 1 and then re-expand the final results via the usual 2D multipolar expansion as discussed below.

There are, therefore, different ways in which we can carry out a direct dimensionality reduction scheme. In practice, we have tested three different ways for achieving this reduction to assess more closely the modifications on the overall anisotropy of the 4D PES introduced by such reduction:

(i) we can define a quantity called *V*_min_ which is obtained as the minimum energy path for a given (*R*, θ) set of values from all the calculated (α, β) pairs discussed earlier;
(ii) we can define another quantity, called *V*_3_, given as the average of three specifically selected conformations:
  (a) α = 0, β = 0, where the H_2_ bond axis lies along the *R* vector;
  (b) α = 90°, β = 0, where the H_2_ bond axis is perpendicular to the *R* vector with the ensuing complex being planar;
  (c) α = 90°, β = 90°, where the H_2_ bond axis is perpendicular to the *R* vector and also perpendicular to the plane defined via the HeH^+^ bond axis and the distance *R* joining their centers of masses;
(iii) we can further define a third choice of a reduced-dimensionality potential which is called *V*_all_ and is given as the average of 21 different (α, β) pairs (4 pairs for θ = 0° or 180°). The latter is the more comprehensive use of the available initial ab initio points.

It was in fact found here that the models produced by *V*_all_ and by *V*_3_ were very close to each other both in energy and spatial distribution, while the *V*_min_ model produced invariably deeper wells but similarly located as the previous two choices. The behavior presented by the cuts reported in Figure 4 can give an indication on the effects of averaging the potential along different selections of the α angle reported in the four panels, which is the procedure followed by the *V*_all_ dimensionality reduction.

For a more direct, and quantitative evaluation of the spatial anisotropy around HeH^+^, it is useful to expand the extensive 2D grid of points obtained from the three averaging schemes mentioned before in terms of the familiar Legendre polynomials in their standard (*R*,θ) form:
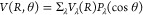
10

We initially obtained 27 multipolar coefficients for the 2-Dimensional rigid-rotor PES (2D-RR-PES), although a varying number of terms were necessary to be included to achieve numerical convergence for the different inelastic processes in the scattering calculations discussed later.

A presentation of the first 10 multipolar coefficients for the anisotropy expansion of the *V*_all_ potential is shown in the Figure S3 reported in the SI. The two inserted panels in Figure S3 further indicate on different energy scales the behavior of the radial coefficients for the deepest interaction well and for the short-range interaction regions.

**Figure 14:**
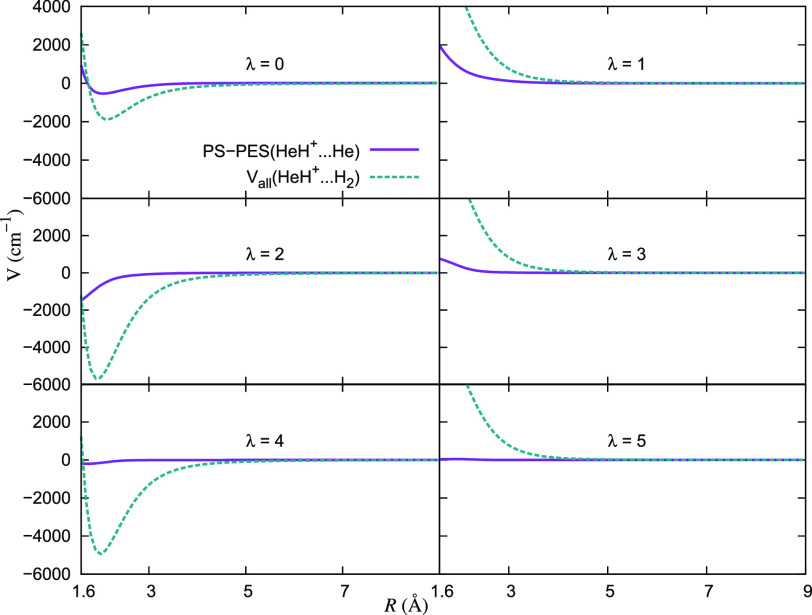
Comparison of the lowest six multipolar expansion coefficients generated for the 2D RR-PES for the HeH^+^···H_2_ system, discussed in this work, and that for the HeH^+^···He system, taken from ref (28).

It is interesting to further note about the radial coefficients reported in Figure S3 additional data that the ones associated with λ = 2 and 4 values correspond to the most attractive anisotropy terms of the expansion and will therefore play an important role for the direct dynamical couplings we shall analyze in the ensuing sections. This was also the case for the multipolar coefficients discussed earlier within the 4D expansion of the RR-PES. The two most abundant partners in interstellar conditions, after the H atoms, are the He atom and the H_2_ molecule. It is therefore of interest to compare the differences in the anisotropy which exist between these two partners and the HeH^+^ cation. The case of the He partner has been extensively discussed in our earlier work.^28^ In the Figure 14, we present a comparison of the lowest six multipolar radial coefficients for these two systems interacting with the cation.

Those data clearly show that the lowest three terms of the expansion with an even index for HeH^+^···H_2_ are all more strongly attractive than the same terms for the HeH^+^···He system from ref (28), thus suggesting that we should expect a more efficient dynamical coupling of the cation rotational states during collisions with H_2_ than with He. With the same token, we also see that the three lowest coefficients with an odd index present more marked repulsive potential branches at short-range interactions for HeH^+^···H_2_, another factor that can enhance the efficiency of rotational inelastic processes. Such features will help us to better understand the behavior of the computed inelastic cross sections and rates which we will be discussing in the next section.

We briefly report below the computational results for purely rotational inelastic scattering of HeH^+^ with para-H_2_ (*j* = 0) using the 2-dimensional RR-PES discussed herein. The standard time-independent formulation of the Coupled-Channel (CC) approach for diatom–atom collisions has already been known for many years (see, for example, Taylor^51^ for a general text-book formulation) and the more recent literature on the actual computational methods has also been very extensive. Additionally, since we have already discussed our specific computational methodology in many of our earlier publications,^52−54^ we simply follow the standard procedure already outlined in the previous section, while we have used here our in-house computational code ASPIN.^52,53^

The number of rotational states coupled within the dynamics was up to *j* = 19 and the expansion over the *J* values to converge the individual cross sections went up to *J* = 100 at the highest energies. The radial range of integration during the propagation of the coupled eq.s covered radial values from 1.0 to 1000.0 Å using a variable number of points which went up to 5000. The range of *E*_trans_ went from 10^–4^ cm^–1^ to 10^4^ cm^–1^ with 1500–2000 points for each considered transition. The reduced masses used were 2.225085 amu for the HeH^+^...He and 1.437398 amu for the HeH^+^–H_2_ system. The multipolar coefficients included to ensure full convergence of the inelastic cross sections went up to a maximum of λ = 25 for the largest values of the Δ*j*_1_ transitions considered important (up to +3), while for most transitions fewer values of λ turned out to be sufficient.

**Figure 15:**
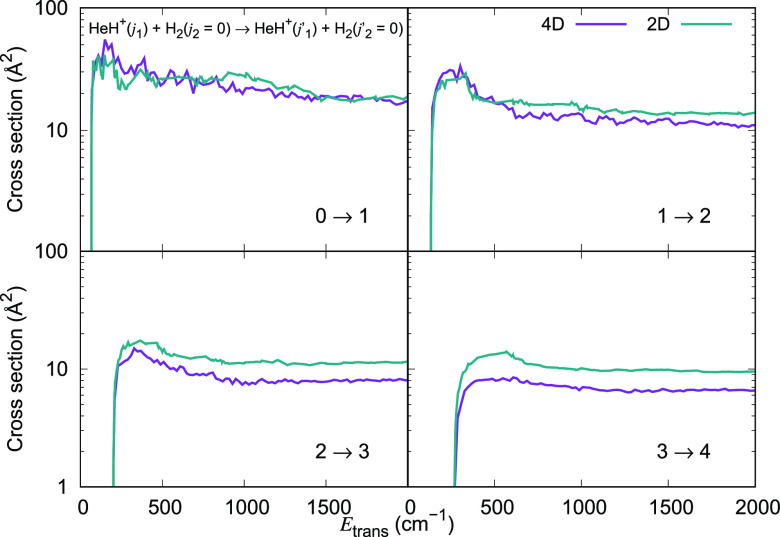
Computed excitation cross sections for a series of inelastic processes with Δ*j*_1_ = +1, generated using the 2D RR-PES for the HeH^+^···H_2_ system, compared with the corresponding results obtained using the 4D RR-PES for HeH^+^(*j*_1_)···H_2_(*j*_2_ = 0) collisions.

**Figure 16:**
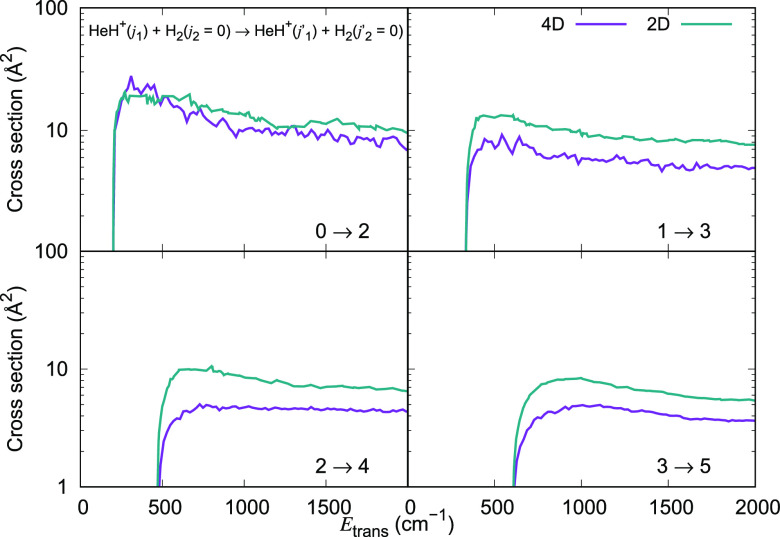
Computed excitation cross sections for a series of inelastic processes with Δ*j*_1_ = +2, generated using the 2D RR-PES for the HeH^+^···H_2_ system, compared with the corresponding results obtained using the 4D RR-PES for HeH^+^(*j*_1_)···H_2_(*j*_2_ = 0) collisions.

Examples of the several inelastic cross sections which we have calculated for the present title system will be reported below in comparison with the previous results obtained using the 4D-PES. Hence, a comparison of the results obtained using the 2D-RR-PES and the 4D-RR-PES in Figure 15 for several Δ*j*_1_ = +1 transitions. It is clear that the reduced dimensional results are remarkably similar to those obtained using the full 4D-RR-PES. This is particularly evident when excitation processes that start from the lowest rotational states are considered (e.g., see upper two panels of Figure 15). When higher excited states are considered, however, the 2D dynamics tends to overestimate the cross sections when compared to the 4D dynamics. Similar comparisons hold for Δ*j*_1_ = +2 transitions as illustrated in Figure 16. In the upper panels of that figure, we see once more that to go from a 4D full inelastic dynamics to the reduced 2D treatment has little effect on the size of the excitation cross sections, while for excitation processes starting from more excited levels the reduced-dimensionality dynamics yields cross sections that are consistently larger by an average 15% .

**Figure 17:**
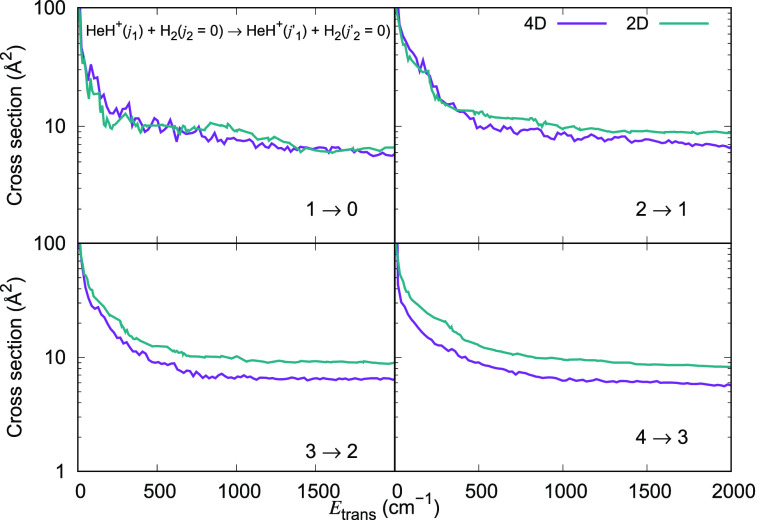
Computed de-excitation cross sections for a series of inelastic processes with Δ*j*_1_ = −1, generated using the 2D RR-PES for the HeH^+^···H_2_ system, compared with the corresponding results obtained using the 4D RR-PES for HeH^+^(*j*_1_)···H_2_(*j*_2_ = 0) collisions.

**Figure 18:**
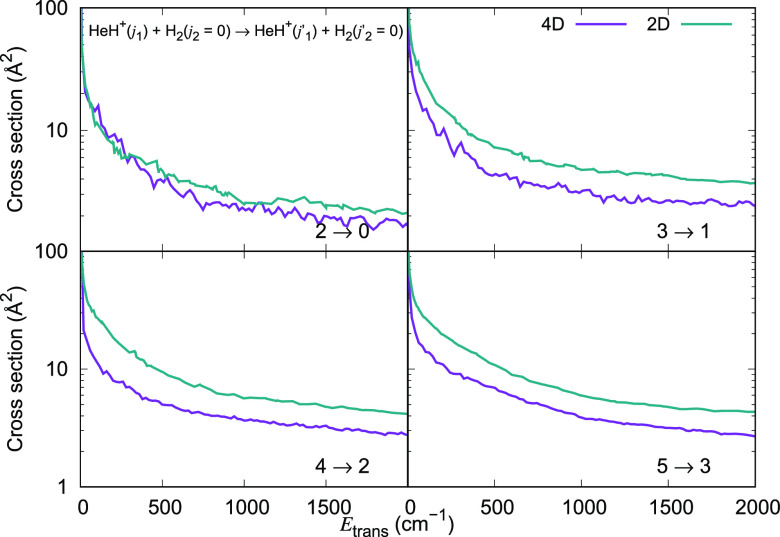
Computed de-excitation cross sections for a series of inelastic processes with Δ*j*_1_ = −2, generated using the 2D RR-PES for the HeH^+^···H_2_ system, compared with the corresponding results obtained using the 4D RR-PES for HeH^+^(*j*_1_)···H_2_(*j*_2_ = 0) collisions.

If we now look at the de-excitation processes obtained using the 2D-RR-PES, as illustrated in Figures 17 and 18 for Δ*j*_1_ = −1 and Δ*j*_1_ = −2 transitions, respectively, we see again the same general trend: all de-excitation cross sections which start from the lowest rotational states of the cations are essentially unchanged when going from the correct 4D dynamics to the simplified 2D dynamics. However, when the initial rotational states of the cations are excited states, we find that the 2D treatment increases the size of the cross sections around 10% to 15% .

### 3.3 Rotationally Inelastic Rate Coefficients

Once the state-to-state inelastic cross sections () were computed, the rotationally inelastic rate coefficients  were evaluated as the convolution of the cross section values over a Boltzmann distribution of the *E*_trans_ values at the selected temperature *T*:
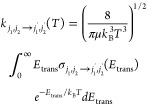
11

The individual rate coefficients were obtained at intervals of 1 K, starting from 5 K and going up to 500 K.

**Figure 19:**
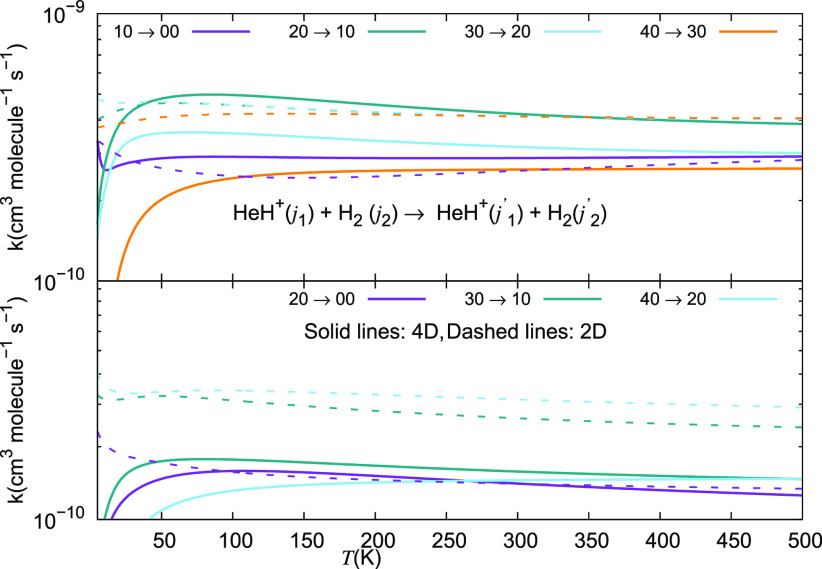
Computed de-excitation rate coefficients for a series of inelastic processes for the HeH^+^(*j*_1_)···para-H_2_(*j*_2_ = 0) system, discussed in this work. The upper panel reports rate coefficients with Δ*j*_1_ = −1, while the lower panel indicates processes with Δ*j*_1_ = −2.

**Figure 20:**
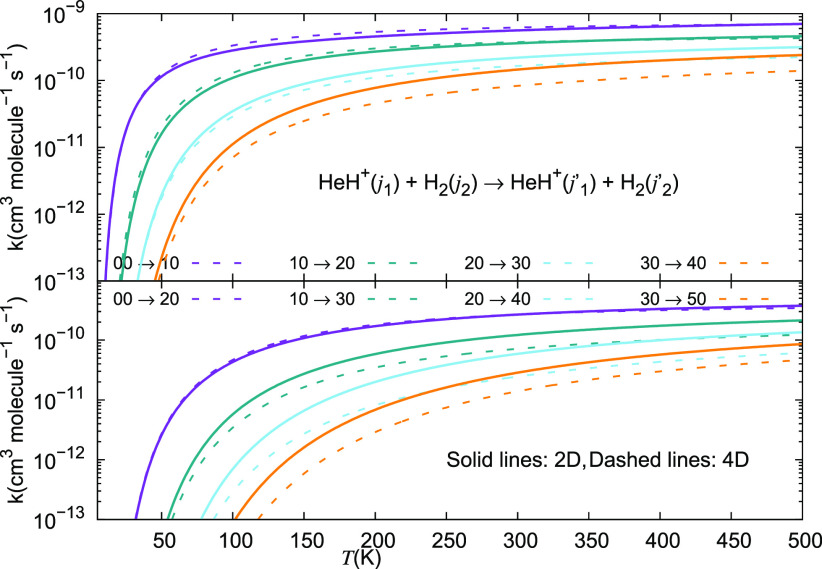
Computed rotational excitation rate coefficients for a series of inelastic processes for the HeH^+^(*j*_1_)···para-H_2_(*j*_2_ = 0) system, discussed in this work. The upper panel reports rate coefficients with Δ*j*_1_ = +1, while the lower panel indicates processes with Δ*j*_1_ = +2.

It is therefore interesting at this point to look at the relative behavior of the de-excitation rate coefficients obtained from the energy-release cross sections of Figures 17 and 18. The calculated rate coefficients for some of the de-excitation processes are shown in the two panels of Figure 19 where the range of temperatures is extended up to 500 K, as discussed earlier. The excitation processes are presented in the two panels of the next Figure 20. The data in the first of the figures show clearly how relatively smaller rate coefficients are obtained for the energy-release transitions associated with the smallest energy gaps, where the 1 → 0 and the 2 → 0 transitions take place. Since the larger quantities of energy which are being released for the other de-excitation processes make the latter transitions to be more impulsive, we see that they correspond to larger rates being generated for them when the collision dynamics becomes more sudden after collision and the interaction times between departing partners are reduced accordingly. Furthermore, the data in both Figures 19 and 20 clearly show that the temperature dependence and relative magnitudes of the larger rates obtained either with the full 4D dynamics (solid lines) or using the reduced 2D dynamics (dashed lines) are largely coinciding with each other. In fact, only when we look at state-changing processes involving excited rotational states of our cation we see some difference in size only: a factor of about 20%, with the exact calculations coming out to be the smaller, as already noticed about the calculated cross sections discussed in the preceding section.

The results we have shown thus far indicate that state-changing collisional dynamics of the title cation with the para-H_2_(*j*_2_ = 0) neutral partner is definitely an efficient process generating fairly large state-to-state cross sections. It is therefore a significant set of data for assessing its relative importance within the network of dynamical processes in the early universe conditions. The latter would involve also other, relatively abundant partners like He and H atoms, hence in the following section, we shall compare the present findings with the earlier results found for the latter species and already discussed by us in our earlier work of ref (28), regarding the He partner of the title cation.

### 3.4 Comparing He, H, and *p*-H_2_(*j*_2_ = 0) State-Changing Collision Efficiencies

**Figure 21:**
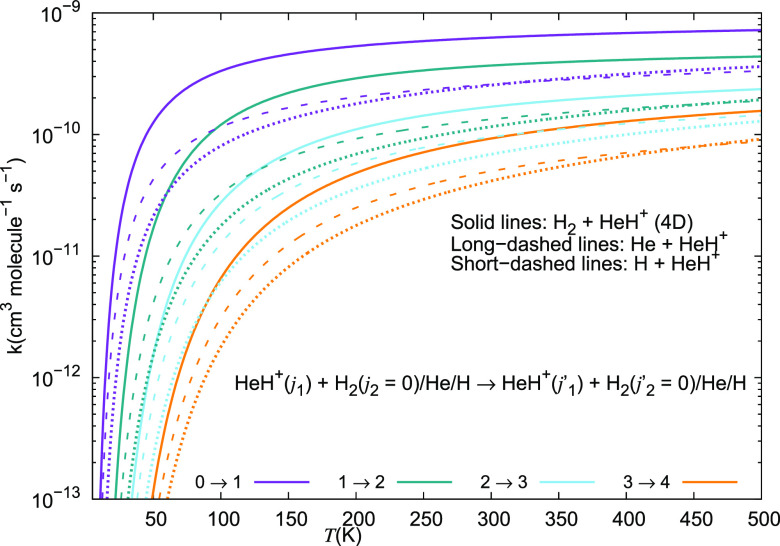
Computed excitation rate coefficients for a series of inelastic processes for the HeH^+^···para-H_2_(*j*_2_ = 0) system, discussed in this work for excitation transitions with Δ*j*_1_ = +1. The present results are given by solid lines, while the long-dashed curves refer to the data for HeH^+^···He taken from Gianturco et al.^28^ The data for the HeH^+^···H system are taken from Desrousseaux and Lique^14^ and are given by the short-dashed lines.

To assess the relative importance of state-changing processes induced by the para-H_2_(*j*_2_ = 0) collisions with the HeH^+^ cation, we compare the present dynamical outcomes with the rate coefficients for rotational energy transfer in HeH^+^ by collision with neutral H, another important component in different interstellar media, and with He atoms. We have taken the reported rate coefficients for the H partner from the earlier calculations by Desrousseaux and Lique,^14^ while for the same range of state-changing, rotationally inelastic collisions involving He as a neutral partner, we use the results from our earlier work on that system.^28^ The data showing the comparisons of these different calculations are reported in Figure 21, which we shall discuss in detail in the following paragraphs.

**Figure 22:**
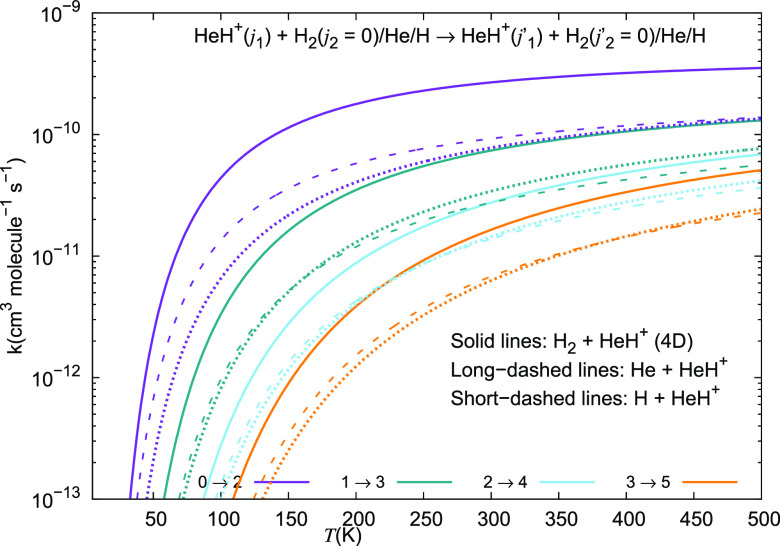
Computed excitation rate coefficients for a series of inelastic processes for the HeH^+^···para-H_2_(*j*_2_ = 0) system, discussed in this work for excitation processes with Δ*j*_1_ = +2. The present results are given by solid lines, while the long-dashed curves refer to the data for HeH^+^···He taken from Gianturco et al.^28^ The data for the HeH^+^···H system are taken from Desrousseaux and Lique^14^ and are given by the short-dashed lines.

We report in Figures 21 and 22 the different inelastic rate coefficients involving HeH^+^ as an ionic collision partner for the three different neutral partners mentioned earlier, which are all considered to be among the most abundant species present in the recombination era from early universe models: H, He, and H_2_. Figure 21 shows excitation processes involving the Δ*j*_1_ = +1 transitions from the lowest four rotational states of the ion, while Figure 22 presents the excitation processes with the Δ*j*_1_ = +2 transition. The following comments can be made:

(i) all the excitation inelastic rates involving the para-H_2_(*j*_2_ = 0) neutral partner are always larger than those obtained either with H or with He as the alternative collision partners;
(ii) especially in the lower range of temperatures below 200 K the inelastic rates with molecular hydrogen are nearly 1 order of magnitude larger than those obtained for He, and even larger than that for the case of the excitation rate coefficients due to the H atom;
(iii) over the entire range of temperatures considered in this study, we therefore see that molecular hydrogen remains by far the most efficient collision partner in causing internal excitations between the rotational states of the present cation.

**Figure 23:**
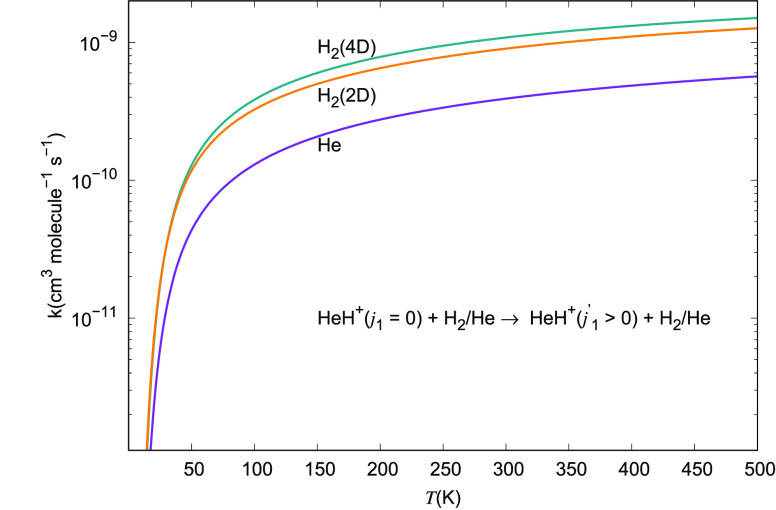
Comparison of global excitation rate coefficients for inelastic processes from the lowest rotational state of the cation in the HeH^+^(*j*_1_)···para-H_2_(*j*_2_ = 0) and the HeH^+^(*j*_1_)···He systems. The excitation considered goes up to *j*_1_ = 9 for both neutral partners. The rotational state of the H_2_ partner is kept fixed and equal to 0.

The results we are presenting in Figure 23 provide a sort of global indicator on the excitation efficiency of the rotational state-changing rate coefficients of the present cation in collision with either He or H_2_ as its neutral partners. The examined range of temperatures is the same as that given in Figures 19 and 20. The dominance of the excitation probability by molecular hydrogen over that with He atoms is clearly visible in Figures 21 and 22, thereby confirming that one should expect the H_2_ partner to be the most efficient collisional partner for HeH^+^. We further see in that figure that the excitation efficiency obtained via either the full 4D quantum dynamics treatment or via the reduced 2D simpler treatment are very much of the same magnitude and show the same temperature dependence. In other words, the interaction and dynamics involving the nonrotating para-H_2_ partner are very realistically described by using the simpler, averaged interaction leading to the usual 2D quantum dynamics with an atom-like partner in collision with the title cation.

**Figure 24:**
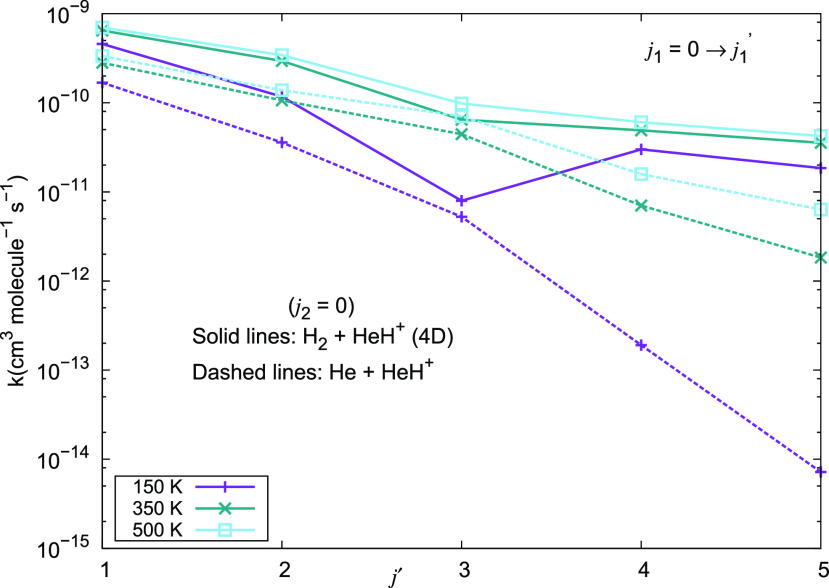
Computed excitation rate coefficients for a series of inelastic processes for excitations into different *j*_1_^′^ states of the cation. They have been generated using the 2D and 4D RR-PES for the HeH^+^···*p*-H_2_(*j*_2_ = 0) system, discussed in this work.

Additional data for the present comparisons are reported by the calculations shown in Figure 24, where the state-to-state excitation rate coefficients are given at three different temperatures and for excitations from the *j*_1_ = 0 initial state to excited states up to *j*_1_ = 5. The exact 4D results for para-H_2_(*j*_2_ = 0) are given by solid lines, while those for the neutral He partner are given by the dashed lines. Once more the data presented indicate that the excitation rate coefficients obtained for the molecular hydrogen which can occur with the HeH^+^ cation under the selected ISM conditions are uniformly larger than those expected for neutral He. We also see that excitation to the higher rotational states shows the largest differences between H_2_ and He as partners.

A fairly common procedure for comparing collisional outcomes involving He and H_2_ when modeling dynamics under ISM conditions is to use the rate coefficients for helium collisions, when available, to approximate those for H_2_ by essentially using the latter data within some scaling prescription. The rate coefficients for the hydrogen partner are then obtained simply as those for the He case scaled by the ratio of their reduced masses, thereby writing
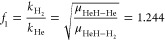
12

One could also use a slightly more sophisticated scale factor that additionally accounts for the differences between the two dominant long-range interactions with the same ionic partner, as shown below:
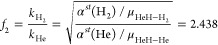
13where α^*st*^(He) = 1.384*a*_0_^3^ and α^*st*^(H_2_) = 5.314*a*_0_^3^ are the dipole polarizability of He and H_2_, respectively.

The validity of such scalings has been extensively discussed in the literature. It was recently tested with calculations involving the CO neutral target with different collision partners^55^ and found them to be unreliable. The same conclusions were also reached by our earlier calculations for the CN^–^ anion in collisions with the same two partners.^56^ Some authors^55^ also suggested different scaling procedures which were found to fare slightly better but which further underline the need to have the actual rate coefficients separately computed for the two systems.

**Figure 25:**
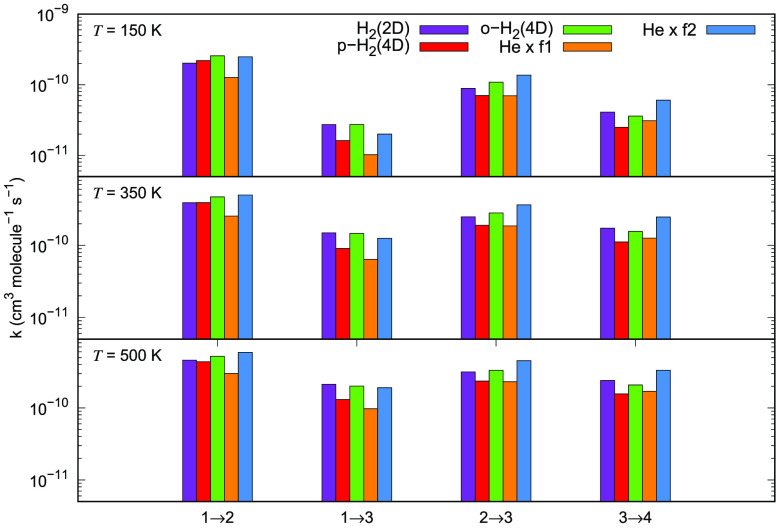
Comparison of the excitation rate coefficients for a series of inelastic processes of the HeH^+^···*p*-H_2_(*j*_2_ = 0), *o*-H_2_ systems obtained from both 2D (magenta) and 4D (green and blue) calculations. The values of the same quantities, obtained by two different scaling procedures discussed in the main text, are shown for the HeH^+^···He (by dark and light yellow colors). Four different excitation processes are compared at three different temperature values.

Here we compare the rate coefficients for HeH^+^···He^28^ with those calculated here for HeH^+^ with *p*-H_2_(*j*_2_ = 0) and *o*-H_2_(*j*_2_ = 1). We employ either of the above scale factors to extend our comparisons. Results are given in Figure 25 which shows in its panels the same unmodified set of excitation rate coefficients, obtained here for H_2_ as a partner of the present cation, with the scaled quantities obtained from the HeH^+^···He system. The values computed for the H_2_ partner are given by the bars colored in purple for the 2D dynamics, in green for the 4D calculations for *p*-H_2_ and in blue for the case of *o*-H_2_. The scaled values obtained from the HeH^+^···He calculations are given by the dark yellow color code when using the *f*_1_ prescription, and by the light yellow for the *f*_2_ scaling choice. The data are for three different temperatures: from 150 K up to 500 K, and four different excitation rate coefficients. The following can be gleaned from the panels reported in that figure:

(i) on the whole, neither of the scaling factors turns out to accurately reproduce results for the molecular hydrogen partner, although the use of the more sophisticated *f*_2_ scaling gives marginally better accord with the data for H_2_;
(ii) the mismatch between scaled rate coefficients and those for the para-H_2_ partner is largely independent of the temperature, while closer to the correct data only for the transitions between the two lowest rotational states, getting worse for the transitions between excited levels of the cation;
(iii) the use of the *f*_1_ scaling factor produces invariably the worse rate coefficient estimates for the para-H_2_ partner, markedly smaller than the values from actual calculations; on the whole, therefore, it is fair to conclude once more that scaling procedures do not succeed, even for the present system, in producing realistic estimates for collisional rate coefficients involving hydrogen molecules as partners of the title cation.

The assumption of a local thermodynamic equilibrium (LTE) in different regions of the interstellar medium in general, is expected to hold whenever the population of the excited levels under consideration is likely to be given by the Boltzman’s law. This might happen whenever the rates of spontaneous emission from the internal levels of the polar molecule (in our present case, the rotational levels of HeH^+^) are smaller than the rates of de-excitation by collision with the most abundant partners present in that ISM region. This implies that the density in the interstellar gas for the partners should be significantly larger than some critical value so that the LTE assumption can be kept. The definition of a critical density (e.g., see: Gianturco et al.,^28^ Lara-Moreno et al.^57^) is given as
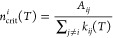
14where the critical density for any *i*^th^ rotational level is therefore obtained by giving equal weights to the consequences of either the collision-induced or the spontaneous emission processes. We have taken the rate coefficients discussed in Section 3.3, including here rotational levels up to *j* = 5. We have also employed the computed spontaneous decay Einstein coefficients discussed and presented in a very accurate set of calculations recently proposed in Reference (58) for a series of early universe molecular systems and their isotopologues.

**Figure 26:**
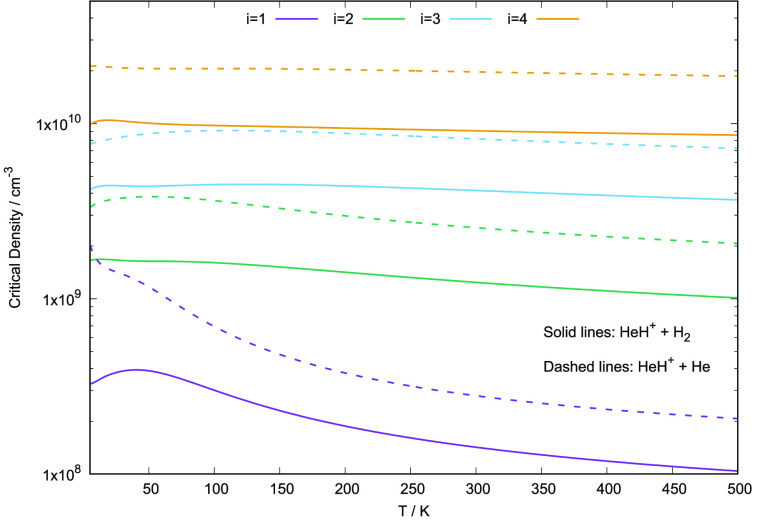
Computed critical densities for the HeH^+^/para-H_2_(*j*_2_ = 0) system, as defined in eq 14, for temperatures from 5 to 500 K. Present results (solid lines) are compared with the earlier calculations from Gianturco et al.^28^ (dashed lines).

The results in Figure 26 were obtained using the collisional rate coefficients calculated here for the hydrogen molecule within the 2D dynamics (solid lines) and those calculated earlier by us for the He partner^28^ (dashed lines). The fairly large values obtained for the critical densities are mainly controlled by the very large spontaneous radiative emission coefficients (taken from Amaral et al.^58^) that appear in the numerator of eq 14. The significant role of the H_2_ partner is again confirmed by the fact that critical density values are found to be larger for the He partner than for the H_2_ case.

Possible values of the baryon densities, *n*_b_, in the early universe environments,^6^ indicate *n*_b_ proportional to the red shift *z* via the relationship: (1 + *z*)^3^ (see: Galli and Palla^6^). Hence, for values of *z* varying between 200 and 5000 the corresponding *n*_b_ values are from about 10^–1^ cm^–3^ up to about 10^3^ cm^–3^. From Figure 26, we see that the critical densities associated with either of the possible collision partners for HeH^+^ are markedly larger than the above estimates. This means that we expect the rotational state population of the present cation not to be under LTE conditions since the critical density values are all large enough to allow the molecules to radiate well before they can collisionally de-excite. Under such conditions, therefore, to accurately know the collision-driven rates would be important since the LTE approximation cannot be employed in kinetic networks as it would not provide reliable estimates of the relative populations.The knowledge of the actual collisional rates with abundant partners like He and H_2_ is then relevant for more realistic modelings of the energy flow processes than simply using LTE conditions.

### 3.5 *o*-H_2_ and *p*-H_2_ Dynamics with HeH^+^

The value of the *o*-H_2_/*p*-H_2_ abundance ratio in interstellar clouds is a relevant quantity when discussing collisional excitation processes in the ISM, and it has been the object of study in models involving dense interstellar clouds (for an extensive anaysis see: Flower and Watt,^59^ Lique et al.^60^ and references quoted therein) where the temporal evolution of that ratio and its temperature dependence are influential for setting up realistic chemical models.

Due to the possible occurrence of proton-exchange interconversion processes like the following:
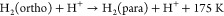
15where thermal equilibrium is considered to be attained over a time-scale τ which is controlled by the proton density in the clouds. In situations where the molecular H_2_ density is estimated to be between 10^2^ and 10^3^ cm^–3^, and when photoprocesses are also significant, the proton densities remain fairly large and thus the τ values are around 10^6^ yr.^59,60^ However, in denser clouds where the molecular hydrogen densities are about 2 orders of magnitude larger, then the proton density is being reduced by charge-exchange reactions with neutrals and τ values are of the order of about 10^7^ yr, which is comparable with the estimated cloud lifetime. Hence, the value of the ratio depends on the chemical history of the clouds.^59^

From the structural standpoint, the spacings of the rotational levels is significantly larger in *o*-H_2_ in comparison with *p*-H_2_: *E*_3_ – *E*_1_ = 875 K while *E*_2_ – *E*_0_ = 525 K. Furthermore, the quadrupole moment contributes to the long-range interaction when *o*-H_2_ is involved, but not with *p*-H_2_. Although we did not find it to be significant to add quadrupolar interactions for the two types of H_2_ partners in the quantum dynamics at the temperatures of interest here, it is reasonable to expect that collision dynamics will change depending on which of the two species of hydrogen molecules is considered in the calculations: such changes will be analyzed below in the following discussion.

The normal hydrogen is known to be a mixture of ortho- and para- hydrogen. At low temperatures, ortho-hydrogen is essentially in *j*_2_ = 1 state and para-hydrogen is in *j*_2_ = 0 state, while the normal (*n*) hydrogen is a mixture of the two. In the limit *T* → 0, ortho and para forms exist in the ratio 3:1. However, at higher temperatures (assuming for now LTE conditions), the ratio would be .^59^ Since we have used *B*_e_ = 60.8 cm^–1^, the energy gap between *j*_2_ = 0 and 1 states = 2*B*_e_ = 121.6 cm^–1^ = 175 K, as also indicated in eq 15.

Therefore, the rate coefficient (*k*_*n*_) for *j*_1_ = 1 → 0 transition in the presence of normal hydrogen is computed as follows:
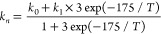
16where *k*_1_ refers to *k* for *j*_2_ = 1 and *k*_0_ refers to *k* for *j*_2_ = 0. In the absence of equilibrium between ortho- and para- forms of hydrogen, *k*_*n*_ = (*k*_0_ + *k*_1_ × 3)/4.

**Figure 27:**
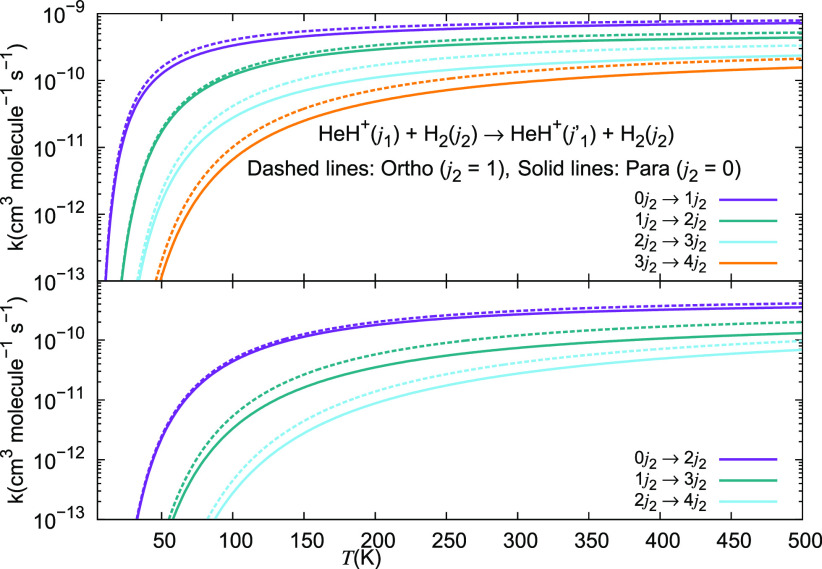
Computed rotational excitation rate coefficients for collisional state-changes of HeH^+^ interacting with either *p*-H_2_ or *o*-H_2_. The upper panel reports Δ*j*_1_ = +1 transitions while the lower panel presents Δ*j*_1_ = +2 transitions.

The data in the two panels of Figure 27 show the computed rotational excitation rates for the Δ*j*_1_ = +1 and Δ*j*_1_ = +2 excitations of the cation partner in collision with either *o*-H_2_ (dashes) or with *p*-H_2_ (solid lines). They clearly indicate that the rates involving the former molecular hydrogen species are consistently larger than those involving the latter variant of that molecule.

**Figure 28:**
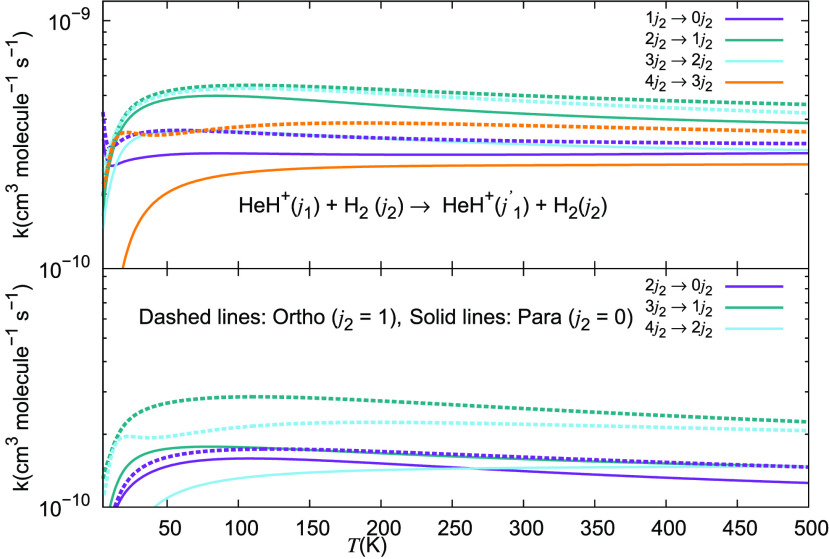
Same type of comparisons as those reported by the previous Figure 27 by this time involving de-excitation inelastic processes. Upper panel: Δ*j*_1_ = −1 transitions. Lower panel: Δ*j*_1_ = −2 transitions.

If we now turn to de-excitation processes, those presented by the two panels of Figure 28, we see that the same trend is confirmed and the *o*-H_2_ molecular partner is a more efficient collision partner in deactivating populations of the HeH^+^ rotational states than the *p*-H_2_. As a matter of fact, the de-excitation probabilities with Δ*j* = −2 show an even more marked difference in size between the two H_2_ variants as partners of the title cation.

**Figure 29:**
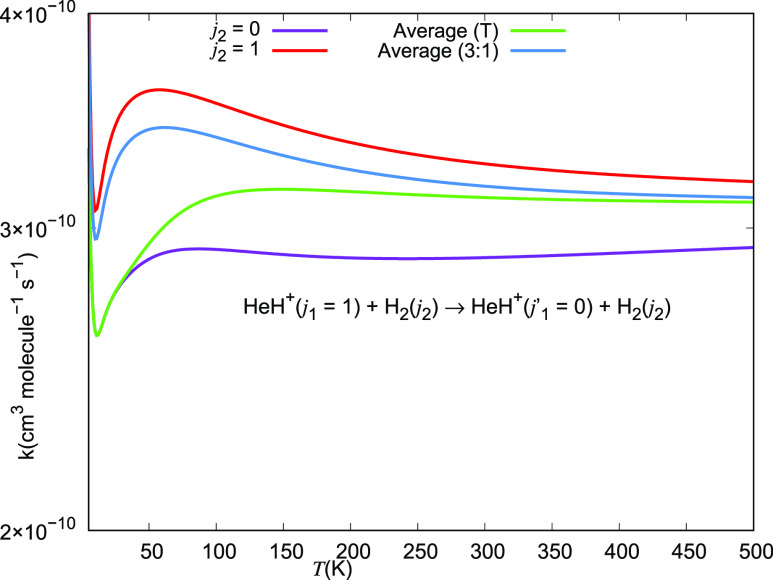
Comparing collisional de-excitation rate coefficients, involving the lowest two rotational states of the HeH^+^ cation, in collision with either *o*-H_2_ or *p*-H_2_ partners. The different curves have been obtained using the definitions discussed in the text.

Another way of analyzing the dynamical differences between *o*-H_2_ and *p*-H_2_ as collision partners of HeH^+^, is presented by the data reported by the different curves of Figure 29. We are considering the collisional cooling process between the *j*_1_ = 1 and *j*_1_ = 0 levels of the cation with either the ortho- or para-variants of the H_2_ molecular partner. We clearly see there that former molecular species yields the larger cooling rate coefficient(red curve) with respect to the latter variant, given by the magenta solid line. We also see that if we consider the fixed ratio of the two species in n-hydrogen to be given by the value of 3:1, we get larger rate values in the low-temperature regimes with respect to conditions which are at 200 K or above (see blue curve). However, when that ratio is taken to also be temperature dependent within LTE conditions (green curve), we see a rapid increase at the lowest *T* values but a much slower *T*-dependence at the higher temperatures above about 100 K. Since such conditions are related to the chemical history of the interstellar clouds, then we see that marked variations in collisional efficiency appear under different cloud conditions depending on the relative abundances of the two molecular hydrogen variant.

**Figure 30:**
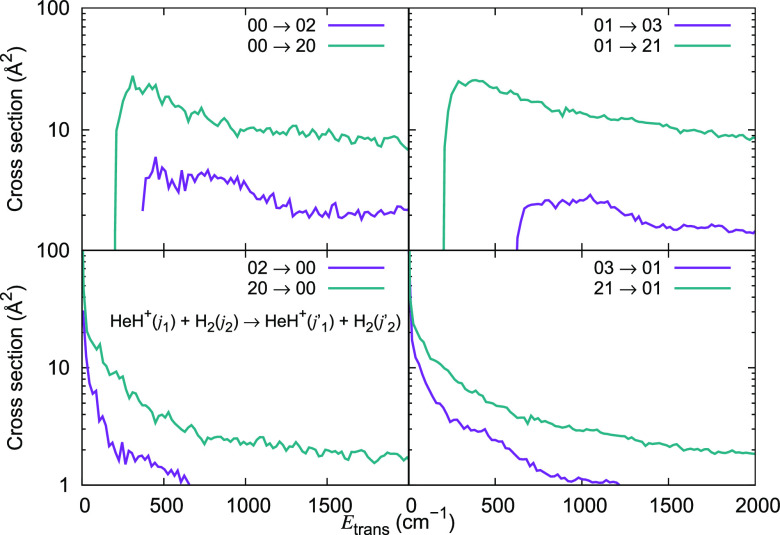
Computed cross section values for (Δ*j*_2_ = +2) and (Δ*j*_2_ = −2) processes in *o*- and *p*-H_2_ activated by collision with HeH^+^(*j*_1_ = 0). Included for comparison in the green-colored curves are the corresponding values for excitation (Δ*j*_1_ = 2) and de-excitation (Δ*j*_1_ = −2) processes in HeH^+^ due to collision with H_2_(*j*_2_ = 0, 1).

In order to extend the analysis of the 4D dynamics of the present study, we have also examined the efficiency of the cationic HeH^+^ on activating rotational excitation and de-excitation processes involving the neutral partners *o*- and *p*-H_2_. Because of the homonuclear symmetry of H_2_, only an even change in its rotational state is allowed, hence only Δ*j*_2_ = 2 will occur. As specific examples, we report the cross sections for Δ*j*_2_ = ± 2 transitions in H_2_(*j*_2_ = 0, 1) in collision with HeH^+^(*j*_1_ = 0) in Figure 30.

Comparisons with the cross section results for similar transitions, but involving HeH^+^ as target, are given in the same Figure 30. They clearly reveal that the probabilities for state-changing processes for H_2_ are an order of magnitude smaller than those for HeH^+^. Since we know that the threshold for excitations is larger (10*B*_*e*_ = 608 cm^–1^) for *o*-H_2_ than for the *p*-H_2_ (6*Be* = 364.8 cm^–1^) case, we also see this difference reflected in the size differences of the two types of cross sections.

**Figure 31:**
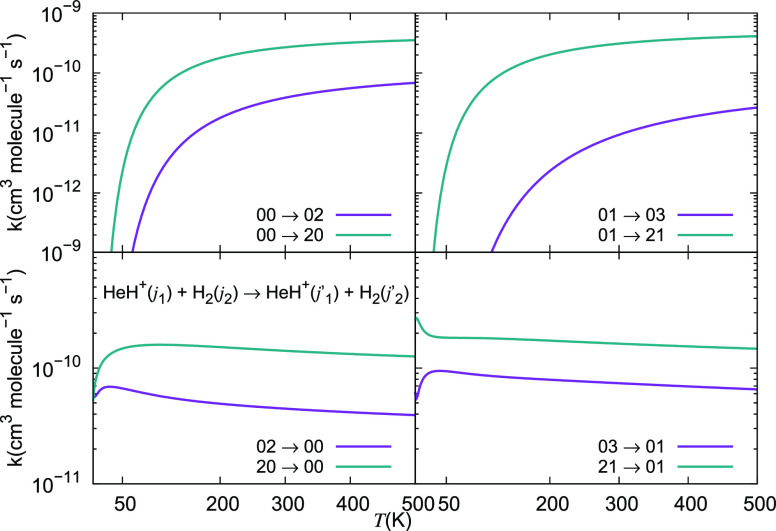
Values for the computed inelastic rate coefficients for excitation (Δ*j*_2_ = +2) and de-excitation (Δ*j*_2_ = −2) processes in *o*- and *p*-H_2_ in collision with HeH^+^(*j*_1_ = 0). Included via the green-colored curves are the corresponding inelastic rate coefficients for excitation (Δ*j*_1_ = 2) and de-excitation (Δ*j*_1_ = −2) processes involving HeH^+^ by collision with H_2_ (*j*_2_ = 0, 1).

To better understand the effects of such differences on the relative behavior of the inelastic rate coefficients, the latter quantities for the corresponding excitation and de-excitation processes in H_2_ are reported in Figure 31 and are also found to be an order of magnitude smaller than those for the collisional excitation probabilities for ionic HeH^+^ under similar conditions.

## 4 Conclusions

The present work has carried out accurate, ab initio calculations for the quantum dynamics of rotational energy transfer processes of the polar molecular cation HeH^+^ in collision with neutral hydrogen molecules, the latter species estimated to be present in relatively large abundance under the conditions produced by the current modeling of the early universe and of interstellar chemistry in general. We have therefore obtained a new potential energy surface from first-principles, using quantum chemical methods with highly correlated functions as described in Section 2. The molecular target was treated as a rigid rotor and so was the molecular hydrogen partner, thus scaling the PES dimensionality from 6D to 4D. We have successfully used the ML-ANN method to fit the ab initio potential energy values and carried out quantum close coupling calculations to examine rotational excitation and de-excitation processes in HeH^+^ in collision with ortho and para molecular hydrogen.

We have further investigated averaging possibilities to reduce the dimensionality of the RR-PES to a 2D description, which then treats the hydrogen molecule as a nonrotating *p*-H_2_ (*j*_2_ = 0) collisional partner. We have largely focused on the rotational state-changing collisions for the HeH^+^ without involving the less probable H_2_ rotational excitations. In earlier studies by Yang et al.,^61^ in fact, the rate coefficients for rotational state-changing processes for H_2_ were found to be orders of magnitude smaller than those involving the primary target (the CN partner), thereby making our present approximation a reasonable choice. To actually test this fact, we have also presented calculations for rotational excitation of *o*-H_2_ and *p*-H_2_ in collision with HeH^+^ and indeed found their rates to be about an order of magnitude smaller than those involving excitations of the cation for the cases where state-changing processes with Δ*j*_1_ = ± 2 transitions. When one further notices that the primary inelastic processes involving HeH^+^ correspond to Δ*j*_1_ = ±1 transitions, and that the latter rate coefficients are larger than the former by a factor of 3 to 4, we can rest reassured of the marginal role of H_2_ inelastic processes within the present network of inelastic collisions.

It is also interesting to note that studies on the ortho/para ratio for H_2_ in astronomical environments done by Flower and Watt^59^ indicated that the ratio can vary from the “normal” value of 3 to lower ratios down to just about 1 depending on the environmental history of that astronomical region. We have therefore presented specific rate coefficient calculations that quantitatively show differences between the two variants of the molecular hydrogen as a partner of HeH^+^, confirming the importance of realistically modeling the chemical history of the molecular cloud under investigation.

The computed rate coefficients for rotational state-changes of the cation have been also compared with an earlier PES involving the He atom as a collision partner already available in the published literature by Gianturco et al.^28^ We found the molecular hydrogen to be markedly greater in coupling strength and anisotropy features than what had been found for the He interaction. We have calculated a wide variety of inelastic cross sections and then extracted from them the corresponding rate coefficients for the rotational energy-transfer channels: all quantities have been compared in the present study with those obtained earlier from He and H as collision partners, indicating that the largest state-changing efficiency is associated with the hydrogen molecule, as expected from the findings on the features of their respective PESs.

The comparison with different collision partners confirmed that, while He and H are inducing rotational excitation processes in the cation with very similar, and fairly large, efficiency, the *o*-H_2_ consistently turns out to be the more efficient partner in comparison with *p*-H_2_, with both of these species being more efficient than either He or H. These findings therefore suggest that neutral hydrogen molecules are important partners in the chemical networks including inelastic processes for HeH^+^ within the ISM kinetics.

We have further employed Einstein Coefficients for spontaneous decay between rotational levels from an earlier study by Amaral et al.^58^ to evaluate the possible range of densities which can occur, under the early universe conditions, and which can tell us about the competition between collisional and radiative state-changing processes. Given the expected baryonic densities at different redshift values suggested by the current models (see discussion in Galli and Palla^6^), one finds that the critical densities required for the collisional paths to compete with the radiative paths are not likely to be present in the interstellar environments where this molecule has been detected. This indicates that LTE conditions are not to be achieved for the molecular internal temperatures and therefore specific values of collisional rate coefficients have to be used within the kinetic models to get realistic results for estimating the efficiency of energy release from rotational states of the present cation.

In comparison with the reactive channel producing H_3_^+^ and destroying the cation, we have also discussed the experimental evidence which exists thus far, indicating that the known reaction rates around room temperature are largely of the same order of magnitude of the energy-transfer rate coefficients presented here over a much broader range of *T* values. Some experiments down to 200 K^8^ even suggest the reactive coefficients to be much smaller than those found here for the purely inelastic processes. Hence, our results suggest that collisional energy-transfer rates are going to remain of significance even in the presence of the flux into reactive channels.

We have thus provided a broad range of dynamical rate coefficients which allow for a more realistic modeling of the chemical and baryonic evolution kinetics in astrophysical environments and a better knowledge of the efficiency of the collisional cooling paths involving the HeH^+^ cation.

## Supporting Information Available

The multipolar coefficients for the Legendre expansion of the rigid rotor HeH^+^···para-H_2_(*j*_2_ = 0) potential and the computed inelastic rate coefficients from the 2D-RR-PES employed in this work; the ML-NN potential FORTRAN subroutine, the 4D dynamics inelastic cross sections and inelastic rate coefficients (PDF)

## Notes

All the data that support the findings of this study are available within the article itself and in its Supporting Information.
