# Supplementary material for: HeH+ Collisions with H2: Rotationally Inelastic Cross Sections and Rate Coefficients from Quantum Dynamics at Interstellar Temperatures
Source: J Phys Chem A. 2022 Apr 1;126(14):2244–61. doi: 10.1021/acs.jpca.1c10309 (PMC9014418; doi:10.1021/acs.jpca.1c10309)
Supplement: Supplementary file 1 — jp1c10309_si_001.pdf [file jp1c10309_si_001.pdf]

HeH^+ Collisions with H_2: Rotationally Inelastic Cross Sections and Rate Coefficients from Quantum Dynamics at Interstellar Temperatures

K. Giri,[†] L. González-Sánchez,[‡] Rupayan Biswas,[¶] E. Yurtsever,[§] F. A. Gianturco,[*,‖] N. Sathyamurthy,[⊥] U. Lourderaj,[¶] and R. Wester[‖]

†*Department of Computational Sciences, Central University of Punjab, Bathinda, Punjab 151401, India*

‡*Departamento de Química Física, University of Salamanca Plaza de los Caídos sn, 37008, Salamanca, Spain*

¶*School of Chemical Sciences, National Institute of Science Education and Research (NISER) Bhubaneswar, An OCC of Homi Bhabha National Institute, P.O. Jatni, Khurda, Odisha 752050, India*

§*Department of Chemistry, Koc University Rumelifeneriyolu, Sariyer TR 34450, Istanbul, Turkey*

‖*Institut für Ionenphysik und Angewandte Physik, Universität Innsbruck Technikerstr. 25 A-6020, Innsbruck, Austria*

⊥*Indian Institute of Science Education and Research Mohali, SAS Nagar, Manauli, Punjab 140306, India*

E-mail: francesco.gianturco@uibk.ac.at

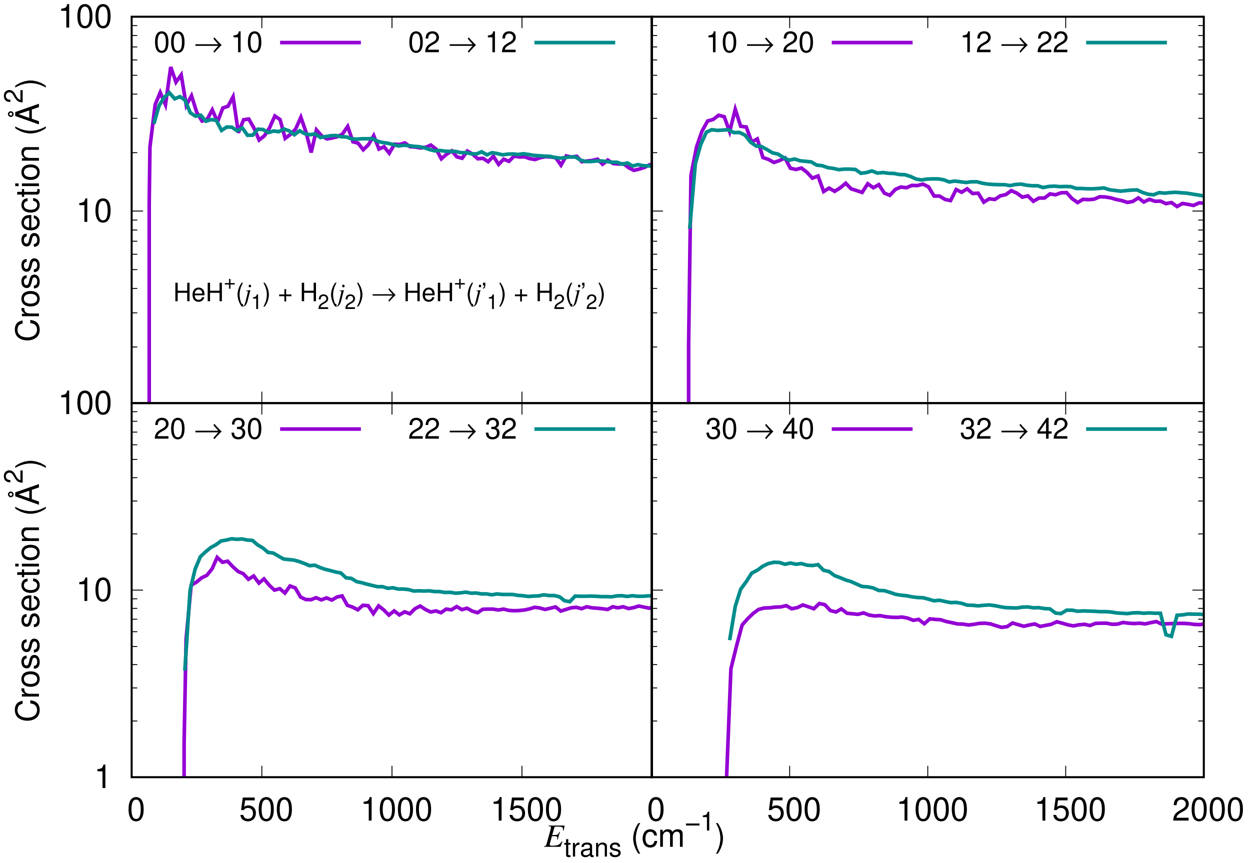

Figure S1: Computed excitation cross sections for a series of inelastic processes with $\Delta j_1 = +1$, generated using the 4D RR-PES for the $HeH^+(j_1)\cdots$p-$H_2(j_2 = 0,2)$ system, discussed in this work.

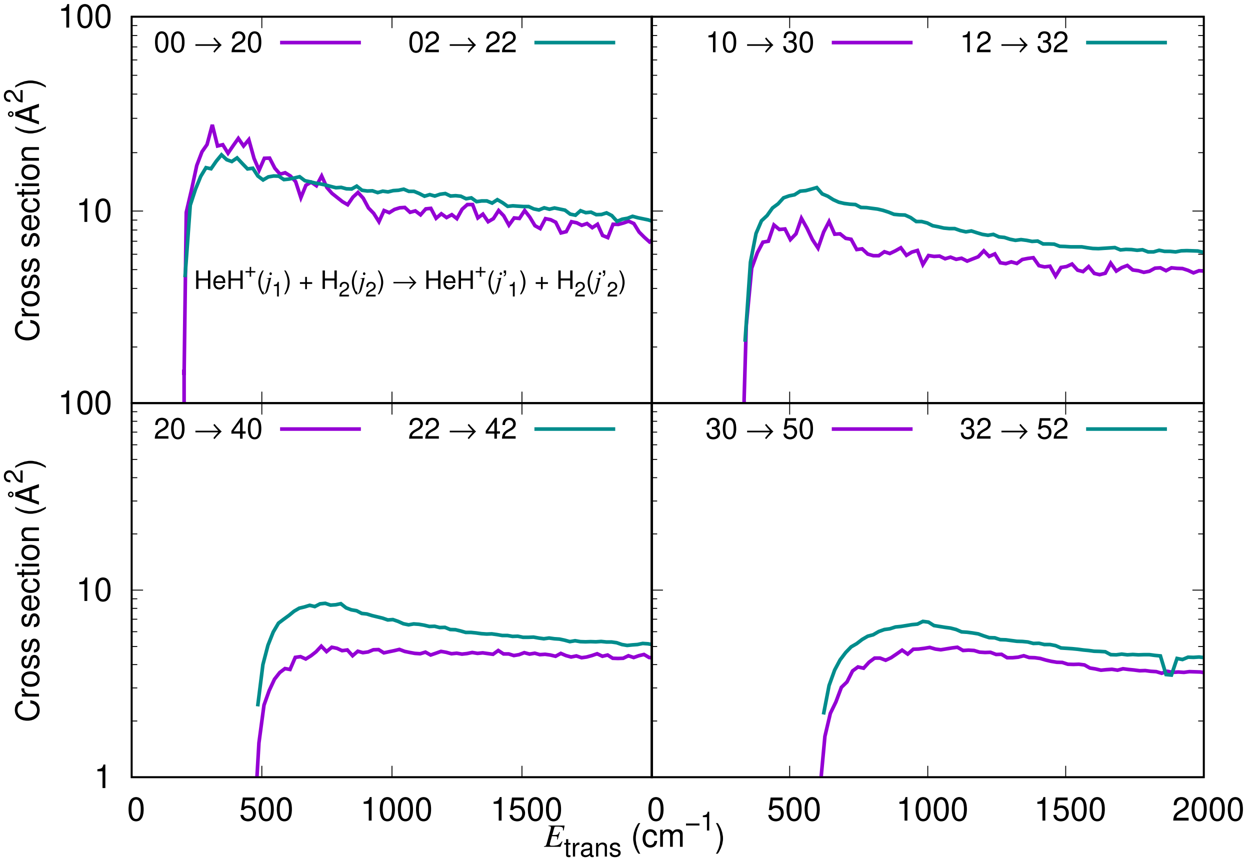

Figure S2: Computed excitation cross sections for a series of inelastic processes with $\Delta j_1 = +2$, generated using the 4D RR-PES for the $HeH^+(j_1)\cdots$p-$H_2(j_2 = 0,2)$ system, discussed in this work.

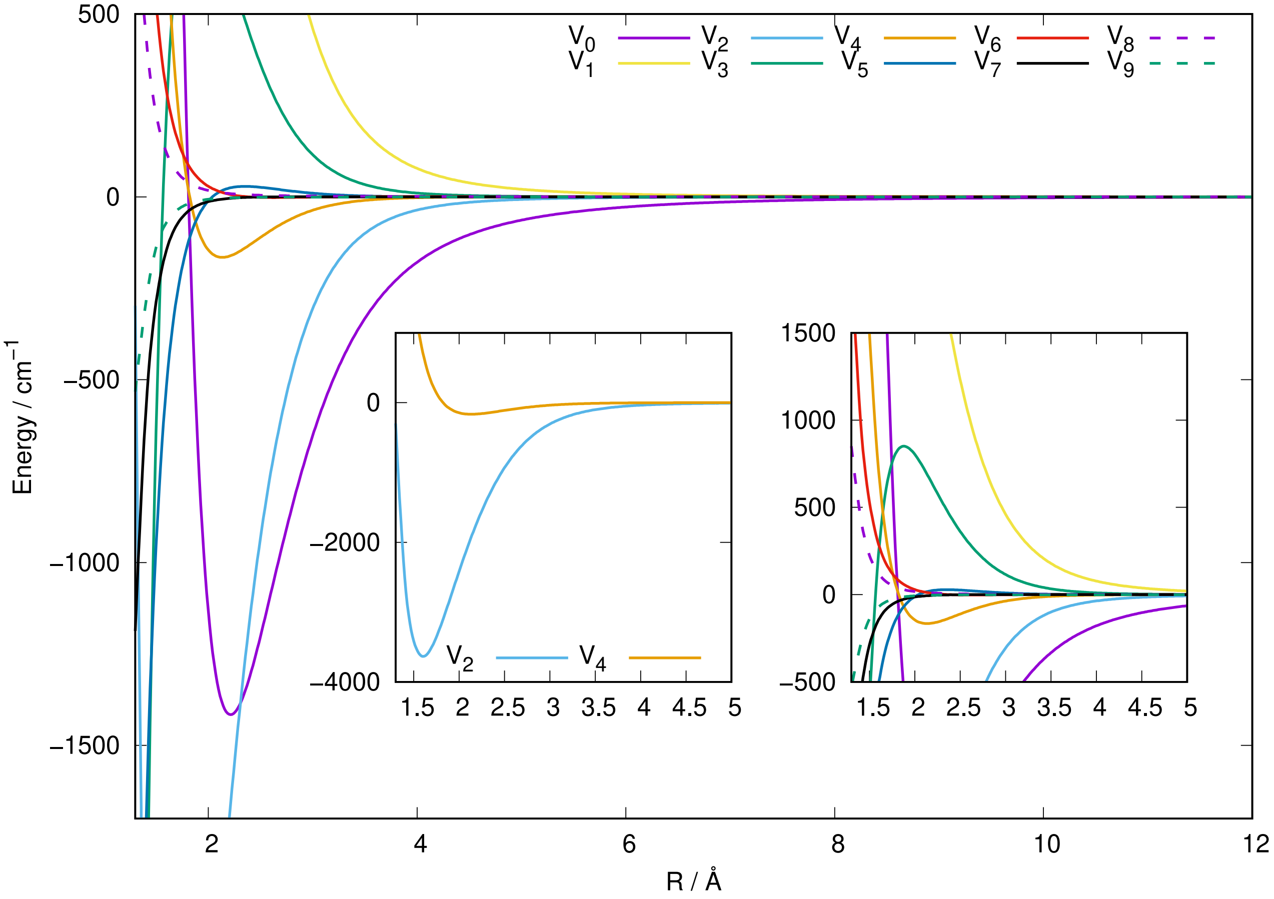

Figure S3: Presentation of the lowest ten multipolar expansion coefficients generated for the 2D RR-PES given by the V_{all} dimensionality reduction scheme. The inserts show the coefficients behaviour around the deepest attractive well and the short-range radial regions.

Subroutine for ANN fit 4D $HeH^+...H_2$ PES

```
SUBROUTINE NEURALPOT(theta,alpha,beta,R,V)
!
!
!     SUBROUTINE FOR 4D HeH+H2 PES, Neural Network FIT
!     FIT TYPE: SHALLOW NEURAL NETWORK WITH 60 NEURONS, TRAINED
!        USING BAYESIAN REGULARIZATION
!     INPUT: theta, alpha, beta, R
!        theta = [0:180]   (degrees)
!        alpha = [0:180]   (degrees)
!        beta = [0:180]     (degrees) 90-180 not covoered by the fit,
!        but rather by the code.
!        R = [1.00:12.00]  (angstrom)
!     OUTPUT: V            (cm-1)
!
!
!
      IMPLICIT NONE
      double precision :: theta,alpha,beta,R
      double precision :: a_idx_1,a_idx_2,a_idx_3,xp1_idx_0,a,V,Vnn,vlr,xx,swi
      integer :: k
      double precision, dimension (60) :: b
      double precision, dimension (60) :: b_a
      double precision, dimension (60) :: c_a
      double precision, dimension (240) :: d_a
      double precision, PARAMETER :: PI=ACOS(-1.D0)
      SAVE
!
     data (b_a(k), k=1,60)/-62.50728204894227d0,-4.945652302732937d0,&
      -10.33575061597528d0,13.11067549024763d0,&
      -13.50700247353357d0,-10.33953935757514d0,&
      -9.209846543331027d0,-21.33409608672802d0,&
      -20.85117062558498d0,-24.85950856856193d0,&
      -37.07548087670428d0,-11.51921643218442d0,&
      -20.69296479699385d0,7.334125023901137d0,&
      35.36627645889779d0,-12.97563038530094d0,&
      13.73722288814229d0,29.75867993904679d0,&
      18.78561017040914d0,31.62619890598152d0,&
      56.88510588958093d0,-25.23137735025941d0,&
      -25.82652403059675d0,-3.780337299284983d0,&
      -21.09823843977986d0,24.91291266047954d0,&
      35.36308254072654d0,-26.91869039634134d0,&
      20.96959418336273d0,-3.789948858236430d0,&
      16.70980615471843d0,27.13628186455847d0,&
      -62.16610066889994d0,26.96321858469311d0,&
      26.02984616864774d0,10.04602856133049d0,&
      29.15039568535510d0,7.861891187565258d0,&
      -28.68229976165538d0,16.34238980191444d0,&
```

```
     -8.169658652714595d0,-28.60538432424299d0,&
     -24.39459454254140d0,-21.18044542012355d0,&
     28.01860975501161d0,3.805300256977252d0,&
     -28.90752767525595d0,-25.64422437120984d0,&
     -25.52245198588326d0,-24.86105735578430d0,&
     30.01526520405102d0,-38.91642226433564d0,&
     22.63161332386640d0,-9.033297532931176d0,&
     4.352039687418654d0,11.51783214621507d0,&
     -12.87214271175449d0,-16.20720418149440d0,&
     17.53723312994921d0,11.04412046637780d0/
!
    data (c_a(k), k=1,60)/-10.57292309985323d0,171.0785194662196d0,&
     -1.730148127237479d0,15.32160252949849d0,&
     -6.286646201447911d0,50.30949287496870d0,&
     40.77966847313574d0,15.46616261435782d0,&
     -20.31248242835245d0,-45.83908871844293d0,&
     0.1197850397967530d0,-17.40758409621311d0,&
     -57.21365572412546d0,-42.73615802852128d0,&
     -1.191941420109328d0,24.83509056651268d0,&
     -95.30070554907265d0,-39.66415139202428d0,&
     0.4086066391364306d0,9.923370569792926d0,&
     0.1346009017990180d0,56.17144295471162d0,&
     -75.07291542876150d0,23.68886837644164d0,&
     -29.51063501736833d0,26.04552725392572d0,&
     1.191513707502110d0,-6.844347237025765d0,&
     21.77084021040513d0,-10.40424133679715d0,&
     45.81942140460026d0,1.113675918420691d0,&
     11.11947095486680d0,-7.537007526636058d0,&
     -26.96698839061458d0,27.72386179897775d0,&
     91.26831925380907d0,7.085201974906484d0,&
     86.98927999649095d0,0.3711106042302713d0,&
     -213.4697173470782d0,-0.1124143971223241d0,&
     65.21209907549108d0,19.00594294275638d0,&
     35.00693410348447d0,14.76412666483790d0,&
     -19.46876920313840d0,-13.66411478643766d0,&
     -1.763470250111077d0,58.80697032692803d0,&
     -23.79669894105976d0,0.3259758215525203d0,&
     7.233643919954782d0,-54.14047744135023d0,&
     50.96754852668879d0,-35.54152959550130d0,&
     70.03915878075998d0,0.3923242484523975d0,&
     -29.03604538898916d0,10.84905322248760d0/
!
    data (d_a(k), k=1,240)/15.65625221387963d0,0.1562391679714117d0,&
     2.912207558738881d0,-0.5726011306920963d0,&
     0.3833354294698882d0,1.122107425037348d0,&
     1.848140200553839d0,0.9693104267360982d0,&
     0.2543762231448159d0,1.490028775275346d0,&
     8.965571288422556d0,0.1161527621333683d0,&
     0.8752460149694253d0,-0.5521440543867698d0,&
     -7.976554367132331d0,0.9950340521747238d0,&
```

```
-0.5721834936147964d0,-6.173751217276735d0,&
-2.219010211560325d0,-4.496617341548901d0,&
-3.748662043068931d0,1.838018439321210d0,&
5.426764930593002d0,0.1333133315808171d0,&
0.8368841652527396d0,-0.8282769259798997d0,&
-7.987860745917458d0,2.492319403606216d0,&
-3.265522571740285d0,0.1316689846983124d0,&
-1.401195748630966d0,-2.700308648505325d0,&
15.69864064275405d0,-2.539017116302983d0,&
-6.015082756767647d0,-1.134324997320676d0,&
-5.116035627650986d0,-0.3059298988194926d0,&
3.781108582065893d0,-0.6805232695512022d0,&
0.5387680690693175d0,-0.1647557422708346d0,&
0.9709569662749630d0,3.408864850718117d0,&
-2.778536393173127d0,-0.1350450886227508d0,&
2.902144442170136d0,2.592552090007236d0,&
7.577023441592702d0,4.486748395357534d0,&
-3.795711330424958d0,1.293710370610740d0,&
-1.081915201057759d0,1.596810842001178d0,&
-0.1565865065949827d0,-0.1190909048459924d0,&
0.3186677795135865d0,0.6987205176209779d0,&
-1.448324580486081d0,-0.3582997722528971d0,&
1.452889477993051d0,0.5253909715307341d0,&
0.1614494668192123d0,-0.2632570652016132d0,&
1.016001904833059d0,-0.4922584673385586d0,&
0.1269718598367762d0,1.915809025362198d0,&
0.9770593618706195d0,2.169170796370391d0,&
2.519416400910442d0,-1.022472978735587d0,&
2.437975387157709d0,-0.3837924059794059d0,&
6.349902282313936d0,0.6606354210178313d0,&
-0.2398958628591118d0,3.739346491857064d0,&
0.7934581989313714d0,-6.765352855559168d0,&
-2.325273421428352d0,2.208645726437295d0,&
1.748157821098672d0,-0.6257322544544577d0,&
0.3805326466009046d0,-3.592785904914648d0,&
6.351957677261665d0,0.04014416360099259d0,&
1.120289209723204d0,-0.7164764046869387d0,&
1.035666458634682d0,0.5129928775173179d0,&
1.446546707193758d0,0.03010209483335512d0,&
-2.020751352046620d0,0.4919573450281849d0,&
3.786760292935898d0,-1.028026203843857d0,&
-3.579097585751665d0,-3.073673729486270d0,&
0.4011782785380622d0,-1.545504210023685d0,&
3.270213385797168d0,-1.171363156661823d0,&
3.260284198729962d0,0.5253463447806405d0,&
4.915625880835646d0,2.261433478997845d0,&
-1.673974947823282d0,1.617252010648383d0,&
-5.713634479755354d0,2.888665244700904d0,&
-1.679473904218543d0,0.1319938246346201d0,&
-0.5304524055310329d0,0.8802211247022720d0,&
```

```
0.6741547373426832d0,2.936217310077469d0,&
1.151510284946079d0,-0.2915723668370288d0,&
-0.7770475362197317d0,-0.01596335615066839d0,&
-0.02042078729153381d0,0.2046157747547349d0,&
-0.04061800283781668d0,0.06821574805383518d0,&
-0.01384939119746214d0,-0.6576016952857504d0,&
-0.06707452443179542d0,-0.1112574041413953d0,&
-0.2078773070510782d0,-0.04475167205219401d0,&
-0.6045429690303962d0,0.003844030083926171d0,&
0.03674478025895466d0,-0.007552963607158985d0,&
0.1865029091255489d0,0.02468984918444309d0,&
-0.1553774201915424d0,0.4350751458808970d0,&
-0.08653563162798289d0,-0.1161604157074527d0,&
-0.2081711693563556d0,0.007126124232343465d0,&
-0.2398888223324485d0,0.5462788269674786d0,&
0.03613126410009609d0,0.1045807971973989d0,&
-0.1150853472486007d0,0.009146129768431913d0,&
-0.05785265221759058d0,-0.1679710757327208d0,&
-0.7570504455818299d0,-0.1124117322889771d0,&
0.3482842930507742d0,-0.07004652734465672d0,&
0.1248159351533727d0,0.03467256526644854d0,&
-0.1936935405071153d0,0.1558772700903902d0,&
-0.002646548334432621d0,0.009012712228566753d0,&
-0.6582618553875765d0,0.1104286598858885d0,&
0.2092498904120032d0,-0.004937403021480120d0,&
-0.1584168875281193d0,-0.1302835628636813d0,&
0.2279479878760966d0,-0.1378351564484813d0,&
0.1992110423040408d0,-0.1377904905235225d0,&
0.7056542784163685d0,-0.01374013273063264d0,&
0.01606581292504723d0,0.03956746701628629d0,&
-0.04233143761926530d0,-0.4586419231066378d0,&
-0.04917269887327144d0,0.05162964643643641d0,&
-48.27862575851360d0,-3.739841652692242d0,&
-7.733093566634753d0,12.38924806486135d0,&
-13.23894005984265d0,-9.131118107204566d0,&
-7.082710960661152d0,-19.57652124335474d0,&
-19.44719734769592d0,-23.18156643392939d0,&
-30.11306460555098d0,-10.04239445064234d0,&
-17.91596387864566d0,6.712115072865807d0,&
28.08037856572131d0,-11.87306747537430d0,&
12.23350384919486d0,20.60384738115368d0,&
18.00142151041149d0,22.70506526841650d0,&
55.39836002316227d0,-23.25615512178527d0,&
-19.41162247152143d0,-3.090914854173156d0,&
-19.56474752154284d0,21.33312024261697d0,&
28.06294450835755d0,-26.15488442777154d0,&
17.97984362008619d0,-3.089684903279639d0,&
14.61985931979215d0,26.04640939408945d0,&
-47.88735440486467d0,26.15184444091908d0,&
18.72078097215553d0,9.100929961347092d0,&
```

```
     20.78588294041514d0,7.383644849123677d0,&
     -21.66703403158201d0,15.24540794716489d0,&
     -6.739298359057971d0,-28.76170432783672d0,&
     -20.92960065632837d0,-18.03000600036907d0,&
     22.20611797569871d0,3.095727502210388d0,&
     -23.35922023997328d0,-23.07546084881157d0,&
     -17.34118629845213d0,-19.40735821654657d0,&
     22.94141424794321d0,-38.34114088737283d0,&
     21.05088064381503d0,-7.021228850869575d0,&
     3.747680047153797d0,9.885533489585544d0,&
     -11.74706765859286d0,-14.83135666136258d0,&
     15.44364831291260d0,10.69156553664594d0/
!
     if ( R < 1.6d0) R = 1.6d0
     if (abs(beta) > 90.d0) then ! in MOLSCAT beta: -180 to 180
       beta = 90.d0 - (abs(beta) - 90.d0)
     else
       beta = abs(beta)
     endif
!  ===== NEURAL NETWORK CONSTANTS =====
!  Input 1
!  Layer 1
!  Layer 2
!
!  ===== SIMULATION ========
!  Dimensions
!  samples
!  Input 1
!  ===== MODULE FUNCTIONS ========
!  Map Minimum and Maximum Input Processing Function
!
     a_idx_1 = alpha * 0.0111111111111111d0
     a_idx_2 = beta * 0.0222222222222222d0
     a_idx_3 = (R - 1.0d0) * 0.181818181818182d0
     xp1_idx_0 = theta * 0.0111111111111111d0 -1.0d0
!
!  Layer 1
!  Sigmoid Symmetric Transfer Function
!  Layer 2
!  Output 1
!  Map Minimum and Maximum Output Reverse-Processing Function
!
     a = 0.0d0
     do k = 1,60
      b(k) = b_a(k)
      a = a+ c_a(k) * (2.0d0 / (1.0d0 + dexp(-2.0d0 * (b(k) + &
       (((d_a(k) * xp1_idx_0 + d_a(k+60) * (a_idx_1 - 1.0)) + &
       d_a(k + 120) * (a_idx_2 -1.0d0)) + d_a(k + 180) &
      *(a_idx_3 -1.0d0))))) - 1.0d0)
     enddo
```

```
!
      Vnn=((44.370995771560544d0 + a) +1.0d0) / 6.57831474660652E-6 &
       -10964.151d0
!
!-------------------------------------------------------------------
      vlr = (- 0.2030d0/R**4 + (0.2809d0*cos(PI*theta/180.d0))/R**5)*219478.d0
      xx  = exp((R-11.d0)/0.5d0) + 1.0d0
      swi = 1.0d0/xx
      V  = vlr*(1.0d0-swi)+swi*Vnn
!~~~~~~~~~~~~~~~~~~~~~~~~~~~~~~~~~~~~~~~~~~~~~~~~~~~~~~~~~~~~~~~~
      RETURN
!
      END SUBROUTINE
```

Table S1: Computed excitation cross sections in Å^2 units for a series of inelastic processes generated using the 4D RR-PES for the $HeH^+(j_1)\cdots$ para-$H_2(j_2)$ system, with both $\Delta j_1 = +1$ and $\Delta j_1 = +2$.

E_{trans}(cm^{-1})	00->10	E_{trans}(cm^{-1})	00->20	E_{trans}(cm^{-1})	02->12	02->22
67.06	2.68388	201.16	0.47515	85.20	28.12660	
67.16	5.53681	201.26	1.52960	105.20	34.81430	
67.26	10.94220	201.36	1.43630	125.20	38.50480	
70.00	21.35220	210.00	9.86615	145.20	40.64130	
90.00	35.33510	230.00	12.84950	165.20	37.80370	
110.00	40.80880	250.00	17.18160	185.20	38.78430	
130.00	35.22080	270.00	20.21900	205.20	37.27780	4.57315
150.00	55.16750	290.00	22.00650	225.20	32.04560	10.70730
170.00	46.09690	310.00	27.81510	245.20	30.81310	12.98090
190.00	50.19320	330.00	21.61320	265.20	31.13990	15.08290
210.00	35.04050	350.00	21.98080	285.20	28.99050	16.72040
230.00	39.26410	370.00	19.84080	305.20	29.55730	16.52530
250.00	32.20010	390.00	21.67140	325.20	29.30170	18.06210
270.00	29.09360	410.00	23.66240	345.20	25.98760	19.48840
290.00	29.59070	430.00	21.63410	365.20	27.08320	18.39020
310.00	33.20850	450.00	23.30470	385.20	27.05500	18.01080
330.00	28.73770	470.00	18.65880	405.20	25.90740	18.76420
350.00	33.92040	490.00	16.24590	425.20	26.78400	17.56730
370.00	34.62520	510.00	18.68770	445.20	24.55300	16.47410
390.00	38.71180	530.00	18.73150	465.20	24.58820	16.61980
410.00	25.32070	550.00	16.62430	485.20	26.41180	15.14900
430.00	29.53350	570.00	15.49250	505.20	26.27570	14.45720
450.00	29.77770	590.00	15.76840	525.20	25.37410	14.98760
470.00	26.65870	610.00	15.15310	545.20	25.83310	15.15930
490.00	23.22250	630.00	14.19520	565.20	25.61320	15.13390
510.00	24.39330	650.00	11.72900	585.20	26.45390	14.54100
530.00	26.84390	670.00	13.58960	605.20	25.91590	14.61160
550.00	30.84610	690.00	14.10130	625.20	24.69290	14.80290
570.00	29.56200	710.00	13.50660	645.20	25.91490	15.04500
590.00	23.65250	730.00	15.15030	665.20	25.01020	14.33740
610.00	24.79080	750.00	13.22840	685.20	24.71790	14.14520

630.00	26.45760	770.00	12.35400	705.20	24.94850	13.94120
650.00	30.40310	790.00	11.74360	725.20	23.94970	13.78860
670.00	25.57460	810.00	11.17850	745.20	24.20180	13.62040
690.00	20.03430	830.00	10.78190	765.20	24.45070	13.38980
710.00	26.18050	850.00	11.79480	785.20	24.47250	13.18690
730.00	24.41430	870.00	12.47700	805.20	24.15070	13.25990
750.00	23.53030	890.00	11.64790	825.20	24.08340	13.01620
770.00	23.79380	910.00	10.36880	845.20	23.53080	12.98410
790.00	24.12930	930.00	9.73710	865.20	23.49120	13.46160
810.00	24.92640	950.00	8.82243	885.20	23.58120	12.80320
830.00	27.28170	970.00	10.06820	905.20	23.54650	12.68050
850.00	22.74660	990.00	9.81031	925.20	23.61870	12.72520
870.00	22.40480	1010.00	10.19540	945.20	22.88030	12.44410
890.00	20.69670	1030.00	10.39370	965.20	22.53200	12.67960
910.00	21.97640	1050.00	10.44070	985.20	22.35160	12.55580
930.00	24.43870	1070.00	9.86576	1005.20	22.02650	12.75860
950.00	20.84510	1090.00	10.03640	1025.20	21.64910	12.80350
970.00	21.44500	1110.00	9.57006	1045.20	21.84210	12.95790
990.00	19.85040	1130.00	10.27040	1065.20	21.70670	12.63030
1010.00	22.10260	1150.00	10.33040	1085.20	21.53150	12.64850
1030.00	22.00440	1170.00	9.18859	1105.20	21.44760	12.32690
1050.00	22.42480	1190.00	9.69258	1125.20	21.16350	11.93030
1070.00	21.42910	1210.00	9.26601	1145.20	20.71090	12.16750
1090.00	21.37550	1230.00	9.59550	1165.20	20.41810	11.95560
1110.00	21.12850	1250.00	9.15073	1185.20	20.49770	12.22310
1130.00	21.31660	1270.00	10.22010	1205.20	20.33120	12.32240
1150.00	21.86690	1290.00	10.81840	1225.20	20.26900	11.96490
1170.00	20.85370	1310.00	10.82020	1245.20	20.28210	12.00780
1190.00	18.57290	1330.00	9.21793	1265.20	19.99630	11.91840
1210.00	19.61830	1350.00	9.65212	1285.20	19.96480	11.57030
1230.00	20.40840	1370.00	8.81324	1305.20	19.72550	11.69340
1250.00	19.02750	1390.00	9.14460	1325.20	19.67880	11.19590
1270.00	18.68370	1410.00	10.07650	1345.20	20.20460	11.27850
1290.00	20.37500	1430.00	9.46108	1365.20	19.49600	11.28160
1310.00	19.62610	1450.00	8.47014	1385.20	19.98120	11.00450
1330.00	18.57960	1470.00	9.22643	1405.20	19.79030	11.44930
1350.00	18.68840	1490.00	9.17469	1425.20	19.48880	11.03960
1370.00	17.95340	1510.00	10.07420	1445.20	19.64930	10.58850
1390.00	19.15850	1530.00	9.32175	1465.20	19.67690	10.64060
1410.00	17.39780	1550.00	8.42286	1485.20	19.58120	10.60180
1430.00	18.51630	1570.00	8.13249	1505.20	19.72180	10.56920
1450.00	18.01870	1590.00	9.13965	1525.20	19.55440	10.56190
1470.00	19.20350	1610.00	9.02636	1545.20	19.35080	10.27990
1490.00	19.00020	1630.00	8.76509	1565.20	19.27510	10.14690
1510.00	19.02180	1650.00	7.72332	1585.20	19.19720	10.39280
1530.00	18.64080	1670.00	7.84074	1605.20	19.15180	10.22990
1550.00	18.40940	1690.00	8.81319	1625.20	18.92070	10.38440
1570.00	18.80490	1710.00	8.70687	1645.20	18.72490	10.24600
1590.00	19.08100	1730.00	8.45236	1665.20	18.83820	10.12120
1610.00	18.98230	1750.00	8.63426	1685.20	19.00980	9.65040

1630.00	18.81910	1770.00	8.21037	1705.20	18.96820	9.52218
1650.00	17.23000	1790.00	8.56510	1725.20	18.29520	10.04100
1670.00	19.22050	1810.00	7.49498	1745.20	18.01470	9.82320
1690.00	18.99060	1830.00	7.30416	1765.20	17.99140	9.88283
1710.00	19.15590	1850.00	8.55707	1785.20	18.12990	9.59874
1730.00	17.97060	1870.00	8.53404	1805.20	18.11350	9.60731
1750.00	18.12980	1890.00	8.68531	1825.20	17.99720	9.79203
1770.00	18.23910	1910.00	8.88111	1845.20	17.84870	9.48176
1790.00	18.49710	1930.00	8.64098	1865.20	17.82840	8.85699
1810.00	17.42590	1950.00	7.80483	1885.20	17.44530	9.08987
1830.00	18.31600	1970.00	7.33763	1905.20	17.69900	9.18798
1850.00	17.56250	1990.00	6.93866	1925.20	17.06000	9.39761
1870.00	17.48140	2010.00	7.29899	1945.20	17.33910	9.23910
1890.00	17.55040	2030.00	7.85352	1965.20	17.13650	9.13072
1910.00	16.72490	2050.00	8.04581	1985.20	17.07870	9.00514
1930.00	16.21370	2070.00	8.55148	2005.20	17.01430	8.83953
1950.00	16.44090	2090.00	8.35139	2025.20	16.88180	8.99349
1970.00	16.78210	2110.00	8.37880	2045.20	16.72160	8.94637
1990.00	17.30120	2130.00	8.33480	2065.20	16.59660	8.73256
2010.00	16.61720	2150.00	7.91899	2085.20	17.02820	8.62998
2030.00	16.47370	2170.00	7.36808	2105.20	16.82130	8.61256
2050.00	16.35880	2190.00	7.80792	2125.20	16.76100	8.52979
2070.00	17.09420	2210.00	7.31997	2145.20	16.55550	8.54179
2090.00	15.72710	2230.00	7.63507	2165.20	16.44720	8.55470
2110.00	15.58800	2250.00	7.56586	2185.20	16.43250	8.43340
2130.00	15.87680	2270.00	7.03859	2205.20	16.39810	8.16283
2150.00	15.69620	2290.00	7.59629	2225.20	16.74830	8.06342
2170.00	15.89090	2310.00	8.12513	2245.20	16.40130	8.02726
2190.00	15.60630	2330.00	7.76358	2265.20	16.31010	8.01720
2210.00	16.07600	2350.00	7.59171	2285.20	16.08830	7.89653
2230.00	16.06460	2370.00	7.04467	2305.20	16.35970	7.72501
2250.00	15.78450	2390.00	7.05896	2325.20	16.40550	7.91488
2270.00	16.13830	2410.00	7.09017	2345.20	16.28930	7.95573
2290.00	15.63060	2430.00	6.60712	2365.20	15.98120	7.92107
2310.00	15.06190	2450.00	6.47176	2385.20	16.08140	7.73857
2330.00	15.34360	2470.00	6.12294	2405.20	16.17360	7.48320
2350.00	15.10980	2490.00	6.44381	2425.20	16.06530	7.49858
2370.00	15.59040	2510.00	7.07220	2445.20	16.00540	7.36544
2390.00	15.26810	2530.00	6.88455	2465.20	15.88610	7.48477
2410.00	14.97130	2550.00	6.42946	2485.20	15.59140	7.48199
2430.00	15.16800	2570.00	6.96406	2505.20	15.78420	7.33552
2450.00	15.41880	2590.00	6.17105	2525.20	15.79100	7.18658
2470.00	15.15390	2610.00	6.00743	2545.20	15.97260	7.33068
2490.00	15.08210	2630.00	6.18804	2565.20	15.77030	7.42276
2510.00	15.75040	2650.00	5.79285	2585.20	15.83790	7.26666
2530.00	14.57690	2670.00	5.80369	2605.20	15.77180	7.34305
2550.00	14.74430	2690.00	6.00689	2625.20	15.36510	7.32312
2570.00	13.78670	2710.00	6.31813	2645.20	15.06310	7.27849
2590.00	14.39390	2730.00	6.32690	2665.20	15.27170	7.13690
2610.00	14.79510	2750.00	6.08421	2685.20	15.31410	7.23946

2630.00	13.89040	2770.00	6.14735	2705.20	15.15560	7.21849
2650.00	14.25580	2790.00	6.09341	2725.20	15.05390	7.10920
2670.00	14.93230	2810.00	6.09450	2745.20	15.37390	7.04090
2690.00	14.07140	2830.00	6.16491	2765.20	15.38490	7.12356
2710.00	14.58490	2850.00	5.89885			
2730.00	14.19350	2870.00	5.87712			
2750.00	14.01160	2890.00	5.73927			
2770.00	13.82590	2910.00	5.82199			
2790.00	13.45820	2930.00	6.10997			
2810.00	13.46700	2950.00	6.06168			
2830.00	13.34590	2970.00	5.98185			
2850.00	13.31630	2990.00	5.90447			
2870.00	13.01950	3010.00	5.77229			
2890.00	13.41400	3030.00	5.89241			
2910.00	13.65860	3050.00	5.92071			
2930.00	13.53080	3070.00	5.90063			
2950.00	13.48220	3090.00	5.64160			
2970.00	13.23640	3110.00	5.54783			
2990.00	13.38140	3130.00	5.51839			
3010.00	13.24720					
3030.00	13.03990					
3050.00	12.68790					
3070.00	12.64750					
3090.00	12.56170					
3110.00	12.46170					
3130.00	12.80620					

E_{trans}(cm^{-1})	10->20	E_{trans}(cm^{-1})	10->30	E_{trans}(cm^{-1})	12->22	12->32
134.108	0.48023	335.270	0.29179	138.148	8.13653	
134.208	1.89221	335.370	0.85498	158.148	17.48580	
134.308	2.06616	335.470	1.07464	178.148	21.99200	
142.948	15.29690	342.950	2.58142	198.148	25.47600	
162.948	21.39270	362.950	5.08490	218.148	26.24600	
182.948	25.80990	382.950	6.10618	238.148	26.00460	
202.948	29.06840	402.950	6.92505	258.148	26.09260	
222.948	29.61330	422.950	7.00301	278.148	26.23260	
242.948	31.13650	442.950	8.48979	298.148	25.37200	
262.948	30.63340	462.950	8.04443	318.148	25.41820	
282.948	25.62800	482.950	8.09477	338.148	24.61720	2.12831
302.948	33.43470	502.950	7.13235	358.148	22.66450	5.46404
322.948	27.29140	522.950	7.63958	378.148	21.62560	7.68300
342.948	27.06040	542.950	9.18140	398.148	21.50790	8.91457
362.948	22.66290	562.950	8.17270	418.148	20.72040	9.48100
382.948	23.50300	582.950	7.13533	438.148	19.95820	10.37990
402.948	18.87390	602.950	6.45325	458.148	19.65220	10.90590
422.948	18.45900	622.950	7.81156	478.148	18.88070	11.69610
442.948	17.79810	642.950	8.84838	498.148	18.26730	11.96980
462.948	18.19250	662.950	7.61051	518.148	18.56230	12.13530
482.948	18.79400	682.950	7.63463	538.148	18.14640	12.67650
502.948	16.64030	702.950	7.23993	558.148	18.04610	12.75380

522.948	16.38620	722.950	7.16483	578.148	17.90710	12.96190
542.948	16.66070	742.950	6.33999	598.148	17.24720	13.22310
562.948	15.82520	762.950	6.39179	618.148	17.12090	12.25780
582.948	14.79230	782.950	6.01292	638.148	16.73800	11.91700
602.948	15.14990	802.950	5.89536	658.148	16.60260	11.45030
622.948	12.60270	822.950	5.89888	678.148	16.32750	11.12050
642.948	13.13370	842.950	6.18611	698.148	16.43240	10.94580
662.948	12.64750	862.950	6.05401	718.148	16.43440	10.85260
682.948	13.16310	882.950	6.25233	738.148	16.37170	10.81670
702.948	13.92850	902.950	6.48668	758.148	15.65410	10.45140
722.948	13.02700	922.950	5.92470	778.148	15.80880	10.42830
742.948	12.75690	942.950	6.14945	798.148	16.08410	10.33210
762.948	12.47350	962.950	6.27687	818.148	15.68360	10.25330
782.948	13.83470	982.950	5.31532	838.148	15.80150	10.08880
802.948	13.18730	1002.950	5.90604	858.148	15.65220	9.88917
822.948	13.67220	1022.950	5.84452	878.148	15.62710	9.64041
842.948	12.38010	1042.950	5.86640	898.148	15.41060	9.56987
862.948	12.04960	1062.950	5.68284	918.148	15.38580	9.55619
882.948	12.95380	1082.950	5.80202	938.148	15.06220	9.26252
902.948	13.29620	1102.950	5.65008	958.148	14.60930	8.84911
922.948	13.19680	1122.950	5.72761	978.148	14.46390	8.83416
942.948	13.57070	1142.950	5.52739	998.148	14.61440	8.69437
962.948	13.54760	1162.950	5.68032	1018.148	14.64750	8.40952
982.948	13.76580	1182.950	5.82040	1038.148	14.66070	8.30605
1002.948	13.33880	1202.950	6.24379	1058.148	14.49350	8.18537
1022.948	11.95860	1222.950	5.84997	1078.148	14.13290	8.13288
1042.948	11.96030	1242.950	6.02001	1098.148	14.28840	8.13519
1062.948	11.47150	1262.950	5.42193	1118.148	14.15210	7.93883
1082.948	11.32420	1282.950	5.36962	1138.148	14.04380	7.82137
1102.948	12.90060	1302.950	5.48858	1158.148	14.15320	7.90040
1122.948	12.50200	1322.950	5.37508	1178.148	14.15690	7.74133
1142.948	13.02770	1342.950	5.63921	1198.148	13.92380	7.58801
1162.948	12.30970	1362.950	5.71501	1218.148	13.76020	7.51014
1182.948	12.36320	1382.950	5.76042	1238.148	13.69950	7.58781
1202.948	11.10760	1402.950	5.24159	1258.148	13.67380	7.37289
1222.948	11.58730	1422.950	5.16926	1278.148	13.68210	7.31944
1242.948	11.47000	1442.950	5.21413	1298.148	13.53870	7.16599
1262.948	11.97090	1462.950	4.62828	1318.148	13.70450	7.07924
1282.948	12.16160	1482.950	5.13115	1338.148	13.76590	7.13964
1302.948	12.75340	1502.950	5.18364	1358.148	13.59610	7.07575
1322.948	12.35930	1522.950	5.34101	1378.148	13.51330	6.98369
1342.948	11.80900	1542.950	4.97030	1398.148	13.50890	7.00773
1362.948	11.99110	1562.950	4.88164	1418.148	13.21680	6.78146
1382.948	11.23630	1582.950	5.18841	1438.148	13.39470	6.77597
1402.948	11.66620	1602.950	4.77818	1458.148	13.45180	6.76633
1422.948	11.67270	1622.950	4.71010	1478.148	13.35180	6.59335
1442.948	12.26350	1642.950	4.79555	1498.148	13.39280	6.58193
1462.948	12.13080	1662.950	5.39809	1518.148	13.36550	6.54432
1482.948	12.42670	1682.950	4.91292	1538.148	13.08540	6.55174
1502.948	12.45020	1702.950	5.11789	1558.148	13.06670	6.55521

1522.948	11.60510	1722.950	5.25222	1578.148	13.01920	6.48626
1542.948	11.06210	1742.950	4.98370	1598.148	13.02100	6.42675
1562.948	11.49330	1762.950	4.93574	1618.148	13.09140	6.40810
1582.948	11.44290	1782.950	4.84121	1638.148	13.16760	6.43468
1602.948	11.52990	1802.950	4.90662	1658.148	12.88040	6.46840
1622.948	11.85470	1822.950	5.08765	1678.148	12.77140	6.48897
1642.948	11.83660	1842.950	5.11353	1698.148	12.67720	6.31019
1662.948	11.69090	1862.950	4.93808	1718.148	12.74000	6.36096
1682.948	11.48820	1882.950	5.10421	1738.148	12.83710	6.34135
1702.948	11.34150	1902.950	5.08209	1758.148	12.86070	6.28990
1722.948	11.09390	1922.950	5.07081	1778.148	12.47430	6.32451
1742.948	11.20280	1942.950	4.95597	1798.148	12.21410	6.17247
1762.948	11.70560	1962.950	4.80937	1818.148	12.13780	6.16279
1782.948	11.13830	1982.950	4.93926	1838.148	12.45150	6.28523
1802.948	11.50570	2002.950	4.91533	1858.148	12.36020	6.20608
1822.948	11.23640	2022.950	5.01461	1878.148	12.44320	6.21429
1842.948	11.25380	2042.950	4.94866	1898.148	12.49440	6.17140
1862.948	10.92480	2062.950	5.26525	1918.148	12.42330	6.24397
1882.948	11.06570	2082.950	5.34038	1938.148	12.38000	6.26552
1902.948	10.55740	2102.950	5.21822	1958.148	12.25080	6.24436
1922.948	10.94250	2122.950	5.11516	1978.148	12.12080	6.23047
1942.948	10.83820	2142.950	4.86788	1998.148	11.99070	6.14725
1962.948	10.68430	2162.950	4.92966	2018.148	12.16650	6.12026
1982.948	11.02560	2182.950	4.74866	2038.148	12.09040	6.06149
2002.948	10.98060	2202.950	4.76386	2058.148	12.07970	6.06651
2022.948	10.84580	2222.950	4.83937	2078.148	12.05410	6.08788
2042.948	10.74910	2242.950	5.21367	2098.148	12.01320	6.10778
2062.948	10.51990	2262.950	5.06509	2118.148	11.85780	6.12021
2082.948	10.69750	2282.950	5.22291	2138.148	11.85770	6.08745
2102.948	10.56720	2302.950	5.16387	2158.148	11.84470	6.06671
2122.948	10.25570	2322.950	4.89681	2178.148	11.74450	6.02857
2142.948	10.69700	2342.950	4.76997	2198.148	10.65300	5.38184
2162.948	10.86180	2362.950	4.70309	2218.148	10.50590	5.12598
2182.948	10.76830	2382.950	4.66424	2238.148	11.67910	5.87615
2202.948	10.70730	2402.950	4.74924	2258.148	11.59570	5.78973
2222.948	10.62270	2422.950	4.54996	2278.148	11.59310	5.86187
2242.948	10.49750	2442.950	4.68981	2298.148	11.56020	5.82593
2262.948	10.54800	2462.950	4.68649	2318.148	11.50690	5.82921
2282.948	10.72850	2482.950	4.98562	2338.148	11.46510	5.81024
2302.948	10.44600	2502.950	5.10701	2358.148	11.44590	5.82078
2322.948	10.36680	2522.950	4.77246	2378.148	11.38540	5.83226
2342.948	10.55030	2542.950	4.71207	2398.148	11.29550	5.83268
2362.948	10.44640	2562.950	4.84986	2418.148	11.28540	5.77989
2382.948	10.55260	2582.950	4.85638	2438.148	11.32190	5.68788
2402.948	10.45590	2602.950	4.57792	2458.148	11.31080	5.65632
2422.948	10.42210	2622.950	4.67767	2478.148	11.30920	5.60848
2442.948	10.64870	2642.950	4.58687	2498.148	11.22980	5.59324
2462.948	10.46420	2662.950	4.50213	2518.148	11.23510	5.61098
2482.948	10.49670	2682.950	4.72101	2538.148	11.22830	5.66291
2502.948	10.08830	2702.950	4.94078	2558.148	11.14310	5.71817

2522.948	10.13710	2722.950	4.90824	2578.148	11.09340	5.72745
2542.948	10.26540	2742.950	4.69438	2598.148	11.07080	5.66761
2562.948	10.23450	2762.950	4.62172	2618.148	11.07030	5.63290
2582.948	10.13060	2782.950	4.70598	2638.148	10.94450	5.56540
2602.948	9.97011	2802.950	4.65934	2658.148	10.96530	5.54306
2622.948	10.01470	2822.950	4.42687	2678.148	11.00010	5.47387
2642.948	10.11490	2842.950	4.38619	2698.148	10.95530	5.49174
2662.948	10.06930	2862.950	4.37865			
2682.948	9.79586	2882.950	4.45637			
2702.948	9.67973	2902.950	4.58318			
2722.948	9.66431	2922.950	4.64139			
2742.948	9.76214	2942.950	4.51230			
2762.948	9.68118	2962.950	4.33911			
2782.948	9.66146	2982.950	4.42849			
2802.948	9.35626	3002.950	4.50334			
2822.948	9.44876	3022.950	4.48397			
2842.948	9.55626	3042.950	4.34465			
2862.948	9.60808	3062.950	4.21314			
2882.948	9.52450					
2902.948	9.51033					
2922.948	9.43215					
2942.948	9.39633					
2962.948	9.27541					
2982.948	9.09335					
3002.948	9.08645					
3022.948	8.91874					
3042.948	8.97574					
3062.948	9.18937					

E_{trans}(cm^{-1})	20->30	E_{trans}(cm^{-1})	20->40	E_{trans}(cm^{-1})	22->32	22->42
201.164	0.36517	469.374	0.08840	204.044	3.71886	
201.264	1.15988	469.474	0.10752	224.044	9.97796	
201.364	1.51133	469.574	0.13262	244.044	12.96730	
208.844	5.46833	488.844	1.52634	264.044	15.10940	
228.844	10.63250	508.844	2.43119	284.044	16.04480	
248.844	11.03240	528.844	2.91044	304.044	16.93390	
268.844	11.65340	548.844	3.33955	324.044	17.48980	
288.844	12.02120	568.844	3.63161	344.044	18.32970	
308.844	13.02750	588.844	3.80719	364.044	18.55570	
328.844	14.98800	608.844	3.76614	384.044	18.81800	
348.844	14.09490	628.844	4.36929	404.044	18.71350	
368.844	14.28170	648.844	4.41239	424.044	18.78240	
388.844	13.33270	668.844	4.28373	444.044	18.54140	
408.844	12.58760	688.844	4.46276	464.044	18.44580	
428.844	12.26660	708.844	4.68775	484.044	17.44980	2.39046
448.844	11.45010	728.844	5.01600	504.044	16.80070	3.99762
468.844	11.90780	748.844	4.70118	524.044	15.93330	5.07735
488.844	10.91340	768.844	4.94964	544.044	15.79990	5.97084
508.844	11.49580	788.844	4.88468	564.044	15.22630	6.66422
528.844	10.63170	808.844	4.74701	584.044	14.68950	7.00200

548.844	10.00470	828.844	4.79370	604.044	14.59440	7.33355
568.844	10.19130	848.844	4.46703	624.044	14.47430	7.72079
588.844	9.66838	868.844	4.72240	644.044	14.24090	8.02205
608.844	10.54770	888.844	4.63668	664.044	13.87140	8.13096
628.844	10.28880	908.844	4.68124	684.044	13.50700	8.30980
648.844	9.19669	928.844	4.80198	704.044	13.61980	8.18217
668.844	8.91764	948.844	4.81606	724.044	13.23180	8.44666
688.844	8.90693	968.844	4.62667	744.044	12.93940	8.51322
708.844	9.11380	988.844	4.68840	764.044	12.71960	8.32101
728.844	8.88021	1008.844	4.75235	784.044	12.50100	8.36819
748.844	9.08813	1028.844	4.82775	804.044	12.36840	8.47642
768.844	8.85002	1048.844	4.71140	824.044	11.63680	8.05060
788.844	9.20428	1068.844	4.64444	844.044	11.56980	7.85230
808.844	9.32984	1088.844	4.59384	864.044	11.12620	7.76077
828.844	8.30586	1108.844	4.57720	884.044	10.96990	7.50719
848.844	8.12806	1128.844	4.66902	904.044	10.77470	7.46243
868.844	7.86145	1148.844	4.49887	924.044	10.61670	7.30647
888.844	8.30378	1168.844	4.60662	944.044	10.43970	7.20441
908.844	8.26962	1188.844	4.70888	964.044	10.44410	7.09239
928.844	7.50202	1208.844	4.65746	984.044	10.23880	6.93852
948.844	8.24867	1228.844	4.56996	1004.044	10.29530	6.97248
968.844	7.84398	1248.844	4.59975	1024.044	10.13680	6.81500
988.844	7.35535	1268.844	4.54995	1044.044	10.13580	6.61643
1008.844	7.75620	1288.844	4.57847	1064.044	10.08030	6.52813
1028.844	7.38786	1308.844	4.64486	1084.044	9.89077	6.59658
1048.844	7.66225	1328.844	4.59208	1104.044	9.91349	6.57995
1068.844	7.47449	1348.844	4.62830	1124.044	9.89737	6.50528
1088.844	7.95964	1368.844	4.58084	1144.044	9.92184	6.40040
1108.844	8.23343	1388.844	4.54012	1164.044	9.84430	6.41139
1128.844	7.88350	1408.844	4.75867	1184.044	9.84305	6.31914
1148.844	7.64139	1428.844	4.56619	1204.044	9.71824	6.32784
1168.844	7.74217	1448.844	4.56384	1224.044	9.75320	6.16636
1188.844	7.58170	1468.844	4.47608	1244.044	9.63313	6.14766
1208.844	7.92364	1488.844	4.52114	1264.044	9.68458	6.04768
1228.844	7.85720	1508.844	4.63116	1284.044	9.76804	5.94976
1248.844	8.17274	1528.844	4.54857	1304.044	9.63996	5.94271
1268.844	7.87774	1548.844	4.60138	1324.044	9.65247	5.90871
1288.844	8.00992	1568.844	4.52192	1344.044	9.55457	5.85933
1308.844	7.73475	1588.844	4.54753	1364.044	9.55185	5.82704
1328.844	7.57565	1608.844	4.50157	1384.044	9.44630	5.83647
1348.844	7.90056	1628.844	4.43315	1404.044	9.55234	5.79251
1368.844	7.92812	1648.844	4.59281	1424.044	9.48955	5.72762
1388.844	7.91922	1668.844	4.46906	1444.044	9.48471	5.72233
1408.844	7.84286	1688.844	4.39147	1464.044	9.48530	5.66125
1428.844	7.93288	1708.844	4.45433	1484.044	9.38563	5.67196
1448.844	7.80998	1728.844	4.34988	1504.044	9.34133	5.60197
1468.844	7.76396	1748.844	4.38965	1524.044	9.26812	5.60437
1488.844	7.78579	1768.844	4.47274	1544.044	9.30877	5.60626
1508.844	7.83796	1788.844	4.57414	1564.044	9.32285	5.54729
1528.844	7.94638	1808.844	4.42867	1584.044	9.44628	5.49728

1548.844	8.11172	1828.844	4.50536	1604.044	9.37038	5.55189
1568.844	8.03650	1848.844	4.32202	1624.044	9.36601	5.50431
1588.844	7.88181	1868.844	4.31506	1644.044	9.21394	5.48254
1608.844	8.01121	1888.844	4.32866	1664.044	8.78192	5.40156
1628.844	8.14097	1908.844	4.46018	1684.044	8.67299	5.31063
1648.844	7.73638	1928.844	4.36513	1704.044	9.30112	5.37290
1668.844	7.88800	1948.844	4.46398	1724.044	9.27995	5.30378
1688.844	8.05513	1968.844	4.52906	1744.044	9.28338	5.29242
1708.844	8.10883	1988.844	4.36837	1764.044	9.31167	5.31197
1728.844	7.95838	2008.844	4.34496	1784.044	9.28074	5.29983
1748.844	8.15360	2028.844	4.24979	1804.044	9.24067	5.31588
1768.844	8.04317	2048.844	4.21100	1824.044	9.23648	5.28270
1788.844	8.01039	2068.844	4.18869	1844.044	9.22666	5.28488
1808.844	7.96843	2088.844	4.34054	1864.044	9.31480	5.23602
1828.844	7.89345	2108.844	4.34889	1884.044	9.34008	5.12286
1848.844	8.15090	2128.844	4.20976	1904.044	9.26341	5.06912
1868.844	8.05432	2148.844	4.28684	1924.044	9.33962	5.10064
1888.844	8.16151	2168.844	4.33692	1944.044	9.28893	5.12262
1908.844	7.97324	2188.844	4.22582	1964.044	9.28167	5.19506
1928.844	8.07442	2208.844	4.22651	1984.044	9.32273	5.16494
1948.844	8.23377	2228.844	4.14062	2004.044	9.35288	5.11117
1968.844	8.11268	2248.844	4.11458	2024.044	9.37974	5.11621
1988.844	8.01386	2268.844	4.15279	2044.044	9.35134	5.10418
2008.844	8.17985	2288.844	4.09811	2064.044	7.49844	4.54331
2028.844	8.21658	2308.844	4.05602	2084.044	7.29378	4.45960
2048.844	8.27707	2328.844	3.95793	2104.044	9.30500	4.99216
2068.844	8.29064	2348.844	4.13246	2124.044	9.31725	5.01846
2088.844	8.30066	2368.844	4.13400	2144.044	9.28341	5.06356
2108.844	8.07925	2388.844	4.12149	2164.044	9.34544	4.98659
2128.844	8.32595	2408.844	4.06109	2184.044	9.36591	5.00437
2148.844	8.30796	2428.844	4.19617	2204.044	9.38613	5.01241
2168.844	8.28327	2448.844	4.06491	2224.044	9.30604	4.99302
2188.844	8.10665	2468.844	4.01283	2244.044	9.22168	4.98830
2208.844	8.24751	2488.844	4.06972	2264.044	9.23766	4.99517
2228.844	8.16642	2508.844	3.91473	2284.044	9.17039	4.97029
2248.844	8.28938	2528.844	3.86934	2304.044	9.19150	4.97548
2268.844	8.38747	2548.844	3.91211	2324.044	9.15891	4.95444
2288.844	8.24019	2568.844	4.06911	2344.044	9.23133	4.96265
2308.844	8.20942	2588.844	4.07011	2364.044	9.21465	4.93102
2328.844	8.23840	2608.844	3.90260	2384.044	9.21302	4.95458
2348.844	8.29414	2628.844	3.93299	2404.044	9.13442	4.94078
2368.844	8.09773	2648.844	3.97313	2424.044	9.06065	4.95377
2388.844	8.18295	2668.844	3.92372	2444.044	9.02642	4.95186
2408.844	8.20111	2688.844	3.91062	2464.044	9.00754	4.92788
2428.844	8.23590	2708.844	3.87432	2484.044	8.97956	4.92398
2448.844	8.23602	2728.844	3.81406	2504.044	8.94039	4.90744
2468.844	8.15371	2748.844	3.84580	2524.044	8.93920	4.86907
2488.844	8.14176	2768.844	3.88720	2544.044	8.98550	4.87545
2508.844	8.19918	2788.844	3.91291	2564.044	8.94228	4.85933
2528.844	8.20741	2808.844	3.89367			

2548.844	8.13725	2828.844	3.80407
2568.844	8.12566	2848.844	3.87593
2588.844	8.07654	2868.844	3.85184
2608.844	7.90509	2888.844	3.81962
2628.844	8.10626	2908.844	3.80220
2648.844	8.05021	2928.844	3.74076
2668.844	7.96080		
2688.844	7.99072		
2708.844	7.99544		
2728.844	8.02947		
2748.844	8.01237		
2768.844	8.01700		
2788.844	7.99282		
2808.844	7.93756		
2828.844	7.74989		
2848.844	7.71941		
2868.844	7.81114		
2888.844	7.62653		
2908.844	7.69694		
2928.844	7.66423		

E_{trans}(cm^{-1})	30->40	E_{trans}(cm^{-1})	30->50	E_{trans}(cm^{-1})	32->42	32->52
268.218	0.25511	603.488	0.06029	282.888	5.40343	
268.318	0.31596	603.588	0.23535	302.888	8.19205	
268.418	0.47161	603.688	0.28424	322.888	10.11660	
287.688	3.80104	607.688	0.70311	342.888	11.10700	
307.688	5.03776	627.688	1.65305	362.888	12.32750	
327.688	6.51031	647.688	2.19382	382.888	12.78410	
347.688	7.02269	667.688	2.52723	402.888	13.43410	
367.688	7.51324	687.688	3.02263	422.888	13.87350	
387.688	7.88229	707.688	3.23290	442.888	14.09120	
407.688	8.03432	727.688	3.70089	462.888	14.07960	
427.688	8.05538	747.688	3.88936	482.888	13.76130	
447.688	8.11459	767.688	3.82853	502.888	13.96850	
467.688	8.13142	787.688	4.11618	522.888	13.77410	
487.688	8.27558	807.688	4.33472	542.888	13.88680	
507.688	7.95582	827.688	4.33291	562.888	13.61330	
527.688	8.07112	847.688	4.22199	582.888	13.54370	
547.688	8.33762	867.688	4.35873	602.888	13.69450	
567.688	8.15642	887.688	4.56430	622.888	12.79760	2.16263
587.688	7.96274	907.688	4.77932	642.888	12.58190	3.09269
607.688	8.49957	927.688	4.55195	662.888	11.95650	3.74414
627.688	8.36092	947.688	4.83363	682.888	11.68410	4.23797
647.688	7.77962	967.688	4.85082	702.888	11.34480	4.64434
667.688	7.64767	987.688	4.88688	722.888	11.11790	4.97801
687.688	7.93563	1007.688	4.93993	742.888	10.76760	5.18480
707.688	7.52227	1027.688	4.84482	762.888	10.45910	5.45162
727.688	7.48619	1047.688	4.80006	782.888	10.41450	5.67264
747.688	7.42385	1067.688	4.85799	802.888	10.28260	5.77933
767.688	7.55864	1087.688	4.91240	822.888	9.97222	6.01829

787.688	7.36253	1107.688	4.95414	842.888	9.94507	6.15658
807.688	7.28510	1127.688	4.75994	862.888	9.74337	6.22024
827.688	7.31260	1147.688	4.77757	882.888	9.51039	6.35284
847.688	7.21572	1167.688	4.76366	902.888	9.41564	6.39341
867.688	7.17180	1187.688	4.66623	922.888	9.33387	6.47580
887.688	7.15110	1207.688	4.64931	942.888	9.21896	6.54629
907.688	7.18045	1227.688	4.65184	962.888	9.13547	6.58603
927.688	7.09447	1247.688	4.52821	982.888	9.11558	6.80938
947.688	6.90667	1267.688	4.54175	1002.888	8.96560	6.74613
967.688	6.97940	1287.688	4.35201	1022.888	8.78540	6.46962
987.688	6.62049	1307.688	4.35738	1042.888	8.84252	6.40049
1007.688	7.02271	1327.688	4.38595	1062.888	8.69254	6.33517
1027.688	6.98649	1347.688	4.46249	1082.888	8.57691	6.27260
1047.688	6.94401	1367.688	4.36754	1102.888	8.60496	6.15779
1067.688	6.79053	1387.688	4.35356	1122.888	8.51684	5.99217
1087.688	6.71160	1407.688	4.31524	1142.888	8.32743	5.96607
1107.688	6.60196	1427.688	4.22633	1162.888	8.30535	5.90189
1127.688	6.58222	1447.688	4.16651	1182.888	8.25368	5.84152
1147.688	6.51163	1467.688	4.11459	1202.888	8.31043	5.69435
1167.688	6.57742	1487.688	4.11004	1222.888	8.33860	5.58081
1187.688	6.64029	1507.688	4.01918	1242.888	8.26738	5.56321
1207.688	6.65441	1527.688	4.01065	1262.888	8.16226	5.44136
1227.688	6.60167	1547.688	4.01987	1282.888	8.07069	5.42271
1247.688	6.45567	1567.688	3.97942	1302.888	8.04609	5.37516
1267.688	6.33059	1587.688	3.83324	1322.888	8.03774	5.32969
1287.688	6.38345	1607.688	3.79422	1342.888	8.11754	5.31845
1307.688	6.67175	1627.688	3.73113	1362.888	8.13360	5.28508
1327.688	6.54458	1647.688	3.76299	1382.888	8.05367	5.18926
1347.688	6.41787	1667.688	3.77489	1402.888	8.06348	5.20085
1367.688	6.50967	1687.688	3.73335	1422.888	7.97433	5.12787
1387.688	6.59738	1707.688	3.80148	1442.888	7.94549	5.12066
1407.688	6.39393	1727.688	3.76699	1462.888	7.54232	5.05606
1427.688	6.48581	1747.688	3.74560	1482.888	7.50105	4.91163
1447.688	6.57872	1767.688	3.71148	1502.888	7.81762	4.88376
1467.688	6.77521	1787.688	3.71604	1522.888	7.81958	4.86272
1487.688	6.65885	1807.688	3.68505	1542.888	7.72330	4.83788
1507.688	6.53134	1827.688	3.66833	1562.888	7.74499	4.79012
1527.688	6.44422	1847.688	3.59704	1582.888	7.72796	4.75786
1547.688	6.52318	1867.688	3.68109	1602.888	7.71411	4.71852
1567.688	6.57499	1887.688	3.63559	1622.888	7.68994	4.70494
1587.688	6.46647	1907.688	3.65144	1642.888	7.68600	4.68147
1607.688	6.64484	1927.688	3.64021	1662.888	7.65585	4.51762
1627.688	6.66161	1947.688	3.66803	1682.888	7.55785	4.50904
1647.688	6.65681	1967.688	3.66174	1702.888	7.52781	4.55033
1667.688	6.57072	1987.688	3.65242	1722.888	7.50194	4.50824
1687.688	6.58275	2007.688	3.61921	1742.888	7.55856	4.52828
1707.688	6.71615	2027.688	3.56504	1762.888	7.58300	4.51387
1727.688	6.65992	2047.688	3.56573	1782.888	7.52933	4.51815
1747.688	6.63580	2067.688	3.57321	1802.888	7.56128	4.52177
1767.688	6.66873	2087.688	3.54187	1822.888	7.53118	4.48257

1787.688	6.60470	2107.688	3.55021	1842.888	7.52869	4.44948
1807.688	6.69983	2127.688	3.54970	1862.888	5.78204	3.54036
1827.688	6.79417	2147.688	3.54397	1882.888	5.65763	3.51968
1847.688	6.58596	2167.688	3.58261	1902.888	7.34709	4.31202
1867.688	6.61907	2187.688	3.53343	1922.888	7.37760	4.26148
1887.688	6.63281	2207.688	3.42335	1942.888	7.46774	4.38204
1907.688	6.64121	2227.688	3.41360	1962.888	7.44332	4.37470
1927.688	6.64436	2247.688	3.50550	1982.888	7.44843	4.39101
1947.688	6.62387	2267.688	3.46906	2002.888	7.40341	4.36362
1967.688	6.55433	2287.688	3.49901	2022.888	7.40851	4.36758
1987.688	6.53943	2307.688	3.44169	2042.888	7.38907	4.36016
2007.688	6.63179	2327.688	3.44263	2062.888	7.41781	4.30453
2027.688	6.53512	2347.688	3.46113	2082.888	7.37882	4.29255
2047.688	6.52321	2367.688	3.44786	2102.888	7.39351	4.25945
2067.688	6.55411	2387.688	3.37668	2122.888	7.43242	4.24259
2087.688	6.60424	2407.688	3.28453	2142.888	7.45508	4.25355
2107.688	6.56041	2427.688	3.34168	2162.888	7.41448	4.23818
2127.688	6.48811	2447.688	3.32471	2182.888	7.44823	4.23847
2147.688	6.44647	2467.688	3.33997	2202.888	7.40024	4.23776
2167.688	6.37802	2487.688	3.35831	2222.888	7.41268	4.24139
2187.688	6.47063	2507.688	3.35108	2242.888	7.46445	4.21616
2207.688	6.42183	2527.688	3.34633	2262.888	7.46185	4.18708
2227.688	6.31755	2547.688	3.32415	2282.888	7.45835	4.17416
2247.688	6.55338	2567.688	3.29412	2302.888	7.47284	4.17512
2267.688	6.45496	2587.688	3.26434	2322.888	7.48154	4.16853
2287.688	6.38828	2607.688	3.23205	2342.888	7.49499	4.16409
2307.688	6.55554	2627.688	3.21407	2362.888	7.49718	4.17321
2327.688	6.51071	2647.688	3.25374			
2347.688	6.32955	2667.688	3.27080			
2367.688	6.41604	2687.688	3.28227			
2387.688	6.34447	2707.688	3.27011			
2407.688	6.30217	2727.688	3.25825			
2427.688	6.41353					
2447.688	6.36440					
2467.688	6.28882					
2487.688	6.40678					
2507.688	6.44135					
2527.688	6.50520					
2547.688	6.40214					
2567.688	6.39379					
2587.688	6.40666					
2607.688	6.35923					
2627.688	6.40705					
2647.688	6.33159					
2667.688	6.38077					
2687.688	6.27863					
2707.688	6.27956					
2727.688	6.38129					

E_{trans}(cm^{-1}) 00->02 E_{trans}(cm^{-1}) 02->00

370.00	2.16397	5.2	30.79500
390.00	4.01440	25.2	12.42550
410.00	4.06085	45.2	7.36703
430.00	4.55915	65.2	6.01360
450.00	6.01661	85.2	6.35558
470.00	3.93851	105.2	3.51920
490.00	4.91554	125.2	3.84762
510.00	4.59140	145.2	3.22536
530.00	3.35079	165.2	2.15002
550.00	3.87389	185.2	2.30091
570.00	3.40598	205.2	1.89221
590.00	3.09728	225.2	1.62291
610.00	4.66608	245.2	2.32162
630.00	3.16241	265.2	1.50250
650.00	4.29579	285.2	1.95811
670.00	4.27433	305.2	1.87667
690.00	3.77114	325.2	1.60030
710.00	4.26296	345.2	1.75359
730.00	4.46177	365.2	1.78373
750.00	4.22366	385.2	1.64473
770.00	4.67477	405.2	1.77669
790.00	3.89576	425.2	1.44762
810.00	4.26301	445.2	1.55123
830.00	4.01530	465.2	1.43280
850.00	4.06442	485.2	1.42405
870.00	3.94627	505.2	1.35917
890.00	3.65158	525.2	1.23759
910.00	4.03170	545.2	1.34587
930.00	3.65665	565.2	1.20335
950.00	4.09764	585.2	1.33040
970.00	3.31241	605.2	1.06181
990.00	3.79739	625.2	1.20263
1010.00	3.43220	645.2	1.07456
1030.00	3.10534	665.2	0.96167
1050.00	2.99161	685.2	0.91687
1070.00	2.71800	705.2	0.82480
1090.00	2.69917	725.2	0.81139
1110.00	2.90347	745.2	0.86496
1130.00	2.54197	765.2	0.75077
1150.00	2.84123	785.2	0.83225
1170.00	3.00185	805.2	0.87237
1190.00	2.30148	825.2	0.66378
1210.00	2.28648	845.2	0.65467
1230.00	2.40878	865.2	0.68488
1250.00	2.37195	885.2	0.66989
1270.00	2.68159	905.2	0.75246
1290.00	1.95836	925.2	0.54611
1310.00	2.07517	945.2	0.57522
1330.00	2.14494	965.2	0.59113
1350.00	2.10509	985.2	0.57691

1370.00	2.17015	1005.2	0.59155
1390.00	1.91791	1025.2	0.52007
1410.00	2.07881	1045.2	0.56087
1430.00	2.29781	1065.2	0.61695
1450.00	2.03719	1085.2	0.54440
1470.00	1.94719	1105.2	0.51798
1490.00	2.12451	1125.2	0.56266
1510.00	1.94219	1145.2	0.51217
1530.00	2.40459	1165.2	0.63148
1550.00	2.50855	1185.2	0.65613
1570.00	1.92757	1205.2	0.50220
1590.00	1.89657	1225.2	0.49225
1610.00	1.97468	1245.2	0.51064
1630.00	2.08778	1265.2	0.53795
1650.00	2.07163	1285.2	0.53193
1670.00	2.01428	1305.2	0.51545
1690.00	2.20022	1325.2	0.56118
1710.00	1.84978	1345.2	0.47028
1730.00	2.11065	1365.2	0.53493
1750.00	2.13052	1385.2	0.53832
1770.00	2.01112	1405.2	0.50664
1790.00	1.95274	1425.2	0.49052
1810.00	2.09775	1445.2	0.52546
1830.00	2.19892	1465.2	0.54928
1850.00	2.02909	1485.2	0.50550
1870.00	2.07191	1505.2	0.51481
1890.00	2.11996	1525.2	0.52540
1910.00	2.07162	1545.2	0.51214
1930.00	2.35723	1565.2	0.58133
1950.00	2.34736	1585.2	0.57751
1970.00	2.21945	1605.2	0.54477
1990.00	2.20764	1625.2	0.54064
2010.00	2.29267	1645.2	0.56021
2030.00	2.22731	1665.2	0.54305
2050.00	2.09960	1685.2	0.51082
2070.00	2.34196	1705.2	0.56860
2090.00	2.40388	1725.2	0.58244
2110.00	2.61088	1745.2	0.63133
2130.00	2.63129	1765.2	0.63502
2150.00	2.41371	1785.2	0.58139
2170.00	2.47633	1805.2	0.59535
2190.00	2.51463	1825.2	0.60345
2210.00	2.39199	1845.2	0.57298
2230.00	2.73145	1865.2	0.65313
2250.00	2.81669	1885.2	0.67235
2270.00	2.53128	1905.2	0.60319
2290.00	2.66983	1925.2	0.63515
2310.00	2.63355	1945.2	0.62549
2330.00	2.79687	1965.2	0.66321
2350.00	2.55256	1985.2	0.60432

2370.00	2.54624	2005.2	0.60189
2390.00	2.67866	2025.2	0.63223
2410.00	2.65163	2045.2	0.62492
2430.00	2.66418	2065.2	0.62696
2450.00	2.50726	2085.2	0.58918
2470.00	2.36640	2105.2	0.55529
2490.00	2.52054	2125.2	0.59064
2510.00	2.63933	2145.2	0.61763
2530.00	2.69596	2165.2	0.63004
2550.00	2.52665	2185.2	0.58969
2570.00	2.44575	2205.2	0.57007
2590.00	2.67197	2225.2	0.62200
2610.00	2.64388	2245.2	0.61469
2630.00	2.53832	2265.2	0.58942
2650.00	2.46484	2285.2	0.57166
2670.00	2.43132	2305.2	0.56322
2690.00	2.53015	2325.2	0.58542
2710.00	2.47275	2345.2	0.57148
2730.00	2.55290	2365.2	0.58933
2750.00	2.62352	2385.2	0.60495
2770.00	2.61678	2405.2	0.60273
2790.00	2.68153	2425.2	0.61698
2810.00	2.61891	2445.2	0.60192
2830.00	2.66459	2465.2	0.61178
2850.00	2.69268	2485.2	0.61759
2870.00	2.70991	2505.2	0.62090
2890.00	2.63858	2525.2	0.60395
2910.00	2.65006	2545.2	0.60598
2930.00	2.72424	2565.2	0.62233
2950.00	2.81685	2585.2	0.64287
2970.00	2.89130	2605.2	0.65923
2990.00	2.84887	2625.2	0.64895
3010.00	2.95891	2645.2	0.67340
3030.00	3.05481	2665.2	0.69459
3050.00	2.92549	2685.2	0.66459
3070.00	2.97028	2705.2	0.67417
3090.00	3.09557	2725.2	0.70199
3110.00	2.99669	2745.2	0.67898
3130.00	3.02503	2765.2	0.68482

Table S2: Computed excitation cross sections in Å^2 units for a series of inelastic processes generated using the 4D RR-PES for the $HeH^+(j_1)\cdot\cdot\cdot$ para-$H_2(j_2)$ system, with both Δj_1 = -1 and Δj_1 = -2.

E_{trans}(cm^{-1})	10->00	E_{trans}(cm^{-1})	20->10	20->00
0.008	7499.200	0.004	9660.380	4779.090
0.108	1147.690	0.104	1465.090	592.0150
0.208	1179.450	0.204	816.1810	283.5430
2.948	169.0020	8.844	148.3480	46.85420
22.948	46.19370	28.844	72.51200	20.49220

42.948	34.84030	48.844	58.00340	17.58820
62.948	24.24590	68.844	51.41510	15.85940
82.948	33.25430	88.844	44.58760	14.36650
102.948	25.37360	108.844	41.69940	15.84410
122.948	25.85570	128.844	37.51040	11.07130
142.948	17.15890	148.844	29.23080	10.33740
162.948	18.47370	168.844	35.99410	8.69570
182.948	14.66720	188.844	28.00320	8.95115
202.948	12.90200	208.844	26.66190	9.29074
222.948	12.83010	228.844	21.56610	8.13015
242.948	14.12460	248.844	21.70130	8.42868
262.948	12.02190	268.844	16.97310	6.52396
282.948	13.98630	288.844	16.21750	5.51196
302.948	14.09630	308.844	15.31580	6.17186
322.948	15.58310	328.844	15.36690	6.03793
342.948	10.09040	348.844	15.61140	5.24211
362.948	11.66320	368.844	13.61420	4.78832
382.948	11.66390	388.844	13.22250	4.78514
402.948	10.36490	408.844	13.27540	4.52172
422.948	8.96803	428.844	12.46430	4.17074
442.948	9.36195	448.844	11.52710	3.39711
462.948	10.24400	468.844	11.68990	3.88402
482.948	11.70960	488.844	9.63600	3.98077
502.948	11.16770	508.844	9.95699	3.76921
522.948	8.89507	528.844	9.51275	4.18260
542.948	9.28413	548.844	9.82762	3.61533
562.948	9.86964	568.844	10.32720	3.34452
582.948	11.30000	588.844	9.59630	3.15108
602.948	9.47288	608.844	9.34006	2.97434
622.948	7.39692	628.844	9.08012	2.84616
642.948	9.63695	648.844	10.01640	3.09030
662.948	8.96119	668.844	9.49883	3.24589
682.948	8.61349	688.844	9.80034	3.00986
702.948	8.68779	708.844	8.83335	2.66225
722.948	8.78907	728.844	8.55998	2.48490
742.948	9.05870	748.844	9.16413	2.23847
762.948	9.89314	768.844	9.36923	2.54048
782.948	8.23156	788.844	9.26417	2.46239
802.948	8.09192	808.844	9.49239	2.54618
822.948	7.46102	828.844	9.44376	2.58324
842.948	7.90818	848.844	9.56433	2.58299
862.948	8.77920	868.844	9.23858	2.42998
882.948	7.47604	888.844	8.25768	2.46156
902.948	7.67916	908.844	8.23508	2.33764
922.948	7.09752	928.844	7.87666	2.49894
942.948	7.89142	948.844	7.75481	2.50409
962.948	7.84554	968.844	8.81175	2.21927
982.948	7.98483	988.844	8.51847	2.33286
1002.948	7.62058	1008.844	8.85570	2.22272
1022.948	7.59219	1028.844	8.34853	2.29432

1042.948	7.49564	1048.844	8.36638	2.18115
1062.948	7.55377	1068.844	7.50072	2.42870
1082.948	7.74025	1088.844	7.80865	2.56339
1102.948	7.37381	1108.844	7.71429	2.55662
1122.948	6.56063	1128.844	8.03582	2.17211
1142.948	6.92309	1148.844	8.14871	2.26843
1162.948	7.19503	1168.844	8.52999	2.06600
1182.948	6.70201	1188.844	8.25205	2.13838
1202.948	6.57503	1208.844	7.87145	2.35065
1222.948	7.16405	1228.844	7.97983	2.20196
1242.948	6.89495	1248.844	7.46570	1.96689
1262.948	6.52201	1268.844	7.73955	2.13783
1282.948	6.55506	1288.844	7.73236	2.12133
1302.948	6.29245	1308.844	8.11202	2.32451
1322.948	6.70984	1328.844	8.01303	2.14657
1342.948	6.08883	1348.844	8.19732	1.93580
1362.948	6.47573	1368.844	8.20197	1.86552
1382.948	6.29744	1388.844	7.63538	2.09268
1402.948	6.70710	1408.844	7.26903	2.06303
1422.948	6.63183	1428.844	7.54319	1.99981
1442.948	6.63523	1448.844	7.50122	1.75912
1462.948	6.49839	1468.844	7.54954	1.78290
1482.948	6.41393	1488.844	7.75347	2.00079
1502.948	6.54795	1508.844	7.73314	1.97353
1522.948	6.64037	1528.844	7.62985	1.91289
1542.948	6.60242	1548.844	7.48976	1.95113
1562.948	6.54214	1568.844	7.38659	1.85262
1582.948	5.98661	1588.844	7.21815	1.92990
1602.948	6.67484	1608.844	7.28195	1.68642
1622.948	6.59172	1628.844	7.60163	1.64124
1642.948	6.64589	1648.844	7.22655	1.92020
1662.948	6.23172	1668.844	7.45814	1.91254
1682.948	6.28404	1688.844	7.27721	1.94396
1702.948	6.31909	1708.844	7.28219	1.98531
1722.948	6.40567	1728.844	7.06336	1.92928
1742.948	6.03209	1748.844	7.14855	1.74051
1762.948	6.33755	1768.844	6.81468	1.63442
1782.948	6.07433	1788.844	7.05770	1.54378
1802.948	6.04384	1808.844	6.98500	1.62214
1822.948	6.06531	1828.844	6.88066	1.74347
1842.948	5.77781	1848.844	7.09521	1.78424
1862.948	5.59910	1868.844	7.06113	1.89439
1882.948	5.67545	1888.844	6.96948	1.84816
1902.948	5.79115	1908.844	6.90259	1.85235
1922.948	5.96817	1928.844	6.75081	1.84081
1942.948	5.73023	1948.844	6.86019	1.74727
1962.948	5.67881	1968.844	6.77219	1.62417
1982.948	5.63732	1988.844	6.56835	1.71953
2002.948	5.88880	2008.844	6.84665	1.61059
2022.948	5.41613	2028.844	6.94786	1.67841

2042.948	5.36654	2048.844	6.88386	1.66174
2062.948	5.46429	2068.844	6.84083	1.54459
2082.948	5.40051	2088.844	6.78282	1.66556
2102.948	5.46585	2108.844	6.69901	1.78003
2122.948	5.36639	2128.844	6.72748	1.69943
2142.948	5.52634	2148.844	6.83881	1.66048
2162.948	5.52086	2168.844	6.65516	1.53961
2182.948	5.42310	2188.844	6.60119	1.54154
2202.948	5.54315	2208.844	6.71451	1.54717
2222.948	5.36736	2228.844	6.64497	1.44068
2242.948	5.17071	2248.844	6.70912	1.41013
2262.948	5.26609	2268.844	6.64437	1.33316
2282.948	5.18453	2288.844	6.61965	1.40203
2302.948	5.34809	2308.844	6.76034	1.53767
2322.948	5.23628	2328.844	6.64009	1.49584
2342.948	5.13325	2348.844	6.65759	1.39602
2362.948	5.19947	2368.844	6.39563	1.51109
2382.948	5.28422	2388.844	6.42372	1.33814
2402.948	5.19224	2408.844	6.50216	1.30182
2422.948	5.16651	2428.844	6.47976	1.34011
2442.948	5.39423	2448.844	6.41123	1.25374
2462.948	4.99124	2468.844	6.30700	1.25531
2482.948	5.04748	2488.844	6.33256	1.29848
2502.948	4.71867	2508.844	6.39336	1.36494
2522.948	4.92548	2528.844	6.36198	1.36603
2542.948	5.06172	2548.844	6.18676	1.31288
2562.948	4.75128	2568.844	6.11103	1.32575
2582.948	4.87531	2588.844	6.09896	1.31337
2602.948	5.10564	2608.844	6.15837	1.31288
2622.948	4.81037	2628.844	6.10503	1.32733
2642.948	4.98498	2648.844	6.09036	1.26936
2662.948	4.85029	2668.844	5.89584	1.26402
2682.948	4.78727	2688.844	5.95201	1.23373
2702.948	4.72294	2708.844	6.01761	1.25086
2722.948	4.59652	2728.844	6.04815	1.31207
2742.948	4.59874	2748.844	5.99350	1.30105
2762.948	4.55660	2768.844	5.98257	1.28329
2782.948	4.54570	2788.844	5.93142	1.26607
2802.948	4.44367	2808.844	5.90697	1.23713
2822.948	4.57755	2828.844	5.82907	1.26228
2842.948	4.66024	2848.844	5.71284	1.26775
2862.948	4.61590	2868.844	5.70672	1.26287
2882.948	4.59858	2888.844	5.59966	1.20689
2902.948	4.51403	2908.844	5.63372	1.18630
2922.948	4.56278	2928.844	5.76608	1.17948
2942.948	4.51635			
2962.948	4.44500			
2982.948	4.32437			
3002.948	4.30998			
3022.948	4.28012			

3042.948	4.24544
3062.948	4.36218

E_{trans}(cm^{-1})	30->20	30->10	E_{trans}(cm^{-1})	40->30	40->20
0.008	6558.850	5240.820	0.010	5321.900	2305.060
0.108	1543.930	1137.830	0.110	599.4310	254.9290
0.208	1045.080	742.8040	0.210	468.8450	164.7450
7.688	106.1050	49.35100	19.480	43.66070	21.27950
27.688	62.77070	28.56660	39.480	30.53700	17.40820
47.688	41.12080	21.01470	59.480	27.89630	14.37620
67.688	33.06090	17.66780	79.480	23.89410	12.81170
87.688	28.28420	14.47620	99.480	21.59860	11.53680
107.688	26.68730	14.96600	119.480	19.89280	10.42410
127.688	27.57130	12.49970	139.480	18.26500	9.13309
147.688	23.78050	11.34440	159.480	16.80210	9.57139
167.688	22.43840	9.16805	179.480	15.74280	8.86188
187.688	19.73000	9.12251	199.480	14.82790	7.97948
207.688	17.69960	10.28680	219.480	14.30210	7.78139
227.688	16.50270	8.65998	239.480	13.11800	7.70855
247.688	14.82080	7.19718	259.480	12.76620	7.82737
267.688	14.89720	6.22950	279.480	12.70810	6.99802
287.688	13.24580	7.24922	299.480	12.02530	7.05946
307.688	13.57950	7.92415	319.480	11.39250	6.70056
327.688	12.25580	6.59866	339.480	11.83370	6.28345
347.688	11.28070	6.42701	359.480	11.35480	6.14039
367.688	11.26200	5.93200	379.480	10.32740	5.55118
387.688	10.48920	5.72603	399.480	9.94175	5.70607
407.688	11.25150	4.95157	419.480	10.11850	5.45820
427.688	10.80570	4.88667	439.480	9.42122	5.37823
447.688	9.52069	4.50677	459.480	9.22136	5.39291
467.688	9.10942	4.33776	479.480	9.00398	5.29473
487.688	8.98626	4.26602	499.480	9.03578	4.98577
507.688	9.08919	4.40194	519.480	8.68297	4.95805
527.688	8.76098	4.24300	539.480	8.48319	4.93724
547.688	8.87575	4.31983	559.480	8.41413	4.93216
567.688	8.56140	4.42179	579.480	8.20980	4.73751
587.688	8.82482	3.98768	599.480	8.07370	4.60045
607.688	8.87013	4.08947	619.480	7.97006	4.48582
627.688	7.83404	4.12691	639.480	7.92715	4.40931
647.688	7.60888	3.45714	659.480	7.76204	4.44003
667.688	7.30707	3.80211	679.480	7.49226	4.22586
687.688	7.66623	3.72592	699.480	7.50990	4.27652
707.688	7.58586	3.70523	719.480	7.06882	4.32266
727.688	6.83987	3.55759	739.480	7.44320	4.22980
747.688	7.47705	3.60155	759.480	7.35291	4.10790
767.688	7.07094	3.47895	779.480	7.25927	4.09416
787.688	6.59552	3.49947	799.480	7.05336	4.01176
807.688	6.91992	3.35217	819.480	6.92863	4.00046
827.688	6.55955	3.42050	839.480	6.77541	4.02325
847.688	6.77178	3.48101	859.480	6.71709	3.94435

867.688	6.57664	3.70984	879.480	6.60911	3.94353
887.688	6.97382	3.45402	899.480	6.64120	3.87289
907.688	7.18433	3.53294	919.480	6.67118	3.80983
927.688	6.85209	3.16344	939.480	6.65323	3.96450
947.688	6.61668	3.11537	959.480	6.56994	3.77772
967.688	6.67968	3.16720	979.480	6.39598	3.75045
987.688	6.51844	3.08554	999.480	6.24507	3.65449
1007.688	6.78955	3.22088	1019.480	6.27109	3.66814
1027.688	6.71081	3.24832	1039.480	6.52804	3.73461
1047.688	6.95850	3.25875	1059.480	6.37882	3.64647
1067.688	6.68710	2.95177	1079.480	6.23191	3.66783
1087.688	6.77948	2.89825	1099.480	6.29817	3.58462
1107.688	6.52813	2.91097	1119.480	6.36067	3.58565
1127.688	6.37642	2.57325	1139.480	6.14361	3.53101
1147.688	6.63236	2.84145	1159.480	6.21140	3.45984
1167.688	6.63849	2.85940	1179.480	6.28031	3.56693
1187.688	6.61463	2.93514	1199.480	6.44791	3.45435
1207.688	6.53514	2.72146	1219.480	6.31818	3.37872
1227.688	6.59477	2.66345	1239.480	6.17916	3.41171
1247.688	6.47795	2.82110	1259.480	6.07952	3.31718
1267.688	6.42567	2.58936	1279.480	6.13712	3.33330
1287.688	6.43004	2.54418	1299.480	6.16937	3.38237
1307.688	6.45974	2.58215	1319.480	6.05181	3.44514
1327.688	6.53595	2.89765	1339.480	6.20305	3.32250
1347.688	6.65891	2.62933	1359.480	6.20345	3.36714
1367.688	6.58464	2.73104	1379.480	6.18416	3.21809
1387.688	6.44596	2.79477	1399.480	6.08999	3.20126
1407.688	6.54000	2.64456	1419.480	6.08732	3.19999
1427.688	6.63429	2.61205	1439.480	6.19696	3.28583
1447.688	6.29382	2.55529	1459.480	6.13185	3.20497
1467.688	6.40650	2.58318	1479.480	6.09683	3.26676
1487.688	6.53164	2.67179	1499.480	6.11454	3.30375
1507.688	6.56480	2.67883	1519.480	6.04374	3.17653
1527.688	6.43306	2.58076	1539.480	6.11883	3.14982
1547.688	6.58096	2.66138	1559.480	6.19318	3.07160
1567.688	6.48230	2.64382	1579.480	5.99224	3.03464
1587.688	6.44664	2.63210	1599.480	6.01143	3.00992
1607.688	6.40389	2.56691	1619.480	6.01323	3.11029
1627.688	6.33497	2.48570	1639.480	6.01041	3.10773
1647.688	6.53285	2.54754	1659.480	6.00307	3.00024
1667.688	6.44702	2.53006	1679.480	5.97465	3.04716
1687.688	6.52449	2.57604	1699.480	5.90233	3.07483
1707.688	6.36603	2.53723	1719.480	5.87959	2.98852
1727.688	6.43895	2.69442	1739.480	5.95337	2.98164
1747.688	6.55819	2.72778	1759.480	5.85768	2.91399
1767.688	6.45419	2.66053	1779.480	5.83832	2.88881
1787.688	6.36829	2.60333	1799.480	5.85743	2.90887
1807.688	6.49292	2.47316	1819.480	5.89381	2.86404
1827.688	6.51493	2.50026	1839.480	5.84653	2.82831
1847.688	6.55585	2.40441	1859.480	5.77418	2.75388

1867.688	6.55969	2.40814	1879.480	5.72942	2.86914
1887.688	6.56085	2.44237	1899.480	5.66114	2.86417
1907.688	6.37940	2.62711	1919.480	5.73593	2.84962
1927.688	6.56769	2.54829	1939.480	5.68547	2.80216
1947.688	6.54714	2.62369	1959.480	5.58622	2.88961
1967.688	6.52147	2.59016	1979.480	5.78769	2.79376
1987.688	6.37647	2.45261	1999.480	5.69397	2.75267
2007.688	6.48132	2.38564	2019.480	5.62855	2.78644
2027.688	6.41183	2.34887	2039.480	5.76928	2.67537
2047.688	6.50263	2.32624	2059.480	5.72336	2.63954
2067.688	6.57389	2.36541	2079.480	5.55794	2.66395
2087.688	6.45297	2.26313	2099.480	5.62776	2.76601
2107.688	6.42351	2.32963	2119.480	5.55903	2.76191
2127.688	6.44091	2.32497	2139.480	5.51617	2.64376
2147.688	6.47928	2.47024	2159.480	5.60785	2.65991
2167.688	6.32084	2.52723	2179.480	5.55925	2.68265
2187.688	6.38241	2.35879	2199.480	5.48776	2.64502
2207.688	6.39169	2.32613	2219.480	5.58522	2.63201
2227.688	6.41399	2.39132	2239.480	5.60994	2.60351
2247.688	6.40936	2.39175	2259.480	5.66019	2.55909
2267.688	6.34070	2.25202	2279.480	5.56533	2.57649
2287.688	6.32691	2.29851	2299.480	5.55298	2.60036
2307.688	6.36706	2.25139	2319.480	5.55915	2.61373
2327.688	6.36906	2.20739	2339.480	5.51311	2.59714
2347.688	6.31034	2.31222	2359.480	5.54972	2.53378
2367.688	6.29715	2.41731	2379.480	5.47965	2.57804
2387.688	6.25497	2.39889	2399.480	5.51755	2.55850
2407.688	6.11824	2.29202	2419.480	5.42472	2.53367
2427.688	6.26995	2.25428	2439.480	5.42108	2.51875
2447.688	6.22271	2.29309	2459.480	5.50447	2.47480
2467.688	6.14981	2.26815			
2487.688	6.16918	2.15292			
2507.688	6.16914	2.13111			
2527.688	6.19176	2.12546			
2547.688	6.17499	2.16120			
2567.688	6.17505	2.22069			
2587.688	6.15297	2.24688			
2607.688	6.10704	2.18247			
2627.688	5.95940	2.09688			
2647.688	5.93277	2.13825			
2667.688	6.00010	2.17255			
2687.688	5.85523	2.16141			
2707.688	5.90625	2.09254			
2727.688	5.87817	2.02756			

E_{trans}(cm^{-1})	50->30
4.220	64.4313
24.220	27.2621
44.220	20.4481
64.220	16.7207

84.220	15.7061
104.220	13.9698
124.220	13.7964
144.220	12.8315
164.220	11.3893
184.220	11.2000
204.220	10.9097
224.220	10.1784
244.220	9.32561
264.220	9.10885
284.220	9.07163
304.220	9.07446
324.220	8.28829
344.220	8.46853
364.220	8.20148
384.220	7.99424
404.220	7.83671
424.220	7.46884
444.220	7.20420
464.220	7.11022
484.220	7.02200
504.220	6.92582
524.220	6.51602
544.220	6.41154
564.220	6.27371
584.220	6.03667
604.220	5.91362
624.220	5.82211
644.220	5.58090
664.220	5.51605
684.220	5.21207
704.220	5.14904
724.220	5.11676
744.220	5.14245
764.220	4.97405
784.220	4.90235
804.220	4.80664
824.220	4.65864
844.220	4.54671
864.220	4.44674
884.220	4.40051
904.220	4.26462
924.220	4.21871
944.220	4.19303
964.220	4.11726
984.220	3.93500
1004.220	3.86546
1024.220	3.77332
1044.220	3.77852
1064.220	3.76438

1084.220	3.69810
1104.220	3.74120
1124.220	3.68395
1144.220	3.64067
1164.220	3.58610
1184.220	3.56981
1204.220	3.52019
1224.220	3.48511
1244.220	3.39924
1264.220	3.46069
1284.220	3.40072
1304.220	3.39880
1324.220	3.37216
1344.220	3.38211
1364.220	3.36097
1384.220	3.33756
1404.220	3.29291
1424.220	3.22993
1444.220	3.21725
1464.220	3.21101
1484.220	3.17033
1504.220	3.16558
1524.220	3.15324
1544.220	3.13659
1564.220	3.15939
1584.220	3.10507
1604.220	2.99799
1624.220	2.97939
1644.220	3.04952
1664.220	3.00808
1684.220	3.02446
1704.220	2.96571
1724.220	2.95752
1744.220	2.96458
1764.220	2.94461
1784.220	2.87557
1804.220	2.78926
1824.220	2.82999
1844.220	2.80803
1864.220	2.81346
1884.220	2.82157
1904.220	2.80832
1924.220	2.79733
1944.220	2.77196
1964.220	2.74029
1984.220	2.70909
2004.220	2.67605
2024.220	2.65508
2044.220	2.68181
2064.220	2.68991

2084.220	2.69349
2104.220	2.67778
2124.220	2.66247

Table S3: Computed excitation cross sections in Å^2 units for a series of inelastic processes generated using the 4D RR-PES for the $HeH^+(j_1)\cdot\cdot\cdot$ ortho-$H_2(j_2)$ system, with both $\Delta j_1 = +1$ and $\Delta j_1 = +2$.

E_{trans}(cm^{-1})	01->11	E_{trans}(cm^{-1})	11->21	E_{trans}(cm^{-1})	21->31
67.06	2.16738	134.11	0.49501	201.164	0.47172
68.40	17.77050	141.35	12.06590	207.244	6.19355
88.40	43.15000	161.35	22.76650	227.244	12.46110
108.40	49.78690	181.35	28.23310	247.244	15.75360
128.40	56.48200	201.35	29.93170	267.244	18.66820
148.40	52.44220	221.35	32.16310	287.244	19.46360
168.40	49.67840	241.35	32.05240	307.244	21.22320
188.40	54.79180	261.35	33.46880	327.244	21.09970
208.40	44.25730	281.35	33.21600	347.244	22.09920
228.40	40.74850	301.35	33.36490	367.244	22.16050
248.40	40.36860	321.35	32.46880	387.244	20.84930
268.40	37.80010	341.35	30.91580	407.244	21.83610
288.40	38.42740	361.35	28.62640	427.244	21.96690
308.40	36.96310	381.35	26.71340	447.244	21.90140
328.40	37.19320	401.35	26.13260	467.244	21.58260
348.40	32.90230	421.35	25.58480	487.244	19.24820
368.40	31.90790	441.35	24.76420	507.244	18.25350
388.40	36.29950	461.35	24.04760	547.244	16.89550
408.40	30.49710	481.35	23.76730	567.244	16.18160
428.40	26.88430	501.35	22.62700	587.244	16.18050
448.40	29.44200	521.35	22.48910	607.244	15.36050
468.40	29.55340	541.35	21.66350	627.244	14.20550
488.40	31.79590	561.35	21.30520	647.244	13.85480
508.40	30.78400	581.35	21.21930	667.244	13.97270
528.40	29.97380	601.35	20.49750	687.244	13.60970
548.40	29.07720	621.35	18.92980	707.244	12.61160
568.40	27.95410	641.35	18.76430	727.244	12.65550
588.40	26.96720	681.35	18.25530	747.244	12.23790
608.40	26.83970	701.35	17.85470	767.244	12.00440
628.40	27.82170	721.35	16.96580	787.244	12.06610
648.40	30.93070	741.35	16.89210	807.244	11.66520
668.40	29.28820	761.35	16.75800	827.244	11.27830
688.40	28.83260	781.35	16.78790	847.244	11.21660
708.40	27.18570	801.35	16.44610	867.244	10.99180
748.40	26.92690	821.35	16.39360	887.244	10.94030
768.40	26.12350	841.35	16.49060	907.244	10.44990
788.40	24.81260	861.35	16.31340	927.244	10.30830
808.40	25.87290	881.35	16.16990	947.244	9.92789
828.40	26.27990	901.35	15.91520	967.244	10.05520
848.40	25.69530	921.35	16.15280	987.244	9.97290
868.40	25.86780	941.35	15.87060	1007.244	9.78969

888.40	26.27400	961.35	15.25810	1027.244	9.70919
908.40	22.87400	981.35	15.11560	1047.244	9.63410
928.40	23.67140	1001.35	15.02000	1067.244	9.47951
948.40	23.17430	1021.35	14.55520	1087.244	9.53178
968.40	22.19210	1041.35	15.28310	1107.244	9.43470
988.40	22.64160	1061.35	14.45320	1127.244	9.35435
1008.40	22.71690	1081.35	14.55820	1147.244	9.10725
1028.40	23.22810	1101.35	14.35210	1167.244	9.24770
1048.40	22.97060	1121.35	14.05930	1227.244	9.12171
1068.40	23.02190	1141.35	14.19440	1247.244	9.23358
1088.40	22.40550	1161.35	13.72900	1267.244	9.39546
1108.40	21.49000	1181.35	14.03530	1287.244	9.42774
1128.40	21.74700	1201.35	13.89430	1307.244	9.18187
1148.40	21.33690	1221.35	13.61570	1327.244	9.13891
1168.40	21.50650	1241.35	13.90870	1347.244	9.11492
1188.40	21.18810	1261.35	13.92290	1367.244	9.23190
1208.40	21.39940	1281.35	13.67090	1387.244	9.22191
1228.40	20.43720	1301.35	13.34320	1407.244	9.10981
1248.40	20.88040	1361.35	13.83670	1427.244	9.20316
1268.40	20.49380	1381.35	13.43570	1447.244	9.12994
1288.40	20.08110	1401.35	13.45440	1467.244	8.96239
1308.40	20.30560	1421.35	13.38900	1487.244	8.95612
1328.40	19.30010	1441.35	13.23490	1507.244	9.22981
1348.40	19.27970	1461.35	13.22540	1527.244	9.19918
1368.40	19.44270	1481.35	13.30940	1547.244	9.14384
1428.40	19.93030	1501.35	13.36250	1567.244	9.12303
1448.40	19.16460	1521.35	13.32230	1587.244	9.09013
1468.40	18.85720	1541.35	13.10540	1607.244	8.93352
1488.40	19.23340	1561.35	12.85880	1627.244	9.03517
1508.40	19.24330	1581.35	12.85540	1647.244	9.01634
1528.40	19.08830	1601.35	12.91640	1667.244	8.95722
1548.40	19.71380	1621.35	12.89920	1687.244	9.19498
1568.40	19.87750	1641.35	13.10810	1707.244	9.13043
1588.40	19.41450	1661.35	12.92020	1727.244	9.03442
1608.40	20.31260	1681.35	12.95780	1747.244	9.12850
1628.40	19.36560	1701.35	12.58050	1767.244	9.04920
1648.40	19.43950	1721.35	12.50840	1787.244	8.90378
1668.40	19.27700	1741.35	12.47860	1807.244	8.93463
1688.40	19.65080	1761.35	12.39690	1827.244	9.06686
1708.40	19.99870	1781.35	12.63740	1847.244	8.95484
1728.40	19.78640	1801.35	12.44110	1867.244	8.94986
1748.40	19.08780	1821.35	12.57990	1887.244	9.06750
1768.40	19.10780	1841.35	12.56540	1907.244	9.16036
1788.40	19.80550	1861.35	12.34340	1927.244	9.08348
1808.40	19.25630	1881.35	12.18260	1947.244	9.07431
1828.40	18.65800	1901.35	12.08640	1967.244	9.04858
1848.40	18.45660	1921.35	11.91970	1987.244	9.03014
1868.40	18.47500	1941.35	11.98660	2007.244	9.17262
1888.40	18.75250	1961.35	12.17720	2027.244	9.15563
1908.40	18.38990	1981.35	11.99680	2047.244	9.10508

1928.40	18.13290	2001.35	12.00610	2067.244	9.16838
1948.40	17.85140	2021.35	12.01820	2087.244	9.17866
1968.40	17.93930	2041.35	12.19260	2107.244	9.20194
1988.40	17.66600	2061.35	11.80300	2127.244	9.29718
2008.40	17.57400	2081.35	11.62190	2147.244	9.16386
2028.40	17.94400	2101.35	11.64580	2167.244	9.15005
2048.40	17.24160	2121.35	11.69420	2187.244	9.07940
2068.40	16.82010	2141.35	11.55950	2207.244	9.07539
2088.40	17.20100	2161.35	11.72120	2227.244	9.10783
2108.40	17.61800	2181.35	11.80490	2247.244	9.16311
2128.40	17.03180	2201.35	11.57450	2267.244	9.15193
2148.40	16.83780	2221.35	11.68920	2287.244	9.14140
2168.40	17.05610	2241.35	11.65600	2307.244	9.12541
2188.40	17.16700	2261.35	11.53920	2327.244	9.15064
2208.40	16.95750	2281.35	11.38090	2347.244	9.15238
2228.40	17.37970	2301.35	11.38470	2367.244	9.03448
2248.40	16.74610	2321.35	11.39790	2387.244	8.99211
2268.40	16.83590	2341.35	11.30870	2407.244	9.01430
2288.40	16.67490	2361.35	11.42140	2427.244	9.00369
2308.40	16.44280	2381.35	11.49190	2447.244	9.01915
2328.40	16.97000	2401.35	11.48400	2467.244	9.08012
2348.40	16.60290	2421.35	11.30970	2487.244	9.08133
2368.40	16.48210	2441.35	11.31590		
2388.40	16.38310	2461.35	11.18280		
2408.40	16.19770	2481.35	11.26220		
2428.40	16.29740	2501.35	11.14960		
2448.40	16.52480	2521.35	11.09450		
2468.40	16.27260	2541.35	10.97690		
2488.40	16.18680	2561.35	11.07570		
2508.40	16.31360	2581.35	11.05830		
2528.40	16.22800	2601.35	11.06130		
2548.40	16.41100	2621.35	10.96880		
2568.40	15.86260				
2588.40	15.77510				
2608.40	15.81840				
2628.40	15.96510				
2648.40	15.76750				
2668.40	15.84940				
2688.40	15.49370				

E_{trans}(cm^{-1})	31->41	E_{trans}(cm^{-1})	01->21	E_{trans}(cm^{-1})	11->31
268.288	0.58219	201.160	0.51446	335.268	0.19294
286.088	5.91297	208.400	7.26338	341.348	3.35536
306.088	8.40361	228.400	14.17630	361.348	6.98569
346.088	11.08540	248.400	19.51100	381.348	9.38851
366.088	11.99450	268.400	22.14980	401.348	10.60850
386.088	13.08070	288.400	25.04680	421.348	12.62490
406.088	13.48230	308.400	24.00830	441.348	12.92010
426.088	12.86730	328.400	23.13790	461.348	13.43520
446.088	13.02030	348.400	24.56090	481.348	13.65820

466.088	12.76560	368.400	25.47280	501.348	15.35570
486.088	12.75880	388.400	25.62920	521.348	14.11170
506.088	12.73120	408.400	25.07320	541.348	14.92320
526.088	12.80240	428.400	25.15540	561.348	15.21510
546.088	12.53910	448.400	23.89080	581.348	15.24660
566.088	12.59740	468.400	22.60650	601.348	15.29830
586.088	12.52850	488.400	22.03130	621.348	14.43190
606.088	12.32710	508.400	22.27850	641.348	13.89030
626.088	11.63280	528.400	20.42330	681.348	12.69060
646.088	11.26240	548.400	20.01100	701.348	12.31090
666.088	10.99600	568.400	19.87370	721.348	11.66250
686.088	10.83650	588.400	21.00950	741.348	11.33200
706.088	10.26270	608.400	19.85350	761.348	10.70650
726.088	10.24140	628.400	19.58420	781.348	10.41950
746.088	10.04180	648.400	17.61910	801.348	10.10310
766.088	9.85518	668.400	17.67780	821.348	9.71036
786.088	9.81814	688.400	17.56130	841.348	9.77379
806.088	9.88934	708.400	16.96020	861.348	9.47262
826.088	9.56635	748.400	17.03070	881.348	9.22070
846.088	9.54975	768.400	16.86960	901.348	9.60957
866.088	9.44798	788.400	15.65200	921.348	9.36942
886.088	9.10454	808.400	14.93900	941.348	8.72940
906.088	9.24244	828.400	14.17550	961.348	8.95370
926.088	9.03701	848.400	14.32840	981.348	8.34170
946.088	8.73887	868.400	14.44060	1001.348	8.18254
966.088	8.69932	888.400	12.93520	1021.348	8.17883
1026.088	8.47686	908.400	15.20780	1041.348	8.10255
1046.088	8.42334	928.400	14.14720	1061.348	8.00053
1066.088	8.32634	948.400	14.63400	1081.348	7.76042
1086.088	8.25157	968.400	13.68010	1101.348	7.72647
1106.088	8.29533	988.400	14.02090	1121.348	7.70889
1126.088	8.06852	1008.400	13.63710	1141.348	7.71058
1146.088	8.06738	1028.400	13.37980	1161.348	7.65789
1166.088	8.07742	1048.400	12.84570	1181.348	7.72495
1186.088	8.02212	1068.400	12.80480	1201.348	7.66552
1206.088	7.95371	1088.400	12.60460	1221.348	7.51356
1226.088	7.93440	1108.400	12.80690	1241.348	7.18151
1246.088	7.85687	1128.400	13.19610	1261.348	7.45270
1266.088	8.00469	1148.400	12.44070	1281.348	7.48585
1286.088	7.91807	1168.400	12.09730	1301.348	7.17657
1306.088	7.88314	1188.400	12.16360	1361.348	6.90908
1326.088	7.91680	1208.400	12.04490	1381.348	6.67753
1346.088	7.86589	1228.400	12.52110	1401.348	6.48623
1366.088	7.85813	1248.400	12.33130	1421.348	6.37720
1386.088	7.85369	1268.400	12.32510	1441.348	6.48926
1406.088	7.85252	1288.400	12.53410	1461.348	6.53798
1426.088	7.83558	1308.400	11.91810	1481.348	6.22805
1446.088	7.74500	1328.400	11.64440	1501.348	6.27433
1466.088	7.78196	1348.400	11.60260	1521.348	6.19087
1486.088	7.78992	1368.400	11.45790	1541.348	6.31974

1506.088	7.69778	1428.400	11.24780	1561.348	6.15705
1526.088	7.70539	1448.400	11.22750	1581.348	6.08495
1546.088	7.68322	1468.400	10.70750	1601.348	6.11179
1566.088	7.64336	1488.400	11.03280	1621.348	6.12518
1586.088	7.62434	1508.400	10.51930	1641.348	6.06346
1606.088	7.54039	1528.400	10.33530	1661.348	6.04042
1626.088	7.54590	1548.400	10.18240	1681.348	6.09841
1646.088	7.62708	1568.400	9.41966	1701.348	6.18372
1666.088	7.59601	1588.400	9.95142	1721.348	5.90393
1686.088	7.58546	1608.400	9.82289	1741.348	5.96798
1706.088	7.59535	1628.400	9.91467	1761.348	6.01698
1726.088	7.58879	1648.400	10.00950	1781.348	5.98671
1746.088	7.50451	1668.400	9.79344	1801.348	5.99250
1766.088	7.51954	1688.400	9.64876	1821.348	5.79416
1786.088	7.53463	1708.400	9.34976	1841.348	5.71614
1806.088	7.46013	1728.400	9.72534	1861.348	5.90876
1826.088	7.47589	1748.400	9.87229	1881.348	5.86416
1846.088	7.46572	1768.400	9.28239	1901.348	5.71006
1866.088	7.44357	1788.400	8.75186	1921.348	5.74061
1886.088	7.41284	1808.400	8.95219	1941.348	5.76180
1906.088	7.35227	1828.400	9.09732	1961.348	5.79691
1926.088	7.42045	1848.400	8.92199	1981.348	5.80677
1946.088	7.32863	1868.400	9.07856	2001.348	5.67296
1966.088	7.37206	1888.400	8.74962	2021.348	5.57323
1986.088	7.34664	1908.400	9.04252	2041.348	5.73816
2006.088	7.29495	1928.400	9.06181	2061.348	5.84483
2026.088	7.29835	1948.400	8.55589	2081.348	5.80757
2046.088	7.22863	1968.400	8.28935	2101.348	5.74841
2066.088	7.24287	1988.400	8.66320	2121.348	5.71487
2086.088	7.22926	2008.400	8.50913	2141.348	5.73868
2106.088	7.15685	2028.400	8.46147	2161.348	5.73928
2126.088	7.23087	2048.400	8.63979	2181.348	5.63315
2146.088	7.25503	2068.400	8.90987	2201.348	5.67790
2166.088	7.18400	2088.400	8.56221	2221.348	5.62210
2186.088	7.20566	2108.400	8.60736	2241.348	5.73195
2206.088	7.21080	2128.400	8.47471	2261.348	5.69469
2226.088	7.16178	2148.400	8.56861	2281.348	5.72423
2246.088	7.14798	2168.400	8.47537	2301.348	5.72998
2266.088	7.20219	2188.400	8.40527	2321.348	5.76835
2286.088	7.16360	2208.400	8.39055	2341.348	5.73534
		2228.400	8.43632	2361.348	5.53571
		2248.400	8.65016	2381.348	5.60175
		2268.400	8.16600	2401.348	5.54783
		2288.400	8.22846	2421.348	5.58155
		2308.400	8.11080	2441.348	5.60872
		2328.400	7.95501	2461.348	5.66413
		2348.400	7.98773	2481.348	5.61760
		2368.400	7.78834	2501.348	5.70875
		2388.400	7.91027	2521.348	5.61844
		2408.400	7.79761	2541.348	5.55417

		2428.400	7.37246	2561.348	5.52821
		2448.400	7.52648	2581.348	5.47467
		2468.400	7.72029	2601.348	5.53148
		2488.400	7.43052	2621.348	5.53886
		2508.400	7.30299		
		2528.400	7.15640		
		2548.400	7.16695		
		2568.400	7.28321		
		2588.400	7.20560		
		2608.400	6.98811		
		2628.400	6.90549		
		2648.400	6.81555		
		2668.400	7.00716		
		2688.400	7.20423		

E_{trans}(cm^{-1})	21->41	E_{trans}(cm^{-1})	31->51	E_{trans}(cm^{-1})	01->03
469.444	0.23525	606.088	0.78820	608.4	0.16215
487.244	2.68610	626.088	1.99772	628.4	1.30252
507.244	4.03424	646.088	2.68928	648.4	1.77691
547.244	5.65834	666.088	3.37126	668.4	2.26961
567.244	6.25620	686.088	3.86672	688.4	2.38152
587.244	6.81192	706.088	4.28866	708.4	2.43786
607.244	7.37919	726.088	4.54426	748.4	2.47067
627.244	7.21891	746.088	4.79758	768.4	2.43974
647.244	7.38593	766.088	4.99901	788.4	2.64364
667.244	7.29622	786.088	5.20861	808.4	2.65202
687.244	7.29477	806.088	5.26166	828.4	2.49788
707.244	7.73829	826.088	5.47106	848.4	2.28524
727.244	7.42015	846.088	5.75813	868.4	2.51333
747.244	7.50512	866.088	5.77922	888.4	2.66038
767.244	7.83405	886.088	5.88677	908.4	2.55513
787.244	7.50773	906.088	6.02173	928.4	2.43494
807.244	7.52471	926.088	6.14883	948.4	2.67197
827.244	7.23957	946.088	6.24620	968.4	2.54148
847.244	6.94609	966.088	6.32489	988.4	2.65743
867.244	7.04388	1026.088	6.35495	1008.4	2.64070
887.244	6.99219	1046.088	6.21331	1028.4	2.74694
907.244	6.76074	1066.088	6.12407	1048.4	2.93362
927.244	6.67899	1086.088	6.01070	1068.4	2.65517
947.244	6.57588	1106.088	5.97803	1088.4	2.49887
967.244	6.44047	1126.088	5.97089	1108.4	2.55295
987.244	6.47098	1146.088	5.85342	1128.4	2.53157
1007.244	6.50241	1166.088	5.80086	1148.4	2.58915
1027.244	6.30384	1186.088	5.73887	1168.4	2.31447
1047.244	6.29212	1206.088	5.65328	1188.4	2.32918
1067.244	6.35244	1226.088	5.59002	1208.4	2.21568
1087.244	6.15159	1246.088	5.45609	1228.4	2.09735

1107.244	6.08868	1266.088	5.44110	1248.4	1.99508
1127.244	6.01164	1286.088	5.35080	1268.4	2.01143
1147.244	6.07175	1306.088	5.30353	1288.4	2.04961
1167.244	6.06937	1326.088	5.26993	1308.4	1.81020
1227.244	5.81375	1346.088	5.30657	1328.4	1.95181
1247.244	5.91841	1366.088	5.19237	1348.4	1.69884
1267.244	5.76537	1386.088	5.18489	1368.4	1.73903
1287.244	5.76781	1406.088	5.14038	1428.4	1.58812
1307.244	5.78394	1426.088	5.03910	1448.4	1.60551
1327.244	5.70832	1446.088	4.98868	1468.4	1.68473
1347.244	5.65705	1466.088	4.97654	1488.4	1.56886
1367.244	5.57409	1486.088	4.89038	1508.4	1.60498
1387.244	5.57906	1506.088	4.82071	1528.4	1.51947
1407.244	5.65078	1526.088	4.71277	1548.4	1.51765
1427.244	5.64391	1546.088	4.66953	1568.4	1.57557
1447.244	5.56903	1566.088	4.67662	1588.4	1.66664
1467.244	5.54823	1586.088	4.59580	1608.4	1.62461
1487.244	5.44055	1606.088	4.53608	1628.4	1.62899
1507.244	5.46314	1626.088	4.50038	1648.4	1.61042
1527.244	5.31948	1646.088	4.49977	1668.4	1.56526
1547.244	5.31480	1666.088	4.45189	1688.4	1.58560
1567.244	5.42871	1686.088	4.41867	1708.4	1.57488
1587.244	5.42217	1706.088	4.40145	1728.4	1.58977
1607.244	5.45062	1726.088	4.43271	1748.4	1.67891
1627.244	5.33323	1746.088	4.39362	1768.4	1.61656
1647.244	5.28773	1766.088	4.37202	1788.4	1.64159
1667.244	5.25465	1786.088	4.31408	1808.4	1.62830
1687.244	5.15017	1806.088	4.30664	1828.4	1.54321
1707.244	5.22227	1826.088	4.28100	1848.4	1.45382
1727.244	5.17042	1846.088	4.27673	1868.4	1.47063
1747.244	5.17617	1866.088	4.25991	1888.4	1.51804
1767.244	5.18437	1886.088	4.30752	1908.4	1.50691
1787.244	5.22196	1906.088	4.25092	1928.4	1.50267
1807.244	5.08597	1926.088	4.23887	1948.4	1.44018
1827.244	5.13569	1946.088	4.25271	1968.4	1.44755
1847.244	5.07387	1966.088	4.23716	1988.4	1.42341
1867.244	5.12194	1986.088	4.19240	2008.4	1.52434
1887.244	5.02940	2006.088	4.19117	2028.4	1.36551
1907.244	4.98783	2026.088	4.15999	2048.4	1.39763
1927.244	5.03788	2046.088	4.14729	2068.4	1.46090
1947.244	5.01302	2066.088	4.19402	2088.4	1.42125
1967.244	5.02660	2086.088	4.16824	2108.4	1.36703
1987.244	4.92803	2106.088	4.11001	2128.4	1.30862
2007.244	4.91393	2126.088	4.11192	2148.4	1.33970
2027.244	4.91951	2146.088	4.09629	2168.4	1.36735
2047.244	4.92795	2166.088	4.06316	2188.4	1.32082
2067.244	4.89380	2186.088	4.07906	2208.4	1.33407
2087.244	4.77870	2206.088	4.04686	2228.4	1.26452
2107.244	4.75376	2226.088	4.05011	2248.4	1.39209
2127.244	4.85604	2246.088	4.05419	2268.4	1.35775

2147.244	4.79982	2266.088	4.05475	2288.4	1.30889
2167.244	4.82396	2286.088	4.02751	2308.4	1.38258
2187.244	4.79475			2328.4	1.38303
2207.244	4.78225			2348.4	1.34782
2227.244	4.75807			2368.4	1.31209
2247.244	4.76787			2388.4	1.36777
2267.244	4.69431			2408.4	1.32221
2287.244	4.72880			2428.4	1.36898
2307.244	4.72593			2448.4	1.33977
2327.244	4.70338			2468.4	1.34818
2347.244	4.72759			2488.4	1.32001
2367.244	4.70823			2508.4	1.39633
2387.244	4.71319			2528.4	1.34555
2407.244	4.67878			2548.4	1.32043
2427.244	4.67085			2568.4	1.32138
2447.244	4.67140			2588.4	1.32629
2467.244	4.68121			2608.4	1.33247
2487.244	4.68864			2628.4	1.31618
				2648.4	1.35824
				2668.4	1.39290
				2688.4	1.35091

Table S4: Computed de-excitation cross sections in Å^2 units for a series of inelastic processes generated using the 4D RR-PES for the $HeH^+(j_1)\cdot\cdot\cdot$ ortho-$H_2(j_2)$ system, with both Δj_1 = -1 and Δj_1 = -2.

E_{trans}(cm^{-1})	11->01	E_{trans}(cm^{-1})	21->11	21->01
0.008	6056.010	0.004	9957.640	5174.45
1.348	300.5690	7.244	141.2610	41.7915
21.348	59.56000	27.244	80.89860	23.7694
41.348	43.50800	47.244	65.02430	20.5170
61.348	39.40520	67.244	53.77460	17.6819
81.348	31.88940	87.244	48.96080	16.5593
101.348	27.51520	107.244	43.27950	13.8081
121.348	28.35580	127.244	41.24520	11.9432
141.348	21.75060	147.244	38.08070	11.6229
161.348	19.22750	167.244	36.07110	11.2221
181.348	18.43150	187.244	33.43370	10.6325
201.348	16.79610	207.244	30.55250	9.88197
221.348	16.68930	227.244	27.31190	9.48458
241.348	15.74410	247.244	24.72160	8.66562
261.348	15.57850	267.244	23.54760	7.92449
281.348	13.58120	287.244	22.51770	7.49196
301.348	13.00250	307.244	21.34390	7.37289
321.348	14.62460	327.244	20.34140	6.59548
341.348	12.16260	347.244	19.76770	6.32066
361.348	10.62430	367.244	18.53370	6.15188
381.348	11.53960	387.244	18.16630	6.38460
401.348	11.49690	407.244	17.27830	5.93200
421.348	12.28530	427.244	16.79550	5.76097

441.348	11.82030	447.244	16.54910	5.10872
461.348	11.44340	467.244	15.82830	5.05769
481.348	11.04260	487.244	14.48390	4.96227
501.348	10.56420	507.244	14.23510	4.73722
521.348	10.14520	547.244	13.63730	4.65818
541.348	10.05470	567.244	13.24550	4.57038
561.348	10.38170	587.244	12.50410	4.20270
581.348	11.49940	607.244	12.37360	3.97754
601.348	10.85130	627.244	12.20450	3.74431
621.348	10.64800	647.244	12.15970	3.75631
641.348	10.00930	667.244	11.85090	3.75880
681.348	9.85894	687.244	11.75550	3.34426
701.348	9.54033	707.244	11.77050	3.90664
721.348	9.03969	727.244	11.59300	3.61207
741.348	9.40434	747.244	11.44310	3.71468
761.348	9.53146	767.244	11.21820	3.45336
781.348	9.30011	787.244	11.34260	3.52070
801.348	9.34409	807.244	11.10430	3.40706
821.348	9.47299	827.244	10.63890	3.32666
841.348	8.23231	847.244	10.50490	3.17912
861.348	8.50471	867.244	10.40550	3.15498
881.348	8.31245	887.244	10.05310	3.09247
901.348	7.94768	907.244	10.52530	3.12930
921.348	8.09647	927.244	9.92608	3.21177
941.348	8.11168	947.244	9.97152	3.01651
961.348	8.28273	967.244	9.80520	2.92263
981.348	8.18002	987.244	9.58143	2.92841
1001.348	8.18782	1007.244	9.65054	2.89007
1021.348	7.95882	1027.244	9.31278	2.99460
1041.348	7.62459	1047.244	9.49954	2.93999
1061.348	7.70697	1067.244	9.38413	2.92963
1081.348	7.55333	1087.244	9.17707	2.97063
1101.348	7.60528	1107.244	9.35597	2.81667
1121.348	7.48503	1127.244	9.34757	2.74448
1141.348	7.55219	1147.244	9.16138	2.72739
1161.348	7.20571	1167.244	8.92573	2.68650
1181.348	7.35519	1227.244	9.20919	2.61828
1201.348	7.21255	1247.244	8.92817	2.60766
1221.348	7.06120	1267.244	8.92689	2.48144
1241.348	7.13414	1287.244	8.87034	2.55137
1261.348	6.77536	1307.244	8.75556	2.42760
1281.348	6.76287	1327.244	8.73702	2.38035
1301.348	6.81482	1347.244	8.78056	2.34055
1361.348	6.97065	1367.244	8.80388	2.16111
1381.348	6.69829	1387.244	8.76610	2.27888
1401.348	6.58649	1407.244	8.61257	2.24540
1421.348	6.71357	1427.244	8.44023	2.26241
1441.348	6.71285	1447.244	8.42796	2.28015
1461.348	6.65471	1467.244	8.45816	2.22722
1481.348	6.86871	1487.244	8.43736	2.19076

1501.348	6.92174	1507.244	8.56459	2.11952
1521.348	6.75674	1527.244	8.43280	2.20126
1541.348	7.06541	1547.244	8.44854	2.23116
1561.348	6.73243	1567.244	8.19419	2.09476
1581.348	6.75460	1587.244	8.13911	1.97220
1601.348	6.69473	1607.244	8.11186	2.01452
1621.348	6.82116	1627.244	8.05112	2.04438
1641.348	6.93855	1647.244	8.19975	2.00230
1661.348	6.86165	1667.244	8.06506	2.03478
1681.348	6.61636	1687.244	8.14786	1.95855
1701.348	6.62029	1707.244	8.13147	2.02159
1721.348	6.85899	1727.244	7.98102	2.02343
1741.348	6.66591	1747.244	7.87059	1.90818
1761.348	6.45608	1767.244	7.80215	1.84658
1781.348	6.38376	1787.244	7.68845	1.92765
1801.348	6.38756	1807.244	7.72562	1.89125
1821.348	6.48095	1827.244	7.84251	1.87859
1841.348	6.35319	1847.244	7.72063	1.91612
1861.348	6.26203	1867.244	7.72105	1.97394
1881.348	6.16253	1887.244	7.72333	1.89497
1901.348	6.19065	1907.244	7.82997	1.90303
1921.348	6.09416	1927.244	7.57458	1.87185
1941.348	6.06032	1947.244	7.45337	1.89075
1961.348	6.18583	1967.244	7.46379	1.86840
1981.348	5.94169	1987.244	7.49004	1.85122
2001.348	5.79456	2007.244	7.39906	1.84628
2021.348	5.92386	2027.244	7.49795	1.85469
2041.348	6.06556	2047.244	7.54688	1.90002
2061.348	5.86194	2067.244	7.39524	1.79212
2081.348	5.79342	2087.244	7.46414	1.80429
2101.348	5.86679	2107.244	7.43866	1.77701
2121.348	5.90322	2127.244	7.35999	1.74145
2141.348	5.82950	2147.244	7.25502	1.74721
2161.348	5.97295	2167.244	7.25348	1.70225
2181.348	5.75362	2187.244	7.25807	1.72755
2201.348	5.78290	2207.244	7.19749	1.70165
2221.348	5.72608	2227.244	7.26548	1.60766
2241.348	5.64488	2247.244	7.30659	1.64004
2261.348	5.82440	2267.244	7.29793	1.68105
2281.348	5.69696	2287.244	7.18368	1.61680
2301.348	5.65411	2307.244	7.18416	1.58794
2321.348	5.61877	2327.244	7.09631	1.55499
2341.348	5.55386	2347.244	7.14337	1.55623
2361.348	5.58671	2367.244	7.06876	1.58042
2381.348	5.66337	2387.244	7.03063	1.56255
2401.348	5.57566	2407.244	6.95305	1.51441
2421.348	5.54501	2427.244	7.01255	1.49555
2441.348	5.58723	2447.244	6.99855	1.47515
2461.348	5.55669	2467.244	6.99755	1.51569
2481.348	5.61816	2487.244	6.93613	1.55738

2501.348	5.42927
2521.348	5.39822
2541.348	5.41191
2561.348	5.46102
2581.348	5.39235
2601.348	5.41931
2621.348	5.29666

E_{trans}(cm^{-1})	31->21	31->11	E_{trans}(cm^{-1})	41->31	41->21
0.008	8472.590	3465.330	0.08	1518.550	766.9300
6.088	150.5980	80.62790	17.88	73.58570	40.66580
26.088	77.53180	41.46840	37.88	52.81510	30.01210
46.088	60.36570	33.29300	77.88	38.31490	22.08880
66.088	53.92110	27.61070	97.88	34.89230	20.14250
86.088	46.38770	26.48190	117.88	33.32200	18.85280
106.088	43.90360	23.03590	137.88	30.88430	18.05500
126.088	39.11520	21.06790	157.88	27.00940	15.93340
146.088	37.52060	19.28690	177.88	25.39620	14.93050
166.088	35.00010	19.86520	197.88	23.38630	13.66810
186.088	30.99060	16.94380	217.88	22.13930	12.78300
206.088	30.82110	16.80000	237.88	21.06640	12.78160
226.088	29.65100	16.19020	257.88	20.31370	11.62530
246.088	28.43140	15.43620	277.88	19.16580	11.21220
266.088	27.07040	14.81720	297.88	18.62000	11.21000
286.088	23.41580	13.43330	317.88	17.96610	10.32960
306.088	21.60670	12.47330	337.88	17.19850	9.98757
346.088	19.08260	10.70750	357.88	15.82840	9.29686
366.088	17.90930	10.10790	377.88	14.97690	8.65212
386.088	17.57900	9.33839	397.88	14.31760	8.52960
406.088	16.40660	8.86609	417.88	13.83790	8.24769
426.088	14.93710	8.19885	437.88	12.87130	7.78199
446.088	14.35890	7.82154	457.88	12.63150	7.51416
466.088	14.28800	7.44440	477.88	12.19380	7.24143
486.088	13.74410	7.03187	497.88	11.79440	6.95115
506.088	12.58880	6.96363	517.88	11.59120	6.85319
526.088	12.49610	6.64681	537.88	11.52710	6.76474
546.088	11.96130	6.37781	557.88	11.01760	6.44860
566.088	11.62140	6.55746	577.88	10.87490	6.33483
586.088	11.57670	6.31244	597.88	10.64490	6.29967
606.088	11.09770	5.81062	617.88	10.15520	6.01364
626.088	10.64420	5.89211	637.88	10.21110	5.87158
646.088	10.50630	5.43012	657.88	9.89431	5.72258
666.088	10.22230	5.27187	677.88	9.48614	5.70879
686.088	10.10570	5.21805	697.88	9.36649	5.63965
706.088	9.59069	5.12132	757.88	8.92637	5.23016
726.088	9.40290	5.01199	777.88	8.81040	5.27195
746.088	9.00328	4.82041	797.88	8.65296	5.08718
766.088	9.06814	4.76048	817.88	8.52251	5.04325
786.088	8.94637	4.71286	837.88	8.51721	5.01333
806.088	8.73762	4.67892	857.88	8.23749	4.90637

826.088	8.62387	4.61390	877.88	8.19164	4.82313
846.088	8.51757	4.62255	897.88	8.15908	4.71552
866.088	8.34371	4.55692	917.88	8.06261	4.68441
886.088	8.35404	4.43845	937.88	7.95531	4.71040
906.088	8.23518	4.21660	957.88	7.89915	4.67191
926.088	8.13301	4.35031	977.88	7.78696	4.57892
946.088	7.88830	4.34510	997.88	7.89924	4.53217
966.088	7.98088	4.14302	1017.88	7.78124	4.41628
1026.088	7.79282	3.92851	1037.88	7.71578	4.40764
1046.088	7.86367	3.77898	1057.88	7.71865	4.26647
1066.088	7.97732	3.65400	1077.88	7.64023	4.23841
1086.088	7.98133	3.57675	1097.88	7.60499	4.30532
1106.088	7.75122	3.62408	1117.88	7.57399	4.27710
1126.088	7.69387	3.63620	1137.88	7.54711	4.27719
1146.088	7.65338	3.44996	1157.88	7.50602	4.16396
1166.088	7.73175	3.46211	1177.88	7.39555	4.10822
1186.088	7.70422	3.40319	1197.88	7.40784	4.06309
1206.088	7.59227	3.46134	1217.88	7.39313	3.96390
1226.088	7.65219	3.36027	1237.88	7.28439	4.00133
1246.088	7.57414	3.30947	1257.88	7.27094	3.94428
1266.088	7.41881	3.31294	1277.88	7.23007	3.93188
1286.088	7.39781	3.30939	1297.88	7.17334	3.92180
1306.088	7.60810	3.26566	1317.88	7.13689	3.93432
1326.088	7.56758	3.24324	1337.88	7.04047	3.81681
1346.088	7.50733	3.26455	1357.88	7.02828	3.83938
1366.088	7.47600	3.30056	1377.88	7.08689	3.77903
1386.088	7.43524	3.14226	1397.88	7.04156	3.80096
1406.088	7.29397	3.16755	1417.88	7.01581	3.71905
1426.088	7.36401	3.18494	1437.88	7.00942	3.67555
1446.088	7.33611	3.16057	1457.88	6.98826	3.69990
1466.088	7.27586	3.15551	1477.88	6.89612	3.66951
1486.088	7.45686	3.04342	1497.88	6.89576	3.66761
1506.088	7.39279	2.99510	1517.88	6.89577	3.58438
1526.088	7.30376	3.08864	1537.88	6.81425	3.56315
1546.088	7.36869	3.05819	1557.88	6.81563	3.55649
1566.088	7.29395	2.97105	1577.88	6.79369	3.55213
1586.088	7.16643	2.98030	1597.88	6.76122	3.51739
1606.088	7.18118	2.98480	1617.88	6.72134	3.42503
1626.088	7.27749	2.99661	1637.88	6.65485	3.39780
1646.088	7.17796	2.99547	1657.88	6.70515	3.46157
1666.088	7.16459	2.92050	1677.88	6.61119	3.41250
1686.088	7.24949	2.86346	1697.88	6.63958	3.42083
1706.088	7.31458	2.94247	1717.88	6.60618	3.39155
1726.088	7.24432	2.99146	1737.88	6.54950	3.37435
1746.088	7.22836	2.96686	1757.88	6.54258	3.34917
1766.088	7.19943	2.93128	1777.88	6.47043	3.34811
1786.088	7.17653	2.90896	1797.88	6.47373	3.28879
1806.088	7.28160	2.91597	1817.88	6.45233	3.30541
1826.088	7.26013	2.91128	1837.88	6.37877	3.29603
1846.088	7.21228	2.85264	1857.88	6.43591	3.27312

1866.088	7.25478	2.87057	1877.88	6.44874	3.28290
1886.088	7.25542	2.83776	1897.88	6.37719	3.26256
1906.088	7.26647	2.88863	1917.88	6.38815	3.25925
1926.088	7.33440	2.86540	1937.88	6.38462	3.22889
1946.088	7.22220	2.87587	1957.88	6.33334	3.21700
1966.088	7.20444	2.87446	1977.88	6.31344	3.21109
1986.088	7.14213	2.88946	1997.88	6.35371	3.21165
2006.088	7.13243	2.86879	2017.88	6.31226	3.21068
2026.088	7.15149	2.76502			
2046.088	7.18854	2.79412			
2066.088	7.17355	2.76346			
2086.088	7.15920	2.77653			
2106.088	7.14071	2.78638			
2126.088	7.15458	2.81027			
2146.088	7.15017	2.78365			
2166.088	7.05248	2.82529			
2186.088	7.01395	2.77718			
2206.088	7.02589	2.74210			
2226.088	7.01235	2.72605			
2246.088	7.01921	2.69650			
2266.088	7.06153	2.72136			
2286.088	7.05743	2.72192			

E_{trans}(cm^{-1})	51->31	E_{trans}(cm^{-1})	03->01
2.620	116.0310	0.4	105.697
22.620	35.18710	20.4	17.1954
42.620	25.94300	40.4	12.2222
62.620	22.82010	60.4	10.7640
82.620	20.43350	80.4	8.73902
102.620	18.77820	100.4	7.37186
122.620	17.12360	140.4	5.64423
142.620	15.97120	160.4	5.00898
162.620	14.98630	180.4	4.95149
182.620	14.26760	200.4	4.58489
202.620	13.32070	220.4	4.02369
222.620	12.91930	240.4	3.45638
242.620	12.77840	260.4	3.59213
262.620	12.12850	280.4	3.61241
282.620	11.74510	300.4	3.31142
302.620	11.47360	320.4	3.02380
322.620	11.23200	340.4	3.19049
342.620	10.97590	360.4	2.92672
362.620	10.72320	380.4	2.95922
422.620	9.81866	400.4	2.85024
442.620	9.34471	420.4	2.87986
462.620	8.98079	440.4	2.99300
482.620	8.60776	460.4	2.64067
502.620	8.37169	480.4	2.42635
522.620	8.18712	500.4	2.42351
542.620	7.86751	520.4	2.35255

562.620	7.65092	540.4	2.35808
582.620	7.43470	560.4	2.06809
602.620	7.20014	580.4	2.04391
622.620	7.00514	600.4	1.91117
642.620	6.73258	620.4	1.77976
662.620	6.61594	640.4	1.66681
682.620	6.41528	660.4	1.65568
702.620	6.27368	680.4	1.66334
722.620	6.15422	700.4	1.44926
742.620	6.12105	720.4	1.54247
762.620	5.91891	740.4	1.32595
782.620	5.84365	760.4	1.34123
802.620	5.73064	820.4	1.18503
822.620	5.55911	840.4	1.18587
842.620	5.44821	860.4	1.23224
862.620	5.38236	880.4	1.13670
882.620	5.23985	900.4	1.15233
902.620	5.11873	920.4	1.08137
922.620	4.96065	940.4	1.07094
942.620	4.87389	960.4	1.10272
962.620	4.84171	980.4	1.15723
982.620	4.72072	1000.4	1.11942
1002.620	4.62401	1020.4	1.11412
1022.620	4.55391	1040.4	1.09351
1042.620	4.52088	1060.4	1.05545
1062.620	4.44190	1080.4	1.06196
1082.620	4.37927	1100.4	1.04787
1102.620	4.33388	1120.4	1.05106
1122.620	4.33715	1140.4	1.10315
1142.620	4.27260	1160.4	1.05582
1162.620	4.22632	1180.4	1.06592
1182.620	4.14620	1200.4	1.05130
1202.620	4.11580	1220.4	0.99087
1222.620	4.06894	1240.4	0.92847
1242.620	4.04325	1260.4	0.93431
1262.620	4.00650	1280.4	0.95952
1282.620	4.03085	1300.4	0.94777
1302.620	3.95834	1320.4	0.94054
1322.620	3.92823	1340.4	0.89719
1342.620	3.92266	1360.4	0.89764
1362.620	3.89053	1380.4	0.87872
1382.620	3.83234	1400.4	0.93692
1402.620	3.81462	1420.4	0.83572
1422.620	3.77022	1440.4	0.85182
1442.620	3.74319	1460.4	0.88676
1462.620	3.77010	1480.4	0.85927
1482.620	3.73216	1500.4	0.82328
1502.620	3.66586	1520.4	0.78512
1522.620	3.65376	1540.4	0.80078
1542.620	3.62647	1560.4	0.81434

1562.620	3.58420	1580.4	0.78384
1582.620	3.58556	1600.4	0.78896
1602.620	3.54500	1620.4	0.74528
1622.620	3.53588	1640.4	0.81774
1642.620	3.52776	1660.4	0.79497
1662.620	3.51685	1680.4	0.76391
1682.620	3.48216	1700.4	0.80440
		1720.4	0.80220
		1740.4	0.77943
		1760.4	0.75654
		1780.4	0.78637
		1800.4	0.75803
		1820.4	0.78266
		1840.4	0.76388
		1860.4	0.76662
		1880.4	0.74864
		1900.4	0.78989
		1920.4	0.75924
		1940.4	0.74322
		1960.4	0.74194
		1980.4	0.74292
		2000.4	0.74463
		2020.4	0.73382
		2040.4	0.75556
		2060.4	0.77311
		2080.4	0.74817

Table S5: Computed excitation rate coefficients in cm^3 $molecule^{-1}$ s^{-1} units for a series of inelastic processes generated using the 4D RR-PES for the $HeH^+(j_1)\cdots$ para-$H_2(j_2 = 0)$ system with $\Delta j_1 = +1$ and $\Delta j_1 = +2$.

T (K)	00->10	00->20	10->20	10->30
5.0	4.17805E-18	1.88592E-35	4.73523E-27	2.03078E-52
6.0	9.48612E-17	3.43523E-31	3.48311E-24	2.18368E-45
7.0	8.80712E-16	3.76069E-28	3.85925E-22	2.30229E-40
8.0	4.71535E-15	7.14985E-26	1.31922E-20	1.35738E-36
9.0	1.75410E-14	4.24574E-24	2.06571E-19	1.17293E-33
10.0	5.05706E-14	1.11787E-22	1.87446E-18	2.64225E-31
11.0	1.20998E-13	1.62937E-21	1.14363E-17	2.23637E-29
12.0	2.51420E-13	1.52376E-20	5.17798E-17	9.07225E-28
13.0	4.68221E-13	1.01249E-19	1.86266E-16	2.08797E-26
14.0	7.99427E-13	5.14155E-19	5.58979E-16	3.07586E-25
15.0	1.27258E-12	2.10500E-18	1.45050E-15	3.16911E-24
16.0	1.91295E-12	7.23280E-18	3.34361E-15	2.44103E-23
17.0	2.74234E-12	2.15090E-17	6.98993E-15	1.47927E-22
18.0	3.77833E-12	5.67024E-17	1.34679E-14	7.33906E-22
19.0	5.03403E-12	1.35044E-16	2.42241E-14	3.07622E-21
20.0	6.51820E-12	2.95010E-16	4.10929E-14	1.11718E-20
21.0	8.23554E-12	5.98443E-16	6.62930E-14	3.58788E-20
22.0	1.01872E-11	1.13870E-15	1.02403E-13	1.03615E-19
23.0	1.23711E-11	2.04934E-15	1.52316E-13	2.72828E-19

24.0	1.47827E-11	3.51278E-15	2.19188E-13	6.62592E-19
25.0	1.74154E-11	5.76837E-15	3.06366E-13	1.49867E-18
26.0	2.02610E-11	9.11939E-15	4.17323E-13	3.18297E-18
27.0	2.33098E-11	1.39385E-14	5.55588E-13	6.39239E-18
28.0	2.65516E-11	2.06714E-14	7.24680E-13	1.22124E-17
29.0	2.99753E-11	2.98386E-14	9.28046E-13	2.23098E-17
30.0	3.35695E-11	4.20352E-14	1.16901E-12	3.91460E-17
31.0	3.73227E-11	5.79285E-14	1.45073E-12	6.62330E-17
32.0	4.12235E-11	7.82543E-14	1.77615E-12	1.08427E-16
33.0	4.52602E-11	1.03811E-13	2.14798E-12	1.72260E-16
34.0	4.94219E-11	1.35455E-13	2.56868E-12	2.66293E-16
35.0	5.36975E-11	1.74089E-13	3.04043E-12	4.01500E-16
36.0	5.80764E-11	2.20658E-13	3.56513E-12	5.91651E-16
37.0	6.25486E-11	2.76136E-13	4.14440E-12	8.53703E-16
38.0	6.71043E-11	3.41522E-13	4.77958E-12	1.20818E-15
39.0	7.17343E-11	4.17828E-13	5.47173E-12	1.67954E-15
40.0	7.64296E-11	5.06070E-13	6.22166E-12	2.29648E-15
41.0	8.11819E-11	6.07259E-13	7.02989E-12	3.09226E-15
42.0	8.59833E-11	7.22398E-13	7.89672E-12	4.10495E-15
43.0	9.08264E-11	8.52468E-13	8.82223E-12	5.37760E-15
44.0	9.57040E-11	9.98424E-13	9.80628E-12	6.95843E-15
45.0	1.00610E-10	1.16119E-12	1.08485E-11	8.90088E-15
46.0	1.05537E-10	1.34166E-12	1.19484E-11	1.12637E-14
47.0	1.10481E-10	1.54067E-12	1.31053E-11	1.41108E-14
48.0	1.15435E-10	1.75904E-12	1.43184E-11	1.75115E-14
49.0	1.20395E-10	1.99749E-12	1.55866E-11	2.15398E-14
50.0	1.25357E-10	2.25675E-12	1.69089E-11	2.62750E-14
51.0	1.30315E-10	2.53746E-12	1.82842E-11	3.18008E-14
52.0	1.35265E-10	2.84021E-12	1.97110E-11	3.82052E-14
53.0	1.40205E-10	3.16554E-12	2.11881E-11	4.55806E-14
54.0	1.45131E-10	3.51393E-12	2.27140E-11	5.40229E-14
55.0	1.50039E-10	3.88581E-12	2.42873E-11	6.36317E-14
56.0	1.54927E-10	4.28155E-12	2.59063E-11	7.45092E-14
57.0	1.59791E-10	4.70147E-12	2.75696E-11	8.67608E-14
58.0	1.64631E-10	5.14583E-12	2.92754E-11	1.00494E-13
59.0	1.69443E-10	5.61485E-12	3.10223E-11	1.15817E-13
60.0	1.74226E-10	6.10868E-12	3.28085E-11	1.32842E-13
61.0	1.78977E-10	6.62745E-12	3.46325E-11	1.51680E-13
62.0	1.83696E-10	7.17121E-12	3.64925E-11	1.72444E-13
63.0	1.88380E-10	7.73998E-12	3.83870E-11	1.95246E-13
64.0	1.93029E-10	8.33375E-12	4.03143E-11	2.20199E-13
65.0	1.97641E-10	8.95245E-12	4.22728E-11	2.47414E-13
66.0	2.02215E-10	9.59597E-12	4.42608E-11	2.77004E-13
67.0	2.06750E-10	1.02642E-11	4.62770E-11	3.09078E-13
68.0	2.11246E-10	1.09569E-11	4.83195E-11	3.43744E-13
69.0	2.15701E-10	1.16740E-11	5.03871E-11	3.81110E-13
70.0	2.20116E-10	1.24151E-11	5.24780E-11	4.21281E-13
71.0	2.24489E-10	1.31800E-11	5.45910E-11	4.64359E-13
72.0	2.28821E-10	1.39684E-11	5.67245E-11	5.10443E-13
73.0	2.33111E-10	1.47801E-11	5.88771E-11	5.59631E-13

74.0	2.37359E-10	1.56146E-11	6.10476E-11	6.12018E-13
75.0	2.41564E-10	1.64716E-11	6.32344E-11	6.67693E-13
76.0	2.45727E-10	1.73508E-11	6.54364E-11	7.26746E-13
77.0	2.49847E-10	1.82517E-11	6.76523E-11	7.89260E-13
78.0	2.53924E-10	1.91740E-11	6.98809E-11	8.55316E-13
79.0	2.57959E-10	2.01172E-11	7.21209E-11	9.24992E-13
80.0	2.61951E-10	2.10808E-11	7.43713E-11	9.98361E-13
81.0	2.65901E-10	2.20645E-11	7.66309E-11	1.07549E-12
82.0	2.69809E-10	2.30678E-11	7.88986E-11	1.15645E-12
83.0	2.73675E-10	2.40903E-11	8.11734E-11	1.24130E-12
84.0	2.77498E-10	2.51314E-11	8.34543E-11	1.33010E-12
85.0	2.81281E-10	2.61907E-11	8.57403E-11	1.42290E-12
86.0	2.85021E-10	2.72677E-11	8.80304E-11	1.51975E-12
87.0	2.88721E-10	2.83620E-11	9.03239E-11	1.62071E-12
88.0	2.92380E-10	2.94730E-11	9.26198E-11	1.72580E-12
89.0	2.95999E-10	3.06004E-11	9.49172E-11	1.83508E-12
90.0	2.99578E-10	3.17435E-11	9.72155E-11	1.94857E-12
91.0	3.03117E-10	3.29019E-11	9.95137E-11	2.06631E-12
92.0	3.06616E-10	3.40752E-11	1.01811E-10	2.18832E-12
93.0	3.10077E-10	3.52628E-11	1.04107E-10	2.31463E-12
94.0	3.13499E-10	3.64643E-11	1.06401E-10	2.44525E-12
95.0	3.16884E-10	3.76792E-11	1.08692E-10	2.58021E-12
96.0	3.20230E-10	3.89071E-11	1.10980E-10	2.71952E-12
97.0	3.23539E-10	4.01474E-11	1.13264E-10	2.86319E-12
98.0	3.26812E-10	4.13997E-11	1.15543E-10	3.01122E-12
99.0	3.30048E-10	4.26635E-11	1.17817E-10	3.16362E-12
100.0	3.33248E-10	4.39385E-11	1.20086E-10	3.32040E-12
101.0	3.36412E-10	4.52240E-11	1.22348E-10	3.48153E-12
102.0	3.39541E-10	4.65198E-11	1.24604E-10	3.64703E-12
103.0	3.42636E-10	4.78254E-11	1.26853E-10	3.81689E-12
104.0	3.45697E-10	4.91403E-11	1.29094E-10	3.99109E-12
105.0	3.48723E-10	5.04641E-11	1.31327E-10	4.16961E-12
106.0	3.51717E-10	5.17964E-11	1.33553E-10	4.35246E-12
107.0	3.54677E-10	5.31368E-11	1.35769E-10	4.53959E-12
108.0	3.57605E-10	5.44849E-11	1.37977E-10	4.73101E-12
109.0	3.60501E-10	5.58402E-11	1.40175E-10	4.92668E-12
110.0	3.63366E-10	5.72025E-11	1.42364E-10	5.12657E-12
111.0	3.66199E-10	5.85713E-11	1.44543E-10	5.33067E-12
112.0	3.69001E-10	5.99463E-11	1.46711E-10	5.53895E-12
113.0	3.71773E-10	6.13270E-11	1.48870E-10	5.75136E-12
114.0	3.74516E-10	6.27131E-11	1.51017E-10	5.96789E-12
115.0	3.77228E-10	6.41044E-11	1.53154E-10	6.18850E-12
116.0	3.79912E-10	6.55003E-11	1.55279E-10	6.41315E-12
117.0	3.82567E-10	6.69007E-11	1.57394E-10	6.64180E-12
118.0	3.85194E-10	6.83051E-11	1.59496E-10	6.87442E-12
119.0	3.87793E-10	6.97133E-11	1.61587E-10	7.11097E-12
120.0	3.90364E-10	7.11249E-11	1.63667E-10	7.35141E-12
121.0	3.92909E-10	7.25396E-11	1.65734E-10	7.59570E-12
122.0	3.95426E-10	7.39572E-11	1.67789E-10	7.84378E-12
123.0	3.97918E-10	7.53772E-11	1.69832E-10	8.09563E-12

124.0	4.00383E-10	7.67996E-11	1.71863E-10	8.35119E-12
125.0	4.02823E-10	7.82239E-11	1.73881E-10	8.61042E-12
126.0	4.05238E-10	7.96499E-11	1.75886E-10	8.87327E-12
127.0	4.07628E-10	8.10773E-11	1.77879E-10	9.13969E-12
128.0	4.09993E-10	8.25059E-11	1.79859E-10	9.40965E-12
129.0	4.12335E-10	8.39355E-11	1.81826E-10	9.68308E-12
130.0	4.14652E-10	8.53657E-11	1.83781E-10	9.95994E-12
131.0	4.16946E-10	8.67964E-11	1.85722E-10	1.02402E-11
132.0	4.19217E-10	8.82273E-11	1.87651E-10	1.05238E-11
133.0	4.21466E-10	8.96582E-11	1.89566E-10	1.08106E-11
134.0	4.23691E-10	9.10889E-11	1.91468E-10	1.11007E-11
135.0	4.25895E-10	9.25191E-11	1.93358E-10	1.13940E-11
136.0	4.28077E-10	9.39487E-11	1.95234E-10	1.16903E-11
137.0	4.30238E-10	9.53774E-11	1.97097E-10	1.19898E-11
138.0	4.32377E-10	9.68052E-11	1.98947E-10	1.22923E-11
139.0	4.34496E-10	9.82317E-11	2.00784E-10	1.25978E-11
140.0	4.36593E-10	9.96567E-11	2.02608E-10	1.29061E-11
141.0	4.38671E-10	1.01080E-10	2.04418E-10	1.32174E-11
142.0	4.40729E-10	1.02502E-10	2.06216E-10	1.35315E-11
143.0	4.42767E-10	1.03922E-10	2.08000E-10	1.38483E-11
144.0	4.44785E-10	1.05340E-10	2.09771E-10	1.41679E-11
145.0	4.46785E-10	1.06755E-10	2.11530E-10	1.44901E-11
146.0	4.48765E-10	1.08168E-10	2.13275E-10	1.48149E-11
147.0	4.50727E-10	1.09579E-10	2.15007E-10	1.51423E-11
148.0	4.52671E-10	1.10987E-10	2.16726E-10	1.54722E-11
149.0	4.54596E-10	1.12392E-10	2.18433E-10	1.58045E-11
150.0	4.56504E-10	1.13794E-10	2.20126E-10	1.61393E-11
151.0	4.58394E-10	1.15193E-10	2.21807E-10	1.64764E-11
152.0	4.60267E-10	1.16589E-10	2.23475E-10	1.68158E-11
153.0	4.62123E-10	1.17982E-10	2.25130E-10	1.71575E-11
154.0	4.63962E-10	1.19371E-10	2.26772E-10	1.75014E-11
155.0	4.65784E-10	1.20756E-10	2.28402E-10	1.78474E-11
156.0	4.67590E-10	1.22138E-10	2.30019E-10	1.81956E-11
157.0	4.69380E-10	1.23517E-10	2.31624E-10	1.85458E-11
158.0	4.71153E-10	1.24891E-10	2.33216E-10	1.88981E-11
159.0	4.72911E-10	1.26262E-10	2.34796E-10	1.92522E-11
160.0	4.74654E-10	1.27628E-10	2.36364E-10	1.96084E-11
161.0	4.76381E-10	1.28990E-10	2.37919E-10	1.99663E-11
162.0	4.78094E-10	1.30348E-10	2.39463E-10	2.03262E-11
163.0	4.79791E-10	1.31702E-10	2.40994E-10	2.06877E-11
164.0	4.81474E-10	1.33052E-10	2.42513E-10	2.10511E-11
165.0	4.83142E-10	1.34397E-10	2.44020E-10	2.14161E-11
166.0	4.84796E-10	1.35737E-10	2.45515E-10	2.17827E-11
167.0	4.86436E-10	1.37073E-10	2.46998E-10	2.21510E-11
168.0	4.88061E-10	1.38404E-10	2.48470E-10	2.25208E-11
169.0	4.89674E-10	1.39731E-10	2.49930E-10	2.28921E-11
170.0	4.91272E-10	1.41053E-10	2.51378E-10	2.32649E-11
171.0	4.92858E-10	1.42370E-10	2.52815E-10	2.36391E-11
172.0	4.94430E-10	1.43682E-10	2.54240E-10	2.40146E-11
173.0	4.95989E-10	1.44989E-10	2.55654E-10	2.43916E-11

174.0	4.97535E-10	1.46291E-10	2.57057E-10	2.47698E-11
175.0	4.99069E-10	1.47588E-10	2.58449E-10	2.51493E-11
176.0	5.00590E-10	1.48880E-10	2.59829E-10	2.55300E-11
177.0	5.02099E-10	1.50167E-10	2.61199E-10	2.59119E-11
178.0	5.03596E-10	1.51449E-10	2.62557E-10	2.62950E-11
179.0	5.05080E-10	1.52725E-10	2.63905E-10	2.66791E-11
180.0	5.06553E-10	1.53997E-10	2.65242E-10	2.70643E-11
181.0	5.08014E-10	1.55263E-10	2.66568E-10	2.74506E-11
182.0	5.09464E-10	1.56524E-10	2.67884E-10	2.78378E-11
183.0	5.10902E-10	1.57779E-10	2.69189E-10	2.82260E-11
184.0	5.12329E-10	1.59029E-10	2.70483E-10	2.86151E-11
185.0	5.13744E-10	1.60274E-10	2.71768E-10	2.90051E-11
186.0	5.15149E-10	1.61513E-10	2.73042E-10	2.93960E-11
187.0	5.16543E-10	1.62747E-10	2.74305E-10	2.97877E-11
188.0	5.17926E-10	1.63976E-10	2.75559E-10	3.01801E-11
189.0	5.19299E-10	1.65199E-10	2.76803E-10	3.05733E-11
190.0	5.20661E-10	1.66416E-10	2.78036E-10	3.09673E-11
191.0	5.22013E-10	1.67628E-10	2.79260E-10	3.13619E-11
192.0	5.23354E-10	1.68835E-10	2.80474E-10	3.17571E-11
193.0	5.24686E-10	1.70035E-10	2.81679E-10	3.21530E-11
194.0	5.26007E-10	1.71231E-10	2.82874E-10	3.25495E-11
195.0	5.27319E-10	1.72421E-10	2.84059E-10	3.29465E-11
196.0	5.28621E-10	1.73605E-10	2.85235E-10	3.33441E-11
197.0	5.29914E-10	1.74784E-10	2.86401E-10	3.37422E-11
198.0	5.31197E-10	1.75957E-10	2.87559E-10	3.41407E-11
199.0	5.32470E-10	1.77124E-10	2.88707E-10	3.45397E-11
200.0	5.33735E-10	1.78286E-10	2.89846E-10	3.49391E-11
201.0	5.34990E-10	1.79443E-10	2.90976E-10	3.53389E-11
202.0	5.36236E-10	1.80594E-10	2.92097E-10	3.57391E-11
203.0	5.37473E-10	1.81739E-10	2.93209E-10	3.61396E-11
204.0	5.38702E-10	1.82878E-10	2.94312E-10	3.65404E-11
205.0	5.39922E-10	1.84012E-10	2.95406E-10	3.69415E-11
206.0	5.41133E-10	1.85141E-10	2.96492E-10	3.73428E-11
207.0	5.42336E-10	1.86264E-10	2.97570E-10	3.77444E-11
208.0	5.43530E-10	1.87381E-10	2.98639E-10	3.81462E-11
209.0	5.44716E-10	1.88493E-10	2.99699E-10	3.85482E-11
210.0	5.45894E-10	1.89599E-10	3.00751E-10	3.89503E-11
211.0	5.47063E-10	1.90699E-10	3.01795E-10	3.93526E-11
212.0	5.48225E-10	1.91794E-10	3.02831E-10	3.97550E-11
213.0	5.49378E-10	1.92884E-10	3.03859E-10	4.01575E-11
214.0	5.50524E-10	1.93968E-10	3.04879E-10	4.05600E-11
215.0	5.51662E-10	1.95046E-10	3.05890E-10	4.09627E-11
216.0	5.52793E-10	1.96119E-10	3.06894E-10	4.13653E-11
217.0	5.53915E-10	1.97186E-10	3.07890E-10	4.17680E-11
218.0	5.55031E-10	1.98248E-10	3.08878E-10	4.21706E-11
219.0	5.56139E-10	1.99305E-10	3.09859E-10	4.25732E-11
220.0	5.57239E-10	2.00355E-10	3.10832E-10	4.29758E-11
221.0	5.58332E-10	2.01401E-10	3.11798E-10	4.33783E-11
222.0	5.59419E-10	2.02441E-10	3.12756E-10	4.37807E-11
223.0	5.60498E-10	2.03475E-10	3.13707E-10	4.41830E-11

224.0	5.61570E-10	2.04505E-10	3.14650E-10	4.45852E-11
225.0	5.62635E-10	2.05528E-10	3.15587E-10	4.49872E-11
226.0	5.63693E-10	2.06547E-10	3.16516E-10	4.53891E-11
227.0	5.64744E-10	2.07560E-10	3.17438E-10	4.57908E-11
228.0	5.65789E-10	2.08567E-10	3.18353E-10	4.61923E-11
229.0	5.66827E-10	2.09570E-10	3.19261E-10	4.65936E-11
230.0	5.67858E-10	2.10567E-10	3.20162E-10	4.69946E-11
231.0	5.68883E-10	2.11558E-10	3.21056E-10	4.73954E-11
232.0	5.69902E-10	2.12545E-10	3.21944E-10	4.77960E-11
233.0	5.70914E-10	2.13526E-10	3.22825E-10	4.81963E-11
234.0	5.71920E-10	2.14502E-10	3.23699E-10	4.85963E-11
235.0	5.72919E-10	2.15472E-10	3.24567E-10	4.89960E-11
236.0	5.73913E-10	2.16438E-10	3.25428E-10	4.93954E-11
237.0	5.74900E-10	2.17398E-10	3.26283E-10	4.97945E-11
238.0	5.75881E-10	2.18353E-10	3.27131E-10	5.01932E-11
239.0	5.76857E-10	2.19303E-10	3.27973E-10	5.05916E-11
240.0	5.77826E-10	2.20248E-10	3.28809E-10	5.09896E-11
241.0	5.78790E-10	2.21188E-10	3.29639E-10	5.13872E-11
242.0	5.79747E-10	2.22122E-10	3.30462E-10	5.17844E-11
243.0	5.80699E-10	2.23052E-10	3.31280E-10	5.21812E-11
244.0	5.81646E-10	2.23977E-10	3.32091E-10	5.25776E-11
245.0	5.82586E-10	2.24896E-10	3.32896E-10	5.29736E-11
246.0	5.83521E-10	2.25811E-10	3.33696E-10	5.33691E-11
247.0	5.84451E-10	2.26720E-10	3.34490E-10	5.37642E-11
248.0	5.85375E-10	2.27625E-10	3.35278E-10	5.41588E-11
249.0	5.86294E-10	2.28525E-10	3.36060E-10	5.45529E-11
250.0	5.87207E-10	2.29419E-10	3.36836E-10	5.49466E-11
251.0	5.88116E-10	2.30309E-10	3.37607E-10	5.53397E-11
252.0	5.89018E-10	2.31194E-10	3.38373E-10	5.57324E-11
253.0	5.89916E-10	2.32074E-10	3.39132E-10	5.61245E-11
254.0	5.90809E-10	2.32950E-10	3.39887E-10	5.65161E-11
255.0	5.91696E-10	2.33820E-10	3.40636E-10	5.69072E-11
256.0	5.92578E-10	2.34686E-10	3.41379E-10	5.72977E-11
257.0	5.93456E-10	2.35547E-10	3.42118E-10	5.76877E-11
258.0	5.94328E-10	2.36404E-10	3.42851E-10	5.80771E-11
259.0	5.95196E-10	2.37255E-10	3.43579E-10	5.84660E-11
260.0	5.96059E-10	2.38102E-10	3.44301E-10	5.88543E-11
261.0	5.96917E-10	2.38945E-10	3.45019E-10	5.92420E-11
262.0	5.97770E-10	2.39783E-10	3.45731E-10	5.96291E-11
263.0	5.98618E-10	2.40616E-10	3.46439E-10	6.00156E-11
264.0	5.99462E-10	2.41444E-10	3.47142E-10	6.04015E-11
265.0	6.00301E-10	2.42268E-10	3.47839E-10	6.07868E-11
266.0	6.01136E-10	2.43088E-10	3.48532E-10	6.11714E-11
267.0	6.01966E-10	2.43903E-10	3.49220E-10	6.15555E-11
268.0	6.02791E-10	2.44713E-10	3.49904E-10	6.19389E-11
269.0	6.03612E-10	2.45519E-10	3.50582E-10	6.23216E-11
270.0	6.04429E-10	2.46321E-10	3.51256E-10	6.27038E-11
271.0	6.05241E-10	2.47118E-10	3.51925E-10	6.30852E-11
272.0	6.06049E-10	2.47911E-10	3.52590E-10	6.34660E-11
273.0	6.06853E-10	2.48699E-10	3.53250E-10	6.38462E-11

274.0	6.07653E-10	2.49483E-10	3.53906E-10	6.42256E-11
275.0	6.08448E-10	2.50263E-10	3.54557E-10	6.46044E-11
276.0	6.09239E-10	2.51038E-10	3.55204E-10	6.49825E-11
277.0	6.10026E-10	2.51809E-10	3.55846E-10	6.53599E-11
278.0	6.10808E-10	2.52576E-10	3.56484E-10	6.57367E-11
279.0	6.11587E-10	2.53339E-10	3.57118E-10	6.61127E-11
280.0	6.12362E-10	2.54098E-10	3.57748E-10	6.64880E-11
281.0	6.13133E-10	2.54852E-10	3.58373E-10	6.68626E-11
282.0	6.13899E-10	2.55602E-10	3.58995E-10	6.72365E-11
283.0	6.14662E-10	2.56348E-10	3.59612E-10	6.76097E-11
284.0	6.15421E-10	2.57090E-10	3.60225E-10	6.79822E-11
285.0	6.16176E-10	2.57828E-10	3.60834E-10	6.83540E-11
286.0	6.16927E-10	2.58562E-10	3.61439E-10	6.87250E-11
287.0	6.17675E-10	2.59292E-10	3.62040E-10	6.90953E-11
288.0	6.18419E-10	2.60018E-10	3.62637E-10	6.94648E-11
289.0	6.19159E-10	2.60740E-10	3.63230E-10	6.98337E-11
290.0	6.19895E-10	2.61458E-10	3.63820E-10	7.02017E-11
291.0	6.20628E-10	2.62172E-10	3.64405E-10	7.05691E-11
292.0	6.21357E-10	2.62882E-10	3.64987E-10	7.09357E-11
293.0	6.22082E-10	2.63588E-10	3.65565E-10	7.13015E-11
294.0	6.22804E-10	2.64290E-10	3.66139E-10	7.16666E-11
295.0	6.23522E-10	2.64988E-10	3.66710E-10	7.20309E-11
296.0	6.24237E-10	2.65683E-10	3.67277E-10	7.23944E-11
297.0	6.24948E-10	2.66374E-10	3.67841E-10	7.27572E-11
298.0	6.25656E-10	2.67061E-10	3.68400E-10	7.31193E-11
299.0	6.26361E-10	2.67744E-10	3.68957E-10	7.34805E-11
300.0	6.27062E-10	2.68424E-10	3.69510E-10	7.38410E-11
301.0	6.27759E-10	2.69099E-10	3.70059E-10	7.42008E-11
302.0	6.28454E-10	2.69772E-10	3.70605E-10	7.45597E-11
303.0	6.29145E-10	2.70440E-10	3.71147E-10	7.49179E-11
304.0	6.29833E-10	2.71105E-10	3.71687E-10	7.52753E-11
305.0	6.30517E-10	2.71766E-10	3.72222E-10	7.56319E-11
306.0	6.31199E-10	2.72424E-10	3.72755E-10	7.59877E-11
307.0	6.31877E-10	2.73078E-10	3.73284E-10	7.63427E-11
308.0	6.32552E-10	2.73728E-10	3.73810E-10	7.66970E-11
309.0	6.33223E-10	2.74375E-10	3.74333E-10	7.70505E-11
310.0	6.33892E-10	2.75019E-10	3.74853E-10	7.74032E-11
311.0	6.34558E-10	2.75659E-10	3.75369E-10	7.77550E-11
312.0	6.35220E-10	2.76295E-10	3.75882E-10	7.81061E-11
313.0	6.35879E-10	2.76928E-10	3.76392E-10	7.84565E-11
314.0	6.36536E-10	2.77558E-10	3.76900E-10	7.88060E-11
315.0	6.37189E-10	2.78184E-10	3.77404E-10	7.91547E-11
316.0	6.37840E-10	2.78807E-10	3.77905E-10	7.95026E-11
317.0	6.38487E-10	2.79426E-10	3.78403E-10	7.98497E-11
318.0	6.39132E-10	2.80042E-10	3.78898E-10	8.01961E-11
319.0	6.39773E-10	2.80655E-10	3.79390E-10	8.05416E-11
320.0	6.40412E-10	2.81264E-10	3.79880E-10	8.08863E-11
321.0	6.41048E-10	2.81871E-10	3.80366E-10	8.12303E-11
322.0	6.41681E-10	2.82474E-10	3.80850E-10	8.15734E-11
323.0	6.42311E-10	2.83073E-10	3.81330E-10	8.19157E-11

324.0	6.42939E-10	2.83670E-10	3.81808E-10	8.22573E-11
325.0	6.43563E-10	2.84263E-10	3.82284E-10	8.25980E-11
326.0	6.44185E-10	2.84853E-10	3.82756E-10	8.29379E-11
327.0	6.44804E-10	2.85440E-10	3.83226E-10	8.32770E-11
328.0	6.45421E-10	2.86024E-10	3.83693E-10	8.36153E-11
329.0	6.46034E-10	2.86604E-10	3.84157E-10	8.39529E-11
330.0	6.46645E-10	2.87182E-10	3.84619E-10	8.42896E-11
331.0	6.47254E-10	2.87756E-10	3.85078E-10	8.46255E-11
332.0	6.47860E-10	2.88328E-10	3.85535E-10	8.49606E-11
333.0	6.48463E-10	2.88896E-10	3.85988E-10	8.52949E-11
334.0	6.49063E-10	2.89461E-10	3.86440E-10	8.56284E-11
335.0	6.49661E-10	2.90024E-10	3.86889E-10	8.59611E-11
336.0	6.50257E-10	2.90583E-10	3.87335E-10	8.62930E-11
337.0	6.50850E-10	2.91140E-10	3.87779E-10	8.66241E-11
338.0	6.51440E-10	2.91693E-10	3.88220E-10	8.69544E-11
339.0	6.52028E-10	2.92243E-10	3.88659E-10	8.72838E-11
340.0	6.52613E-10	2.92791E-10	3.89096E-10	8.76125E-11
341.0	6.53196E-10	2.93336E-10	3.89530E-10	8.79404E-11
342.0	6.53777E-10	2.93877E-10	3.89962E-10	8.82675E-11
343.0	6.54355E-10	2.94416E-10	3.90391E-10	8.85938E-11
344.0	6.54930E-10	2.94952E-10	3.90818E-10	8.89192E-11
345.0	6.55503E-10	2.95486E-10	3.91243E-10	8.92439E-11
346.0	6.56074E-10	2.96016E-10	3.91665E-10	8.95678E-11
347.0	6.56643E-10	2.96544E-10	3.92085E-10	8.98909E-11
348.0	6.57209E-10	2.97069E-10	3.92503E-10	9.02132E-11
349.0	6.57773E-10	2.97591E-10	3.92919E-10	9.05347E-11
350.0	6.58334E-10	2.98110E-10	3.93332E-10	9.08553E-11
351.0	6.58893E-10	2.98627E-10	3.93744E-10	9.11752E-11
352.0	6.59450E-10	2.99141E-10	3.94153E-10	9.14943E-11
353.0	6.60005E-10	2.99652E-10	3.94560E-10	9.18126E-11
354.0	6.60557E-10	3.00161E-10	3.94965E-10	9.21302E-11
355.0	6.61107E-10	3.00667E-10	3.95367E-10	9.24469E-11
356.0	6.61655E-10	3.01170E-10	3.95768E-10	9.27628E-11
357.0	6.62201E-10	3.01671E-10	3.96166E-10	9.30779E-11
358.0	6.62744E-10	3.02169E-10	3.96563E-10	9.33923E-11
359.0	6.63286E-10	3.02665E-10	3.96957E-10	9.37059E-11
360.0	6.63825E-10	3.03158E-10	3.97350E-10	9.40186E-11
361.0	6.64362E-10	3.03648E-10	3.97740E-10	9.43306E-11
362.0	6.64897E-10	3.04136E-10	3.98128E-10	9.46418E-11
363.0	6.65430E-10	3.04621E-10	3.98515E-10	9.49522E-11
364.0	6.65960E-10	3.05104E-10	3.98899E-10	9.52619E-11
365.0	6.66489E-10	3.05584E-10	3.99282E-10	9.55707E-11
366.0	6.67016E-10	3.06062E-10	3.99662E-10	9.58788E-11
367.0	6.67540E-10	3.06538E-10	4.00041E-10	9.61861E-11
368.0	6.68062E-10	3.07010E-10	4.00418E-10	9.64926E-11
369.0	6.68583E-10	3.07481E-10	4.00793E-10	9.67983E-11
370.0	6.69101E-10	3.07949E-10	4.01166E-10	9.71033E-11
371.0	6.69617E-10	3.08415E-10	4.01537E-10	9.74075E-11
372.0	6.70132E-10	3.08878E-10	4.01906E-10	9.77109E-11
373.0	6.70644E-10	3.09339E-10	4.02274E-10	9.80135E-11

374.0	6.71155E-10	3.09798E-10	4.02639E-10	9.83154E-11
375.0	6.71663E-10	3.10254E-10	4.03003E-10	9.86165E-11
376.0	6.72169E-10	3.10708E-10	4.03365E-10	9.89168E-11
377.0	6.72674E-10	3.11159E-10	4.03726E-10	9.92164E-11
378.0	6.73177E-10	3.11609E-10	4.04085E-10	9.95151E-11
379.0	6.73677E-10	3.12056E-10	4.04441E-10	9.98132E-11
380.0	6.74176E-10	3.12501E-10	4.04797E-10	1.00110E-10
381.0	6.74673E-10	3.12943E-10	4.05150E-10	1.00407E-10
382.0	6.75168E-10	3.13383E-10	4.05502E-10	1.00703E-10
383.0	6.75661E-10	3.13821E-10	4.05852E-10	1.00998E-10
384.0	6.76153E-10	3.14257E-10	4.06201E-10	1.01292E-10
385.0	6.76642E-10	3.14691E-10	4.06548E-10	1.01585E-10
386.0	6.77130E-10	3.15122E-10	4.06893E-10	1.01878E-10
387.0	6.77616E-10	3.15551E-10	4.07236E-10	1.02170E-10
388.0	6.78100E-10	3.15979E-10	4.07578E-10	1.02461E-10
389.0	6.78582E-10	3.16403E-10	4.07919E-10	1.02752E-10
390.0	6.79063E-10	3.16826E-10	4.08258E-10	1.03041E-10
391.0	6.79541E-10	3.17247E-10	4.08595E-10	1.03330E-10
392.0	6.80018E-10	3.17666E-10	4.08931E-10	1.03619E-10
393.0	6.80493E-10	3.18082E-10	4.09265E-10	1.03906E-10
394.0	6.80967E-10	3.18496E-10	4.09597E-10	1.04193E-10
395.0	6.81438E-10	3.18909E-10	4.09928E-10	1.04479E-10
396.0	6.81908E-10	3.19319E-10	4.10258E-10	1.04764E-10
397.0	6.82377E-10	3.19727E-10	4.10586E-10	1.05049E-10
398.0	6.82843E-10	3.20133E-10	4.10913E-10	1.05333E-10
399.0	6.83308E-10	3.20538E-10	4.11238E-10	1.05616E-10
400.0	6.83771E-10	3.20940E-10	4.11562E-10	1.05898E-10
401.0	6.84233E-10	3.21340E-10	4.11884E-10	1.06179E-10
402.0	6.84693E-10	3.21738E-10	4.12205E-10	1.06460E-10
403.0	6.85151E-10	3.22134E-10	4.12524E-10	1.06740E-10
404.0	6.85607E-10	3.22529E-10	4.12842E-10	1.07020E-10
405.0	6.86062E-10	3.22921E-10	4.13159E-10	1.07299E-10
406.0	6.86516E-10	3.23311E-10	4.13474E-10	1.07577E-10
407.0	6.86967E-10	3.23700E-10	4.13788E-10	1.07854E-10
408.0	6.87417E-10	3.24086E-10	4.14100E-10	1.08130E-10
409.0	6.87866E-10	3.24471E-10	4.14411E-10	1.08406E-10
410.0	6.88313E-10	3.24854E-10	4.14721E-10	1.08681E-10
411.0	6.88758E-10	3.25235E-10	4.15029E-10	1.08956E-10
412.0	6.89202E-10	3.25614E-10	4.15336E-10	1.09229E-10
413.0	6.89644E-10	3.25991E-10	4.15642E-10	1.09502E-10
414.0	6.90084E-10	3.26366E-10	4.15946E-10	1.09775E-10
415.0	6.90523E-10	3.26739E-10	4.16249E-10	1.10046E-10
416.0	6.90961E-10	3.27111E-10	4.16551E-10	1.10317E-10
417.0	6.91397E-10	3.27481E-10	4.16852E-10	1.10587E-10
418.0	6.91831E-10	3.27849E-10	4.17151E-10	1.10856E-10
419.0	6.92264E-10	3.28215E-10	4.17449E-10	1.11125E-10
420.0	6.92695E-10	3.28580E-10	4.17746E-10	1.11393E-10
421.0	6.93125E-10	3.28942E-10	4.18041E-10	1.11661E-10
422.0	6.93554E-10	3.29303E-10	4.18335E-10	1.11927E-10
423.0	6.93981E-10	3.29662E-10	4.18628E-10	1.12193E-10

424.0	6.94406E-10	3.30020E-10	4.18920E-10	1.12458E-10
425.0	6.94830E-10	3.30375E-10	4.19211E-10	1.12723E-10
426.0	6.95253E-10	3.30729E-10	4.19500E-10	1.12987E-10
427.0	6.95674E-10	3.31082E-10	4.19788E-10	1.13250E-10
428.0	6.96093E-10	3.31432E-10	4.20075E-10	1.13513E-10
429.0	6.96512E-10	3.31781E-10	4.20361E-10	1.13774E-10
430.0	6.96928E-10	3.32128E-10	4.20646E-10	1.14036E-10
431.0	6.97344E-10	3.32474E-10	4.20929E-10	1.14296E-10
432.0	6.97758E-10	3.32818E-10	4.21212E-10	1.14556E-10
433.0	6.98170E-10	3.33160E-10	4.21493E-10	1.14815E-10
434.0	6.98581E-10	3.33500E-10	4.21773E-10	1.15073E-10
435.0	6.98991E-10	3.33839E-10	4.22052E-10	1.15331E-10
436.0	6.99399E-10	3.34177E-10	4.22330E-10	1.15588E-10
437.0	6.99806E-10	3.34512E-10	4.22606E-10	1.15845E-10
438.0	7.00212E-10	3.34846E-10	4.22882E-10	1.16100E-10
439.0	7.00616E-10	3.35179E-10	4.23156E-10	1.16355E-10
440.0	7.01019E-10	3.35510E-10	4.23430E-10	1.16610E-10
441.0	7.01420E-10	3.35839E-10	4.23702E-10	1.16864E-10
442.0	7.01820E-10	3.36167E-10	4.23974E-10	1.17117E-10
443.0	7.02219E-10	3.36493E-10	4.24244E-10	1.17369E-10
444.0	7.02616E-10	3.36818E-10	4.24513E-10	1.17621E-10
445.0	7.03013E-10	3.37141E-10	4.24781E-10	1.17872E-10
446.0	7.03407E-10	3.37462E-10	4.25048E-10	1.18123E-10
447.0	7.03801E-10	3.37783E-10	4.25314E-10	1.18372E-10
448.0	7.04193E-10	3.38101E-10	4.25579E-10	1.18622E-10
449.0	7.04584E-10	3.38418E-10	4.25843E-10	1.18870E-10
450.0	7.04973E-10	3.38734E-10	4.26106E-10	1.19118E-10
451.0	7.05362E-10	3.39048E-10	4.26368E-10	1.19365E-10
452.0	7.05749E-10	3.39360E-10	4.26629E-10	1.19612E-10
453.0	7.06134E-10	3.39671E-10	4.26889E-10	1.19858E-10
454.0	7.06519E-10	3.39981E-10	4.27147E-10	1.20103E-10
455.0	7.06902E-10	3.40289E-10	4.27405E-10	1.20348E-10
456.0	7.07284E-10	3.40596E-10	4.27662E-10	1.20592E-10
457.0	7.07664E-10	3.40901E-10	4.27918E-10	1.20835E-10
458.0	7.08044E-10	3.41205E-10	4.28173E-10	1.21078E-10
459.0	7.08422E-10	3.41507E-10	4.28427E-10	1.21320E-10
460.0	7.08799E-10	3.41808E-10	4.28680E-10	1.21561E-10
461.0	7.09174E-10	3.42108E-10	4.28933E-10	1.21802E-10
462.0	7.09549E-10	3.42406E-10	4.29184E-10	1.22042E-10
463.0	7.09922E-10	3.42703E-10	4.29434E-10	1.22282E-10
464.0	7.10294E-10	3.42998E-10	4.29683E-10	1.22521E-10
465.0	7.10664E-10	3.43293E-10	4.29932E-10	1.22759E-10
466.0	7.11034E-10	3.43585E-10	4.30179E-10	1.22997E-10
467.0	7.11402E-10	3.43877E-10	4.30426E-10	1.23234E-10
468.0	7.11769E-10	3.44166E-10	4.30672E-10	1.23471E-10
469.0	7.12135E-10	3.44455E-10	4.30916E-10	1.23707E-10
470.0	7.12500E-10	3.44742E-10	4.31160E-10	1.23942E-10
471.0	7.12863E-10	3.45028E-10	4.31403E-10	1.24177E-10
472.0	7.13226E-10	3.45313E-10	4.31645E-10	1.24411E-10
473.0	7.13587E-10	3.45596E-10	4.31886E-10	1.24644E-10

474.0	7.13947E-10	3.45878E-10	4.32127E-10	1.24877E-10
475.0	7.14306E-10	3.46159E-10	4.32366E-10	1.25109E-10
476.0	7.14663E-10	3.46438E-10	4.32605E-10	1.25341E-10
477.0	7.15020E-10	3.46716E-10	4.32842E-10	1.25572E-10
478.0	7.15375E-10	3.46993E-10	4.33079E-10	1.25802E-10
479.0	7.15730E-10	3.47268E-10	4.33315E-10	1.26032E-10
480.0	7.16083E-10	3.47543E-10	4.33550E-10	1.26261E-10
481.0	7.16434E-10	3.47816E-10	4.33785E-10	1.26490E-10
482.0	7.16785E-10	3.48087E-10	4.34018E-10	1.26718E-10
483.0	7.17135E-10	3.48358E-10	4.34251E-10	1.26945E-10
484.0	7.17483E-10	3.48627E-10	4.34483E-10	1.27172E-10
485.0	7.17831E-10	3.48895E-10	4.34714E-10	1.27398E-10
486.0	7.18177E-10	3.49162E-10	4.34944E-10	1.27624E-10
487.0	7.18522E-10	3.49427E-10	4.35173E-10	1.27849E-10
488.0	7.18866E-10	3.49692E-10	4.35402E-10	1.28074E-10
489.0	7.19209E-10	3.49955E-10	4.35629E-10	1.28298E-10
490.0	7.19551E-10	3.50217E-10	4.35856E-10	1.28521E-10
491.0	7.19892E-10	3.50477E-10	4.36083E-10	1.28744E-10
492.0	7.20232E-10	3.50737E-10	4.36308E-10	1.28966E-10
493.0	7.20570E-10	3.50995E-10	4.36532E-10	1.29187E-10
494.0	7.20908E-10	3.51252E-10	4.36756E-10	1.29408E-10
495.0	7.21244E-10	3.51508E-10	4.36979E-10	1.29629E-10
496.0	7.21580E-10	3.51763E-10	4.37201E-10	1.29849E-10
497.0	7.21914E-10	3.52016E-10	4.37423E-10	1.30068E-10
498.0	7.22247E-10	3.52269E-10	4.37644E-10	1.30287E-10
499.0	7.22579E-10	3.52520E-10	4.37864E-10	1.30505E-10
500.0	7.22910E-10	3.52770E-10	4.38083E-10	1.30722E-10

T (K)	20->30	20->40	30->40	30->50
5.0	1.46535E-35	1.41171E-70	3.02944E-45	7.72554E-86
6.0	2.56064E-31	1.32713E-60	1.77370E-39	2.78176E-73
7.0	2.74660E-28	1.89834E-53	2.52583E-35	2.59492E-64
8.0	5.18661E-26	4.54253E-48	3.38974E-32	1.39921E-57
9.0	3.08357E-24	7.03683E-44	9.31100E-30	2.43547E-52
10.0	8.16092E-23	1.59782E-40	8.38974E-28	3.83051E-48
11.0	1.19743E-21	8.95789E-38	3.35383E-26	1.04703E-44
12.0	1.12730E-20	1.75634E-35	7.27849E-25	7.69141E-42
13.0	7.53511E-20	1.53303E-33	9.86438E-24	2.05373E-39
14.0	3.84504E-19	7.07937E-32	9.22901E-23	2.47375E-37
15.0	1.58001E-18	1.96336E-30	6.41622E-22	1.57511E-35
16.0	5.44275E-18	3.59680E-29	3.50330E-21	5.97220E-34
17.0	1.62098E-17	4.68175E-28	1.56734E-20	1.47717E-32
18.0	4.27552E-17	4.58284E-27	5.93857E-20	2.55883E-31
19.0	1.01797E-16	3.52846E-26	1.95605E-19	3.28351E-30
20.0	2.22154E-16	2.21495E-25	5.71931E-19	3.26465E-29
21.0	4.49917E-16	1.16712E-24	1.50987E-18	2.60822E-28
22.0	8.54254E-16	5.28683E-24	3.64926E-18	1.72506E-27
23.0	1.53345E-15	2.09969E-23	8.16812E-18	9.68075E-27
24.0	2.62077E-15	7.43249E-23	1.70945E-17	4.70520E-26
25.0	4.28968E-15	2.37752E-22	3.37210E-17	2.01512E-25

26.0	6.75817E-15	6.95348E-22	6.31273E-17	7.71650E-25
27.0	1.02917E-14	1.87797E-21	1.12805E-16	2.67508E-24
28.0	1.52051E-14	4.72383E-21	1.93375E-16	8.48556E-24
29.0	2.18625E-14	1.11484E-20	3.19371E-16	2.48568E-23
30.0	3.06763E-14	2.48436E-20	5.10073E-16	6.77780E-23
31.0	4.21046E-14	5.25671E-20	7.90348E-16	1.73231E-22
32.0	5.66478E-14	1.06126E-19	1.19148E-15	4.17537E-22
33.0	7.48439E-14	2.05302E-19	1.75196E-15	9.54136E-22
34.0	9.72631E-14	3.82002E-19	2.51816E-15	2.07691E-21
35.0	1.24503E-13	6.85945E-19	3.54497E-15	4.32441E-21
36.0	1.57180E-13	1.19221E-18	4.89626E-15	8.64466E-21
37.0	1.95927E-13	2.01098E-18	6.64523E-15	1.66462E-20
38.0	2.41385E-13	3.29976E-18	8.87465E-15	3.09681E-20
39.0	2.94195E-13	5.27833E-18	1.16769E-14	5.58080E-20
40.0	3.54997E-13	8.24678E-18	1.51538E-14	9.76559E-20
41.0	4.24423E-13	1.26065E-17	1.94166E-14	1.66286E-19
42.0	5.03089E-13	1.88844E-17	2.45856E-14	2.76068E-19
43.0	5.91595E-13	2.77605E-17	3.07893E-14	4.47656E-19
44.0	6.90520E-13	4.00984E-17	3.81645E-14	7.10135E-19
45.0	8.00415E-13	5.69782E-17	4.68551E-14	1.10367E-18
46.0	9.21807E-13	7.97333E-17	5.70117E-14	1.68277E-18
47.0	1.05519E-12	1.09988E-16	6.87905E-14	2.52013E-18
48.0	1.20103E-12	1.49697E-16	8.23530E-14	3.71126E-18
49.0	1.35975E-12	2.01188E-16	9.78645E-14	5.37984E-18
50.0	1.53175E-12	2.67203E-16	1.15494E-13	7.68380E-18
51.0	1.71738E-12	3.50943E-16	1.35412E-13	1.08223E-17
52.0	1.91697E-12	4.56106E-16	1.57792E-13	1.50437E-17
53.0	2.13082E-12	5.86933E-16	1.82807E-13	2.06537E-17
54.0	2.35916E-12	7.48247E-16	2.10631E-13	2.80254E-17
55.0	2.60221E-12	9.45488E-16	2.41436E-13	3.76092E-17
56.0	2.86017E-12	1.18476E-15	2.75392E-13	4.99439E-17
57.0	3.13318E-12	1.47284E-15	3.12670E-13	6.56682E-17
58.0	3.42134E-12	1.81725E-15	3.53434E-13	8.55336E-17
59.0	3.72476E-12	2.22624E-15	3.97846E-13	1.10417E-16
60.0	4.04348E-12	2.70884E-15	4.46066E-13	1.41333E-16
61.0	4.37753E-12	3.27487E-15	4.98246E-13	1.79450E-16
62.0	4.72690E-12	3.93496E-15	5.54536E-13	2.26102E-16
63.0	5.09158E-12	4.70055E-15	6.15078E-13	2.82805E-16
64.0	5.47149E-12	5.58390E-15	6.80010E-13	3.51268E-16
65.0	5.86658E-12	6.59811E-15	7.49465E-13	4.33409E-16
66.0	6.27673E-12	7.75712E-15	8.23566E-13	5.31372E-16
67.0	6.70183E-12	9.07565E-15	9.02434E-13	6.47534E-16
68.0	7.14174E-12	1.05693E-14	9.86180E-13	7.84525E-16
69.0	7.59630E-12	1.22543E-14	1.07491E-12	9.45237E-16
70.0	8.06534E-12	1.41480E-14	1.16872E-12	1.13284E-15
71.0	8.54867E-12	1.62681E-14	1.26771E-12	1.35078E-15
72.0	9.04609E-12	1.86334E-14	1.37196E-12	1.60281E-15
73.0	9.55739E-12	2.12631E-14	1.48155E-12	1.89299E-15
74.0	1.00823E-11	2.41774E-14	1.59654E-12	2.22570E-15
75.0	1.06207E-11	2.73968E-14	1.71701E-12	2.60564E-15

76.0	1.11722E-11	3.09427E-14	1.84301E-12	3.03784E-15
77.0	1.17366E-11	3.48369E-14	1.97460E-12	3.52767E-15
78.0	1.23136E-11	3.91020E-14	2.11181E-12	4.08085E-15
79.0	1.29031E-11	4.37608E-14	2.25469E-12	4.70343E-15
80.0	1.35046E-11	4.88367E-14	2.40327E-12	5.40183E-15
81.0	1.41180E-11	5.43534E-14	2.55757E-12	6.18281E-15
82.0	1.47428E-11	6.03353E-14	2.71762E-12	7.05349E-15
83.0	1.53790E-11	6.68069E-14	2.88343E-12	8.02134E-15
84.0	1.60261E-11	7.37929E-14	3.05500E-12	9.09416E-15
85.0	1.66839E-11	8.13184E-14	3.23236E-12	1.02801E-14
86.0	1.73520E-11	8.94087E-14	3.41548E-12	1.15878E-14
87.0	1.80302E-11	9.80893E-14	3.60437E-12	1.30259E-14
88.0	1.87183E-11	1.07386E-13	3.79901E-12	1.46038E-14
89.0	1.94158E-11	1.17324E-13	3.99940E-12	1.63308E-14
90.0	2.01225E-11	1.27929E-13	4.20550E-12	1.82169E-14
91.0	2.08382E-11	1.39227E-13	4.41730E-12	2.02722E-14
92.0	2.15624E-11	1.51243E-13	4.63477E-12	2.25071E-14
93.0	2.22950E-11	1.64004E-13	4.85787E-12	2.49324E-14
94.0	2.30357E-11	1.77534E-13	5.08658E-12	2.75591E-14
95.0	2.37842E-11	1.91859E-13	5.32085E-12	3.03985E-14
96.0	2.45401E-11	2.07004E-13	5.56064E-12	3.34622E-14
97.0	2.53032E-11	2.22995E-13	5.80590E-12	3.67619E-14
98.0	2.60733E-11	2.39854E-13	6.05660E-12	4.03098E-14
99.0	2.68501E-11	2.57608E-13	6.31267E-12	4.41180E-14
100.0	2.76333E-11	2.76280E-13	6.57407E-12	4.81992E-14
101.0	2.84226E-11	2.95894E-13	6.84074E-12	5.25659E-14
102.0	2.92178E-11	3.16474E-13	7.11262E-12	5.72309E-14
103.0	3.00186E-11	3.38041E-13	7.38966E-12	6.22075E-14
104.0	3.08248E-11	3.60620E-13	7.67179E-12	6.75086E-14
105.0	3.16361E-11	3.84232E-13	7.95895E-12	7.31477E-14
106.0	3.24524E-11	4.08899E-13	8.25107E-12	7.91382E-14
107.0	3.32733E-11	4.34642E-13	8.54810E-12	8.54937E-14
108.0	3.40986E-11	4.61482E-13	8.84996E-12	9.22278E-14
109.0	3.49281E-11	4.89440E-13	9.15658E-12	9.93543E-14
110.0	3.57616E-11	5.18535E-13	9.46789E-12	1.06887E-13
111.0	3.65988E-11	5.48786E-13	9.78383E-12	1.14840E-13
112.0	3.74396E-11	5.80212E-13	1.01043E-11	1.23226E-13
113.0	3.82837E-11	6.12832E-13	1.04293E-11	1.32061E-13
114.0	3.91309E-11	6.46663E-13	1.07587E-11	1.41358E-13
115.0	3.99811E-11	6.81722E-13	1.10924E-11	1.51130E-13
116.0	4.08339E-11	7.18026E-13	1.14304E-11	1.61392E-13
117.0	4.16894E-11	7.55590E-13	1.17725E-11	1.72158E-13
118.0	4.25471E-11	7.94430E-13	1.21188E-11	1.83441E-13
119.0	4.34070E-11	8.34561E-13	1.24691E-11	1.95256E-13
120.0	4.42690E-11	8.75997E-13	1.28234E-11	2.07616E-13
121.0	4.51327E-11	9.18752E-13	1.31816E-11	2.20535E-13
122.0	4.59981E-11	9.62838E-13	1.35435E-11	2.34026E-13
123.0	4.68650E-11	1.00827E-12	1.39092E-11	2.48104E-13
124.0	4.77331E-11	1.05505E-12	1.42786E-11	2.62780E-13
125.0	4.86025E-11	1.10321E-12	1.46516E-11	2.78070E-13

126.0	4.94728E-11	1.15274E-12	1.50281E-11	2.93985E-13
127.0	5.03441E-11	1.20366E-12	1.54080E-11	3.10539E-13
128.0	5.12160E-11	1.25597E-12	1.57913E-11	3.27745E-13
129.0	5.20885E-11	1.30970E-12	1.61778E-11	3.45616E-13
130.0	5.29615E-11	1.36483E-12	1.65676E-11	3.64163E-13
131.0	5.38348E-11	1.42139E-12	1.69606E-11	3.83400E-13
132.0	5.47082E-11	1.47938E-12	1.73566E-11	4.03339E-13
133.0	5.55818E-11	1.53881E-12	1.77556E-11	4.23992E-13
134.0	5.64552E-11	1.59968E-12	1.81575E-11	4.45370E-13
135.0	5.73285E-11	1.66200E-12	1.85624E-11	4.67485E-13
136.0	5.82015E-11	1.72577E-12	1.89700E-11	4.90350E-13
137.0	5.90740E-11	1.79100E-12	1.93803E-11	5.13974E-13
138.0	5.99461E-11	1.85769E-12	1.97933E-11	5.38369E-13
139.0	6.08175E-11	1.92585E-12	2.02088E-11	5.63546E-13
140.0	6.16882E-11	1.99548E-12	2.06269E-11	5.89516E-13
141.0	6.25581E-11	2.06658E-12	2.10474E-11	6.16288E-13
142.0	6.34271E-11	2.13915E-12	2.14704E-11	6.43874E-13
143.0	6.42951E-11	2.21320E-12	2.18956E-11	6.72282E-13
144.0	6.51619E-11	2.28872E-12	2.23231E-11	7.01523E-13
145.0	6.60276E-11	2.36572E-12	2.27528E-11	7.31606E-13
146.0	6.68921E-11	2.44420E-12	2.31846E-11	7.62539E-13
147.0	6.77552E-11	2.52416E-12	2.36185E-11	7.94333E-13
148.0	6.86168E-11	2.60559E-12	2.40544E-11	8.26996E-13
149.0	6.94770E-11	2.68850E-12	2.44922E-11	8.60536E-13
150.0	7.03356E-11	2.77288E-12	2.49320E-11	8.94961E-13
151.0	7.11926E-11	2.85874E-12	2.53735E-11	9.30279E-13
152.0	7.20478E-11	2.94607E-12	2.58169E-11	9.66499E-13
153.0	7.29013E-11	3.03487E-12	2.62619E-11	1.00363E-12
154.0	7.37530E-11	3.12513E-12	2.67086E-11	1.04167E-12
155.0	7.46028E-11	3.21685E-12	2.71569E-11	1.08064E-12
156.0	7.54506E-11	3.31003E-12	2.76067E-11	1.12053E-12
157.0	7.62964E-11	3.40467E-12	2.80581E-11	1.16136E-12
158.0	7.71401E-11	3.50076E-12	2.85109E-11	1.20314E-12
159.0	7.79818E-11	3.59829E-12	2.89650E-11	1.24586E-12
160.0	7.88212E-11	3.69727E-12	2.94205E-11	1.28953E-12
161.0	7.96585E-11	3.79768E-12	2.98773E-11	1.33416E-12
162.0	8.04935E-11	3.89952E-12	3.03353E-11	1.37976E-12
163.0	8.13262E-11	4.00279E-12	3.07946E-11	1.42632E-12
164.0	8.21565E-11	4.10747E-12	3.12549E-11	1.47386E-12
165.0	8.29845E-11	4.21357E-12	3.17163E-11	1.52238E-12
166.0	8.38100E-11	4.32107E-12	3.21788E-11	1.57188E-12
167.0	8.46331E-11	4.42998E-12	3.26423E-11	1.62236E-12
168.0	8.54537E-11	4.54027E-12	3.31067E-11	1.67384E-12
169.0	8.62718E-11	4.65196E-12	3.35721E-11	1.72631E-12
170.0	8.70873E-11	4.76501E-12	3.40383E-11	1.77977E-12
171.0	8.79002E-11	4.87945E-12	3.45053E-11	1.83424E-12
172.0	8.87104E-11	4.99524E-12	3.49731E-11	1.88970E-12
173.0	8.95181E-11	5.11239E-12	3.54417E-11	1.94617E-12
174.0	9.03230E-11	5.23088E-12	3.59109E-11	2.00365E-12
175.0	9.11253E-11	5.35071E-12	3.63809E-11	2.06214E-12

176.0	9.19248E-11	5.47187E-12	3.68514E-11	2.12163E-12
177.0	9.27216E-11	5.59436E-12	3.73225E-11	2.18214E-12
178.0	9.35156E-11	5.71815E-12	3.77942E-11	2.24367E-12
179.0	9.43068E-11	5.84325E-12	3.82663E-11	2.30621E-12
180.0	9.50952E-11	5.96964E-12	3.87390E-11	2.36976E-12
181.0	9.58808E-11	6.09732E-12	3.92120E-11	2.43433E-12
182.0	9.66635E-11	6.22627E-12	3.96855E-11	2.49992E-12
183.0	9.74434E-11	6.35649E-12	4.01594E-11	2.56653E-12
184.0	9.82204E-11	6.48796E-12	4.06335E-11	2.63416E-12
185.0	9.89945E-11	6.62068E-12	4.11080E-11	2.70280E-12
186.0	9.97657E-11	6.75464E-12	4.15828E-11	2.77246E-12
187.0	1.00534E-10	6.88982E-12	4.20578E-11	2.84314E-12
188.0	1.01299E-10	7.02622E-12	4.25329E-11	2.91484E-12
189.0	1.02062E-10	7.16383E-12	4.30083E-11	2.98755E-12
190.0	1.02821E-10	7.30263E-12	4.34838E-11	3.06128E-12
191.0	1.03578E-10	7.44262E-12	4.39595E-11	3.13603E-12
192.0	1.04331E-10	7.58379E-12	4.44352E-11	3.21178E-12
193.0	1.05082E-10	7.72612E-12	4.49110E-11	3.28855E-12
194.0	1.05830E-10	7.86960E-12	4.53868E-11	3.36633E-12
195.0	1.06574E-10	8.01423E-12	4.58626E-11	3.44511E-12
196.0	1.07316E-10	8.16000E-12	4.63384E-11	3.52491E-12
197.0	1.08054E-10	8.30689E-12	4.68142E-11	3.60570E-12
198.0	1.08790E-10	8.45489E-12	4.72899E-11	3.68750E-12
199.0	1.09523E-10	8.60399E-12	4.77654E-11	3.77030E-12
200.0	1.10253E-10	8.75418E-12	4.82409E-11	3.85410E-12
201.0	1.10979E-10	8.90546E-12	4.87163E-11	3.93889E-12
202.0	1.11703E-10	9.05780E-12	4.91914E-11	4.02467E-12
203.0	1.12424E-10	9.21120E-12	4.96664E-11	4.11144E-12
204.0	1.13141E-10	9.36566E-12	5.01412E-11	4.19920E-12
205.0	1.13856E-10	9.52114E-12	5.06157E-11	4.28794E-12
206.0	1.14568E-10	9.67766E-12	5.10900E-11	4.37766E-12
207.0	1.15276E-10	9.83519E-12	5.15640E-11	4.46835E-12
208.0	1.15982E-10	9.99373E-12	5.20378E-11	4.56002E-12
209.0	1.16685E-10	1.01533E-11	5.25112E-11	4.65266E-12
210.0	1.17385E-10	1.03138E-11	5.29843E-11	4.74626E-12
211.0	1.18081E-10	1.04753E-11	5.34570E-11	4.84082E-12
212.0	1.18775E-10	1.06377E-11	5.39294E-11	4.93634E-12
213.0	1.19466E-10	1.08011E-11	5.44014E-11	5.03281E-12
214.0	1.20154E-10	1.09654E-11	5.48729E-11	5.13023E-12
215.0	1.20839E-10	1.11307E-11	5.53441E-11	5.22860E-12
216.0	1.21521E-10	1.12969E-11	5.58148E-11	5.32791E-12
217.0	1.22200E-10	1.14640E-11	5.62851E-11	5.42815E-12
218.0	1.22876E-10	1.16320E-11	5.67549E-11	5.52932E-12
219.0	1.23549E-10	1.18009E-11	5.72242E-11	5.63142E-12
220.0	1.24219E-10	1.19706E-11	5.76930E-11	5.73444E-12
221.0	1.24886E-10	1.21412E-11	5.81613E-11	5.83838E-12
222.0	1.25551E-10	1.23127E-11	5.86291E-11	5.94323E-12
223.0	1.26212E-10	1.24850E-11	5.90963E-11	6.04899E-12
224.0	1.26871E-10	1.26582E-11	5.95630E-11	6.15565E-12
225.0	1.27526E-10	1.28322E-11	6.00291E-11	6.26321E-12

226.0	1.28179E-10	1.30070E-11	6.04946E-11	6.37165E-12
227.0	1.28829E-10	1.31825E-11	6.09596E-11	6.48099E-12
228.0	1.29476E-10	1.33589E-11	6.14239E-11	6.59120E-12
229.0	1.30120E-10	1.35361E-11	6.18876E-11	6.70230E-12
230.0	1.30761E-10	1.37140E-11	6.23507E-11	6.81426E-12
231.0	1.31400E-10	1.38927E-11	6.28131E-11	6.92709E-12
232.0	1.32035E-10	1.40722E-11	6.32749E-11	7.04077E-12
233.0	1.32668E-10	1.42524E-11	6.37360E-11	7.15531E-12
234.0	1.33298E-10	1.44333E-11	6.41964E-11	7.27070E-12
235.0	1.33926E-10	1.46149E-11	6.46562E-11	7.38693E-12
236.0	1.34550E-10	1.47973E-11	6.51152E-11	7.50399E-12
237.0	1.35172E-10	1.49803E-11	6.55736E-11	7.62189E-12
238.0	1.35791E-10	1.51641E-11	6.60312E-11	7.74061E-12
239.0	1.36407E-10	1.53485E-11	6.64882E-11	7.86016E-12
240.0	1.37020E-10	1.55336E-11	6.69444E-11	7.98051E-12
241.0	1.37631E-10	1.57194E-11	6.73998E-11	8.10167E-12
242.0	1.38239E-10	1.59058E-11	6.78545E-11	8.22363E-12
243.0	1.38844E-10	1.60928E-11	6.83085E-11	8.34639E-12
244.0	1.39447E-10	1.62805E-11	6.87616E-11	8.46994E-12
245.0	1.40047E-10	1.64688E-11	6.92140E-11	8.59427E-12
246.0	1.40644E-10	1.66578E-11	6.96657E-11	8.71938E-12
247.0	1.41239E-10	1.68473E-11	7.01165E-11	8.84525E-12
248.0	1.41831E-10	1.70374E-11	7.05666E-11	8.97190E-12
249.0	1.42420E-10	1.72281E-11	7.10158E-11	9.09930E-12
250.0	1.43007E-10	1.74194E-11	7.14643E-11	9.22745E-12
251.0	1.43591E-10	1.76113E-11	7.19119E-11	9.35635E-12
252.0	1.44172E-10	1.78037E-11	7.23587E-11	9.48599E-12
253.0	1.44751E-10	1.79967E-11	7.28047E-11	9.61636E-12
254.0	1.45327E-10	1.81902E-11	7.32499E-11	9.74746E-12
255.0	1.45901E-10	1.83843E-11	7.36942E-11	9.87928E-12
256.0	1.46472E-10	1.85788E-11	7.41377E-11	1.00118E-11
257.0	1.47041E-10	1.87739E-11	7.45803E-11	1.01451E-11
258.0	1.47607E-10	1.89695E-11	7.50221E-11	1.02790E-11
259.0	1.48171E-10	1.91656E-11	7.54630E-11	1.04137E-11
260.0	1.48732E-10	1.93622E-11	7.59031E-11	1.05490E-11
261.0	1.49291E-10	1.95593E-11	7.63423E-11	1.06850E-11
262.0	1.49847E-10	1.97568E-11	7.67806E-11	1.08217E-11
263.0	1.50401E-10	1.99548E-11	7.72181E-11	1.09591E-11
264.0	1.50952E-10	2.01533E-11	7.76547E-11	1.10971E-11
265.0	1.51501E-10	2.03522E-11	7.80904E-11	1.12358E-11
266.0	1.52047E-10	2.05516E-11	7.85252E-11	1.13751E-11
267.0	1.52592E-10	2.07513E-11	7.89591E-11	1.15151E-11
268.0	1.53133E-10	2.09516E-11	7.93922E-11	1.16557E-11
269.0	1.53673E-10	2.11522E-11	7.98243E-11	1.17970E-11
270.0	1.54209E-10	2.13532E-11	8.02556E-11	1.19388E-11
271.0	1.54744E-10	2.15547E-11	8.06859E-11	1.20813E-11
272.0	1.55276E-10	2.17565E-11	8.11153E-11	1.22245E-11
273.0	1.55806E-10	2.19587E-11	8.15439E-11	1.23682E-11
274.0	1.56334E-10	2.21613E-11	8.19715E-11	1.25125E-11
275.0	1.56859E-10	2.23643E-11	8.23982E-11	1.26574E-11

276.0	1.57382E-10	2.25676E-11	8.28240E-11	1.28029E-11
277.0	1.57903E-10	2.27713E-11	8.32489E-11	1.29490E-11
278.0	1.58421E-10	2.29754E-11	8.36729E-11	1.30957E-11
279.0	1.58938E-10	2.31798E-11	8.40960E-11	1.32429E-11
280.0	1.59452E-10	2.33845E-11	8.45181E-11	1.33907E-11
281.0	1.59963E-10	2.35895E-11	8.49393E-11	1.35391E-11
282.0	1.60473E-10	2.37949E-11	8.53596E-11	1.36880E-11
283.0	1.60980E-10	2.40006E-11	8.57789E-11	1.38374E-11
284.0	1.61485E-10	2.42066E-11	8.61974E-11	1.39874E-11
285.0	1.61988E-10	2.44129E-11	8.66149E-11	1.41379E-11
286.0	1.62489E-10	2.46195E-11	8.70315E-11	1.42890E-11
287.0	1.62988E-10	2.48263E-11	8.74471E-11	1.44405E-11
288.0	1.63485E-10	2.50335E-11	8.78618E-11	1.45926E-11
289.0	1.63979E-10	2.52409E-11	8.82756E-11	1.47452E-11
290.0	1.64471E-10	2.54486E-11	8.86884E-11	1.48983E-11
291.0	1.64962E-10	2.56566E-11	8.91004E-11	1.50519E-11
292.0	1.65450E-10	2.58648E-11	8.95113E-11	1.52060E-11
293.0	1.65936E-10	2.60732E-11	8.99214E-11	1.53605E-11
294.0	1.66420E-10	2.62819E-11	9.03305E-11	1.55156E-11
295.0	1.66902E-10	2.64909E-11	9.07387E-11	1.56711E-11
296.0	1.67382E-10	2.67000E-11	9.11459E-11	1.58271E-11
297.0	1.67860E-10	2.69094E-11	9.15522E-11	1.59835E-11
298.0	1.68335E-10	2.71191E-11	9.19575E-11	1.61404E-11
299.0	1.68809E-10	2.73289E-11	9.23620E-11	1.62977E-11
300.0	1.69281E-10	2.75389E-11	9.27654E-11	1.64555E-11
301.0	1.69751E-10	2.77491E-11	9.31680E-11	1.66137E-11
302.0	1.70219E-10	2.79596E-11	9.35696E-11	1.67724E-11
303.0	1.70685E-10	2.81702E-11	9.39703E-11	1.69315E-11
304.0	1.71149E-10	2.83810E-11	9.43700E-11	1.70910E-11
305.0	1.71611E-10	2.85919E-11	9.47688E-11	1.72509E-11
306.0	1.72072E-10	2.88031E-11	9.51667E-11	1.74112E-11
307.0	1.72530E-10	2.90144E-11	9.55636E-11	1.75719E-11
308.0	1.72986E-10	2.92259E-11	9.59596E-11	1.77330E-11
309.0	1.73441E-10	2.94375E-11	9.63546E-11	1.78945E-11
310.0	1.73894E-10	2.96493E-11	9.67488E-11	1.80564E-11
311.0	1.74344E-10	2.98612E-11	9.71420E-11	1.82187E-11
312.0	1.74793E-10	3.00733E-11	9.75342E-11	1.83814E-11
313.0	1.75240E-10	3.02854E-11	9.79255E-11	1.85444E-11
314.0	1.75686E-10	3.04978E-11	9.83159E-11	1.87078E-11
315.0	1.76129E-10	3.07102E-11	9.87054E-11	1.88715E-11
316.0	1.76571E-10	3.09228E-11	9.90939E-11	1.90356E-11
317.0	1.77011E-10	3.11355E-11	9.94815E-11	1.92000E-11
318.0	1.77449E-10	3.13483E-11	9.98682E-11	1.93648E-11
319.0	1.77885E-10	3.15612E-11	1.00254E-10	1.95299E-11
320.0	1.78320E-10	3.17742E-11	1.00639E-10	1.96954E-11
321.0	1.78753E-10	3.19873E-11	1.01023E-10	1.98612E-11
322.0	1.79184E-10	3.22005E-11	1.01406E-10	2.00273E-11
323.0	1.79613E-10	3.24137E-11	1.01788E-10	2.01937E-11
324.0	1.80041E-10	3.26271E-11	1.02169E-10	2.03604E-11
325.0	1.80467E-10	3.28405E-11	1.02549E-10	2.05274E-11

326.0	1.80891E-10	3.30540E-11	1.02928E-10	2.06948E-11
327.0	1.81314E-10	3.32676E-11	1.03307E-10	2.08624E-11
328.0	1.81735E-10	3.34812E-11	1.03684E-10	2.10303E-11
329.0	1.82154E-10	3.36949E-11	1.04061E-10	2.11985E-11
330.0	1.82572E-10	3.39087E-11	1.04436E-10	2.13670E-11
331.0	1.82988E-10	3.41225E-11	1.04811E-10	2.15357E-11
332.0	1.83402E-10	3.43363E-11	1.05185E-10	2.17048E-11
333.0	1.83815E-10	3.45502E-11	1.05558E-10	2.18741E-11
334.0	1.84226E-10	3.47641E-11	1.05930E-10	2.20436E-11
335.0	1.84636E-10	3.49781E-11	1.06301E-10	2.22134E-11
336.0	1.85043E-10	3.51921E-11	1.06671E-10	2.23835E-11
337.0	1.85450E-10	3.54061E-11	1.07040E-10	2.25538E-11
338.0	1.85855E-10	3.56202E-11	1.07409E-10	2.27243E-11
339.0	1.86258E-10	3.58342E-11	1.07776E-10	2.28951E-11
340.0	1.86659E-10	3.60483E-11	1.08143E-10	2.30661E-11
341.0	1.87060E-10	3.62624E-11	1.08508E-10	2.32374E-11
342.0	1.87458E-10	3.64765E-11	1.08873E-10	2.34088E-11
343.0	1.87855E-10	3.66905E-11	1.09237E-10	2.35805E-11
344.0	1.88251E-10	3.69046E-11	1.09600E-10	2.37524E-11
345.0	1.88645E-10	3.71187E-11	1.09962E-10	2.39245E-11
346.0	1.89037E-10	3.73328E-11	1.10323E-10	2.40968E-11
347.0	1.89429E-10	3.75469E-11	1.10683E-10	2.42693E-11
348.0	1.89818E-10	3.77609E-11	1.11043E-10	2.44420E-11
349.0	1.90206E-10	3.79750E-11	1.11401E-10	2.46149E-11
350.0	1.90593E-10	3.81890E-11	1.11759E-10	2.47880E-11
351.0	1.90978E-10	3.84030E-11	1.12116E-10	2.49612E-11
352.0	1.91362E-10	3.86169E-11	1.12472E-10	2.51347E-11
353.0	1.91744E-10	3.88308E-11	1.12827E-10	2.53083E-11
354.0	1.92125E-10	3.90447E-11	1.13181E-10	2.54821E-11
355.0	1.92505E-10	3.92586E-11	1.13534E-10	2.56560E-11
356.0	1.92883E-10	3.94724E-11	1.13887E-10	2.58301E-11
357.0	1.93260E-10	3.96862E-11	1.14238E-10	2.60044E-11
358.0	1.93635E-10	3.98999E-11	1.14589E-10	2.61788E-11
359.0	1.94009E-10	4.01135E-11	1.14939E-10	2.63534E-11
360.0	1.94381E-10	4.03271E-11	1.15288E-10	2.65281E-11
361.0	1.94753E-10	4.05407E-11	1.15636E-10	2.67029E-11
362.0	1.95123E-10	4.07542E-11	1.15983E-10	2.68779E-11
363.0	1.95491E-10	4.09676E-11	1.16329E-10	2.70530E-11
364.0	1.95858E-10	4.11810E-11	1.16675E-10	2.72283E-11
365.0	1.96224E-10	4.13943E-11	1.17020E-10	2.74036E-11
366.0	1.96589E-10	4.16075E-11	1.17363E-10	2.75791E-11
367.0	1.96952E-10	4.18206E-11	1.17706E-10	2.77547E-11
368.0	1.97314E-10	4.20337E-11	1.18048E-10	2.79304E-11
369.0	1.97674E-10	4.22467E-11	1.18390E-10	2.81063E-11
370.0	1.98034E-10	4.24596E-11	1.18730E-10	2.82822E-11
371.0	1.98392E-10	4.26724E-11	1.19070E-10	2.84582E-11
372.0	1.98749E-10	4.28851E-11	1.19408E-10	2.86343E-11
373.0	1.99104E-10	4.30977E-11	1.19746E-10	2.88106E-11
374.0	1.99458E-10	4.33103E-11	1.20083E-10	2.89869E-11
375.0	1.99811E-10	4.35227E-11	1.20420E-10	2.91633E-11

376.0	2.00163E-10	4.37351E-11	1.20755E-10	2.93397E-11
377.0	2.00514E-10	4.39473E-11	1.21090E-10	2.95163E-11
378.0	2.00863E-10	4.41594E-11	1.21423E-10	2.96929E-11
379.0	2.01211E-10	4.43715E-11	1.21756E-10	2.98696E-11
380.0	2.01558E-10	4.45834E-11	1.22088E-10	3.00464E-11
381.0	2.01904E-10	4.47952E-11	1.22420E-10	3.02232E-11
382.0	2.02248E-10	4.50069E-11	1.22750E-10	3.04001E-11
383.0	2.02591E-10	4.52184E-11	1.23080E-10	3.05771E-11
384.0	2.02933E-10	4.54299E-11	1.23409E-10	3.07541E-11
385.0	2.03274E-10	4.56412E-11	1.23737E-10	3.09312E-11
386.0	2.03614E-10	4.58524E-11	1.24064E-10	3.11083E-11
387.0	2.03953E-10	4.60635E-11	1.24390E-10	3.12854E-11
388.0	2.04290E-10	4.62745E-11	1.24716E-10	3.14626E-11
389.0	2.04626E-10	4.64853E-11	1.25040E-10	3.16399E-11
390.0	2.04961E-10	4.66960E-11	1.25364E-10	3.18171E-11
391.0	2.05295E-10	4.69065E-11	1.25688E-10	3.19944E-11
392.0	2.05628E-10	4.71170E-11	1.26010E-10	3.21717E-11
393.0	2.05960E-10	4.73272E-11	1.26331E-10	3.23491E-11
394.0	2.06291E-10	4.75374E-11	1.26652E-10	3.25265E-11
395.0	2.06620E-10	4.77474E-11	1.26972E-10	3.27038E-11
396.0	2.06949E-10	4.79572E-11	1.27291E-10	3.28812E-11
397.0	2.07276E-10	4.81669E-11	1.27610E-10	3.30587E-11
398.0	2.07602E-10	4.83765E-11	1.27927E-10	3.32361E-11
399.0	2.07927E-10	4.85859E-11	1.28244E-10	3.34135E-11
400.0	2.08251E-10	4.87951E-11	1.28560E-10	3.35910E-11
401.0	2.08574E-10	4.90042E-11	1.28875E-10	3.37684E-11
402.0	2.08896E-10	4.92131E-11	1.29190E-10	3.39458E-11
403.0	2.09217E-10	4.94219E-11	1.29504E-10	3.41232E-11
404.0	2.09537E-10	4.96305E-11	1.29816E-10	3.43007E-11
405.0	2.09856E-10	4.98390E-11	1.30129E-10	3.44781E-11
406.0	2.10173E-10	5.00472E-11	1.30440E-10	3.46555E-11
407.0	2.10490E-10	5.02554E-11	1.30751E-10	3.48328E-11
408.0	2.10806E-10	5.04633E-11	1.31060E-10	3.50102E-11
409.0	2.11120E-10	5.06711E-11	1.31369E-10	3.51875E-11
410.0	2.11434E-10	5.08787E-11	1.31678E-10	3.53648E-11
411.0	2.11746E-10	5.10861E-11	1.31985E-10	3.55421E-11
412.0	2.12058E-10	5.12934E-11	1.32292E-10	3.57193E-11
413.0	2.12368E-10	5.15004E-11	1.32598E-10	3.58965E-11
414.0	2.12678E-10	5.17073E-11	1.32903E-10	3.60737E-11
415.0	2.12987E-10	5.19141E-11	1.33208E-10	3.62509E-11
416.0	2.13294E-10	5.21206E-11	1.33511E-10	3.64279E-11
417.0	2.13601E-10	5.23270E-11	1.33814E-10	3.66050E-11
418.0	2.13907E-10	5.25331E-11	1.34117E-10	3.67820E-11
419.0	2.14211E-10	5.27391E-11	1.34418E-10	3.69589E-11
420.0	2.14515E-10	5.29449E-11	1.34719E-10	3.71358E-11
421.0	2.14818E-10	5.31505E-11	1.35019E-10	3.73127E-11
422.0	2.15119E-10	5.33559E-11	1.35318E-10	3.74895E-11
423.0	2.15420E-10	5.35611E-11	1.35617E-10	3.76662E-11
424.0	2.15720E-10	5.37661E-11	1.35914E-10	3.78429E-11
425.0	2.16019E-10	5.39710E-11	1.36211E-10	3.80195E-11

426.0	2.16317E-10	5.41756E-11	1.36508E-10	3.81960E-11
427.0	2.16614E-10	5.43800E-11	1.36803E-10	3.83725E-11
428.0	2.16911E-10	5.45843E-11	1.37098E-10	3.85489E-11
429.0	2.17206E-10	5.47883E-11	1.37392E-10	3.87252E-11
430.0	2.17500E-10	5.49921E-11	1.37686E-10	3.89014E-11
431.0	2.17794E-10	5.51957E-11	1.37978E-10	3.90776E-11
432.0	2.18086E-10	5.53992E-11	1.38270E-10	3.92537E-11
433.0	2.18378E-10	5.56024E-11	1.38561E-10	3.94297E-11
434.0	2.18669E-10	5.58054E-11	1.38852E-10	3.96056E-11
435.0	2.18958E-10	5.60082E-11	1.39142E-10	3.97814E-11
436.0	2.19247E-10	5.62107E-11	1.39431E-10	3.99571E-11
437.0	2.19535E-10	5.64131E-11	1.39719E-10	4.01328E-11
438.0	2.19823E-10	5.66152E-11	1.40007E-10	4.03083E-11
439.0	2.20109E-10	5.68172E-11	1.40294E-10	4.04838E-11
440.0	2.20395E-10	5.70189E-11	1.40580E-10	4.06591E-11
441.0	2.20679E-10	5.72204E-11	1.40865E-10	4.08344E-11
442.0	2.20963E-10	5.74217E-11	1.41150E-10	4.10095E-11
443.0	2.21246E-10	5.76228E-11	1.41434E-10	4.11846E-11
444.0	2.21528E-10	5.78236E-11	1.41718E-10	4.13595E-11
445.0	2.21809E-10	5.80242E-11	1.42000E-10	4.15343E-11
446.0	2.22089E-10	5.82246E-11	1.42282E-10	4.17091E-11
447.0	2.22369E-10	5.84248E-11	1.42563E-10	4.18837E-11
448.0	2.22648E-10	5.86248E-11	1.42844E-10	4.20581E-11
449.0	2.22926E-10	5.88245E-11	1.43124E-10	4.22325E-11
450.0	2.23203E-10	5.90240E-11	1.43403E-10	4.24068E-11
451.0	2.23479E-10	5.92233E-11	1.43682E-10	4.25809E-11
452.0	2.23754E-10	5.94223E-11	1.43959E-10	4.27549E-11
453.0	2.24029E-10	5.96211E-11	1.44236E-10	4.29288E-11
454.0	2.24303E-10	5.98197E-11	1.44513E-10	4.31026E-11
455.0	2.24576E-10	6.00180E-11	1.44789E-10	4.32762E-11
456.0	2.24848E-10	6.02161E-11	1.45064E-10	4.34497E-11
457.0	2.25119E-10	6.04140E-11	1.45338E-10	4.36231E-11
458.0	2.25390E-10	6.06117E-11	1.45612E-10	4.37963E-11
459.0	2.25660E-10	6.08091E-11	1.45885E-10	4.39694E-11
460.0	2.25929E-10	6.10062E-11	1.46157E-10	4.41423E-11
461.0	2.26197E-10	6.12032E-11	1.46429E-10	4.43152E-11
462.0	2.26464E-10	6.13998E-11	1.46700E-10	4.44878E-11
463.0	2.26731E-10	6.15963E-11	1.46970E-10	4.46604E-11
464.0	2.26997E-10	6.17925E-11	1.47240E-10	4.48328E-11
465.0	2.27262E-10	6.19885E-11	1.47509E-10	4.50050E-11
466.0	2.27526E-10	6.21842E-11	1.47777E-10	4.51771E-11
467.0	2.27790E-10	6.23797E-11	1.48045E-10	4.53491E-11
468.0	2.28053E-10	6.25749E-11	1.48312E-10	4.55209E-11
469.0	2.28315E-10	6.27699E-11	1.48578E-10	4.56925E-11
470.0	2.28576E-10	6.29646E-11	1.48844E-10	4.58640E-11
471.0	2.28837E-10	6.31591E-11	1.49109E-10	4.60353E-11
472.0	2.29097E-10	6.33534E-11	1.49373E-10	4.62065E-11
473.0	2.29356E-10	6.35474E-11	1.49637E-10	4.63775E-11
474.0	2.29614E-10	6.37411E-11	1.49900E-10	4.65484E-11
475.0	2.29872E-10	6.39346E-11	1.50162E-10	4.67191E-11

476.0	2.30129E-10	6.41278E-11	1.50424E-10	4.68896E-11
477.0	2.30385E-10	6.43208E-11	1.50685E-10	4.70600E-11
478.0	2.30641E-10	6.45136E-11	1.50945E-10	4.72302E-11
479.0	2.30895E-10	6.47061E-11	1.51205E-10	4.74002E-11
480.0	2.31150E-10	6.48983E-11	1.51464E-10	4.75701E-11
481.0	2.31403E-10	6.50903E-11	1.51723E-10	4.77398E-11
482.0	2.31656E-10	6.52820E-11	1.51980E-10	4.79093E-11
483.0	2.31907E-10	6.54734E-11	1.52238E-10	4.80787E-11
484.0	2.32159E-10	6.56646E-11	1.52494E-10	4.82478E-11
485.0	2.32409E-10	6.58556E-11	1.52750E-10	4.84168E-11
486.0	2.32659E-10	6.60463E-11	1.53005E-10	4.85857E-11
487.0	2.32908E-10	6.62367E-11	1.53260E-10	4.87543E-11
488.0	2.33157E-10	6.64269E-11	1.53514E-10	4.89228E-11
489.0	2.33404E-10	6.66168E-11	1.53767E-10	4.90910E-11
490.0	2.33651E-10	6.68064E-11	1.54020E-10	4.92591E-11
491.0	2.33898E-10	6.69958E-11	1.54272E-10	4.94270E-11
492.0	2.34143E-10	6.71849E-11	1.54524E-10	4.95948E-11
493.0	2.34388E-10	6.73738E-11	1.54774E-10	4.97623E-11
494.0	2.34633E-10	6.75624E-11	1.55025E-10	4.99296E-11
495.0	2.34876E-10	6.77507E-11	1.55274E-10	5.00968E-11
496.0	2.35119E-10	6.79388E-11	1.55523E-10	5.02638E-11
497.0	2.35362E-10	6.81266E-11	1.55772E-10	5.04305E-11
498.0	2.35603E-10	6.83141E-11	1.56019E-10	5.05971E-11
499.0	2.35844E-10	6.85014E-11	1.56266E-10	5.07635E-11
500.0	2.36085E-10	6.86884E-11	1.56513E-10	5.09297E-11

Table S6: Computed de-excitation rate coefficients in cm^3 $molecule^{-1}$ s^{-1} units for a series of inelastic processes generated using the 4D RR-PES for the $HeH^+(j_1)\cdot\cdot\cdot$ para-$H_2(j_2 = 0)$ system with $\Delta j_1 = -1$ and $\Delta j_1 = -2$.

T (K)	10->00	20->00	20->10	30->10
5.0	3.33744E-10	5.19083E-11	1.63160E-10	6.87848E-11
6.0	3.04043E-10	6.10786E-11	1.93220E-10	7.69219E-11
7.0	2.83866E-10	6.79987E-11	2.16498E-10	8.34042E-11
8.0	2.71405E-10	7.36215E-11	2.36004E-10	8.93002E-11
9.0	2.64390E-10	7.85089E-11	2.53420E-10	9.50286E-11
10.0	2.60950E-10	8.29401E-11	2.69518E-10	1.00670E-10
11.0	2.59745E-10	8.70410E-11	2.84590E-10	1.06176E-10
12.0	2.59874E-10	9.08652E-11	2.98729E-10	1.11470E-10
13.0	2.60758E-10	9.44379E-11	3.11961E-10	1.16490E-10
14.0	2.62034E-10	9.77745E-11	3.24300E-10	1.21197E-10
15.0	2.63481E-10	1.00889E-10	3.35770E-10	1.25573E-10
16.0	2.64969E-10	1.03795E-10	3.46411E-10	1.29616E-10
17.0	2.66426E-10	1.06508E-10	3.56268E-10	1.33338E-10
18.0	2.67814E-10	1.09043E-10	3.65394E-10	1.36755E-10
19.0	2.69117E-10	1.11416E-10	3.73846E-10	1.39887E-10
20.0	2.70331E-10	1.13642E-10	3.81678E-10	1.42758E-10
21.0	2.71459E-10	1.15732E-10	3.88942E-10	1.45388E-10
22.0	2.72507E-10	1.17700E-10	3.95688E-10	1.47800E-10
23.0	2.73483E-10	1.19557E-10	4.01962E-10	1.50014E-10
24.0	2.74394E-10	1.21313E-10	4.07804E-10	1.52047E-10

25.0	2.75249E-10	1.22976E-10	4.13252E-10	1.53918E-10
26.0	2.76053E-10	1.24554E-10	4.18341E-10	1.55642E-10
27.0	2.76814E-10	1.26054E-10	4.23101E-10	1.57231E-10
28.0	2.77536E-10	1.27483E-10	4.27560E-10	1.58700E-10
29.0	2.78223E-10	1.28844E-10	4.31742E-10	1.60058E-10
30.0	2.78880E-10	1.30144E-10	4.35669E-10	1.61316E-10
31.0	2.79508E-10	1.31386E-10	4.39361E-10	1.62483E-10
32.0	2.80111E-10	1.32574E-10	4.42836E-10	1.63566E-10
33.0	2.80689E-10	1.33711E-10	4.46110E-10	1.64573E-10
34.0	2.81246E-10	1.34800E-10	4.49197E-10	1.65510E-10
35.0	2.81781E-10	1.35844E-10	4.52111E-10	1.66383E-10
36.0	2.82297E-10	1.36846E-10	4.54864E-10	1.67197E-10
37.0	2.82794E-10	1.37807E-10	4.57466E-10	1.67956E-10
38.0	2.83272E-10	1.38730E-10	4.59926E-10	1.68665E-10
39.0	2.83732E-10	1.39616E-10	4.62254E-10	1.69327E-10
40.0	2.84175E-10	1.40467E-10	4.64458E-10	1.69946E-10
41.0	2.84600E-10	1.41285E-10	4.66546E-10	1.70525E-10
42.0	2.85010E-10	1.42071E-10	4.68523E-10	1.71066E-10
43.0	2.85403E-10	1.42827E-10	4.70397E-10	1.71573E-10
44.0	2.85780E-10	1.43553E-10	4.72172E-10	1.72047E-10
45.0	2.86142E-10	1.44252E-10	4.73855E-10	1.72491E-10
46.0	2.86488E-10	1.44923E-10	4.75451E-10	1.72907E-10
47.0	2.86819E-10	1.45569E-10	4.76962E-10	1.73296E-10
48.0	2.87136E-10	1.46190E-10	4.78395E-10	1.73660E-10
49.0	2.87439E-10	1.46787E-10	4.79752E-10	1.74001E-10
50.0	2.87728E-10	1.47361E-10	4.81038E-10	1.74320E-10
51.0	2.88003E-10	1.47913E-10	4.82256E-10	1.74618E-10
52.0	2.88265E-10	1.48443E-10	4.83408E-10	1.74896E-10
53.0	2.88514E-10	1.48954E-10	4.84498E-10	1.75156E-10
54.0	2.88751E-10	1.49444E-10	4.85529E-10	1.75399E-10
55.0	2.88975E-10	1.49915E-10	4.86504E-10	1.75626E-10
56.0	2.89188E-10	1.50368E-10	4.87424E-10	1.75836E-10
57.0	2.89389E-10	1.50803E-10	4.88291E-10	1.76032E-10
58.0	2.89579E-10	1.51221E-10	4.89109E-10	1.76215E-10
59.0	2.89758E-10	1.51623E-10	4.89880E-10	1.76384E-10
60.0	2.89927E-10	1.52008E-10	4.90604E-10	1.76540E-10
61.0	2.90086E-10	1.52379E-10	4.91285E-10	1.76685E-10
62.0	2.90235E-10	1.52734E-10	4.91923E-10	1.76818E-10
63.0	2.90374E-10	1.53075E-10	4.92520E-10	1.76941E-10
64.0	2.90505E-10	1.53402E-10	4.93079E-10	1.77053E-10
65.0	2.90627E-10	1.53715E-10	4.93599E-10	1.77156E-10
66.0	2.90741E-10	1.54016E-10	4.94084E-10	1.77249E-10
67.0	2.90847E-10	1.54303E-10	4.94534E-10	1.77334E-10
68.0	2.90945E-10	1.54579E-10	4.94951E-10	1.77411E-10
69.0	2.91035E-10	1.54843E-10	4.95335E-10	1.77479E-10
70.0	2.91119E-10	1.55096E-10	4.95688E-10	1.77540E-10
71.0	2.91195E-10	1.55337E-10	4.96011E-10	1.77593E-10
72.0	2.91266E-10	1.55568E-10	4.96306E-10	1.77640E-10
73.0	2.91329E-10	1.55788E-10	4.96573E-10	1.77680E-10
74.0	2.91387E-10	1.55999E-10	4.96812E-10	1.77713E-10

75.0	2.91440E-10	1.56199E-10	4.97026E-10	1.77741E-10
76.0	2.91486E-10	1.56390E-10	4.97215E-10	1.77763E-10
77.0	2.91528E-10	1.56573E-10	4.97380E-10	1.77779E-10
78.0	2.91564E-10	1.56746E-10	4.97522E-10	1.77790E-10
79.0	2.91596E-10	1.56911E-10	4.97641E-10	1.77796E-10
80.0	2.91623E-10	1.57067E-10	4.97739E-10	1.77797E-10
81.0	2.91646E-10	1.57215E-10	4.97815E-10	1.77793E-10
82.0	2.91665E-10	1.57356E-10	4.97872E-10	1.77785E-10
83.0	2.91680E-10	1.57489E-10	4.97909E-10	1.77772E-10
84.0	2.91691E-10	1.57615E-10	4.97928E-10	1.77756E-10
85.0	2.91698E-10	1.57733E-10	4.97928E-10	1.77736E-10
86.0	2.91702E-10	1.57845E-10	4.97911E-10	1.77711E-10
87.0	2.91703E-10	1.57950E-10	4.97876E-10	1.77684E-10
88.0	2.91701E-10	1.58048E-10	4.97826E-10	1.77652E-10
89.0	2.91696E-10	1.58140E-10	4.97760E-10	1.77618E-10
90.0	2.91688E-10	1.58226E-10	4.97678E-10	1.77580E-10
91.0	2.91678E-10	1.58306E-10	4.97582E-10	1.77539E-10
92.0	2.91665E-10	1.58380E-10	4.97472E-10	1.77496E-10
93.0	2.91650E-10	1.58449E-10	4.97348E-10	1.77449E-10
94.0	2.91633E-10	1.58512E-10	4.97210E-10	1.77400E-10
95.0	2.91614E-10	1.58570E-10	4.97060E-10	1.77348E-10
96.0	2.91592E-10	1.58622E-10	4.96898E-10	1.77294E-10
97.0	2.91569E-10	1.58670E-10	4.96724E-10	1.77237E-10
98.0	2.91544E-10	1.58713E-10	4.96538E-10	1.77178E-10
99.0	2.91518E-10	1.58751E-10	4.96341E-10	1.77116E-10
100.0	2.91490E-10	1.58785E-10	4.96134E-10	1.77053E-10
101.0	2.91461E-10	1.58814E-10	4.95916E-10	1.76987E-10
102.0	2.91430E-10	1.58839E-10	4.95688E-10	1.76920E-10
103.0	2.91398E-10	1.58859E-10	4.95451E-10	1.76851E-10
104.0	2.91365E-10	1.58876E-10	4.95204E-10	1.76779E-10
105.0	2.91331E-10	1.58889E-10	4.94948E-10	1.76706E-10
106.0	2.91296E-10	1.58897E-10	4.94684E-10	1.76632E-10
107.0	2.91260E-10	1.58902E-10	4.94412E-10	1.76556E-10
108.0	2.91223E-10	1.58904E-10	4.94131E-10	1.76478E-10
109.0	2.91185E-10	1.58901E-10	4.93843E-10	1.76398E-10
110.0	2.91147E-10	1.58896E-10	4.93547E-10	1.76318E-10
111.0	2.91107E-10	1.58887E-10	4.93245E-10	1.76236E-10
112.0	2.91068E-10	1.58875E-10	4.92935E-10	1.76152E-10
113.0	2.91028E-10	1.58859E-10	4.92619E-10	1.76067E-10
114.0	2.90987E-10	1.58841E-10	4.92296E-10	1.75982E-10
115.0	2.90946E-10	1.58820E-10	4.91967E-10	1.75894E-10
116.0	2.90904E-10	1.58795E-10	4.91633E-10	1.75806E-10
117.0	2.90862E-10	1.58768E-10	4.91292E-10	1.75717E-10
118.0	2.90820E-10	1.58738E-10	4.90946E-10	1.75626E-10
119.0	2.90778E-10	1.58706E-10	4.90595E-10	1.75535E-10
120.0	2.90735E-10	1.58671E-10	4.90239E-10	1.75443E-10
121.0	2.90692E-10	1.58633E-10	4.89878E-10	1.75350E-10
122.0	2.90649E-10	1.58593E-10	4.89513E-10	1.75256E-10
123.0	2.90606E-10	1.58551E-10	4.89143E-10	1.75161E-10
124.0	2.90563E-10	1.58506E-10	4.88769E-10	1.75065E-10

125.0	2.90520E-10	1.58459E-10	4.88390E-10	1.74968E-10
126.0	2.90477E-10	1.58410E-10	4.88008E-10	1.74871E-10
127.0	2.90433E-10	1.58358E-10	4.87622E-10	1.74773E-10
128.0	2.90390E-10	1.58305E-10	4.87233E-10	1.74674E-10
129.0	2.90347E-10	1.58250E-10	4.86840E-10	1.74575E-10
130.0	2.90304E-10	1.58192E-10	4.86444E-10	1.74475E-10
131.0	2.90261E-10	1.58133E-10	4.86044E-10	1.74375E-10
132.0	2.90219E-10	1.58072E-10	4.85642E-10	1.74274E-10
133.0	2.90176E-10	1.58009E-10	4.85237E-10	1.74172E-10
134.0	2.90134E-10	1.57945E-10	4.84829E-10	1.74070E-10
135.0	2.90092E-10	1.57879E-10	4.84419E-10	1.73967E-10
136.0	2.90050E-10	1.57811E-10	4.84006E-10	1.73864E-10
137.0	2.90008E-10	1.57741E-10	4.83591E-10	1.73761E-10
138.0	2.89967E-10	1.57670E-10	4.83173E-10	1.73657E-10
139.0	2.89926E-10	1.57598E-10	4.82754E-10	1.73553E-10
140.0	2.89885E-10	1.57524E-10	4.82333E-10	1.73448E-10
141.0	2.89845E-10	1.57449E-10	4.81910E-10	1.73343E-10
142.0	2.89805E-10	1.57372E-10	4.81485E-10	1.73238E-10
143.0	2.89765E-10	1.57294E-10	4.81058E-10	1.73132E-10
144.0	2.89725E-10	1.57214E-10	4.80630E-10	1.73026E-10
145.0	2.89686E-10	1.57134E-10	4.80201E-10	1.72920E-10
146.0	2.89647E-10	1.57052E-10	4.79770E-10	1.72814E-10
147.0	2.89609E-10	1.56969E-10	4.79338E-10	1.72707E-10
148.0	2.89571E-10	1.56885E-10	4.78905E-10	1.72600E-10
149.0	2.89533E-10	1.56800E-10	4.78471E-10	1.72493E-10
150.0	2.89496E-10	1.56713E-10	4.78036E-10	1.72386E-10
151.0	2.89459E-10	1.56626E-10	4.77600E-10	1.72278E-10
152.0	2.89422E-10	1.56537E-10	4.77163E-10	1.72170E-10
153.0	2.89386E-10	1.56448E-10	4.76725E-10	1.72063E-10
154.0	2.89351E-10	1.56358E-10	4.76287E-10	1.71955E-10
155.0	2.89316E-10	1.56266E-10	4.75848E-10	1.71847E-10
156.0	2.89281E-10	1.56174E-10	4.75409E-10	1.71739E-10
157.0	2.89246E-10	1.56081E-10	4.74969E-10	1.71630E-10
158.0	2.89212E-10	1.55987E-10	4.74529E-10	1.71522E-10
159.0	2.89179E-10	1.55893E-10	4.74088E-10	1.71414E-10
160.0	2.89146E-10	1.55797E-10	4.73647E-10	1.71305E-10
161.0	2.89113E-10	1.55701E-10	4.73206E-10	1.71197E-10
162.0	2.89081E-10	1.55604E-10	4.72765E-10	1.71088E-10
163.0	2.89050E-10	1.55506E-10	4.72324E-10	1.70980E-10
164.0	2.89019E-10	1.55408E-10	4.71883E-10	1.70871E-10
165.0	2.88988E-10	1.55309E-10	4.71442E-10	1.70763E-10
166.0	2.88957E-10	1.55209E-10	4.71001E-10	1.70654E-10
167.0	2.88928E-10	1.55109E-10	4.70560E-10	1.70546E-10
168.0	2.88898E-10	1.55008E-10	4.70119E-10	1.70437E-10
169.0	2.88869E-10	1.54907E-10	4.69679E-10	1.70329E-10
170.0	2.88841E-10	1.54805E-10	4.69239E-10	1.70220E-10
171.0	2.88813E-10	1.54702E-10	4.68799E-10	1.70112E-10
172.0	2.88785E-10	1.54599E-10	4.68359E-10	1.70004E-10
173.0	2.88758E-10	1.54496E-10	4.67920E-10	1.69896E-10
174.0	2.88732E-10	1.54391E-10	4.67482E-10	1.69787E-10

175.0	2.88706E-10	1.54287E-10	4.67044E-10	1.69679E-10
176.0	2.88680E-10	1.54182E-10	4.66606E-10	1.69571E-10
177.0	2.88655E-10	1.54077E-10	4.66169E-10	1.69464E-10
178.0	2.88630E-10	1.53971E-10	4.65733E-10	1.69356E-10
179.0	2.88606E-10	1.53865E-10	4.65297E-10	1.69248E-10
180.0	2.88582E-10	1.53758E-10	4.64862E-10	1.69141E-10
181.0	2.88559E-10	1.53651E-10	4.64428E-10	1.69033E-10
182.0	2.88536E-10	1.53544E-10	4.63994E-10	1.68926E-10
183.0	2.88513E-10	1.53437E-10	4.63561E-10	1.68819E-10
184.0	2.88491E-10	1.53329E-10	4.63130E-10	1.68712E-10
185.0	2.88470E-10	1.53220E-10	4.62698E-10	1.68605E-10
186.0	2.88449E-10	1.53112E-10	4.62268E-10	1.68499E-10
187.0	2.88428E-10	1.53003E-10	4.61839E-10	1.68392E-10
188.0	2.88408E-10	1.52894E-10	4.61410E-10	1.68286E-10
189.0	2.88388E-10	1.52785E-10	4.60983E-10	1.68180E-10
190.0	2.88369E-10	1.52676E-10	4.60556E-10	1.68074E-10
191.0	2.88350E-10	1.52566E-10	4.60130E-10	1.67968E-10
192.0	2.88332E-10	1.52456E-10	4.59706E-10	1.67862E-10
193.0	2.88314E-10	1.52346E-10	4.59282E-10	1.67757E-10
194.0	2.88296E-10	1.52236E-10	4.58860E-10	1.67652E-10
195.0	2.88279E-10	1.52125E-10	4.58438E-10	1.67547E-10
196.0	2.88262E-10	1.52015E-10	4.58018E-10	1.67442E-10
197.0	2.88246E-10	1.51904E-10	4.57599E-10	1.67337E-10
198.0	2.88230E-10	1.51793E-10	4.57181E-10	1.67233E-10
199.0	2.88215E-10	1.51682E-10	4.56764E-10	1.67128E-10
200.0	2.88199E-10	1.51571E-10	4.56348E-10	1.67025E-10
201.0	2.88185E-10	1.51459E-10	4.55933E-10	1.66921E-10
202.0	2.88171E-10	1.51348E-10	4.55520E-10	1.66817E-10
203.0	2.88157E-10	1.51237E-10	4.55108E-10	1.66714E-10
204.0	2.88143E-10	1.51125E-10	4.54697E-10	1.66611E-10
205.0	2.88130E-10	1.51013E-10	4.54287E-10	1.66508E-10
206.0	2.88118E-10	1.50902E-10	4.53878E-10	1.66405E-10
207.0	2.88106E-10	1.50790E-10	4.53471E-10	1.66303E-10
208.0	2.88094E-10	1.50678E-10	4.53065E-10	1.66201E-10
209.0	2.88082E-10	1.50566E-10	4.52661E-10	1.66099E-10
210.0	2.88071E-10	1.50455E-10	4.52257E-10	1.65997E-10
211.0	2.88061E-10	1.50343E-10	4.51855E-10	1.65896E-10
212.0	2.88050E-10	1.50231E-10	4.51455E-10	1.65795E-10
213.0	2.88041E-10	1.50119E-10	4.51055E-10	1.65694E-10
214.0	2.88031E-10	1.50007E-10	4.50657E-10	1.65593E-10
215.0	2.88022E-10	1.49895E-10	4.50260E-10	1.65493E-10
216.0	2.88013E-10	1.49783E-10	4.49865E-10	1.65393E-10
217.0	2.88005E-10	1.49671E-10	4.49471E-10	1.65293E-10
218.0	2.87997E-10	1.49560E-10	4.49079E-10	1.65193E-10
219.0	2.87989E-10	1.49448E-10	4.48687E-10	1.65094E-10
220.0	2.87982E-10	1.49336E-10	4.48298E-10	1.64995E-10
221.0	2.87975E-10	1.49224E-10	4.47909E-10	1.64896E-10
222.0	2.87968E-10	1.49113E-10	4.47522E-10	1.64797E-10
223.0	2.87962E-10	1.49001E-10	4.47137E-10	1.64699E-10
224.0	2.87956E-10	1.48890E-10	4.46752E-10	1.64601E-10

225.0	2.87950E-10	1.48778E-10	4.46370E-10	1.64503E-10
226.0	2.87945E-10	1.48667E-10	4.45988E-10	1.64406E-10
227.0	2.87940E-10	1.48555E-10	4.45609E-10	1.64308E-10
228.0	2.87936E-10	1.48444E-10	4.45230E-10	1.64212E-10
229.0	2.87931E-10	1.48333E-10	4.44853E-10	1.64115E-10
230.0	2.87927E-10	1.48222E-10	4.44477E-10	1.64019E-10
231.0	2.87924E-10	1.48111E-10	4.44103E-10	1.63922E-10
232.0	2.87921E-10	1.48000E-10	4.43731E-10	1.63827E-10
233.0	2.87918E-10	1.47889E-10	4.43359E-10	1.63731E-10
234.0	2.87915E-10	1.47779E-10	4.42990E-10	1.63636E-10
235.0	2.87913E-10	1.47668E-10	4.42621E-10	1.63541E-10
236.0	2.87911E-10	1.47558E-10	4.42255E-10	1.63446E-10
237.0	2.87909E-10	1.47448E-10	4.41889E-10	1.63352E-10
238.0	2.87908E-10	1.47337E-10	4.41525E-10	1.63258E-10
239.0	2.87906E-10	1.47227E-10	4.41163E-10	1.63164E-10
240.0	2.87906E-10	1.47118E-10	4.40802E-10	1.63070E-10
241.0	2.87905E-10	1.47008E-10	4.40442E-10	1.62977E-10
242.0	2.87905E-10	1.46898E-10	4.40084E-10	1.62884E-10
243.0	2.87905E-10	1.46789E-10	4.39728E-10	1.62792E-10
244.0	2.87905E-10	1.46680E-10	4.39373E-10	1.62699E-10
245.0	2.87906E-10	1.46570E-10	4.39019E-10	1.62607E-10
246.0	2.87907E-10	1.46461E-10	4.38667E-10	1.62515E-10
247.0	2.87908E-10	1.46353E-10	4.38316E-10	1.62424E-10
248.0	2.87909E-10	1.46244E-10	4.37967E-10	1.62333E-10
249.0	2.87911E-10	1.46135E-10	4.37619E-10	1.62242E-10
250.0	2.87913E-10	1.46027E-10	4.37273E-10	1.62151E-10
251.0	2.87915E-10	1.45919E-10	4.36928E-10	1.62061E-10
252.0	2.87918E-10	1.45811E-10	4.36585E-10	1.61971E-10
253.0	2.87921E-10	1.45703E-10	4.36243E-10	1.61881E-10
254.0	2.87924E-10	1.45596E-10	4.35903E-10	1.61791E-10
255.0	2.87927E-10	1.45488E-10	4.35564E-10	1.61702E-10
256.0	2.87931E-10	1.45381E-10	4.35226E-10	1.61613E-10
257.0	2.87934E-10	1.45274E-10	4.34890E-10	1.61525E-10
258.0	2.87939E-10	1.45167E-10	4.34556E-10	1.61436E-10
259.0	2.87943E-10	1.45060E-10	4.34223E-10	1.61348E-10
260.0	2.87947E-10	1.44954E-10	4.33891E-10	1.61261E-10
261.0	2.87952E-10	1.44848E-10	4.33561E-10	1.61173E-10
262.0	2.87957E-10	1.44742E-10	4.33232E-10	1.61086E-10
263.0	2.87963E-10	1.44636E-10	4.32905E-10	1.60999E-10
264.0	2.87968E-10	1.44530E-10	4.32579E-10	1.60912E-10
265.0	2.87974E-10	1.44425E-10	4.32255E-10	1.60826E-10
266.0	2.87980E-10	1.44319E-10	4.31932E-10	1.60740E-10
267.0	2.87986E-10	1.44214E-10	4.31610E-10	1.60654E-10
268.0	2.87992E-10	1.44109E-10	4.31290E-10	1.60569E-10
269.0	2.87999E-10	1.44005E-10	4.30972E-10	1.60484E-10
270.0	2.88006E-10	1.43900E-10	4.30655E-10	1.60399E-10
271.0	2.88013E-10	1.43796E-10	4.30339E-10	1.60315E-10
272.0	2.88020E-10	1.43692E-10	4.30025E-10	1.60230E-10
273.0	2.88028E-10	1.43588E-10	4.29712E-10	1.60146E-10
274.0	2.88035E-10	1.43485E-10	4.29400E-10	1.60063E-10

275.0	2.88043E-10	1.43382E-10	4.29090E-10	1.59979E-10
276.0	2.88051E-10	1.43278E-10	4.28782E-10	1.59896E-10
277.0	2.88060E-10	1.43176E-10	4.28474E-10	1.59813E-10
278.0	2.88068E-10	1.43073E-10	4.28169E-10	1.59731E-10
279.0	2.88077E-10	1.42970E-10	4.27864E-10	1.59648E-10
280.0	2.88086E-10	1.42868E-10	4.27561E-10	1.59566E-10
281.0	2.88095E-10	1.42766E-10	4.27260E-10	1.59485E-10
282.0	2.88104E-10	1.42665E-10	4.26959E-10	1.59403E-10
283.0	2.88114E-10	1.42563E-10	4.26661E-10	1.59322E-10
284.0	2.88123E-10	1.42462E-10	4.26363E-10	1.59241E-10
285.0	2.88133E-10	1.42361E-10	4.26067E-10	1.59161E-10
286.0	2.88143E-10	1.42260E-10	4.25772E-10	1.59080E-10
287.0	2.88154E-10	1.42159E-10	4.25479E-10	1.59000E-10
288.0	2.88164E-10	1.42059E-10	4.25187E-10	1.58921E-10
289.0	2.88175E-10	1.41959E-10	4.24897E-10	1.58841E-10
290.0	2.88185E-10	1.41859E-10	4.24607E-10	1.58762E-10
291.0	2.88196E-10	1.41759E-10	4.24320E-10	1.58683E-10
292.0	2.88208E-10	1.41660E-10	4.24033E-10	1.58605E-10
293.0	2.88219E-10	1.41561E-10	4.23748E-10	1.58526E-10
294.0	2.88230E-10	1.41462E-10	4.23464E-10	1.58448E-10
295.0	2.88242E-10	1.41363E-10	4.23182E-10	1.58370E-10
296.0	2.88254E-10	1.41265E-10	4.22901E-10	1.58293E-10
297.0	2.88266E-10	1.41166E-10	4.22621E-10	1.58216E-10
298.0	2.88278E-10	1.41069E-10	4.22342E-10	1.58139E-10
299.0	2.88290E-10	1.40971E-10	4.22065E-10	1.58062E-10
300.0	2.88302E-10	1.40873E-10	4.21789E-10	1.57986E-10
301.0	2.88315E-10	1.40776E-10	4.21515E-10	1.57909E-10
302.0	2.88328E-10	1.40679E-10	4.21242E-10	1.57834E-10
303.0	2.88341E-10	1.40582E-10	4.20970E-10	1.57758E-10
304.0	2.88354E-10	1.40486E-10	4.20699E-10	1.57683E-10
305.0	2.88367E-10	1.40390E-10	4.20430E-10	1.57608E-10
306.0	2.88380E-10	1.40294E-10	4.20162E-10	1.57533E-10
307.0	2.88394E-10	1.40198E-10	4.19895E-10	1.57458E-10
308.0	2.88407E-10	1.40102E-10	4.19629E-10	1.57384E-10
309.0	2.88421E-10	1.40007E-10	4.19365E-10	1.57310E-10
310.0	2.88435E-10	1.39912E-10	4.19102E-10	1.57236E-10
311.0	2.88449E-10	1.39817E-10	4.18841E-10	1.57163E-10
312.0	2.88463E-10	1.39723E-10	4.18580E-10	1.57090E-10
313.0	2.88478E-10	1.39628E-10	4.18321E-10	1.57017E-10
314.0	2.88492E-10	1.39534E-10	4.18063E-10	1.56944E-10
315.0	2.88507E-10	1.39440E-10	4.17807E-10	1.56872E-10
316.0	2.88522E-10	1.39347E-10	4.17551E-10	1.56799E-10
317.0	2.88536E-10	1.39253E-10	4.17297E-10	1.56728E-10
318.0	2.88551E-10	1.39160E-10	4.17044E-10	1.56656E-10
319.0	2.88567E-10	1.39068E-10	4.16792E-10	1.56584E-10
320.0	2.88582E-10	1.38975E-10	4.16542E-10	1.56513E-10
321.0	2.88597E-10	1.38883E-10	4.16292E-10	1.56442E-10
322.0	2.88613E-10	1.38791E-10	4.16044E-10	1.56372E-10
323.0	2.88628E-10	1.38699E-10	4.15797E-10	1.56302E-10
324.0	2.88644E-10	1.38607E-10	4.15552E-10	1.56231E-10

325.0	2.88660E-10	1.38516E-10	4.15307E-10	1.56162E-10
326.0	2.88676E-10	1.38425E-10	4.15064E-10	1.56092E-10
327.0	2.88692E-10	1.38334E-10	4.14822E-10	1.56023E-10
328.0	2.88708E-10	1.38243E-10	4.14581E-10	1.55953E-10
329.0	2.88724E-10	1.38153E-10	4.14341E-10	1.55885E-10
330.0	2.88741E-10	1.38063E-10	4.14103E-10	1.55816E-10
331.0	2.88757E-10	1.37973E-10	4.13865E-10	1.55748E-10
332.0	2.88774E-10	1.37883E-10	4.13629E-10	1.55680E-10
333.0	2.88791E-10	1.37794E-10	4.13394E-10	1.55612E-10
334.0	2.88807E-10	1.37705E-10	4.13160E-10	1.55544E-10
335.0	2.88824E-10	1.37616E-10	4.12927E-10	1.55477E-10
336.0	2.88841E-10	1.37527E-10	4.12695E-10	1.55410E-10
337.0	2.88859E-10	1.37439E-10	4.12465E-10	1.55343E-10
338.0	2.88876E-10	1.37350E-10	4.12235E-10	1.55276E-10
339.0	2.88893E-10	1.37262E-10	4.12007E-10	1.55210E-10
340.0	2.88911E-10	1.37175E-10	4.11780E-10	1.55143E-10
341.0	2.88928E-10	1.37087E-10	4.11554E-10	1.55078E-10
342.0	2.88946E-10	1.37000E-10	4.11329E-10	1.55012E-10
343.0	2.88963E-10	1.36913E-10	4.11105E-10	1.54946E-10
344.0	2.88981E-10	1.36826E-10	4.10883E-10	1.54881E-10
345.0	2.88999E-10	1.36740E-10	4.10661E-10	1.54816E-10
346.0	2.89017E-10	1.36654E-10	4.10440E-10	1.54751E-10
347.0	2.89035E-10	1.36568E-10	4.10221E-10	1.54687E-10
348.0	2.89053E-10	1.36482E-10	4.10003E-10	1.54623E-10
349.0	2.89072E-10	1.36396E-10	4.09785E-10	1.54559E-10
350.0	2.89090E-10	1.36311E-10	4.09569E-10	1.54495E-10
351.0	2.89108E-10	1.36226E-10	4.09354E-10	1.54431E-10
352.0	2.89127E-10	1.36141E-10	4.09140E-10	1.54368E-10
353.0	2.89146E-10	1.36057E-10	4.08927E-10	1.54305E-10
354.0	2.89164E-10	1.35972E-10	4.08715E-10	1.54242E-10
355.0	2.89183E-10	1.35888E-10	4.08504E-10	1.54179E-10
356.0	2.89202E-10	1.35804E-10	4.08294E-10	1.54117E-10
357.0	2.89221E-10	1.35721E-10	4.08085E-10	1.54054E-10
358.0	2.89240E-10	1.35637E-10	4.07877E-10	1.53992E-10
359.0	2.89259E-10	1.35554E-10	4.07670E-10	1.53931E-10
360.0	2.89278E-10	1.35471E-10	4.07464E-10	1.53869E-10
361.0	2.89297E-10	1.35388E-10	4.07260E-10	1.53808E-10
362.0	2.89316E-10	1.35306E-10	4.07056E-10	1.53746E-10
363.0	2.89336E-10	1.35224E-10	4.06853E-10	1.53686E-10
364.0	2.89355E-10	1.35142E-10	4.06651E-10	1.53625E-10
365.0	2.89375E-10	1.35060E-10	4.06451E-10	1.53564E-10
366.0	2.89394E-10	1.34978E-10	4.06251E-10	1.53504E-10
367.0	2.89414E-10	1.34897E-10	4.06052E-10	1.53444E-10
368.0	2.89433E-10	1.34816E-10	4.05854E-10	1.53384E-10
369.0	2.89453E-10	1.34735E-10	4.05657E-10	1.53325E-10
370.0	2.89473E-10	1.34655E-10	4.05461E-10	1.53265E-10
371.0	2.89493E-10	1.34574E-10	4.05267E-10	1.53206E-10
372.0	2.89513E-10	1.34494E-10	4.05073E-10	1.53147E-10
373.0	2.89533E-10	1.34414E-10	4.04880E-10	1.53088E-10
374.0	2.89553E-10	1.34334E-10	4.04688E-10	1.53030E-10

375.0	2.89573E-10	1.34255E-10	4.04497E-10	1.52971E-10
376.0	2.89593E-10	1.34175E-10	4.04306E-10	1.52913E-10
377.0	2.89613E-10	1.34096E-10	4.04117E-10	1.52855E-10
378.0	2.89633E-10	1.34018E-10	4.03929E-10	1.52797E-10
379.0	2.89654E-10	1.33939E-10	4.03742E-10	1.52740E-10
380.0	2.89674E-10	1.33861E-10	4.03555E-10	1.52683E-10
381.0	2.89694E-10	1.33782E-10	4.03370E-10	1.52625E-10
382.0	2.89715E-10	1.33705E-10	4.03185E-10	1.52568E-10
383.0	2.89735E-10	1.33627E-10	4.03001E-10	1.52512E-10
384.0	2.89756E-10	1.33549E-10	4.02819E-10	1.52455E-10
385.0	2.89777E-10	1.33472E-10	4.02637E-10	1.52399E-10
386.0	2.89797E-10	1.33395E-10	4.02456E-10	1.52343E-10
387.0	2.89818E-10	1.33318E-10	4.02276E-10	1.52287E-10
388.0	2.89839E-10	1.33242E-10	4.02097E-10	1.52231E-10
389.0	2.89860E-10	1.33165E-10	4.01918E-10	1.52175E-10
390.0	2.89880E-10	1.33089E-10	4.01741E-10	1.52120E-10
391.0	2.89901E-10	1.33013E-10	4.01564E-10	1.52065E-10
392.0	2.89922E-10	1.32937E-10	4.01389E-10	1.52010E-10
393.0	2.89943E-10	1.32862E-10	4.01214E-10	1.51955E-10
394.0	2.89964E-10	1.32786E-10	4.01040E-10	1.51901E-10
395.0	2.89985E-10	1.32711E-10	4.00867E-10	1.51846E-10
396.0	2.90006E-10	1.32636E-10	4.00695E-10	1.51792E-10
397.0	2.90027E-10	1.32562E-10	4.00523E-10	1.51738E-10
398.0	2.90049E-10	1.32487E-10	4.00353E-10	1.51684E-10
399.0	2.90070E-10	1.32413E-10	4.00183E-10	1.51630E-10
400.0	2.90091E-10	1.32339E-10	4.00014E-10	1.51577E-10
401.0	2.90112E-10	1.32265E-10	3.99846E-10	1.51524E-10
402.0	2.90133E-10	1.32191E-10	3.99679E-10	1.51470E-10
403.0	2.90155E-10	1.32118E-10	3.99512E-10	1.51418E-10
404.0	2.90176E-10	1.32045E-10	3.99347E-10	1.51365E-10
405.0	2.90197E-10	1.31972E-10	3.99182E-10	1.51312E-10
406.0	2.90219E-10	1.31899E-10	3.99018E-10	1.51260E-10
407.0	2.90240E-10	1.31826E-10	3.98855E-10	1.51208E-10
408.0	2.90262E-10	1.31754E-10	3.98692E-10	1.51156E-10
409.0	2.90283E-10	1.31681E-10	3.98531E-10	1.51104E-10
410.0	2.90305E-10	1.31609E-10	3.98370E-10	1.51052E-10
411.0	2.90326E-10	1.31538E-10	3.98210E-10	1.51001E-10
412.0	2.90348E-10	1.31466E-10	3.98051E-10	1.50949E-10
413.0	2.90369E-10	1.31395E-10	3.97892E-10	1.50898E-10
414.0	2.90391E-10	1.31323E-10	3.97734E-10	1.50847E-10
415.0	2.90413E-10	1.31252E-10	3.97578E-10	1.50796E-10
416.0	2.90434E-10	1.31182E-10	3.97421E-10	1.50746E-10
417.0	2.90456E-10	1.31111E-10	3.97266E-10	1.50695E-10
418.0	2.90478E-10	1.31041E-10	3.97111E-10	1.50645E-10
419.0	2.90499E-10	1.30970E-10	3.96957E-10	1.50595E-10
420.0	2.90521E-10	1.30900E-10	3.96804E-10	1.50545E-10
421.0	2.90543E-10	1.30830E-10	3.96652E-10	1.50495E-10
422.0	2.90565E-10	1.30761E-10	3.96500E-10	1.50445E-10
423.0	2.90586E-10	1.30691E-10	3.96349E-10	1.50396E-10
424.0	2.90608E-10	1.30622E-10	3.96199E-10	1.50346E-10

425.0	2.90630E-10	1.30553E-10	3.96050E-10	1.50297E-10
426.0	2.90652E-10	1.30484E-10	3.95901E-10	1.50248E-10
427.0	2.90674E-10	1.30415E-10	3.95753E-10	1.50199E-10
428.0	2.90696E-10	1.30347E-10	3.95606E-10	1.50151E-10
429.0	2.90717E-10	1.30279E-10	3.95459E-10	1.50102E-10
430.0	2.90739E-10	1.30210E-10	3.95313E-10	1.50054E-10
431.0	2.90761E-10	1.30143E-10	3.95168E-10	1.50006E-10
432.0	2.90783E-10	1.30075E-10	3.95023E-10	1.49958E-10
433.0	2.90805E-10	1.30007E-10	3.94880E-10	1.49910E-10
434.0	2.90827E-10	1.29940E-10	3.94737E-10	1.49862E-10
435.0	2.90849E-10	1.29873E-10	3.94594E-10	1.49814E-10
436.0	2.90871E-10	1.29806E-10	3.94452E-10	1.49767E-10
437.0	2.90892E-10	1.29739E-10	3.94311E-10	1.49720E-10
438.0	2.90914E-10	1.29672E-10	3.94171E-10	1.49672E-10
439.0	2.90936E-10	1.29606E-10	3.94031E-10	1.49626E-10
440.0	2.90958E-10	1.29539E-10	3.93892E-10	1.49579E-10
441.0	2.90980E-10	1.29473E-10	3.93754E-10	1.49532E-10
442.0	2.91002E-10	1.29407E-10	3.93616E-10	1.49485E-10
443.0	2.91024E-10	1.29342E-10	3.93479E-10	1.49439E-10
444.0	2.91046E-10	1.29276E-10	3.93343E-10	1.49393E-10
445.0	2.91068E-10	1.29211E-10	3.93207E-10	1.49347E-10
446.0	2.91090E-10	1.29145E-10	3.93072E-10	1.49301E-10
447.0	2.91112E-10	1.29080E-10	3.92937E-10	1.49255E-10
448.0	2.91134E-10	1.29016E-10	3.92803E-10	1.49209E-10
449.0	2.91156E-10	1.28951E-10	3.92670E-10	1.49164E-10
450.0	2.91177E-10	1.28886E-10	3.92538E-10	1.49118E-10
451.0	2.91199E-10	1.28822E-10	3.92406E-10	1.49073E-10
452.0	2.91221E-10	1.28758E-10	3.92274E-10	1.49028E-10
453.0	2.91243E-10	1.28694E-10	3.92143E-10	1.48983E-10
454.0	2.91265E-10	1.28630E-10	3.92013E-10	1.48938E-10
455.0	2.91287E-10	1.28566E-10	3.91884E-10	1.48894E-10
456.0	2.91309E-10	1.28503E-10	3.91755E-10	1.48849E-10
457.0	2.91331E-10	1.28440E-10	3.91627E-10	1.48805E-10
458.0	2.91352E-10	1.28376E-10	3.91499E-10	1.48760E-10
459.0	2.91374E-10	1.28313E-10	3.91372E-10	1.48716E-10
460.0	2.91396E-10	1.28251E-10	3.91245E-10	1.48672E-10
461.0	2.91418E-10	1.28188E-10	3.91120E-10	1.48628E-10
462.0	2.91440E-10	1.28125E-10	3.90994E-10	1.48585E-10
463.0	2.91462E-10	1.28063E-10	3.90869E-10	1.48541E-10
464.0	2.91483E-10	1.28001E-10	3.90745E-10	1.48497E-10
465.0	2.91505E-10	1.27939E-10	3.90622E-10	1.48454E-10
466.0	2.91527E-10	1.27877E-10	3.90499E-10	1.48411E-10
467.0	2.91549E-10	1.27815E-10	3.90376E-10	1.48368E-10
468.0	2.91570E-10	1.27754E-10	3.90254E-10	1.48325E-10
469.0	2.91592E-10	1.27693E-10	3.90133E-10	1.48282E-10
470.0	2.91614E-10	1.27631E-10	3.90012E-10	1.48239E-10
471.0	2.91635E-10	1.27570E-10	3.89892E-10	1.48196E-10
472.0	2.91657E-10	1.27509E-10	3.89772E-10	1.48154E-10
473.0	2.91679E-10	1.27449E-10	3.89653E-10	1.48112E-10
474.0	2.91700E-10	1.27388E-10	3.89535E-10	1.48069E-10

475.0	2.91722E-10	1.27328E-10	3.89417E-10	1.48027E-10
476.0	2.91743E-10	1.27267E-10	3.89299E-10	1.47985E-10
477.0	2.91765E-10	1.27207E-10	3.89182E-10	1.47943E-10
478.0	2.91786E-10	1.27147E-10	3.89066E-10	1.47902E-10
479.0	2.91808E-10	1.27088E-10	3.88950E-10	1.47860E-10
480.0	2.91829E-10	1.27028E-10	3.88835E-10	1.47818E-10
481.0	2.91851E-10	1.26969E-10	3.88720E-10	1.47777E-10
482.0	2.91872E-10	1.26909E-10	3.88606E-10	1.47736E-10
483.0	2.91894E-10	1.26850E-10	3.88492E-10	1.47695E-10
484.0	2.91915E-10	1.26791E-10	3.88378E-10	1.47654E-10
485.0	2.91936E-10	1.26732E-10	3.88266E-10	1.47613E-10
486.0	2.91958E-10	1.26673E-10	3.88153E-10	1.47572E-10
487.0	2.91979E-10	1.26615E-10	3.88042E-10	1.47531E-10
488.0	2.92000E-10	1.26556E-10	3.87930E-10	1.47490E-10
489.0	2.92021E-10	1.26498E-10	3.87820E-10	1.47450E-10
490.0	2.92043E-10	1.26440E-10	3.87709E-10	1.47410E-10
491.0	2.92064E-10	1.26382E-10	3.87599E-10	1.47369E-10
492.0	2.92085E-10	1.26324E-10	3.87490E-10	1.47329E-10
493.0	2.92106E-10	1.26266E-10	3.87381E-10	1.47289E-10
494.0	2.92127E-10	1.26209E-10	3.87273E-10	1.47249E-10
495.0	2.92148E-10	1.26151E-10	3.87165E-10	1.47209E-10
496.0	2.92169E-10	1.26094E-10	3.87058E-10	1.47169E-10
497.0	2.92190E-10	1.26037E-10	3.86951E-10	1.47130E-10
498.0	2.92211E-10	1.25980E-10	3.86844E-10	1.47090E-10
499.0	2.92232E-10	1.25923E-10	3.86738E-10	1.47051E-10
500.0	2.92253E-10	1.25866E-10	3.86633E-10	1.47011E-10

T (K)	30->20	40->20	40->30
5.0	1.44045E-10	3.55961E-12	7.77076E-12
6.0	1.62601E-10	5.60293E-12	1.17925E-11
7.0	1.77366E-10	8.33507E-12	1.71737E-11
8.0	1.90736E-10	1.15507E-11	2.34382E-11
9.0	2.03640E-10	1.51108E-11	3.02761E-11
10.0	2.16249E-10	1.89112E-11	3.74737E-11
11.0	2.28453E-10	2.28652E-11	4.48706E-11
12.0	2.40085E-10	2.68983E-11	5.23398E-11
13.0	2.51008E-10	3.09482E-11	5.97803E-11
14.0	2.61141E-10	3.49644E-11	6.71139E-11
15.0	2.70454E-10	3.89079E-11	7.42820E-11
16.0	2.78953E-10	4.27499E-11	8.12427E-11
17.0	2.86669E-10	4.64702E-11	8.79681E-11
18.0	2.93650E-10	5.00558E-11	9.44411E-11
19.0	2.99951E-10	5.34991E-11	1.00653E-10
20.0	3.05631E-10	5.67969E-11	1.06601E-10
21.0	3.10747E-10	5.99487E-11	1.12287E-10
22.0	3.15353E-10	6.29569E-11	1.17718E-10
23.0	3.19503E-10	6.58250E-11	1.22900E-10
24.0	3.23242E-10	6.85577E-11	1.27843E-10
25.0	3.26614E-10	7.11604E-11	1.32558E-10
26.0	3.29659E-10	7.36391E-11	1.37053E-10

27.0	3.32410E-10	7.59997E-11	1.41341E-10
28.0	3.34900E-10	7.82482E-11	1.45431E-10
29.0	3.37155E-10	8.03905E-11	1.49334E-10
30.0	3.39201E-10	8.24324E-11	1.53060E-10
31.0	3.41059E-10	8.43796E-11	1.56618E-10
32.0	3.42749E-10	8.62372E-11	1.60018E-10
33.0	3.44287E-10	8.80105E-11	1.63268E-10
34.0	3.45690E-10	8.97041E-11	1.66377E-10
35.0	3.46971E-10	9.13227E-11	1.69351E-10
36.0	3.48141E-10	9.28705E-11	1.72199E-10
37.0	3.49211E-10	9.43514E-11	1.74928E-10
38.0	3.50191E-10	9.57694E-11	1.77542E-10
39.0	3.51088E-10	9.71279E-11	1.80050E-10
40.0	3.51912E-10	9.84303E-11	1.82456E-10
41.0	3.52667E-10	9.96795E-11	1.84765E-10
42.0	3.53361E-10	1.00879E-10	1.86983E-10
43.0	3.53998E-10	1.02030E-10	1.89115E-10
44.0	3.54583E-10	1.03137E-10	1.91164E-10
45.0	3.55121E-10	1.04201E-10	1.93136E-10
46.0	3.55614E-10	1.05225E-10	1.95032E-10
47.0	3.56067E-10	1.06211E-10	1.96859E-10
48.0	3.56483E-10	1.07161E-10	1.98618E-10
49.0	3.56865E-10	1.08076E-10	2.00313E-10
50.0	3.57214E-10	1.08959E-10	2.01948E-10
51.0	3.57533E-10	1.09811E-10	2.03524E-10
52.0	3.57825E-10	1.10633E-10	2.05045E-10
53.0	3.58091E-10	1.11427E-10	2.06513E-10
54.0	3.58332E-10	1.12194E-10	2.07931E-10
55.0	3.58551E-10	1.12936E-10	2.09300E-10
56.0	3.58749E-10	1.13654E-10	2.10624E-10
57.0	3.58927E-10	1.14348E-10	2.11904E-10
58.0	3.59085E-10	1.15020E-10	2.13141E-10
59.0	3.59227E-10	1.15672E-10	2.14339E-10
60.0	3.59351E-10	1.16303E-10	2.15497E-10
61.0	3.59460E-10	1.16914E-10	2.16619E-10
62.0	3.59553E-10	1.17508E-10	2.17705E-10
63.0	3.59633E-10	1.18083E-10	2.18758E-10
64.0	3.59698E-10	1.18642E-10	2.19778E-10
65.0	3.59752E-10	1.19184E-10	2.20767E-10
66.0	3.59793E-10	1.19711E-10	2.21725E-10
67.0	3.59822E-10	1.20223E-10	2.22655E-10
68.0	3.59840E-10	1.20721E-10	2.23557E-10
69.0	3.59848E-10	1.21205E-10	2.24433E-10
70.0	3.59846E-10	1.21676E-10	2.25283E-10
71.0	3.59834E-10	1.22134E-10	2.26108E-10
72.0	3.59812E-10	1.22580E-10	2.26910E-10
73.0	3.59782E-10	1.23014E-10	2.27688E-10
74.0	3.59744E-10	1.23436E-10	2.28445E-10
75.0	3.59697E-10	1.23848E-10	2.29181E-10
76.0	3.59643E-10	1.24249E-10	2.29896E-10

77.0	3.59581E-10	1.24641E-10	2.30592E-10
78.0	3.59512E-10	1.25022E-10	2.31268E-10
79.0	3.59437E-10	1.25394E-10	2.31926E-10
80.0	3.59354E-10	1.25757E-10	2.32567E-10
81.0	3.59266E-10	1.26111E-10	2.33190E-10
82.0	3.59171E-10	1.26457E-10	2.33797E-10
83.0	3.59070E-10	1.26794E-10	2.34388E-10
84.0	3.58964E-10	1.27124E-10	2.34964E-10
85.0	3.58852E-10	1.27445E-10	2.35524E-10
86.0	3.58735E-10	1.27760E-10	2.36071E-10
87.0	3.58613E-10	1.28067E-10	2.36603E-10
88.0	3.58486E-10	1.28367E-10	2.37122E-10
89.0	3.58355E-10	1.28661E-10	2.37628E-10
90.0	3.58219E-10	1.28948E-10	2.38121E-10
91.0	3.58079E-10	1.29229E-10	2.38602E-10
92.0	3.57934E-10	1.29503E-10	2.39071E-10
93.0	3.57786E-10	1.29772E-10	2.39529E-10
94.0	3.57634E-10	1.30035E-10	2.39975E-10
95.0	3.57478E-10	1.30292E-10	2.40411E-10
96.0	3.57318E-10	1.30544E-10	2.40836E-10
97.0	3.57155E-10	1.30790E-10	2.41251E-10
98.0	3.56989E-10	1.31032E-10	2.41657E-10
99.0	3.56820E-10	1.31268E-10	2.42052E-10
100.0	3.56647E-10	1.31500E-10	2.42439E-10
101.0	3.56472E-10	1.31726E-10	2.42816E-10
102.0	3.56294E-10	1.31949E-10	2.43185E-10
103.0	3.56113E-10	1.32167E-10	2.43545E-10
104.0	3.55929E-10	1.32380E-10	2.43897E-10
105.0	3.55743E-10	1.32589E-10	2.44241E-10
106.0	3.55554E-10	1.32795E-10	2.44577E-10
107.0	3.55364E-10	1.32996E-10	2.44905E-10
108.0	3.55171E-10	1.33193E-10	2.45226E-10
109.0	3.54975E-10	1.33387E-10	2.45540E-10
110.0	3.54778E-10	1.33576E-10	2.45847E-10
111.0	3.54579E-10	1.33763E-10	2.46148E-10
112.0	3.54378E-10	1.33945E-10	2.46441E-10
113.0	3.54175E-10	1.34125E-10	2.46728E-10
114.0	3.53970E-10	1.34301E-10	2.47009E-10
115.0	3.53764E-10	1.34473E-10	2.47284E-10
116.0	3.53556E-10	1.34643E-10	2.47553E-10
117.0	3.53346E-10	1.34810E-10	2.47816E-10
118.0	3.53135E-10	1.34973E-10	2.48073E-10
119.0	3.52923E-10	1.35133E-10	2.48325E-10
120.0	3.52710E-10	1.35291E-10	2.48572E-10
121.0	3.52495E-10	1.35446E-10	2.48813E-10
122.0	3.52279E-10	1.35598E-10	2.49050E-10
123.0	3.52062E-10	1.35747E-10	2.49281E-10
124.0	3.51843E-10	1.35894E-10	2.49508E-10
125.0	3.51624E-10	1.36039E-10	2.49729E-10
126.0	3.51404E-10	1.36180E-10	2.49947E-10

127.0	3.51183E-10	1.36320E-10	2.50159E-10
128.0	3.50961E-10	1.36457E-10	2.50368E-10
129.0	3.50738E-10	1.36591E-10	2.50572E-10
130.0	3.50514E-10	1.36724E-10	2.50772E-10
131.0	3.50290E-10	1.36854E-10	2.50968E-10
132.0	3.50065E-10	1.36982E-10	2.51160E-10
133.0	3.49839E-10	1.37107E-10	2.51348E-10
134.0	3.49613E-10	1.37231E-10	2.51532E-10
135.0	3.49386E-10	1.37353E-10	2.51713E-10
136.0	3.49159E-10	1.37472E-10	2.51890E-10
137.0	3.48931E-10	1.37590E-10	2.52063E-10
138.0	3.48703E-10	1.37706E-10	2.52233E-10
139.0	3.48474E-10	1.37820E-10	2.52400E-10
140.0	3.48245E-10	1.37932E-10	2.52563E-10
141.0	3.48016E-10	1.38042E-10	2.52723E-10
142.0	3.47786E-10	1.38151E-10	2.52880E-10
143.0	3.47556E-10	1.38258E-10	2.53034E-10
144.0	3.47326E-10	1.38363E-10	2.53185E-10
145.0	3.47096E-10	1.38466E-10	2.53333E-10
146.0	3.46865E-10	1.38568E-10	2.53478E-10
147.0	3.46635E-10	1.38669E-10	2.53620E-10
148.0	3.46404E-10	1.38767E-10	2.53760E-10
149.0	3.46173E-10	1.38865E-10	2.53897E-10
150.0	3.45943E-10	1.38960E-10	2.54031E-10
151.0	3.45712E-10	1.39055E-10	2.54163E-10
152.0	3.45481E-10	1.39147E-10	2.54292E-10
153.0	3.45250E-10	1.39239E-10	2.54419E-10
154.0	3.45019E-10	1.39329E-10	2.54543E-10
155.0	3.44788E-10	1.39418E-10	2.54665E-10
156.0	3.44558E-10	1.39505E-10	2.54785E-10
157.0	3.44327E-10	1.39591E-10	2.54902E-10
158.0	3.44097E-10	1.39676E-10	2.55017E-10
159.0	3.43867E-10	1.39760E-10	2.55131E-10
160.0	3.43637E-10	1.39842E-10	2.55241E-10
161.0	3.43407E-10	1.39923E-10	2.55350E-10
162.0	3.43177E-10	1.40003E-10	2.55457E-10
163.0	3.42948E-10	1.40082E-10	2.55562E-10
164.0	3.42719E-10	1.40160E-10	2.55665E-10
165.0	3.42490E-10	1.40236E-10	2.55766E-10
166.0	3.42261E-10	1.40312E-10	2.55865E-10
167.0	3.42033E-10	1.40386E-10	2.55963E-10
168.0	3.41805E-10	1.40460E-10	2.56058E-10
169.0	3.41578E-10	1.40532E-10	2.56152E-10
170.0	3.41350E-10	1.40603E-10	2.56244E-10
171.0	3.41123E-10	1.40674E-10	2.56335E-10
172.0	3.40897E-10	1.40743E-10	2.56423E-10
173.0	3.40671E-10	1.40812E-10	2.56511E-10
174.0	3.40445E-10	1.40879E-10	2.56596E-10
175.0	3.40220E-10	1.40946E-10	2.56680E-10
176.0	3.39995E-10	1.41011E-10	2.56763E-10

177.0	3.39770E-10	1.41076E-10	2.56844E-10
178.0	3.39547E-10	1.41140E-10	2.56924E-10
179.0	3.39323E-10	1.41203E-10	2.57002E-10
180.0	3.39100E-10	1.41265E-10	2.57079E-10
181.0	3.38878E-10	1.41327E-10	2.57154E-10
182.0	3.38656E-10	1.41387E-10	2.57228E-10
183.0	3.38434E-10	1.41447E-10	2.57301E-10
184.0	3.38213E-10	1.41506E-10	2.57372E-10
185.0	3.37993E-10	1.41564E-10	2.57443E-10
186.0	3.37773E-10	1.41621E-10	2.57512E-10
187.0	3.37553E-10	1.41678E-10	2.57580E-10
188.0	3.37335E-10	1.41734E-10	2.57646E-10
189.0	3.37116E-10	1.41789E-10	2.57712E-10
190.0	3.36899E-10	1.41844E-10	2.57776E-10
191.0	3.36682E-10	1.41898E-10	2.57839E-10
192.0	3.36465E-10	1.41951E-10	2.57901E-10
193.0	3.36249E-10	1.42003E-10	2.57962E-10
194.0	3.36034E-10	1.42055E-10	2.58022E-10
195.0	3.35820E-10	1.42106E-10	2.58081E-10
196.0	3.35606E-10	1.42157E-10	2.58139E-10
197.0	3.35392E-10	1.42207E-10	2.58196E-10
198.0	3.35179E-10	1.42256E-10	2.58252E-10
199.0	3.34967E-10	1.42305E-10	2.58307E-10
200.0	3.34756E-10	1.42353E-10	2.58361E-10
201.0	3.34545E-10	1.42400E-10	2.58414E-10
202.0	3.34335E-10	1.42447E-10	2.58467E-10
203.0	3.34125E-10	1.42494E-10	2.58518E-10
204.0	3.33917E-10	1.42540E-10	2.58569E-10
205.0	3.33708E-10	1.42585E-10	2.58618E-10
206.0	3.33501E-10	1.42630E-10	2.58667E-10
207.0	3.33294E-10	1.42674E-10	2.58715E-10
208.0	3.33088E-10	1.42718E-10	2.58762E-10
209.0	3.32883E-10	1.42761E-10	2.58809E-10
210.0	3.32678E-10	1.42803E-10	2.58855E-10
211.0	3.32474E-10	1.42846E-10	2.58900E-10
212.0	3.32270E-10	1.42887E-10	2.58944E-10
213.0	3.32068E-10	1.42929E-10	2.58987E-10
214.0	3.31866E-10	1.42969E-10	2.59030E-10
215.0	3.31665E-10	1.43010E-10	2.59072E-10
216.0	3.31464E-10	1.43049E-10	2.59114E-10
217.0	3.31264E-10	1.43089E-10	2.59154E-10
218.0	3.31065E-10	1.43128E-10	2.59195E-10
219.0	3.30867E-10	1.43166E-10	2.59234E-10
220.0	3.30669E-10	1.43204E-10	2.59273E-10
221.0	3.30473E-10	1.43242E-10	2.59311E-10
222.0	3.30277E-10	1.43279E-10	2.59349E-10
223.0	3.30081E-10	1.43316E-10	2.59386E-10
224.0	3.29886E-10	1.43353E-10	2.59422E-10
225.0	3.29693E-10	1.43389E-10	2.59458E-10
226.0	3.29499E-10	1.43424E-10	2.59494E-10

227.0	3.29307E-10	1.43460E-10	2.59528E-10
228.0	3.29115E-10	1.43495E-10	2.59563E-10
229.0	3.28924E-10	1.43529E-10	2.59596E-10
230.0	3.28734E-10	1.43563E-10	2.59630E-10
231.0	3.28545E-10	1.43597E-10	2.59662E-10
232.0	3.28356E-10	1.43631E-10	2.59695E-10
233.0	3.28168E-10	1.43664E-10	2.59726E-10
234.0	3.27981E-10	1.43696E-10	2.59758E-10
235.0	3.27794E-10	1.43729E-10	2.59788E-10
236.0	3.27609E-10	1.43761E-10	2.59819E-10
237.0	3.27424E-10	1.43793E-10	2.59849E-10
238.0	3.27240E-10	1.43824E-10	2.59878E-10
239.0	3.27056E-10	1.43855E-10	2.59907E-10
240.0	3.26873E-10	1.43886E-10	2.59936E-10
241.0	3.26692E-10	1.43917E-10	2.59964E-10
242.0	3.26510E-10	1.43947E-10	2.59992E-10
243.0	3.26330E-10	1.43977E-10	2.60019E-10
244.0	3.26150E-10	1.44007E-10	2.60046E-10
245.0	3.25972E-10	1.44036E-10	2.60073E-10
246.0	3.25793E-10	1.44065E-10	2.60099E-10
247.0	3.25616E-10	1.44094E-10	2.60125E-10
248.0	3.25440E-10	1.44122E-10	2.60151E-10
249.0	3.25264E-10	1.44150E-10	2.60176E-10
250.0	3.25089E-10	1.44178E-10	2.60201E-10
251.0	3.24914E-10	1.44206E-10	2.60226E-10
252.0	3.24741E-10	1.44234E-10	2.60250E-10
253.0	3.24568E-10	1.44261E-10	2.60274E-10
254.0	3.24396E-10	1.44288E-10	2.60298E-10
255.0	3.24225E-10	1.44314E-10	2.60321E-10
256.0	3.24054E-10	1.44341E-10	2.60344E-10
257.0	3.23885E-10	1.44367E-10	2.60367E-10
258.0	3.23716E-10	1.44393E-10	2.60389E-10
259.0	3.23547E-10	1.44419E-10	2.60411E-10
260.0	3.23380E-10	1.44444E-10	2.60433E-10
261.0	3.23213E-10	1.44470E-10	2.60455E-10
262.0	3.23047E-10	1.44495E-10	2.60476E-10
263.0	3.22882E-10	1.44519E-10	2.60497E-10
264.0	3.22718E-10	1.44544E-10	2.60518E-10
265.0	3.22554E-10	1.44568E-10	2.60539E-10
266.0	3.22391E-10	1.44593E-10	2.60559E-10
267.0	3.22229E-10	1.44617E-10	2.60580E-10
268.0	3.22068E-10	1.44640E-10	2.60599E-10
269.0	3.21907E-10	1.44664E-10	2.60619E-10
270.0	3.21747E-10	1.44687E-10	2.60639E-10
271.0	3.21588E-10	1.44711E-10	2.60658E-10
272.0	3.21429E-10	1.44734E-10	2.60677E-10
273.0	3.21272E-10	1.44756E-10	2.60696E-10
274.0	3.21115E-10	1.44779E-10	2.60715E-10
275.0	3.20959E-10	1.44801E-10	2.60733E-10
276.0	3.20803E-10	1.44824E-10	2.60751E-10

277.0	3.20648E-10	1.44846E-10	2.60769E-10
278.0	3.20495E-10	1.44867E-10	2.60787E-10
279.0	3.20341E-10	1.44889E-10	2.60805E-10
280.0	3.20189E-10	1.44911E-10	2.60823E-10
281.0	3.20037E-10	1.44932E-10	2.60840E-10
282.0	3.19886E-10	1.44953E-10	2.60857E-10
283.0	3.19736E-10	1.44974E-10	2.60874E-10
284.0	3.19586E-10	1.44995E-10	2.60891E-10
285.0	3.19437E-10	1.45016E-10	2.60908E-10
286.0	3.19289E-10	1.45036E-10	2.60925E-10
287.0	3.19142E-10	1.45057E-10	2.60941E-10
288.0	3.18995E-10	1.45077E-10	2.60957E-10
289.0	3.18850E-10	1.45097E-10	2.60974E-10
290.0	3.18704E-10	1.45117E-10	2.60990E-10
291.0	3.18560E-10	1.45137E-10	2.61006E-10
292.0	3.18416E-10	1.45156E-10	2.61021E-10
293.0	3.18273E-10	1.45176E-10	2.61037E-10
294.0	3.18131E-10	1.45195E-10	2.61053E-10
295.0	3.17989E-10	1.45214E-10	2.61068E-10
296.0	3.17848E-10	1.45233E-10	2.61083E-10
297.0	3.17708E-10	1.45252E-10	2.61099E-10
298.0	3.17569E-10	1.45271E-10	2.61114E-10
299.0	3.17430E-10	1.45290E-10	2.61129E-10
300.0	3.17292E-10	1.45308E-10	2.61143E-10
301.0	3.17155E-10	1.45326E-10	2.61158E-10
302.0	3.17018E-10	1.45345E-10	2.61173E-10
303.0	3.16882E-10	1.45363E-10	2.61187E-10
304.0	3.16747E-10	1.45381E-10	2.61202E-10
305.0	3.16612E-10	1.45399E-10	2.61216E-10
306.0	3.16478E-10	1.45416E-10	2.61231E-10
307.0	3.16345E-10	1.45434E-10	2.61245E-10
308.0	3.16213E-10	1.45451E-10	2.61259E-10
309.0	3.16081E-10	1.45469E-10	2.61273E-10
310.0	3.15950E-10	1.45486E-10	2.61287E-10
311.0	3.15820E-10	1.45503E-10	2.61301E-10
312.0	3.15690E-10	1.45520E-10	2.61315E-10
313.0	3.15561E-10	1.45537E-10	2.61328E-10
314.0	3.15433E-10	1.45554E-10	2.61342E-10
315.0	3.15305E-10	1.45570E-10	2.61356E-10
316.0	3.15178E-10	1.45587E-10	2.61369E-10
317.0	3.15052E-10	1.45603E-10	2.61383E-10
318.0	3.14926E-10	1.45620E-10	2.61396E-10
319.0	3.14801E-10	1.45636E-10	2.61409E-10
320.0	3.14677E-10	1.45652E-10	2.61423E-10
321.0	3.14553E-10	1.45668E-10	2.61436E-10
322.0	3.14430E-10	1.45684E-10	2.61449E-10
323.0	3.14308E-10	1.45700E-10	2.61462E-10
324.0	3.14186E-10	1.45715E-10	2.61475E-10
325.0	3.14065E-10	1.45731E-10	2.61488E-10
326.0	3.13944E-10	1.45747E-10	2.61501E-10

327.0	3.13825E-10	1.45762E-10	2.61514E-10
328.0	3.13706E-10	1.45777E-10	2.61527E-10
329.0	3.13587E-10	1.45792E-10	2.61540E-10
330.0	3.13469E-10	1.45807E-10	2.61553E-10
331.0	3.13352E-10	1.45822E-10	2.61565E-10
332.0	3.13236E-10	1.45837E-10	2.61578E-10
333.0	3.13120E-10	1.45852E-10	2.61591E-10
334.0	3.13005E-10	1.45867E-10	2.61603E-10
335.0	3.12890E-10	1.45882E-10	2.61616E-10
336.0	3.12776E-10	1.45896E-10	2.61628E-10
337.0	3.12663E-10	1.45911E-10	2.61641E-10
338.0	3.12550E-10	1.45925E-10	2.61653E-10
339.0	3.12438E-10	1.45939E-10	2.61666E-10
340.0	3.12327E-10	1.45953E-10	2.61678E-10
341.0	3.12216E-10	1.45967E-10	2.61691E-10
342.0	3.12105E-10	1.45981E-10	2.61703E-10
343.0	3.11996E-10	1.45995E-10	2.61715E-10
344.0	3.11887E-10	1.46009E-10	2.61727E-10
345.0	3.11778E-10	1.46023E-10	2.61740E-10
346.0	3.11671E-10	1.46037E-10	2.61752E-10
347.0	3.11563E-10	1.46050E-10	2.61764E-10
348.0	3.11457E-10	1.46064E-10	2.61776E-10
349.0	3.11351E-10	1.46077E-10	2.61788E-10
350.0	3.11245E-10	1.46090E-10	2.61801E-10
351.0	3.11141E-10	1.46104E-10	2.61813E-10
352.0	3.11036E-10	1.46117E-10	2.61825E-10
353.0	3.10933E-10	1.46130E-10	2.61837E-10
354.0	3.10830E-10	1.46143E-10	2.61849E-10
355.0	3.10727E-10	1.46156E-10	2.61861E-10
356.0	3.10626E-10	1.46169E-10	2.61873E-10
357.0	3.10524E-10	1.46181E-10	2.61885E-10
358.0	3.10424E-10	1.46194E-10	2.61897E-10
359.0	3.10324E-10	1.46207E-10	2.61909E-10
360.0	3.10224E-10	1.46219E-10	2.61921E-10
361.0	3.10125E-10	1.46232E-10	2.61932E-10
362.0	3.10027E-10	1.46244E-10	2.61944E-10
363.0	3.09929E-10	1.46256E-10	2.61956E-10
364.0	3.09832E-10	1.46269E-10	2.61968E-10
365.0	3.09735E-10	1.46281E-10	2.61980E-10
366.0	3.09639E-10	1.46293E-10	2.61992E-10
367.0	3.09543E-10	1.46305E-10	2.62003E-10
368.0	3.09448E-10	1.46317E-10	2.62015E-10
369.0	3.09354E-10	1.46329E-10	2.62027E-10
370.0	3.09260E-10	1.46340E-10	2.62039E-10
371.0	3.09166E-10	1.46352E-10	2.62051E-10
372.0	3.09073E-10	1.46364E-10	2.62062E-10
373.0	3.08981E-10	1.46375E-10	2.62074E-10
374.0	3.08889E-10	1.46387E-10	2.62086E-10
375.0	3.08798E-10	1.46398E-10	2.62097E-10
376.0	3.08708E-10	1.46410E-10	2.62109E-10

377.0	3.08617E-10	1.46421E-10	2.62121E-10
378.0	3.08528E-10	1.46432E-10	2.62132E-10
379.0	3.08439E-10	1.46443E-10	2.62144E-10
380.0	3.08350E-10	1.46454E-10	2.62156E-10
381.0	3.08262E-10	1.46465E-10	2.62167E-10
382.0	3.08175E-10	1.46476E-10	2.62179E-10
383.0	3.08088E-10	1.46487E-10	2.62190E-10
384.0	3.08001E-10	1.46498E-10	2.62202E-10
385.0	3.07915E-10	1.46509E-10	2.62214E-10
386.0	3.07830E-10	1.46519E-10	2.62225E-10
387.0	3.07745E-10	1.46530E-10	2.62237E-10
388.0	3.07660E-10	1.46540E-10	2.62248E-10
389.0	3.07576E-10	1.46551E-10	2.62260E-10
390.0	3.07493E-10	1.46561E-10	2.62271E-10
391.0	3.07410E-10	1.46572E-10	2.62283E-10
392.0	3.07328E-10	1.46582E-10	2.62294E-10
393.0	3.07246E-10	1.46592E-10	2.62306E-10
394.0	3.07164E-10	1.46602E-10	2.62317E-10
395.0	3.07083E-10	1.46612E-10	2.62329E-10
396.0	3.07003E-10	1.46622E-10	2.62340E-10
397.0	3.06923E-10	1.46632E-10	2.62351E-10
398.0	3.06843E-10	1.46642E-10	2.62363E-10
399.0	3.06764E-10	1.46652E-10	2.62374E-10
400.0	3.06686E-10	1.46661E-10	2.62386E-10
401.0	3.06607E-10	1.46671E-10	2.62397E-10
402.0	3.06530E-10	1.46680E-10	2.62408E-10
403.0	3.06453E-10	1.46690E-10	2.62420E-10
404.0	3.06376E-10	1.46699E-10	2.62431E-10
405.0	3.06300E-10	1.46709E-10	2.62442E-10
406.0	3.06224E-10	1.46718E-10	2.62454E-10
407.0	3.06149E-10	1.46727E-10	2.62465E-10
408.0	3.06074E-10	1.46736E-10	2.62476E-10
409.0	3.06000E-10	1.46746E-10	2.62487E-10
410.0	3.05926E-10	1.46755E-10	2.62498E-10
411.0	3.05852E-10	1.46764E-10	2.62510E-10
412.0	3.05779E-10	1.46773E-10	2.62521E-10
413.0	3.05706E-10	1.46781E-10	2.62532E-10
414.0	3.05634E-10	1.46790E-10	2.62543E-10
415.0	3.05563E-10	1.46799E-10	2.62554E-10
416.0	3.05491E-10	1.46807E-10	2.62565E-10
417.0	3.05421E-10	1.46816E-10	2.62577E-10
418.0	3.05350E-10	1.46825E-10	2.62588E-10
419.0	3.05280E-10	1.46833E-10	2.62599E-10
420.0	3.05211E-10	1.46841E-10	2.62610E-10
421.0	3.05142E-10	1.46850E-10	2.62621E-10
422.0	3.05073E-10	1.46858E-10	2.62632E-10
423.0	3.05005E-10	1.46866E-10	2.62643E-10
424.0	3.04937E-10	1.46874E-10	2.62654E-10
425.0	3.04869E-10	1.46882E-10	2.62665E-10
426.0	3.04802E-10	1.46890E-10	2.62675E-10

427.0	3.04736E-10	1.46898E-10	2.62686E-10
428.0	3.04670E-10	1.46906E-10	2.62697E-10
429.0	3.04604E-10	1.46914E-10	2.62708E-10
430.0	3.04538E-10	1.46921E-10	2.62719E-10
431.0	3.04473E-10	1.46929E-10	2.62730E-10
432.0	3.04409E-10	1.46937E-10	2.62740E-10
433.0	3.04345E-10	1.46944E-10	2.62751E-10
434.0	3.04281E-10	1.46952E-10	2.62762E-10
435.0	3.04218E-10	1.46959E-10	2.62773E-10
436.0	3.04155E-10	1.46966E-10	2.62783E-10
437.0	3.04092E-10	1.46974E-10	2.62794E-10
438.0	3.04030E-10	1.46981E-10	2.62804E-10
439.0	3.03968E-10	1.46988E-10	2.62815E-10
440.0	3.03906E-10	1.46995E-10	2.62826E-10
441.0	3.03845E-10	1.47002E-10	2.62836E-10
442.0	3.03785E-10	1.47009E-10	2.62847E-10
443.0	3.03724E-10	1.47016E-10	2.62857E-10
444.0	3.03664E-10	1.47022E-10	2.62867E-10
445.0	3.03605E-10	1.47029E-10	2.62878E-10
446.0	3.03546E-10	1.47036E-10	2.62888E-10
447.0	3.03487E-10	1.47042E-10	2.62898E-10
448.0	3.03428E-10	1.47049E-10	2.62909E-10
449.0	3.03370E-10	1.47055E-10	2.62919E-10
450.0	3.03312E-10	1.47062E-10	2.62929E-10
451.0	3.03255E-10	1.47068E-10	2.62939E-10
452.0	3.03198E-10	1.47074E-10	2.62950E-10
453.0	3.03141E-10	1.47080E-10	2.62960E-10
454.0	3.03085E-10	1.47087E-10	2.62970E-10
455.0	3.03029E-10	1.47093E-10	2.62980E-10
456.0	3.02973E-10	1.47099E-10	2.62990E-10
457.0	3.02918E-10	1.47105E-10	2.63000E-10
458.0	3.02863E-10	1.47110E-10	2.63010E-10
459.0	3.02808E-10	1.47116E-10	2.63020E-10
460.0	3.02754E-10	1.47122E-10	2.63029E-10
461.0	3.02700E-10	1.47127E-10	2.63039E-10
462.0	3.02646E-10	1.47133E-10	2.63049E-10
463.0	3.02593E-10	1.47138E-10	2.63059E-10
464.0	3.02540E-10	1.47144E-10	2.63068E-10
465.0	3.02488E-10	1.47149E-10	2.63078E-10
466.0	3.02435E-10	1.47155E-10	2.63088E-10
467.0	3.02383E-10	1.47160E-10	2.63097E-10
468.0	3.02332E-10	1.47165E-10	2.63107E-10
469.0	3.02280E-10	1.47170E-10	2.63116E-10
470.0	3.02229E-10	1.47175E-10	2.63125E-10
471.0	3.02178E-10	1.47180E-10	2.63135E-10
472.0	3.02128E-10	1.47185E-10	2.63144E-10
473.0	3.02078E-10	1.47190E-10	2.63153E-10
474.0	3.02028E-10	1.47194E-10	2.63163E-10
475.0	3.01979E-10	1.47199E-10	2.63172E-10
476.0	3.01929E-10	1.47204E-10	2.63181E-10

477.0	3.01880E-10	1.47208E-10	2.63190E-10
478.0	3.01832E-10	1.47213E-10	2.63199E-10
479.0	3.01784E-10	1.47217E-10	2.63208E-10
480.0	3.01736E-10	1.47221E-10	2.63217E-10
481.0	3.01688E-10	1.47225E-10	2.63226E-10
482.0	3.01640E-10	1.47230E-10	2.63235E-10
483.0	3.01593E-10	1.47234E-10	2.63243E-10
484.0	3.01546E-10	1.47238E-10	2.63252E-10
485.0	3.01500E-10	1.47242E-10	2.63261E-10
486.0	3.01454E-10	1.47246E-10	2.63269E-10
487.0	3.01408E-10	1.47249E-10	2.63278E-10
488.0	3.01362E-10	1.47253E-10	2.63286E-10
489.0	3.01316E-10	1.47257E-10	2.63295E-10
490.0	3.01271E-10	1.47260E-10	2.63303E-10
491.0	3.01226E-10	1.47264E-10	2.63311E-10
492.0	3.01182E-10	1.47267E-10	2.63320E-10
493.0	3.01137E-10	1.47271E-10	2.63328E-10
494.0	3.01093E-10	1.47274E-10	2.63336E-10
495.0	3.01049E-10	1.47277E-10	2.63344E-10
496.0	3.01006E-10	1.47280E-10	2.63352E-10
497.0	3.00962E-10	1.47283E-10	2.63360E-10
498.0	3.00919E-10	1.47287E-10	2.63368E-10
499.0	3.00876E-10	1.47289E-10	2.63375E-10
500.0	3.00834E-10	1.47292E-10	2.63383E-10

Table S7: Computed excitation and de-excitation rate coefficients in cm^3 $molecule^{-1}$ s^{-1} units for inelastic processes generated using the 4D RR-PES for the $HeH^+(j_1 = 0)\cdot\cdot\cdot$ para-$H_2(j_2)$ system with $\Delta j_2 = +2$ and $\Delta j_2 = -2$.

T (K)	00->02	02->00
5.0	7.08022E-56	5.50211E-11
6.0	2.84773E-48	5.58136E-11
7.0	7.66100E-43	5.61320E-11
8.0	9.08867E-39	5.66159E-11
9.0	1.34981E-35	5.73821E-11
10.0	4.68175E-33	5.83666E-11
11.0	5.63354E-31	5.94689E-11
12.0	3.06094E-29	6.06024E-11
13.0	9.01346E-28	6.17058E-11
14.0	1.63899E-26	6.27411E-11
15.0	2.02555E-25	6.36878E-11
16.0	1.82843E-24	6.45373E-11
17.0	1.27393E-23	6.52888E-11
18.0	7.15188E-23	6.59458E-11
19.0	3.34705E-22	6.65145E-11
20.0	1.34182E-21	6.70021E-11
21.0	4.71089E-21	6.74162E-11
22.0	1.47479E-20	6.77642E-11
23.0	4.17896E-20	6.80532E-11
24.0	1.08523E-19	6.82894E-11
25.0	2.60998E-19	6.84789E-11

26.0	5.86491E-19	6.86267E-11
27.0	1.24072E-18	6.87376E-11
28.0	2.48704E-18	6.88156E-11
29.0	4.75023E-18	6.88644E-11
30.0	8.68694E-18	6.88872E-11
31.0	1.52746E-17	6.88869E-11
32.0	2.59193E-17	6.88659E-11
33.0	4.25828E-17	6.88266E-11
34.0	6.79272E-17	6.87709E-11
35.0	1.05475E-16	6.87004E-11
36.0	1.59784E-16	6.86169E-11
37.0	2.36625E-16	6.85216E-11
38.0	3.43174E-16	6.84158E-11
39.0	4.88195E-16	6.83006E-11
40.0	6.82223E-16	6.81769E-11
41.0	9.37739E-16	6.80457E-11
42.0	1.26933E-15	6.79078E-11
43.0	1.69383E-15	6.77638E-11
44.0	2.23044E-15	6.76145E-11
45.0	2.90084E-15	6.74603E-11
46.0	3.72926E-15	6.73019E-11
47.0	4.74251E-15	6.71398E-11
48.0	5.97004E-15	6.69743E-11
49.0	7.44389E-15	6.68058E-11
50.0	9.19870E-15	6.66348E-11
51.0	1.12716E-14	6.64616E-11
52.0	1.37023E-14	6.62864E-11
53.0	1.65327E-14	6.61096E-11
54.0	1.98069E-14	6.59314E-11
55.0	2.35713E-14	6.57520E-11
56.0	2.78741E-14	6.55718E-11
57.0	3.27651E-14	6.53907E-11
58.0	3.82960E-14	6.52092E-11
59.0	4.45197E-14	6.50272E-11
60.0	5.14904E-14	6.48451E-11
61.0	5.92631E-14	6.46628E-11
62.0	6.78939E-14	6.44807E-11
63.0	7.74393E-14	6.42987E-11
64.0	8.79563E-14	6.41170E-11
65.0	9.95021E-14	6.39356E-11
66.0	1.12134E-13	6.37548E-11
67.0	1.25909E-13	6.35746E-11
68.0	1.40883E-13	6.33950E-11
69.0	1.57114E-13	6.32162E-11
70.0	1.74656E-13	6.30382E-11
71.0	1.93565E-13	6.28610E-11
72.0	2.13894E-13	6.26848E-11
73.0	2.35696E-13	6.25095E-11
74.0	2.59022E-13	6.23353E-11
75.0	2.83922E-13	6.21622E-11

76.0	3.10447E-13	6.19901E-11
77.0	3.38641E-13	6.18192E-11
78.0	3.68552E-13	6.16495E-11
79.0	4.00224E-13	6.14810E-11
80.0	4.33698E-13	6.13138E-11
81.0	4.69016E-13	6.11478E-11
82.0	5.06215E-13	6.09831E-11
83.0	5.45333E-13	6.08197E-11
84.0	5.86404E-13	6.06576E-11
85.0	6.29463E-13	6.04968E-11
86.0	6.74539E-13	6.03373E-11
87.0	7.21662E-13	6.01793E-11
88.0	7.70859E-13	6.00225E-11
89.0	8.22155E-13	5.98672E-11
90.0	8.75575E-13	5.97132E-11
91.0	9.31138E-13	5.95606E-11
92.0	9.88866E-13	5.94093E-11
93.0	1.04877E-12	5.92595E-11
94.0	1.11088E-12	5.91110E-11
95.0	1.17520E-12	5.89639E-11
96.0	1.24174E-12	5.88181E-11
97.0	1.31052E-12	5.86738E-11
98.0	1.38154E-12	5.85308E-11
99.0	1.45480E-12	5.83891E-11
100.0	1.53033E-12	5.82488E-11
101.0	1.60811E-12	5.81099E-11
102.0	1.68816E-12	5.79723E-11
103.0	1.77047E-12	5.78360E-11
104.0	1.85504E-12	5.77010E-11
105.0	1.94187E-12	5.75674E-11
106.0	2.03096E-12	5.74351E-11
107.0	2.12230E-12	5.73040E-11
108.0	2.21589E-12	5.71743E-11
109.0	2.31172E-12	5.70458E-11
110.0	2.40977E-12	5.69185E-11
111.0	2.51005E-12	5.67926E-11
112.0	2.61254E-12	5.66679E-11
113.0	2.71723E-12	5.65444E-11
114.0	2.82411E-12	5.64221E-11
115.0	2.93316E-12	5.63010E-11
116.0	3.04437E-12	5.61811E-11
117.0	3.15773E-12	5.60624E-11
118.0	3.27321E-12	5.59449E-11
119.0	3.39080E-12	5.58285E-11
120.0	3.51049E-12	5.57132E-11
121.0	3.63226E-12	5.55991E-11
122.0	3.75608E-12	5.54861E-11
123.0	3.88193E-12	5.53742E-11
124.0	4.00981E-12	5.52634E-11
125.0	4.13968E-12	5.51537E-11

126.0	4.27153E-12	5.50451E-11
127.0	4.40533E-12	5.49374E-11
128.0	4.54106E-12	5.48309E-11
129.0	4.67870E-12	5.47253E-11
130.0	4.81823E-12	5.46208E-11
131.0	4.95963E-12	5.45173E-11
132.0	5.10286E-12	5.44147E-11
133.0	5.24791E-12	5.43132E-11
134.0	5.39475E-12	5.42126E-11
135.0	5.54336E-12	5.41129E-11
136.0	5.69371E-12	5.40142E-11
137.0	5.84578E-12	5.39164E-11
138.0	5.99955E-12	5.38196E-11
139.0	6.15498E-12	5.37236E-11
140.0	6.31206E-12	5.36285E-11
141.0	6.47076E-12	5.35343E-11
142.0	6.63105E-12	5.34410E-11
143.0	6.79290E-12	5.33485E-11
144.0	6.95630E-12	5.32568E-11
145.0	7.12122E-12	5.31660E-11
146.0	7.28763E-12	5.30760E-11
147.0	7.45550E-12	5.29868E-11
148.0	7.62481E-12	5.28985E-11
149.0	7.79554E-12	5.28109E-11
150.0	7.96765E-12	5.27240E-11
151.0	8.14113E-12	5.26380E-11
152.0	8.31595E-12	5.25526E-11
153.0	8.49208E-12	5.24681E-11
154.0	8.66950E-12	5.23842E-11
155.0	8.84818E-12	5.23011E-11
156.0	9.02809E-12	5.22187E-11
157.0	9.20922E-12	5.21370E-11
158.0	9.39154E-12	5.20560E-11
159.0	9.57502E-12	5.19757E-11
160.0	9.75965E-12	5.18960E-11
161.0	9.94538E-12	5.18170E-11
162.0	1.01322E-11	5.17387E-11
163.0	1.03201E-11	5.16610E-11
164.0	1.05090E-11	5.15840E-11
165.0	1.06990E-11	5.15075E-11
166.0	1.08899E-11	5.14317E-11
167.0	1.10818E-11	5.13565E-11
168.0	1.12747E-11	5.12819E-11
169.0	1.14685E-11	5.12079E-11
170.0	1.16632E-11	5.11345E-11
171.0	1.18588E-11	5.10616E-11
172.0	1.20552E-11	5.09894E-11
173.0	1.22525E-11	5.09176E-11
174.0	1.24506E-11	5.08465E-11
175.0	1.26494E-11	5.07758E-11

176.0	1.28491E-11	5.07057E-11
177.0	1.30495E-11	5.06362E-11
178.0	1.32506E-11	5.05671E-11
179.0	1.34524E-11	5.04986E-11
180.0	1.36550E-11	5.04306E-11
181.0	1.38582E-11	5.03631E-11
182.0	1.40620E-11	5.02961E-11
183.0	1.42664E-11	5.02295E-11
184.0	1.44715E-11	5.01634E-11
185.0	1.46772E-11	5.00979E-11
186.0	1.48834E-11	5.00327E-11
187.0	1.50901E-11	4.99681E-11
188.0	1.52974E-11	4.99039E-11
189.0	1.55053E-11	4.98401E-11
190.0	1.57136E-11	4.97768E-11
191.0	1.59223E-11	4.97139E-11
192.0	1.61316E-11	4.96514E-11
193.0	1.63412E-11	4.95894E-11
194.0	1.65513E-11	4.95278E-11
195.0	1.67618E-11	4.94666E-11
196.0	1.69727E-11	4.94058E-11
197.0	1.71840E-11	4.93454E-11
198.0	1.73956E-11	4.92854E-11
199.0	1.76076E-11	4.92258E-11
200.0	1.78198E-11	4.91665E-11
201.0	1.80324E-11	4.91077E-11
202.0	1.82453E-11	4.90492E-11
203.0	1.84584E-11	4.89911E-11
204.0	1.86718E-11	4.89334E-11
205.0	1.88855E-11	4.88760E-11
206.0	1.90994E-11	4.88190E-11
207.0	1.93135E-11	4.87623E-11
208.0	1.95278E-11	4.87060E-11
209.0	1.97422E-11	4.86500E-11
210.0	1.99569E-11	4.85944E-11
211.0	2.01717E-11	4.85391E-11
212.0	2.03867E-11	4.84841E-11
213.0	2.06018E-11	4.84294E-11
214.0	2.08170E-11	4.83751E-11
215.0	2.10323E-11	4.83211E-11
216.0	2.12477E-11	4.82674E-11
217.0	2.14632E-11	4.82140E-11
218.0	2.16788E-11	4.81609E-11
219.0	2.18944E-11	4.81081E-11
220.0	2.21101E-11	4.80556E-11
221.0	2.23258E-11	4.80034E-11
222.0	2.25415E-11	4.79515E-11
223.0	2.27572E-11	4.78999E-11
224.0	2.29730E-11	4.78485E-11
225.0	2.31887E-11	4.77975E-11

226.0	2.34044E-11	4.77467E-11
227.0	2.36201E-11	4.76962E-11
228.0	2.38357E-11	4.76460E-11
229.0	2.40513E-11	4.75960E-11
230.0	2.42668E-11	4.75463E-11
231.0	2.44822E-11	4.74968E-11
232.0	2.46975E-11	4.74477E-11
233.0	2.49128E-11	4.73987E-11
234.0	2.51280E-11	4.73501E-11
235.0	2.53430E-11	4.73016E-11
236.0	2.55579E-11	4.72534E-11
237.0	2.57727E-11	4.72055E-11
238.0	2.59874E-11	4.71578E-11
239.0	2.62019E-11	4.71103E-11
240.0	2.64162E-11	4.70631E-11
241.0	2.66304E-11	4.70161E-11
242.0	2.68444E-11	4.69694E-11
243.0	2.70583E-11	4.69228E-11
244.0	2.72719E-11	4.68765E-11
245.0	2.74854E-11	4.68304E-11
246.0	2.76986E-11	4.67846E-11
247.0	2.79116E-11	4.67389E-11
248.0	2.81245E-11	4.66935E-11
249.0	2.83371E-11	4.66483E-11
250.0	2.85494E-11	4.66033E-11
251.0	2.87616E-11	4.65585E-11
252.0	2.89734E-11	4.65139E-11
253.0	2.91851E-11	4.64695E-11
254.0	2.93964E-11	4.64253E-11
255.0	2.96076E-11	4.63813E-11
256.0	2.98184E-11	4.63375E-11
257.0	3.00289E-11	4.62940E-11
258.0	3.02392E-11	4.62506E-11
259.0	3.04492E-11	4.62074E-11
260.0	3.06589E-11	4.61644E-11
261.0	3.08683E-11	4.61215E-11
262.0	3.10774E-11	4.60789E-11
263.0	3.12862E-11	4.60365E-11
264.0	3.14946E-11	4.59942E-11
265.0	3.17028E-11	4.59521E-11
266.0	3.19106E-11	4.59102E-11
267.0	3.21181E-11	4.58685E-11
268.0	3.23252E-11	4.58270E-11
269.0	3.25321E-11	4.57856E-11
270.0	3.27385E-11	4.57444E-11
271.0	3.29446E-11	4.57034E-11
272.0	3.31504E-11	4.56625E-11
273.0	3.33558E-11	4.56218E-11
274.0	3.35608E-11	4.55813E-11
275.0	3.37655E-11	4.55410E-11

276.0	3.39698E-11	4.55008E-11
277.0	3.41738E-11	4.54608E-11
278.0	3.43773E-11	4.54209E-11
279.0	3.45805E-11	4.53812E-11
280.0	3.47833E-11	4.53417E-11
281.0	3.49857E-11	4.53023E-11
282.0	3.51877E-11	4.52631E-11
283.0	3.53893E-11	4.52240E-11
284.0	3.55905E-11	4.51851E-11
285.0	3.57913E-11	4.51464E-11
286.0	3.59917E-11	4.51077E-11
287.0	3.61917E-11	4.50693E-11
288.0	3.63913E-11	4.50310E-11
289.0	3.65905E-11	4.49928E-11
290.0	3.67893E-11	4.49548E-11
291.0	3.69876E-11	4.49169E-11
292.0	3.71855E-11	4.48792E-11
293.0	3.73830E-11	4.48416E-11
294.0	3.75801E-11	4.48042E-11
295.0	3.77767E-11	4.47669E-11
296.0	3.79729E-11	4.47297E-11
297.0	3.81687E-11	4.46927E-11
298.0	3.83641E-11	4.46558E-11
299.0	3.85590E-11	4.46191E-11
300.0	3.87534E-11	4.45825E-11
301.0	3.89475E-11	4.45460E-11
302.0	3.91410E-11	4.45097E-11
303.0	3.93342E-11	4.44735E-11
304.0	3.95268E-11	4.44374E-11
305.0	3.97191E-11	4.44014E-11
306.0	3.99109E-11	4.43656E-11
307.0	4.01022E-11	4.43299E-11
308.0	4.02931E-11	4.42944E-11
309.0	4.04835E-11	4.42590E-11
310.0	4.06735E-11	4.42237E-11
311.0	4.08630E-11	4.41885E-11
312.0	4.10520E-11	4.41534E-11
313.0	4.12406E-11	4.41185E-11
314.0	4.14287E-11	4.40837E-11
315.0	4.16164E-11	4.40490E-11
316.0	4.18036E-11	4.40145E-11
317.0	4.19903E-11	4.39801E-11
318.0	4.21766E-11	4.39457E-11
319.0	4.23624E-11	4.39115E-11
320.0	4.25478E-11	4.38775E-11
321.0	4.27326E-11	4.38435E-11
322.0	4.29170E-11	4.38097E-11
323.0	4.31009E-11	4.37760E-11
324.0	4.32844E-11	4.37423E-11
325.0	4.34674E-11	4.37089E-11

326.0	4.36499E-11	4.36755E-11
327.0	4.38319E-11	4.36422E-11
328.0	4.40135E-11	4.36091E-11
329.0	4.41946E-11	4.35760E-11
330.0	4.43752E-11	4.35431E-11
331.0	4.45553E-11	4.35103E-11
332.0	4.47350E-11	4.34776E-11
333.0	4.49142E-11	4.34450E-11
334.0	4.50929E-11	4.34125E-11
335.0	4.52711E-11	4.33801E-11
336.0	4.54489E-11	4.33479E-11
337.0	4.56262E-11	4.33157E-11
338.0	4.58030E-11	4.32836E-11
339.0	4.59793E-11	4.32517E-11
340.0	4.61552E-11	4.32199E-11
341.0	4.63306E-11	4.31881E-11
342.0	4.65055E-11	4.31565E-11
343.0	4.66799E-11	4.31250E-11
344.0	4.68538E-11	4.30935E-11
345.0	4.70273E-11	4.30622E-11
346.0	4.72003E-11	4.30310E-11
347.0	4.73728E-11	4.29999E-11
348.0	4.75448E-11	4.29689E-11
349.0	4.77164E-11	4.29380E-11
350.0	4.78875E-11	4.29071E-11
351.0	4.80581E-11	4.28764E-11
352.0	4.82282E-11	4.28458E-11
353.0	4.83979E-11	4.28153E-11
354.0	4.85670E-11	4.27849E-11
355.0	4.87357E-11	4.27546E-11
356.0	4.89040E-11	4.27243E-11
357.0	4.90717E-11	4.26942E-11
358.0	4.92390E-11	4.26642E-11
359.0	4.94058E-11	4.26342E-11
360.0	4.95722E-11	4.26044E-11
361.0	4.97380E-11	4.25747E-11
362.0	4.99034E-11	4.25450E-11
363.0	5.00683E-11	4.25155E-11
364.0	5.02328E-11	4.24860E-11
365.0	5.03968E-11	4.24566E-11
366.0	5.05603E-11	4.24274E-11
367.0	5.07233E-11	4.23982E-11
368.0	5.08859E-11	4.23691E-11
369.0	5.10480E-11	4.23401E-11
370.0	5.12096E-11	4.23112E-11
371.0	5.13708E-11	4.22824E-11
372.0	5.15315E-11	4.22536E-11
373.0	5.16917E-11	4.22250E-11
374.0	5.18515E-11	4.21964E-11
375.0	5.20108E-11	4.21680E-11

376.0	5.21697E-11	4.21396E-11
377.0	5.23281E-11	4.21113E-11
378.0	5.24860E-11	4.20831E-11
379.0	5.26434E-11	4.20550E-11
380.0	5.28004E-11	4.20270E-11
381.0	5.29570E-11	4.19991E-11
382.0	5.31131E-11	4.19712E-11
383.0	5.32687E-11	4.19435E-11
384.0	5.34239E-11	4.19158E-11
385.0	5.35786E-11	4.18882E-11
386.0	5.37328E-11	4.18607E-11
387.0	5.38866E-11	4.18333E-11
388.0	5.40400E-11	4.18059E-11
389.0	5.41929E-11	4.17787E-11
390.0	5.43453E-11	4.17515E-11
391.0	5.44973E-11	4.17244E-11
392.0	5.46489E-11	4.16974E-11
393.0	5.48000E-11	4.16705E-11
394.0	5.49506E-11	4.16437E-11
395.0	5.51009E-11	4.16169E-11
396.0	5.52506E-11	4.15902E-11
397.0	5.53999E-11	4.15636E-11
398.0	5.55488E-11	4.15371E-11
399.0	5.56972E-11	4.15107E-11
400.0	5.58452E-11	4.14843E-11
401.0	5.59928E-11	4.14581E-11
402.0	5.61399E-11	4.14319E-11
403.0	5.62866E-11	4.14057E-11
404.0	5.64328E-11	4.13797E-11
405.0	5.65786E-11	4.13537E-11
406.0	5.67240E-11	4.13279E-11
407.0	5.68689E-11	4.13021E-11
408.0	5.70134E-11	4.12763E-11
409.0	5.71575E-11	4.12507E-11
410.0	5.73011E-11	4.12251E-11
411.0	5.74443E-11	4.11996E-11
412.0	5.75871E-11	4.11742E-11
413.0	5.77294E-11	4.11488E-11
414.0	5.78714E-11	4.11236E-11
415.0	5.80129E-11	4.10984E-11
416.0	5.81539E-11	4.10733E-11
417.0	5.82946E-11	4.10482E-11
418.0	5.84348E-11	4.10232E-11
419.0	5.85746E-11	4.09983E-11
420.0	5.87140E-11	4.09735E-11
421.0	5.88530E-11	4.09488E-11
422.0	5.89916E-11	4.09241E-11
423.0	5.91297E-11	4.08995E-11
424.0	5.92674E-11	4.08750E-11
425.0	5.94047E-11	4.08505E-11

426.0	5.95416E-11	4.08261E-11
427.0	5.96781E-11	4.08018E-11
428.0	5.98142E-11	4.07776E-11
429.0	5.99499E-11	4.07534E-11
430.0	6.00851E-11	4.07293E-11
431.0	6.02200E-11	4.07053E-11
432.0	6.03544E-11	4.06813E-11
433.0	6.04885E-11	4.06574E-11
434.0	6.06221E-11	4.06336E-11
435.0	6.07554E-11	4.06098E-11
436.0	6.08882E-11	4.05861E-11
437.0	6.10206E-11	4.05625E-11
438.0	6.11527E-11	4.05390E-11
439.0	6.12843E-11	4.05155E-11
440.0	6.14156E-11	4.04921E-11
441.0	6.15464E-11	4.04688E-11
442.0	6.16769E-11	4.04455E-11
443.0	6.18070E-11	4.04223E-11
444.0	6.19366E-11	4.03992E-11
445.0	6.20659E-11	4.03761E-11
446.0	6.21948E-11	4.03531E-11
447.0	6.23233E-11	4.03301E-11
448.0	6.24515E-11	4.03073E-11
449.0	6.25792E-11	4.02845E-11
450.0	6.27066E-11	4.02617E-11
451.0	6.28335E-11	4.02390E-11
452.0	6.29601E-11	4.02164E-11
453.0	6.30863E-11	4.01939E-11
454.0	6.32122E-11	4.01714E-11
455.0	6.33376E-11	4.01490E-11
456.0	6.34627E-11	4.01266E-11
457.0	6.35874E-11	4.01044E-11
458.0	6.37117E-11	4.00821E-11
459.0	6.38357E-11	4.00600E-11
460.0	6.39593E-11	4.00379E-11
461.0	6.40825E-11	4.00158E-11
462.0	6.42053E-11	3.99939E-11
463.0	6.43278E-11	3.99719E-11
464.0	6.44499E-11	3.99501E-11
465.0	6.45717E-11	3.99283E-11
466.0	6.46930E-11	3.99066E-11
467.0	6.48140E-11	3.98849E-11
468.0	6.49347E-11	3.98633E-11
469.0	6.50550E-11	3.98418E-11
470.0	6.51749E-11	3.98203E-11
471.0	6.52945E-11	3.97989E-11
472.0	6.54137E-11	3.97775E-11
473.0	6.55326E-11	3.97562E-11
474.0	6.56511E-11	3.97350E-11
475.0	6.57692E-11	3.97138E-11

476.0	6.58870E-11	3.96927E-11
477.0	6.60044E-11	3.96716E-11
478.0	6.61215E-11	3.96506E-11
479.0	6.62383E-11	3.96297E-11
480.0	6.63546E-11	3.96088E-11
481.0	6.64707E-11	3.95879E-11
482.0	6.65864E-11	3.95672E-11
483.0	6.67017E-11	3.95465E-11
484.0	6.68168E-11	3.95258E-11
485.0	6.69314E-11	3.95052E-11
486.0	6.70457E-11	3.94847E-11
487.0	6.71597E-11	3.94642E-11
488.0	6.72734E-11	3.94437E-11
489.0	6.73867E-11	3.94234E-11
490.0	6.74996E-11	3.94030E-11
491.0	6.76123E-11	3.93828E-11
492.0	6.77246E-11	3.93626E-11
493.0	6.78365E-11	3.93424E-11
494.0	6.79482E-11	3.93223E-11
495.0	6.80595E-11	3.93023E-11
496.0	6.81704E-11	3.92823E-11
497.0	6.82811E-11	3.92624E-11
498.0	6.83914E-11	3.92425E-11
499.0	6.85014E-11	3.92227E-11
500.0	6.86110E-11	3.92029E-11

Table S8: Computed excitation rate coefficients in cm^3 $molecule^{-1}$ s^{-1} units for a series of inelastic processes generated using the 4D RR-PES for the $HeH^+(j_1)\cdot\cdot\cdot$ ortho-$H_2(j_2 = 1)$ system with $\Delta j_1 = +1$ and $\Delta j_1 = +2$.

T (K)	01->11	01->21	11->21	11->31
5.0	5.35737E-18	2.10355E-35	5.68072E-27	3.86850E-52
6.0	1.15077E-16	3.65641E-31	3.96298E-24	3.95615E-45
7.0	1.04054E-15	3.89897E-28	4.25097E-22	4.01639E-40
8.0	5.52122E-15	7.32691E-26	1.42695E-20	2.30197E-36
9.0	2.05479E-14	4.34038E-24	2.21398E-19	1.94686E-33
10.0	5.95280E-14	1.14603E-22	2.00123E-18	4.31300E-31
11.0	1.43359E-13	1.67952E-21	1.21980E-17	3.60239E-29
12.0	2.99908E-13	1.58091E-20	5.52574E-17	1.44582E-27
13.0	5.62125E-13	1.05750E-19	1.99013E-16	3.29856E-26
14.0	9.65418E-13	5.40458E-19	5.98078E-16	4.82431E-25
15.0	1.54497E-12	2.22577E-18	1.55414E-15	4.94098E-24
16.0	2.33345E-12	7.68862E-18	3.58712E-15	3.78700E-23
17.0	3.35937E-12	2.29731E-17	7.50742E-15	2.28550E-22
18.0	4.64604E-12	6.08154E-17	1.44787E-14	1.13004E-21
19.0	6.21119E-12	1.45370E-16	2.60624E-14	4.72331E-21
20.0	8.06691E-12	3.18575E-16	4.42390E-14	1.71140E-20
21.0	1.02200E-11	6.48020E-16	7.14038E-14	5.48591E-20
22.0	1.26726E-11	1.23594E-15	1.10339E-13	1.58190E-19
23.0	1.54224E-11	2.22881E-15	1.64168E-13	4.16036E-19
24.0	1.84639E-11	3.82688E-15	2.36291E-13	1.00947E-18

25.0	2.17885E-11	6.29307E-15	3.30317E-13	2.28173E-18
26.0	2.53852E-11	9.96057E-15	4.49987E-13	4.84386E-18
27.0	2.92417E-11	1.52387E-14	5.99103E-13	9.72528E-18
28.0	3.33438E-11	2.26170E-14	7.81452E-13	1.85776E-17
29.0	3.76767E-11	3.26664E-14	1.00075E-12	3.39385E-17
30.0	4.22253E-11	4.60393E-14	1.26056E-12	5.95591E-17
31.0	4.69737E-11	6.34665E-14	1.56430E-12	1.00796E-16
32.0	5.19065E-11	8.57527E-14	1.91514E-12	1.65067E-16
33.0	5.70080E-11	1.13770E-13	2.31599E-12	2.62358E-16
34.0	6.22633E-11	1.48453E-13	2.76953E-12	4.05780E-16
35.0	6.76575E-11	1.90783E-13	3.27810E-12	6.12160E-16
36.0	7.31765E-11	2.41789E-13	3.84378E-12	9.02650E-16
37.0	7.88066E-11	3.02529E-13	4.46835E-12	1.30334E-15
38.0	8.45348E-11	3.74086E-13	5.15326E-12	1.84587E-15
39.0	9.03489E-11	4.57554E-13	5.89972E-12	2.56800E-15
40.0	9.62370E-11	5.54032E-13	6.70862E-12	3.51415E-15
41.0	1.02188E-10	6.64612E-13	7.58059E-12	4.73588E-15
42.0	1.08192E-10	7.90374E-13	8.51603E-12	6.29234E-15
43.0	1.14239E-10	9.32375E-13	9.51506E-12	8.25056E-15
44.0	1.20321E-10	1.09164E-12	1.05776E-11	1.06858E-14
45.0	1.26427E-10	1.26917E-12	1.17034E-11	1.36816E-14
46.0	1.32552E-10	1.46591E-12	1.28919E-11	1.73302E-14
47.0	1.38687E-10	1.68278E-12	1.41426E-11	2.17319E-14
48.0	1.44825E-10	1.92062E-12	1.54545E-11	2.69959E-14
49.0	1.50961E-10	2.18025E-12	1.68268E-11	3.32393E-14
50.0	1.57089E-10	2.46242E-12	1.82584E-11	4.05874E-14
51.0	1.63203E-10	2.76783E-12	1.97480E-11	4.91731E-14
52.0	1.69299E-10	3.09710E-12	2.12945E-11	5.91367E-14
53.0	1.75372E-10	3.45082E-12	2.28963E-11	7.06254E-14
54.0	1.81418E-10	3.82951E-12	2.45522E-11	8.37929E-14
55.0	1.87433E-10	4.23362E-12	2.62606E-11	9.87987E-14
56.0	1.93414E-10	4.66357E-12	2.80199E-11	1.15808E-13
57.0	1.99359E-10	5.11969E-12	2.98285E-11	1.34990E-13
58.0	2.05263E-10	5.60227E-12	3.16849E-11	1.56518E-13
59.0	2.11126E-10	6.11155E-12	3.35873E-11	1.80570E-13
60.0	2.16943E-10	6.64771E-12	3.55341E-11	2.07327E-13
61.0	2.22715E-10	7.21088E-12	3.75237E-11	2.36970E-13
62.0	2.28438E-10	7.80115E-12	3.95543E-11	2.69684E-13
63.0	2.34111E-10	8.41853E-12	4.16242E-11	3.05653E-13
64.0	2.39733E-10	9.06303E-12	4.37319E-11	3.45063E-13
65.0	2.45303E-10	9.73460E-12	4.58755E-11	3.88099E-13
66.0	2.50819E-10	1.04331E-11	4.80535E-11	4.34945E-13
67.0	2.56281E-10	1.11585E-11	5.02643E-11	4.85785E-13
68.0	2.61688E-10	1.19105E-11	5.25063E-11	5.40799E-13
69.0	2.67039E-10	1.26890E-11	5.47777E-11	6.00166E-13
70.0	2.72333E-10	1.34937E-11	5.70772E-11	6.64062E-13
71.0	2.77571E-10	1.43244E-11	5.94032E-11	7.32659E-13
72.0	2.82753E-10	1.51808E-11	6.17541E-11	8.06126E-13
73.0	2.87877E-10	1.60625E-11	6.41286E-11	8.84627E-13
74.0	2.92943E-10	1.69692E-11	6.65251E-11	9.68323E-13

75.0	2.97952E-10	1.79006E-11	6.89423E-11	1.05737E-12
76.0	3.02904E-10	1.88563E-11	7.13788E-11	1.15192E-12
77.0	3.07799E-10	1.98358E-11	7.38332E-11	1.25211E-12
78.0	3.12637E-10	2.08389E-11	7.63044E-11	1.35808E-12
79.0	3.17417E-10	2.18649E-11	7.87910E-11	1.46998E-12
80.0	3.22141E-10	2.29136E-11	8.12917E-11	1.58792E-12
81.0	3.26809E-10	2.39844E-11	8.38055E-11	1.71203E-12
82.0	3.31421E-10	2.50769E-11	8.63311E-11	1.84242E-12
83.0	3.35977E-10	2.61906E-11	8.88675E-11	1.97920E-12
84.0	3.40477E-10	2.73251E-11	9.14135E-11	2.12248E-12
85.0	3.44923E-10	2.84798E-11	9.39681E-11	2.27235E-12
86.0	3.49315E-10	2.96542E-11	9.65303E-11	2.42890E-12
87.0	3.53652E-10	3.08480E-11	9.90991E-11	2.59222E-12
88.0	3.57936E-10	3.20605E-11	1.01674E-10	2.76238E-12
89.0	3.62168E-10	3.32913E-11	1.04253E-10	2.93945E-12
90.0	3.66347E-10	3.45398E-11	1.06836E-10	3.12350E-12
91.0	3.70474E-10	3.58057E-11	1.09422E-10	3.31459E-12
92.0	3.74550E-10	3.70883E-11	1.12010E-10	3.51276E-12
93.0	3.78575E-10	3.83872E-11	1.14600E-10	3.71807E-12
94.0	3.82550E-10	3.97019E-11	1.17190E-10	3.93055E-12
95.0	3.86476E-10	4.10318E-11	1.19780E-10	4.15024E-12
96.0	3.90353E-10	4.23766E-11	1.22370E-10	4.37716E-12
97.0	3.94182E-10	4.37357E-11	1.24958E-10	4.61134E-12
98.0	3.97963E-10	4.51086E-11	1.27544E-10	4.85279E-12
99.0	4.01696E-10	4.64948E-11	1.30127E-10	5.10153E-12
100.0	4.05384E-10	4.78940E-11	1.32707E-10	5.35755E-12
101.0	4.09025E-10	4.93055E-11	1.35283E-10	5.62087E-12
102.0	4.12622E-10	5.07289E-11	1.37854E-10	5.89148E-12
103.0	4.16173E-10	5.21639E-11	1.40421E-10	6.16936E-12
104.0	4.19681E-10	5.36099E-11	1.42982E-10	6.45451E-12
105.0	4.23145E-10	5.50664E-11	1.45537E-10	6.74691E-12
106.0	4.26567E-10	5.65331E-11	1.48085E-10	7.04653E-12
107.0	4.29946E-10	5.80095E-11	1.50627E-10	7.35334E-12
108.0	4.33284E-10	5.94952E-11	1.53162E-10	7.66733E-12
109.0	4.36580E-10	6.09898E-11	1.55688E-10	7.98844E-12
110.0	4.39836E-10	6.24927E-11	1.58207E-10	8.31664E-12
111.0	4.43053E-10	6.40038E-11	1.60717E-10	8.65189E-12
112.0	4.46230E-10	6.55225E-11	1.63219E-10	8.99415E-12
113.0	4.49368E-10	6.70484E-11	1.65712E-10	9.34336E-12
114.0	4.52468E-10	6.85812E-11	1.68195E-10	9.69948E-12
115.0	4.55531E-10	7.01205E-11	1.70668E-10	1.00624E-11
116.0	4.58557E-10	7.16659E-11	1.73131E-10	1.04322E-11
117.0	4.61546E-10	7.32171E-11	1.75584E-10	1.08087E-11
118.0	4.64499E-10	7.47737E-11	1.78027E-10	1.11918E-11
119.0	4.67417E-10	7.63353E-11	1.80459E-10	1.15816E-11
120.0	4.70300E-10	7.79017E-11	1.82880E-10	1.19778E-11
121.0	4.73148E-10	7.94725E-11	1.85290E-10	1.23806E-11
122.0	4.75963E-10	8.10473E-11	1.87688E-10	1.27897E-11
123.0	4.78744E-10	8.26259E-11	1.90075E-10	1.32052E-11
124.0	4.81493E-10	8.42079E-11	1.92450E-10	1.36268E-11

125.0	4.84209E-10	8.57930E-11	1.94813E-10	1.40547E-11
126.0	4.86893E-10	8.73810E-11	1.97164E-10	1.44886E-11
127.0	4.89546E-10	8.89715E-11	1.99503E-10	1.49285E-11
128.0	4.92169E-10	9.05643E-11	2.01830E-10	1.53744E-11
129.0	4.94760E-10	9.21590E-11	2.04144E-10	1.58261E-11
130.0	4.97322E-10	9.37555E-11	2.06445E-10	1.62835E-11
131.0	4.99854E-10	9.53535E-11	2.08734E-10	1.67467E-11
132.0	5.02357E-10	9.69526E-11	2.11010E-10	1.72154E-11
133.0	5.04832E-10	9.85527E-11	2.13274E-10	1.76896E-11
134.0	5.07278E-10	1.00154E-10	2.15524E-10	1.81692E-11
135.0	5.09697E-10	1.01755E-10	2.17761E-10	1.86541E-11
136.0	5.12088E-10	1.03356E-10	2.19985E-10	1.91443E-11
137.0	5.14452E-10	1.04958E-10	2.22196E-10	1.96396E-11
138.0	5.16790E-10	1.06559E-10	2.24394E-10	2.01399E-11
139.0	5.19102E-10	1.08160E-10	2.26579E-10	2.06453E-11
140.0	5.21387E-10	1.09761E-10	2.28750E-10	2.11555E-11
141.0	5.23648E-10	1.11360E-10	2.30908E-10	2.16705E-11
142.0	5.25883E-10	1.12959E-10	2.33052E-10	2.21901E-11
143.0	5.28094E-10	1.14556E-10	2.35183E-10	2.27144E-11
144.0	5.30281E-10	1.16152E-10	2.37301E-10	2.32432E-11
145.0	5.32444E-10	1.17746E-10	2.39405E-10	2.37765E-11
146.0	5.34583E-10	1.19339E-10	2.41496E-10	2.43140E-11
147.0	5.36699E-10	1.20930E-10	2.43574E-10	2.48558E-11
148.0	5.38793E-10	1.22519E-10	2.45638E-10	2.54018E-11
149.0	5.40863E-10	1.24105E-10	2.47688E-10	2.59518E-11
150.0	5.42912E-10	1.25689E-10	2.49726E-10	2.65058E-11
151.0	5.44939E-10	1.27271E-10	2.51749E-10	2.70637E-11
152.0	5.46944E-10	1.28850E-10	2.53760E-10	2.76254E-11
153.0	5.48928E-10	1.30426E-10	2.55757E-10	2.81908E-11
154.0	5.50892E-10	1.31999E-10	2.57741E-10	2.87599E-11
155.0	5.52834E-10	1.33569E-10	2.59711E-10	2.93324E-11
156.0	5.54757E-10	1.35136E-10	2.61668E-10	2.99085E-11
157.0	5.56659E-10	1.36700E-10	2.63612E-10	3.04878E-11
158.0	5.58542E-10	1.38260E-10	2.65542E-10	3.10705E-11
159.0	5.60406E-10	1.39817E-10	2.67460E-10	3.16564E-11
160.0	5.62250E-10	1.41370E-10	2.69364E-10	3.22454E-11
161.0	5.64075E-10	1.42919E-10	2.71255E-10	3.28374E-11
162.0	5.65882E-10	1.44464E-10	2.73133E-10	3.34323E-11
163.0	5.67671E-10	1.46006E-10	2.74998E-10	3.40301E-11
164.0	5.69441E-10	1.47543E-10	2.76851E-10	3.46307E-11
165.0	5.71194E-10	1.49076E-10	2.78690E-10	3.52341E-11
166.0	5.72929E-10	1.50605E-10	2.80516E-10	3.58400E-11
167.0	5.74647E-10	1.52130E-10	2.82330E-10	3.64485E-11
168.0	5.76348E-10	1.53650E-10	2.84130E-10	3.70594E-11
169.0	5.78032E-10	1.55166E-10	2.85919E-10	3.76728E-11
170.0	5.79700E-10	1.56677E-10	2.87694E-10	3.82884E-11
171.0	5.81351E-10	1.58183E-10	2.89457E-10	3.89063E-11
172.0	5.82986E-10	1.59685E-10	2.91207E-10	3.95264E-11
173.0	5.84606E-10	1.61182E-10	2.92945E-10	4.01486E-11
174.0	5.86209E-10	1.62674E-10	2.94671E-10	4.07727E-11

175.0	5.87798E-10	1.64162E-10	2.96384E-10	4.13989E-11
176.0	5.89371E-10	1.65644E-10	2.98085E-10	4.20269E-11
177.0	5.90929E-10	1.67121E-10	2.99774E-10	4.26567E-11
178.0	5.92472E-10	1.68594E-10	3.01451E-10	4.32882E-11
179.0	5.94001E-10	1.70061E-10	3.03115E-10	4.39215E-11
180.0	5.95515E-10	1.71523E-10	3.04768E-10	4.45563E-11
181.0	5.97015E-10	1.72979E-10	3.06409E-10	4.51926E-11
182.0	5.98501E-10	1.74431E-10	3.08038E-10	4.58305E-11
183.0	5.99973E-10	1.75877E-10	3.09655E-10	4.64697E-11
184.0	6.01432E-10	1.77318E-10	3.11260E-10	4.71103E-11
185.0	6.02877E-10	1.78753E-10	3.12854E-10	4.77521E-11
186.0	6.04309E-10	1.80183E-10	3.14437E-10	4.83952E-11
187.0	6.05728E-10	1.81608E-10	3.16007E-10	4.90394E-11
188.0	6.07134E-10	1.83027E-10	3.17567E-10	4.96847E-11
189.0	6.08527E-10	1.84440E-10	3.19115E-10	5.03311E-11
190.0	6.09908E-10	1.85848E-10	3.20652E-10	5.09784E-11
191.0	6.11276E-10	1.87251E-10	3.22178E-10	5.16266E-11
192.0	6.12632E-10	1.88648E-10	3.23692E-10	5.22758E-11
193.0	6.13976E-10	1.90039E-10	3.25196E-10	5.29257E-11
194.0	6.15308E-10	1.91424E-10	3.26689E-10	5.35764E-11
195.0	6.16629E-10	1.92804E-10	3.28171E-10	5.42277E-11
196.0	6.17938E-10	1.94178E-10	3.29642E-10	5.48797E-11
197.0	6.19235E-10	1.95547E-10	3.31102E-10	5.55323E-11
198.0	6.20521E-10	1.96909E-10	3.32552E-10	5.61855E-11
199.0	6.21796E-10	1.98266E-10	3.33991E-10	5.68391E-11
200.0	6.23060E-10	1.99618E-10	3.35419E-10	5.74932E-11
201.0	6.24312E-10	2.00963E-10	3.36838E-10	5.81477E-11
202.0	6.25555E-10	2.02303E-10	3.38245E-10	5.88025E-11
203.0	6.26786E-10	2.03637E-10	3.39643E-10	5.94576E-11
204.0	6.28008E-10	2.04965E-10	3.41031E-10	6.01129E-11
205.0	6.29218E-10	2.06287E-10	3.42408E-10	6.07685E-11
206.0	6.30419E-10	2.07604E-10	3.43775E-10	6.14242E-11
207.0	6.31610E-10	2.08915E-10	3.45133E-10	6.20800E-11
208.0	6.32790E-10	2.10219E-10	3.46480E-10	6.27359E-11
209.0	6.33961E-10	2.11519E-10	3.47818E-10	6.33918E-11
210.0	6.35122E-10	2.12812E-10	3.49146E-10	6.40478E-11
211.0	6.36274E-10	2.14099E-10	3.50464E-10	6.47036E-11
212.0	6.37416E-10	2.15381E-10	3.51773E-10	6.53594E-11
213.0	6.38549E-10	2.16657E-10	3.53072E-10	6.60150E-11
214.0	6.39672E-10	2.17927E-10	3.54361E-10	6.66704E-11
215.0	6.40787E-10	2.19191E-10	3.55642E-10	6.73257E-11
216.0	6.41892E-10	2.20449E-10	3.56913E-10	6.79807E-11
217.0	6.42989E-10	2.21702E-10	3.58175E-10	6.86354E-11
218.0	6.44077E-10	2.22948E-10	3.59427E-10	6.92897E-11
219.0	6.45156E-10	2.24189E-10	3.60671E-10	6.99438E-11
220.0	6.46226E-10	2.25424E-10	3.61906E-10	7.05974E-11
221.0	6.47289E-10	2.26654E-10	3.63131E-10	7.12506E-11
222.0	6.48342E-10	2.27877E-10	3.64348E-10	7.19033E-11
223.0	6.49388E-10	2.29095E-10	3.65556E-10	7.25556E-11
224.0	6.50425E-10	2.30307E-10	3.66756E-10	7.32073E-11

225.0	6.51454E-10	2.31513E-10	3.67946E-10	7.38584E-11
226.0	6.52476E-10	2.32714E-10	3.69129E-10	7.45090E-11
227.0	6.53489E-10	2.33908E-10	3.70302E-10	7.51590E-11
228.0	6.54495E-10	2.35098E-10	3.71467E-10	7.58083E-11
229.0	6.55493E-10	2.36281E-10	3.72624E-10	7.64569E-11
230.0	6.56483E-10	2.37459E-10	3.73773E-10	7.71048E-11
231.0	6.57466E-10	2.38631E-10	3.74913E-10	7.77520E-11
232.0	6.58441E-10	2.39797E-10	3.76045E-10	7.83985E-11
233.0	6.59409E-10	2.40957E-10	3.77169E-10	7.90441E-11
234.0	6.60370E-10	2.42112E-10	3.78285E-10	7.96890E-11
235.0	6.61323E-10	2.43262E-10	3.79393E-10	8.03330E-11
236.0	6.62270E-10	2.44406E-10	3.80493E-10	8.09761E-11
237.0	6.63209E-10	2.45544E-10	3.81585E-10	8.16184E-11
238.0	6.64142E-10	2.46676E-10	3.82670E-10	8.22597E-11
239.0	6.65068E-10	2.47803E-10	3.83746E-10	8.29001E-11
240.0	6.65987E-10	2.48925E-10	3.84815E-10	8.35396E-11
241.0	6.66899E-10	2.50041E-10	3.85877E-10	8.41781E-11
242.0	6.67805E-10	2.51151E-10	3.86931E-10	8.48155E-11
243.0	6.68704E-10	2.52256E-10	3.87977E-10	8.54520E-11
244.0	6.69596E-10	2.53356E-10	3.89017E-10	8.60874E-11
245.0	6.70482E-10	2.54450E-10	3.90048E-10	8.67218E-11
246.0	6.71362E-10	2.55538E-10	3.91073E-10	8.73550E-11
247.0	6.72236E-10	2.56621E-10	3.92090E-10	8.79872E-11
248.0	6.73103E-10	2.57699E-10	3.93100E-10	8.86182E-11
249.0	6.73965E-10	2.58771E-10	3.94103E-10	8.92482E-11
250.0	6.74820E-10	2.59838E-10	3.95099E-10	8.98769E-11
251.0	6.75669E-10	2.60900E-10	3.96088E-10	9.05045E-11
252.0	6.76512E-10	2.61956E-10	3.97070E-10	9.11309E-11
253.0	6.77350E-10	2.63007E-10	3.98046E-10	9.17560E-11
254.0	6.78181E-10	2.64053E-10	3.99014E-10	9.23800E-11
255.0	6.79007E-10	2.65094E-10	3.99976E-10	9.30027E-11
256.0	6.79828E-10	2.66129E-10	4.00931E-10	9.36242E-11
257.0	6.80642E-10	2.67159E-10	4.01879E-10	9.42444E-11
258.0	6.81451E-10	2.68184E-10	4.02821E-10	9.48633E-11
259.0	6.82255E-10	2.69203E-10	4.03756E-10	9.54809E-11
260.0	6.83053E-10	2.70218E-10	4.04685E-10	9.60972E-11
261.0	6.83846E-10	2.71227E-10	4.05607E-10	9.67121E-11
262.0	6.84633E-10	2.72231E-10	4.06523E-10	9.73257E-11
263.0	6.85415E-10	2.73230E-10	4.07433E-10	9.79380E-11
264.0	6.86192E-10	2.74224E-10	4.08336E-10	9.85489E-11
265.0	6.86964E-10	2.75213E-10	4.09233E-10	9.91585E-11
266.0	6.87731E-10	2.76197E-10	4.10124E-10	9.97666E-11
267.0	6.88492E-10	2.77176E-10	4.11009E-10	1.00373E-10
268.0	6.89249E-10	2.78150E-10	4.11888E-10	1.00979E-10
269.0	6.90000E-10	2.79119E-10	4.12761E-10	1.01583E-10
270.0	6.90747E-10	2.80083E-10	4.13628E-10	1.02185E-10
271.0	6.91489E-10	2.81042E-10	4.14489E-10	1.02786E-10
272.0	6.92226E-10	2.81996E-10	4.15344E-10	1.03386E-10
273.0	6.92959E-10	2.82946E-10	4.16194E-10	1.03984E-10
274.0	6.93686E-10	2.83890E-10	4.17037E-10	1.04580E-10

275.0	6.94409E-10	2.84830E-10	4.17875E-10	1.05176E-10
276.0	6.95128E-10	2.85765E-10	4.18707E-10	1.05769E-10
277.0	6.95842E-10	2.86695E-10	4.19534E-10	1.06361E-10
278.0	6.96551E-10	2.87620E-10	4.20355E-10	1.06952E-10
279.0	6.97256E-10	2.88540E-10	4.21171E-10	1.07541E-10
280.0	6.97956E-10	2.89456E-10	4.21981E-10	1.08129E-10
281.0	6.98652E-10	2.90367E-10	4.22786E-10	1.08715E-10
282.0	6.99344E-10	2.91274E-10	4.23585E-10	1.09300E-10
283.0	7.00031E-10	2.92176E-10	4.24379E-10	1.09883E-10
284.0	7.00715E-10	2.93073E-10	4.25168E-10	1.10464E-10
285.0	7.01394E-10	2.93966E-10	4.25951E-10	1.11044E-10
286.0	7.02068E-10	2.94854E-10	4.26729E-10	1.11623E-10
287.0	7.02739E-10	2.95737E-10	4.27502E-10	1.12199E-10
288.0	7.03406E-10	2.96616E-10	4.28270E-10	1.12775E-10
289.0	7.04068E-10	2.97490E-10	4.29033E-10	1.13348E-10
290.0	7.04727E-10	2.98360E-10	4.29791E-10	1.13920E-10
291.0	7.05381E-10	2.99226E-10	4.30544E-10	1.14491E-10
292.0	7.06032E-10	3.00087E-10	4.31292E-10	1.15060E-10
293.0	7.06678E-10	3.00943E-10	4.32035E-10	1.15627E-10
294.0	7.07321E-10	3.01795E-10	4.32773E-10	1.16193E-10
295.0	7.07960E-10	3.02643E-10	4.33506E-10	1.16757E-10
296.0	7.08595E-10	3.03486E-10	4.34235E-10	1.17319E-10
297.0	7.09227E-10	3.04325E-10	4.34959E-10	1.17880E-10
298.0	7.09855E-10	3.05160E-10	4.35678E-10	1.18440E-10
299.0	7.10479E-10	3.05991E-10	4.36392E-10	1.18997E-10
300.0	7.11099E-10	3.06817E-10	4.37102E-10	1.19553E-10
301.0	7.11716E-10	3.07639E-10	4.37807E-10	1.20108E-10
302.0	7.12329E-10	3.08456E-10	4.38508E-10	1.20661E-10
303.0	7.12939E-10	3.09270E-10	4.39204E-10	1.21212E-10
304.0	7.13545E-10	3.10079E-10	4.39895E-10	1.21761E-10
305.0	7.14147E-10	3.10884E-10	4.40583E-10	1.22309E-10
306.0	7.14747E-10	3.11685E-10	4.41265E-10	1.22856E-10
307.0	7.15342E-10	3.12482E-10	4.41944E-10	1.23400E-10
308.0	7.15935E-10	3.13275E-10	4.42618E-10	1.23943E-10
309.0	7.16524E-10	3.14064E-10	4.43288E-10	1.24485E-10
310.0	7.17110E-10	3.14849E-10	4.43953E-10	1.25024E-10
311.0	7.17692E-10	3.15629E-10	4.44615E-10	1.25562E-10
312.0	7.18271E-10	3.16406E-10	4.45272E-10	1.26099E-10
313.0	7.18847E-10	3.17179E-10	4.45925E-10	1.26634E-10
314.0	7.19420E-10	3.17947E-10	4.46574E-10	1.27167E-10
315.0	7.19989E-10	3.18712E-10	4.47218E-10	1.27698E-10
316.0	7.20556E-10	3.19473E-10	4.47859E-10	1.28228E-10
317.0	7.21119E-10	3.20230E-10	4.48496E-10	1.28756E-10
318.0	7.21679E-10	3.20983E-10	4.49128E-10	1.29283E-10
319.0	7.22236E-10	3.21732E-10	4.49757E-10	1.29808E-10
320.0	7.22790E-10	3.22477E-10	4.50382E-10	1.30331E-10
321.0	7.23341E-10	3.23219E-10	4.51003E-10	1.30853E-10
322.0	7.23889E-10	3.23956E-10	4.51620E-10	1.31373E-10
323.0	7.24434E-10	3.24690E-10	4.52233E-10	1.31891E-10
324.0	7.24976E-10	3.25421E-10	4.52842E-10	1.32408E-10

325.0	7.25515E-10	3.26147E-10	4.53448E-10	1.32923E-10
326.0	7.26052E-10	3.26870E-10	4.54050E-10	1.33436E-10
327.0	7.26585E-10	3.27589E-10	4.54648E-10	1.33948E-10
328.0	7.27116E-10	3.28304E-10	4.55242E-10	1.34458E-10
329.0	7.27643E-10	3.29016E-10	4.55833E-10	1.34966E-10
330.0	7.28168E-10	3.29724E-10	4.56421E-10	1.35473E-10
331.0	7.28691E-10	3.30428E-10	4.57004E-10	1.35978E-10
332.0	7.29210E-10	3.31129E-10	4.57584E-10	1.36482E-10
333.0	7.29727E-10	3.31826E-10	4.58161E-10	1.36984E-10
334.0	7.30241E-10	3.32520E-10	4.58734E-10	1.37484E-10
335.0	7.30752E-10	3.33210E-10	4.59303E-10	1.37982E-10
336.0	7.31261E-10	3.33896E-10	4.59869E-10	1.38479E-10
337.0	7.31767E-10	3.34580E-10	4.60432E-10	1.38975E-10
338.0	7.32270E-10	3.35259E-10	4.60991E-10	1.39468E-10
339.0	7.32771E-10	3.35935E-10	4.61547E-10	1.39961E-10
340.0	7.33269E-10	3.36608E-10	4.62100E-10	1.40451E-10
341.0	7.33765E-10	3.37277E-10	4.62649E-10	1.40940E-10
342.0	7.34258E-10	3.37943E-10	4.63195E-10	1.41427E-10
343.0	7.34749E-10	3.38606E-10	4.63737E-10	1.41913E-10
344.0	7.35237E-10	3.39265E-10	4.64277E-10	1.42397E-10
345.0	7.35723E-10	3.39920E-10	4.64813E-10	1.42879E-10
346.0	7.36206E-10	3.40573E-10	4.65346E-10	1.43360E-10
347.0	7.36687E-10	3.41222E-10	4.65876E-10	1.43839E-10
348.0	7.37165E-10	3.41868E-10	4.66402E-10	1.44316E-10
349.0	7.37641E-10	3.42510E-10	4.66926E-10	1.44792E-10
350.0	7.38115E-10	3.43149E-10	4.67447E-10	1.45266E-10
351.0	7.38586E-10	3.43785E-10	4.67964E-10	1.45739E-10
352.0	7.39055E-10	3.44418E-10	4.68478E-10	1.46210E-10
353.0	7.39522E-10	3.45048E-10	4.68990E-10	1.46680E-10
354.0	7.39986E-10	3.45674E-10	4.69498E-10	1.47148E-10
355.0	7.40448E-10	3.46297E-10	4.70003E-10	1.47614E-10
356.0	7.40908E-10	3.46917E-10	4.70506E-10	1.48079E-10
357.0	7.41366E-10	3.47534E-10	4.71005E-10	1.48542E-10
358.0	7.41821E-10	3.48148E-10	4.71502E-10	1.49003E-10
359.0	7.42274E-10	3.48759E-10	4.71996E-10	1.49463E-10
360.0	7.42725E-10	3.49366E-10	4.72486E-10	1.49922E-10
361.0	7.43174E-10	3.49971E-10	4.72974E-10	1.50379E-10
362.0	7.43620E-10	3.50572E-10	4.73460E-10	1.50834E-10
363.0	7.44065E-10	3.51171E-10	4.73942E-10	1.51287E-10
364.0	7.44507E-10	3.51766E-10	4.74422E-10	1.51740E-10
365.0	7.44947E-10	3.52359E-10	4.74898E-10	1.52190E-10
366.0	7.45385E-10	3.52948E-10	4.75373E-10	1.52639E-10
367.0	7.45821E-10	3.53534E-10	4.75844E-10	1.53087E-10
368.0	7.46255E-10	3.54118E-10	4.76313E-10	1.53533E-10
369.0	7.46687E-10	3.54698E-10	4.76779E-10	1.53977E-10
370.0	7.47117E-10	3.55276E-10	4.77242E-10	1.54420E-10
371.0	7.47545E-10	3.55851E-10	4.77703E-10	1.54861E-10
372.0	7.47971E-10	3.56423E-10	4.78161E-10	1.55301E-10
373.0	7.48394E-10	3.56991E-10	4.78616E-10	1.55739E-10
374.0	7.48816E-10	3.57558E-10	4.79069E-10	1.56176E-10

375.0	7.49236E-10	3.58121E-10	4.79520E-10	1.56611E-10
376.0	7.49654E-10	3.58681E-10	4.79968E-10	1.57044E-10
377.0	7.50070E-10	3.59239E-10	4.80413E-10	1.57476E-10
378.0	7.50484E-10	3.59794E-10	4.80856E-10	1.57907E-10
379.0	7.50896E-10	3.60346E-10	4.81296E-10	1.58336E-10
380.0	7.51306E-10	3.60895E-10	4.81734E-10	1.58764E-10
381.0	7.51715E-10	3.61441E-10	4.82169E-10	1.59190E-10
382.0	7.52121E-10	3.61985E-10	4.82602E-10	1.59614E-10
383.0	7.52526E-10	3.62526E-10	4.83033E-10	1.60037E-10
384.0	7.52928E-10	3.63064E-10	4.83461E-10	1.60459E-10
385.0	7.53329E-10	3.63600E-10	4.83887E-10	1.60879E-10
386.0	7.53728E-10	3.64133E-10	4.84311E-10	1.61297E-10
387.0	7.54126E-10	3.64663E-10	4.84732E-10	1.61715E-10
388.0	7.54521E-10	3.65191E-10	4.85150E-10	1.62130E-10
389.0	7.54915E-10	3.65716E-10	4.85567E-10	1.62544E-10
390.0	7.55307E-10	3.66238E-10	4.85981E-10	1.62957E-10
391.0	7.55697E-10	3.66758E-10	4.86393E-10	1.63368E-10
392.0	7.56085E-10	3.67275E-10	4.86803E-10	1.63778E-10
393.0	7.56472E-10	3.67790E-10	4.87210E-10	1.64186E-10
394.0	7.56857E-10	3.68302E-10	4.87615E-10	1.64593E-10
395.0	7.57240E-10	3.68811E-10	4.88018E-10	1.64999E-10
396.0	7.57622E-10	3.69318E-10	4.88419E-10	1.65403E-10
397.0	7.58001E-10	3.69822E-10	4.88818E-10	1.65805E-10
398.0	7.58379E-10	3.70324E-10	4.89214E-10	1.66206E-10
399.0	7.58756E-10	3.70824E-10	4.89608E-10	1.66606E-10
400.0	7.59131E-10	3.71320E-10	4.90001E-10	1.67004E-10
401.0	7.59504E-10	3.71815E-10	4.90391E-10	1.67401E-10
402.0	7.59875E-10	3.72307E-10	4.90779E-10	1.67796E-10
403.0	7.60245E-10	3.72796E-10	4.91164E-10	1.68190E-10
404.0	7.60613E-10	3.73283E-10	4.91548E-10	1.68583E-10
405.0	7.60980E-10	3.73768E-10	4.91930E-10	1.68974E-10
406.0	7.61345E-10	3.74250E-10	4.92310E-10	1.69363E-10
407.0	7.61709E-10	3.74730E-10	4.92687E-10	1.69752E-10
408.0	7.62070E-10	3.75208E-10	4.93063E-10	1.70139E-10
409.0	7.62431E-10	3.75683E-10	4.93436E-10	1.70524E-10
410.0	7.62789E-10	3.76156E-10	4.93808E-10	1.70908E-10
411.0	7.63147E-10	3.76626E-10	4.94178E-10	1.71291E-10
412.0	7.63502E-10	3.77094E-10	4.94545E-10	1.71673E-10
413.0	7.63856E-10	3.77560E-10	4.94911E-10	1.72053E-10
414.0	7.64209E-10	3.78023E-10	4.95275E-10	1.72431E-10
415.0	7.64560E-10	3.78484E-10	4.95637E-10	1.72808E-10
416.0	7.64910E-10	3.78943E-10	4.95997E-10	1.73184E-10
417.0	7.65258E-10	3.79400E-10	4.96355E-10	1.73559E-10
418.0	7.65604E-10	3.79854E-10	4.96711E-10	1.73932E-10
419.0	7.65949E-10	3.80306E-10	4.97065E-10	1.74304E-10
420.0	7.66293E-10	3.80756E-10	4.97417E-10	1.74674E-10
421.0	7.66635E-10	3.81204E-10	4.97768E-10	1.75043E-10
422.0	7.66976E-10	3.81649E-10	4.98117E-10	1.75411E-10
423.0	7.67315E-10	3.82092E-10	4.98463E-10	1.75778E-10
424.0	7.67653E-10	3.82533E-10	4.98809E-10	1.76143E-10

425.0	7.67989E-10	3.82972E-10	4.99152E-10	1.76507E-10
426.0	7.68324E-10	3.83409E-10	4.99493E-10	1.76869E-10
427.0	7.68658E-10	3.83843E-10	4.99833E-10	1.77230E-10
428.0	7.68990E-10	3.84276E-10	5.00171E-10	1.77590E-10
429.0	7.69321E-10	3.84706E-10	5.00507E-10	1.77949E-10
430.0	7.69650E-10	3.85134E-10	5.00842E-10	1.78306E-10
431.0	7.69978E-10	3.85560E-10	5.01174E-10	1.78662E-10
432.0	7.70304E-10	3.85984E-10	5.01505E-10	1.79016E-10
433.0	7.70630E-10	3.86406E-10	5.01834E-10	1.79370E-10
434.0	7.70953E-10	3.86825E-10	5.02162E-10	1.79722E-10
435.0	7.71276E-10	3.87243E-10	5.02488E-10	1.80073E-10
436.0	7.71597E-10	3.87659E-10	5.02812E-10	1.80422E-10
437.0	7.71917E-10	3.88072E-10	5.03135E-10	1.80770E-10
438.0	7.72235E-10	3.88484E-10	5.03455E-10	1.81117E-10
439.0	7.72552E-10	3.88893E-10	5.03775E-10	1.81463E-10
440.0	7.72868E-10	3.89301E-10	5.04092E-10	1.81807E-10
441.0	7.73183E-10	3.89706E-10	5.04408E-10	1.82150E-10
442.0	7.73496E-10	3.90110E-10	5.04723E-10	1.82492E-10
443.0	7.73808E-10	3.90511E-10	5.05035E-10	1.82833E-10
444.0	7.74118E-10	3.90911E-10	5.05346E-10	1.83172E-10
445.0	7.74428E-10	3.91308E-10	5.05656E-10	1.83510E-10
446.0	7.74736E-10	3.91704E-10	5.05964E-10	1.83847E-10
447.0	7.75042E-10	3.92097E-10	5.06270E-10	1.84183E-10
448.0	7.75348E-10	3.92489E-10	5.06575E-10	1.84517E-10
449.0	7.75652E-10	3.92879E-10	5.06878E-10	1.84850E-10
450.0	7.75955E-10	3.93267E-10	5.07180E-10	1.85182E-10
451.0	7.76257E-10	3.93653E-10	5.07480E-10	1.85513E-10
452.0	7.76557E-10	3.94037E-10	5.07779E-10	1.85843E-10
453.0	7.76857E-10	3.94419E-10	5.08076E-10	1.86171E-10
454.0	7.77155E-10	3.94799E-10	5.08372E-10	1.86498E-10
455.0	7.77451E-10	3.95177E-10	5.08666E-10	1.86824E-10
456.0	7.77747E-10	3.95554E-10	5.08959E-10	1.87149E-10
457.0	7.78041E-10	3.95929E-10	5.09250E-10	1.87472E-10
458.0	7.78334E-10	3.96301E-10	5.09540E-10	1.87794E-10
459.0	7.78626E-10	3.96672E-10	5.09828E-10	1.88116E-10
460.0	7.78917E-10	3.97042E-10	5.10115E-10	1.88436E-10
461.0	7.79206E-10	3.97409E-10	5.10400E-10	1.88754E-10
462.0	7.79495E-10	3.97775E-10	5.10684E-10	1.89072E-10
463.0	7.79782E-10	3.98138E-10	5.10967E-10	1.89388E-10
464.0	7.80068E-10	3.98500E-10	5.11248E-10	1.89703E-10
465.0	7.80353E-10	3.98860E-10	5.11527E-10	1.90018E-10
466.0	7.80636E-10	3.99219E-10	5.11806E-10	1.90330E-10
467.0	7.80919E-10	3.99575E-10	5.12083E-10	1.90642E-10
468.0	7.81200E-10	3.99930E-10	5.12358E-10	1.90953E-10
469.0	7.81480E-10	4.00283E-10	5.12632E-10	1.91262E-10
470.0	7.81759E-10	4.00635E-10	5.12905E-10	1.91570E-10
471.0	7.82037E-10	4.00985E-10	5.13177E-10	1.91878E-10
472.0	7.82314E-10	4.01333E-10	5.13447E-10	1.92184E-10
473.0	7.82590E-10	4.01679E-10	5.13715E-10	1.92488E-10
474.0	7.82864E-10	4.02023E-10	5.13983E-10	1.92792E-10

475.0	7.83138E-10	4.02366E-10	5.14249E-10	1.93095E-10
476.0	7.83410E-10	4.02707E-10	5.14513E-10	1.93396E-10
477.0	7.83681E-10	4.03047E-10	5.14777E-10	1.93697E-10
478.0	7.83951E-10	4.03384E-10	5.15039E-10	1.93996E-10
479.0	7.84220E-10	4.03721E-10	5.15300E-10	1.94294E-10
480.0	7.84488E-10	4.04055E-10	5.15559E-10	1.94591E-10
481.0	7.84754E-10	4.04388E-10	5.15817E-10	1.94887E-10
482.0	7.85020E-10	4.04719E-10	5.16074E-10	1.95182E-10
483.0	7.85285E-10	4.05049E-10	5.16330E-10	1.95475E-10
484.0	7.85548E-10	4.05377E-10	5.16584E-10	1.95768E-10
485.0	7.85810E-10	4.05703E-10	5.16837E-10	1.96060E-10
486.0	7.86072E-10	4.06028E-10	5.17089E-10	1.96350E-10
487.0	7.86332E-10	4.06351E-10	5.17340E-10	1.96639E-10
488.0	7.86591E-10	4.06672E-10	5.17589E-10	1.96928E-10
489.0	7.86849E-10	4.06992E-10	5.17837E-10	1.97215E-10
490.0	7.87106E-10	4.07310E-10	5.18084E-10	1.97501E-10
491.0	7.87362E-10	4.07627E-10	5.18330E-10	1.97786E-10
492.0	7.87617E-10	4.07942E-10	5.18574E-10	1.98070E-10
493.0	7.87871E-10	4.08256E-10	5.18817E-10	1.98353E-10
494.0	7.88124E-10	4.08568E-10	5.19059E-10	1.98635E-10
495.0	7.88376E-10	4.08878E-10	5.19300E-10	1.98916E-10
496.0	7.88627E-10	4.09187E-10	5.19540E-10	1.99195E-10
497.0	7.88876E-10	4.09495E-10	5.19778E-10	1.99474E-10
498.0	7.89125E-10	4.09801E-10	5.20016E-10	1.99752E-10
499.0	7.89373E-10	4.10105E-10	5.20252E-10	2.00028E-10
500.0	7.89620E-10	4.10408E-10	5.20487E-10	2.00304E-10

T (K)	21->31	21->41	31->41	31->51
5.0	2.48978E-35	4.56883E-69	8.72350E-44	1.33397E-85
6.0	4.09937E-31	2.70848E-59	3.28777E-38	4.49092E-73
7.0	4.20881E-28	2.77926E-52	3.38381E-34	4.00237E-64
8.0	7.69266E-26	5.22753E-47	3.58764E-31	2.08992E-57
9.0	4.46143E-24	6.71969E-43	8.22194E-29	3.55324E-52
10.0	1.15833E-22	1.30832E-39	6.38995E-27	5.48952E-48
11.0	1.67431E-21	6.42670E-37	2.25140E-25	1.47952E-44
12.0	1.55779E-20	1.12144E-34	4.37279E-24	1.07451E-41
13.0	1.03166E-19	8.81847E-33	5.36607E-23	2.84214E-39
14.0	5.22644E-19	3.70568E-31	4.58877E-22	3.39633E-37
15.0	2.13569E-18	9.43196E-30	2.93905E-21	2.14801E-35
16.0	7.32597E-18	1.59758E-28	1.48852E-20	8.09753E-34
17.0	2.17511E-17	1.93524E-27	6.21430E-20	1.99292E-32
18.0	5.72482E-17	1.77324E-26	2.20895E-19	3.43745E-31
19.0	1.36119E-16	1.28466E-25	6.85871E-19	4.39455E-30
20.0	2.96849E-16	7.62405E-25	1.89869E-18	4.35517E-29
21.0	6.01110E-16	3.81428E-24	4.76454E-18	3.46964E-28
22.0	1.14169E-15	1.64685E-23	1.09857E-17	2.28913E-27
23.0	2.05088E-15	6.25639E-23	2.35358E-17	1.28185E-26
24.0	3.50869E-15	2.12532E-22	4.72906E-17	6.21842E-26
25.0	5.75050E-15	6.54390E-22	8.98167E-17	2.65875E-25
26.0	9.07335E-15	1.84727E-21	1.62309E-16	1.01661E-24

27.0	1.38409E-14	4.82764E-21	2.80653E-16	3.51970E-24
28.0	2.04863E-14	1.17780E-20	4.66579E-16	1.11519E-23
29.0	2.95136E-14	2.70185E-20	7.48866E-16	3.26337E-23
30.0	4.14967E-14	5.86409E-20	1.16455E-15	8.89022E-23
31.0	5.70768E-14	1.21071E-19	1.76009E-15	2.27036E-22
32.0	7.69581E-14	2.38906E-19	2.59248E-15	5.46818E-22
33.0	1.01903E-13	4.52445E-19	3.73018E-15	1.24874E-21
34.0	1.32723E-13	8.25360E-19	5.25397E-15	2.71655E-21
35.0	1.70274E-13	1.45500E-18	7.25762E-15	5.65316E-21
36.0	2.15449E-13	2.48582E-18	9.84828E-15	1.12953E-20
37.0	2.69163E-13	4.12642E-18	1.31468E-14	2.17404E-20
38.0	3.32353E-13	6.67063E-18	1.72879E-14	4.04282E-20
39.0	4.05965E-13	1.05230E-17	2.24198E-14	7.28279E-20
40.0	4.90946E-13	1.62290E-17	2.87041E-14	1.27392E-19
41.0	5.88237E-13	2.45098E-17	3.63152E-14	2.16848E-19
42.0	6.98766E-13	3.63028E-17	4.54400E-14	3.59897E-19
43.0	8.23441E-13	5.28055E-17	5.62765E-14	5.83416E-19
44.0	9.63145E-13	7.55258E-17	6.90336E-14	9.25237E-19
45.0	1.11873E-12	1.06335E-16	8.39296E-14	1.43759E-18
46.0	1.29101E-12	1.47525E-16	1.01191E-13	2.19133E-18
47.0	1.48076E-12	2.01870E-16	1.21053E-13	3.28095E-18
48.0	1.68872E-12	2.72689E-16	1.43756E-13	4.83055E-18
49.0	1.91557E-12	3.63912E-16	1.69545E-13	7.00074E-18
50.0	2.16195E-12	4.80143E-16	1.98670E-13	9.99663E-18
51.0	2.42845E-12	6.26732E-16	2.31383E-13	1.40768E-17
52.0	2.71561E-12	8.09837E-16	2.67939E-13	1.95633E-17
53.0	3.02393E-12	1.03649E-15	3.08592E-13	2.68532E-17
54.0	3.35383E-12	1.31466E-15	3.53595E-13	3.64300E-17
55.0	3.70570E-12	1.65330E-15	4.03201E-13	4.88779E-17
56.0	4.07989E-12	2.06245E-15	4.57659E-13	6.48951E-17
57.0	4.47667E-12	2.55321E-15	5.17215E-13	8.53094E-17
58.0	4.89628E-12	3.13786E-15	5.82110E-13	1.11094E-16
59.0	5.33892E-12	3.82987E-15	6.52581E-13	1.43384E-16
60.0	5.80472E-12	4.64392E-15	7.28857E-13	1.83494E-16
61.0	6.29379E-12	5.59598E-15	8.11163E-13	2.32934E-16
62.0	6.80618E-12	6.70327E-15	8.99715E-13	2.93431E-16
63.0	7.34190E-12	7.98434E-15	9.94720E-13	3.66943E-16
64.0	7.90094E-12	9.45903E-15	1.09638E-12	4.55679E-16
65.0	8.48323E-12	1.11485E-14	1.20489E-12	5.62119E-16
66.0	9.08868E-12	1.30752E-14	1.32042E-12	6.89028E-16
67.0	9.71716E-12	1.52630E-14	1.44316E-12	8.39475E-16
68.0	1.03685E-11	1.77368E-14	1.57326E-12	1.01685E-15
69.0	1.10425E-11	2.05231E-14	1.71088E-12	1.22489E-15
70.0	1.17390E-11	2.36493E-14	1.85617E-12	1.46766E-15
71.0	1.24577E-11	2.71444E-14	2.00925E-12	1.74962E-15
72.0	1.31983E-11	3.10381E-14	2.17026E-12	2.07559E-15
73.0	1.39606E-11	3.53617E-14	2.33931E-12	2.45080E-15
74.0	1.47442E-11	4.01472E-14	2.51650E-12	2.88087E-15
75.0	1.55489E-11	4.54278E-14	2.70192E-12	3.37184E-15
76.0	1.63742E-11	5.12377E-14	2.89567E-12	3.93017E-15

77.0	1.72198E-11	5.76120E-14	3.09782E-12	4.56275E-15
78.0	1.80854E-11	6.45865E-14	3.30842E-12	5.27691E-15
79.0	1.89705E-11	7.21981E-14	3.52755E-12	6.08041E-15
80.0	1.98748E-11	8.04841E-14	3.75525E-12	6.98146E-15
81.0	2.07978E-11	8.94827E-14	3.99155E-12	7.98871E-15
82.0	2.17391E-11	9.92326E-14	4.23649E-12	9.11125E-15
83.0	2.26983E-11	1.09773E-13	4.49008E-12	1.03586E-14
84.0	2.36750E-11	1.21144E-13	4.75235E-12	1.17408E-14
85.0	2.46688E-11	1.33384E-13	5.02329E-12	1.32682E-14
86.0	2.56791E-11	1.46536E-13	5.30290E-12	1.49517E-14
87.0	2.67056E-11	1.60639E-13	5.59118E-12	1.68025E-14
88.0	2.77478E-11	1.75734E-13	5.88810E-12	1.88323E-14
89.0	2.88053E-11	1.91862E-13	6.19364E-12	2.10531E-14
90.0	2.98776E-11	2.09063E-13	6.50778E-12	2.34775E-14
91.0	3.09643E-11	2.27380E-13	6.83047E-12	2.61184E-14
92.0	3.20649E-11	2.46853E-13	7.16168E-12	2.89888E-14
93.0	3.31790E-11	2.67522E-13	7.50135E-12	3.21026E-14
94.0	3.43061E-11	2.89429E-13	7.84943E-12	3.54734E-14
95.0	3.54458E-11	3.12612E-13	8.20585E-12	3.91157E-14
96.0	3.65977E-11	3.37113E-13	8.57057E-12	4.30440E-14
97.0	3.77613E-11	3.62971E-13	8.94350E-12	4.72731E-14
98.0	3.89362E-11	3.90224E-13	9.32458E-12	5.18183E-14
99.0	4.01220E-11	4.18912E-13	9.71372E-12	5.66949E-14
100.0	4.13182E-11	4.49073E-13	1.01108E-11	6.19185E-14
101.0	4.25245E-11	4.80744E-13	1.05159E-11	6.75051E-14
102.0	4.37404E-11	5.13962E-13	1.09287E-11	7.34707E-14
103.0	4.49656E-11	5.48764E-13	1.13493E-11	7.98316E-14
104.0	4.61995E-11	5.85185E-13	1.17774E-11	8.66043E-14
105.0	4.74419E-11	6.23260E-13	1.22131E-11	9.38053E-14
106.0	4.86923E-11	6.63023E-13	1.26563E-11	1.01451E-13
107.0	4.99504E-11	7.04507E-13	1.31067E-11	1.09560E-13
108.0	5.12158E-11	7.47746E-13	1.35644E-11	1.18147E-13
109.0	5.24880E-11	7.92770E-13	1.40292E-11	1.27230E-13
110.0	5.37669E-11	8.39610E-13	1.45009E-11	1.36826E-13
111.0	5.50519E-11	8.88297E-13	1.49796E-11	1.46952E-13
112.0	5.63428E-11	9.38860E-13	1.54651E-11	1.57625E-13
113.0	5.76392E-11	9.91326E-13	1.59572E-11	1.68863E-13
114.0	5.89408E-11	1.04572E-12	1.64559E-11	1.80682E-13
115.0	6.02472E-11	1.10208E-12	1.69610E-11	1.93100E-13
116.0	6.15582E-11	1.16041E-12	1.74724E-11	2.06134E-13
117.0	6.28733E-11	1.22075E-12	1.79900E-11	2.19801E-13
118.0	6.41924E-11	1.28313E-12	1.85137E-11	2.34117E-13
119.0	6.55151E-11	1.34755E-12	1.90434E-11	2.49101E-13
120.0	6.68411E-11	1.41405E-12	1.95790E-11	2.64767E-13
121.0	6.81701E-11	1.48264E-12	2.01202E-11	2.81133E-13
122.0	6.95019E-11	1.55334E-12	2.06671E-11	2.98216E-13
123.0	7.08361E-11	1.62618E-12	2.12195E-11	3.16032E-13
124.0	7.21725E-11	1.70116E-12	2.17772E-11	3.34596E-13
125.0	7.35109E-11	1.77831E-12	2.23402E-11	3.53925E-13
126.0	7.48510E-11	1.85764E-12	2.29083E-11	3.74036E-13

127.0	7.61925E-11	1.93917E-12	2.34815E-11	3.94942E-13
128.0	7.75352E-11	2.02290E-12	2.40595E-11	4.16661E-13
129.0	7.88788E-11	2.10886E-12	2.46424E-11	4.39206E-13
130.0	8.02233E-11	2.19705E-12	2.52299E-11	4.62594E-13
131.0	8.15682E-11	2.28748E-12	2.58219E-11	4.86838E-13
132.0	8.29134E-11	2.38016E-12	2.64184E-11	5.11954E-13
133.0	8.42587E-11	2.47510E-12	2.70192E-11	5.37955E-13
134.0	8.56039E-11	2.57232E-12	2.76243E-11	5.64855E-13
135.0	8.69488E-11	2.67181E-12	2.82334E-11	5.92669E-13
136.0	8.82932E-11	2.77358E-12	2.88465E-11	6.21410E-13
137.0	8.96369E-11	2.87764E-12	2.94634E-11	6.51090E-13
138.0	9.09797E-11	2.98399E-12	3.00842E-11	6.81723E-13
139.0	9.23215E-11	3.09263E-12	3.07086E-11	7.13322E-13
140.0	9.36620E-11	3.20358E-12	3.13365E-11	7.45898E-13
141.0	9.50011E-11	3.31682E-12	3.19679E-11	7.79463E-13
142.0	9.63387E-11	3.43236E-12	3.26026E-11	8.14031E-13
143.0	9.76746E-11	3.55020E-12	3.32405E-11	8.49610E-13
144.0	9.90086E-11	3.67035E-12	3.38815E-11	8.86214E-13
145.0	1.00341E-10	3.79279E-12	3.45256E-11	9.23852E-13
146.0	1.01670E-10	3.91753E-12	3.51726E-11	9.62536E-13
147.0	1.02998E-10	4.04456E-12	3.58224E-11	1.00227E-12
148.0	1.04323E-10	4.17388E-12	3.64749E-11	1.04308E-12
149.0	1.05646E-10	4.30549E-12	3.71301E-11	1.08496E-12
150.0	1.06965E-10	4.43938E-12	3.77878E-11	1.12792E-12
151.0	1.08283E-10	4.57554E-12	3.84479E-11	1.17197E-12
152.0	1.09597E-10	4.71398E-12	3.91104E-11	1.21713E-12
153.0	1.10908E-10	4.85468E-12	3.97751E-11	1.26339E-12
154.0	1.12216E-10	4.99763E-12	4.04419E-11	1.31078E-12
155.0	1.13521E-10	5.14283E-12	4.11109E-11	1.35928E-12
156.0	1.14822E-10	5.29027E-12	4.17818E-11	1.40892E-12
157.0	1.16120E-10	5.43995E-12	4.24546E-11	1.45970E-12
158.0	1.17415E-10	5.59184E-12	4.31292E-11	1.51163E-12
159.0	1.18705E-10	5.74594E-12	4.38055E-11	1.56470E-12
160.0	1.19992E-10	5.90225E-12	4.44835E-11	1.61894E-12
161.0	1.21276E-10	6.06074E-12	4.51630E-11	1.67434E-12
162.0	1.22555E-10	6.22141E-12	4.58441E-11	1.73090E-12
163.0	1.23831E-10	6.38425E-12	4.65265E-11	1.78865E-12
164.0	1.25102E-10	6.54924E-12	4.72102E-11	1.84757E-12
165.0	1.26369E-10	6.71637E-12	4.78952E-11	1.90767E-12
166.0	1.27633E-10	6.88562E-12	4.85813E-11	1.96896E-12
167.0	1.28892E-10	7.05700E-12	4.92686E-11	2.03145E-12
168.0	1.30146E-10	7.23047E-12	4.99568E-11	2.09513E-12
169.0	1.31397E-10	7.40603E-12	5.06460E-11	2.16000E-12
170.0	1.32642E-10	7.58365E-12	5.13362E-11	2.22608E-12
171.0	1.33884E-10	7.76334E-12	5.20271E-11	2.29337E-12
172.0	1.35121E-10	7.94506E-12	5.27187E-11	2.36185E-12
173.0	1.36353E-10	8.12882E-12	5.34111E-11	2.43155E-12
174.0	1.37581E-10	8.31458E-12	5.41040E-11	2.50246E-12
175.0	1.38804E-10	8.50233E-12	5.47976E-11	2.57459E-12
176.0	1.40022E-10	8.69206E-12	5.54916E-11	2.64792E-12

177.0	1.41236E-10	8.88375E-12	5.61860E-11	2.72247E-12
178.0	1.42444E-10	9.07738E-12	5.68808E-11	2.79824E-12
179.0	1.43648E-10	9.27295E-12	5.75759E-11	2.87522E-12
180.0	1.44847E-10	9.47042E-12	5.82713E-11	2.95342E-12
181.0	1.46042E-10	9.66978E-12	5.89669E-11	3.03284E-12
182.0	1.47231E-10	9.87101E-12	5.96626E-11	3.11347E-12
183.0	1.48415E-10	1.00741E-11	6.03584E-11	3.19532E-12
184.0	1.49594E-10	1.02790E-11	6.10542E-11	3.27839E-12
185.0	1.50769E-10	1.04858E-11	6.17500E-11	3.36267E-12
186.0	1.51938E-10	1.06943E-11	6.24457E-11	3.44816E-12
187.0	1.53102E-10	1.09047E-11	6.31413E-11	3.53487E-12
188.0	1.54261E-10	1.11168E-11	6.38368E-11	3.62279E-12
189.0	1.55415E-10	1.13306E-11	6.45320E-11	3.71192E-12
190.0	1.56564E-10	1.15462E-11	6.52270E-11	3.80226E-12
191.0	1.57708E-10	1.17635E-11	6.59217E-11	3.89380E-12
192.0	1.58847E-10	1.19824E-11	6.66160E-11	3.98654E-12
193.0	1.59980E-10	1.22031E-11	6.73099E-11	4.08049E-12
194.0	1.61109E-10	1.24254E-11	6.80034E-11	4.17564E-12
195.0	1.62232E-10	1.26493E-11	6.86963E-11	4.27198E-12
196.0	1.63350E-10	1.28748E-11	6.93888E-11	4.36951E-12
197.0	1.64462E-10	1.31020E-11	7.00807E-11	4.46823E-12
198.0	1.65570E-10	1.33307E-11	7.07720E-11	4.56814E-12
199.0	1.66672E-10	1.35609E-11	7.14627E-11	4.66923E-12
200.0	1.67769E-10	1.37927E-11	7.21527E-11	4.77150E-12
201.0	1.68861E-10	1.40261E-11	7.28420E-11	4.87494E-12
202.0	1.69948E-10	1.42609E-11	7.35305E-11	4.97956E-12
203.0	1.71029E-10	1.44972E-11	7.42183E-11	5.08534E-12
204.0	1.72105E-10	1.47349E-11	7.49052E-11	5.19228E-12
205.0	1.73176E-10	1.49741E-11	7.55913E-11	5.30039E-12
206.0	1.74242E-10	1.52147E-11	7.62765E-11	5.40964E-12
207.0	1.75303E-10	1.54567E-11	7.69608E-11	5.52005E-12
208.0	1.76358E-10	1.57001E-11	7.76441E-11	5.63159E-12
209.0	1.77408E-10	1.59449E-11	7.83265E-11	5.74428E-12
210.0	1.78453E-10	1.61910E-11	7.90079E-11	5.85810E-12
211.0	1.79492E-10	1.64384E-11	7.96882E-11	5.97305E-12
212.0	1.80527E-10	1.66872E-11	8.03675E-11	6.08912E-12
213.0	1.81556E-10	1.69372E-11	8.10457E-11	6.20631E-12
214.0	1.82580E-10	1.71885E-11	8.17228E-11	6.32461E-12
215.0	1.83599E-10	1.74410E-11	8.23988E-11	6.44402E-12
216.0	1.84613E-10	1.76948E-11	8.30736E-11	6.56452E-12
217.0	1.85621E-10	1.79497E-11	8.37472E-11	6.68613E-12
218.0	1.86624E-10	1.82059E-11	8.44196E-11	6.80882E-12
219.0	1.87623E-10	1.84633E-11	8.50908E-11	6.93259E-12
220.0	1.88616E-10	1.87218E-11	8.57607E-11	7.05744E-12
221.0	1.89604E-10	1.89814E-11	8.64294E-11	7.18336E-12
222.0	1.90586E-10	1.92422E-11	8.70967E-11	7.31035E-12
223.0	1.91564E-10	1.95040E-11	8.77628E-11	7.43839E-12
224.0	1.92537E-10	1.97670E-11	8.84275E-11	7.56748E-12
225.0	1.93504E-10	2.00310E-11	8.90908E-11	7.69762E-12
226.0	1.94467E-10	2.02960E-11	8.97528E-11	7.82879E-12

227.0	1.95424E-10	2.05621E-11	9.04134E-11	7.96100E-12
228.0	1.96377E-10	2.08292E-11	9.10726E-11	8.09422E-12
229.0	1.97324E-10	2.10973E-11	9.17303E-11	8.22847E-12
230.0	1.98267E-10	2.13663E-11	9.23867E-11	8.36373E-12
231.0	1.99204E-10	2.16364E-11	9.30415E-11	8.49999E-12
232.0	2.00137E-10	2.19073E-11	9.36949E-11	8.63725E-12
233.0	2.01064E-10	2.21792E-11	9.43468E-11	8.77549E-12
234.0	2.01987E-10	2.24520E-11	9.49972E-11	8.91472E-12
235.0	2.02905E-10	2.27257E-11	9.56461E-11	9.05492E-12
236.0	2.03817E-10	2.30002E-11	9.62935E-11	9.19609E-12
237.0	2.04725E-10	2.32757E-11	9.69393E-11	9.33822E-12
238.0	2.05628E-10	2.35519E-11	9.75836E-11	9.48131E-12
239.0	2.06527E-10	2.38290E-11	9.82263E-11	9.62534E-12
240.0	2.07420E-10	2.41069E-11	9.88674E-11	9.77031E-12
241.0	2.08309E-10	2.43856E-11	9.95069E-11	9.91621E-12
242.0	2.09193E-10	2.46650E-11	1.00145E-10	1.00630E-11
243.0	2.10072E-10	2.49452E-11	1.00781E-10	1.02108E-11
244.0	2.10946E-10	2.52262E-11	1.01416E-10	1.03594E-11
245.0	2.11816E-10	2.55079E-11	1.02049E-10	1.05090E-11
246.0	2.12680E-10	2.57903E-11	1.02680E-10	1.06594E-11
247.0	2.13541E-10	2.60733E-11	1.03310E-10	1.08107E-11
248.0	2.14396E-10	2.63571E-11	1.03938E-10	1.09629E-11
249.0	2.15247E-10	2.66416E-11	1.04565E-10	1.11160E-11
250.0	2.16093E-10	2.69267E-11	1.05190E-10	1.12700E-11
251.0	2.16935E-10	2.72124E-11	1.05813E-10	1.14247E-11
252.0	2.17772E-10	2.74987E-11	1.06434E-10	1.15804E-11
253.0	2.18605E-10	2.77857E-11	1.07054E-10	1.17369E-11
254.0	2.19433E-10	2.80732E-11	1.07672E-10	1.18942E-11
255.0	2.20256E-10	2.83614E-11	1.08288E-10	1.20523E-11
256.0	2.21076E-10	2.86501E-11	1.08903E-10	1.22113E-11
257.0	2.21890E-10	2.89393E-11	1.09516E-10	1.23710E-11
258.0	2.22700E-10	2.92291E-11	1.10127E-10	1.25316E-11
259.0	2.23506E-10	2.95194E-11	1.10737E-10	1.26930E-11
260.0	2.24307E-10	2.98102E-11	1.11344E-10	1.28551E-11
261.0	2.25104E-10	3.01015E-11	1.11950E-10	1.30180E-11
262.0	2.25897E-10	3.03933E-11	1.12555E-10	1.31817E-11
263.0	2.26685E-10	3.06856E-11	1.13157E-10	1.33462E-11
264.0	2.27469E-10	3.09783E-11	1.13758E-10	1.35114E-11
265.0	2.28248E-10	3.12714E-11	1.14357E-10	1.36773E-11
266.0	2.29024E-10	3.15650E-11	1.14954E-10	1.38440E-11
267.0	2.29795E-10	3.18590E-11	1.15550E-10	1.40114E-11
268.0	2.30562E-10	3.21534E-11	1.16143E-10	1.41796E-11
269.0	2.31324E-10	3.24482E-11	1.16735E-10	1.43485E-11
270.0	2.32083E-10	3.27434E-11	1.17326E-10	1.45180E-11
271.0	2.32837E-10	3.30389E-11	1.17914E-10	1.46883E-11
272.0	2.33587E-10	3.33348E-11	1.18501E-10	1.48593E-11
273.0	2.34333E-10	3.36311E-11	1.19086E-10	1.50309E-11
274.0	2.35075E-10	3.39276E-11	1.19669E-10	1.52032E-11
275.0	2.35813E-10	3.42245E-11	1.20250E-10	1.53762E-11
276.0	2.36547E-10	3.45217E-11	1.20830E-10	1.55499E-11

277.0	2.37277E-10	3.48192E-11	1.21408E-10	1.57242E-11
278.0	2.38003E-10	3.51170E-11	1.21984E-10	1.58992E-11
279.0	2.38725E-10	3.54150E-11	1.22558E-10	1.60748E-11
280.0	2.39443E-10	3.57133E-11	1.23130E-10	1.62510E-11
281.0	2.40156E-10	3.60119E-11	1.23701E-10	1.64279E-11
282.0	2.40867E-10	3.63107E-11	1.24270E-10	1.66053E-11
283.0	2.41573E-10	3.66097E-11	1.24837E-10	1.67834E-11
284.0	2.42275E-10	3.69090E-11	1.25403E-10	1.69621E-11
285.0	2.42973E-10	3.72085E-11	1.25966E-10	1.71414E-11
286.0	2.43668E-10	3.75081E-11	1.26528E-10	1.73212E-11
287.0	2.44359E-10	3.78080E-11	1.27088E-10	1.75017E-11
288.0	2.45046E-10	3.81080E-11	1.27647E-10	1.76827E-11
289.0	2.45729E-10	3.84082E-11	1.28203E-10	1.78643E-11
290.0	2.46409E-10	3.87085E-11	1.28758E-10	1.80464E-11
291.0	2.47085E-10	3.90090E-11	1.29311E-10	1.82291E-11
292.0	2.47757E-10	3.93097E-11	1.29862E-10	1.84123E-11
293.0	2.48425E-10	3.96105E-11	1.30411E-10	1.85961E-11
294.0	2.49090E-10	3.99113E-11	1.30959E-10	1.87804E-11
295.0	2.49751E-10	4.02123E-11	1.31505E-10	1.89652E-11
296.0	2.50409E-10	4.05134E-11	1.32049E-10	1.91505E-11
297.0	2.51063E-10	4.08146E-11	1.32592E-10	1.93364E-11
298.0	2.51713E-10	4.11159E-11	1.33132E-10	1.95227E-11
299.0	2.52360E-10	4.14173E-11	1.33671E-10	1.97095E-11
300.0	2.53004E-10	4.17187E-11	1.34208E-10	1.98969E-11
301.0	2.53644E-10	4.20201E-11	1.34744E-10	2.00846E-11
302.0	2.54280E-10	4.23217E-11	1.35277E-10	2.02729E-11
303.0	2.54913E-10	4.26232E-11	1.35809E-10	2.04616E-11
304.0	2.55543E-10	4.29248E-11	1.36339E-10	2.06508E-11
305.0	2.56169E-10	4.32264E-11	1.36868E-10	2.08405E-11
306.0	2.56791E-10	4.35280E-11	1.37394E-10	2.10305E-11
307.0	2.57411E-10	4.38297E-11	1.37919E-10	2.12210E-11
308.0	2.58027E-10	4.41313E-11	1.38443E-10	2.14120E-11
309.0	2.58640E-10	4.44329E-11	1.38964E-10	2.16034E-11
310.0	2.59249E-10	4.47345E-11	1.39484E-10	2.17951E-11
311.0	2.59855E-10	4.50361E-11	1.40002E-10	2.19873E-11
312.0	2.60458E-10	4.53377E-11	1.40518E-10	2.21799E-11
313.0	2.61057E-10	4.56392E-11	1.41033E-10	2.23729E-11
314.0	2.61654E-10	4.59406E-11	1.41546E-10	2.25663E-11
315.0	2.62247E-10	4.62421E-11	1.42057E-10	2.27601E-11
316.0	2.62837E-10	4.65434E-11	1.42566E-10	2.29542E-11
317.0	2.63423E-10	4.68447E-11	1.43074E-10	2.31487E-11
318.0	2.64007E-10	4.71459E-11	1.43580E-10	2.33436E-11
319.0	2.64588E-10	4.74470E-11	1.44084E-10	2.35388E-11
320.0	2.65165E-10	4.77480E-11	1.44587E-10	2.37344E-11
321.0	2.65739E-10	4.80490E-11	1.45088E-10	2.39303E-11
322.0	2.66310E-10	4.83498E-11	1.45587E-10	2.41266E-11
323.0	2.66879E-10	4.86505E-11	1.46085E-10	2.43232E-11
324.0	2.67444E-10	4.89511E-11	1.46581E-10	2.45201E-11
325.0	2.68006E-10	4.92516E-11	1.47075E-10	2.47174E-11
326.0	2.68565E-10	4.95520E-11	1.47568E-10	2.49149E-11

327.0	2.69121E-10	4.98522E-11	1.48059E-10	2.51128E-11
328.0	2.69674E-10	5.01523E-11	1.48548E-10	2.53109E-11
329.0	2.70224E-10	5.04522E-11	1.49036E-10	2.55094E-11
330.0	2.70772E-10	5.07520E-11	1.49522E-10	2.57081E-11
331.0	2.71316E-10	5.10516E-11	1.50006E-10	2.59072E-11
332.0	2.71858E-10	5.13511E-11	1.50489E-10	2.61065E-11
333.0	2.72396E-10	5.16504E-11	1.50970E-10	2.63061E-11
334.0	2.72932E-10	5.19495E-11	1.51450E-10	2.65059E-11
335.0	2.73465E-10	5.22484E-11	1.51927E-10	2.67060E-11
336.0	2.73995E-10	5.25472E-11	1.52404E-10	2.69063E-11
337.0	2.74523E-10	5.28457E-11	1.52878E-10	2.71069E-11
338.0	2.75047E-10	5.31441E-11	1.53351E-10	2.73078E-11
339.0	2.75569E-10	5.34422E-11	1.53822E-10	2.75089E-11
340.0	2.76088E-10	5.37402E-11	1.54292E-10	2.77102E-11
341.0	2.76604E-10	5.40379E-11	1.54760E-10	2.79117E-11
342.0	2.77118E-10	5.43354E-11	1.55227E-10	2.81135E-11
343.0	2.77629E-10	5.46327E-11	1.55692E-10	2.83154E-11
344.0	2.78137E-10	5.49298E-11	1.56155E-10	2.85176E-11
345.0	2.78643E-10	5.52266E-11	1.56617E-10	2.87200E-11
346.0	2.79146E-10	5.55232E-11	1.57077E-10	2.89225E-11
347.0	2.79646E-10	5.58195E-11	1.57536E-10	2.91253E-11
348.0	2.80144E-10	5.61156E-11	1.57993E-10	2.93282E-11
349.0	2.80639E-10	5.64115E-11	1.58448E-10	2.95314E-11
350.0	2.81132E-10	5.67070E-11	1.58902E-10	2.97347E-11
351.0	2.81622E-10	5.70024E-11	1.59354E-10	2.99381E-11
352.0	2.82109E-10	5.72974E-11	1.59805E-10	3.01418E-11
353.0	2.82594E-10	5.75922E-11	1.60254E-10	3.03456E-11
354.0	2.83077E-10	5.78867E-11	1.60702E-10	3.05495E-11
355.0	2.83557E-10	5.81810E-11	1.61148E-10	3.07536E-11
356.0	2.84034E-10	5.84749E-11	1.61593E-10	3.09579E-11
357.0	2.84509E-10	5.87686E-11	1.62036E-10	3.11623E-11
358.0	2.84982E-10	5.90620E-11	1.62477E-10	3.13668E-11
359.0	2.85452E-10	5.93551E-11	1.62917E-10	3.15714E-11
360.0	2.85920E-10	5.96478E-11	1.63356E-10	3.17762E-11
361.0	2.86385E-10	5.99403E-11	1.63793E-10	3.19811E-11
362.0	2.86848E-10	6.02325E-11	1.64228E-10	3.21861E-11
363.0	2.87308E-10	6.05244E-11	1.64662E-10	3.23912E-11
364.0	2.87767E-10	6.08159E-11	1.65095E-10	3.25964E-11
365.0	2.88222E-10	6.11071E-11	1.65526E-10	3.28017E-11
366.0	2.88676E-10	6.13980E-11	1.65955E-10	3.30071E-11
367.0	2.89127E-10	6.16886E-11	1.66383E-10	3.32126E-11
368.0	2.89576E-10	6.19789E-11	1.66810E-10	3.34182E-11
369.0	2.90023E-10	6.22688E-11	1.67235E-10	3.36239E-11
370.0	2.90467E-10	6.25584E-11	1.67659E-10	3.38296E-11
371.0	2.90909E-10	6.28476E-11	1.68081E-10	3.40354E-11
372.0	2.91349E-10	6.31366E-11	1.68501E-10	3.42413E-11
373.0	2.91787E-10	6.34251E-11	1.68921E-10	3.44472E-11
374.0	2.92222E-10	6.37133E-11	1.69338E-10	3.46532E-11
375.0	2.92655E-10	6.40012E-11	1.69755E-10	3.48593E-11
376.0	2.93086E-10	6.42887E-11	1.70170E-10	3.50654E-11

377.0	2.93515E-10	6.45759E-11	1.70583E-10	3.52716E-11
378.0	2.93942E-10	6.48626E-11	1.70995E-10	3.54777E-11
379.0	2.94366E-10	6.51491E-11	1.71406E-10	3.56840E-11
380.0	2.94789E-10	6.54351E-11	1.71815E-10	3.58902E-11
381.0	2.95209E-10	6.57208E-11	1.72222E-10	3.60965E-11
382.0	2.95627E-10	6.60061E-11	1.72629E-10	3.63028E-11
383.0	2.96044E-10	6.62911E-11	1.73034E-10	3.65091E-11
384.0	2.96458E-10	6.65757E-11	1.73437E-10	3.67155E-11
385.0	2.96869E-10	6.68598E-11	1.73839E-10	3.69218E-11
386.0	2.97279E-10	6.71436E-11	1.74240E-10	3.71282E-11
387.0	2.97687E-10	6.74271E-11	1.74639E-10	3.73345E-11
388.0	2.98093E-10	6.77101E-11	1.75037E-10	3.75409E-11
389.0	2.98497E-10	6.79927E-11	1.75434E-10	3.77473E-11
390.0	2.98899E-10	6.82750E-11	1.75829E-10	3.79536E-11
391.0	2.99299E-10	6.85568E-11	1.76223E-10	3.81599E-11
392.0	2.99696E-10	6.88383E-11	1.76615E-10	3.83662E-11
393.0	3.00092E-10	6.91193E-11	1.77006E-10	3.85725E-11
394.0	3.00486E-10	6.94000E-11	1.77396E-10	3.87788E-11
395.0	3.00878E-10	6.96802E-11	1.77784E-10	3.89850E-11
396.0	3.01268E-10	6.99600E-11	1.78171E-10	3.91912E-11
397.0	3.01656E-10	7.02394E-11	1.78557E-10	3.93974E-11
398.0	3.02042E-10	7.05185E-11	1.78941E-10	3.96035E-11
399.0	3.02427E-10	7.07971E-11	1.79324E-10	3.98096E-11
400.0	3.02809E-10	7.10752E-11	1.79705E-10	4.00157E-11
401.0	3.03190E-10	7.13530E-11	1.80086E-10	4.02217E-11
402.0	3.03568E-10	7.16303E-11	1.80465E-10	4.04276E-11
403.0	3.03945E-10	7.19073E-11	1.80842E-10	4.06335E-11
404.0	3.04320E-10	7.21837E-11	1.81218E-10	4.08393E-11
405.0	3.04693E-10	7.24598E-11	1.81593E-10	4.10450E-11
406.0	3.05065E-10	7.27355E-11	1.81967E-10	4.12507E-11
407.0	3.05434E-10	7.30107E-11	1.82339E-10	4.14563E-11
408.0	3.05802E-10	7.32854E-11	1.82711E-10	4.16618E-11
409.0	3.06168E-10	7.35598E-11	1.83080E-10	4.18673E-11
410.0	3.06532E-10	7.38337E-11	1.83449E-10	4.20727E-11
411.0	3.06894E-10	7.41072E-11	1.83816E-10	4.22780E-11
412.0	3.07255E-10	7.43802E-11	1.84182E-10	4.24832E-11
413.0	3.07614E-10	7.46528E-11	1.84546E-10	4.26883E-11
414.0	3.07971E-10	7.49249E-11	1.84910E-10	4.28933E-11
415.0	3.08326E-10	7.51966E-11	1.85272E-10	4.30982E-11
416.0	3.08680E-10	7.54679E-11	1.85633E-10	4.33030E-11
417.0	3.09032E-10	7.57387E-11	1.85992E-10	4.35077E-11
418.0	3.09382E-10	7.60091E-11	1.86350E-10	4.37123E-11
419.0	3.09731E-10	7.62790E-11	1.86707E-10	4.39168E-11
420.0	3.10078E-10	7.65484E-11	1.87063E-10	4.41212E-11
421.0	3.10423E-10	7.68174E-11	1.87418E-10	4.43254E-11
422.0	3.10767E-10	7.70860E-11	1.87771E-10	4.45295E-11
423.0	3.11109E-10	7.73541E-11	1.88123E-10	4.47336E-11
424.0	3.11449E-10	7.76217E-11	1.88474E-10	4.49374E-11
425.0	3.11788E-10	7.78889E-11	1.88823E-10	4.51412E-11
426.0	3.12125E-10	7.81556E-11	1.89172E-10	4.53448E-11

427.0	3.12461E-10	7.84218E-11	1.89519E-10	4.55483E-11
428.0	3.12794E-10	7.86876E-11	1.89865E-10	4.57517E-11
429.0	3.13127E-10	7.89530E-11	1.90209E-10	4.59549E-11
430.0	3.13457E-10	7.92178E-11	1.90553E-10	4.61579E-11
431.0	3.13787E-10	7.94822E-11	1.90895E-10	4.63609E-11
432.0	3.14114E-10	7.97461E-11	1.91236E-10	4.65636E-11
433.0	3.14440E-10	8.00096E-11	1.91576E-10	4.67662E-11
434.0	3.14765E-10	8.02726E-11	1.91914E-10	4.69687E-11
435.0	3.15088E-10	8.05351E-11	1.92252E-10	4.71710E-11
436.0	3.15409E-10	8.07971E-11	1.92588E-10	4.73732E-11
437.0	3.15729E-10	8.10587E-11	1.92923E-10	4.75752E-11
438.0	3.16047E-10	8.13198E-11	1.93257E-10	4.77770E-11
439.0	3.16364E-10	8.15804E-11	1.93590E-10	4.79786E-11
440.0	3.16680E-10	8.18406E-11	1.93921E-10	4.81801E-11
441.0	3.16994E-10	8.21002E-11	1.94252E-10	4.83814E-11
442.0	3.17306E-10	8.23594E-11	1.94581E-10	4.85826E-11
443.0	3.17617E-10	8.26181E-11	1.94909E-10	4.87835E-11
444.0	3.17926E-10	8.28763E-11	1.95236E-10	4.89843E-11
445.0	3.18234E-10	8.31341E-11	1.95561E-10	4.91849E-11
446.0	3.18541E-10	8.33914E-11	1.95886E-10	4.93853E-11
447.0	3.18846E-10	8.36481E-11	1.96209E-10	4.95855E-11
448.0	3.19150E-10	8.39044E-11	1.96532E-10	4.97856E-11
449.0	3.19452E-10	8.41602E-11	1.96853E-10	4.99854E-11
450.0	3.19753E-10	8.44156E-11	1.97173E-10	5.01851E-11
451.0	3.20053E-10	8.46704E-11	1.97492E-10	5.03845E-11
452.0	3.20351E-10	8.49247E-11	1.97809E-10	5.05838E-11
453.0	3.20647E-10	8.51786E-11	1.98126E-10	5.07828E-11
454.0	3.20943E-10	8.54320E-11	1.98441E-10	5.09817E-11
455.0	3.21237E-10	8.56849E-11	1.98756E-10	5.11803E-11
456.0	3.21529E-10	8.59372E-11	1.99069E-10	5.13787E-11
457.0	3.21820E-10	8.61891E-11	1.99381E-10	5.15770E-11
458.0	3.22110E-10	8.64406E-11	1.99692E-10	5.17750E-11
459.0	3.22399E-10	8.66915E-11	2.00002E-10	5.19728E-11
460.0	3.22686E-10	8.69419E-11	2.00311E-10	5.21704E-11
461.0	3.22972E-10	8.71918E-11	2.00618E-10	5.23677E-11
462.0	3.23256E-10	8.74413E-11	2.00925E-10	5.25649E-11
463.0	3.23539E-10	8.76902E-11	2.01230E-10	5.27618E-11
464.0	3.23821E-10	8.79387E-11	2.01535E-10	5.29585E-11
465.0	3.24102E-10	8.81866E-11	2.01838E-10	5.31549E-11
466.0	3.24381E-10	8.84341E-11	2.02140E-10	5.33512E-11
467.0	3.24659E-10	8.86810E-11	2.02441E-10	5.35472E-11
468.0	3.24936E-10	8.89275E-11	2.02742E-10	5.37430E-11
469.0	3.25211E-10	8.91734E-11	2.03041E-10	5.39385E-11
470.0	3.25485E-10	8.94189E-11	2.03339E-10	5.41338E-11
471.0	3.25758E-10	8.96639E-11	2.03635E-10	5.43289E-11
472.0	3.26030E-10	8.99083E-11	2.03931E-10	5.45237E-11
473.0	3.26300E-10	9.01523E-11	2.04226E-10	5.47183E-11
474.0	3.26569E-10	9.03958E-11	2.04520E-10	5.49127E-11
475.0	3.26837E-10	9.06387E-11	2.04812E-10	5.51068E-11
476.0	3.27104E-10	9.08812E-11	2.05104E-10	5.53006E-11

477.0	3.27369E-10	9.11232E-11	2.05394E-10	5.54942E-11
478.0	3.27633E-10	9.13646E-11	2.05684E-10	5.56876E-11
479.0	3.27896E-10	9.16056E-11	2.05972E-10	5.58807E-11
480.0	3.28158E-10	9.18461E-11	2.06260E-10	5.60735E-11
481.0	3.28419E-10	9.20860E-11	2.06546E-10	5.62661E-11
482.0	3.28678E-10	9.23255E-11	2.06831E-10	5.64584E-11
483.0	3.28936E-10	9.25644E-11	2.07116E-10	5.66505E-11
484.0	3.29193E-10	9.28029E-11	2.07399E-10	5.68423E-11
485.0	3.29449E-10	9.30408E-11	2.07681E-10	5.70339E-11
486.0	3.29704E-10	9.32782E-11	2.07963E-10	5.72252E-11
487.0	3.29957E-10	9.35152E-11	2.08243E-10	5.74162E-11
488.0	3.30210E-10	9.37516E-11	2.08522E-10	5.76070E-11
489.0	3.30461E-10	9.39875E-11	2.08800E-10	5.77974E-11
490.0	3.30711E-10	9.42230E-11	2.09077E-10	5.79877E-11
491.0	3.30960E-10	9.44579E-11	2.09354E-10	5.81776E-11
492.0	3.31207E-10	9.46923E-11	2.09629E-10	5.83673E-11
493.0	3.31454E-10	9.49262E-11	2.09903E-10	5.85567E-11
494.0	3.31700E-10	9.51596E-11	2.10176E-10	5.87458E-11
495.0	3.31944E-10	9.53925E-11	2.10448E-10	5.89347E-11
496.0	3.32187E-10	9.56249E-11	2.10720E-10	5.91232E-11
497.0	3.32429E-10	9.58567E-11	2.10990E-10	5.93115E-11
498.0	3.32670E-10	9.60881E-11	2.11259E-10	5.94995E-11
499.0	3.32910E-10	9.63190E-11	2.11527E-10	5.96873E-11
500.0	3.33149E-10	9.65493E-11	2.11795E-10	5.98747E-11

Table S9: Computed de-excitation rate in cm^3 $molecule^{-1}$ s^{-1} units coefficients for a series of inelastic processes generated using the 4D RR-PES for the $HeH^+(j_1)\cdot\cdot\cdot$ ortho-$H_2(j_2 = 1)$ system with $\Delta j_1 = -1$ and $\Delta j_1 = -2$.

T (K)	11->01	21->01	21->11	31->11
5.0	4.27949E-10	5.78983E-11	1.95739E-10	1.31031E-10
6.0	3.68838E-10	6.50112E-11	2.19841E-10	1.39359E-10
7.0	3.35379E-10	7.04989E-11	2.38474E-10	1.45500E-10
8.0	3.17789E-10	7.54446E-11	2.55278E-10	1.51444E-10
9.0	3.09711E-10	8.02590E-11	2.71612E-10	1.57731E-10
10.0	3.07171E-10	8.50288E-11	2.87747E-10	1.64326E-10
11.0	3.07747E-10	8.97203E-11	3.03546E-10	1.71030E-10
12.0	3.09992E-10	9.42732E-11	3.18794E-10	1.77646E-10
13.0	3.13054E-10	9.86362E-11	3.33311E-10	1.84030E-10
14.0	3.16442E-10	1.02776E-10	3.46985E-10	1.90091E-10
15.0	3.19879E-10	1.06677E-10	3.59763E-10	1.95782E-10
16.0	3.23214E-10	1.10336E-10	3.71641E-10	2.01087E-10
17.0	3.26372E-10	1.13758E-10	3.82645E-10	2.06011E-10
18.0	3.29320E-10	1.16953E-10	3.92819E-10	2.10570E-10
19.0	3.32048E-10	1.19935E-10	4.02218E-10	2.14787E-10
20.0	3.34562E-10	1.22719E-10	4.10902E-10	2.18689E-10
21.0	3.36871E-10	1.25320E-10	4.18929E-10	2.22300E-10
22.0	3.38992E-10	1.27751E-10	4.26356E-10	2.25648E-10
23.0	3.40937E-10	1.30027E-10	4.33239E-10	2.28757E-10
24.0	3.42723E-10	1.32160E-10	4.39625E-10	2.31647E-10
25.0	3.44364E-10	1.34162E-10	4.45560E-10	2.34341E-10

26.0	3.45871E-10	1.36043E-10	4.51087E-10	2.36856E-10
27.0	3.47257E-10	1.37813E-10	4.56241E-10	2.39209E-10
28.0	3.48533E-10	1.39481E-10	4.61057E-10	2.41415E-10
29.0	3.49707E-10	1.41055E-10	4.65564E-10	2.43487E-10
30.0	3.50788E-10	1.42541E-10	4.69790E-10	2.45436E-10
31.0	3.51784E-10	1.43946E-10	4.73758E-10	2.47274E-10
32.0	3.52701E-10	1.45277E-10	4.77490E-10	2.49010E-10
33.0	3.53546E-10	1.46538E-10	4.81005E-10	2.50651E-10
34.0	3.54323E-10	1.47735E-10	4.84322E-10	2.52206E-10
35.0	3.55038E-10	1.48871E-10	4.87455E-10	2.53682E-10
36.0	3.55695E-10	1.49951E-10	4.90418E-10	2.55084E-10
37.0	3.56299E-10	1.50979E-10	4.93225E-10	2.56418E-10
38.0	3.56852E-10	1.51958E-10	4.95886E-10	2.57688E-10
39.0	3.57359E-10	1.52891E-10	4.98412E-10	2.58900E-10
40.0	3.57821E-10	1.53780E-10	5.00812E-10	2.60058E-10
41.0	3.58243E-10	1.54629E-10	5.03095E-10	2.61164E-10
42.0	3.58626E-10	1.55440E-10	5.05268E-10	2.62222E-10
43.0	3.58974E-10	1.56215E-10	5.07339E-10	2.63236E-10
44.0	3.59287E-10	1.56957E-10	5.09313E-10	2.64207E-10
45.0	3.59569E-10	1.57666E-10	5.11197E-10	2.65139E-10
46.0	3.59820E-10	1.58345E-10	5.12996E-10	2.66033E-10
47.0	3.60044E-10	1.58996E-10	5.14714E-10	2.66892E-10
48.0	3.60241E-10	1.59619E-10	5.16356E-10	2.67717E-10
49.0	3.60413E-10	1.60217E-10	5.17926E-10	2.68511E-10
50.0	3.60562E-10	1.60791E-10	5.19428E-10	2.69275E-10
51.0	3.60688E-10	1.61341E-10	5.20866E-10	2.70010E-10
52.0	3.60794E-10	1.61870E-10	5.22243E-10	2.70717E-10
53.0	3.60880E-10	1.62378E-10	5.23561E-10	2.71399E-10
54.0	3.60947E-10	1.62865E-10	5.24823E-10	2.72055E-10
55.0	3.60996E-10	1.63334E-10	5.26032E-10	2.72688E-10
56.0	3.61029E-10	1.63785E-10	5.27191E-10	2.73298E-10
57.0	3.61046E-10	1.64218E-10	5.28301E-10	2.73886E-10
58.0	3.61049E-10	1.64635E-10	5.29365E-10	2.74453E-10
59.0	3.61037E-10	1.65036E-10	5.30385E-10	2.74999E-10
60.0	3.61012E-10	1.65422E-10	5.31362E-10	2.75526E-10
61.0	3.60975E-10	1.65793E-10	5.32299E-10	2.76035E-10
62.0	3.60926E-10	1.66151E-10	5.33196E-10	2.76524E-10
63.0	3.60866E-10	1.66495E-10	5.34056E-10	2.76997E-10
64.0	3.60795E-10	1.66826E-10	5.34879E-10	2.77452E-10
65.0	3.60714E-10	1.67145E-10	5.35668E-10	2.77891E-10
66.0	3.60624E-10	1.67452E-10	5.36423E-10	2.78314E-10
67.0	3.60525E-10	1.67748E-10	5.37146E-10	2.78721E-10
68.0	3.60418E-10	1.68032E-10	5.37837E-10	2.79113E-10
69.0	3.60303E-10	1.68307E-10	5.38499E-10	2.79491E-10
70.0	3.60180E-10	1.68571E-10	5.39131E-10	2.79855E-10
71.0	3.60051E-10	1.68825E-10	5.39736E-10	2.80204E-10
72.0	3.59914E-10	1.69070E-10	5.40313E-10	2.80541E-10
73.0	3.59772E-10	1.69305E-10	5.40864E-10	2.80864E-10
74.0	3.59624E-10	1.69532E-10	5.41390E-10	2.81175E-10
75.0	3.59470E-10	1.69750E-10	5.41891E-10	2.81473E-10

76.0	3.59312E-10	1.69960E-10	5.42368E-10	2.81760E-10
77.0	3.59148E-10	1.70162E-10	5.42823E-10	2.82034E-10
78.0	3.58980E-10	1.70356E-10	5.43255E-10	2.82297E-10
79.0	3.58807E-10	1.70543E-10	5.43666E-10	2.82549E-10
80.0	3.58631E-10	1.70723E-10	5.44055E-10	2.82790E-10
81.0	3.58451E-10	1.70895E-10	5.44425E-10	2.83021E-10
82.0	3.58267E-10	1.71061E-10	5.44774E-10	2.83241E-10
83.0	3.58080E-10	1.71220E-10	5.45105E-10	2.83451E-10
84.0	3.57891E-10	1.71373E-10	5.45417E-10	2.83651E-10
85.0	3.57698E-10	1.71519E-10	5.45711E-10	2.83841E-10
86.0	3.57503E-10	1.71660E-10	5.45987E-10	2.84023E-10
87.0	3.57305E-10	1.71794E-10	5.46247E-10	2.84194E-10
88.0	3.57105E-10	1.71923E-10	5.46490E-10	2.84357E-10
89.0	3.56903E-10	1.72047E-10	5.46717E-10	2.84511E-10
90.0	3.56699E-10	1.72165E-10	5.46929E-10	2.84657E-10
91.0	3.56494E-10	1.72277E-10	5.47125E-10	2.84794E-10
92.0	3.56286E-10	1.72385E-10	5.47307E-10	2.84923E-10
93.0	3.56078E-10	1.72488E-10	5.47474E-10	2.85044E-10
94.0	3.55868E-10	1.72586E-10	5.47628E-10	2.85157E-10
95.0	3.55657E-10	1.72679E-10	5.47768E-10	2.85262E-10
96.0	3.55444E-10	1.72768E-10	5.47895E-10	2.85360E-10
97.0	3.55231E-10	1.72852E-10	5.48009E-10	2.85451E-10
98.0	3.55017E-10	1.72932E-10	5.48110E-10	2.85534E-10
99.0	3.54803E-10	1.73008E-10	5.48200E-10	2.85611E-10
100.0	3.54587E-10	1.73079E-10	5.48278E-10	2.85680E-10
101.0	3.54371E-10	1.73147E-10	5.48344E-10	2.85743E-10
102.0	3.54155E-10	1.73211E-10	5.48399E-10	2.85800E-10
103.0	3.53938E-10	1.73270E-10	5.48444E-10	2.85850E-10
104.0	3.53722E-10	1.73327E-10	5.48477E-10	2.85894E-10
105.0	3.53504E-10	1.73379E-10	5.48501E-10	2.85931E-10
106.0	3.53287E-10	1.73428E-10	5.48514E-10	2.85963E-10
107.0	3.53070E-10	1.73474E-10	5.48518E-10	2.85989E-10
108.0	3.52853E-10	1.73516E-10	5.48512E-10	2.86010E-10
109.0	3.52636E-10	1.73555E-10	5.48498E-10	2.86024E-10
110.0	3.52419E-10	1.73591E-10	5.48474E-10	2.86034E-10
111.0	3.52202E-10	1.73624E-10	5.48441E-10	2.86038E-10
112.0	3.51985E-10	1.73653E-10	5.48400E-10	2.86036E-10
113.0	3.51769E-10	1.73680E-10	5.48350E-10	2.86030E-10
114.0	3.51553E-10	1.73704E-10	5.48293E-10	2.86019E-10
115.0	3.51338E-10	1.73725E-10	5.48228E-10	2.86003E-10
116.0	3.51123E-10	1.73743E-10	5.48155E-10	2.85982E-10
117.0	3.50909E-10	1.73758E-10	5.48074E-10	2.85957E-10
118.0	3.50695E-10	1.73771E-10	5.47987E-10	2.85927E-10
119.0	3.50482E-10	1.73781E-10	5.47892E-10	2.85892E-10
120.0	3.50269E-10	1.73789E-10	5.47790E-10	2.85854E-10
121.0	3.50057E-10	1.73794E-10	5.47682E-10	2.85811E-10
122.0	3.49845E-10	1.73797E-10	5.47567E-10	2.85764E-10
123.0	3.49635E-10	1.73798E-10	5.47446E-10	2.85713E-10
124.0	3.49425E-10	1.73796E-10	5.47319E-10	2.85658E-10
125.0	3.49216E-10	1.73792E-10	5.47186E-10	2.85599E-10

126.0	3.49007E-10	1.73786E-10	5.47046E-10	2.85536E-10
127.0	3.48800E-10	1.73777E-10	5.46902E-10	2.85470E-10
128.0	3.48593E-10	1.73767E-10	5.46751E-10	2.85400E-10
129.0	3.48387E-10	1.73754E-10	5.46596E-10	2.85327E-10
130.0	3.48183E-10	1.73740E-10	5.46435E-10	2.85250E-10
131.0	3.47979E-10	1.73723E-10	5.46269E-10	2.85170E-10
132.0	3.47775E-10	1.73705E-10	5.46098E-10	2.85087E-10
133.0	3.47573E-10	1.73685E-10	5.45922E-10	2.85001E-10
134.0	3.47372E-10	1.73663E-10	5.45742E-10	2.84911E-10
135.0	3.47172E-10	1.73639E-10	5.45557E-10	2.84818E-10
136.0	3.46973E-10	1.73614E-10	5.45367E-10	2.84723E-10
137.0	3.46774E-10	1.73586E-10	5.45174E-10	2.84625E-10
138.0	3.46577E-10	1.73557E-10	5.44976E-10	2.84523E-10
139.0	3.46381E-10	1.73527E-10	5.44774E-10	2.84419E-10
140.0	3.46186E-10	1.73495E-10	5.44568E-10	2.84313E-10
141.0	3.45992E-10	1.73461E-10	5.44358E-10	2.84203E-10
142.0	3.45798E-10	1.73426E-10	5.44145E-10	2.84091E-10
143.0	3.45606E-10	1.73390E-10	5.43928E-10	2.83977E-10
144.0	3.45415E-10	1.73352E-10	5.43707E-10	2.83860E-10
145.0	3.45225E-10	1.73312E-10	5.43483E-10	2.83741E-10
146.0	3.45037E-10	1.73271E-10	5.43256E-10	2.83620E-10
147.0	3.44849E-10	1.73229E-10	5.43025E-10	2.83496E-10
148.0	3.44662E-10	1.73186E-10	5.42792E-10	2.83370E-10
149.0	3.44476E-10	1.73141E-10	5.42555E-10	2.83242E-10
150.0	3.44292E-10	1.73095E-10	5.42315E-10	2.83112E-10
151.0	3.44108E-10	1.73048E-10	5.42073E-10	2.82980E-10
152.0	3.43926E-10	1.72999E-10	5.41828E-10	2.82846E-10
153.0	3.43745E-10	1.72950E-10	5.41580E-10	2.82710E-10
154.0	3.43565E-10	1.72899E-10	5.41329E-10	2.82572E-10
155.0	3.43386E-10	1.72847E-10	5.41076E-10	2.82432E-10
156.0	3.43208E-10	1.72794E-10	5.40821E-10	2.82290E-10
157.0	3.43031E-10	1.72740E-10	5.40563E-10	2.82147E-10
158.0	3.42855E-10	1.72685E-10	5.40303E-10	2.82002E-10
159.0	3.42680E-10	1.72629E-10	5.40041E-10	2.81855E-10
160.0	3.42507E-10	1.72572E-10	5.39776E-10	2.81706E-10
161.0	3.42334E-10	1.72514E-10	5.39510E-10	2.81557E-10
162.0	3.42163E-10	1.72455E-10	5.39241E-10	2.81405E-10
163.0	3.41993E-10	1.72395E-10	5.38971E-10	2.81252E-10
164.0	3.41823E-10	1.72334E-10	5.38698E-10	2.81098E-10
165.0	3.41655E-10	1.72273E-10	5.38424E-10	2.80942E-10
166.0	3.41488E-10	1.72210E-10	5.38148E-10	2.80785E-10
167.0	3.41322E-10	1.72147E-10	5.37871E-10	2.80626E-10
168.0	3.41158E-10	1.72083E-10	5.37592E-10	2.80466E-10
169.0	3.40994E-10	1.72018E-10	5.37311E-10	2.80305E-10
170.0	3.40831E-10	1.71952E-10	5.37029E-10	2.80143E-10
171.0	3.40670E-10	1.71886E-10	5.36745E-10	2.79979E-10
172.0	3.40509E-10	1.71819E-10	5.36460E-10	2.79814E-10
173.0	3.40350E-10	1.71751E-10	5.36174E-10	2.79649E-10
174.0	3.40191E-10	1.71682E-10	5.35886E-10	2.79482E-10
175.0	3.40034E-10	1.71613E-10	5.35597E-10	2.79313E-10

176.0	3.39878E-10	1.71543E-10	5.35307E-10	2.79144E-10
177.0	3.39723E-10	1.71472E-10	5.35016E-10	2.78974E-10
178.0	3.39568E-10	1.71401E-10	5.34724E-10	2.78803E-10
179.0	3.39415E-10	1.71329E-10	5.34430E-10	2.78631E-10
180.0	3.39263E-10	1.71257E-10	5.34136E-10	2.78458E-10
181.0	3.39112E-10	1.71184E-10	5.33841E-10	2.78284E-10
182.0	3.38962E-10	1.71110E-10	5.33545E-10	2.78110E-10
183.0	3.38813E-10	1.71036E-10	5.33248E-10	2.77934E-10
184.0	3.38665E-10	1.70962E-10	5.32950E-10	2.77758E-10
185.0	3.38518E-10	1.70886E-10	5.32651E-10	2.77581E-10
186.0	3.38372E-10	1.70811E-10	5.32352E-10	2.77403E-10
187.0	3.38227E-10	1.70735E-10	5.32051E-10	2.77224E-10
188.0	3.38083E-10	1.70658E-10	5.31751E-10	2.77044E-10
189.0	3.37940E-10	1.70581E-10	5.31449E-10	2.76864E-10
190.0	3.37798E-10	1.70503E-10	5.31147E-10	2.76684E-10
191.0	3.37657E-10	1.70425E-10	5.30845E-10	2.76502E-10
192.0	3.37517E-10	1.70347E-10	5.30542E-10	2.76320E-10
193.0	3.37378E-10	1.70268E-10	5.30238E-10	2.76137E-10
194.0	3.37240E-10	1.70189E-10	5.29934E-10	2.75954E-10
195.0	3.37103E-10	1.70109E-10	5.29629E-10	2.75770E-10
196.0	3.36967E-10	1.70029E-10	5.29325E-10	2.75586E-10
197.0	3.36832E-10	1.69949E-10	5.29019E-10	2.75401E-10
198.0	3.36698E-10	1.69868E-10	5.28714E-10	2.75215E-10
199.0	3.36564E-10	1.69787E-10	5.28408E-10	2.75029E-10
200.0	3.36432E-10	1.69706E-10	5.28102E-10	2.74843E-10
201.0	3.36300E-10	1.69624E-10	5.27795E-10	2.74656E-10
202.0	3.36170E-10	1.69542E-10	5.27489E-10	2.74469E-10
203.0	3.36040E-10	1.69459E-10	5.27182E-10	2.74281E-10
204.0	3.35911E-10	1.69377E-10	5.26875E-10	2.74093E-10
205.0	3.35784E-10	1.69294E-10	5.26568E-10	2.73905E-10
206.0	3.35657E-10	1.69211E-10	5.26260E-10	2.73716E-10
207.0	3.35531E-10	1.69127E-10	5.25953E-10	2.73526E-10
208.0	3.35405E-10	1.69043E-10	5.25646E-10	2.73337E-10
209.0	3.35281E-10	1.68959E-10	5.25338E-10	2.73147E-10
210.0	3.35158E-10	1.68875E-10	5.25031E-10	2.72957E-10
211.0	3.35035E-10	1.68791E-10	5.24723E-10	2.72766E-10
212.0	3.34914E-10	1.68706E-10	5.24416E-10	2.72576E-10
213.0	3.34793E-10	1.68621E-10	5.24108E-10	2.72384E-10
214.0	3.34673E-10	1.68536E-10	5.23801E-10	2.72193E-10
215.0	3.34554E-10	1.68451E-10	5.23493E-10	2.72002E-10
216.0	3.34435E-10	1.68365E-10	5.23186E-10	2.71810E-10
217.0	3.34318E-10	1.68279E-10	5.22879E-10	2.71618E-10
218.0	3.34201E-10	1.68194E-10	5.22572E-10	2.71426E-10
219.0	3.34085E-10	1.68107E-10	5.22265E-10	2.71233E-10
220.0	3.33970E-10	1.68021E-10	5.21959E-10	2.71041E-10
221.0	3.33856E-10	1.67935E-10	5.21652E-10	2.70848E-10
222.0	3.33743E-10	1.67848E-10	5.21346E-10	2.70655E-10
223.0	3.33630E-10	1.67762E-10	5.21040E-10	2.70462E-10
224.0	3.33518E-10	1.67675E-10	5.20734E-10	2.70269E-10
225.0	3.33407E-10	1.67588E-10	5.20428E-10	2.70076E-10

226.0	3.33297E-10	1.67501E-10	5.20123E-10	2.69882E-10
227.0	3.33187E-10	1.67414E-10	5.19818E-10	2.69689E-10
228.0	3.33079E-10	1.67327E-10	5.19513E-10	2.69495E-10
229.0	3.32971E-10	1.67239E-10	5.19209E-10	2.69302E-10
230.0	3.32864E-10	1.67152E-10	5.18905E-10	2.69108E-10
231.0	3.32757E-10	1.67064E-10	5.18601E-10	2.68914E-10
232.0	3.32651E-10	1.66976E-10	5.18298E-10	2.68720E-10
233.0	3.32547E-10	1.66889E-10	5.17995E-10	2.68527E-10
234.0	3.32442E-10	1.66801E-10	5.17692E-10	2.68333E-10
235.0	3.32339E-10	1.66713E-10	5.17390E-10	2.68139E-10
236.0	3.32236E-10	1.66625E-10	5.17088E-10	2.67945E-10
237.0	3.32134E-10	1.66537E-10	5.16786E-10	2.67751E-10
238.0	3.32033E-10	1.66449E-10	5.16485E-10	2.67557E-10
239.0	3.31932E-10	1.66361E-10	5.16184E-10	2.67363E-10
240.0	3.31832E-10	1.66273E-10	5.15884E-10	2.67169E-10
241.0	3.31733E-10	1.66184E-10	5.15585E-10	2.66975E-10
242.0	3.31634E-10	1.66096E-10	5.15285E-10	2.66782E-10
243.0	3.31536E-10	1.66008E-10	5.14986E-10	2.66588E-10
244.0	3.31439E-10	1.65920E-10	5.14688E-10	2.66394E-10
245.0	3.31343E-10	1.65831E-10	5.14390E-10	2.66200E-10
246.0	3.31247E-10	1.65743E-10	5.14093E-10	2.66007E-10
247.0	3.31152E-10	1.65654E-10	5.13796E-10	2.65813E-10
248.0	3.31057E-10	1.65566E-10	5.13500E-10	2.65620E-10
249.0	3.30963E-10	1.65478E-10	5.13204E-10	2.65426E-10
250.0	3.30870E-10	1.65389E-10	5.12909E-10	2.65233E-10
251.0	3.30778E-10	1.65301E-10	5.12614E-10	2.65040E-10
252.0	3.30686E-10	1.65212E-10	5.12320E-10	2.64847E-10
253.0	3.30594E-10	1.65124E-10	5.12026E-10	2.64654E-10
254.0	3.30504E-10	1.65035E-10	5.11733E-10	2.64461E-10
255.0	3.30414E-10	1.64947E-10	5.11441E-10	2.64268E-10
256.0	3.30324E-10	1.64859E-10	5.11149E-10	2.64075E-10
257.0	3.30236E-10	1.64770E-10	5.10857E-10	2.63883E-10
258.0	3.30147E-10	1.64682E-10	5.10567E-10	2.63690E-10
259.0	3.30060E-10	1.64594E-10	5.10276E-10	2.63498E-10
260.0	3.29973E-10	1.64505E-10	5.09987E-10	2.63306E-10
261.0	3.29887E-10	1.64417E-10	5.09698E-10	2.63114E-10
262.0	3.29801E-10	1.64329E-10	5.09410E-10	2.62922E-10
263.0	3.29716E-10	1.64241E-10	5.09122E-10	2.62731E-10
264.0	3.29631E-10	1.64152E-10	5.08835E-10	2.62539E-10
265.0	3.29547E-10	1.64064E-10	5.08548E-10	2.62348E-10
266.0	3.29464E-10	1.63976E-10	5.08262E-10	2.62157E-10
267.0	3.29381E-10	1.63888E-10	5.07977E-10	2.61966E-10
268.0	3.29298E-10	1.63800E-10	5.07693E-10	2.61775E-10
269.0	3.29217E-10	1.63712E-10	5.07409E-10	2.61584E-10
270.0	3.29136E-10	1.63624E-10	5.07125E-10	2.61394E-10
271.0	3.29055E-10	1.63536E-10	5.06843E-10	2.61204E-10
272.0	3.28975E-10	1.63449E-10	5.06561E-10	2.61014E-10
273.0	3.28895E-10	1.63361E-10	5.06280E-10	2.60824E-10
274.0	3.28816E-10	1.63273E-10	5.05999E-10	2.60635E-10
275.0	3.28738E-10	1.63186E-10	5.05719E-10	2.60445E-10

276.0	3.28660E-10	1.63098E-10	5.05440E-10	2.60256E-10
277.0	3.28583E-10	1.63011E-10	5.05161E-10	2.60067E-10
278.0	3.28506E-10	1.62923E-10	5.04883E-10	2.59878E-10
279.0	3.28429E-10	1.62836E-10	5.04606E-10	2.59690E-10
280.0	3.28354E-10	1.62749E-10	5.04329E-10	2.59502E-10
281.0	3.28278E-10	1.62662E-10	5.04053E-10	2.59314E-10
282.0	3.28204E-10	1.62575E-10	5.03778E-10	2.59126E-10
283.0	3.28129E-10	1.62488E-10	5.03504E-10	2.58938E-10
284.0	3.28055E-10	1.62401E-10	5.03230E-10	2.58751E-10
285.0	3.27982E-10	1.62314E-10	5.02957E-10	2.58564E-10
286.0	3.27909E-10	1.62227E-10	5.02684E-10	2.58377E-10
287.0	3.27837E-10	1.62141E-10	5.02413E-10	2.58191E-10
288.0	3.27765E-10	1.62054E-10	5.02142E-10	2.58004E-10
289.0	3.27694E-10	1.61968E-10	5.01871E-10	2.57818E-10
290.0	3.27623E-10	1.61881E-10	5.01602E-10	2.57632E-10
291.0	3.27553E-10	1.61795E-10	5.01333E-10	2.57447E-10
292.0	3.27483E-10	1.61709E-10	5.01065E-10	2.57262E-10
293.0	3.27413E-10	1.61623E-10	5.00797E-10	2.57077E-10
294.0	3.27344E-10	1.61537E-10	5.00531E-10	2.56892E-10
295.0	3.27276E-10	1.61451E-10	5.00264E-10	2.56707E-10
296.0	3.27208E-10	1.61365E-10	4.99999E-10	2.56523E-10
297.0	3.27140E-10	1.61279E-10	4.99735E-10	2.56339E-10
298.0	3.27073E-10	1.61194E-10	4.99471E-10	2.56155E-10
299.0	3.27006E-10	1.61108E-10	4.99208E-10	2.55972E-10
300.0	3.26940E-10	1.61023E-10	4.98945E-10	2.55789E-10
301.0	3.26874E-10	1.60937E-10	4.98683E-10	2.55606E-10
302.0	3.26809E-10	1.60852E-10	4.98422E-10	2.55424E-10
303.0	3.26744E-10	1.60767E-10	4.98162E-10	2.55241E-10
304.0	3.26679E-10	1.60682E-10	4.97903E-10	2.55059E-10
305.0	3.26615E-10	1.60597E-10	4.97644E-10	2.54878E-10
306.0	3.26551E-10	1.60513E-10	4.97386E-10	2.54696E-10
307.0	3.26488E-10	1.60428E-10	4.97128E-10	2.54515E-10
308.0	3.26425E-10	1.60344E-10	4.96872E-10	2.54334E-10
309.0	3.26363E-10	1.60259E-10	4.96616E-10	2.54154E-10
310.0	3.26301E-10	1.60175E-10	4.96360E-10	2.53974E-10
311.0	3.26239E-10	1.60091E-10	4.96106E-10	2.53794E-10
312.0	3.26178E-10	1.60007E-10	4.95852E-10	2.53614E-10
313.0	3.26117E-10	1.59923E-10	4.95599E-10	2.53435E-10
314.0	3.26057E-10	1.59839E-10	4.95347E-10	2.53256E-10
315.0	3.25997E-10	1.59755E-10	4.95095E-10	2.53077E-10
316.0	3.25937E-10	1.59672E-10	4.94844E-10	2.52898E-10
317.0	3.25878E-10	1.59588E-10	4.94594E-10	2.52720E-10
318.0	3.25819E-10	1.59505E-10	4.94345E-10	2.52542E-10
319.0	3.25761E-10	1.59422E-10	4.94096E-10	2.52365E-10
320.0	3.25703E-10	1.59339E-10	4.93848E-10	2.52188E-10
321.0	3.25645E-10	1.59256E-10	4.93601E-10	2.52011E-10
322.0	3.25588E-10	1.59173E-10	4.93355E-10	2.51834E-10
323.0	3.25531E-10	1.59090E-10	4.93109E-10	2.51658E-10
324.0	3.25474E-10	1.59007E-10	4.92864E-10	2.51482E-10
325.0	3.25418E-10	1.58925E-10	4.92619E-10	2.51306E-10

326.0	3.25362E-10	1.58843E-10	4.92376E-10	2.51131E-10
327.0	3.25307E-10	1.58760E-10	4.92133E-10	2.50956E-10
328.0	3.25251E-10	1.58678E-10	4.91891E-10	2.50781E-10
329.0	3.25197E-10	1.58596E-10	4.91649E-10	2.50607E-10
330.0	3.25142E-10	1.58515E-10	4.91408E-10	2.50433E-10
331.0	3.25088E-10	1.58433E-10	4.91168E-10	2.50259E-10
332.0	3.25034E-10	1.58351E-10	4.90929E-10	2.50086E-10
333.0	3.24981E-10	1.58270E-10	4.90691E-10	2.49912E-10
334.0	3.24928E-10	1.58189E-10	4.90453E-10	2.49740E-10
335.0	3.24875E-10	1.58107E-10	4.90216E-10	2.49567E-10
336.0	3.24823E-10	1.58026E-10	4.89979E-10	2.49395E-10
337.0	3.24771E-10	1.57945E-10	4.89743E-10	2.49223E-10
338.0	3.24719E-10	1.57865E-10	4.89508E-10	2.49051E-10
339.0	3.24668E-10	1.57784E-10	4.89274E-10	2.48880E-10
340.0	3.24617E-10	1.57703E-10	4.89040E-10	2.48709E-10
341.0	3.24566E-10	1.57623E-10	4.88807E-10	2.48539E-10
342.0	3.24516E-10	1.57543E-10	4.88575E-10	2.48369E-10
343.0	3.24466E-10	1.57463E-10	4.88344E-10	2.48199E-10
344.0	3.24416E-10	1.57383E-10	4.88113E-10	2.48029E-10
345.0	3.24366E-10	1.57303E-10	4.87883E-10	2.47860E-10
346.0	3.24317E-10	1.57223E-10	4.87653E-10	2.47691E-10
347.0	3.24268E-10	1.57143E-10	4.87425E-10	2.47522E-10
348.0	3.24220E-10	1.57064E-10	4.87197E-10	2.47354E-10
349.0	3.24172E-10	1.56984E-10	4.86969E-10	2.47186E-10
350.0	3.24124E-10	1.56905E-10	4.86743E-10	2.47018E-10
351.0	3.24076E-10	1.56826E-10	4.86517E-10	2.46851E-10
352.0	3.24029E-10	1.56747E-10	4.86291E-10	2.46684E-10
353.0	3.23982E-10	1.56668E-10	4.86067E-10	2.46517E-10
354.0	3.23935E-10	1.56590E-10	4.85843E-10	2.46350E-10
355.0	3.23888E-10	1.56511E-10	4.85620E-10	2.46184E-10
356.0	3.23842E-10	1.56433E-10	4.85397E-10	2.46019E-10
357.0	3.23796E-10	1.56354E-10	4.85175E-10	2.45853E-10
358.0	3.23751E-10	1.56276E-10	4.84954E-10	2.45688E-10
359.0	3.23705E-10	1.56198E-10	4.84734E-10	2.45523E-10
360.0	3.23660E-10	1.56120E-10	4.84514E-10	2.45359E-10
361.0	3.23616E-10	1.56043E-10	4.84295E-10	2.45195E-10
362.0	3.23571E-10	1.55965E-10	4.84076E-10	2.45031E-10
363.0	3.23527E-10	1.55887E-10	4.83859E-10	2.44867E-10
364.0	3.23483E-10	1.55810E-10	4.83642E-10	2.44704E-10
365.0	3.23439E-10	1.55733E-10	4.83425E-10	2.44541E-10
366.0	3.23396E-10	1.55656E-10	4.83209E-10	2.44379E-10
367.0	3.23353E-10	1.55579E-10	4.82994E-10	2.44217E-10
368.0	3.23310E-10	1.55502E-10	4.82780E-10	2.44055E-10
369.0	3.23267E-10	1.55425E-10	4.82566E-10	2.43893E-10
370.0	3.23225E-10	1.55349E-10	4.82353E-10	2.43732E-10
371.0	3.23183E-10	1.55272E-10	4.82140E-10	2.43571E-10
372.0	3.23141E-10	1.55196E-10	4.81928E-10	2.43410E-10
373.0	3.23099E-10	1.55120E-10	4.81717E-10	2.43250E-10
374.0	3.23058E-10	1.55044E-10	4.81507E-10	2.43090E-10
375.0	3.23017E-10	1.54968E-10	4.81297E-10	2.42930E-10

376.0	3.22976E-10	1.54892E-10	4.81087E-10	2.42771E-10
377.0	3.22935E-10	1.54817E-10	4.80879E-10	2.42612E-10
378.0	3.22895E-10	1.54741E-10	4.80671E-10	2.42453E-10
379.0	3.22854E-10	1.54666E-10	4.80463E-10	2.42295E-10
380.0	3.22814E-10	1.54590E-10	4.80257E-10	2.42137E-10
381.0	3.22775E-10	1.54515E-10	4.80051E-10	2.41979E-10
382.0	3.22735E-10	1.54440E-10	4.79845E-10	2.41822E-10
383.0	3.22696E-10	1.54366E-10	4.79640E-10	2.41664E-10
384.0	3.22657E-10	1.54291E-10	4.79436E-10	2.41508E-10
385.0	3.22618E-10	1.54216E-10	4.79233E-10	2.41351E-10
386.0	3.22580E-10	1.54142E-10	4.79030E-10	2.41195E-10
387.0	3.22541E-10	1.54068E-10	4.78827E-10	2.41039E-10
388.0	3.22503E-10	1.53993E-10	4.78626E-10	2.40884E-10
389.0	3.22465E-10	1.53919E-10	4.78425E-10	2.40728E-10
390.0	3.22428E-10	1.53845E-10	4.78224E-10	2.40573E-10
391.0	3.22390E-10	1.53772E-10	4.78024E-10	2.40419E-10
392.0	3.22353E-10	1.53698E-10	4.77825E-10	2.40264E-10
393.0	3.22316E-10	1.53624E-10	4.77626E-10	2.40110E-10
394.0	3.22279E-10	1.53551E-10	4.77428E-10	2.39957E-10
395.0	3.22242E-10	1.53478E-10	4.77231E-10	2.39803E-10
396.0	3.22206E-10	1.53404E-10	4.77034E-10	2.39650E-10
397.0	3.22170E-10	1.53331E-10	4.76838E-10	2.39498E-10
398.0	3.22134E-10	1.53259E-10	4.76642E-10	2.39345E-10
399.0	3.22098E-10	1.53186E-10	4.76447E-10	2.39193E-10
400.0	3.22062E-10	1.53113E-10	4.76252E-10	2.39041E-10
401.0	3.22027E-10	1.53041E-10	4.76058E-10	2.38890E-10
402.0	3.21991E-10	1.52968E-10	4.75865E-10	2.38738E-10
403.0	3.21956E-10	1.52896E-10	4.75672E-10	2.38587E-10
404.0	3.21922E-10	1.52824E-10	4.75480E-10	2.38437E-10
405.0	3.21887E-10	1.52752E-10	4.75289E-10	2.38286E-10
406.0	3.21852E-10	1.52680E-10	4.75098E-10	2.38136E-10
407.0	3.21818E-10	1.52608E-10	4.74907E-10	2.37987E-10
408.0	3.21784E-10	1.52537E-10	4.74717E-10	2.37837E-10
409.0	3.21750E-10	1.52465E-10	4.74528E-10	2.37688E-10
410.0	3.21716E-10	1.52394E-10	4.74339E-10	2.37539E-10
411.0	3.21683E-10	1.52322E-10	4.74151E-10	2.37391E-10
412.0	3.21649E-10	1.52251E-10	4.73963E-10	2.37242E-10
413.0	3.21616E-10	1.52180E-10	4.73776E-10	2.37094E-10
414.0	3.21583E-10	1.52109E-10	4.73590E-10	2.36947E-10
415.0	3.21550E-10	1.52039E-10	4.73404E-10	2.36799E-10
416.0	3.21518E-10	1.51968E-10	4.73219E-10	2.36652E-10
417.0	3.21485E-10	1.51897E-10	4.73034E-10	2.36506E-10
418.0	3.21453E-10	1.51827E-10	4.72849E-10	2.36359E-10
419.0	3.21420E-10	1.51757E-10	4.72666E-10	2.36213E-10
420.0	3.21388E-10	1.51686E-10	4.72482E-10	2.36067E-10
421.0	3.21356E-10	1.51616E-10	4.72300E-10	2.35922E-10
422.0	3.21325E-10	1.51547E-10	4.72118E-10	2.35776E-10
423.0	3.21293E-10	1.51477E-10	4.71936E-10	2.35631E-10
424.0	3.21262E-10	1.51407E-10	4.71755E-10	2.35486E-10
425.0	3.21231E-10	1.51337E-10	4.71574E-10	2.35342E-10

426.0	3.21199E-10	1.51268E-10	4.71394E-10	2.35198E-10
427.0	3.21168E-10	1.51199E-10	4.71215E-10	2.35054E-10
428.0	3.21138E-10	1.51129E-10	4.71036E-10	2.34910E-10
429.0	3.21107E-10	1.51060E-10	4.70857E-10	2.34767E-10
430.0	3.21077E-10	1.50991E-10	4.70679E-10	2.34624E-10
431.0	3.21046E-10	1.50923E-10	4.70502E-10	2.34481E-10
432.0	3.21016E-10	1.50854E-10	4.70325E-10	2.34339E-10
433.0	3.20986E-10	1.50785E-10	4.70149E-10	2.34197E-10
434.0	3.20956E-10	1.50717E-10	4.69973E-10	2.34055E-10
435.0	3.20926E-10	1.50648E-10	4.69797E-10	2.33913E-10
436.0	3.20897E-10	1.50580E-10	4.69622E-10	2.33772E-10
437.0	3.20867E-10	1.50512E-10	4.69448E-10	2.33631E-10
438.0	3.20838E-10	1.50444E-10	4.69274E-10	2.33490E-10
439.0	3.20808E-10	1.50376E-10	4.69101E-10	2.33349E-10
440.0	3.20779E-10	1.50308E-10	4.68928E-10	2.33209E-10
441.0	3.20750E-10	1.50240E-10	4.68755E-10	2.33069E-10
442.0	3.20721E-10	1.50173E-10	4.68583E-10	2.32930E-10
443.0	3.20693E-10	1.50105E-10	4.68412E-10	2.32790E-10
444.0	3.20664E-10	1.50038E-10	4.68241E-10	2.32651E-10
445.0	3.20636E-10	1.49971E-10	4.68070E-10	2.32512E-10
446.0	3.20607E-10	1.49903E-10	4.67900E-10	2.32373E-10
447.0	3.20579E-10	1.49836E-10	4.67731E-10	2.32235E-10
448.0	3.20551E-10	1.49769E-10	4.67562E-10	2.32097E-10
449.0	3.20523E-10	1.49703E-10	4.67393E-10	2.31959E-10
450.0	3.20495E-10	1.49636E-10	4.67225E-10	2.31822E-10
451.0	3.20467E-10	1.49569E-10	4.67057E-10	2.31684E-10
452.0	3.20440E-10	1.49503E-10	4.66890E-10	2.31547E-10
453.0	3.20412E-10	1.49436E-10	4.66723E-10	2.31411E-10
454.0	3.20385E-10	1.49370E-10	4.66557E-10	2.31274E-10
455.0	3.20358E-10	1.49304E-10	4.66391E-10	2.31138E-10
456.0	3.20330E-10	1.49238E-10	4.66226E-10	2.31002E-10
457.0	3.20303E-10	1.49172E-10	4.66061E-10	2.30866E-10
458.0	3.20276E-10	1.49106E-10	4.65896E-10	2.30731E-10
459.0	3.20249E-10	1.49040E-10	4.65732E-10	2.30596E-10
460.0	3.20223E-10	1.48975E-10	4.65569E-10	2.30461E-10
461.0	3.20196E-10	1.48909E-10	4.65405E-10	2.30326E-10
462.0	3.20169E-10	1.48844E-10	4.65243E-10	2.30192E-10
463.0	3.20143E-10	1.48778E-10	4.65080E-10	2.30057E-10
464.0	3.20117E-10	1.48713E-10	4.64918E-10	2.29923E-10
465.0	3.20090E-10	1.48648E-10	4.64757E-10	2.29790E-10
466.0	3.20064E-10	1.48583E-10	4.64596E-10	2.29656E-10
467.0	3.20038E-10	1.48518E-10	4.64435E-10	2.29523E-10
468.0	3.20012E-10	1.48453E-10	4.64275E-10	2.29390E-10
469.0	3.19986E-10	1.48389E-10	4.64116E-10	2.29258E-10
470.0	3.19960E-10	1.48324E-10	4.63956E-10	2.29125E-10
471.0	3.19935E-10	1.48260E-10	4.63797E-10	2.28993E-10
472.0	3.19909E-10	1.48195E-10	4.63639E-10	2.28861E-10
473.0	3.19883E-10	1.48131E-10	4.63481E-10	2.28729E-10
474.0	3.19858E-10	1.48067E-10	4.63323E-10	2.28598E-10
475.0	3.19833E-10	1.48003E-10	4.63166E-10	2.28467E-10

476.0	3.19807E-10	1.47939E-10	4.63009E-10	2.28336E-10
477.0	3.19782E-10	1.47875E-10	4.62852E-10	2.28205E-10
478.0	3.19757E-10	1.47811E-10	4.62696E-10	2.28075E-10
479.0	3.19732E-10	1.47747E-10	4.62541E-10	2.27944E-10
480.0	3.19707E-10	1.47683E-10	4.62385E-10	2.27814E-10
481.0	3.19682E-10	1.47620E-10	4.62230E-10	2.27685E-10
482.0	3.19657E-10	1.47557E-10	4.62076E-10	2.27555E-10
483.0	3.19632E-10	1.47493E-10	4.61922E-10	2.27426E-10
484.0	3.19608E-10	1.47430E-10	4.61768E-10	2.27297E-10
485.0	3.19583E-10	1.47367E-10	4.61615E-10	2.27168E-10
486.0	3.19558E-10	1.47304E-10	4.61462E-10	2.27039E-10
487.0	3.19534E-10	1.47241E-10	4.61309E-10	2.26911E-10
488.0	3.19510E-10	1.47178E-10	4.61157E-10	2.26783E-10
489.0	3.19485E-10	1.47115E-10	4.61005E-10	2.26655E-10
490.0	3.19461E-10	1.47053E-10	4.60854E-10	2.26527E-10
491.0	3.19437E-10	1.46990E-10	4.60703E-10	2.26400E-10
492.0	3.19413E-10	1.46928E-10	4.60552E-10	2.26273E-10
493.0	3.19389E-10	1.46865E-10	4.60402E-10	2.26146E-10
494.0	3.19364E-10	1.46803E-10	4.60252E-10	2.26019E-10
495.0	3.19341E-10	1.46741E-10	4.60102E-10	2.25893E-10
496.0	3.19317E-10	1.46678E-10	4.59953E-10	2.25766E-10
497.0	3.19293E-10	1.46616E-10	4.59804E-10	2.25640E-10
498.0	3.19269E-10	1.46555E-10	4.59655E-10	2.25514E-10
499.0	3.19245E-10	1.46493E-10	4.59507E-10	2.25389E-10
500.0	3.19222E-10	1.46431E-10	4.59359E-10	2.25263E-10

T (K)	31->21	41->21	41->31
5.0	2.44747E-10	1.15203E-10	2.23765E-10
6.0	2.60311E-10	1.14348E-10	2.18589E-10
7.0	2.71791E-10	1.22029E-10	2.30073E-10
8.0	2.82895E-10	1.32925E-10	2.48066E-10
9.0	2.94633E-10	1.44298E-10	2.67348E-10
10.0	3.06936E-10	1.54848E-10	2.85414E-10
11.0	3.19435E-10	1.64043E-10	3.01214E-10
12.0	3.31766E-10	1.71748E-10	3.14449E-10
13.0	3.43663E-10	1.78024E-10	3.25196E-10
14.0	3.54960E-10	1.83020E-10	3.33698E-10
15.0	3.65572E-10	1.86913E-10	3.40260E-10
16.0	3.75471E-10	1.89881E-10	3.45192E-10
17.0	3.84666E-10	1.92089E-10	3.48783E-10
18.0	3.93189E-10	1.93681E-10	3.51289E-10
19.0	4.01082E-10	1.94783E-10	3.52930E-10
20.0	4.08393E-10	1.95500E-10	3.53893E-10
21.0	4.15171E-10	1.95919E-10	3.54333E-10
22.0	4.21461E-10	1.96111E-10	3.54376E-10
23.0	4.27309E-10	1.96136E-10	3.54128E-10
24.0	4.32756E-10	1.96041E-10	3.53670E-10
25.0	4.37839E-10	1.95862E-10	3.53071E-10
26.0	4.42590E-10	1.95631E-10	3.52383E-10
27.0	4.47041E-10	1.95370E-10	3.51648E-10

28.0	4.51218E-10	1.95098E-10	3.50899E-10
29.0	4.55146E-10	1.94829E-10	3.50161E-10
30.0	4.58845E-10	1.94573E-10	3.49452E-10
31.0	4.62336E-10	1.94340E-10	3.48786E-10
32.0	4.65635E-10	1.94133E-10	3.48174E-10
33.0	4.68758E-10	1.93958E-10	3.47622E-10
34.0	4.71718E-10	1.93816E-10	3.47134E-10
35.0	4.74528E-10	1.93710E-10	3.46713E-10
36.0	4.77199E-10	1.93639E-10	3.46360E-10
37.0	4.79741E-10	1.93604E-10	3.46074E-10
38.0	4.82162E-10	1.93603E-10	3.45855E-10
39.0	4.84472E-10	1.93636E-10	3.45699E-10
40.0	4.86677E-10	1.93702E-10	3.45605E-10
41.0	4.88784E-10	1.93799E-10	3.45569E-10
42.0	4.90799E-10	1.93926E-10	3.45590E-10
43.0	4.92728E-10	1.94080E-10	3.45663E-10
44.0	4.94575E-10	1.94260E-10	3.45786E-10
45.0	4.96345E-10	1.94465E-10	3.45955E-10
46.0	4.98043E-10	1.94691E-10	3.46168E-10
47.0	4.99672E-10	1.94939E-10	3.46420E-10
48.0	5.01236E-10	1.95206E-10	3.46709E-10
49.0	5.02737E-10	1.95490E-10	3.47032E-10
50.0	5.04180E-10	1.95791E-10	3.47386E-10
51.0	5.05566E-10	1.96105E-10	3.47769E-10
52.0	5.06899E-10	1.96433E-10	3.48177E-10
53.0	5.08180E-10	1.96773E-10	3.48609E-10
54.0	5.09411E-10	1.97123E-10	3.49062E-10
55.0	5.10596E-10	1.97483E-10	3.49535E-10
56.0	5.11735E-10	1.97850E-10	3.50024E-10
57.0	5.12831E-10	1.98225E-10	3.50529E-10
58.0	5.13885E-10	1.98607E-10	3.51046E-10
59.0	5.14899E-10	1.98993E-10	3.51576E-10
60.0	5.15874E-10	1.99384E-10	3.52116E-10
61.0	5.16811E-10	1.99779E-10	3.52664E-10
62.0	5.17712E-10	2.00176E-10	3.53219E-10
63.0	5.18578E-10	2.00576E-10	3.53781E-10
64.0	5.19410E-10	2.00977E-10	3.54347E-10
65.0	5.20209E-10	2.01380E-10	3.54918E-10
66.0	5.20977E-10	2.01782E-10	3.55491E-10
67.0	5.21714E-10	2.02185E-10	3.56066E-10
68.0	5.22421E-10	2.02588E-10	3.56642E-10
69.0	5.23099E-10	2.02989E-10	3.57218E-10
70.0	5.23748E-10	2.03389E-10	3.57794E-10
71.0	5.24371E-10	2.03787E-10	3.58369E-10
72.0	5.24967E-10	2.04184E-10	3.58941E-10
73.0	5.25537E-10	2.04578E-10	3.59512E-10
74.0	5.26082E-10	2.04969E-10	3.60079E-10
75.0	5.26602E-10	2.05358E-10	3.60643E-10
76.0	5.27099E-10	2.05743E-10	3.61204E-10
77.0	5.27573E-10	2.06126E-10	3.61760E-10

78.0	5.28024E-10	2.06504E-10	3.62311E-10
79.0	5.28453E-10	2.06879E-10	3.62858E-10
80.0	5.28861E-10	2.07251E-10	3.63400E-10
81.0	5.29248E-10	2.07618E-10	3.63935E-10
82.0	5.29615E-10	2.07981E-10	3.64466E-10
83.0	5.29962E-10	2.08340E-10	3.64990E-10
84.0	5.30289E-10	2.08695E-10	3.65508E-10
85.0	5.30598E-10	2.09045E-10	3.66020E-10
86.0	5.30889E-10	2.09391E-10	3.66525E-10
87.0	5.31162E-10	2.09733E-10	3.67024E-10
88.0	5.31417E-10	2.10069E-10	3.67516E-10
89.0	5.31655E-10	2.10401E-10	3.68001E-10
90.0	5.31877E-10	2.10729E-10	3.68479E-10
91.0	5.32083E-10	2.11052E-10	3.68950E-10
92.0	5.32273E-10	2.11370E-10	3.69414E-10
93.0	5.32448E-10	2.11683E-10	3.69871E-10
94.0	5.32607E-10	2.11992E-10	3.70321E-10
95.0	5.32752E-10	2.12295E-10	3.70764E-10
96.0	5.32883E-10	2.12594E-10	3.71199E-10
97.0	5.33000E-10	2.12889E-10	3.71627E-10
98.0	5.33103E-10	2.13178E-10	3.72048E-10
99.0	5.33194E-10	2.13463E-10	3.72461E-10
100.0	5.33271E-10	2.13743E-10	3.72868E-10
101.0	5.33336E-10	2.14018E-10	3.73267E-10
102.0	5.33388E-10	2.14289E-10	3.73659E-10
103.0	5.33429E-10	2.14554E-10	3.74043E-10
104.0	5.33457E-10	2.14816E-10	3.74421E-10
105.0	5.33475E-10	2.15072E-10	3.74791E-10
106.0	5.33481E-10	2.15324E-10	3.75155E-10
107.0	5.33477E-10	2.15571E-10	3.75511E-10
108.0	5.33462E-10	2.15814E-10	3.75860E-10
109.0	5.33437E-10	2.16053E-10	3.76202E-10
110.0	5.33402E-10	2.16287E-10	3.76537E-10
111.0	5.33357E-10	2.16516E-10	3.76866E-10
112.0	5.33302E-10	2.16741E-10	3.77188E-10
113.0	5.33238E-10	2.16962E-10	3.77503E-10
114.0	5.33165E-10	2.17178E-10	3.77811E-10
115.0	5.33084E-10	2.17391E-10	3.78112E-10
116.0	5.32993E-10	2.17598E-10	3.78407E-10
117.0	5.32895E-10	2.17802E-10	3.78696E-10
118.0	5.32788E-10	2.18002E-10	3.78978E-10
119.0	5.32673E-10	2.18197E-10	3.79254E-10
120.0	5.32550E-10	2.18389E-10	3.79523E-10
121.0	5.32420E-10	2.18576E-10	3.79786E-10
122.0	5.32283E-10	2.18760E-10	3.80043E-10
123.0	5.32138E-10	2.18940E-10	3.80294E-10
124.0	5.31986E-10	2.19115E-10	3.80539E-10
125.0	5.31828E-10	2.19287E-10	3.80778E-10
126.0	5.31663E-10	2.19455E-10	3.81011E-10
127.0	5.31491E-10	2.19620E-10	3.81238E-10

128.0	5.31313E-10	2.19781E-10	3.81460E-10
129.0	5.31129E-10	2.19938E-10	3.81676E-10
130.0	5.30939E-10	2.20091E-10	3.81886E-10
131.0	5.30743E-10	2.20241E-10	3.82091E-10
132.0	5.30542E-10	2.20388E-10	3.82290E-10
133.0	5.30335E-10	2.20531E-10	3.82484E-10
134.0	5.30123E-10	2.20670E-10	3.82672E-10
135.0	5.29905E-10	2.20806E-10	3.82855E-10
136.0	5.29683E-10	2.20939E-10	3.83033E-10
137.0	5.29455E-10	2.21069E-10	3.83206E-10
138.0	5.29223E-10	2.21195E-10	3.83374E-10
139.0	5.28986E-10	2.21318E-10	3.83537E-10
140.0	5.28744E-10	2.21438E-10	3.83695E-10
141.0	5.28498E-10	2.21555E-10	3.83848E-10
142.0	5.28248E-10	2.21669E-10	3.83996E-10
143.0	5.27993E-10	2.21780E-10	3.84140E-10
144.0	5.27735E-10	2.21888E-10	3.84279E-10
145.0	5.27472E-10	2.21993E-10	3.84413E-10
146.0	5.27206E-10	2.22095E-10	3.84543E-10
147.0	5.26936E-10	2.22194E-10	3.84668E-10
148.0	5.26662E-10	2.22290E-10	3.84789E-10
149.0	5.26385E-10	2.22384E-10	3.84906E-10
150.0	5.26104E-10	2.22475E-10	3.85018E-10
151.0	5.25820E-10	2.22563E-10	3.85127E-10
152.0	5.25533E-10	2.22648E-10	3.85231E-10
153.0	5.25243E-10	2.22731E-10	3.85331E-10
154.0	5.24949E-10	2.22812E-10	3.85427E-10
155.0	5.24653E-10	2.22889E-10	3.85519E-10
156.0	5.24354E-10	2.22965E-10	3.85607E-10
157.0	5.24052E-10	2.23037E-10	3.85691E-10
158.0	5.23747E-10	2.23108E-10	3.85772E-10
159.0	5.23440E-10	2.23176E-10	3.85849E-10
160.0	5.23130E-10	2.23241E-10	3.85922E-10
161.0	5.22818E-10	2.23304E-10	3.85991E-10
162.0	5.22503E-10	2.23365E-10	3.86057E-10
163.0	5.22186E-10	2.23424E-10	3.86120E-10
164.0	5.21867E-10	2.23480E-10	3.86179E-10
165.0	5.21546E-10	2.23535E-10	3.86235E-10
166.0	5.21222E-10	2.23587E-10	3.86287E-10
167.0	5.20897E-10	2.23637E-10	3.86336E-10
168.0	5.20570E-10	2.23685E-10	3.86382E-10
169.0	5.20240E-10	2.23730E-10	3.86425E-10
170.0	5.19909E-10	2.23774E-10	3.86464E-10
171.0	5.19576E-10	2.23816E-10	3.86501E-10
172.0	5.19242E-10	2.23856E-10	3.86534E-10
173.0	5.18905E-10	2.23894E-10	3.86564E-10
174.0	5.18568E-10	2.23930E-10	3.86592E-10
175.0	5.18228E-10	2.23964E-10	3.86617E-10
176.0	5.17888E-10	2.23996E-10	3.86638E-10
177.0	5.17545E-10	2.24027E-10	3.86657E-10

178.0	5.17202E-10	2.24055E-10	3.86674E-10
179.0	5.16857E-10	2.24082E-10	3.86687E-10
180.0	5.16511E-10	2.24107E-10	3.86698E-10
181.0	5.16163E-10	2.24131E-10	3.86707E-10
182.0	5.15815E-10	2.24152E-10	3.86712E-10
183.0	5.15465E-10	2.24173E-10	3.86716E-10
184.0	5.15114E-10	2.24191E-10	3.86716E-10
185.0	5.14763E-10	2.24208E-10	3.86715E-10
186.0	5.14410E-10	2.24223E-10	3.86710E-10
187.0	5.14056E-10	2.24237E-10	3.86704E-10
188.0	5.13702E-10	2.24249E-10	3.86695E-10
189.0	5.13346E-10	2.24260E-10	3.86684E-10
190.0	5.12990E-10	2.24269E-10	3.86671E-10
191.0	5.12633E-10	2.24277E-10	3.86656E-10
192.0	5.12275E-10	2.24283E-10	3.86638E-10
193.0	5.11917E-10	2.24288E-10	3.86618E-10
194.0	5.11558E-10	2.24292E-10	3.86596E-10
195.0	5.11198E-10	2.24294E-10	3.86573E-10
196.0	5.10838E-10	2.24295E-10	3.86547E-10
197.0	5.10477E-10	2.24295E-10	3.86519E-10
198.0	5.10115E-10	2.24293E-10	3.86489E-10
199.0	5.09754E-10	2.24290E-10	3.86457E-10
200.0	5.09391E-10	2.24286E-10	3.86424E-10
201.0	5.09028E-10	2.24280E-10	3.86388E-10
202.0	5.08665E-10	2.24274E-10	3.86351E-10
203.0	5.08302E-10	2.24266E-10	3.86312E-10
204.0	5.07938E-10	2.24257E-10	3.86272E-10
205.0	5.07574E-10	2.24246E-10	3.86229E-10
206.0	5.07209E-10	2.24235E-10	3.86185E-10
207.0	5.06845E-10	2.24223E-10	3.86139E-10
208.0	5.06480E-10	2.24209E-10	3.86092E-10
209.0	5.06114E-10	2.24195E-10	3.86043E-10
210.0	5.05749E-10	2.24179E-10	3.85993E-10
211.0	5.05384E-10	2.24162E-10	3.85941E-10
212.0	5.05018E-10	2.24145E-10	3.85887E-10
213.0	5.04653E-10	2.24126E-10	3.85832E-10
214.0	5.04287E-10	2.24106E-10	3.85776E-10
215.0	5.03921E-10	2.24085E-10	3.85718E-10
216.0	5.03555E-10	2.24064E-10	3.85659E-10
217.0	5.03189E-10	2.24041E-10	3.85598E-10
218.0	5.02824E-10	2.24018E-10	3.85537E-10
219.0	5.02458E-10	2.23994E-10	3.85473E-10
220.0	5.02092E-10	2.23968E-10	3.85409E-10
221.0	5.01726E-10	2.23942E-10	3.85343E-10
222.0	5.01361E-10	2.23915E-10	3.85276E-10
223.0	5.00996E-10	2.23887E-10	3.85208E-10
224.0	5.00630E-10	2.23859E-10	3.85139E-10
225.0	5.00265E-10	2.23829E-10	3.85069E-10
226.0	4.99900E-10	2.23799E-10	3.84997E-10
227.0	4.99535E-10	2.23768E-10	3.84924E-10

228.0	4.99171E-10	2.23736E-10	3.84850E-10
229.0	4.98806E-10	2.23704E-10	3.84776E-10
230.0	4.98442E-10	2.23670E-10	3.84700E-10
231.0	4.98079E-10	2.23636E-10	3.84623E-10
232.0	4.97715E-10	2.23602E-10	3.84545E-10
233.0	4.97352E-10	2.23566E-10	3.84466E-10
234.0	4.96989E-10	2.23530E-10	3.84386E-10
235.0	4.96626E-10	2.23493E-10	3.84306E-10
236.0	4.96264E-10	2.23456E-10	3.84224E-10
237.0	4.95902E-10	2.23418E-10	3.84141E-10
238.0	4.95540E-10	2.23379E-10	3.84058E-10
239.0	4.95179E-10	2.23340E-10	3.83974E-10
240.0	4.94818E-10	2.23300E-10	3.83889E-10
241.0	4.94457E-10	2.23259E-10	3.83803E-10
242.0	4.94097E-10	2.23218E-10	3.83716E-10
243.0	4.93738E-10	2.23176E-10	3.83628E-10
244.0	4.93378E-10	2.23134E-10	3.83540E-10
245.0	4.93020E-10	2.23091E-10	3.83451E-10
246.0	4.92661E-10	2.23048E-10	3.83361E-10
247.0	4.92303E-10	2.23004E-10	3.83271E-10
248.0	4.91946E-10	2.22959E-10	3.83180E-10
249.0	4.91589E-10	2.22914E-10	3.83088E-10
250.0	4.91233E-10	2.22869E-10	3.82995E-10
251.0	4.90877E-10	2.22823E-10	3.82902E-10
252.0	4.90522E-10	2.22776E-10	3.82808E-10
253.0	4.90167E-10	2.22729E-10	3.82714E-10
254.0	4.89812E-10	2.22682E-10	3.82619E-10
255.0	4.89459E-10	2.22634E-10	3.82523E-10
256.0	4.89106E-10	2.22585E-10	3.82427E-10
257.0	4.88753E-10	2.22537E-10	3.82330E-10
258.0	4.88401E-10	2.22487E-10	3.82233E-10
259.0	4.88049E-10	2.22438E-10	3.82135E-10
260.0	4.87698E-10	2.22388E-10	3.82037E-10
261.0	4.87348E-10	2.22337E-10	3.81938E-10
262.0	4.86999E-10	2.22286E-10	3.81838E-10
263.0	4.86649E-10	2.22235E-10	3.81739E-10
264.0	4.86301E-10	2.22183E-10	3.81638E-10
265.0	4.85953E-10	2.22131E-10	3.81538E-10
266.0	4.85606E-10	2.22079E-10	3.81436E-10
267.0	4.85259E-10	2.22026E-10	3.81335E-10
268.0	4.84914E-10	2.21973E-10	3.81233E-10
269.0	4.84568E-10	2.21920E-10	3.81130E-10
270.0	4.84224E-10	2.21866E-10	3.81028E-10
271.0	4.83880E-10	2.21812E-10	3.80924E-10
272.0	4.83537E-10	2.21757E-10	3.80821E-10
273.0	4.83194E-10	2.21703E-10	3.80717E-10
274.0	4.82852E-10	2.21648E-10	3.80613E-10
275.0	4.82511E-10	2.21592E-10	3.80508E-10
276.0	4.82170E-10	2.21537E-10	3.80403E-10
277.0	4.81831E-10	2.21481E-10	3.80298E-10

278.0	4.81491E-10	2.21424E-10	3.80192E-10
279.0	4.81153E-10	2.21368E-10	3.80086E-10
280.0	4.80815E-10	2.21311E-10	3.79980E-10
281.0	4.80478E-10	2.21254E-10	3.79874E-10
282.0	4.80142E-10	2.21197E-10	3.79767E-10
283.0	4.79807E-10	2.21139E-10	3.79660E-10
284.0	4.79472E-10	2.21081E-10	3.79553E-10
285.0	4.79138E-10	2.21023E-10	3.79445E-10
286.0	4.78804E-10	2.20965E-10	3.79337E-10
287.0	4.78472E-10	2.20907E-10	3.79229E-10
288.0	4.78140E-10	2.20848E-10	3.79121E-10
289.0	4.77809E-10	2.20789E-10	3.79013E-10
290.0	4.77478E-10	2.20730E-10	3.78904E-10
291.0	4.77149E-10	2.20670E-10	3.78795E-10
292.0	4.76820E-10	2.20611E-10	3.78687E-10
293.0	4.76492E-10	2.20551E-10	3.78577E-10
294.0	4.76165E-10	2.20491E-10	3.78468E-10
295.0	4.75838E-10	2.20431E-10	3.78358E-10
296.0	4.75512E-10	2.20370E-10	3.78249E-10
297.0	4.75187E-10	2.20310E-10	3.78139E-10
298.0	4.74863E-10	2.20249E-10	3.78029E-10
299.0	4.74540E-10	2.20188E-10	3.77919E-10
300.0	4.74217E-10	2.20127E-10	3.77809E-10
301.0	4.73895E-10	2.20066E-10	3.77698E-10
302.0	4.73574E-10	2.20005E-10	3.77588E-10
303.0	4.73253E-10	2.19943E-10	3.77477E-10
304.0	4.72934E-10	2.19881E-10	3.77366E-10
305.0	4.72615E-10	2.19819E-10	3.77256E-10
306.0	4.72297E-10	2.19757E-10	3.77145E-10
307.0	4.71980E-10	2.19695E-10	3.77034E-10
308.0	4.71664E-10	2.19633E-10	3.76923E-10
309.0	4.71348E-10	2.19571E-10	3.76811E-10
310.0	4.71033E-10	2.19508E-10	3.76700E-10
311.0	4.70719E-10	2.19445E-10	3.76589E-10
312.0	4.70406E-10	2.19382E-10	3.76477E-10
313.0	4.70094E-10	2.19320E-10	3.76366E-10
314.0	4.69782E-10	2.19256E-10	3.76254E-10
315.0	4.69471E-10	2.19193E-10	3.76143E-10
316.0	4.69161E-10	2.19130E-10	3.76031E-10
317.0	4.68852E-10	2.19067E-10	3.75920E-10
318.0	4.68543E-10	2.19003E-10	3.75808E-10
319.0	4.68236E-10	2.18940E-10	3.75696E-10
320.0	4.67929E-10	2.18876E-10	3.75584E-10
321.0	4.67623E-10	2.18812E-10	3.75473E-10
322.0	4.67318E-10	2.18748E-10	3.75361E-10
323.0	4.67013E-10	2.18684E-10	3.75249E-10
324.0	4.66710E-10	2.18620E-10	3.75137E-10
325.0	4.66407E-10	2.18556E-10	3.75025E-10
326.0	4.66105E-10	2.18492E-10	3.74913E-10
327.0	4.65804E-10	2.18427E-10	3.74802E-10

328.0	4.65504E-10	2.18363E-10	3.74690E-10
329.0	4.65204E-10	2.18299E-10	3.74578E-10
330.0	4.64905E-10	2.18234E-10	3.74466E-10
331.0	4.64607E-10	2.18169E-10	3.74354E-10
332.0	4.64310E-10	2.18105E-10	3.74242E-10
333.0	4.64014E-10	2.18040E-10	3.74130E-10
334.0	4.63718E-10	2.17975E-10	3.74019E-10
335.0	4.63424E-10	2.17910E-10	3.73907E-10
336.0	4.63130E-10	2.17845E-10	3.73795E-10
337.0	4.62836E-10	2.17780E-10	3.73683E-10
338.0	4.62544E-10	2.17715E-10	3.73572E-10
339.0	4.62253E-10	2.17650E-10	3.73460E-10
340.0	4.61962E-10	2.17585E-10	3.73348E-10
341.0	4.61672E-10	2.17520E-10	3.73237E-10
342.0	4.61383E-10	2.17454E-10	3.73125E-10
343.0	4.61095E-10	2.17389E-10	3.73014E-10
344.0	4.60807E-10	2.17324E-10	3.72902E-10
345.0	4.60520E-10	2.17258E-10	3.72791E-10
346.0	4.60234E-10	2.17193E-10	3.72679E-10
347.0	4.59949E-10	2.17127E-10	3.72568E-10
348.0	4.59665E-10	2.17062E-10	3.72457E-10
349.0	4.59381E-10	2.16996E-10	3.72345E-10
350.0	4.59099E-10	2.16931E-10	3.72234E-10
351.0	4.58817E-10	2.16865E-10	3.72123E-10
352.0	4.58536E-10	2.16799E-10	3.72012E-10
353.0	4.58255E-10	2.16734E-10	3.71901E-10
354.0	4.57976E-10	2.16668E-10	3.71790E-10
355.0	4.57697E-10	2.16602E-10	3.71679E-10
356.0	4.57419E-10	2.16536E-10	3.71568E-10
357.0	4.57142E-10	2.16470E-10	3.71458E-10
358.0	4.56865E-10	2.16405E-10	3.71347E-10
359.0	4.56589E-10	2.16339E-10	3.71236E-10
360.0	4.56314E-10	2.16273E-10	3.71126E-10
361.0	4.56040E-10	2.16207E-10	3.71015E-10
362.0	4.55767E-10	2.16141E-10	3.70905E-10
363.0	4.55494E-10	2.16075E-10	3.70795E-10
364.0	4.55223E-10	2.16009E-10	3.70684E-10
365.0	4.54952E-10	2.15943E-10	3.70574E-10
366.0	4.54681E-10	2.15877E-10	3.70464E-10
367.0	4.54412E-10	2.15811E-10	3.70354E-10
368.0	4.54143E-10	2.15745E-10	3.70244E-10
369.0	4.53875E-10	2.15679E-10	3.70134E-10
370.0	4.53608E-10	2.15613E-10	3.70024E-10
371.0	4.53342E-10	2.15547E-10	3.69915E-10
372.0	4.53076E-10	2.15481E-10	3.69805E-10
373.0	4.52811E-10	2.15414E-10	3.69696E-10
374.0	4.52547E-10	2.15348E-10	3.69586E-10
375.0	4.52284E-10	2.15282E-10	3.69477E-10
376.0	4.52021E-10	2.15216E-10	3.69367E-10
377.0	4.51759E-10	2.15150E-10	3.69258E-10

378.0	4.51498E-10	2.15084E-10	3.69149E-10
379.0	4.51238E-10	2.15018E-10	3.69040E-10
380.0	4.50978E-10	2.14951E-10	3.68931E-10
381.0	4.50719E-10	2.14885E-10	3.68822E-10
382.0	4.50461E-10	2.14819E-10	3.68714E-10
383.0	4.50203E-10	2.14753E-10	3.68605E-10
384.0	4.49947E-10	2.14687E-10	3.68496E-10
385.0	4.49691E-10	2.14621E-10	3.68388E-10
386.0	4.49436E-10	2.14554E-10	3.68279E-10
387.0	4.49181E-10	2.14488E-10	3.68171E-10
388.0	4.48927E-10	2.14422E-10	3.68063E-10
389.0	4.48674E-10	2.14356E-10	3.67955E-10
390.0	4.48422E-10	2.14290E-10	3.67846E-10
391.0	4.48170E-10	2.14223E-10	3.67738E-10
392.0	4.47919E-10	2.14157E-10	3.67631E-10
393.0	4.47669E-10	2.14091E-10	3.67523E-10
394.0	4.47420E-10	2.14025E-10	3.67415E-10
395.0	4.47171E-10	2.13959E-10	3.67307E-10
396.0	4.46923E-10	2.13892E-10	3.67200E-10
397.0	4.46676E-10	2.13826E-10	3.67092E-10
398.0	4.46429E-10	2.13760E-10	3.66985E-10
399.0	4.46183E-10	2.13694E-10	3.66878E-10
400.0	4.45938E-10	2.13628E-10	3.66771E-10
401.0	4.45693E-10	2.13562E-10	3.66663E-10
402.0	4.45450E-10	2.13495E-10	3.66556E-10
403.0	4.45207E-10	2.13429E-10	3.66450E-10
404.0	4.44964E-10	2.13363E-10	3.66343E-10
405.0	4.44722E-10	2.13297E-10	3.66236E-10
406.0	4.44481E-10	2.13231E-10	3.66129E-10
407.0	4.44241E-10	2.13165E-10	3.66023E-10
408.0	4.44001E-10	2.13098E-10	3.65916E-10
409.0	4.43762E-10	2.13032E-10	3.65810E-10
410.0	4.43524E-10	2.12966E-10	3.65703E-10
411.0	4.43286E-10	2.12900E-10	3.65597E-10
412.0	4.43049E-10	2.12834E-10	3.65491E-10
413.0	4.42813E-10	2.12768E-10	3.65385E-10
414.0	4.42577E-10	2.12702E-10	3.65279E-10
415.0	4.42342E-10	2.12636E-10	3.65173E-10
416.0	4.42108E-10	2.12570E-10	3.65067E-10
417.0	4.41874E-10	2.12503E-10	3.64962E-10
418.0	4.41641E-10	2.12437E-10	3.64856E-10
419.0	4.41409E-10	2.12371E-10	3.64750E-10
420.0	4.41177E-10	2.12305E-10	3.64645E-10
421.0	4.40946E-10	2.12239E-10	3.64540E-10
422.0	4.40716E-10	2.12173E-10	3.64434E-10
423.0	4.40486E-10	2.12107E-10	3.64329E-10
424.0	4.40257E-10	2.12041E-10	3.64224E-10
425.0	4.40028E-10	2.11975E-10	3.64119E-10
426.0	4.39800E-10	2.11909E-10	3.64014E-10
427.0	4.39573E-10	2.11843E-10	3.63909E-10

428.0	4.39347E-10	2.11777E-10	3.63804E-10
429.0	4.39121E-10	2.11711E-10	3.63699E-10
430.0	4.38895E-10	2.11645E-10	3.63594E-10
431.0	4.38671E-10	2.11579E-10	3.63490E-10
432.0	4.38446E-10	2.11513E-10	3.63385E-10
433.0	4.38223E-10	2.11447E-10	3.63281E-10
434.0	4.38000E-10	2.11381E-10	3.63176E-10
435.0	4.37778E-10	2.11315E-10	3.63072E-10
436.0	4.37556E-10	2.11249E-10	3.62968E-10
437.0	4.37335E-10	2.11183E-10	3.62864E-10
438.0	4.37115E-10	2.11117E-10	3.62760E-10
439.0	4.36895E-10	2.11051E-10	3.62656E-10
440.0	4.36676E-10	2.10985E-10	3.62552E-10
441.0	4.36457E-10	2.10919E-10	3.62448E-10
442.0	4.36239E-10	2.10854E-10	3.62344E-10
443.0	4.36021E-10	2.10788E-10	3.62240E-10
444.0	4.35805E-10	2.10722E-10	3.62136E-10
445.0	4.35588E-10	2.10656E-10	3.62033E-10
446.0	4.35373E-10	2.10590E-10	3.61929E-10
447.0	4.35158E-10	2.10524E-10	3.61826E-10
448.0	4.34943E-10	2.10458E-10	3.61722E-10
449.0	4.34729E-10	2.10392E-10	3.61619E-10
450.0	4.34516E-10	2.10326E-10	3.61516E-10
451.0	4.34303E-10	2.10261E-10	3.61412E-10
452.0	4.34091E-10	2.10195E-10	3.61309E-10
453.0	4.33879E-10	2.10129E-10	3.61206E-10
454.0	4.33668E-10	2.10063E-10	3.61103E-10
455.0	4.33457E-10	2.09997E-10	3.61000E-10
456.0	4.33247E-10	2.09931E-10	3.60897E-10
457.0	4.33038E-10	2.09865E-10	3.60794E-10
458.0	4.32829E-10	2.09800E-10	3.60691E-10
459.0	4.32621E-10	2.09734E-10	3.60588E-10
460.0	4.32413E-10	2.09668E-10	3.60486E-10
461.0	4.32205E-10	2.09602E-10	3.60383E-10
462.0	4.31999E-10	2.09536E-10	3.60280E-10
463.0	4.31793E-10	2.09471E-10	3.60178E-10
464.0	4.31587E-10	2.09405E-10	3.60075E-10
465.0	4.31382E-10	2.09339E-10	3.59973E-10
466.0	4.31177E-10	2.09273E-10	3.59870E-10
467.0	4.30973E-10	2.09207E-10	3.59768E-10
468.0	4.30770E-10	2.09142E-10	3.59666E-10
469.0	4.30567E-10	2.09076E-10	3.59563E-10
470.0	4.30364E-10	2.09010E-10	3.59461E-10
471.0	4.30162E-10	2.08944E-10	3.59359E-10
472.0	4.29961E-10	2.08878E-10	3.59257E-10
473.0	4.29760E-10	2.08813E-10	3.59155E-10
474.0	4.29559E-10	2.08747E-10	3.59053E-10
475.0	4.29360E-10	2.08681E-10	3.58951E-10
476.0	4.29160E-10	2.08615E-10	3.58849E-10
477.0	4.28961E-10	2.08549E-10	3.58747E-10

478.0	4.28763E-10	2.08484E-10	3.58645E-10
479.0	4.28565E-10	2.08418E-10	3.58543E-10
480.0	4.28367E-10	2.08352E-10	3.58441E-10
481.0	4.28171E-10	2.08286E-10	3.58340E-10
482.0	4.27974E-10	2.08221E-10	3.58238E-10
483.0	4.27778E-10	2.08155E-10	3.58136E-10
484.0	4.27583E-10	2.08089E-10	3.58035E-10
485.0	4.27388E-10	2.08023E-10	3.57933E-10
486.0	4.27193E-10	2.07957E-10	3.57831E-10
487.0	4.26999E-10	2.07892E-10	3.57730E-10
488.0	4.26806E-10	2.07826E-10	3.57628E-10
489.0	4.26613E-10	2.07760E-10	3.57527E-10
490.0	4.26420E-10	2.07694E-10	3.57425E-10
491.0	4.26228E-10	2.07628E-10	3.57324E-10
492.0	4.26036E-10	2.07563E-10	3.57222E-10
493.0	4.25845E-10	2.07497E-10	3.57121E-10
494.0	4.25654E-10	2.07431E-10	3.57020E-10
495.0	4.25464E-10	2.07365E-10	3.56918E-10
496.0	4.25274E-10	2.07299E-10	3.56817E-10
497.0	4.25085E-10	2.07234E-10	3.56716E-10
498.0	4.24896E-10	2.07168E-10	3.56615E-10
499.0	4.24707E-10	2.07102E-10	3.56513E-10
500.0	4.24519E-10	2.07036E-10	3.56412E-10

Table S10: Computed excitation and de-excitation rate coefficients in cm^3 $molecule^{-1}$ s^{-1} units for inelastic processes generated using the 4D RR-PES for the $HeH^+(j_1 = 0)\cdot\cdot\cdot$ ortho-$H_2(j_2)$ system with $\Delta j_2 = +2$ and $\Delta j_2 = -2$.

T (K)	01->03	03->01
5.0	1.51507E-86	6.23559E-11
6.0	6.25237E-74	5.58152E-11
7.0	6.78907E-65	5.45747E-11
8.0	4.24733E-58	5.61245E-11
9.0	8.44095E-53	5.90025E-11
10.0	1.48539E-48	6.23843E-11
11.0	4.45567E-45	6.58264E-11
12.0	3.53305E-42	6.91059E-11
13.0	1.00472E-39	7.21235E-11
14.0	1.27519E-37	7.48473E-11
15.0	8.48336E-36	7.72795E-11
16.0	3.33814E-34	7.94389E-11
17.0	8.52253E-33	8.13508E-11
18.0	1.51721E-31	8.30416E-11
19.0	1.99365E-30	8.45366E-11
20.0	2.02375E-29	8.58587E-11
21.0	1.64659E-28	8.70283E-11
22.0	1.10672E-27	8.80629E-11
23.0	6.30003E-27	8.89779E-11
24.0	3.10112E-26	8.97865E-11
25.0	1.34321E-25	9.05002E-11
26.0	5.19553E-25	9.11289E-11

27.0	1.81735E-24	9.16813E-11
28.0	5.81104E-24	9.21649E-11
29.0	1.71438E-23	9.25864E-11
30.0	4.70434E-23	9.29519E-11
31.0	1.20913E-22	9.32665E-11
32.0	2.92886E-22	9.35349E-11
33.0	6.72225E-22	9.37615E-11
34.0	1.46889E-21	9.39501E-11
35.0	3.06870E-21	9.41040E-11
36.0	6.15229E-21	9.42265E-11
37.0	1.18765E-20	9.43204E-11
38.0	2.21416E-20	9.43883E-11
39.0	3.99727E-20	9.44325E-11
40.0	7.00489E-20	9.44553E-11
41.0	1.19418E-19	9.44587E-11
42.0	1.98439E-19	9.44443E-11
43.0	3.21995E-19	9.44140E-11
44.0	5.11025E-19	9.43692E-11
45.0	7.94420E-19	9.43113E-11
46.0	1.21133E-18	9.42417E-11
47.0	1.81390E-18	9.41614E-11
48.0	2.67055E-18	9.40716E-11
49.0	3.86966E-18	9.39732E-11
50.0	5.52394E-18	9.38671E-11
51.0	7.77520E-18	9.37543E-11
52.0	1.07998E-17	9.36353E-11
53.0	1.48145E-17	9.35110E-11
54.0	2.00831E-17	9.33820E-11
55.0	2.69233E-17	9.32488E-11
56.0	3.57140E-17	9.31120E-11
57.0	4.69035E-17	9.29720E-11
58.0	6.10175E-17	9.28294E-11
59.0	7.86677E-17	9.26845E-11
60.0	1.00561E-16	9.25377E-11
61.0	1.27506E-16	9.23893E-11
62.0	1.60428E-16	9.22396E-11
63.0	2.00371E-16	9.20890E-11
64.0	2.48510E-16	9.19376E-11
65.0	3.06162E-16	9.17858E-11
66.0	3.74790E-16	9.16336E-11
67.0	4.56016E-16	9.14814E-11
68.0	5.51626E-16	9.13292E-11
69.0	6.63577E-16	9.11773E-11
70.0	7.94010E-16	9.10258E-11
71.0	9.45248E-16	9.08748E-11
72.0	1.11981E-15	9.07244E-11
73.0	1.32041E-15	9.05747E-11
74.0	1.54997E-15	9.04259E-11
75.0	1.81161E-15	9.02779E-11
76.0	2.10867E-15	9.01309E-11

77.0	2.44469E-15	8.99850E-11
78.0	2.82345E-15	8.98401E-11
79.0	3.24892E-15	8.96964E-11
80.0	3.72529E-15	8.95539E-11
81.0	4.25700E-15	8.94126E-11
82.0	4.84865E-15	8.92726E-11
83.0	5.50511E-15	8.91339E-11
84.0	6.23141E-15	8.89964E-11
85.0	7.03284E-15	8.88603E-11
86.0	7.91486E-15	8.87255E-11
87.0	8.88314E-15	8.85921E-11
88.0	9.94355E-15	8.84601E-11
89.0	1.11021E-14	8.83294E-11
90.0	1.23652E-14	8.82000E-11
91.0	1.37391E-14	8.80721E-11
92.0	1.52304E-14	8.79455E-11
93.0	1.68459E-14	8.78202E-11
94.0	1.85926E-14	8.76963E-11
95.0	2.04775E-14	8.75738E-11
96.0	2.25077E-14	8.74526E-11
97.0	2.46908E-14	8.73327E-11
98.0	2.70340E-14	8.72142E-11
99.0	2.95450E-14	8.70969E-11
100.0	3.22315E-14	8.69809E-11
101.0	3.51012E-14	8.68663E-11
102.0	3.81621E-14	8.67528E-11
103.0	4.14221E-14	8.66407E-11
104.0	4.48891E-14	8.65297E-11
105.0	4.85714E-14	8.64200E-11
106.0	5.24770E-14	8.63114E-11
107.0	5.66141E-14	8.62041E-11
108.0	6.09909E-14	8.60979E-11
109.0	6.56156E-14	8.59928E-11
110.0	7.04965E-14	8.58889E-11
111.0	7.56419E-14	8.57861E-11
112.0	8.10599E-14	8.56843E-11
113.0	8.67589E-14	8.55837E-11
114.0	9.27470E-14	8.54841E-11
115.0	9.90324E-14	8.53855E-11
116.0	1.05623E-13	8.52879E-11
117.0	1.12528E-13	8.51913E-11
118.0	1.19754E-13	8.50957E-11
119.0	1.27310E-13	8.50011E-11
120.0	1.35204E-13	8.49074E-11
121.0	1.43443E-13	8.48146E-11
122.0	1.52035E-13	8.47227E-11
123.0	1.60988E-13	8.46317E-11
124.0	1.70310E-13	8.45416E-11
125.0	1.80008E-13	8.44523E-11
126.0	1.90090E-13	8.43639E-11

127.0	2.00562E-13	8.42763E-11
128.0	2.11432E-13	8.41894E-11
129.0	2.22708E-13	8.41034E-11
130.0	2.34395E-13	8.40181E-11
131.0	2.46502E-13	8.39336E-11
132.0	2.59034E-13	8.38498E-11
133.0	2.71999E-13	8.37667E-11
134.0	2.85402E-13	8.36844E-11
135.0	2.99250E-13	8.36027E-11
136.0	3.13549E-13	8.35217E-11
137.0	3.28305E-13	8.34413E-11
138.0	3.43524E-13	8.33616E-11
139.0	3.59212E-13	8.32825E-11
140.0	3.75374E-13	8.32041E-11
141.0	3.92015E-13	8.31262E-11
142.0	4.09142E-13	8.30490E-11
143.0	4.26758E-13	8.29723E-11
144.0	4.44870E-13	8.28961E-11
145.0	4.63481E-13	8.28206E-11
146.0	4.82596E-13	8.27455E-11
147.0	5.02220E-13	8.26710E-11
148.0	5.22357E-13	8.25970E-11
149.0	5.43011E-13	8.25235E-11
150.0	5.64187E-13	8.24505E-11
151.0	5.85887E-13	8.23780E-11
152.0	6.08116E-13	8.23059E-11
153.0	6.30876E-13	8.22343E-11
154.0	6.54172E-13	8.21631E-11
155.0	6.78007E-13	8.20924E-11
156.0	7.02383E-13	8.20221E-11
157.0	7.27303E-13	8.19522E-11
158.0	7.52770E-13	8.18828E-11
159.0	7.78787E-13	8.18137E-11
160.0	8.05356E-13	8.17450E-11
161.0	8.32478E-13	8.16767E-11
162.0	8.60157E-13	8.16088E-11
163.0	8.88393E-13	8.15413E-11
164.0	9.17189E-13	8.14741E-11
165.0	9.46547E-13	8.14072E-11
166.0	9.76467E-13	8.13407E-11
167.0	1.00695E-12	8.12745E-11
168.0	1.03800E-12	8.12086E-11
169.0	1.06962E-12	8.11431E-11
170.0	1.10180E-12	8.10778E-11
171.0	1.13455E-12	8.10129E-11
172.0	1.16787E-12	8.09483E-11
173.0	1.20175E-12	8.08839E-11
174.0	1.23621E-12	8.08199E-11
175.0	1.27123E-12	8.07561E-11
176.0	1.30683E-12	8.06925E-11

177.0	1.34299E-12	8.06293E-11
178.0	1.37973E-12	8.05663E-11
179.0	1.41703E-12	8.05035E-11
180.0	1.45490E-12	8.04410E-11
181.0	1.49333E-12	8.03788E-11
182.0	1.53234E-12	8.03168E-11
183.0	1.57191E-12	8.02550E-11
184.0	1.61204E-12	8.01934E-11
185.0	1.65274E-12	8.01321E-11
186.0	1.69400E-12	8.00710E-11
187.0	1.73582E-12	8.00100E-11
188.0	1.77821E-12	7.99493E-11
189.0	1.82115E-12	7.98888E-11
190.0	1.86465E-12	7.98285E-11
191.0	1.90871E-12	7.97684E-11
192.0	1.95332E-12	7.97085E-11
193.0	1.99848E-12	7.96488E-11
194.0	2.04419E-12	7.95893E-11
195.0	2.09045E-12	7.95299E-11
196.0	2.13726E-12	7.94707E-11
197.0	2.18461E-12	7.94117E-11
198.0	2.23250E-12	7.93529E-11
199.0	2.28093E-12	7.92942E-11
200.0	2.32990E-12	7.92357E-11
201.0	2.37940E-12	7.91773E-11
202.0	2.42943E-12	7.91191E-11
203.0	2.48000E-12	7.90611E-11
204.0	2.53109E-12	7.90032E-11
205.0	2.58270E-12	7.89454E-11
206.0	2.63483E-12	7.88878E-11
207.0	2.68749E-12	7.88304E-11
208.0	2.74065E-12	7.87731E-11
209.0	2.79433E-12	7.87159E-11
210.0	2.84852E-12	7.86589E-11
211.0	2.90321E-12	7.86020E-11
212.0	2.95841E-12	7.85452E-11
213.0	3.01410E-12	7.84886E-11
214.0	3.07030E-12	7.84320E-11
215.0	3.12698E-12	7.83757E-11
216.0	3.18415E-12	7.83194E-11
217.0	3.24181E-12	7.82632E-11
218.0	3.29995E-12	7.82072E-11
219.0	3.35857E-12	7.81513E-11
220.0	3.41767E-12	7.80955E-11
221.0	3.47723E-12	7.80399E-11
222.0	3.53727E-12	7.79843E-11
223.0	3.59777E-12	7.79288E-11
224.0	3.65873E-12	7.78735E-11
225.0	3.72015E-12	7.78183E-11
226.0	3.78202E-12	7.77631E-11

227.0	3.84434E-12	7.77081E-11
228.0	3.90710E-12	7.76532E-11
229.0	3.97031E-12	7.75984E-11
230.0	4.03396E-12	7.75437E-11
231.0	4.09804E-12	7.74891E-11
232.0	4.16255E-12	7.74346E-11
233.0	4.22749E-12	7.73802E-11
234.0	4.29285E-12	7.73259E-11
235.0	4.35863E-12	7.72716E-11
236.0	4.42482E-12	7.72175E-11
237.0	4.49142E-12	7.71635E-11
238.0	4.55843E-12	7.71096E-11
239.0	4.62585E-12	7.70557E-11
240.0	4.69366E-12	7.70020E-11
241.0	4.76187E-12	7.69483E-11
242.0	4.83046E-12	7.68947E-11
243.0	4.89945E-12	7.68413E-11
244.0	4.96881E-12	7.67879E-11
245.0	5.03856E-12	7.67346E-11
246.0	5.10868E-12	7.66813E-11
247.0	5.17917E-12	7.66282E-11
248.0	5.25002E-12	7.65752E-11
249.0	5.32124E-12	7.65222E-11
250.0	5.39282E-12	7.64693E-11
251.0	5.46475E-12	7.64165E-11
252.0	5.53703E-12	7.63638E-11
253.0	5.60966E-12	7.63112E-11
254.0	5.68263E-12	7.62587E-11
255.0	5.75593E-12	7.62062E-11
256.0	5.82957E-12	7.61538E-11
257.0	5.90355E-12	7.61015E-11
258.0	5.97784E-12	7.60493E-11
259.0	6.05246E-12	7.59972E-11
260.0	6.12740E-12	7.59451E-11
261.0	6.20265E-12	7.58931E-11
262.0	6.27821E-12	7.58412E-11
263.0	6.35408E-12	7.57894E-11
264.0	6.43025E-12	7.57377E-11
265.0	6.50671E-12	7.56860E-11
266.0	6.58347E-12	7.56344E-11
267.0	6.66052E-12	7.55829E-11
268.0	6.73786E-12	7.55314E-11
269.0	6.81548E-12	7.54801E-11
270.0	6.89338E-12	7.54288E-11
271.0	6.97155E-12	7.53776E-11
272.0	7.04999E-12	7.53264E-11
273.0	7.12870E-12	7.52754E-11
274.0	7.20767E-12	7.52244E-11
275.0	7.28690E-12	7.51735E-11
276.0	7.36639E-12	7.51227E-11

277.0	7.44612E-12	7.50719E-11
278.0	7.52611E-12	7.50212E-11
279.0	7.60634E-12	7.49706E-11
280.0	7.68681E-12	7.49200E-11
281.0	7.76751E-12	7.48696E-11
282.0	7.84845E-12	7.48192E-11
283.0	7.92961E-12	7.47689E-11
284.0	8.01100E-12	7.47186E-11
285.0	8.09262E-12	7.46684E-11
286.0	8.17445E-12	7.46183E-11
287.0	8.25649E-12	7.45683E-11
288.0	8.33875E-12	7.45184E-11
289.0	8.42121E-12	7.44685E-11
290.0	8.50387E-12	7.44187E-11
291.0	8.58674E-12	7.43689E-11
292.0	8.66980E-12	7.43192E-11
293.0	8.75306E-12	7.42696E-11
294.0	8.83650E-12	7.42201E-11
295.0	8.92013E-12	7.41707E-11
296.0	9.00394E-12	7.41213E-11
297.0	9.08794E-12	7.40720E-11
298.0	9.17210E-12	7.40227E-11
299.0	9.25644E-12	7.39736E-11
300.0	9.34095E-12	7.39245E-11
301.0	9.42563E-12	7.38754E-11
302.0	9.51047E-12	7.38265E-11
303.0	9.59546E-12	7.37776E-11
304.0	9.68061E-12	7.37288E-11
305.0	9.76592E-12	7.36800E-11
306.0	9.85137E-12	7.36313E-11
307.0	9.93697E-12	7.35827E-11
308.0	1.00227E-11	7.35342E-11
309.0	1.01086E-11	7.34857E-11
310.0	1.01946E-11	7.34373E-11
311.0	1.02808E-11	7.33890E-11
312.0	1.03670E-11	7.33408E-11
313.0	1.04535E-11	7.32926E-11
314.0	1.05400E-11	7.32445E-11
315.0	1.06266E-11	7.31964E-11
316.0	1.07134E-11	7.31484E-11
317.0	1.08003E-11	7.31005E-11
318.0	1.08873E-11	7.30527E-11
319.0	1.09744E-11	7.30049E-11
320.0	1.10616E-11	7.29572E-11
321.0	1.11490E-11	7.29096E-11
322.0	1.12364E-11	7.28620E-11
323.0	1.13239E-11	7.28145E-11
324.0	1.14115E-11	7.27671E-11
325.0	1.14992E-11	7.27198E-11
326.0	1.15870E-11	7.26725E-11

327.0	1.16749E-11	7.26253E-11
328.0	1.17629E-11	7.25781E-11
329.0	1.18509E-11	7.25310E-11
330.0	1.19391E-11	7.24840E-11
331.0	1.20273E-11	7.24371E-11
332.0	1.21155E-11	7.23902E-11
333.0	1.22039E-11	7.23434E-11
334.0	1.22923E-11	7.22967E-11
335.0	1.23808E-11	7.22500E-11
336.0	1.24693E-11	7.22034E-11
337.0	1.25580E-11	7.21569E-11
338.0	1.26466E-11	7.21104E-11
339.0	1.27353E-11	7.20640E-11
340.0	1.28241E-11	7.20177E-11
341.0	1.29129E-11	7.19714E-11
342.0	1.30018E-11	7.19252E-11
343.0	1.30907E-11	7.18791E-11
344.0	1.31797E-11	7.18330E-11
345.0	1.32687E-11	7.17870E-11
346.0	1.33577E-11	7.17411E-11
347.0	1.34468E-11	7.16953E-11
348.0	1.35359E-11	7.16495E-11
349.0	1.36251E-11	7.16037E-11
350.0	1.37143E-11	7.15581E-11
351.0	1.38035E-11	7.15125E-11
352.0	1.38927E-11	7.14670E-11
353.0	1.39819E-11	7.14215E-11
354.0	1.40712E-11	7.13761E-11
355.0	1.41605E-11	7.13308E-11
356.0	1.42498E-11	7.12856E-11
357.0	1.43391E-11	7.12404E-11
358.0	1.44285E-11	7.11952E-11
359.0	1.45178E-11	7.11502E-11
360.0	1.46072E-11	7.11052E-11
361.0	1.46966E-11	7.10603E-11
362.0	1.47859E-11	7.10154E-11
363.0	1.48753E-11	7.09706E-11
364.0	1.49647E-11	7.09259E-11
365.0	1.50540E-11	7.08812E-11
366.0	1.51434E-11	7.08366E-11
367.0	1.52328E-11	7.07921E-11
368.0	1.53221E-11	7.07476E-11
369.0	1.54115E-11	7.07032E-11
370.0	1.55008E-11	7.06589E-11
371.0	1.55901E-11	7.06146E-11
372.0	1.56794E-11	7.05704E-11
373.0	1.57687E-11	7.05262E-11
374.0	1.58580E-11	7.04822E-11
375.0	1.59472E-11	7.04381E-11
376.0	1.60365E-11	7.03942E-11

377.0	1.61257E-11	7.03503E-11
378.0	1.62149E-11	7.03065E-11
379.0	1.63040E-11	7.02627E-11
380.0	1.63932E-11	7.02190E-11
381.0	1.64823E-11	7.01754E-11
382.0	1.65713E-11	7.01318E-11
383.0	1.66604E-11	7.00883E-11
384.0	1.67494E-11	7.00449E-11
385.0	1.68383E-11	7.00015E-11
386.0	1.69273E-11	6.99582E-11
387.0	1.70162E-11	6.99149E-11
388.0	1.71050E-11	6.98717E-11
389.0	1.71938E-11	6.98286E-11
390.0	1.72826E-11	6.97855E-11
391.0	1.73713E-11	6.97425E-11
392.0	1.74600E-11	6.96996E-11
393.0	1.75486E-11	6.96567E-11
394.0	1.76372E-11	6.96139E-11
395.0	1.77257E-11	6.95711E-11
396.0	1.78142E-11	6.95284E-11
397.0	1.79026E-11	6.94858E-11
398.0	1.79909E-11	6.94432E-11
399.0	1.80792E-11	6.94007E-11
400.0	1.81675E-11	6.93583E-11
401.0	1.82557E-11	6.93159E-11
402.0	1.83438E-11	6.92735E-11
403.0	1.84319E-11	6.92313E-11
404.0	1.85199E-11	6.91891E-11
405.0	1.86078E-11	6.91469E-11
406.0	1.86957E-11	6.91048E-11
407.0	1.87835E-11	6.90628E-11
408.0	1.88712E-11	6.90208E-11
409.0	1.89589E-11	6.89789E-11
410.0	1.90465E-11	6.89371E-11
411.0	1.91340E-11	6.88953E-11
412.0	1.92215E-11	6.88535E-11
413.0	1.93088E-11	6.88119E-11
414.0	1.93961E-11	6.87702E-11
415.0	1.94834E-11	6.87287E-11
416.0	1.95705E-11	6.86872E-11
417.0	1.96576E-11	6.86458E-11
418.0	1.97446E-11	6.86044E-11
419.0	1.98315E-11	6.85630E-11
420.0	1.99183E-11	6.85218E-11
421.0	2.00051E-11	6.84806E-11
422.0	2.00917E-11	6.84394E-11
423.0	2.01783E-11	6.83983E-11
424.0	2.02648E-11	6.83573E-11
425.0	2.03512E-11	6.83163E-11
426.0	2.04375E-11	6.82754E-11

427.0	2.05238E-11	6.82345E-11
428.0	2.06099E-11	6.81937E-11
429.0	2.06960E-11	6.81530E-11
430.0	2.07819E-11	6.81123E-11
431.0	2.08678E-11	6.80716E-11
432.0	2.09536E-11	6.80311E-11
433.0	2.10392E-11	6.79905E-11
434.0	2.11248E-11	6.79501E-11
435.0	2.12103E-11	6.79096E-11
436.0	2.12957E-11	6.78693E-11
437.0	2.13810E-11	6.78290E-11
438.0	2.14662E-11	6.77887E-11
439.0	2.15513E-11	6.77485E-11
440.0	2.16363E-11	6.77084E-11
441.0	2.17212E-11	6.76683E-11
442.0	2.18060E-11	6.76283E-11
443.0	2.18907E-11	6.75883E-11
444.0	2.19752E-11	6.75484E-11
445.0	2.20597E-11	6.75085E-11
446.0	2.21441E-11	6.74687E-11
447.0	2.22284E-11	6.74289E-11
448.0	2.23125E-11	6.73892E-11
449.0	2.23966E-11	6.73495E-11
450.0	2.24805E-11	6.73099E-11
451.0	2.25644E-11	6.72704E-11
452.0	2.26481E-11	6.72309E-11
453.0	2.27317E-11	6.71914E-11
454.0	2.28152E-11	6.71520E-11
455.0	2.28986E-11	6.71127E-11
456.0	2.29819E-11	6.70734E-11
457.0	2.30651E-11	6.70342E-11
458.0	2.31482E-11	6.69950E-11
459.0	2.32311E-11	6.69558E-11
460.0	2.33139E-11	6.69168E-11
461.0	2.33967E-11	6.68777E-11
462.0	2.34793E-11	6.68387E-11
463.0	2.35617E-11	6.67998E-11
464.0	2.36441E-11	6.67609E-11
465.0	2.37264E-11	6.67221E-11
466.0	2.38085E-11	6.66833E-11
467.0	2.38905E-11	6.66446E-11
468.0	2.39724E-11	6.66059E-11
469.0	2.40542E-11	6.65673E-11
470.0	2.41358E-11	6.65287E-11
471.0	2.42173E-11	6.64901E-11
472.0	2.42988E-11	6.64517E-11
473.0	2.43800E-11	6.64132E-11
474.0	2.44612E-11	6.63748E-11
475.0	2.45423E-11	6.63365E-11
476.0	2.46232E-11	6.62982E-11

477.0	2.47040E-11	6.62600E-11
478.0	2.47846E-11	6.62218E-11
479.0	2.48652E-11	6.61836E-11
480.0	2.49456E-11	6.61456E-11
481.0	2.50259E-11	6.61075E-11
482.0	2.51061E-11	6.60695E-11
483.0	2.51861E-11	6.60316E-11
484.0	2.52660E-11	6.59936E-11
485.0	2.53458E-11	6.59558E-11
486.0	2.54254E-11	6.59180E-11
487.0	2.55050E-11	6.58802E-11
488.0	2.55844E-11	6.58425E-11
489.0	2.56636E-11	6.58048E-11
490.0	2.57428E-11	6.57672E-11
491.0	2.58218E-11	6.57296E-11
492.0	2.59007E-11	6.56921E-11
493.0	2.59794E-11	6.56546E-11
494.0	2.60580E-11	6.56172E-11
495.0	2.61365E-11	6.55798E-11
496.0	2.62148E-11	6.55424E-11
497.0	2.62931E-11	6.55051E-11
498.0	2.63711E-11	6.54678E-11
499.0	2.64491E-11	6.54306E-11
500.0	2.65269E-11	6.53935E-11

Table S11: Computed excitation and de-excitation cross sections in Å^2 units for a series of inelastic processes generated using the 2D RR-PES for the $HeH^+(j_1)\cdots H_2$ system with $\Delta j_1 = +1$, $\Delta j_1 = +2$, $\Delta j_1 = -1$ and $\Delta j_1 = -2$.

#Tot_ener(cm-1)	0--->1 Cross_sec(angs^2)
6.71457584E+01	2.56336088E-01
6.71467584E+01	3.47599768E-01
6.71477584E+01	4.12299486E-01
6.71487584E+01	4.63618679E-01
6.71497584E+01	5.06643354E-01
6.71507584E+01	5.43991086E-01
6.71517584E+01	5.77197807E-01
6.71527584E+01	6.07386572E-01
6.71537584E+01	6.34980639E-01
6.71547584E+01	6.60635243E-01
6.71647584E+01	8.68091776E-01
6.71747584E+01	1.06605742E+00
6.71847584E+01	1.30305359E+00
6.71947584E+01	1.59578860E+00
6.72047584E+01	1.94114888E+00
6.72147584E+01	2.32020410E+00
6.72247584E+01	2.70535111E+00
6.72347584E+01	3.06949593E+00
6.72447584E+01	3.39310831E+00
6.73447584E+01	4.62129800E+00

6.74447584E+01	5.14442927E+00
6.75447584E+01	5.90969381E+00
6.76447584E+01	6.85585432E+00
6.77447584E+01	7.96549076E+00
6.78447584E+01	9.12038309E+00
6.79447584E+01	1.01825370E+01
6.80447584E+01	1.11021038E+01
7.31447584E+01	2.22140519E+01
7.41447584E+01	2.39750024E+01
7.51447584E+01	2.60334584E+01
7.61447584E+01	2.90811561E+01
7.71447584E+01	3.71758857E+01
8.71447584E+01	3.16413091E+01
9.71447584E+01	3.12178836E+01
1.07144758E+02	4.05381751E+01
1.17144758E+02	3.73789371E+01
1.27144758E+02	2.85739694E+01
1.37144758E+02	2.60322746E+01
1.47144758E+02	4.14317085E+01
1.57144758E+02	3.44220634E+01
1.67144758E+02	3.34864000E+01
2.01435275E+02	3.76900184E+01
2.01436275E+02	3.76998298E+01
2.01437275E+02	3.77102064E+01
2.01438275E+02	3.77207147E+01
2.01439275E+02	3.77312027E+01
2.01440275E+02	3.77416327E+01
2.01441275E+02	3.77519780E+01
2.01442275E+02	3.77622221E+01
2.01443275E+02	3.77723611E+01
2.01444275E+02	3.77823967E+01
2.01454275E+02	3.78786812E+01
2.01464275E+02	3.79703120E+01
2.01474275E+02	3.80589217E+01
2.01484275E+02	3.81444075E+01
2.01494275E+02	3.82261634E+01
2.01504275E+02	3.83037992E+01
2.01514275E+02	3.83773658E+01
2.01524275E+02	3.84471551E+01
2.01534275E+02	3.85137224E+01
2.01634275E+02	3.90778028E+01
2.01734275E+02	3.90667204E+01
2.01834275E+02	3.81929951E+01
2.01934275E+02	3.78130189E+01
2.02034275E+02	3.76593356E+01
2.02134275E+02	3.76211426E+01
2.02234275E+02	3.75662961E+01
2.02334275E+02	3.74494070E+01
2.07434275E+02	2.81132901E+01
2.08434275E+02	2.75105495E+01

2.09434275E+02 2.82462160E+01
2.10434275E+02 2.66342001E+01
2.11434275E+02 2.69344389E+01
2.21434275E+02 3.12035692E+01
2.31434275E+02 2.20112851E+01
2.41434275E+02 2.16156648E+01
2.51434275E+02 2.55732376E+01
2.61434275E+02 2.40044899E+01
2.67144758E+02 2.32733450E+01
2.71434275E+02 2.19597412E+01
2.81434275E+02 2.17347280E+01
2.91434275E+02 2.36805591E+01
3.01434275E+02 2.40466960E+01
3.67144758E+02 3.13147707E+01
4.01434275E+02 2.72418470E+01
4.02869550E+02 2.73064370E+01
4.02870550E+02 2.73019877E+01
4.02871550E+02 2.72990599E+01
4.02872550E+02 2.72968375E+01
4.02873550E+02 2.72949933E+01
4.02874550E+02 2.72934000E+01
4.02875550E+02 2.72919449E+01
4.02876550E+02 2.72906084E+01
4.02877550E+02 2.72893337E+01
4.02878550E+02 2.72880880E+01
4.02888550E+02 2.72756305E+01
4.02898550E+02 2.72602493E+01
4.02908550E+02 2.72418446E+01
4.02918550E+02 2.72219523E+01
4.02928550E+02 2.72024357E+01
4.02938550E+02 2.71849217E+01
4.02948550E+02 2.71703410E+01
4.02958550E+02 2.71590273E+01
4.02968550E+02 2.71508062E+01
4.03068550E+02 2.71000553E+01
4.03168550E+02 2.69118453E+01
4.03268550E+02 2.68843203E+01
4.03368550E+02 2.69405148E+01
4.03468550E+02 2.71440328E+01
4.03568550E+02 2.72601605E+01
4.03668550E+02 2.72987437E+01
4.03768550E+02 2.73429491E+01
4.08868550E+02 2.95243667E+01
4.09868550E+02 2.96251442E+01
4.10868550E+02 2.89835568E+01
4.11868550E+02 2.84638823E+01
4.12868550E+02 2.87285385E+01
4.22868550E+02 2.67111721E+01
4.32868550E+02 2.54987763E+01
4.42868550E+02 2.67021990E+01

4.52868550E+02 2.40363297E+01
4.62868550E+02 2.26743682E+01
4.67144758E+02 2.30640720E+01
4.72868550E+02 2.50680838E+01
4.82868550E+02 2.61572980E+01
4.92868550E+02 2.64770224E+01
5.01434275E+02 2.60183128E+01
5.02868550E+02 2.63438716E+01
5.67144758E+02 2.68200169E+01
6.01434275E+02 2.74839397E+01
6.02868550E+02 2.71240893E+01
6.67144758E+02 2.66000248E+01
6.71448584E+02 2.73695784E+01
6.71449584E+02 2.73689514E+01
6.71450584E+02 2.73682812E+01
6.71451584E+02 2.73676262E+01
6.71452584E+02 2.73669790E+01
6.71453584E+02 2.73663412E+01
6.71454584E+02 2.73657147E+01
6.71455584E+02 2.73651071E+01
6.71456584E+02 2.73645207E+01
6.71457584E+02 2.73639285E+01
6.71467584E+02 2.73579494E+01
6.71477584E+02 2.73510660E+01
6.71487584E+02 2.73427834E+01
6.71497584E+02 2.73331628E+01
6.71507584E+02 2.73224069E+01
6.71517584E+02 2.73107467E+01
6.71527584E+02 2.72983775E+01
6.71537584E+02 2.72854203E+01
6.71547584E+02 2.72720051E+01
6.71647584E+02 2.71274253E+01
6.71747584E+02 2.69855354E+01
6.71847584E+02 2.68437389E+01
6.71947584E+02 2.66870300E+01
6.72047584E+02 2.65157802E+01
6.72147584E+02 2.63352671E+01
6.72247584E+02 2.61534430E+01
6.72347584E+02 2.59814103E+01
6.77447584E+02 2.66818586E+01
6.78447584E+02 2.75014421E+01
6.79447584E+02 2.71216842E+01
6.80447584E+02 2.62082323E+01
6.81447584E+02 2.60329285E+01
6.91447584E+02 2.61785633E+01
7.01434275E+02 2.41440836E+01
7.01447584E+02 2.41418909E+01
7.02868550E+02 2.38570688E+01
7.11447584E+02 2.33404440E+01
7.21447584E+02 2.57368245E+01

7.31447584E+02 2.75250912E+01
7.41447584E+02 2.70586757E+01
7.51447584E+02 2.45814449E+01
7.61447584E+02 2.53825939E+01
7.67144758E+02 2.49344619E+01
7.71447584E+02 2.51691265E+01
8.01434275E+02 2.70766456E+01
8.02868550E+02 2.64278799E+01
8.67144758E+02 2.56682449E+01
8.71447584E+02 2.65190614E+01
9.01434275E+02 2.92441710E+01
9.02868550E+02 2.96421918E+01
9.67144758E+02 2.81789499E+01
9.71447584E+02 2.85842495E+01
1.00143428E+03 2.77160257E+01
1.00286855E+03 2.74825239E+01
1.00717238E+03 2.73043615E+01
1.00717338E+03 2.73054952E+01
1.00717438E+03 2.73065984E+01
1.00717538E+03 2.73076878E+01
1.00717638E+03 2.73087761E+01
1.00717738E+03 2.73098621E+01
1.00717838E+03 2.73109471E+01
1.00717938E+03 2.73120305E+01
1.00718038E+03 2.73131132E+01
1.00718138E+03 2.73141922E+01
1.00719138E+03 2.73247427E+01
1.00720138E+03 2.73346281E+01
1.00721138E+03 2.73437926E+01
1.00722138E+03 2.73523271E+01
1.00723138E+03 2.73602989E+01
1.00724138E+03 2.73677885E+01
1.00725138E+03 2.73748544E+01
1.00726138E+03 2.73815662E+01
1.00727138E+03 2.73879893E+01
1.00737138E+03 2.74455279E+01
1.00747138E+03 2.75014493E+01
1.00757138E+03 2.75615080E+01
1.00767138E+03 2.76233447E+01
1.00777138E+03 2.76798513E+01
1.00787138E+03 2.77285953E+01
1.00797138E+03 2.77712324E+01
1.00807138E+03 2.78112469E+01
1.01317138E+03 2.92319288E+01
1.01417138E+03 2.92232634E+01
1.01517138E+03 2.91900371E+01
1.01617138E+03 2.91108441E+01
1.01717138E+03 2.90118464E+01
1.02717138E+03 2.82177847E+01
1.03717138E+03 2.71461795E+01

1.04717138E+03 2.61523173E+01
1.05717138E+03 2.55439006E+01
1.06714476E+03 2.59759094E+01
1.06717138E+03 2.59913086E+01
1.07144758E+03 2.55494721E+01
1.07717138E+03 2.62381837E+01
1.08717138E+03 2.64965759E+01
1.09717138E+03 2.59371742E+01
1.10143428E+03 2.60210195E+01
1.10286855E+03 2.59600514E+01
1.10717138E+03 2.57546626E+01
1.16714476E+03 2.44700204E+01
1.17144758E+03 2.39036851E+01
1.20143428E+03 2.19005554E+01
1.20286855E+03 2.19841671E+01
1.20717138E+03 2.19486848E+01
1.26714476E+03 2.27256829E+01
1.27144758E+03 2.14771570E+01
1.30143428E+03 2.29595997E+01
1.30286855E+03 2.27901202E+01
1.30717138E+03 2.31655114E+01
1.36714476E+03 2.09580746E+01
1.37144758E+03 2.10404749E+01
1.40143428E+03 2.06348383E+01
1.40286855E+03 2.06304831E+01
1.40717138E+03 2.03110344E+01
1.46714476E+03 1.79277921E+01
1.47144758E+03 1.73342291E+01
1.50143428E+03 1.86049374E+01
1.50286855E+03 1.86702307E+01
1.50717138E+03 1.87740029E+01
1.56714476E+03 1.79596550E+01
1.57144758E+03 1.79542062E+01
1.60143428E+03 1.76626694E+01
1.60286855E+03 1.76612949E+01
1.60717138E+03 1.76890350E+01
1.66714476E+03 1.74291139E+01
1.67144758E+03 1.71696630E+01
1.70143428E+03 1.78728593E+01
1.70286855E+03 1.78107585E+01
1.70717138E+03 1.77928560E+01
1.76714476E+03 1.83182971E+01
1.77144758E+03 1.83098673E+01
1.80143428E+03 1.86849720E+01
1.80286855E+03 1.87992430E+01
1.80717138E+03 1.79463732E+01
1.86714476E+03 1.87670913E+01
1.87144758E+03 1.88268268E+01
1.90143428E+03 1.89405783E+01
1.90286855E+03 1.89485101E+01

1.90717138E+03 1.89650984E+01
1.96714476E+03 1.80421648E+01
1.97144758E+03 1.78478454E+01
2.00143428E+03 1.89803211E+01
2.00286855E+03 1.90467575E+01
2.00717138E+03 1.91668625E+01
2.06714476E+03 1.92186521E+01
2.07144758E+03 1.91287124E+01
2.10143428E+03 1.90426385E+01
2.10286855E+03 1.90101710E+01
2.10717138E+03 1.89196504E+01
2.16714476E+03 1.91190378E+01
2.17144758E+03 1.92456840E+01
2.20143428E+03 1.94160171E+01
2.20286855E+03 1.90311814E+01
2.20717138E+03 1.94984040E+01
2.26714476E+03 1.88908188E+01
2.27144758E+03 1.91798393E+01
2.30143428E+03 1.95150223E+01
2.30286855E+03 1.95169528E+01
2.30717138E+03 1.95260413E+01
2.36714476E+03 1.91238057E+01
2.37144758E+03 1.90734639E+01
2.40143428E+03 1.92965258E+01
2.40286855E+03 1.92657469E+01
2.40717138E+03 1.91664191E+01
2.46714476E+03 1.99589992E+01
2.47144758E+03 1.97595307E+01
2.50143428E+03 2.00285177E+01
2.50286855E+03 2.00159157E+01
2.50717138E+03 1.99690337E+01
2.56714476E+03 1.95354178E+01
2.57144758E+03 1.95024329E+01
2.60143428E+03 1.90911043E+01
2.60286855E+03 1.90811329E+01
2.60717138E+03 1.90395350E+01
2.66714476E+03 1.99737231E+01
2.67144758E+03 1.99353051E+01
2.70143428E+03 1.94710014E+01
2.70286855E+03 1.94386835E+01
2.70717138E+03 1.93233003E+01
2.76714476E+03 1.86964521E+01
2.77144758E+03 1.89091145E+01
2.80143428E+03 1.85383680E+01
2.80286855E+03 1.84969440E+01
2.80717138E+03 1.85560432E+01
2.86714476E+03 1.92322716E+01
2.87144758E+03 1.92006461E+01
2.90143428E+03 1.88845995E+01
2.90286855E+03 1.88651611E+01

2.90717138E+03 1.88031029E+01
2.96714476E+03 1.83339568E+01
2.97144758E+03 1.83598416E+01
3.00143428E+03 1.85055202E+01
3.00286855E+03 1.84798012E+01
3.00717138E+03 1.84005269E+01
3.07144758E+03 1.82512652E+01
3.10143428E+03 1.79620996E+01
3.10286855E+03 1.79452353E+01
3.10717138E+03 1.78897737E+01
3.17144758E+03 1.74878397E+01
3.20286855E+03 1.81107165E+01
3.20717138E+03 1.80526262E+01
3.27144758E+03 1.74354926E+01
3.30286855E+03 1.70795109E+01
3.30717138E+03 1.70444196E+01
3.37144758E+03 1.70283391E+01
3.40717138E+03 1.68507188E+01
3.47144758E+03 1.68136117E+01
3.50717138E+03 1.64338341E+01
3.57144758E+03 1.61224645E+01
3.60717138E+03 1.64965495E+01
3.70717138E+03 1.59386429E+01
3.80717138E+03 1.56116438E+01
3.90717138E+03 1.56438022E+01

#Tot_ener(cm-1)   0 -->2 Cross_sec(angs^2)
2.01435275E+02 1.15274239E-01
2.01436275E+02 1.53834868E-01
2.01437275E+02 1.81065729E-01
2.01438275E+02 2.02933948E-01
2.01439275E+02 2.21656334E-01
2.01440275E+02 2.38329822E-01
2.01441275E+02 2.53563909E-01
2.01442275E+02 2.67742179E-01
2.01443275E+02 2.81111495E-01
2.01444275E+02 2.93813340E-01
2.01454275E+02 4.02432530E-01
2.01464275E+02 4.95112152E-01
2.01474275E+02 5.80592341E-01
2.01484275E+02 6.62479750E-01
2.01494275E+02 7.42595657E-01
2.01504275E+02 8.21539049E-01
2.01514275E+02 8.99170823E-01
2.01524275E+02 9.75073096E-01
2.01534275E+02 1.04877306E+00
2.01634275E+02 1.74383028E+00
2.01734275E+02 2.68392664E+00
2.01834275E+02 3.07304431E+00
2.01934275E+02 3.30619841E+00

2.02034275E+02	3.50717449E+00
2.02134275E+02	3.66923126E+00
2.02234275E+02	3.86411044E+00
2.02334275E+02	4.08729411E+00
2.07434275E+02	9.69212832E+00
2.08434275E+02	9.67566761E+00
2.09434275E+02	9.65743704E+00
2.10434275E+02	1.02891116E+01
2.11434275E+02	1.06309313E+01
2.21434275E+02	1.41004146E+01
2.31434275E+02	1.47485858E+01
2.41434275E+02	1.72469933E+01
2.51434275E+02	1.83219275E+01
2.61434275E+02	1.81117056E+01
2.67144758E+02	2.01377640E+01
2.71434275E+02	2.05702679E+01
2.81434275E+02	1.79526515E+01
2.91434275E+02	1.70227793E+01
3.01434275E+02	1.91377393E+01
3.67144758E+02	1.89302880E+01
4.01434275E+02	1.92676747E+01
4.02869550E+02	1.98204866E+01
4.02870550E+02	1.98236641E+01
4.02871550E+02	1.98266752E+01
4.02872550E+02	1.98295862E+01
4.02873550E+02	1.98323801E+01
4.02874550E+02	1.98350852E+01
4.02875550E+02	1.98377055E+01
4.02876550E+02	1.98402460E+01
4.02877550E+02	1.98427072E+01
4.02878550E+02	1.98451042E+01
4.02888550E+02	1.98660487E+01
4.02898550E+02	1.98826310E+01
4.02908550E+02	1.98950331E+01
4.02918550E+02	1.99027026E+01
4.02928550E+02	1.99053698E+01
4.02938550E+02	1.99033608E+01
4.02948550E+02	1.98975282E+01
4.02958550E+02	1.98889450E+01
4.02968550E+02	1.98786385E+01
4.03068550E+02	1.97407042E+01
4.03168550E+02	1.95556762E+01
4.03268550E+02	1.93965569E+01
4.03368550E+02	1.92340338E+01
4.03468550E+02	1.92396959E+01
4.03568550E+02	1.92270282E+01
4.03668550E+02	1.91571293E+01
4.03768550E+02	1.90640151E+01
4.08868550E+02	1.63035774E+01
4.09868550E+02	1.64867578E+01

4.10868550E+02 1.72061063E+01
4.11868550E+02 1.77811851E+01
4.12868550E+02 1.77144675E+01
4.22868550E+02 1.87252704E+01
4.32868550E+02 1.73656292E+01
4.42868550E+02 1.86640711E+01
4.52868550E+02 1.63429042E+01
4.62868550E+02 1.67696503E+01
4.67144758E+02 1.69769783E+01
4.72868550E+02 1.78393340E+01
4.82868550E+02 1.73931247E+01
4.92868550E+02 1.75133809E+01
5.01434275E+02 1.90430336E+01
5.02868550E+02 1.90096263E+01
5.67144758E+02 1.89973059E+01
6.01434275E+02 1.76391038E+01
6.02868550E+02 1.75242305E+01
6.67144758E+02 1.96295884E+01
6.71448584E+02 1.79372790E+01
6.71449584E+02 1.79378346E+01
6.71450584E+02 1.79381719E+01
6.71451584E+02 1.79383204E+01
6.71452584E+02 1.79383005E+01
6.71453584E+02 1.79381301E+01
6.71454584E+02 1.79378199E+01
6.71455584E+02 1.79373831E+01
6.71456584E+02 1.79368254E+01
6.71457584E+02 1.79361576E+01
6.71467584E+02 1.79251928E+01
6.71477584E+02 1.79107550E+01
6.71487584E+02 1.78967071E+01
6.71497584E+02 1.78845618E+01
6.71507584E+02 1.78745481E+01
6.71517584E+02 1.78664308E+01
6.71527584E+02 1.78598587E+01
6.71537584E+02 1.78544885E+01
6.71547584E+02 1.78500455E+01
6.71647584E+02 1.78263668E+01
6.71747584E+02 1.78093738E+01
6.71847584E+02 1.77949789E+01
6.71947584E+02 1.77791997E+01
6.72047584E+02 1.77506049E+01
6.72147584E+02 1.77007716E+01
6.72247584E+02 1.76396609E+01
6.72347584E+02 1.75849357E+01
6.77447584E+02 1.63404644E+01
6.78447584E+02 1.61039211E+01
6.79447584E+02 1.61313452E+01
6.80447584E+02 1.63919555E+01
6.81447584E+02 1.65500510E+01

6.91447584E+02	1.71400936E+01
7.01434275E+02	1.66947718E+01
7.01447584E+02	1.66937307E+01
7.02868550E+02	1.66754392E+01
7.11447584E+02	1.58583166E+01
7.21447584E+02	1.48105597E+01
7.31447584E+02	1.55888925E+01
7.41447584E+02	1.59115837E+01
7.51447584E+02	1.57062398E+01
7.61447584E+02	1.57085418E+01
7.67144758E+02	1.57011413E+01
7.71447584E+02	1.59559389E+01
8.01434275E+02	1.42488967E+01
8.02868550E+02	1.43533284E+01
8.67144758E+02	1.48271591E+01
8.71447584E+02	1.43100225E+01
9.01434275E+02	1.40617348E+01
9.02868550E+02	1.37956946E+01
9.67144758E+02	1.36059770E+01
9.71447584E+02	1.39086285E+01
1.00143428E+03	1.32916165E+01
1.00286855E+03	1.33642938E+01
1.00717238E+03	1.30568769E+01
1.00717338E+03	1.30563701E+01
1.00717438E+03	1.30559420E+01
1.00717538E+03	1.30555517E+01
1.00717638E+03	1.30551926E+01
1.00717738E+03	1.30548582E+01
1.00717838E+03	1.30545461E+01
1.00717938E+03	1.30542515E+01
1.00718038E+03	1.30539719E+01
1.00718138E+03	1.30537100E+01
1.00719138E+03	1.30517897E+01
1.00720138E+03	1.30507711E+01
1.00721138E+03	1.30502975E+01
1.00722138E+03	1.30501634E+01
1.00723138E+03	1.30502426E+01
1.00724138E+03	1.30504658E+01
1.00725138E+03	1.30507878E+01
1.00726138E+03	1.30511755E+01
1.00727138E+03	1.30516100E+01
1.00737138E+03	1.30566725E+01
1.00747138E+03	1.30619453E+01
1.00757138E+03	1.30708854E+01
1.00767138E+03	1.30864852E+01
1.00777138E+03	1.31058456E+01
1.00787138E+03	1.31258991E+01
1.00797138E+03	1.31456850E+01
1.00807138E+03	1.31656327E+01
1.01317138E+03	1.36360613E+01

1.01417138E+03 1.36708164E+01
1.01517138E+03 1.37160785E+01
1.01617138E+03 1.37830358E+01
1.01717138E+03 1.38572646E+01
1.02717138E+03 1.40482079E+01
1.03717138E+03 1.27694358E+01
1.04717138E+03 1.26443486E+01
1.05717138E+03 1.26489817E+01
1.06714476E+03 1.20227059E+01
1.06717138E+03 1.19992681E+01
1.07144758E+03 1.19596890E+01
1.07717138E+03 1.19658405E+01
1.08717138E+03 1.25665538E+01
1.09717138E+03 1.20531668E+01
1.10143428E+03 1.24857980E+01
1.10286855E+03 1.25462976E+01
1.10717138E+03 1.26781448E+01
1.16714476E+03 1.11182245E+01
1.17144758E+03 1.10858122E+01
1.20143428E+03 1.03130438E+01
1.20286855E+03 1.03474653E+01
1.20717138E+03 1.06499016E+01
1.26714476E+03 1.06906781E+01
1.27144758E+03 1.04050924E+01
1.30143428E+03 1.08383178E+01
1.30286855E+03 1.06696505E+01
1.30717138E+03 1.07985246E+01
1.36714476E+03 1.06750024E+01
1.37144758E+03 1.07967222E+01
1.40143428E+03 1.17003732E+01
1.40286855E+03 1.18074376E+01
1.40717138E+03 1.19145174E+01
1.46714476E+03 1.16017422E+01
1.47144758E+03 1.14610576E+01
1.50143428E+03 1.12720781E+01
1.50286855E+03 1.13125212E+01
1.50717138E+03 1.14571157E+01
1.56714476E+03 1.23938617E+01
1.57144758E+03 1.23405526E+01
1.60143428E+03 1.12540607E+01
1.60286855E+03 1.12398689E+01
1.60717138E+03 1.12254663E+01
1.66714476E+03 1.13880591E+01
1.67144758E+03 1.13639379E+01
1.70143428E+03 1.02159573E+01
1.70286855E+03 1.08152305E+01
1.70717138E+03 1.07644202E+01
1.76714476E+03 1.06956627E+01
1.77144758E+03 1.08206701E+01
1.80143428E+03 1.01697928E+01

1.80286855E+03	1.03616416E+01
1.80717138E+03	1.09281285E+01
1.86714476E+03	1.00567865E+01
1.87144758E+03	1.00352134E+01
1.90143428E+03	1.03306039E+01
1.90286855E+03	1.03539632E+01
1.90717138E+03	1.04055859E+01
1.96714476E+03	1.00140480E+01
1.97144758E+03	9.85014732E+00
2.00143428E+03	9.44159221E+00
2.00286855E+03	9.53818548E+00
2.00717138E+03	9.76631372E+00
2.06714476E+03	1.02151534E+01
2.07144758E+03	1.02802860E+01
2.10143428E+03	1.01601070E+01
2.10286855E+03	1.01686729E+01
2.10717138E+03	1.01668243E+01
2.16714476E+03	9.43696790E+00
2.17144758E+03	9.44132147E+00
2.20143428E+03	9.64545251E+00
2.20286855E+03	9.53300462E+00
2.20717138E+03	9.57578225E+00
2.26714476E+03	9.08157106E+00
2.27144758E+03	8.91668352E+00
2.30143428E+03	8.89352721E+00
2.30286855E+03	8.89083073E+00
2.30717138E+03	8.88851358E+00
2.36714476E+03	8.88249932E+00
2.37144758E+03	8.59671171E+00
2.40143428E+03	8.94879282E+00
2.40286855E+03	8.95841881E+00
2.40717138E+03	8.96226018E+00
2.46714476E+03	8.54976250E+00
2.47144758E+03	8.66719824E+00
2.50143428E+03	8.41192903E+00
2.50286855E+03	8.41796000E+00
2.50717138E+03	8.43005779E+00
2.56714476E+03	8.62619982E+00
2.57144758E+03	8.68219016E+00
2.60143428E+03	8.21648918E+00
2.60286855E+03	8.21563659E+00
2.60717138E+03	8.20280054E+00
2.66714476E+03	8.28452112E+00
2.67144758E+03	8.30297649E+00
2.70143428E+03	8.41952313E+00
2.70286855E+03	8.42733068E+00
2.70717138E+03	8.45846359E+00
2.76714476E+03	8.37624449E+00
2.77144758E+03	8.14756874E+00
2.80143428E+03	8.00093424E+00

2.80286855E+03 7.90422566E+00
2.80717138E+03 7.78635671E+00
2.86714476E+03 8.18051995E+00
2.87144758E+03 8.13742030E+00
2.90143428E+03 8.23518756E+00
2.90286855E+03 8.23811611E+00
2.90717138E+03 8.24708309E+00
2.96714476E+03 7.78926025E+00
2.97144758E+03 7.71298507E+00
3.00143428E+03 7.70006102E+00
3.00286855E+03 7.70249561E+00
3.00717138E+03 7.69361623E+00
3.07144758E+03 8.00317332E+00
3.10143428E+03 7.95660793E+00
3.10286855E+03 7.94998465E+00
3.10717138E+03 7.92611170E+00
3.17144758E+03 7.29467841E+00
3.20286855E+03 7.29398321E+00
3.20717138E+03 7.31243001E+00
3.27144758E+03 7.70050988E+00
3.30286855E+03 7.60853305E+00
3.30717138E+03 7.60690241E+00
3.37144758E+03 7.15806550E+00
3.40717138E+03 7.06877767E+00
3.47144758E+03 7.47345654E+00
3.50717138E+03 7.38600392E+00
3.57144758E+03 7.20286877E+00
3.60717138E+03 7.00034018E+00
3.70717138E+03 7.36298724E+00
3.80717138E+03 7.03439823E+00
3.90717138E+03 7.27702288E+00

#Tot_ener(cm-1) 1--->2 Cross_sec(angs^2)
2.01435275E+02 6.20814075E-02
2.01436275E+02 8.32447153E-02
2.01437275E+02 9.84764204E-02
2.01438275E+02 1.10951088E-01
2.01439275E+02 1.21843337E-01
2.01440275E+02 1.31719670E-01
2.01441275E+02 1.40908691E-01
2.01442275E+02 1.49604798E-01
2.01443275E+02 1.57933757E-01
2.01444275E+02 1.65984895E-01
2.01454275E+02 2.39150660E-01
2.01464275E+02 3.06680198E-01
2.01474275E+02 3.71287103E-01
2.01484275E+02 4.33508566E-01
2.01494275E+02 4.93406614E-01
2.01504275E+02 5.50865229E-01
2.01514275E+02 6.05783973E-01

2.01524275E+02	6.58158835E-01
2.01534275E+02	7.08184521E-01
2.01634275E+02	1.26795801E+00
2.01734275E+02	2.41449637E+00
2.01834275E+02	3.00303866E+00
2.01934275E+02	2.97497028E+00
2.02034275E+02	2.86992695E+00
2.02134275E+02	2.84791875E+00
2.02234275E+02	2.88045023E+00
2.02334275E+02	2.95972749E+00
2.07434275E+02	8.93366625E+00
2.08434275E+02	9.54729317E+00
2.09434275E+02	9.98668360E+00
2.10434275E+02	1.05072662E+01
2.11434275E+02	1.10394815E+01
2.21434275E+02	1.76033159E+01
2.31434275E+02	2.05504047E+01
2.41434275E+02	2.14953577E+01
2.51434275E+02	2.20076888E+01
2.61434275E+02	2.52646257E+01
2.67144758E+02	2.52398843E+01
2.71434275E+02	2.44085694E+01
2.81434275E+02	2.25263628E+01
2.91434275E+02	2.57997388E+01
3.01434275E+02	2.57456143E+01
3.67144758E+02	2.62955424E+01
4.01434275E+02	2.92516569E+01
4.02869550E+02	2.84141625E+01
4.02870550E+02	2.84042698E+01
4.02871550E+02	2.83970449E+01
4.02872550E+02	2.83910636E+01
4.02873550E+02	2.83857949E+01
4.02874550E+02	2.83809662E+01
4.02875550E+02	2.83764304E+01
4.02876550E+02	2.83720876E+01
4.02877550E+02	2.83678873E+01
4.02878550E+02	2.83637672E+01
4.02888550E+02	2.83234463E+01
4.02898550E+02	2.82809116E+01
4.02908550E+02	2.82358790E+01
4.02918550E+02	2.81895402E+01
4.02928550E+02	2.81432865E+01
4.02938550E+02	2.80982267E+01
4.02948550E+02	2.80550754E+01
4.02958550E+02	2.80141403E+01
4.02968550E+02	2.79754383E+01
4.03068550E+02	2.76528562E+01
4.03168550E+02	2.74045419E+01
4.03268550E+02	2.71142000E+01
4.03368550E+02	2.67211021E+01

4.03468550E+02 2.66597579E+01
4.03568550E+02 2.67879980E+01
4.03668550E+02 2.68709054E+01
4.03768550E+02 2.69099114E+01
4.08868550E+02 2.55718306E+01
4.09868550E+02 2.44594052E+01
4.10868550E+02 2.34308724E+01
4.11868550E+02 2.39580677E+01
4.12868550E+02 2.39022450E+01
4.22868550E+02 2.07973230E+01
4.32868550E+02 1.99108509E+01
4.42868550E+02 1.81844233E+01
4.52868550E+02 1.96985804E+01
4.62868550E+02 1.82168707E+01
4.67144758E+02 1.84263844E+01
4.72868550E+02 1.88556854E+01
4.82868550E+02 1.77351989E+01
4.92868550E+02 1.79438911E+01
5.01434275E+02 1.74536235E+01
5.02868550E+02 1.76662242E+01
5.67144758E+02 1.67395478E+01
6.01434275E+02 1.69430307E+01
6.02868550E+02 1.69921179E+01
6.67144758E+02 1.74833699E+01
6.71448584E+02 1.72278272E+01
6.71449584E+02 1.72268806E+01
6.71450584E+02 1.72265093E+01
6.71451584E+02 1.72263221E+01
6.71452584E+02 1.72261983E+01
6.71453584E+02 1.72260950E+01
6.71454584E+02 1.72259934E+01
6.71455584E+02 1.72258746E+01
6.71456584E+02 1.72257430E+01
6.71457584E+02 1.72255862E+01
6.71467584E+02 1.72232192E+01
6.71477584E+02 1.72202558E+01
6.71487584E+02 1.72176815E+01
6.71497584E+02 1.72158195E+01
6.71507584E+02 1.72146281E+01
6.71517584E+02 1.72139377E+01
6.71527584E+02 1.72135708E+01
6.71537584E+02 1.72133822E+01
6.71547584E+02 1.72132727E+01
6.71647584E+02 1.72079662E+01
6.71747584E+02 1.71964972E+01
6.71847584E+02 1.71886707E+01
6.71947584E+02 1.71780660E+01
6.72047584E+02 1.71591924E+01
6.72147584E+02 1.71413107E+01
6.72247584E+02 1.71460474E+01

6.72347584E+02 1.72247254E+01
6.77447584E+02 1.62554365E+01
6.78447584E+02 1.61043257E+01
6.79447584E+02 1.64191654E+01
6.80447584E+02 1.69250904E+01
6.81447584E+02 1.71115692E+01
6.91447584E+02 1.71587418E+01
7.01434275E+02 1.67946828E+01
7.01447584E+02 1.67949043E+01
7.02868550E+02 1.68984611E+01
7.11447584E+02 1.74870642E+01
7.21447584E+02 1.76147636E+01
7.31447584E+02 1.66820555E+01
7.41447584E+02 1.63360178E+01
7.51447584E+02 1.68333531E+01
7.61447584E+02 1.61837394E+01
7.67144758E+02 1.59613811E+01
7.71447584E+02 1.63253004E+01
8.01434275E+02 1.61389232E+01
8.02868550E+02 1.61704416E+01
8.67144758E+02 1.61730368E+01
8.71447584E+02 1.63390226E+01
9.01434275E+02 1.61364713E+01
9.02868550E+02 1.63602165E+01
9.67144758E+02 1.63704713E+01
9.71447584E+02 1.62779203E+01
1.00143428E+03 1.62024201E+01
1.00286855E+03 1.63878315E+01
1.00717238E+03 1.66971554E+01
1.00717338E+03 1.66970137E+01
1.00717438E+03 1.66969005E+01
1.00717538E+03 1.66967953E+01
1.00717638E+03 1.66967016E+01
1.00717738E+03 1.66966090E+01
1.00717838E+03 1.66965229E+01
1.00717938E+03 1.66964402E+01
1.00718038E+03 1.66963563E+01
1.00718138E+03 1.66962755E+01
1.00719138E+03 1.66955984E+01
1.00720138E+03 1.66950818E+01
1.00721138E+03 1.66946349E+01
1.00722138E+03 1.66941715E+01
1.00723138E+03 1.66936468E+01
1.00724138E+03 1.66930299E+01
1.00725138E+03 1.66923120E+01
1.00726138E+03 1.66915019E+01
1.00727138E+03 1.66906060E+01
1.00737138E+03 1.66797679E+01
1.00747138E+03 1.66748733E+01
1.00757138E+03 1.66809305E+01

1.00767138E+03 1.66835428E+01
1.00777138E+03 1.66842908E+01
1.00787138E+03 1.66856121E+01
1.00797138E+03 1.66886509E+01
1.00807138E+03 1.66939415E+01
1.01317138E+03 1.67797416E+01
1.01417138E+03 1.67800494E+01
1.01517138E+03 1.67791331E+01
1.01617138E+03 1.67712419E+01
1.01717138E+03 1.67430253E+01
1.02717138E+03 1.60770898E+01
1.03717138E+03 1.64378667E+01
1.04717138E+03 1.56813764E+01
1.05717138E+03 1.46851795E+01
1.06714476E+03 1.48892098E+01
1.06717138E+03 1.49318010E+01
1.07144758E+03 1.49347091E+01
1.07717138E+03 1.43052065E+01
1.08717138E+03 1.53285302E+01
1.09717138E+03 1.58270621E+01
1.10143428E+03 1.53456172E+01
1.10286855E+03 1.51644367E+01
1.10717138E+03 1.52959292E+01
1.16714476E+03 1.48698697E+01
1.17144758E+03 1.46190714E+01
1.20143428E+03 1.38699248E+01
1.20286855E+03 1.39749524E+01
1.20717138E+03 1.41996665E+01
1.26714476E+03 1.48203784E+01
1.27144758E+03 1.45473391E+01
1.30143428E+03 1.38387200E+01
1.30286855E+03 1.40498500E+01
1.30717138E+03 1.38199790E+01
1.36714476E+03 1.43626989E+01
1.37144758E+03 1.43626081E+01
1.40143428E+03 1.42014294E+01
1.40286855E+03 1.42295407E+01
1.40717138E+03 1.42487954E+01
1.46714476E+03 1.36161510E+01
1.47144758E+03 1.36664457E+01
1.50143428E+03 1.34212509E+01
1.50286855E+03 1.34289398E+01
1.50717138E+03 1.34790535E+01
1.56714476E+03 1.39058916E+01
1.57144758E+03 1.38386886E+01
1.60143428E+03 1.33987808E+01
1.60286855E+03 1.34447540E+01
1.60717138E+03 1.38456195E+01
1.66714476E+03 1.38397592E+01
1.67144758E+03 1.38501097E+01

1.70143428E+03	1.40242039E+01
1.70286855E+03	1.39652078E+01
1.70717138E+03	1.39410254E+01
1.76714476E+03	1.36682667E+01
1.77144758E+03	1.36524490E+01
1.80143428E+03	1.36003667E+01
1.80286855E+03	1.35010493E+01
1.80717138E+03	1.37039731E+01
1.86714476E+03	1.38525058E+01
1.87144758E+03	1.37560014E+01
1.90143428E+03	1.38152065E+01
1.90286855E+03	1.38152637E+01
1.90717138E+03	1.38068177E+01
1.96714476E+03	1.36297044E+01
1.97144758E+03	1.39440659E+01
2.00143428E+03	1.33356556E+01
2.00286855E+03	1.33314667E+01
2.00717138E+03	1.34679457E+01
2.06714476E+03	1.39616950E+01
2.07144758E+03	1.39228094E+01
2.10143428E+03	1.40247962E+01
2.10286855E+03	1.40352884E+01
2.10717138E+03	1.40555393E+01
2.16714476E+03	1.37082343E+01
2.17144758E+03	1.36637468E+01
2.20143428E+03	1.37305430E+01
2.20286855E+03	1.38537762E+01
2.20717138E+03	1.39490928E+01
2.26714476E+03	1.38419554E+01
2.27144758E+03	1.38627265E+01
2.30143428E+03	1.37463099E+01
2.30286855E+03	1.37319751E+01
2.30717138E+03	1.36938876E+01
2.36714476E+03	1.36533773E+01
2.37144758E+03	1.36688658E+01
2.40143428E+03	1.38176655E+01
2.40286855E+03	1.38156722E+01
2.40717138E+03	1.37802877E+01
2.46714476E+03	1.35135785E+01
2.47144758E+03	1.34411359E+01
2.50143428E+03	1.33727585E+01
2.50286855E+03	1.33708042E+01
2.50717138E+03	1.33636893E+01
2.56714476E+03	1.33700506E+01
2.57144758E+03	1.33422093E+01
2.60143428E+03	1.32505417E+01
2.60286855E+03	1.32438052E+01
2.60717138E+03	1.32194357E+01
2.66714476E+03	1.31586288E+01
2.67144758E+03	1.31566250E+01

2.70143428E+03 1.30935497E+01
2.70286855E+03 1.30882271E+01
2.70717138E+03 1.30683655E+01
2.76714476E+03 1.28514388E+01
2.77144758E+03 1.28164168E+01
2.80143428E+03 1.28246227E+01
2.80286855E+03 1.28088867E+01
2.80717138E+03 1.28235862E+01
2.86714476E+03 1.28577535E+01
2.87144758E+03 1.29018221E+01
2.90143428E+03 1.28940165E+01
2.90286855E+03 1.28898830E+01
2.90717138E+03 1.28756768E+01
2.96714476E+03 1.26494922E+01
2.97144758E+03 1.26320424E+01
3.00143428E+03 1.27235216E+01
3.00286855E+03 1.27134208E+01
3.00717138E+03 1.26837457E+01
3.07144758E+03 1.26137018E+01
3.10143428E+03 1.27080132E+01
3.10286855E+03 1.27052635E+01
3.10717138E+03 1.26912086E+01
3.17144758E+03 1.24897548E+01
3.20286855E+03 1.24707931E+01
3.20717138E+03 1.24272769E+01
3.27144758E+03 1.24998050E+01
3.30286855E+03 1.24629644E+01
3.30717138E+03 1.24777078E+01
3.37144758E+03 1.24251434E+01
3.40717138E+03 1.23278659E+01
3.47144758E+03 1.23853184E+01
3.50717138E+03 1.22928842E+01
3.57144758E+03 1.22446404E+01
3.60717138E+03 1.22777228E+01
3.70717138E+03 1.22441506E+01
3.80717138E+03 1.20735548E+01
3.90717138E+03 1.21649891E+01

#Tot_ener(cm-1) 1---3 Cross_sec(angs^2)
4.02869550E+02 5.34703868E-02
4.02870550E+02 6.92780968E-02
4.02871550E+02 7.98043497E-02
4.02872550E+02 8.79105479E-02
4.02873550E+02 9.46428243E-02
4.02874550E+02 1.00506505E-01
4.02875550E+02 1.05783664E-01
4.02876550E+02 1.10646101E-01
4.02877550E+02 1.15205454E-01
4.02878550E+02 1.19537168E-01
4.02888550E+02 1.57163587E-01

4.02898550E+02 1.90922172E-01
4.02908550E+02 2.23095959E-01
4.02918550E+02 2.54342518E-01
4.02928550E+02 2.84902394E-01
4.02938550E+02 3.14706709E-01
4.02948550E+02 3.43543987E-01
4.02958550E+02 3.71196728E-01
4.02968550E+02 3.97521150E-01
4.03068550E+02 6.09748463E-01
4.03168550E+02 7.79342575E-01
4.03268550E+02 8.85149548E-01
4.03368550E+02 1.00315245E+00
4.03468550E+02 1.09614627E+00
4.03568550E+02 1.18170795E+00
4.03668550E+02 1.27621399E+00
4.03768550E+02 1.35534484E+00
4.08868550E+02 3.69298171E+00
4.09868550E+02 4.03523285E+00
4.10868550E+02 4.23104194E+00
4.11868550E+02 4.53788991E+00
4.12868550E+02 4.90534951E+00
4.22868550E+02 7.05070414E+00
4.32868550E+02 8.38287090E+00
4.42868550E+02 1.00078198E+01
4.52868550E+02 1.04772757E+01
4.62868550E+02 1.27985995E+01
4.67144758E+02 1.23015428E+01
4.72868550E+02 1.21314670E+01
4.82868550E+02 1.29274907E+01
4.92868550E+02 1.30558802E+01
5.01434275E+02 1.31713326E+01
5.02868550E+02 1.32318754E+01
5.67144758E+02 1.26985909E+01
6.01434275E+02 1.32145457E+01
6.02868550E+02 1.32762591E+01
6.67144758E+02 1.30342021E+01
6.71448584E+02 1.31805701E+01
6.71449584E+02 1.31812342E+01
6.71450584E+02 1.31817400E+01
6.71451584E+02 1.31821173E+01
6.71452584E+02 1.31823927E+01
6.71453584E+02 1.31825862E+01
6.71454584E+02 1.31827121E+01
6.71455584E+02 1.31827818E+01
6.71456584E+02 1.31828056E+01
6.71457584E+02 1.31827910E+01
6.71467584E+02 1.31815211E+01
6.71477584E+02 1.31799499E+01
6.71487584E+02 1.31789202E+01
6.71497584E+02 1.31783857E+01

6.71507584E+02	1.31781131E+01
6.71517584E+02	1.31778999E+01
6.71527584E+02	1.31776069E+01
6.71537584E+02	1.31771502E+01
6.71547584E+02	1.31764806E+01
6.71647584E+02	1.31556233E+01
6.71747584E+02	1.31158385E+01
6.71847584E+02	1.30693316E+01
6.71947584E+02	1.30287330E+01
6.72047584E+02	1.30027356E+01
6.72147584E+02	1.29931804E+01
6.72247584E+02	1.29942288E+01
6.72347584E+02	1.29975473E+01
6.77447584E+02	1.27268984E+01
6.78447584E+02	1.27941887E+01
6.79447584E+02	1.27198311E+01
6.80447584E+02	1.24562086E+01
6.81447584E+02	1.22240826E+01
6.91447584E+02	1.22464361E+01
7.01434275E+02	1.24246269E+01
7.01447584E+02	1.24237116E+01
7.02868550E+02	1.22467346E+01
7.11447584E+02	1.20186856E+01
7.21447584E+02	1.18215016E+01
7.31447584E+02	1.18198031E+01
7.41447584E+02	1.16658814E+01
7.51447584E+02	1.09865917E+01
7.61447584E+02	1.12066960E+01
7.67144758E+02	1.10634079E+01
7.71447584E+02	1.13009287E+01
8.01434275E+02	1.08353999E+01
8.02868550E+02	1.07967682E+01
8.67144758E+02	1.08041272E+01
8.71447584E+02	1.06714239E+01
9.01434275E+02	1.00711107E+01
9.02868550E+02	1.01254635E+01
9.67144758E+02	1.01863151E+01
9.71447584E+02	1.02249523E+01
1.00143428E+03	1.00648865E+01
1.00286855E+03	9.89757678E+00
1.00717238E+03	9.88831774E+00
1.00717338E+03	9.88807821E+00
1.00717438E+03	9.88784854E+00
1.00717538E+03	9.88761594E+00
1.00717638E+03	9.88737999E+00
1.00717738E+03	9.88713870E+00
1.00717838E+03	9.88689124E+00
1.00717938E+03	9.88663799E+00
1.00718038E+03	9.88637727E+00
1.00718138E+03	9.88611203E+00

1.00719138E+03	9.88321495E+00
1.00720138E+03	9.88013472E+00
1.00721138E+03	9.87710787E+00
1.00722138E+03	9.87424275E+00
1.00723138E+03	9.87158424E+00
1.00724138E+03	9.86914724E+00
1.00725138E+03	9.86693463E+00
1.00726138E+03	9.86494403E+00
1.00727138E+03	9.86316527E+00
1.00737138E+03	9.85353555E+00
1.00747138E+03	9.84306631E+00
1.00757138E+03	9.82618157E+00
1.00767138E+03	9.81791455E+00
1.00777138E+03	9.80996159E+00
1.00787138E+03	9.79704959E+00
1.00797138E+03	9.77989443E+00
1.00807138E+03	9.76206634E+00
1.01317138E+03	9.80672076E+00
1.01417138E+03	9.76573424E+00
1.01517138E+03	9.74807221E+00
1.01617138E+03	9.71957884E+00
1.01717138E+03	9.68740365E+00
1.02717138E+03	9.71786086E+00
1.03717138E+03	9.64284326E+00
1.04717138E+03	9.89107860E+00
1.05717138E+03	1.00491571E+01
1.06714476E+03	9.17804267E+00
1.06717138E+03	9.13821593E+00
1.07144758E+03	9.02092654E+00
1.07717138E+03	9.49539903E+00
1.08717138E+03	9.38406060E+00
1.09717138E+03	9.43303192E+00
1.10143428E+03	9.08922345E+00
1.10286855E+03	9.00676217E+00
1.10717138E+03	8.85353344E+00
1.16714476E+03	9.13384184E+00
1.17144758E+03	9.15501094E+00
1.20143428E+03	8.70759098E+00
1.20286855E+03	8.69992254E+00
1.20717138E+03	8.80224728E+00
1.26714476E+03	8.52413729E+00
1.27144758E+03	8.54372042E+00
1.30143428E+03	8.66341772E+00
1.30286855E+03	8.87487789E+00
1.30717138E+03	8.95917439E+00
1.36714476E+03	8.75756692E+00
1.37144758E+03	8.50424508E+00
1.40143428E+03	8.33759990E+00
1.40286855E+03	8.28252143E+00
1.40717138E+03	8.31369464E+00

1.46714476E+03 8.51250761E+00
1.47144758E+03 8.60228736E+00
1.50143428E+03 8.11131230E+00
1.50286855E+03 8.11653624E+00
1.50717138E+03 8.12034886E+00
1.56714476E+03 8.25661533E+00
1.57144758E+03 8.33476756E+00
1.60143428E+03 8.36066664E+00
1.60286855E+03 8.37855711E+00
1.60717138E+03 8.49856615E+00
1.66714476E+03 7.97381735E+00
1.67144758E+03 8.08048490E+00
1.70143428E+03 8.27670382E+00
1.70286855E+03 8.30357097E+00
1.70717138E+03 8.31923214E+00
1.76714476E+03 8.31002248E+00
1.77144758E+03 8.20880103E+00
1.80143428E+03 8.28677376E+00
1.80286855E+03 8.25344301E+00
1.80717138E+03 8.09543312E+00
1.86714476E+03 8.32918671E+00
1.87144758E+03 8.15123689E+00
1.90143428E+03 8.17609128E+00
1.90286855E+03 8.16384625E+00
1.90717138E+03 8.13146304E+00
1.96714476E+03 7.82930279E+00
1.97144758E+03 7.72814616E+00
2.00143428E+03 7.74368838E+00
2.00286855E+03 7.71542432E+00
2.00717138E+03 7.62993231E+00
2.06714476E+03 7.62339042E+00
2.07144758E+03 7.61859873E+00
2.10143428E+03 7.64861980E+00
2.10286855E+03 7.64316216E+00
2.10717138E+03 7.62507391E+00
2.16714476E+03 7.60422143E+00
2.17144758E+03 7.57423371E+00
2.20143428E+03 7.42372848E+00
2.20286855E+03 7.44474113E+00
2.20717138E+03 7.44108352E+00
2.26714476E+03 7.58308561E+00
2.27144758E+03 7.58330449E+00
2.30143428E+03 7.57619890E+00
2.30286855E+03 7.57492268E+00
2.30717138E+03 7.56829359E+00
2.36714476E+03 7.40068317E+00
2.37144758E+03 7.33283801E+00
2.40143428E+03 7.42332852E+00
2.40286855E+03 7.43015097E+00
2.40717138E+03 7.45721313E+00

2.46714476E+03	7.36358171E+00
2.47144758E+03	7.39748272E+00
2.50143428E+03	7.35215471E+00
2.50286855E+03	7.35438017E+00
2.50717138E+03	7.36599589E+00
2.56714476E+03	7.25813064E+00
2.57144758E+03	7.27898488E+00
2.60143428E+03	7.22282688E+00
2.60286855E+03	7.23764170E+00
2.60717138E+03	7.26935634E+00
2.66714476E+03	7.02431141E+00
2.67144758E+03	7.04906536E+00
2.70143428E+03	7.12029347E+00
2.70286855E+03	7.12020001E+00
2.70717138E+03	7.12125355E+00
2.76714476E+03	7.12309368E+00
2.77144758E+03	7.05969391E+00
2.80143428E+03	7.07335560E+00
2.80286855E+03	7.07868760E+00
2.80717138E+03	7.09048043E+00
2.86714476E+03	6.89324606E+00
2.87144758E+03	6.91803754E+00
2.90143428E+03	7.00100565E+00
2.90286855E+03	7.00305633E+00
2.90717138E+03	7.01026623E+00
2.96714476E+03	6.98479575E+00
2.97144758E+03	6.95358208E+00
3.00143428E+03	6.95005477E+00
3.00286855E+03	6.95725148E+00
3.00717138E+03	6.98340384E+00
3.07144758E+03	6.92373870E+00
3.10143428E+03	6.97162962E+00
3.10286855E+03	6.97301450E+00
3.10717138E+03	6.97534316E+00
3.17144758E+03	6.86954850E+00
3.20286855E+03	6.90993960E+00
3.20717138E+03	6.90019544E+00
3.27144758E+03	6.85141615E+00
3.30286855E+03	6.87637629E+00
3.30717138E+03	6.87813316E+00
3.37144758E+03	6.78638541E+00
3.40717138E+03	6.80438638E+00
3.47144758E+03	6.71957494E+00
3.50717138E+03	6.71497339E+00
3.57144758E+03	6.65548795E+00
3.60717138E+03	6.51865248E+00
3.70717138E+03	6.48890572E+00
3.80717138E+03	6.35193643E+00
3.90717138E+03	6.19801739E+00

#Tot_ener(cm-1) 2---3 Cross_sec(angs^2)
4.02869550E+02 3.79383567E-02
4.02870550E+02 4.93702101E-02
4.02871550E+02 5.71481454E-02
4.02872550E+02 6.32806971E-02
4.02873550E+02 6.84988852E-02
4.02874550E+02 7.31541357E-02
4.02875550E+02 7.74414799E-02
4.02876550E+02 8.14789484E-02
4.02877550E+02 8.53428062E-02
4.02878550E+02 8.90840523E-02
4.02888550E+02 1.24278564E-01
4.02898550E+02 1.59481580E-01
4.02908550E+02 1.95743105E-01
4.02918550E+02 2.32878131E-01
4.02928550E+02 2.70291541E-01
4.02938550E+02 3.07222724E-01
4.02948550E+02 3.43009160E-01
4.02958550E+02 3.77225243E-01
4.02968550E+02 4.09727713E-01
4.03068550E+02 7.04309530E-01
4.03168550E+02 9.19474684E-01
4.03268550E+02 1.10662502E+00
4.03368550E+02 1.49635158E+00
4.03468550E+02 1.69277820E+00
4.03568550E+02 1.75657183E+00
4.03668550E+02 1.84702247E+00
4.03768550E+02 1.93677909E+00
4.08868550E+02 5.57522734E+00
4.09868550E+02 6.26653493E+00
4.10868550E+02 6.48429633E+00
4.11868550E+02 6.99028446E+00
4.12868550E+02 7.70817493E+00
4.22868550E+02 9.73101138E+00
4.32868550E+02 1.18458807E+01
4.42868550E+02 1.23939491E+01
4.52868550E+02 1.44921782E+01
4.62868550E+02 1.55673980E+01
4.67144758E+02 1.50014704E+01
4.72868550E+02 1.49563994E+01
4.82868550E+02 1.56736772E+01
4.92868550E+02 1.68980228E+01
5.01434275E+02 1.57981815E+01
5.02868550E+02 1.58740263E+01
5.67144758E+02 1.74739989E+01
6.01434275E+02 1.63173009E+01
6.02868550E+02 1.64944159E+01
6.67144758E+02 1.67203122E+01
6.71448584E+02 1.63847827E+01
6.71449584E+02 1.63807163E+01

6.71450584E+02	1.63786091E+01
6.71451584E+02	1.63773051E+01
6.71452584E+02	1.63764230E+01
6.71453584E+02	1.63757874E+01
6.71454584E+02	1.63753119E+01
6.71455584E+02	1.63749430E+01
6.71456584E+02	1.63746453E+01
6.71457584E+02	1.63744013E+01
6.71467584E+02	1.63728705E+01
6.71477584E+02	1.63712512E+01
6.71487584E+02	1.63692266E+01
6.71497584E+02	1.63670288E+01
6.71507584E+02	1.63648601E+01
6.71517584E+02	1.63628451E+01
6.71527584E+02	1.63610545E+01
6.71537584E+02	1.63595299E+01
6.71547584E+02	1.63582887E+01
6.71647584E+02	1.63583550E+01
6.71747584E+02	1.63635439E+01
6.71847584E+02	1.63592173E+01
6.71947584E+02	1.63359945E+01
6.72047584E+02	1.62775342E+01
6.72147584E+02	1.61792293E+01
6.72247584E+02	1.60686810E+01
6.72347584E+02	1.59693017E+01
6.77447584E+02	1.53517848E+01
6.78447584E+02	1.54728372E+01
6.79447584E+02	1.55295635E+01
6.80447584E+02	1.54231271E+01
6.81447584E+02	1.53953687E+01
6.91447584E+02	1.49365726E+01
7.01434275E+02	1.39892229E+01
7.01447584E+02	1.39898482E+01
7.02868550E+02	1.42250408E+01
7.11447584E+02	1.47324333E+01
7.21447584E+02	1.37132314E+01
7.31447584E+02	1.33848357E+01
7.41447584E+02	1.34020470E+01
7.51447584E+02	1.29441458E+01
7.61447584E+02	1.29616574E+01
7.67144758E+02	1.31178136E+01
7.71447584E+02	1.33695402E+01
8.01434275E+02	1.28822730E+01
8.02868550E+02	1.28939099E+01
8.67144758E+02	1.23314986E+01
8.71447584E+02	1.22538336E+01
9.01434275E+02	1.25178600E+01
9.02868550E+02	1.25436606E+01
9.67144758E+02	1.27964091E+01
9.71447584E+02	1.26269323E+01

1.00143428E+03 1.18676356E+01
1.00286855E+03 1.19472352E+01
1.00717238E+03 1.20007382E+01
1.00717338E+03 1.20007770E+01
1.00717438E+03 1.20007781E+01
1.00717538E+03 1.20007574E+01
1.00717638E+03 1.20007195E+01
1.00717738E+03 1.20006706E+01
1.00717838E+03 1.20006112E+01
1.00717938E+03 1.20005444E+01
1.00718038E+03 1.20004725E+01
1.00718138E+03 1.20003958E+01
1.00719138E+03 1.19995868E+01
1.00720138E+03 1.19989275E+01
1.00721138E+03 1.19984451E+01
1.00722138E+03 1.19980573E+01
1.00723138E+03 1.19976920E+01
1.00724138E+03 1.19973017E+01
1.00725138E+03 1.19968610E+01
1.00726138E+03 1.19963572E+01
1.00727138E+03 1.19957893E+01
1.00737138E+03 1.19881370E+01
1.00747138E+03 1.19789105E+01
1.00757138E+03 1.19731936E+01
1.00767138E+03 1.19698721E+01
1.00777138E+03 1.19654392E+01
1.00787138E+03 1.19615561E+01
1.00797138E+03 1.19589970E+01
1.00807138E+03 1.19574517E+01
1.01317138E+03 1.18424922E+01
1.01417138E+03 1.18044364E+01
1.01517138E+03 1.17792483E+01
1.01617138E+03 1.17776652E+01
1.01717138E+03 1.18079121E+01
1.02717138E+03 1.20558050E+01
1.03717138E+03 1.16909887E+01
1.04717138E+03 1.17408669E+01
1.05717138E+03 1.19203411E+01
1.06714476E+03 1.14387504E+01
1.06717138E+03 1.14230285E+01
1.07144758E+03 1.16593117E+01
1.07717138E+03 1.15737518E+01
1.08717138E+03 1.11811686E+01
1.09717138E+03 1.12162727E+01
1.10143428E+03 1.12196394E+01
1.10286855E+03 1.12247509E+01
1.10717138E+03 1.11075247E+01
1.16714476E+03 1.12718904E+01
1.17144758E+03 1.12606996E+01
1.20143428E+03 1.11578187E+01

1.20286855E+03 1.11554628E+01
1.20717138E+03 1.13999342E+01
1.26714476E+03 1.14662577E+01
1.27144758E+03 1.15364949E+01
1.30143428E+03 1.14758460E+01
1.30286855E+03 1.15363516E+01
1.30717138E+03 1.17788904E+01
1.36714476E+03 1.13854413E+01
1.37144758E+03 1.13126747E+01
1.40143428E+03 1.18643291E+01
1.40286855E+03 1.18828587E+01
1.40717138E+03 1.19165696E+01
1.46714476E+03 1.08073084E+01
1.47144758E+03 1.09039373E+01
1.50143428E+03 1.11644187E+01
1.50286855E+03 1.11662778E+01
1.50717138E+03 1.11550291E+01
1.56714476E+03 1.09813159E+01
1.57144758E+03 1.09637408E+01
1.60143428E+03 1.07666645E+01
1.60286855E+03 1.07816453E+01
1.60717138E+03 1.08104686E+01
1.66714476E+03 1.12529785E+01
1.67144758E+03 1.12897380E+01
1.70143428E+03 1.11774219E+01
1.70286855E+03 1.11020331E+01
1.70717138E+03 1.10756754E+01
1.76714476E+03 1.10282496E+01
1.77144758E+03 1.10066446E+01
1.80143428E+03 1.11989146E+01
1.80286855E+03 1.12576636E+01
1.80717138E+03 1.13774531E+01
1.86714476E+03 1.12598844E+01
1.87144758E+03 1.13583799E+01
1.90143428E+03 1.14374756E+01
1.90286855E+03 1.14415870E+01
1.90717138E+03 1.14220809E+01
1.96714476E+03 1.12658947E+01
1.97144758E+03 1.13552058E+01
2.00143428E+03 1.12704376E+01
2.00286855E+03 1.12974477E+01
2.00717138E+03 1.13639898E+01
2.06714476E+03 1.14885310E+01
2.07144758E+03 1.14839639E+01
2.10143428E+03 1.13622460E+01
2.10286855E+03 1.13565062E+01
2.10717138E+03 1.13455389E+01
2.16714476E+03 1.13389156E+01
2.17144758E+03 1.14367803E+01
2.20143428E+03 1.15321960E+01

2.20286855E+03	1.14322846E+01
2.20717138E+03	1.16472519E+01
2.26714476E+03	1.13143943E+01
2.27144758E+03	1.13628877E+01
2.30143428E+03	1.12451839E+01
2.30286855E+03	1.12433262E+01
2.30717138E+03	1.12378671E+01
2.36714476E+03	1.11492725E+01
2.37144758E+03	1.12170856E+01
2.40143428E+03	1.14153320E+01
2.40286855E+03	1.14055685E+01
2.40717138E+03	1.13740034E+01
2.46714476E+03	1.12790161E+01
2.47144758E+03	1.12919809E+01
2.50143428E+03	1.12471225E+01
2.50286855E+03	1.12425438E+01
2.50717138E+03	1.12292872E+01
2.56714476E+03	1.12243500E+01
2.57144758E+03	1.12400957E+01
2.60143428E+03	1.11939842E+01
2.60286855E+03	1.11901996E+01
2.60717138E+03	1.11842686E+01
2.66714476E+03	1.12217761E+01
2.67144758E+03	1.12351224E+01
2.70143428E+03	1.11189684E+01
2.70286855E+03	1.11156382E+01
2.70717138E+03	1.11039964E+01
2.76714476E+03	1.10244227E+01
2.77144758E+03	1.10653812E+01
2.80143428E+03	1.10195642E+01
2.80286855E+03	1.10123675E+01
2.80717138E+03	1.10127950E+01
2.86714476E+03	1.10596622E+01
2.87144758E+03	1.09769654E+01
2.90143428E+03	1.10328502E+01
2.90286855E+03	1.10306868E+01
2.90717138E+03	1.10218241E+01
2.96714476E+03	1.09070854E+01
2.97144758E+03	1.09027376E+01
3.00143428E+03	1.09326926E+01
3.00286855E+03	1.09249651E+01
3.00717138E+03	1.09062418E+01
3.07144758E+03	1.08633831E+01
3.10143428E+03	1.08443946E+01
3.10286855E+03	1.08448662E+01
3.10717138E+03	1.08417564E+01
3.17144758E+03	1.07501986E+01
3.20286855E+03	1.07263169E+01
3.20717138E+03	1.07050532E+01
3.27144758E+03	1.06666076E+01

3.30286855E+03 1.06290912E+01
3.30717138E+03 1.06354789E+01
3.37144758E+03 1.06638516E+01
3.40717138E+03 1.06369172E+01
3.47144758E+03 1.06097579E+01
3.50717138E+03 1.05765946E+01
3.57144758E+03 1.05625609E+01
3.60717138E+03 1.05835107E+01
3.70717138E+03 1.05240796E+01
3.80717138E+03 1.04717962E+01
3.90717138E+03 1.04521119E+01

#Tot_ener(cm-1) 2--->4 Cross_sec(angs^2)
6.71448584E+02 6.51198160E-02
6.71449584E+02 7.40884805E-02
6.71450584E+02 7.85769075E-02
6.71451584E+02 8.17037817E-02
6.71452584E+02 8.43615692E-02
6.71453584E+02 8.68891931E-02
6.71454584E+02 8.94316354E-02
6.71455584E+02 9.20522814E-02
6.71456584E+02 9.47761829E-02
6.71457584E+02 9.76090412E-02
6.71467584E+02 1.29680732E-01
6.71477584E+02 1.61029938E-01
6.71487584E+02 1.86635174E-01
6.71497584E+02 2.06493353E-01
6.71507584E+02 2.22173138E-01
6.71517584E+02 2.35246610E-01
6.71527584E+02 2.46863165E-01
6.71537584E+02 2.57730418E-01
6.71547584E+02 2.68242172E-01
6.71647584E+02 3.62453794E-01
6.71747584E+02 4.26613916E-01
6.71847584E+02 4.81656232E-01
6.71947584E+02 5.57537464E-01
6.72047584E+02 6.67867384E-01
6.72147584E+02 7.85468841E-01
6.72247584E+02 8.69951654E-01
6.72347584E+02 9.18096538E-01
6.77447584E+02 2.19745053E+00
6.78447584E+02 2.42468139E+00
6.79447584E+02 2.59055711E+00
6.80447584E+02 2.71347812E+00
6.81447584E+02 2.87164505E+00
6.91447584E+02 3.86843457E+00
7.01434275E+02 4.84556107E+00
7.01447584E+02 4.84638780E+00
7.02868550E+02 4.89451449E+00
7.11447584E+02 5.38913214E+00

7.21447584E+02	6.10100990E+00
7.31447584E+02	6.85677438E+00
7.41447584E+02	7.20043523E+00
7.51447584E+02	8.23113700E+00
7.61447584E+02	8.20005579E+00
7.67144758E+02	8.35805567E+00
7.71447584E+02	8.64186855E+00
8.01434275E+02	9.63048389E+00
8.02868550E+02	9.85050006E+00
8.67144758E+02	9.95581232E+00
8.71447584E+02	9.88800436E+00
9.01434275E+02	9.92224650E+00
9.02868550E+02	9.92253334E+00
9.67144758E+02	9.68464075E+00
9.71447584E+02	9.70208340E+00
1.00143428E+03	1.05460444E+01
1.00286855E+03	1.03755015E+01
1.00717238E+03	1.03959874E+01
1.00717338E+03	1.03954919E+01
1.00717438E+03	1.03949969E+01
1.00717538E+03	1.03944981E+01
1.00717638E+03	1.03939966E+01
1.00717738E+03	1.03934933E+01
1.00717838E+03	1.03929900E+01
1.00717938E+03	1.03924881E+01
1.00718038E+03	1.03919885E+01
1.00718138E+03	1.03914928E+01
1.00719138E+03	1.03868261E+01
1.00720138E+03	1.03827070E+01
1.00721138E+03	1.03789688E+01
1.00722138E+03	1.03754494E+01
1.00723138E+03	1.03720519E+01
1.00724138E+03	1.03687248E+01
1.00725138E+03	1.03654473E+01
1.00726138E+03	1.03622134E+01
1.00727138E+03	1.03590225E+01
1.00737138E+03	1.03279378E+01
1.00747138E+03	1.02855928E+01
1.00757138E+03	1.02403410E+01
1.00767138E+03	1.02064499E+01
1.00777138E+03	1.01785567E+01
1.00787138E+03	1.01521934E+01
1.00797138E+03	1.01267586E+01
1.00807138E+03	1.01024302E+01
1.01317138E+03	9.59105958E+00
1.01417138E+03	9.56187739E+00
1.01517138E+03	9.57007218E+00
1.01617138E+03	9.56415941E+00
1.01717138E+03	9.55516628E+00
1.02717138E+03	9.52318495E+00

1.03717138E+03	9.48431546E+00
1.04717138E+03	9.62988615E+00
1.05717138E+03	9.56615601E+00
1.06714476E+03	9.27645714E+00
1.06717138E+03	9.28027521E+00
1.07144758E+03	9.16081823E+00
1.07717138E+03	8.86940149E+00
1.08717138E+03	9.03992780E+00
1.09717138E+03	9.08817698E+00
1.10143428E+03	9.11682757E+00
1.10286855E+03	9.15877498E+00
1.10717138E+03	9.06244172E+00
1.16714476E+03	8.77084386E+00
1.17144758E+03	8.81277043E+00
1.20143428E+03	8.51418096E+00
1.20286855E+03	8.49348806E+00
1.20717138E+03	8.44697712E+00
1.26714476E+03	8.34031586E+00
1.27144758E+03	8.36651484E+00
1.30143428E+03	8.03762686E+00
1.30286855E+03	8.04302073E+00
1.30717138E+03	7.99269331E+00
1.36714476E+03	7.60362325E+00
1.37144758E+03	7.66988740E+00
1.40143428E+03	8.07412628E+00
1.40286855E+03	8.05767352E+00
1.40717138E+03	7.96743790E+00
1.46714476E+03	7.52996162E+00
1.47144758E+03	7.48676828E+00
1.50143428E+03	7.16069182E+00
1.50286855E+03	7.14576065E+00
1.50717138E+03	7.14513339E+00
1.56714476E+03	7.12965074E+00
1.57144758E+03	7.14602830E+00
1.60143428E+03	7.28678417E+00
1.60286855E+03	7.30466195E+00
1.60717138E+03	7.34176833E+00
1.66714476E+03	6.88636943E+00
1.67144758E+03	6.91974067E+00
1.70143428E+03	7.05347786E+00
1.70286855E+03	7.05982029E+00
1.70717138E+03	7.07568347E+00
1.76714476E+03	7.12839753E+00
1.77144758E+03	7.14809861E+00
1.80143428E+03	7.19434478E+00
1.80286855E+03	7.20695948E+00
1.80717138E+03	7.12295353E+00
1.86714476E+03	6.91701245E+00
1.87144758E+03	6.99986787E+00
1.90143428E+03	6.89117254E+00

1.90286855E+03	6.90188631E+00
1.90717138E+03	6.92416056E+00
1.96714476E+03	7.04328969E+00
1.97144758E+03	6.99484651E+00
2.00143428E+03	6.76578293E+00
2.00286855E+03	6.75706192E+00
2.00717138E+03	6.70227095E+00
2.06714476E+03	6.78337910E+00
2.07144758E+03	6.79698656E+00
2.10143428E+03	6.87074226E+00
2.10286855E+03	6.87510300E+00
2.10717138E+03	6.88402826E+00
2.16714476E+03	6.55032078E+00
2.17144758E+03	6.55581297E+00
2.20143428E+03	6.46608820E+00
2.20286855E+03	6.42673888E+00
2.20717138E+03	6.42671029E+00
2.26714476E+03	6.67463582E+00
2.27144758E+03	6.66262238E+00
2.30143428E+03	6.53140186E+00
2.30286855E+03	6.52570878E+00
2.30717138E+03	6.50893376E+00
2.36714476E+03	6.42805350E+00
2.37144758E+03	6.37084776E+00
2.40143428E+03	6.33144184E+00
2.40286855E+03	6.33632833E+00
2.40717138E+03	6.36239006E+00
2.46714476E+03	6.37047819E+00
2.47144758E+03	6.33650554E+00
2.50143428E+03	6.26295739E+00
2.50286855E+03	6.27045820E+00
2.50717138E+03	6.29501793E+00
2.56714476E+03	6.16130138E+00
2.57144758E+03	6.14625637E+00
2.60143428E+03	6.10783716E+00
2.60286855E+03	6.10904337E+00
2.60717138E+03	6.11376168E+00
2.66714476E+03	6.07161286E+00
2.67144758E+03	6.02752519E+00
2.70143428E+03	6.01807415E+00
2.70286855E+03	6.01848095E+00
2.70717138E+03	6.01782106E+00
2.76714476E+03	6.03741442E+00
2.77144758E+03	6.00334076E+00
2.80143428E+03	5.92892769E+00
2.80286855E+03	5.92874230E+00
2.80717138E+03	5.92866806E+00
2.86714476E+03	6.02572344E+00
2.87144758E+03	5.92845401E+00
2.90143428E+03	5.96027124E+00

2.90286855E+03 5.96164064E+00
2.90717138E+03 5.96533615E+00
2.96714476E+03 5.92907585E+00
2.97144758E+03 5.88170324E+00
3.00143428E+03 5.80074339E+00
3.00286855E+03 5.80365321E+00
3.00717138E+03 5.80800270E+00
3.07144758E+03 5.83253953E+00
3.10143428E+03 5.78179497E+00
3.10286855E+03 5.78361470E+00
3.10717138E+03 5.78673149E+00
3.17144758E+03 5.70829377E+00
3.20286855E+03 5.65492211E+00
3.20717138E+03 5.64247436E+00
3.27144758E+03 5.62071710E+00
3.30286855E+03 5.53249535E+00
3.30717138E+03 5.52093379E+00
3.37144758E+03 5.52038425E+00
3.40717138E+03 5.47488513E+00
3.47144758E+03 5.42857235E+00
3.50717138E+03 5.37107275E+00
3.57144758E+03 5.26302296E+00
3.60717138E+03 5.26817875E+00
3.70717138E+03 5.21512492E+00
3.80717138E+03 5.12061927E+00
3.90717138E+03 5.03970076E+00

#Tot_ener(cm-1) 3--->4 Cross_sec(angs^2)
6.71448584E+02 3.02061130E-02
6.71449584E+02 3.47594362E-02
6.71450584E+02 3.73949673E-02
6.71451584E+02 3.95199384E-02
6.71452584E+02 4.15254178E-02
6.71453584E+02 4.35518748E-02
6.71454584E+02 4.56528926E-02
6.71455584E+02 4.78461581E-02
6.71456584E+02 5.01329612E-02
6.71457584E+02 5.25067091E-02
6.71467584E+02 7.85199697E-02
6.71477584E+02 1.02767628E-01
6.71487584E+02 1.22197702E-01
6.71497584E+02 1.37452127E-01
6.71507584E+02 1.49973157E-01
6.71517584E+02 1.60964902E-01
6.71527584E+02 1.71207500E-01
6.71537584E+02 1.81124266E-01
6.71547584E+02 1.90912218E-01
6.71647584E+02 2.82174364E-01
6.71747584E+02 3.62885633E-01
6.71847584E+02 4.54244903E-01

6.71947584E+02	5.71044380E-01
6.72047584E+02	7.13534111E-01
6.72147584E+02	8.49644421E-01
6.72247584E+02	9.44999375E-01
6.72347584E+02	1.00776317E+00
6.77447584E+02	2.97449350E+00
6.78447584E+02	3.32214230E+00
6.79447584E+02	3.52207465E+00
6.80447584E+02	3.72640850E+00
6.81447584E+02	4.07719342E+00
6.91447584E+02	6.19330018E+00
7.01434275E+02	7.00562319E+00
7.01447584E+02	7.00719955E+00
7.02868550E+02	7.18699236E+00
7.11447584E+02	8.29049286E+00
7.21447584E+02	9.31596063E+00
7.31447584E+02	9.49631173E+00
7.41447584E+02	9.98482177E+00
7.51447584E+02	1.10838280E+01
7.61447584E+02	1.09368613E+01
7.67144758E+02	1.11324813E+01
7.71447584E+02	1.12338952E+01
8.01434275E+02	1.23473035E+01
8.02868550E+02	1.24446625E+01
8.67144758E+02	1.31385556E+01
8.71447584E+02	1.31981853E+01
9.01434275E+02	1.33554439E+01
9.02868550E+02	1.33294057E+01
9.67144758E+02	1.38462952E+01
9.71447584E+02	1.40631398E+01
1.00143428E+03	1.27888226E+01
1.00286855E+03	1.28366602E+01
1.00717238E+03	1.31388310E+01
1.00717338E+03	1.31384024E+01
1.00717438E+03	1.31379890E+01
1.00717538E+03	1.31375691E+01
1.00717638E+03	1.31371383E+01
1.00717738E+03	1.31366971E+01
1.00717838E+03	1.31362464E+01
1.00717938E+03	1.31357870E+01
1.00718038E+03	1.31353217E+01
1.00718138E+03	1.31348524E+01
1.00719138E+03	1.31302427E+01
1.00720138E+03	1.31262601E+01
1.00721138E+03	1.31229598E+01
1.00722138E+03	1.31201304E+01
1.00723138E+03	1.31175737E+01
1.00724138E+03	1.31151641E+01
1.00725138E+03	1.31128376E+01
1.00726138E+03	1.31105737E+01

1.00727138E+03	1.31083750E+01
1.00737138E+03	1.30919493E+01
1.00747138E+03	1.30797269E+01
1.00757138E+03	1.30629127E+01
1.00767138E+03	1.30490004E+01
1.00777138E+03	1.30417242E+01
1.00787138E+03	1.30398160E+01
1.00797138E+03	1.30419319E+01
1.00807138E+03	1.30465424E+01
1.01317138E+03	1.28053316E+01
1.01417138E+03	1.26672649E+01
1.01517138E+03	1.25428221E+01
1.01617138E+03	1.24530072E+01
1.01717138E+03	1.23640844E+01
1.02717138E+03	1.22434841E+01
1.03717138E+03	1.22626137E+01
1.04717138E+03	1.20330309E+01
1.05717138E+03	1.20392465E+01
1.06714476E+03	1.16666281E+01
1.06717138E+03	1.16982377E+01
1.07144758E+03	1.13764652E+01
1.07717138E+03	1.12039012E+01
1.08717138E+03	1.10748196E+01
1.09717138E+03	1.09915434E+01
1.10143428E+03	1.10242255E+01
1.10286855E+03	1.10487882E+01
1.10717138E+03	1.10510739E+01
1.16714476E+03	1.07614325E+01
1.17144758E+03	1.06148015E+01
1.20143428E+03	1.03597391E+01
1.20286855E+03	1.03552553E+01
1.20717138E+03	1.03550931E+01
1.26714476E+03	1.02568241E+01
1.27144758E+03	1.01505475E+01
1.30143428E+03	1.01494153E+01
1.30286855E+03	1.01785351E+01
1.30717138E+03	1.01183509E+01
1.36714476E+03	9.93124440E+00
1.37144758E+03	9.79889629E+00
1.40143428E+03	1.01429115E+01
1.40286855E+03	1.00796940E+01
1.40717138E+03	1.01130007E+01
1.46714476E+03	9.77218789E+00
1.47144758E+03	9.83541536E+00
1.50143428E+03	9.73294902E+00
1.50286855E+03	9.71107477E+00
1.50717138E+03	9.62876251E+00
1.56714476E+03	9.68904018E+00
1.57144758E+03	9.70564365E+00
1.60143428E+03	9.90089532E+00

1.60286855E+03 9.90549915E+00
1.60717138E+03 9.89798686E+00
1.66714476E+03 9.75865914E+00
1.67144758E+03 9.68335881E+00
1.70143428E+03 9.65892170E+00
1.70286855E+03 9.68285715E+00
1.70717138E+03 9.75317121E+00
1.76714476E+03 9.91503401E+00
1.77144758E+03 9.96169050E+00
1.80143428E+03 9.84402761E+00
1.80286855E+03 9.84133509E+00
1.80717138E+03 9.82856060E+00
1.86714476E+03 9.88713024E+00
1.87144758E+03 9.82566097E+00
1.90143428E+03 9.87154513E+00
1.90286855E+03 9.86130317E+00
1.90717138E+03 9.85307090E+00
1.96714476E+03 9.60397344E+00
1.97144758E+03 9.57853907E+00
2.00143428E+03 9.63308678E+00
2.00286855E+03 9.63186909E+00
2.00717138E+03 9.63743463E+00
2.06714476E+03 9.70549724E+00
2.07144758E+03 9.68581887E+00
2.10143428E+03 9.47018738E+00
2.10286855E+03 9.46230835E+00
2.10717138E+03 9.43854243E+00
2.16714476E+03 9.43489762E+00
2.17144758E+03 9.41201944E+00
2.20143428E+03 9.45340275E+00
2.20286855E+03 9.44604993E+00
2.20717138E+03 9.49805597E+00
2.26714476E+03 9.49766705E+00
2.27144758E+03 9.46482894E+00
2.30143428E+03 9.44344242E+00
2.30286855E+03 9.44735453E+00
2.30717138E+03 9.45828902E+00
2.36714476E+03 9.50312322E+00
2.37144758E+03 9.51944247E+00
2.40143428E+03 9.54052412E+00
2.40286855E+03 9.54851652E+00
2.40717138E+03 9.57380096E+00
2.46714476E+03 9.46964274E+00
2.47144758E+03 9.42946637E+00
2.50143428E+03 9.44562887E+00
2.50286855E+03 9.44634397E+00
2.50717138E+03 9.44387803E+00
2.56714476E+03 9.37746474E+00
2.57144758E+03 9.36069587E+00
2.60143428E+03 9.37187132E+00

2.60286855E+03 9.37269431E+00
2.60717138E+03 9.37076750E+00
2.66714476E+03 9.34479855E+00
2.67144758E+03 9.31079191E+00
2.70143428E+03 9.23645626E+00
2.70286855E+03 9.23343378E+00
2.70717138E+03 9.22494296E+00
2.76714476E+03 9.23432137E+00
2.77144758E+03 9.22072062E+00
2.80143428E+03 9.21976171E+00
2.80286855E+03 9.22390378E+00
2.80717138E+03 9.22933733E+00
2.86714476E+03 9.15987156E+00
2.87144758E+03 9.14988574E+00
2.90143428E+03 9.11668640E+00
2.90286855E+03 9.11422868E+00
2.90717138E+03 9.10777422E+00
2.96714476E+03 9.06846174E+00
2.97144758E+03 9.05416965E+00
3.00143428E+03 9.07833420E+00
3.00286855E+03 9.07972511E+00
3.00717138E+03 9.07878015E+00
3.07144758E+03 9.00682198E+00
3.10143428E+03 9.08730528E+00
3.10286855E+03 9.08767697E+00
3.10717138E+03 9.08497167E+00
3.17144758E+03 8.98442484E+00
3.20286855E+03 8.98067187E+00
3.20717138E+03 8.97941125E+00
3.27144758E+03 8.97324453E+00
3.30286855E+03 9.01359954E+00
3.30717138E+03 9.03696000E+00
3.37144758E+03 9.03322624E+00
3.40717138E+03 8.97574789E+00
3.47144758E+03 9.05482983E+00
3.50717138E+03 9.05825927E+00
3.57144758E+03 9.07438775E+00
3.60717138E+03 9.07314522E+00
3.70717138E+03 9.14735822E+00
3.80717138E+03 9.08788115E+00
3.90717138E+03 9.19376403E+00

#Tot_ener(cm-1) 3--->5 Cross_sec(angs^2)
1.00717238E+03 1.60580019E-03
1.00717338E+03 2.50845736E-03
1.00717438E+03 3.37097801E-03
1.00717538E+03 4.23752772E-03
1.00717638E+03 5.11890014E-03
1.00717738E+03 6.01702729E-03
1.00717838E+03 6.93072949E-03

1.00717938E+03 7.85764821E-03
1.00718038E+03 8.79502863E-03
1.00718138E+03 9.74007384E-03
1.00719138E+03 1.91396418E-02
1.00720138E+03 2.77333165E-02
1.00721138E+03 3.54156621E-02
1.00722138E+03 4.24650186E-02
1.00723138E+03 4.91431938E-02
1.00724138E+03 5.56151029E-02
1.00725138E+03 6.19599538E-02
1.00726138E+03 6.81995078E-02
1.00727138E+03 7.43231954E-02
1.00737138E+03 1.26281239E-01
1.00747138E+03 1.76369597E-01
1.00757138E+03 2.24885072E-01
1.00767138E+03 2.58166512E-01
1.00777138E+03 2.86219426E-01
1.00787138E+03 3.14383581E-01
1.00797138E+03 3.42209314E-01
1.00807138E+03 3.67888063E-01
1.01317138E+03 1.22688622E+00
1.01417138E+03 1.42042740E+00
1.01517138E+03 1.49822324E+00
1.01617138E+03 1.57126410E+00
1.01717138E+03 1.67085074E+00
1.02717138E+03 2.55008580E+00
1.03717138E+03 3.12053260E+00
1.04717138E+03 3.68362675E+00
1.05717138E+03 4.13042723E+00
1.06714476E+03 4.67861581E+00
1.06717138E+03 4.68458684E+00
1.07144758E+03 4.78741139E+00
1.07717138E+03 5.06116861E+00
1.08717138E+03 5.36742512E+00
1.09717138E+03 5.62375045E+00
1.10143428E+03 5.84078266E+00
1.10286855E+03 5.91871172E+00
1.10717138E+03 6.05214597E+00
1.16714476E+03 7.28942509E+00
1.17144758E+03 7.42509837E+00
1.20143428E+03 7.54108059E+00
1.20286855E+03 7.59330920E+00
1.20717138E+03 7.69821922E+00
1.26714476E+03 8.15477290E+00
1.27144758E+03 8.11870058E+00
1.30143428E+03 8.12359791E+00
1.30286855E+03 8.15187739E+00
1.30717138E+03 8.22043673E+00
1.36714476E+03 8.26758313E+00
1.37144758E+03 8.25883413E+00

1.40143428E+03	8.39430632E+00
1.40286855E+03	8.38417810E+00
1.40717138E+03	8.25994737E+00
1.46714476E+03	7.83323029E+00
1.47144758E+03	7.76292105E+00
1.50143428E+03	7.71005737E+00
1.50286855E+03	7.70860584E+00
1.50717138E+03	7.71217211E+00
1.56714476E+03	7.41643802E+00
1.57144758E+03	7.39130574E+00
1.60143428E+03	7.02638507E+00
1.60286855E+03	7.01147219E+00
1.60717138E+03	6.98530478E+00
1.66714476E+03	7.03802712E+00
1.67144758E+03	6.98622200E+00
1.70143428E+03	6.71613816E+00
1.70286855E+03	6.70548476E+00
1.70717138E+03	6.66749488E+00
1.76714476E+03	6.57523739E+00
1.77144758E+03	6.54679148E+00
1.80143428E+03	6.52349974E+00
1.80286855E+03	6.51495970E+00
1.80717138E+03	6.50433868E+00
1.86714476E+03	6.40791909E+00
1.87144758E+03	6.39890866E+00
1.90143428E+03	6.16801157E+00
1.90286855E+03	6.16891348E+00
1.90717138E+03	6.16962427E+00
1.96714476E+03	6.00577636E+00
1.97144758E+03	5.97719397E+00
2.00143428E+03	5.83036823E+00
2.00286855E+03	5.83653412E+00
2.00717138E+03	5.84341760E+00
2.06714476E+03	5.70403839E+00
2.07144758E+03	5.68380634E+00
2.10143428E+03	5.66877517E+00
2.10286855E+03	5.67152331E+00
2.10717138E+03	5.68049059E+00
2.16714476E+03	5.55476249E+00
2.17144758E+03	5.53402540E+00
2.20143428E+03	5.55653925E+00
2.20286855E+03	5.52076148E+00
2.20717138E+03	5.45945583E+00
2.26714476E+03	5.51703478E+00
2.27144758E+03	5.49912605E+00
2.30143428E+03	5.46120528E+00
2.30286855E+03	5.45739179E+00
2.30717138E+03	5.44591493E+00
2.36714476E+03	5.51346742E+00
2.37144758E+03	5.50055581E+00

2.40143428E+03 5.39600439E+00
2.40286855E+03 5.40501076E+00
2.40717138E+03 5.41553204E+00
2.46714476E+03 5.39826418E+00
2.47144758E+03 5.36229391E+00
2.50143428E+03 5.34773495E+00
2.50286855E+03 5.34423811E+00
2.50717138E+03 5.33156193E+00
2.56714476E+03 5.20365558E+00
2.57144758E+03 5.18160896E+00
2.60143428E+03 5.20982876E+00
2.60286855E+03 5.20919096E+00
2.60717138E+03 5.21177893E+00
2.66714476E+03 5.31419751E+00
2.67144758E+03 5.31757631E+00
2.70143428E+03 5.29281945E+00
2.70286855E+03 5.29170210E+00
2.70717138E+03 5.28585019E+00
2.76714476E+03 5.13670513E+00
2.77144758E+03 5.13095755E+00
2.80143428E+03 5.17608098E+00
2.80286855E+03 5.18167069E+00
2.80717138E+03 5.19744956E+00
2.86714476E+03 5.22904864E+00
2.87144758E+03 5.24049895E+00
2.90143428E+03 5.26149271E+00
2.90286855E+03 5.26044185E+00
2.90717138E+03 5.25789641E+00
2.96714476E+03 5.19387047E+00
2.97144758E+03 5.21034770E+00
3.00143428E+03 5.22976532E+00
3.00286855E+03 5.23152182E+00
3.00717138E+03 5.23552377E+00
3.07144758E+03 5.21313255E+00
3.10143428E+03 5.18777480E+00
3.10286855E+03 5.18799897E+00
3.10717138E+03 5.18760419E+00
3.17144758E+03 5.15100879E+00
3.20286855E+03 5.14036839E+00
3.20717138E+03 5.13578594E+00
3.27144758E+03 5.13867407E+00
3.30286855E+03 5.06226886E+00
3.30717138E+03 5.05868311E+00
3.37144758E+03 5.05136670E+00
3.40717138E+03 5.00507602E+00
3.47144758E+03 5.00047495E+00
3.50717138E+03 4.97448150E+00
3.57144758E+03 4.98858964E+00
3.60717138E+03 4.95882974E+00
3.70717138E+03 4.90055198E+00

3.80717138E+03 4.85751513E+00
3.90717138E+03 4.79848664E+00

#Tot_ener(cm-1) 4--->5 Cross_sec(angs^2)
1.00717238E+03 4.80604933E-03
1.00717338E+03 7.59370355E-03
1.00717438E+03 1.03039569E-02
1.00717538E+03 1.30600524E-02
1.00717638E+03 1.58886281E-02
1.00717738E+03 1.87909169E-02
1.00717838E+03 2.17593855E-02
1.00717938E+03 2.47833124E-02
1.00718038E+03 2.78510701E-02
1.00718138E+03 3.09511559E-02
1.00719138E+03 6.18038904E-02
1.00720138E+03 8.94353023E-02
1.00721138E+03 1.13361002E-01
1.00722138E+03 1.34710009E-01
1.00723138E+03 1.54578133E-01
1.00724138E+03 1.73694803E-01
1.00725138E+03 1.92462231E-01
1.00726138E+03 2.11060489E-01
1.00727138E+03 2.29539556E-01
1.00737138E+03 4.03281662E-01
1.00747138E+03 5.75022602E-01
1.00757138E+03 7.38722158E-01
1.00767138E+03 8.61493722E-01
1.00777138E+03 9.59362673E-01
1.00787138E+03 1.05352451E+00
1.00797138E+03 1.14676983E+00
1.00807138E+03 1.23400177E+00
1.01317138E+03 3.02875271E+00
1.01417138E+03 3.30884161E+00
1.01517138E+03 3.40442118E+00
1.01617138E+03 3.52068863E+00
1.01717138E+03 3.67230387E+00
1.02717138E+03 5.08159745E+00
1.03717138E+03 6.17679529E+00
1.04717138E+03 6.94634257E+00
1.05717138E+03 7.66257056E+00
1.06714476E+03 7.74365812E+00
1.06717138E+03 7.73438809E+00
1.07144758E+03 7.79267652E+00
1.07717138E+03 8.07873570E+00
1.08717138E+03 8.68816896E+00
1.09717138E+03 8.91131430E+00
1.10143428E+03 9.18123406E+00
1.10286855E+03 9.25506681E+00
1.10717138E+03 9.57555209E+00
1.16714476E+03 1.13392584E+01

1.17144758E+03	1.12606268E+01
1.20143428E+03	1.09038767E+01
1.20286855E+03	1.09385729E+01
1.20717138E+03	1.10156172E+01
1.26714476E+03	1.10921266E+01
1.27144758E+03	1.10719625E+01
1.30143428E+03	1.20498758E+01
1.30286855E+03	1.20753048E+01
1.30717138E+03	1.20158478E+01
1.36714476E+03	1.22271926E+01
1.37144758E+03	1.22270046E+01
1.40143428E+03	1.15139496E+01
1.40286855E+03	1.15997950E+01
1.40717138E+03	1.16227051E+01
1.46714476E+03	1.06357118E+01
1.47144758E+03	1.06276036E+01
1.50143428E+03	1.04016888E+01
1.50286855E+03	1.03887374E+01
1.50717138E+03	1.03697506E+01
1.56714476E+03	9.70773136E+00
1.57144758E+03	9.63943459E+00
1.60143428E+03	9.53869901E+00
1.60286855E+03	9.52190229E+00
1.60717138E+03	9.48151627E+00
1.66714476E+03	9.33175129E+00
1.67144758E+03	9.26688610E+00
1.70143428E+03	9.12777375E+00
1.70286855E+03	9.13710723E+00
1.70717138E+03	9.16427048E+00
1.76714476E+03	9.03664914E+00
1.77144758E+03	9.07695144E+00
1.80143428E+03	9.04373472E+00
1.80286855E+03	9.04837200E+00
1.80717138E+03	9.03114823E+00
1.86714476E+03	8.94735295E+00
1.87144758E+03	8.91591547E+00
1.90286855E+03	8.76006902E+00
1.90717138E+03	8.75841112E+00
1.96714476E+03	8.54990558E+00
1.97144758E+03	8.52472587E+00
2.00286855E+03	8.53487091E+00
2.00717138E+03	8.50919163E+00
2.06714476E+03	8.58094150E+00
2.07144758E+03	8.58283656E+00
2.10143428E+03	8.45658496E+00
2.10286855E+03	8.45374390E+00
2.10717138E+03	8.44997285E+00
2.16714476E+03	8.49563214E+00
2.17144758E+03	8.53215127E+00
2.20143428E+03	8.44563691E+00

2.20286855E+03	8.45363118E+00
2.20717138E+03	8.46237172E+00
2.26714476E+03	8.54670621E+00
2.27144758E+03	8.55490744E+00
2.30286855E+03	8.47116565E+00
2.30717138E+03	8.47490982E+00
2.36714476E+03	8.45187568E+00
2.37144758E+03	8.46891283E+00
2.40143428E+03	8.62113455E+00
2.40286855E+03	8.62142965E+00
2.40717138E+03	8.60883674E+00
2.46714476E+03	8.46315473E+00
2.47144758E+03	8.47081650E+00
2.50143428E+03	8.50927951E+00
2.50286855E+03	8.50443780E+00
2.50717138E+03	8.48491801E+00
2.56714476E+03	8.41225329E+00
2.57144758E+03	8.44110261E+00
2.60143428E+03	8.43233396E+00
2.60286855E+03	8.43518140E+00
2.60717138E+03	8.44226992E+00
2.66714476E+03	8.41080568E+00
2.67144758E+03	8.44349073E+00
2.70143428E+03	8.43099729E+00
2.70286855E+03	8.42885403E+00
2.70717138E+03	8.42496134E+00
2.76714476E+03	8.34761147E+00
2.77144758E+03	8.36992630E+00
2.80143428E+03	8.35193887E+00
2.80286855E+03	8.35592655E+00
2.80717138E+03	8.36757991E+00
2.86714476E+03	8.32478135E+00
2.87144758E+03	8.33971338E+00
2.90143428E+03	8.35876190E+00
2.90286855E+03	8.36169819E+00
2.90717138E+03	8.36439471E+00
2.96714476E+03	8.36189366E+00
2.97144758E+03	8.35730960E+00
3.00143428E+03	8.34670936E+00
3.00286855E+03	8.34641738E+00
3.00717138E+03	8.34678174E+00
3.07144758E+03	8.28205810E+00
3.10143428E+03	8.31022395E+00
3.10286855E+03	8.31027676E+00
3.10717138E+03	8.31095794E+00
3.17144758E+03	8.22576137E+00
3.20286855E+03	8.19143458E+00
3.20717138E+03	8.19611923E+00
3.27144758E+03	8.22916927E+00
3.30286855E+03	8.23240222E+00

3.30717138E+03 8.22969264E+00
3.37144758E+03 8.11972249E+00
3.40717138E+03 8.10172705E+00
3.47144758E+03 8.15206366E+00
3.50717138E+03 8.15090297E+00
3.57144758E+03 8.13027212E+00
3.60717138E+03 8.07524646E+00
3.70717138E+03 8.06127179E+00
3.80717138E+03 7.99890001E+00
3.90717138E+03 7.98405104E+00

#Tot_ener(cm-1) 1--->0 Cross_sec(angs^2)
6.71457584E+01 5.73729367E+03
6.71467584E+01 3.89003293E+03
6.71477584E+01 3.07610959E+03
6.71487584E+01 2.59428489E+03
6.71497584E+01 2.26806525E+03
6.71507584E+01 2.02941189E+03
6.71517584E+01 1.84570703E+03
6.71527584E+01 1.69948682E+03
6.71537584E+01 1.57930876E+03
6.71547584E+01 1.47882667E+03
6.71647584E+01 9.71752907E+02
6.71747584E+01 7.95690553E+02
6.71847584E+01 7.29544507E+02
6.71947584E+01 7.14857528E+02
6.72047584E+01 7.24746899E+02
6.72147584E+01 7.42628372E+02
6.72247584E+01 7.57777394E+02
6.72347584E+01 7.64358582E+02
6.72447584E+01 7.60562494E+02
6.73447584E+01 5.18700328E+02
6.74447584E+01 3.85516433E+02
6.75447584E+01 3.32640700E+02
6.76447584E+01 3.09175073E+02
6.77447584E+01 2.99789026E+02
6.78447584E+01 2.94652470E+02
6.79447584E+01 2.88270841E+02
6.80447584E+01 2.79792581E+02
7.31447584E+01 9.02689698E+01
7.41447584E+01 8.46486076E+01
7.51447584E+01 8.15115808E+01
7.61447584E+01 8.20139852E+01
7.71447584E+01 9.55974906E+01
8.71447584E+01 4.59562373E+01
9.71447584E+01 3.36961528E+01
1.07144758E+02 3.61954415E+01
1.17144758E+02 2.91916437E+01
1.27144758E+02 2.01835024E+01
1.37144758E+02 1.70009048E+01

1.47144758E+02	2.54019114E+01
1.57144758E+02	2.00342476E+01
1.67144758E+02	1.86569208E+01
2.01435275E+02	1.88449624E+01
2.01436275E+02	1.88498213E+01
2.01437275E+02	1.88549628E+01
2.01438275E+02	1.88601701E+01
2.01439275E+02	1.88653672E+01
2.01440275E+02	1.88705353E+01
2.01441275E+02	1.88756610E+01
2.01442275E+02	1.88807361E+01
2.01443275E+02	1.88857586E+01
2.01444275E+02	1.88907295E+01
2.01454275E+02	1.89384005E+01
2.01464275E+02	1.89837425E+01
2.01474275E+02	1.90275720E+01
2.01484275E+02	1.90698376E+01
2.01494275E+02	1.91102364E+01
2.01504275E+02	1.91485736E+01
2.01514275E+02	1.91848748E+01
2.01524275E+02	1.92192859E+01
2.01534275E+02	1.92520848E+01
2.01634275E+02	1.95292159E+01
2.01734275E+02	1.95188469E+01
2.01834275E+02	1.90775934E+01
2.01934275E+02	1.88831316E+01
2.02034275E+02	1.88017491E+01
2.02134275E+02	1.87780567E+01
2.02234275E+02	1.87460701E+01
2.02334275E+02	1.86831514E+01
2.07434275E+02	1.38562503E+01
2.08434275E+02	1.35281125E+01
2.09434275E+02	1.38584250E+01
2.10434275E+02	1.30382848E+01
2.11434275E+02	1.31561038E+01
2.21434275E+02	1.49276500E+01
2.31434275E+02	1.03357494E+01
2.41434275E+02	9.98102173E+00
2.51434275E+02	1.16302301E+01
2.61434275E+02	1.07667439E+01
2.67144758E+02	1.03622536E+01
2.71434275E+02	9.72578286E+00
2.81434275E+02	9.51500492E+00
2.91434275E+02	1.02565747E+01
3.01434275E+02	1.03127368E+01
3.67144758E+02	1.27745043E+01
4.01434275E+02	1.09045309E+01
4.02869550E+02	1.09225694E+01
4.02870550E+02	1.09207842E+01
4.02871550E+02	1.09196077E+01

4.02872550E+02 1.09187133E+01
4.02873550E+02 1.09179702E+01
4.02874550E+02 1.09173275E+01
4.02875550E+02 1.09167400E+01
4.02876550E+02 1.09162000E+01
4.02877550E+02 1.09156847E+01
4.02878550E+02 1.09151810E+01
4.02888550E+02 1.09101439E+01
4.02898550E+02 1.09039373E+01
4.02908550E+02 1.08965215E+01
4.02918550E+02 1.08885107E+01
4.02928550E+02 1.08806502E+01
4.02938550E+02 1.08735909E+01
4.02948550E+02 1.08677049E+01
4.02958550E+02 1.08631257E+01
4.02968550E+02 1.08597835E+01
4.03068550E+02 1.08389465E+01
4.03168550E+02 1.07631364E+01
4.03268550E+02 1.07515952E+01
4.03368550E+02 1.07735350E+01
4.03468550E+02 1.08543848E+01
4.03568550E+02 1.09002828E+01
4.03668550E+02 1.09151711E+01
4.03768550E+02 1.09323060E+01
4.08868550E+02 1.17751874E+01
4.09868550E+02 1.18097189E+01
4.10868550E+02 1.15484509E+01
4.11868550E+02 1.13360108E+01
4.12868550E+02 1.14360175E+01
4.22868550E+02 1.05843493E+01
4.32868550E+02 1.00600677E+01
4.42868550E+02 1.04913631E+01
4.52868550E+02 9.40681562E+00
4.62868550E+02 8.84055338E+00
4.67144758E+02 8.97855028E+00
4.72868550E+02 9.73889848E+00
4.82868550E+02 1.01273464E+01
4.92868550E+02 1.02176512E+01
5.01434275E+02 1.00136532E+01
5.02868550E+02 1.01344818E+01
5.67144758E+02 1.01405547E+01
6.01434275E+02 1.03126256E+01
6.02868550E+02 1.01745593E+01
6.67144758E+02 9.85892618E+00
6.71448584E+02 1.01368792E+01
6.71449584E+02 1.01366453E+01
6.71450584E+02 1.01363954E+01
6.71451584E+02 1.01361512E+01
6.71452584E+02 1.01359098E+01
6.71453584E+02 1.01356719E+01

6.71454584E+02	1.01354381E+01
6.71455584E+02	1.01352114E+01
6.71456584E+02	1.01349926E+01
6.71457584E+02	1.01347715E+01
6.71467584E+02	1.01325403E+01
6.71477584E+02	1.01299741E+01
6.71487584E+02	1.01268898E+01
6.71497584E+02	1.01233099E+01
6.71507584E+02	1.01193095E+01
6.71517584E+02	1.01149742E+01
6.71527584E+02	1.01103764E+01
6.71537584E+02	1.01055608E+01
6.71547584E+02	1.01005755E+01
6.71647584E+02	1.00468622E+01
6.71747584E+02	9.99414680E+00
6.71847584E+02	9.94146787E+00
6.71947584E+02	9.88326805E+00
6.72047584E+02	9.81968521E+00
6.72147584E+02	9.75267410E+00
6.72247584E+02	9.68517975E+00
6.72347584E+02	9.62131354E+00
6.77447584E+02	9.87245450E+00
6.78447584E+02	1.01740557E+01
6.79447584E+02	1.00319439E+01
6.80447584E+02	9.69250839E+00
6.81447584E+02	9.62612981E+00
6.91447584E+02	9.66470307E+00
7.01434275E+02	8.89997777E+00
7.01447584E+02	8.89915163E+00
7.02868550E+02	8.79227926E+00
7.11447584E+02	8.59094091E+00
7.21447584E+02	9.45931512E+00
7.31447584E+02	1.01024014E+01
7.41447584E+02	9.91769521E+00
7.51447584E+02	8.99780365E+00
7.61447584E+02	9.27909939E+00
7.67144758E+02	9.10873416E+00
7.71447584E+02	9.18954229E+00
8.01434275E+02	9.85086097E+00
8.02868550E+02	9.61326060E+00
8.67144758E+02	9.27420168E+00
8.71447584E+02	9.57764133E+00
9.01434275E+02	1.05325939E+01
9.02868550E+02	1.06745805E+01
9.67144758E+02	1.00937495E+01
9.71447584E+02	1.02355462E+01
1.00143428E+03	9.90263281E+00
1.00286855E+03	9.81819604E+00
1.00717238E+03	9.75155699E+00
1.00717338E+03	9.75196118E+00

1.00717438E+03	9.75235449E+00
1.00717538E+03	9.75274286E+00
1.00717638E+03	9.75313086E+00
1.00717738E+03	9.75351804E+00
1.00717838E+03	9.75390485E+00
1.00717938E+03	9.75429108E+00
1.00718038E+03	9.75467705E+00
1.00718138E+03	9.75506172E+00
1.00719138E+03	9.75882284E+00
1.00720138E+03	9.76234642E+00
1.00721138E+03	9.76561253E+00
1.00722138E+03	9.76865361E+00
1.00723138E+03	9.77149374E+00
1.00724138E+03	9.77416166E+00
1.00725138E+03	9.77667825E+00
1.00726138E+03	9.77906836E+00
1.00727138E+03	9.78135540E+00
1.00737138E+03	9.80183525E+00
1.00747138E+03	9.82173728E+00
1.00757138E+03	9.84311661E+00
1.00767138E+03	9.86513061E+00
1.00777138E+03	9.88524080E+00
1.00787138E+03	9.90257848E+00
1.00797138E+03	9.91773503E+00
1.00807138E+03	9.93195481E+00
1.01317138E+03	1.04355603E+01
1.01417138E+03	1.04317368E+01
1.01517138E+03	1.04191484E+01
1.01617138E+03	1.03901569E+01
1.01717138E+03	1.03541028E+01
1.02717138E+03	1.00637837E+01
1.03717138E+03	9.67507484E+00
1.04717138E+03	9.31469875E+00
1.05717138E+03	9.09210588E+00
1.06714476E+03	9.24001850E+00
1.06717138E+03	9.24548076E+00
1.07144758E+03	9.08587843E+00
1.07717138E+03	9.32748370E+00
1.08717138E+03	9.41358405E+00
1.09717138E+03	9.20931707E+00
1.10143428E+03	9.23675699E+00
1.10286855E+03	9.21433701E+00
1.10717138E+03	9.13913316E+00
1.16714476E+03	8.65456242E+00
1.17144758E+03	8.45236576E+00
1.20143428E+03	7.73232275E+00
1.20286855E+03	7.76129529E+00
1.20717138E+03	7.74713608E+00
1.26714476E+03	7.99909165E+00
1.27144758E+03	7.55819850E+00

1.30143428E+03	8.06953029E+00
1.30286855E+03	8.00948427E+00
1.30717138E+03	8.13995791E+00
1.36714476E+03	7.34685176E+00
1.37144758E+03	7.37454216E+00
1.40143428E+03	7.22441152E+00
1.40286855E+03	7.22251513E+00
1.40717138E+03	7.10958669E+00
1.46714476E+03	6.26253958E+00
1.47144758E+03	6.05434695E+00
1.50143428E+03	6.49196933E+00
1.50286855E+03	6.51446162E+00
1.50717138E+03	6.54979555E+00
1.56714476E+03	6.25452870E+00
1.57144758E+03	6.25186487E+00
1.60143428E+03	6.14521226E+00
1.60286855E+03	6.14449342E+00
1.60717138E+03	6.15342409E+00
1.66714476E+03	6.05351163E+00
1.67144758E+03	5.96275453E+00
1.70143428E+03	6.20238847E+00
1.70286855E+03	6.18062388E+00
1.70717138E+03	6.17377267E+00
1.76714476E+03	6.34727110E+00
1.77144758E+03	6.34374159E+00
1.80143428E+03	6.46945979E+00
1.80286855E+03	6.50882438E+00
1.80717138E+03	6.21296471E+00
1.86714476E+03	6.48905113E+00
1.87144758E+03	6.50914746E+00
1.90143428E+03	6.54463475E+00
1.90286855E+03	6.54719481E+00
1.90717138E+03	6.55238578E+00
1.96714476E+03	6.22658769E+00
1.97144758E+03	6.15905047E+00
2.00143428E+03	6.54639425E+00
2.00286855E+03	6.56914512E+00
2.00717138E+03	6.61007730E+00
2.06714476E+03	6.62128932E+00
2.07144758E+03	6.58984338E+00
2.10143428E+03	6.55705639E+00
2.10286855E+03	6.54572932E+00
2.10717138E+03	6.51412184E+00
2.16714476E+03	6.57678135E+00
2.17144758E+03	6.61992713E+00
2.20143428E+03	6.67561502E+00
2.20286855E+03	6.54316680E+00
2.20717138E+03	6.70339313E+00
2.26714476E+03	6.48912437E+00
2.27144758E+03	6.58802397E+00

2.30143428E+03 6.70049559E+00
2.30286855E+03 6.70103301E+00
2.30717138E+03 6.70377802E+00
2.36714476E+03 6.56069802E+00
2.37144758E+03 6.54308096E+00
2.40143428E+03 6.61719383E+00
2.40286855E+03 6.60652565E+00
2.40717138E+03 6.57212686E+00
2.46714476E+03 6.83913059E+00
2.47144758E+03 6.77045113E+00
2.50143428E+03 6.86032074E+00
2.50286855E+03 6.85589585E+00
2.50717138E+03 6.83951415E+00
2.56714476E+03 6.68669939E+00
2.57144758E+03 6.67510913E+00
2.60143428E+03 6.53230459E+00
2.60286855E+03 6.52879741E+00
2.60717138E+03 6.51427960E+00
2.66714476E+03 6.82984756E+00
2.67144758E+03 6.81642732E+00
2.70143428E+03 6.65576433E+00
2.70286855E+03 6.64462723E+00
2.70717138E+03 6.60491894E+00
2.76714476E+03 6.38713448E+00
2.77144758E+03 6.45953542E+00
2.80143428E+03 6.33120220E+00
2.80286855E+03 6.31697575E+00
2.80717138E+03 6.33692059E+00
2.86714476E+03 6.56448890E+00
2.87144758E+03 6.55345878E+00
2.90143428E+03 6.44399288E+00
2.90286855E+03 6.43728456E+00
2.90717138E+03 6.41588383E+00
2.96714476E+03 6.25281652E+00
2.97144758E+03 6.26143463E+00
3.00143428E+03 6.30965945E+00
3.00286855E+03 6.30082140E+00
3.00717138E+03 6.27358695E+00
3.07144758E+03 6.21972413E+00
3.10143428E+03 6.11985894E+00
3.10286855E+03 6.11405055E+00
3.10717138E+03 6.09496777E+00
3.17144758E+03 5.95536475E+00
3.20286855E+03 6.16617281E+00
3.20717138E+03 6.14621816E+00
3.27144758E+03 5.93361522E+00
3.30286855E+03 5.81130973E+00
3.30717138E+03 5.79921332E+00
3.37144758E+03 5.79145394E+00
3.40717138E+03 5.72982326E+00

3.47144758E+03 5.71507831E+00
3.50717138E+03 5.58486693E+00
3.57144758E+03 5.47712720E+00
3.60717138E+03 5.60314820E+00
3.70717138E+03 5.41088357E+00
3.80717138E+03 5.29730663E+00
3.90717138E+03 5.30578060E+00

#Tot_ener(cm-1) 2--->0 Cross_sec(angs^2)
2.01435275E+02 4.64405961E+03
2.01436275E+02 3.09879228E+03
2.01437275E+02 2.43155914E+03
2.01438275E+02 2.04393323E+03
2.01439275E+02 1.78601165E+03
2.01440275E+02 1.60030749E+03
2.01441275E+02 1.45937820E+03
2.01442275E+02 1.34836484E+03
2.01443275E+02 1.25840045E+03
2.01444275E+02 1.18374031E+03
2.01454275E+02 8.10717537E+02
2.01464275E+02 6.64982739E+02
2.01474275E+02 5.84872105E+02
2.01484275E+02 5.33917009E+02
2.01494275E+02 4.98762579E+02
2.01504275E+02 4.72981802E+02
2.01514275E+02 4.52989391E+02
2.01524275E+02 4.36668664E+02
2.01534275E+02 4.22727439E+02
2.01634275E+02 3.51615954E+02
2.01734275E+02 3.60959996E+02
2.01834275E+02 3.10122836E+02
2.01934275E+02 2.67053912E+02
2.02034275E+02 2.36189819E+02
2.02134275E+02 2.11907829E+02
2.02234275E+02 1.95363893E+02
2.02334275E+02 1.83777709E+02
2.07434275E+02 6.70159871E+01
2.08434275E+02 5.76211647E+01
2.09434275E+02 5.05649581E+01
2.10434275E+02 4.81151499E+01
2.11434275E+02 4.49548650E+01
2.21434275E+02 3.12231509E+01
2.31434275E+02 2.27555218E+01
2.41434275E+02 2.08200767E+01
2.51434275E+02 1.84270422E+01
2.61434275E+02 1.57834021E+01
2.67144758E+02 1.63739416E+01
2.71434275E+02 1.59527879E+01
2.81434275E+02 1.26312286E+01
2.91434275E+02 1.10244919E+01

3.01434275E+02	1.15375411E+01
3.67144758E+02	8.38831179E+00
4.01434275E+02	7.73470502E+00
4.02869550E+02	7.92817497E+00
4.02870550E+02	7.92942626E+00
4.02871550E+02	7.93061103E+00
4.02872550E+02	7.93175572E+00
4.02873550E+02	7.93285359E+00
4.02874550E+02	7.93391593E+00
4.02875550E+02	7.93494434E+00
4.02876550E+02	7.93594080E+00
4.02877550E+02	7.93690556E+00
4.02878550E+02	7.93784464E+00
4.02888550E+02	7.94602503E+00
4.02898550E+02	7.95246027E+00
4.02908550E+02	7.95722327E+00
4.02918550E+02	7.96009325E+00
4.02928550E+02	7.96096246E+00
4.02938550E+02	7.95996148E+00
4.02948550E+02	7.95743144E+00
4.02958550E+02	7.95380153E+00
4.02968550E+02	7.94948266E+00
4.03068550E+02	7.89236555E+00
4.03168550E+02	7.81645423E+00
4.03268550E+02	7.75093464E+00
4.03368550E+02	7.68408863E+00
4.03468550E+02	7.68445078E+00
4.03568550E+02	7.67749447E+00
4.03668550E+02	7.64769533E+00
4.03768550E+02	7.60864637E+00
4.08868550E+02	6.42711534E+00
4.09868550E+02	6.48396576E+00
4.10868550E+02	6.75099426E+00
4.11868550E+02	6.96037842E+00
4.12868550E+02	6.91822223E+00
4.22868550E+02	7.15185392E+00
4.32868550E+02	6.49604276E+00
4.42868550E+02	6.84718862E+00
4.52868550E+02	5.88717455E+00
4.62868550E+02	5.93812248E+00
4.67144758E+02	5.96943433E+00
4.72868550E+02	6.21561886E+00
4.82868550E+02	5.96842221E+00
4.92868550E+02	5.92366472E+00
5.01434275E+02	6.36588649E+00
5.02868550E+02	6.34257215E+00
5.67144758E+02	5.89221419E+00
6.01434275E+02	5.30438081E+00
6.02868550E+02	5.26353035E+00
6.67144758E+02	5.62399924E+00

6.71448584E+02	5.12493360E+00
6.71449584E+02	5.12508905E+00
6.71450584E+02	5.12518216E+00
6.71451584E+02	5.12522132E+00
6.71452584E+02	5.12521236E+00
6.71453584E+02	5.12516041E+00
6.71454584E+02	5.12506850E+00
6.71455584E+02	5.12494044E+00
6.71456584E+02	5.12477782E+00
6.71457584E+02	5.12458374E+00
6.71467584E+02	5.12141828E+00
6.71477584E+02	5.11726058E+00
6.71487584E+02	5.11321435E+00
6.71497584E+02	5.10971173E+00
6.71507584E+02	5.10681818E+00
6.71517584E+02	5.10446647E+00
6.71527584E+02	5.10255626E+00
6.71537584E+02	5.10098943E+00
6.71547584E+02	5.09968753E+00
6.71647584E+02	5.09259774E+00
6.71747584E+02	5.08741879E+00
6.71847584E+02	5.08298271E+00
6.71947584E+02	5.07815190E+00
6.72047584E+02	5.06966162E+00
6.72147584E+02	5.05510707E+00
6.72247584E+02	5.03733398E+00
6.72347584E+02	5.02138667E+00
6.77447584E+02	4.65104983E+00
6.78447584E+02	4.58086437E+00
6.79447584E+02	4.58581523E+00
6.80447584E+02	4.65701738E+00
6.81447584E+02	4.69903315E+00
6.91447584E+02	4.83720587E+00
7.01434275E+02	4.68411406E+00
7.01447584E+02	4.68378616E+00
7.02868550E+02	4.67484669E+00
7.11447584E+02	4.42433985E+00
7.21447584E+02	4.10952656E+00
7.31447584E+02	4.30270621E+00
7.41447584E+02	4.36937575E+00
7.51447584E+02	4.29168376E+00
7.61447584E+02	4.27176676E+00
7.67144758E+02	4.25837902E+00
7.71447584E+02	4.31890636E+00
8.01434275E+02	3.80651807E+00
8.02868550E+02	3.83211813E+00
8.67144758E+02	3.86272820E+00
8.71447584E+02	3.72244383E+00
9.01434275E+02	3.62163706E+00
9.02868550E+02	3.55149420E+00

9.67144758E+02	3.43705608E+00
9.71447584E+02	3.50942079E+00
1.00143428E+03	3.32767008E+00
1.00286855E+03	3.34466104E+00
1.00717238E+03	3.26421842E+00
1.00717338E+03	3.26409091E+00
1.00717438E+03	3.26398308E+00
1.00717538E+03	3.26388468E+00
1.00717638E+03	3.26379411E+00
1.00717738E+03	3.26370968E+00
1.00717838E+03	3.26363086E+00
1.00717938E+03	3.26355641E+00
1.00718038E+03	3.26348569E+00
1.00718138E+03	3.26341940E+00
1.00719138E+03	3.26293124E+00
1.00720138E+03	3.26266848E+00
1.00721138E+03	3.26254198E+00
1.00722138E+03	3.26250037E+00
1.00723138E+03	3.26251207E+00
1.00724138E+03	3.26255978E+00
1.00725138E+03	3.26263217E+00
1.00726138E+03	3.26272100E+00
1.00727138E+03	3.26282152E+00
1.00737138E+03	3.26400612E+00
1.00747138E+03	3.26524324E+00
1.00757138E+03	3.26739706E+00
1.00767138E+03	3.27121551E+00
1.00777138E+03	3.27597378E+00
1.00787138E+03	3.28090509E+00
1.00797138E+03	3.28576930E+00
1.00807138E+03	3.29067370E+00
1.01317138E+03	3.40397574E+00
1.01417138E+03	3.41181686E+00
1.01517138E+03	3.42227736E+00
1.01617138E+03	3.43814623E+00
1.01717138E+03	3.45582244E+00
1.02717138E+03	3.49503904E+00
1.03717138E+03	3.16944008E+00
1.04717138E+03	3.13118577E+00
1.05717138E+03	3.12529196E+00
1.06714476E+03	2.96403192E+00
1.06717138E+03	2.95823649E+00
1.07144758E+03	2.94574341E+00
1.07717138E+03	2.94363706E+00
1.08717138E+03	3.08488774E+00
1.09717138E+03	2.95273905E+00
1.10143428E+03	3.05606353E+00
1.10286855E+03	3.06997802E+00
1.10717138E+03	3.09954819E+00
1.16714476E+03	2.68746746E+00

1.17144758E+03	2.67758139E+00
1.20143428E+03	2.47808886E+00
1.20286855E+03	2.48576284E+00
1.20717138E+03	2.55658390E+00
1.26714476E+03	2.54227332E+00
1.27144758E+03	2.47277851E+00
1.30143428E+03	2.56461059E+00
1.30286855E+03	2.52419094E+00
1.30717138E+03	2.55314255E+00
1.36714476E+03	2.50392764E+00
1.37144758E+03	2.53110602E+00
1.40143428E+03	2.73288400E+00
1.40286855E+03	2.75741806E+00
1.40717138E+03	2.78099891E+00
1.46714476E+03	2.68962538E+00
1.47144758E+03	2.65577461E+00
1.50143428E+03	2.60373606E+00
1.50286855E+03	2.61269164E+00
1.50717138E+03	2.64491786E+00
1.56714476E+03	2.84437670E+00
1.57144758E+03	2.83099901E+00
1.60143428E+03	2.57466264E+00
1.60286855E+03	2.57108488E+00
1.60717138E+03	2.56680258E+00
1.66714476E+03	2.59062664E+00
1.67144758E+03	2.58422512E+00
1.70143428E+03	2.31757066E+00
1.70286855E+03	2.45324304E+00
1.70717138E+03	2.44089224E+00
1.76714476E+03	2.41433962E+00
1.77144758E+03	2.44179458E+00
1.80143428E+03	2.29002667E+00
1.80286855E+03	2.33299337E+00
1.80717138E+03	2.45980503E+00
1.86714476E+03	2.25459064E+00
1.87144758E+03	2.24912889E+00
1.90143428E+03	2.31093698E+00
1.90286855E+03	2.31595557E+00
1.90717138E+03	2.32688093E+00
1.96714476E+03	2.23129240E+00
1.97144758E+03	2.19422634E+00
2.00143428E+03	2.09963625E+00
2.00286855E+03	2.12094685E+00
2.00717138E+03	2.17115386E+00
2.06714476E+03	2.26360961E+00
2.07144758E+03	2.27753177E+00
2.10143428E+03	2.24745232E+00
2.10286855E+03	2.24918449E+00
2.10717138E+03	2.24828925E+00
2.16714476E+03	2.08080241E+00

2.17144758E+03	2.08133971E+00
2.20143428E+03	2.12338298E+00
2.20286855E+03	2.09849070E+00
2.20717138E+03	2.10749380E+00
2.26714476E+03	1.99342903E+00
2.27144758E+03	1.95687430E+00
2.30143428E+03	1.94932080E+00
2.30286855E+03	1.94861336E+00
2.30717138E+03	1.94775730E+00
2.36714476E+03	1.94173338E+00
2.37144758E+03	1.87894251E+00
2.40143428E+03	1.95363071E+00
2.40286855E+03	1.95562530E+00
2.40717138E+03	1.95614393E+00
2.46714476E+03	1.86197680E+00
2.47144758E+03	1.88725996E+00
2.50143428E+03	1.82972936E+00
2.50286855E+03	1.83094930E+00
2.50717138E+03	1.83330524E+00
2.56714476E+03	1.87213979E+00
2.57144758E+03	1.88402292E+00
2.60143428E+03	1.78122138E+00
2.60286855E+03	1.78095418E+00
2.60717138E+03	1.77792551E+00
2.66714476E+03	1.79226371E+00
2.67144758E+03	1.79602000E+00
2.70143428E+03	1.81958307E+00
2.70286855E+03	1.82119253E+00
2.70717138E+03	1.82768659E+00
2.76714476E+03	1.80677291E+00
2.77144758E+03	1.75723290E+00
2.80143428E+03	1.72416088E+00
2.80286855E+03	1.70325314E+00
2.80717138E+03	1.67765487E+00
2.86714476E+03	1.75973610E+00
2.87144758E+03	1.75026662E+00
2.90143428E+03	1.76991522E+00
2.90286855E+03	1.77047936E+00
2.90717138E+03	1.77221090E+00
2.96714476E+03	1.67131469E+00
2.97144758E+03	1.65477406E+00
3.00143428E+03	1.65080193E+00
3.00286855E+03	1.65126714E+00
3.00717138E+03	1.64919390E+00
3.07144758E+03	1.71297654E+00
3.10143428E+03	1.70185494E+00
3.10286855E+03	1.70038367E+00
3.10717138E+03	1.69511463E+00
3.17144758E+03	1.55788462E+00
3.20286855E+03	1.55670038E+00

3.20717138E+03 1.56049684E+00
3.27144758E+03 1.64115343E+00
3.30286855E+03 1.62053955E+00
3.30717138E+03 1.62005534E+00
3.37144758E+03 1.52258305E+00
3.40717138E+03 1.50258965E+00
3.47144758E+03 1.58676496E+00
3.50717138E+03 1.56721365E+00
3.57144758E+03 1.52668052E+00
3.60717138E+03 1.48287586E+00
3.70717138E+03 1.55721064E+00
3.80717138E+03 1.48547489E+00
3.90717138E+03 1.53451660E+00

#Tot_ener(cm-1) 2--->1 Cross_sec(angs^2)
2.01435275E+02 5.00216658E+03
2.01436275E+02 3.35371772E+03
2.01437275E+02 2.64492927E+03
2.01438275E+02 2.23500176E+03
2.01439275E+02 1.96354705E+03
2.01440275E+02 1.76893611E+03
2.01441275E+02 1.62201827E+03
2.01442275E+02 1.50686647E+03
2.01443275E+02 1.41401795E+03
2.01444275E+02 1.33750147E+03
2.01454275E+02 9.63606287E+02
2.01464275E+02 8.23862719E+02
2.01474275E+02 7.48122257E+02
2.01484275E+02 6.98847974E+02
2.01494275E+02 6.62889401E+02
2.01504275E+02 6.34405594E+02
2.01514275E+02 6.10491747E+02
2.01524275E+02 5.89620441E+02
2.01534275E+02 5.71035453E+02
2.01634275E+02 5.11581178E+02
2.01734275E+02 6.49931798E+02
2.01834275E+02 6.06716739E+02
2.01934275E+02 4.81193767E+02
2.02034275E+02 3.87123059E+02
2.02134275E+02 3.29519293E+02
2.02234275E+02 2.91838972E+02
2.02334275E+02 2.66749419E+02
2.07434275E+02 1.25329972E+02
2.08434275E+02 1.15622780E+02
2.09434275E+02 1.06575029E+02
2.10434275E+02 1.00372073E+02
2.11434275E+02 9.55728868E+01
2.21434275E+02 8.14802133E+01
2.31434275E+02 6.75243210E+01
2.41434275E+02 5.61962327E+01

2.51434275E+02	4.86694360E+01
2.61434275E+02	4.90865192E+01
2.67144758E+02	4.60928906E+01
2.71434275E+02	4.27406987E+01
2.81434275E+02	3.62037254E+01
2.91434275E+02	3.85774063E+01
3.01434275E+02	3.61915652E+01
3.67144758E+02	2.85630549E+01
4.01434275E+02	2.93355668E+01
4.02869550E+02	2.84141061E+01
4.02870550E+02	2.84041570E+01
4.02871550E+02	2.83968757E+01
4.02872550E+02	2.83908381E+01
4.02873550E+02	2.83855130E+01
4.02874550E+02	2.83806281E+01
4.02875550E+02	2.83760359E+01
4.02876550E+02	2.83716369E+01
4.02877550E+02	2.83673803E+01
4.02878550E+02	2.83632039E+01
4.02888550E+02	2.83223215E+01
4.02898550E+02	2.82792270E+01
4.02908550E+02	2.82336367E+01
4.02918550E+02	2.81867420E+01
4.02928550E+02	2.81399344E+01
4.02938550E+02	2.80943223E+01
4.02948550E+02	2.80506203E+01
4.02958550E+02	2.80091359E+01
4.02968550E+02	2.79698858E+01
4.03068550E+02	2.76418848E+01
4.03168550E+02	2.73882405E+01
4.03268550E+02	2.70927058E+01
4.03368550E+02	2.66946369E+01
4.03468550E+02	2.66280883E+01
4.03568550E+02	2.67508908E+01
4.03668550E+02	2.68283869E+01
4.03768550E+02	2.68620323E+01
4.08868550E+02	2.52759663E+01
4.09868550E+02	2.41308300E+01
4.10868550E+02	2.30728661E+01
4.11868550E+02	2.35482055E+01
4.12868550E+02	2.34500526E+01
4.22868550E+02	2.00459552E+01
4.32868550E+02	1.88784618E+01
4.42868550E+02	1.69793302E+01
4.52868550E+02	1.81316834E+01
4.62868550E+02	1.65445388E+01
4.67144758E+02	1.66434241E+01
4.72868550E+02	1.69106134E+01
4.82868550E+02	1.57186486E+01
4.92868550E+02	1.57273362E+01

5.01434275E+02 1.51598514E+01
5.02868550E+02 1.53219355E+01
5.67144758E+02 1.37318031E+01
6.01434275E+02 1.35787255E+01
6.02868550E+02 1.36058365E+01
6.67144758E+02 1.35148626E+01
6.71448584E+02 1.32900318E+01
6.71449584E+02 1.32892953E+01
6.71450584E+02 1.32890026E+01
6.71451584E+02 1.32888519E+01
6.71452584E+02 1.32887501E+01
6.71453584E+02 1.32886641E+01
6.71454584E+02 1.32885795E+01
6.71455584E+02 1.32884816E+01
6.71456584E+02 1.32883737E+01
6.71457584E+02 1.32882465E+01
6.71467584E+02 1.32863577E+01
6.71477584E+02 1.32840090E+01
6.71487584E+02 1.32819602E+01
6.71497584E+02 1.32804611E+01
6.71507584E+02 1.32794793E+01
6.71517584E+02 1.32788840E+01
6.71527584E+02 1.32785381E+01
6.71537584E+02 1.32783299E+01
6.71547584E+02 1.32781827E+01
6.71647584E+02 1.32734620E+01
6.71747584E+02 1.32639888E+01
6.71847584E+02 1.32573261E+01
6.71947584E+02 1.32485215E+01
6.72047584E+02 1.32333410E+01
6.72147584E+02 1.32189269E+01
6.72247584E+02 1.32219564E+01
6.72347584E+02 1.32820019E+01
6.77447584E+02 1.25047834E+01
6.78447584E+02 1.23828241E+01
6.79447584E+02 1.26191065E+01
6.80447584E+02 1.30019842E+01
6.81447584E+02 1.31392423E+01
6.91447584E+02 1.31166857E+01
7.01434275E+02 1.27832295E+01
7.01447584E+02 1.27833260E+01
7.02868550E+02 1.28544310E+01
7.11447584E+02 1.32549069E+01
7.21447584E+02 1.32981861E+01
7.31447584E+02 1.25452736E+01
7.41447584E+02 1.22390572E+01
7.51447584E+02 1.25659989E+01
7.61447584E+02 1.20387311E+01
7.67144758E+02 1.18501959E+01
7.71447584E+02 1.21028282E+01

8.01434275E+02	1.18506421E+01
8.02868550E+02	1.18686072E+01
8.67144758E+02	1.16613120E+01
8.71447584E+02	1.17682935E+01
9.01434275E+02	1.15392762E+01
9.02868550E+02	1.16954269E+01
9.67144758E+02	1.15449046E+01
9.71447584E+02	1.14700635E+01
1.00143428E+03	1.13533134E+01
1.00286855E+03	1.14802805E+01
1.00717238E+03	1.16880067E+01
1.00717338E+03	1.16879054E+01
1.00717438E+03	1.16878241E+01
1.00717538E+03	1.16877485E+01
1.00717638E+03	1.16876807E+01
1.00717738E+03	1.16876138E+01
1.00717838E+03	1.16875515E+01
1.00717938E+03	1.16874916E+01
1.00718038E+03	1.16874308E+01
1.00718138E+03	1.16873721E+01
1.00719138E+03	1.16868774E+01
1.00720138E+03	1.16864951E+01
1.00721138E+03	1.16861616E+01
1.00722138E+03	1.16858164E+01
1.00723138E+03	1.16854285E+01
1.00724138E+03	1.16849759E+01
1.00725138E+03	1.16844527E+01
1.00726138E+03	1.16838649E+01
1.00727138E+03	1.16832171E+01
1.00737138E+03	1.16754236E+01
1.00747138E+03	1.16717907E+01
1.00757138E+03	1.16758236E+01
1.00767138E+03	1.16774453E+01
1.00777138E+03	1.16777620E+01
1.00787138E+03	1.16784801E+01
1.00797138E+03	1.16804003E+01
1.00807138E+03	1.16838964E+01
1.01317138E+03	1.17334163E+01
1.01417138E+03	1.17315821E+01
1.01517138E+03	1.17288973E+01
1.01617138E+03	1.17213430E+01
1.01717138E+03	1.16995927E+01
1.02717138E+03	1.12150229E+01
1.03717138E+03	1.14475006E+01
1.04717138E+03	1.09027968E+01
1.05717138E+03	1.01938214E+01
1.06714476E+03	1.03192996E+01
1.06717138E+03	1.03487757E+01
1.07144758E+03	1.03439594E+01
1.07717138E+03	9.89929924E+00

1.08717138E+03	1.05915235E+01
1.09717138E+03	1.09199196E+01
1.10143428E+03	1.05812073E+01
1.10286855E+03	1.04541185E+01
1.10717138E+03	1.05382722E+01
1.16714476E+03	1.01625841E+01
1.17144758E+03	9.98576929E+00
1.20143428E+03	9.43950620E+00
1.20286855E+03	9.50937250E+00
1.20717138E+03	9.65739320E+00
1.26714476E+03	1.00127310E+01
1.27144758E+03	9.82384130E+00
1.30143428E+03	9.31690202E+00
1.30286855E+03	9.45770492E+00
1.30717138E+03	9.29903234E+00
1.36714476E+03	9.61036667E+00
1.37144758E+03	9.60665500E+00
1.40143428E+03	9.47440917E+00
1.40286855E+03	9.49202287E+00
1.40717138E+03	9.50145690E+00
1.46714476E+03	9.03647954E+00
1.47144758E+03	9.06691051E+00
1.50143428E+03	8.88459666E+00
1.50286855E+03	8.88876927E+00
1.50717138E+03	8.91919016E+00
1.56714476E+03	9.16394991E+00
1.57144758E+03	9.11709906E+00
1.60143428E+03	8.81040385E+00
1.60286855E+03	8.83984169E+00
1.60717138E+03	9.10097165E+00
1.66714476E+03	9.06466115E+00
1.67144758E+03	9.06921181E+00
1.70143428E+03	9.16784376E+00
1.70286855E+03	9.12856050E+00
1.70717138E+03	9.11061543E+00
1.76714476E+03	8.904334865E+00
1.77144758E+03	8.89211855E+00
1.80143428E+03	8.84511500E+00
1.80286855E+03	8.77991415E+00
1.80717138E+03	8.91003064E+00
1.86714476E+03	8.98157655E+00
1.87144758E+03	8.91729142E+00
1.90143428E+03	8.94391358E+00
1.90286855E+03	8.94339864E+00
1.90717138E+03	8.93628167E+00
1.96714476E+03	8.79977957E+00
1.97144758E+03	9.00119475E+00
2.00143428E+03	8.59833963E+00
2.00286855E+03	8.59516365E+00
2.00717138E+03	8.68171996E+00

2.06714476E+03	8.97997529E+00
2.07144758E+03	8.95358108E+00
2.10143428E+03	9.00963026E+00
2.10286855E+03	9.01592155E+00
2.10717138E+03	9.02758546E+00
2.16714476E+03	8.78683577E+00
2.17144758E+03	8.75709650E+00
2.20143428E+03	8.79148617E+00
2.20286855E+03	8.86999081E+00
2.20717138E+03	8.92981334E+00
2.26714476E+03	8.84508321E+00
2.27144758E+03	8.85723212E+00
2.30143428E+03	8.77521029E+00
2.30286855E+03	8.76569980E+00
2.30717138E+03	8.74031406E+00
2.36714476E+03	8.69999055E+00
2.37144758E+03	8.70885152E+00
2.40143428E+03	8.79666320E+00
2.40286855E+03	8.79506457E+00
2.40717138E+03	8.77155489E+00
2.46714476E+03	8.58872007E+00
2.47144758E+03	8.54177221E+00
2.50143428E+03	8.49213022E+00
2.50286855E+03	8.49059727E+00
2.50717138E+03	8.48520611E+00
2.56714476E+03	8.47740078E+00
2.57144758E+03	8.45892271E+00
2.60143428E+03	8.39517720E+00
2.60286855E+03	8.39064361E+00
2.60717138E+03	8.37441098E+00
2.66714476E+03	8.32517079E+00
2.67144758E+03	8.32315409E+00
2.70143428E+03	8.27812817E+00
2.70286855E+03	8.27452117E+00
2.70717138E+03	8.26124162E+00
2.76714476E+03	8.11445054E+00
2.77144758E+03	8.09166365E+00
2.80143428E+03	8.09220726E+00
2.80286855E+03	8.08205916E+00
2.80717138E+03	8.09067824E+00
2.86714476E+03	8.10329028E+00
2.87144758E+03	8.13043502E+00
2.90143428E+03	8.12119463E+00
2.90286855E+03	8.11838697E+00
2.90717138E+03	8.10882879E+00
2.96714476E+03	7.95821417E+00
2.97144758E+03	7.94666432E+00
3.00143428E+03	8.00024915E+00
3.00286855E+03	7.99371072E+00
3.00717138E+03	7.97449267E+00

3.07144758E+03 7.92234236E+00
3.10143428E+03 7.97788783E+00
3.10286855E+03 7.97598709E+00
3.10717138E+03 7.96664199E+00
3.17144758E+03 7.83268834E+00
3.20286855E+03 7.81725517E+00
3.20717138E+03 7.78949966E+00
3.27144758E+03 7.82794531E+00
3.30286855E+03 7.80156019E+00
3.30717138E+03 7.81034019E+00
3.37144758E+03 7.77090169E+00
3.40717138E+03 7.70657087E+00
3.47144758E+03 7.73636751E+00
3.50717138E+03 7.67535608E+00
3.57144758E+03 7.63954159E+00
3.60717138E+03 7.65710286E+00
3.70717138E+03 7.62790241E+00
3.80717138E+03 7.51392821E+00
3.90717138E+03 7.56349640E+00

#Tot_ener(cm-1) 3--->1 Cross_sec(angs^2)
4.02869550E+02 7.69342906E+03
4.02870550E+02 4.98395227E+03
4.02871550E+02 3.82749404E+03
4.02872550E+02 3.16221580E+03
4.02873550E+02 2.72351323E+03
4.02874550E+02 2.41021629E+03
4.02875550E+02 2.17437754E+03
4.02876550E+02 1.99004002E+03
4.02877550E+02 1.84182136E+03
4.02878550E+02 1.71997143E+03
4.02888550E+02 1.13071497E+03
4.02898550E+02 9.15754905E+02
4.02908550E+02 8.02580842E+02
4.02918550E+02 7.32013300E+02
4.02928550E+02 6.83325759E+02
4.02938550E+02 6.46999342E+02
4.02948550E+02 6.18018072E+02
4.02958550E+02 5.93585623E+02
4.02968550E+02 5.72130257E+02
4.03068550E+02 4.38919319E+02
4.03168550E+02 3.74110924E+02
4.03268550E+02 3.18771238E+02
4.03368550E+02 2.89100332E+02
4.03468550E+02 2.63328622E+02
4.03568550E+02 2.43400817E+02
4.03668550E+02 2.30076627E+02
4.03768550E+02 2.17257771E+02
4.08868550E+02 9.01414081E+01
4.09868550E+02 8.46716511E+01

4.10868550E+02 7.79094525E+01
4.11868550E+02 7.44913626E+01
4.12868550E+02 7.26812585E+01
4.22868550E+02 5.37450688E+01
4.32868550E+02 4.37973619E+01
4.42868550E+02 4.02876000E+01
4.52868550E+02 3.46400102E+01
4.62868550E+02 3.61765022E+01
4.67144758E+02 3.28089656E+01
4.72868550E+02 3.01348457E+01
4.82868550E+02 2.87907078E+01
4.92868550E+02 2.64676135E+01
5.01434275E+02 2.48717508E+01
5.02868550E+02 2.47090412E+01
5.67144758E+02 1.65643378E+01
6.01434275E+02 1.52386965E+01
6.02868550E+02 1.52408740E+01
6.67144758E+02 1.26823826E+01
6.71448584E+02 1.27098092E+01
6.71449584E+02 1.27104232E+01
6.71450584E+02 1.27108847E+01
6.71451584E+02 1.27112222E+01
6.71452584E+02 1.27114615E+01
6.71453584E+02 1.27116218E+01
6.71454584E+02 1.27117169E+01
6.71455584E+02 1.27117578E+01
6.71456584E+02 1.27117544E+01
6.71457584E+02 1.27117141E+01
6.71467584E+02 1.27102267E+01
6.71477584E+02 1.27084489E+01
6.71487584E+02 1.27071931E+01
6.71497584E+02 1.27064150E+01
6.71507584E+02 1.27058895E+01
6.71517584E+02 1.27054211E+01
6.71527584E+02 1.27048759E+01
6.71537584E+02 1.27041729E+01
6.71547584E+02 1.27032648E+01
6.71647584E+02 1.26805354E+01
6.71747584E+02 1.26395761E+01
6.71847584E+02 1.25921579E+01
6.71947584E+02 1.25504515E+01
6.72047584E+02 1.25228255E+01
6.72147584E+02 1.25110439E+01
6.72247584E+02 1.25094759E+01
6.72347584E+02 1.25100944E+01
6.77447584E+02 1.21233823E+01
6.78447584E+02 1.21631538E+01
6.79447584E+02 1.20684520E+01
6.80447584E+02 1.17949851E+01
6.81447584E+02 1.15524362E+01

6.91447584E+02	1.13543810E+01
7.01434275E+02	1.13123844E+01
7.01447584E+02	1.13112842E+01
7.02868550E+02	1.11222008E+01
7.11447584E+02	1.07548365E+01
7.21447584E+02	1.04053685E+01
7.31447584E+02	1.02414089E+01
7.41447584E+02	9.95716472E+00
7.51447584E+02	9.24343244E+00
7.61447584E+02	9.29961236E+00
7.67144758E+02	9.11127954E+00
7.71447584E+02	9.25478112E+00
8.01434275E+02	8.55530580E+00
8.02868550E+02	8.51082772E+00
8.67144758E+02	7.97859577E+00
8.71447584E+02	7.85022903E+00
9.01434275E+02	7.22262307E+00
9.02868550E+02	7.25322062E+00
9.67144758E+02	6.96291496E+00
9.71447584E+02	6.96959517E+00
1.00143428E+03	6.73289323E+00
1.00286855E+03	6.61528434E+00
1.00717238E+03	6.59220793E+00
1.00717338E+03	6.59204435E+00
1.00717438E+03	6.59188734E+00
1.00717538E+03	6.59172838E+00
1.00717638E+03	6.59156718E+00
1.00717738E+03	6.59140243E+00
1.00717838E+03	6.59123356E+00
1.00717938E+03	6.59106083E+00
1.00718038E+03	6.59088312E+00
1.00718138E+03	6.59070240E+00
1.00719138E+03	6.58873209E+00
1.00720138E+03	6.58663970E+00
1.00721138E+03	6.58458293E+00
1.00722138E+03	6.58263399E+00
1.00723138E+03	6.58082282E+00
1.00724138E+03	6.57915933E+00
1.00725138E+03	6.57764545E+00
1.00726138E+03	6.57627960E+00
1.00727138E+03	6.57505497E+00
1.00737138E+03	6.56824750E+00
1.00747138E+03	6.56088133E+00
1.00757138E+03	6.54924013E+00
1.00767138E+03	6.54334384E+00
1.00777138E+03	6.53765762E+00
1.00787138E+03	6.52866749E+00
1.00797138E+03	6.51685108E+00
1.00807138E+03	6.50458775E+00
1.01317138E+03	6.51485868E+00

1.01417138E+03 6.48386403E+00
1.01517138E+03 6.46839034E+00
1.01617138E+03 6.44575938E+00
1.01717138E+03 6.42072207E+00
1.02717138E+03 6.40445038E+00
1.03717138E+03 6.31997452E+00
1.04717138E+03 6.44784670E+00
1.05717138E+03 6.51659795E+00
1.06714476E+03 5.92140258E+00
1.06717138E+03 5.89562824E+00
1.07144758E+03 5.80746087E+00
1.07717138E+03 6.09556931E+00
1.08717138E+03 5.99483439E+00
1.09717138E+03 5.99755216E+00
1.10143428E+03 5.76746337E+00
1.10286855E+03 5.71133747E+00
1.10717138E+03 5.60305481E+00
1.16714476E+03 5.63402860E+00
1.17144758E+03 5.63743741E+00
1.20143428E+03 5.30071540E+00
1.20286855E+03 5.29323697E+00
1.20717138E+03 5.34702453E+00
1.26714476E+03 5.07226971E+00
1.27144758E+03 5.07687659E+00
1.30143428E+03 5.10011139E+00
1.30286855E+03 5.22233226E+00
1.30717138E+03 5.26512061E+00
1.36714476E+03 5.05997744E+00
1.37144758E+03 4.90797513E+00
1.40143428E+03 4.77460759E+00
1.40286855E+03 4.74135468E+00
1.40717138E+03 4.75407495E+00
1.46714476E+03 4.79904044E+00
1.47144758E+03 4.84497211E+00
1.50143428E+03 4.53863352E+00
1.50286855E+03 4.54017046E+00
1.50717138E+03 4.53816454E+00
1.56714476E+03 4.55890458E+00
1.57144758E+03 4.59826386E+00
1.60143428E+03 4.58679770E+00
1.60286855E+03 4.59541053E+00
1.60717138E+03 4.65759150E+00
1.66714476E+03 4.32481481E+00
1.67144758E+03 4.37954968E+00
1.70143428E+03 4.46422102E+00
1.70286855E+03 4.47769734E+00
1.70717138E+03 4.48310509E+00
1.76714476E+03 4.43784397E+00
1.77144758E+03 4.38106630E+00
1.80143428E+03 4.40400107E+00

1.80286855E+03	4.38541757E+00
1.80717138E+03	4.29891072E+00
1.86714476E+03	4.38808783E+00
1.87144758E+03	4.29199132E+00
1.90143428E+03	4.28904927E+00
1.90286855E+03	4.28187622E+00
1.90717138E+03	4.26266046E+00
1.96714476E+03	4.07555225E+00
1.97144758E+03	4.02094514E+00
2.00143428E+03	4.01570743E+00
2.00286855E+03	4.00042779E+00
2.00717138E+03	3.95426016E+00
2.06714476E+03	3.92623208E+00
2.07144758E+03	3.92206576E+00
2.10143428E+03	3.92587704E+00
2.10286855E+03	3.92252951E+00
2.10717138E+03	3.91161716E+00
2.16714476E+03	3.87909742E+00
2.17144758E+03	3.86229714E+00
2.20143428E+03	3.77548120E+00
2.20286855E+03	3.78569304E+00
2.20717138E+03	3.78241468E+00
2.26714476E+03	3.83514331E+00
2.27144758E+03	3.83390630E+00
2.30143428E+03	3.82109992E+00
2.30286855E+03	3.82002290E+00
2.30717138E+03	3.81538487E+00
2.36714476E+03	3.71381535E+00
2.37144758E+03	3.67859523E+00
2.40143428E+03	3.71585005E+00
2.40286855E+03	3.71888152E+00
2.40717138E+03	3.73127475E+00
2.46714476E+03	3.66906799E+00
2.47144758E+03	3.68488740E+00
2.50143428E+03	3.65500105E+00
2.50286855E+03	3.65576301E+00
2.50717138E+03	3.66050507E+00
2.56714476E+03	3.59314976E+00
2.57144758E+03	3.60251353E+00
2.60143428E+03	3.56818361E+00
2.60286855E+03	3.57519356E+00
2.60717138E+03	3.58993168E+00
2.66714476E+03	3.45677344E+00
2.67144758E+03	3.46810566E+00
2.70143428E+03	3.49725811E+00
2.70286855E+03	3.49693427E+00
2.70717138E+03	3.49661983E+00
2.76714476E+03	3.48624114E+00
2.77144758E+03	3.45443104E+00
2.80143428E+03	3.45574414E+00

2.80286855E+03 3.45809537E+00
2.80717138E+03 3.46309569E+00
2.86714476E+03 3.35672407E+00
2.87144758E+03 3.36809244E+00
2.90143428E+03 3.40358871E+00
2.90286855E+03 3.40435431E+00
2.90717138E+03 3.40716600E+00
2.96714476E+03 3.38540102E+00
2.97144758E+03 3.36961874E+00
3.00143428E+03 3.36341687E+00
3.00286855E+03 3.36668715E+00
3.00717138E+03 3.37870405E+00
3.07144758E+03 3.34062341E+00
3.10143428E+03 3.35955331E+00
3.10286855E+03 3.36002318E+00
3.10717138E+03 3.36055384E+00
3.17144758E+03 3.30109911E+00
3.20286855E+03 3.31647888E+00
3.20717138E+03 3.31125804E+00
3.27144758E+03 3.27997320E+00
3.30286855E+03 3.28818537E+00
3.30717138E+03 3.28851990E+00
3.37144758E+03 3.23737464E+00
3.40717138E+03 3.24204026E+00
3.47144758E+03 3.19488984E+00
3.50717138E+03 3.18907868E+00
3.57144758E+03 3.15457023E+00
3.60717138E+03 3.08641285E+00
3.70717138E+03 3.06351066E+00
3.80717138E+03 2.99072076E+00
3.90717138E+03 2.91077483E+00

#Tot_ener(cm-1) 3--->2 Cross_sec(angs^2)
4.02869550E+02 5.45865952E+03
4.02870550E+02 3.55176829E+03
4.02871550E+02 2.74089683E+03
4.02872550E+02 2.27627759E+03
4.02873550E+02 1.97119511E+03
4.02874550E+02 1.75430824E+03
4.02875550E+02 1.59182760E+03
4.02876550E+02 1.46547364E+03
4.02877550E+02 1.36442336E+03
4.02878550E+02 1.28181945E+03
4.02888550E+02 8.94158857E+02
4.02898550E+02 7.64996211E+02
4.02908550E+02 7.04235718E+02
4.02918550E+02 6.70304020E+02
4.02928550E+02 6.48359501E+02
4.02938550E+02 6.31700942E+02
4.02948550E+02 6.17153949E+02

4.02958550E+02	6.03333679E+02
4.02968550E+02	5.89815554E+02
4.03068550E+02	5.07189077E+02
4.03168550E+02	4.41641807E+02
4.03268550E+02	3.98847964E+02
4.03368550E+02	4.31663816E+02
4.03468550E+02	4.07141923E+02
4.03568550E+02	3.62309565E+02
4.03668550E+02	3.33510044E+02
4.03768550E+02	3.11013328E+02
4.08868550E+02	1.37677767E+02
4.09868550E+02	1.33281701E+02
4.10868550E+02	1.21253027E+02
4.11868550E+02	1.16745670E+02
4.12868550E+02	1.16412313E+02
4.22868550E+02	7.69564089E+01
4.32868550E+02	6.52748291E+01
4.42868550E+02	5.34343593E+01
4.52868550E+02	5.20547187E+01
4.62868550E+02	4.84506120E+01
4.67144758E+02	4.42959719E+01
4.72868550E+02	4.14253004E+01
4.82868550E+02	3.93849105E+01
4.92868550E+02	3.90846271E+01
5.01434275E+02	3.43458601E+01
5.02868550E+02	3.41783973E+01
5.67144758E+02	2.77860626E+01
6.01434275E+02	2.34788052E+01
6.02868550E+02	2.36479425E+01
6.67144758E+02	2.10462290E+01
6.71448584E+02	2.04809456E+01
6.71449584E+02	2.04758301E+01
6.71450584E+02	2.04731634E+01
6.71451584E+02	2.04715007E+01
6.71452584E+02	2.04703654E+01
6.71453584E+02	2.04695383E+01
6.71454584E+02	2.04689112E+01
6.71455584E+02	2.04684174E+01
6.71456584E+02	2.04680127E+01
6.71457584E+02	2.04676750E+01
6.71467584E+02	2.04654351E+01
6.71477584E+02	2.04630845E+01
6.71487584E+02	2.04602274E+01
6.71497584E+02	2.04571540E+01
6.71507584E+02	2.04541171E+01
6.71517584E+02	2.04512724E+01
6.71527584E+02	2.04487082E+01
6.71537584E+02	2.04464766E+01
6.71547584E+02	2.04445992E+01
6.71647584E+02	2.04414228E+01

6.71747584E+02	2.04446490E+01
6.71847584E+02	2.04359888E+01
6.71947584E+02	2.04037313E+01
6.72047584E+02	2.03274806E+01
6.72147584E+02	2.02015053E+01
6.72247584E+02	2.00602867E+01
6.72347584E+02	1.99330554E+01
6.77447584E+02	1.90100194E+01
6.78447584E+02	1.91304966E+01
6.79447584E+02	1.91713169E+01
6.80447584E+02	1.90110158E+01
6.81447584E+02	1.89481542E+01
6.91447584E+02	1.81161546E+01
7.01434275E+02	1.67338399E+01
7.01447584E+02	1.67342873E+01
7.02868550E+02	1.69831500E+01
7.11447584E+02	1.73924811E+01
7.21447584E+02	1.59885484E+01
7.31447584E+02	1.54216888E+01
7.41447584E+02	1.52681882E+01
7.51447584E+02	1.45887236E+01
7.61447584E+02	1.44592564E+01
7.67144758E+02	1.45511340E+01
7.71447584E+02	1.47687182E+01
8.01434275E+02	1.38521047E+01
8.02868550E+02	1.38479274E+01
8.67144758E+02	1.26298092E+01
8.71447584E+02	1.25153938E+01
9.01434275E+02	1.25538714E+01
9.02868550E+02	1.25693621E+01
9.67144758E+02	1.24031768E+01
9.71447584E+02	1.22145425E+01
1.00143428E+03	1.13295930E+01
1.00286855E+03	1.13987188E+01
1.00717238E+03	1.14292698E+01
1.00717338E+03	1.14293020E+01
1.00717438E+03	1.14292983E+01
1.00717538E+03	1.14292738E+01
1.00717638E+03	1.14292330E+01
1.00717738E+03	1.14291818E+01
1.00717838E+03	1.14291204E+01
1.00717938E+03	1.14290521E+01
1.00718038E+03	1.14289788E+01
1.00718138E+03	1.14289011E+01
1.00719138E+03	1.14280834E+01
1.00720138E+03	1.14274082E+01
1.00721138E+03	1.14269015E+01
1.00722138E+03	1.14264849E+01
1.00723138E+03	1.14260898E+01
1.00724138E+03	1.14256708E+01

1.00725138E+03	1.14252038E+01
1.00726138E+03	1.14246768E+01
1.00727138E+03	1.14240887E+01
1.00737138E+03	1.14163290E+01
1.00747138E+03	1.14070710E+01
1.00757138E+03	1.14011558E+01
1.00767138E+03	1.13975221E+01
1.00777138E+03	1.13928306E+01
1.00787138E+03	1.13886630E+01
1.00797138E+03	1.13857564E+01
1.00807138E+03	1.13838154E+01
1.01317138E+03	1.12508436E+01
1.01417138E+03	1.12101366E+01
1.01517138E+03	1.11816886E+01
1.01617138E+03	1.11756733E+01
1.01717138E+03	1.11998647E+01
1.02717138E+03	1.13897627E+01
1.03717138E+03	1.10026232E+01
1.04717138E+03	1.10082295E+01
1.05717138E+03	1.11358202E+01
1.06714476E+03	1.06481589E+01
1.06717138E+03	1.06334246E+01
1.07144758E+03	1.08372233E+01
1.07717138E+03	1.07365537E+01
1.08717138E+03	1.03375033E+01
1.09717138E+03	1.03359918E+01
1.10143428E+03	1.03249058E+01
1.10286855E+03	1.03248726E+01
1.10717138E+03	1.02030961E+01
1.16714476E+03	1.01733814E+01
1.17144758E+03	1.01514130E+01
1.20143428E+03	9.98023112E+00
1.20286855E+03	9.97452033E+00
1.20717138E+03	1.01821429E+01
1.26714476E+03	1.00990458E+01
1.27144758E+03	1.01513939E+01
1.30143428E+03	1.00345872E+01
1.30286855E+03	1.00845501E+01
1.30717138E+03	1.02876064E+01
1.36714476E+03	9.83130309E+00
1.37144758E+03	9.76097051E+00
1.40143428E+03	1.01840316E+01
1.40286855E+03	1.01974812E+01
1.40717138E+03	1.02190650E+01
1.46714476E+03	9.18056758E+00
1.47144758E+03	9.25671589E+00
1.50143428E+03	9.43681383E+00
1.50286855E+03	9.43647836E+00
1.50717138E+03	9.42128697E+00
1.56714476E+03	9.20087163E+00

1.57144758E+03 9.18115715E+00
1.60143428E+03 8.98295710E+00
1.60286855E+03 8.99390911E+00
1.60717138E+03 9.01332200E+00
1.66714476E+03 9.31849294E+00
1.67144758E+03 9.34457525E+00
1.70143428E+03 9.22233580E+00
1.70286855E+03 9.15877640E+00
1.70717138E+03 9.13298825E+00
1.76714476E+03 9.04040122E+00
1.77144758E+03 9.01904097E+00
1.80143428E+03 9.15134646E+00
1.80286855E+03 9.19816754E+00
1.80717138E+03 9.29245982E+00
1.86714476E+03 9.14918501E+00
1.87144758E+03 9.22594718E+00
1.90143428E+03 9.26777051E+00
1.90286855E+03 9.27005153E+00
1.90717138E+03 9.25111371E+00
1.96714476E+03 9.08330105E+00
1.97144758E+03 9.15244442E+00
2.00143428E+03 9.06472743E+00
2.00286855E+03 9.08553994E+00
2.00717138E+03 9.13631302E+00
2.06714476E+03 9.19931126E+00
2.07144758E+03 9.19309396E+00
2.10143428E+03 9.07835996E+00
2.10286855E+03 9.07296225E+00
2.10717138E+03 9.06177591E+00
2.16714476E+03 9.02394551E+00
2.17144758E+03 9.09956082E+00
2.20143428E+03 9.15983522E+00
2.20286855E+03 9.07974853E+00
2.20717138E+03 9.24825957E+00
2.26714476E+03 8.95493564E+00
2.27144758E+03 8.99129700E+00
2.30143428E+03 8.88448350E+00
2.30286855E+03 8.88237255E+00
2.30717138E+03 8.87613694E+00
2.36714476E+03 8.78044119E+00
2.37144758E+03 8.83205044E+00
2.40143428E+03 8.97562615E+00
2.40286855E+03 8.96736049E+00
2.40717138E+03 8.94078656E+00
2.46714476E+03 8.84259609E+00
2.47144758E+03 8.85112313E+00
2.50143428E+03 8.80478288E+00
2.50286855E+03 8.80067196E+00
2.50717138E+03 8.78872154E+00
2.56714476E+03 8.76359048E+00

2.57144758E+03 8.77440149E+00
2.60143428E+03 8.72827543E+00
2.60286855E+03 8.72484706E+00
2.60717138E+03 8.71879493E+00
2.66714476E+03 8.72863315E+00
2.67144758E+03 8.73766025E+00
2.70143428E+03 8.63812613E+00
2.70286855E+03 8.63510505E+00
2.70717138E+03 8.62476412E+00
2.76714476E+03 8.54549563E+00
2.77144758E+03 8.57602103E+00
2.80143428E+03 8.53214264E+00
2.80286855E+03 8.52617570E+00
2.80717138E+03 8.52532509E+00
2.86714476E+03 8.54549891E+00
2.87144758E+03 8.48048422E+00
2.90143428E+03 8.51594154E+00
2.90286855E+03 8.51390724E+00
2.90717138E+03 8.50597679E+00
2.96714476E+03 8.40277222E+00
2.97144758E+03 8.39839789E+00
3.00143428E+03 8.41440528E+00
3.00286855E+03 8.40812410E+00
3.00717138E+03 8.39271703E+00
3.07144758E+03 8.34527973E+00
3.10143428E+03 8.32419559E+00
3.10286855E+03 8.32425042E+00
3.10717138E+03 8.32094409E+00
3.17144758E+03 8.23739558E+00
3.20286855E+03 8.21284060E+00
3.20717138E+03 8.19571554E+00
3.27144758E+03 8.15401902E+00
3.30286855E+03 8.11956347E+00
3.30717138E+03 8.12366127E+00
3.37144758E+03 8.13389436E+00
3.40717138E+03 8.10721967E+00
3.47144758E+03 8.07587606E+00
3.50717138E+03 8.04492598E+00
3.57144758E+03 8.02432044E+00
3.60717138E+03 8.03487795E+00
3.70717138E+03 7.97545729E+00
3.80717138E+03 7.92244112E+00
3.90717138E+03 7.89494287E+00

#Tot_ener(cm-1) 4--->2 Cross_sec(angs^2)
6.71448584E+02 1.70040251E+04
6.71449584E+02 9.67297778E+03
6.71450584E+02 6.83933852E+03
6.71451584E+02 5.33363772E+03
6.71452584E+02 4.40572023E+03

6.71453584E+02	3.78144430E+03
6.71454584E+02	3.33608610E+03
6.71455584E+02	3.00462040E+03
6.71456584E+02	2.74980989E+03
6.71457584E+02	2.54880692E+03
6.71467584E+02	1.69317398E+03
6.71477584E+02	1.40168602E+03
6.71487584E+02	1.21845113E+03
6.71497584E+02	1.07849943E+03
6.71507584E+02	9.67015391E+02
6.71517584E+02	8.77662737E+02
6.71527584E+02	8.05893902E+02
6.71537584E+02	7.47900755E+02
6.71547584E+02	7.00578973E+02
6.71647584E+02	4.73418326E+02
6.71747584E+02	3.71559634E+02
6.71847584E+02	3.14690975E+02
6.71947584E+02	2.91476441E+02
6.72047584E+02	2.91025258E+02
6.72147584E+02	2.93437013E+02
6.72247584E+02	2.84433900E+02
6.72347584E+02	2.66878937E+02
6.77447584E+02	9.68533054E+01
6.78447584E+02	9.17940707E+01
6.79447584E+02	8.59944981E+01
6.80447584E+02	8.02340823E+01
6.81447584E+02	7.65793244E+01
6.91447584E+02	5.26551228E+01
7.01434275E+02	4.48862184E+01
7.01447584E+02	4.48751555E+01
7.02868550E+02	4.33941902E+01
7.11447584E+02	3.81740155E+01
7.21447584E+02	3.52511816E+01
7.31447584E+02	3.36498303E+01
7.41447584E+02	3.08597686E+01
7.51447584E+02	3.14391312E+01
7.61447584E+02	2.83465455E+01
7.67144758E+02	2.74490845E+01
7.71447584E+02	2.73665560E+01
8.01434275E+02	2.46960766E+01
8.02868550E+02	2.50443227E+01
8.67144758E+02	1.88150352E+01
8.71447584E+02	1.84030403E+01
9.01434275E+02	1.67777161E+01
9.02868550E+02	1.67083799E+01
9.67144758E+02	1.39324800E+01
9.71447584E+02	1.38346914E+01
1.00143428E+03	1.42039996E+01
1.00286855E+03	1.39387723E+01
1.00717238E+03	1.38612925E+01

1.00717338E+03	1.38606076E+01
1.00717438E+03	1.38599236E+01
1.00717538E+03	1.38592345E+01
1.00717638E+03	1.38585417E+01
1.00717738E+03	1.38578466E+01
1.00717838E+03	1.38571515E+01
1.00717938E+03	1.38564582E+01
1.00718038E+03	1.38557680E+01
1.00718138E+03	1.38550830E+01
1.00719138E+03	1.38486202E+01
1.00720138E+03	1.38428878E+01
1.00721138E+03	1.38376633E+01
1.00722138E+03	1.38327309E+01
1.00723138E+03	1.38279611E+01
1.00724138E+03	1.38232852E+01
1.00725138E+03	1.38186758E+01
1.00726138E+03	1.38141246E+01
1.00727138E+03	1.38096308E+01
1.00737138E+03	1.37658011E+01
1.00747138E+03	1.37069815E+01
1.00757138E+03	1.36443097E+01
1.00767138E+03	1.35967947E+01
1.00777138E+03	1.35572857E+01
1.00787138E+03	1.35198282E+01
1.00797138E+03	1.34836208E+01
1.00807138E+03	1.34488992E+01
1.01317138E+03	1.26571015E+01
1.01417138E+03	1.25972717E+01
1.01517138E+03	1.25868551E+01
1.01617138E+03	1.25580016E+01
1.01717138E+03	1.25252583E+01
1.02717138E+03	1.22811376E+01
1.03717138E+03	1.20406506E+01
1.04717138E+03	1.20424633E+01
1.05717138E+03	1.17904095E+01
1.06714476E+03	1.12750731E+01
1.06717138E+03	1.12793018E+01
1.07144758E+03	1.10694914E+01
1.07717138E+03	1.06356756E+01
1.08717138E+03	1.07002128E+01
1.09717138E+03	1.06232380E+01
1.10143428E+03	1.06012904E+01
1.10286855E+03	1.06315774E+01
1.10717138E+03	1.04655873E+01
1.16714476E+03	9.49291030E+00
1.17144758E+03	9.49833844E+00
1.20143428E+03	8.92494210E+00
1.20286855E+03	8.89195675E+00
1.20717138E+03	8.80992781E+00
1.26714476E+03	8.28941133E+00

1.27144758E+03	8.28915021E+00
1.30143428E+03	7.79680452E+00
1.30286855E+03	7.79446428E+00
1.30717138E+03	7.72332055E+00
1.36714476E+03	7.07813020E+00
1.37144758E+03	7.12211931E+00
1.40143428E+03	7.37376573E+00
1.40286855E+03	7.35308817E+00
1.40717138E+03	7.25410781E+00
1.46714476E+03	6.65436806E+00
1.47144758E+03	6.60298288E+00
1.50143428E+03	6.23095624E+00
1.50286855E+03	6.21408555E+00
1.50717138E+03	6.20198646E+00
1.56714476E+03	6.03939158E+00
1.57144758E+03	6.04330486E+00
1.60143428E+03	6.09417194E+00
1.60286855E+03	6.10596545E+00
1.60717138E+03	6.12751814E+00
1.66714476E+03	5.63168988E+00
1.67144758E+03	5.65117271E+00
1.70143428E+03	5.70677103E+00
1.70286855E+03	5.70941368E+00
1.70717138E+03	5.71480071E+00
1.76714476E+03	5.65900820E+00
1.77144758E+03	5.66798482E+00
1.80143428E+03	5.65933491E+00
1.80286855E+03	5.66714692E+00
1.80717138E+03	5.59486129E+00
1.86714476E+03	5.35333434E+00
1.87144758E+03	5.41197801E+00
1.90143428E+03	5.29138214E+00
1.90286855E+03	5.29790211E+00
1.90717138E+03	5.30988740E+00
1.96714476E+03	5.33235469E+00
1.97144758E+03	5.29101343E+00
2.00143428E+03	5.08710574E+00
2.00286855E+03	5.07911942E+00
2.00717138E+03	5.03370007E+00
2.06714476E+03	5.03763429E+00
2.07144758E+03	5.04383148E+00
2.10143428E+03	5.07169067E+00
2.10286855E+03	5.07365167E+00
2.10717138E+03	5.07647476E+00
2.16714476E+03	4.78262075E+00
2.17144758E+03	4.78334770E+00
2.20143428E+03	4.69582022E+00
2.20286855E+03	4.66621660E+00
2.20717138E+03	4.66312560E+00
2.26714476E+03	4.80036264E+00

2.27144758E+03 4.78879063E+00
2.30143428E+03 4.67486568E+00
2.30286855E+03 4.66987177E+00
2.30717138E+03 4.65512691E+00
2.36714476E+03 4.56098958E+00
2.37144758E+03 4.51791648E+00
2.40143428E+03 4.47311471E+00
2.40286855E+03 4.47577472E+00
2.40717138E+03 4.49180524E+00
2.46714476E+03 4.46550770E+00
2.47144758E+03 4.43949133E+00
2.50143428E+03 4.37307431E+00
2.50286855E+03 4.37761100E+00
2.50717138E+03 4.39265320E+00
2.56714476E+03 4.27161969E+00
2.57144758E+03 4.25927175E+00
2.60143428E+03 4.21960572E+00
2.60286855E+03 4.21982525E+00
2.60717138E+03 4.22124713E+00
2.66714476E+03 4.16753260E+00
2.67144758E+03 4.13557428E+00
2.70143428E+03 4.11748331E+00
2.70286855E+03 4.11721505E+00
2.70717138E+03 4.11512864E+00
2.76714476E+03 4.10636551E+00
2.77144758E+03 4.08165758E+00
2.80143428E+03 4.02068553E+00
2.80286855E+03 4.02007072E+00
2.80717138E+03 4.01855708E+00
2.86714476E+03 4.06422017E+00
2.87144758E+03 3.99723513E+00
2.90143428E+03 4.00917499E+00
2.90286855E+03 4.00964741E+00
2.90717138E+03 4.01078946E+00
2.96714476E+03 3.96831937E+00
2.97144758E+03 3.93536142E+00
3.00143428E+03 3.87271680E+00
3.00286855E+03 3.87425934E+00
3.00717138E+03 3.87596454E+00
3.07144758E+03 3.87487641E+00
3.10143428E+03 3.83340129E+00
3.10286855E+03 3.83424118E+00
3.10717138E+03 3.83520962E+00
3.17144758E+03 3.76749077E+00
3.20286855E+03 3.72493404E+00
3.20717138E+03 3.71574700E+00
3.27144758E+03 3.68711032E+00
3.30286855E+03 3.62260351E+00
3.30717138E+03 3.61413880E+00
3.37144758E+03 3.60075958E+00

3.40717138E+03 3.56416797E+00
3.47144758E+03 3.52212378E+00
3.50717138E+03 3.47850726E+00
3.57144758E+03 3.39778877E+00
3.60717138E+03 3.39534512E+00
3.70717138E+03 3.34587184E+00
3.80717138E+03 3.27119353E+00
3.90717138E+03 3.20653075E+00

#Tot_ener(cm-1) 4--->3 Cross_sec(angs^2)
6.71448584E+02 6.30992354E+03
6.71449584E+02 3.63055984E+03
6.71450584E+02 2.60390054E+03
6.71451584E+02 2.06390818E+03
6.71452584E+02 1.73492110E+03
6.71453584E+02 1.51632726E+03
6.71454584E+02 1.36241437E+03
6.71455584E+02 1.24938894E+03
6.71456584E+02 1.16365178E+03
6.71457584E+02 1.09687871E+03
6.71467584E+02 8.20181755E+02
6.71477584E+02 7.15667381E+02
6.71487584E+02 6.38256668E+02
6.71497584E+02 5.74367608E+02
6.71507584E+02 5.22260199E+02
6.71517584E+02 4.80478503E+02
6.71527584E+02 4.47187625E+02
6.71537584E+02 4.20539964E+02
6.71547584E+02 3.98954191E+02
6.71647584E+02 2.94943261E+02
6.71747584E+02 2.52965322E+02
6.71847584E+02 2.37576801E+02
6.71947584E+02 2.39020553E+02
6.72047584E+02 2.48977584E+02
6.72147584E+02 2.54212698E+02
6.72247584E+02 2.47491823E+02
6.72347584E+02 2.34691025E+02
6.77447584E+02 1.05872868E+02
6.78447584E+02 1.01723641E+02
6.79447584E+02 9.47072780E+01
6.80447584E+02 8.93902481E+01
6.81447584E+02 8.83416023E+01
6.91447584E+02 6.95044225E+01
7.01434275E+02 5.42517438E+01
7.01447584E+02 5.42422966E+01
7.02868550E+02 5.33708878E+01
7.11447584E+02 4.97441831E+01
7.21447584E+02 4.61668625E+01
7.31447584E+02 4.04481898E+01
7.41447584E+02 3.75627922E+01

7.51447584E+02 3.75626811E+01
7.61447584E+02 3.38914864E+01
7.67144758E+02 3.29593923E+01
7.71447584E+02 3.22044974E+01
8.01434275E+02 2.94461629E+01
8.02868550E+02 2.94600846E+01
8.67144758E+02 2.42434837E+01
8.71447584E+02 2.40504168E+01
9.01434275E+02 2.25181948E+01
9.02868550E+02 2.23992573E+01
9.67144758E+02 2.05510342E+01
9.71447584E+02 2.07303871E+01
1.00143428E+03 1.80426993E+01
1.00286855E+03 1.80750224E+01
1.00717238E+03 1.83943391E+01
1.00717338E+03 1.83937147E+01
1.00717438E+03 1.83931116E+01
1.00717538E+03 1.83924993E+01
1.00717638E+03 1.83918719E+01
1.00717738E+03 1.83912299E+01
1.00717838E+03 1.83905746E+01
1.00717938E+03 1.83899070E+01
1.00718038E+03 1.83892313E+01
1.00718138E+03 1.83885500E+01
1.00719138E+03 1.83818531E+01
1.00720138E+03 1.83760344E+01
1.00721138E+03 1.83711710E+01
1.00722138E+03 1.83669669E+01
1.00723138E+03 1.83631447E+01
1.00724138E+03 1.83595285E+01
1.00725138E+03 1.83560289E+01
1.00726138E+03 1.83526169E+01
1.00727138E+03 1.83492962E+01
1.00737138E+03 1.83238791E+01
1.00747138E+03 1.83043517E+01
1.00757138E+03 1.82784051E+01
1.00767138E+03 1.82565262E+01
1.00777138E+03 1.82439371E+01
1.00787138E+03 1.82388602E+01
1.00797138E+03 1.82394134E+01
1.00807138E+03 1.82434554E+01
1.01317138E+03 1.77875660E+01
1.01417138E+03 1.75731870E+01
1.01517138E+03 1.73783069E+01
1.01617138E+03 1.72319123E+01
1.01717138E+03 1.70871935E+01
1.02717138E+03 1.67125585E+01
1.03717138E+03 1.65417712E+01
1.04717138E+03 1.60491450E+01
1.05717138E+03 1.58839022E+01

1.06714476E+03	1.52330417E+01
1.06717138E+03	1.52738989E+01
1.07144758E+03	1.47895730E+01
1.07717138E+03	1.44826923E+01
1.08717138E+03	1.41786749E+01
1.09717138E+03	1.39423256E+01
1.10143428E+03	1.39301527E+01
1.10286855E+03	1.39433450E+01
1.10717138E+03	1.38933881E+01
1.16714476E+03	1.29050447E+01
1.17144758E+03	1.26907105E+01
1.20143428E+03	1.21408767E+01
1.20286855E+03	1.21246063E+01
1.20717138E+03	1.20917232E+01
1.26714476E+03	1.15743192E+01
1.27144758E+03	1.14288646E+01
1.30143428E+03	1.12593943E+01
1.30286855E+03	1.12840323E+01
1.30717138E+03	1.11946543E+01
1.36714476E+03	1.07063247E+01
1.37144758E+03	1.05455617E+01
1.40143428E+03	1.07914519E+01
1.40286855E+03	1.07185361E+01
1.40717138E+03	1.07370618E+01
1.46714476E+03	1.01660883E+01
1.47144758E+03	1.02179764E+01
1.50143428E+03	1.00197041E+01
1.50286855E+03	9.99296903E+00
1.50717138E+03	9.89581170E+00
1.56714476E+03	9.79560143E+00
1.57144758E+03	9.80156564E+00
1.60143428E+03	9.92464817E+00
1.60286855E+03	9.92583684E+00
1.60717138E+03	9.90810126E+00
1.66714476E+03	9.63741076E+00
1.67144758E+03	9.55430464E+00
1.70143428E+03	9.47145060E+00
1.70286855E+03	9.49219064E+00
1.70717138E+03	9.55291385E+00
1.76714476E+03	9.60199560E+00
1.77144758E+03	9.63975003E+00
1.80143428E+03	9.47628047E+00
1.80286855E+03	9.47138222E+00
1.80717138E+03	9.45221292E+00
1.86714476E+03	9.41732565E+00
1.87144758E+03	9.35262202E+00
1.90143428E+03	9.35440241E+00
1.90286855E+03	9.34274632E+00
1.90717138E+03	9.32912691E+00
1.96714476E+03	9.01812829E+00

1.97144758E+03	8.98913409E+00
2.00143428E+03	9.00542482E+00
2.00286855E+03	9.00265677E+00
2.00717138E+03	9.00298782E+00
2.06714476E+03	9.00134747E+00
2.07144758E+03	8.97864127E+00
2.10143428E+03	8.74912182E+00
2.10286855E+03	8.74045767E+00
2.10717138E+03	8.71437670E+00
2.16714476E+03	8.65596769E+00
2.17144758E+03	8.63120753E+00
2.20143428E+03	8.64335501E+00
2.20286855E+03	8.63542435E+00
2.20717138E+03	8.67933740E+00
2.26714476E+03	8.63042638E+00
2.27144758E+03	8.59725461E+00
2.30143428E+03	8.55514639E+00
2.30286855E+03	8.55762606E+00
2.30717138E+03	8.56434498E+00
2.36714476E+03	8.56201828E+00
2.37144758E+03	8.57375320E+00
2.40143428E+03	8.57241993E+00
2.40286855E+03	8.57864621E+00
2.40717138E+03	8.59849904E+00
2.46714476E+03	8.46688836E+00
2.47144758E+03	8.42834413E+00
2.50143428E+03	8.42482817E+00
2.50286855E+03	8.42462152E+00
2.50717138E+03	8.41989744E+00
2.56714476E+03	8.32692576E+00
2.57144758E+03	8.30969951E+00
2.60143428E+03	8.30361083E+00
2.60286855E+03	8.30358666E+00
2.60717138E+03	8.29962676E+00
2.66714476E+03	8.24632097E+00
2.67144758E+03	8.21421507E+00
2.70143428E+03	8.13438351E+00
2.70286855E+03	8.13105081E+00
2.70717138E+03	8.12156866E+00
2.76714476E+03	8.10270820E+00
2.77144758E+03	8.08889094E+00
2.80143428E+03	8.07513814E+00
2.80286855E+03	8.07815725E+00
2.80717138E+03	8.08109349E+00
2.86714476E+03	7.99579877E+00
2.87144758E+03	7.98537943E+00
2.90143428E+03	7.94476496E+00
2.90286855E+03	7.94207440E+00
2.90717138E+03	7.93480908E+00
2.96714476E+03	7.87842416E+00

2.97144758E+03 7.86446629E+00
3.00143428E+03 7.87484406E+00
3.00286855E+03 7.87554979E+00
3.00717138E+03 7.87323161E+00
3.07144758E+03 7.78925528E+00
3.10143428E+03 7.84909804E+00
3.10286855E+03 7.84895824E+00
3.10717138E+03 7.84524285E+00
3.17144758E+03 7.73860586E+00
3.20286855E+03 7.72605896E+00
3.20717138E+03 7.72371708E+00
3.27144758E+03 7.70013794E+00
3.30286855E+03 7.72612014E+00
3.30717138E+03 7.74497269E+00
3.37144758E+03 7.72472930E+00
3.40717138E+03 7.66650874E+00
3.47144758E+03 7.71818361E+00
3.50717138E+03 7.71259330E+00
3.57144758E+03 7.71150971E+00
3.60717138E+03 7.70250081E+00
3.70717138E+03 7.74406171E+00
3.80717138E+03 7.67376603E+00
3.90717138E+03 7.74424475E+00

Table S12: Computed excitation and de-excitation rate coefficients in cm^3 $molecule^{-1}$ s^{-1} units for a series of inelastic processes generated using the 2D RR-PES for the $HeH^+(j_1)\cdots H_2$ system with $\Delta j_1 = +1$, $\Delta j_1 = +2$, $\Delta j_1 = -1$ and $\Delta j_1 = -2$.

T (K)	0--->1	0--->2	1--->2	1--->3
5.0	3.91519E-18	6.91464E-35	9.83475E-27	6.94340E-52
6.0	9.80566E-17	1.06735E-30	6.31899E-24	6.94445E-45
7.0	9.76852E-16	1.04611E-27	6.42399E-22	6.95764E-40
8.0	5.46055E-15	1.82880E-25	2.06057E-20	3.91984E-36
9.0	2.07607E-14	1.01302E-23	3.06213E-19	3.24077E-33
10.0	6.02769E-14	2.51040E-22	2.65513E-18	6.99284E-31
11.0	1.43885E-13	3.46660E-21	1.55575E-17	5.68103E-29
12.0	2.96627E-13	3.08792E-20	6.79366E-17	2.21851E-27
13.0	5.46409E-13	1.96352E-19	2.36602E-16	4.93175E-26
14.0	9.21480E-13	9.58139E-19	6.89795E-16	7.04207E-25
15.0	1.44823E-12	3.78342E-18	1.74437E-15	7.05705E-24
16.0	2.14958E-12	1.25798E-17	3.92963E-15	5.30413E-23
17.0	3.04406E-12	3.63076E-17	8.04852E-15	3.14577E-22
18.0	4.14537E-12	9.31363E-17	1.52271E-14	1.53148E-21
19.0	5.46245E-12	2.16341E-16	2.69462E-14	6.31397E-21
20.0	6.99980E-12	4.61880E-16	4.50507E-14	2.26011E-20
21.0	8.75796E-12	9.17343E-16	7.17387E-14	7.16722E-20
22.0	1.07341E-11	1.71170E-15	1.09529E-13	2.04708E-19
23.0	1.29227E-11	3.02522E-15	1.61217E-13	5.33823E-19
24.0	1.53158E-11	5.09871E-15	2.29812E-13	1.28549E-18
25.0	1.79041E-11	8.24163E-15	3.18479E-13	2.88598E-18
26.0	2.06767E-11	1.28382E-14	4.30469E-13	6.08936E-18

27.0	2.36222E-11	1.93517E-14	5.69052E-13	1.21587E-17
28.0	2.67284E-11	2.83256E-14	7.37460E-13	2.31098E-17
29.0	2.99831E-11	4.03832E-14	9.38831E-13	4.20248E-17
30.0	3.33740E-11	5.62242E-14	1.17616E-12	7.34389E-17
31.0	3.68888E-11	7.66198E-14	1.45225E-12	1.23801E-16
32.0	4.05158E-11	1.02405E-13	1.76970E-12	2.02000E-16
33.0	4.42435E-11	1.34471E-13	2.13087E-12	3.19960E-16
34.0	4.80608E-11	1.73755E-13	2.53786E-12	4.93271E-16
35.0	5.19572E-11	2.21231E-13	2.99250E-12	7.41861E-16
36.0	5.59227E-11	2.77896E-13	3.49635E-12	1.09068E-15
37.0	5.99480E-11	3.44766E-13	4.05071E-12	1.57039E-15
38.0	6.40243E-11	4.22858E-13	4.65660E-12	2.21801E-15
39.0	6.81432E-11	5.13185E-13	5.31479E-12	3.07755E-15
40.0	7.22970E-11	6.16747E-13	6.02581E-12	4.20056E-15
41.0	7.64786E-11	7.34519E-13	6.78995E-12	5.64666E-15
42.0	8.06813E-11	8.67446E-13	7.60728E-12	7.48391E-15
43.0	8.48991E-11	1.01644E-12	8.47766E-12	9.78915E-15
44.0	8.91261E-11	1.18236E-12	9.40079E-12	1.26482E-14
45.0	9.33572E-11	1.36603E-12	1.03762E-11	1.61561E-14
46.0	9.75877E-11	1.56822E-12	1.14032E-11	2.04168E-14
47.0	1.01813E-10	1.78963E-12	1.24810E-11	2.55437E-14
48.0	1.06030E-10	2.03093E-12	1.36088E-11	3.16587E-14
49.0	1.10233E-10	2.29271E-12	1.47854E-11	3.88926E-14
50.0	1.14421E-10	2.57551E-12	1.60099E-11	4.73842E-14
51.0	1.18590E-10	2.87981E-12	1.72810E-11	5.72804E-14
52.0	1.22737E-10	3.20603E-12	1.85974E-11	6.87352E-14
53.0	1.26860E-10	3.55453E-12	1.99579E-11	8.19096E-14
54.0	1.30957E-10	3.92561E-12	2.13610E-11	9.69707E-14
55.0	1.35026E-10	4.31953E-12	2.28054E-11	1.14091E-13
56.0	1.39065E-10	4.73648E-12	2.42896E-11	1.33448E-13
57.0	1.43073E-10	5.17661E-12	2.58123E-11	1.55223E-13
58.0	1.47048E-10	5.64000E-12	2.73718E-11	1.79602E-13
59.0	1.50989E-10	6.12671E-12	2.89667E-11	2.06772E-13
60.0	1.54895E-10	6.63674E-12	3.05956E-11	2.36922E-13
61.0	1.58765E-10	7.17004E-12	3.22570E-11	2.70244E-13
62.0	1.62600E-10	7.72654E-12	3.39494E-11	3.06930E-13
63.0	1.66397E-10	8.30613E-12	3.56714E-11	3.47171E-13
64.0	1.70156E-10	8.90864E-12	3.74215E-11	3.91158E-13
65.0	1.73878E-10	9.53389E-12	3.91983E-11	4.39080E-13
66.0	1.77562E-10	1.01817E-11	4.10004E-11	4.91125E-13
67.0	1.81207E-10	1.08518E-11	4.28264E-11	5.47478E-13
68.0	1.84814E-10	1.15438E-11	4.46751E-11	6.08321E-13
69.0	1.88382E-10	1.22577E-11	4.65450E-11	6.73832E-13
70.0	1.91912E-10	1.29929E-11	4.84349E-11	7.44186E-13
71.0	1.95403E-10	1.37493E-11	5.03436E-11	8.19553E-13
72.0	1.98857E-10	1.45263E-11	5.22698E-11	9.00099E-13
73.0	2.02272E-10	1.53238E-11	5.42123E-11	9.85982E-13
74.0	2.05649E-10	1.61413E-11	5.61700E-11	1.07736E-12
75.0	2.08988E-10	1.69783E-11	5.81417E-11	1.17438E-12
76.0	2.12290E-10	1.78347E-11	6.01264E-11	1.27718E-12

77.0	2.15555E-10	1.87098E-11	6.21230E-11	1.38591E-12
78.0	2.18783E-10	1.96033E-11	6.41304E-11	1.50069E-12
79.0	2.21975E-10	2.05148E-11	6.61477E-11	1.62164E-12
80.0	2.25131E-10	2.14439E-11	6.81739E-11	1.74889E-12
81.0	2.28252E-10	2.23902E-11	7.02081E-11	1.88254E-12
82.0	2.31337E-10	2.33531E-11	7.22494E-11	2.02271E-12
83.0	2.34388E-10	2.43323E-11	7.42970E-11	2.16948E-12
84.0	2.37404E-10	2.53273E-11	7.63499E-11	2.32295E-12
85.0	2.40387E-10	2.63378E-11	7.84075E-11	2.48319E-12
86.0	2.43337E-10	2.73632E-11	8.04689E-11	2.65030E-12
87.0	2.46254E-10	2.84032E-11	8.25334E-11	2.82434E-12
88.0	2.49138E-10	2.94573E-11	8.46003E-11	3.00537E-12
89.0	2.51991E-10	3.05252E-11	8.66689E-11	3.19345E-12
90.0	2.54812E-10	3.16062E-11	8.87385E-11	3.38863E-12
91.0	2.57602E-10	3.27002E-11	9.08085E-11	3.59095E-12
92.0	2.60362E-10	3.38066E-11	9.28784E-11	3.80046E-12
93.0	2.63092E-10	3.49250E-11	9.49475E-11	4.01719E-12
94.0	2.65793E-10	3.60551E-11	9.70153E-11	4.24116E-12
95.0	2.68465E-10	3.71964E-11	9.90813E-11	4.47239E-12
96.0	2.71108E-10	3.83485E-11	1.01145E-10	4.71090E-12
97.0	2.73724E-10	3.95110E-11	1.03206E-10	4.95670E-12
98.0	2.76312E-10	4.06837E-11	1.05263E-10	5.20979E-12
99.0	2.78873E-10	4.18660E-11	1.07317E-10	5.47017E-12
100.0	2.81407E-10	4.30576E-11	1.09367E-10	5.73783E-12
101.0	2.83915E-10	4.42582E-11	1.11412E-10	6.01277E-12
102.0	2.86398E-10	4.54674E-11	1.13452E-10	6.29496E-12
103.0	2.88855E-10	4.66849E-11	1.15487E-10	6.58439E-12
104.0	2.91288E-10	4.79102E-11	1.17516E-10	6.88104E-12
105.0	2.93697E-10	4.91431E-11	1.19540E-10	7.18486E-12
106.0	2.96081E-10	5.03832E-11	1.21557E-10	7.49584E-12
107.0	2.98442E-10	5.16303E-11	1.23568E-10	7.81393E-12
108.0	3.00780E-10	5.28839E-11	1.25571E-10	8.13909E-12
109.0	3.03096E-10	5.41438E-11	1.27568E-10	8.47128E-12
110.0	3.05389E-10	5.54097E-11	1.29558E-10	8.81046E-12
111.0	3.07660E-10	5.66812E-11	1.31539E-10	9.15657E-12
112.0	3.09910E-10	5.79582E-11	1.33514E-10	9.50955E-12
113.0	3.12139E-10	5.92402E-11	1.35480E-10	9.86936E-12
114.0	3.14348E-10	6.05271E-11	1.37438E-10	1.02359E-11
115.0	3.16536E-10	6.18185E-11	1.39387E-10	1.06092E-11
116.0	3.18704E-10	6.31141E-11	1.41328E-10	1.09891E-11
117.0	3.20852E-10	6.44138E-11	1.43261E-10	1.13756E-11
118.0	3.22981E-10	6.57173E-11	1.45184E-10	1.17686E-11
119.0	3.25092E-10	6.70243E-11	1.47099E-10	1.21680E-11
120.0	3.27184E-10	6.83346E-11	1.49004E-10	1.25738E-11
121.0	3.29257E-10	6.96479E-11	1.50901E-10	1.29859E-11
122.0	3.31313E-10	7.09640E-11	1.52787E-10	1.34042E-11
123.0	3.33351E-10	7.22828E-11	1.54665E-10	1.38286E-11
124.0	3.35372E-10	7.36039E-11	1.56533E-10	1.42591E-11
125.0	3.37376E-10	7.49271E-11	1.58391E-10	1.46955E-11
126.0	3.39363E-10	7.62523E-11	1.60240E-10	1.51379E-11

127.0	3.41334E-10	7.75793E-11	1.62079E-10	1.55860E-11
128.0	3.43289E-10	7.89078E-11	1.63908E-10	1.60399E-11
129.0	3.45228E-10	8.02377E-11	1.65728E-10	1.64995E-11
130.0	3.47152E-10	8.15688E-11	1.67537E-10	1.69646E-11
131.0	3.49060E-10	8.29009E-11	1.69336E-10	1.74352E-11
132.0	3.50953E-10	8.42338E-11	1.71126E-10	1.79111E-11
133.0	3.52832E-10	8.55673E-11	1.72905E-10	1.83924E-11
134.0	3.54696E-10	8.69013E-11	1.74674E-10	1.88789E-11
135.0	3.56546E-10	8.82357E-11	1.76434E-10	1.93705E-11
136.0	3.58382E-10	8.95702E-11	1.78183E-10	1.98672E-11
137.0	3.60204E-10	9.09048E-11	1.79922E-10	2.03689E-11
138.0	3.62013E-10	9.22392E-11	1.81650E-10	2.08754E-11
139.0	3.63808E-10	9.35733E-11	1.83369E-10	2.13866E-11
140.0	3.65590E-10	9.49070E-11	1.85078E-10	2.19026E-11
141.0	3.67360E-10	9.62401E-11	1.86776E-10	2.24232E-11
142.0	3.69117E-10	9.75726E-11	1.88465E-10	2.29483E-11
143.0	3.70861E-10	9.89042E-11	1.90143E-10	2.34779E-11
144.0	3.72593E-10	1.00235E-10	1.91811E-10	2.40118E-11
145.0	3.74314E-10	1.01565E-10	1.93469E-10	2.45499E-11
146.0	3.76022E-10	1.02893E-10	1.95117E-10	2.50922E-11
147.0	3.77719E-10	1.04220E-10	1.96755E-10	2.56386E-11
148.0	3.79404E-10	1.05546E-10	1.98383E-10	2.61890E-11
149.0	3.81078E-10	1.06870E-10	2.00001E-10	2.67433E-11
150.0	3.82741E-10	1.08193E-10	2.01609E-10	2.73015E-11
151.0	3.84393E-10	1.09514E-10	2.03207E-10	2.78634E-11
152.0	3.86035E-10	1.10833E-10	2.04795E-10	2.84289E-11
153.0	3.87666E-10	1.12150E-10	2.06374E-10	2.89981E-11
154.0	3.89286E-10	1.13465E-10	2.07942E-10	2.95707E-11
155.0	3.90897E-10	1.14778E-10	2.09501E-10	3.01467E-11
156.0	3.92497E-10	1.16089E-10	2.11050E-10	3.07261E-11
157.0	3.94087E-10	1.17398E-10	2.12590E-10	3.13087E-11
158.0	3.95668E-10	1.18704E-10	2.14120E-10	3.18945E-11
159.0	3.97239E-10	1.20008E-10	2.15640E-10	3.24834E-11
160.0	3.98800E-10	1.21309E-10	2.17151E-10	3.30753E-11
161.0	4.00352E-10	1.22608E-10	2.18652E-10	3.36701E-11
162.0	4.01895E-10	1.23904E-10	2.20144E-10	3.42678E-11
163.0	4.03429E-10	1.25198E-10	2.21626E-10	3.48683E-11
164.0	4.04954E-10	1.26489E-10	2.23099E-10	3.54715E-11
165.0	4.06471E-10	1.27777E-10	2.24563E-10	3.60773E-11
166.0	4.07978E-10	1.29062E-10	2.26017E-10	3.66857E-11
167.0	4.09478E-10	1.30344E-10	2.27463E-10	3.72965E-11
168.0	4.10968E-10	1.31623E-10	2.28899E-10	3.79098E-11
169.0	4.12451E-10	1.32899E-10	2.30326E-10	3.85254E-11
170.0	4.13925E-10	1.34171E-10	2.31745E-10	3.91432E-11
171.0	4.15391E-10	1.35441E-10	2.33154E-10	3.97633E-11
172.0	4.16850E-10	1.36708E-10	2.34554E-10	4.03855E-11
173.0	4.18300E-10	1.37971E-10	2.35946E-10	4.10098E-11
174.0	4.19743E-10	1.39231E-10	2.37329E-10	4.16361E-11
175.0	4.21178E-10	1.40487E-10	2.38703E-10	4.22642E-11
176.0	4.22606E-10	1.41740E-10	2.40068E-10	4.28943E-11

177.0	4.24026E-10	1.42990E-10	2.41425E-10	4.35262E-11
178.0	4.25439E-10	1.44236E-10	2.42774E-10	4.41597E-11
179.0	4.26845E-10	1.45479E-10	2.44113E-10	4.47950E-11
180.0	4.28243E-10	1.46718E-10	2.45445E-10	4.54319E-11
181.0	4.29635E-10	1.47953E-10	2.46768E-10	4.60703E-11
182.0	4.31020E-10	1.49185E-10	2.48083E-10	4.67102E-11
183.0	4.32398E-10	1.50414E-10	2.49390E-10	4.73516E-11
184.0	4.33769E-10	1.51638E-10	2.50688E-10	4.79943E-11
185.0	4.35133E-10	1.52859E-10	2.51979E-10	4.86383E-11
186.0	4.36491E-10	1.54076E-10	2.53261E-10	4.92836E-11
187.0	4.37843E-10	1.55290E-10	2.54536E-10	4.99301E-11
188.0	4.39188E-10	1.56499E-10	2.55802E-10	5.05777E-11
189.0	4.40526E-10	1.57705E-10	2.57061E-10	5.12264E-11
190.0	4.41859E-10	1.58907E-10	2.58312E-10	5.18762E-11
191.0	4.43185E-10	1.60105E-10	2.59555E-10	5.25269E-11
192.0	4.44505E-10	1.61299E-10	2.60790E-10	5.31786E-11
193.0	4.45820E-10	1.62490E-10	2.62018E-10	5.38312E-11
194.0	4.47128E-10	1.63676E-10	2.63239E-10	5.44846E-11
195.0	4.48430E-10	1.64858E-10	2.64451E-10	5.51388E-11
196.0	4.49727E-10	1.66037E-10	2.65657E-10	5.57937E-11
197.0	4.51017E-10	1.67211E-10	2.66855E-10	5.64493E-11
198.0	4.52302E-10	1.68382E-10	2.68046E-10	5.71055E-11
199.0	4.53582E-10	1.69549E-10	2.69229E-10	5.77624E-11
200.0	4.54856E-10	1.70711E-10	2.70406E-10	5.84198E-11
201.0	4.56124E-10	1.71870E-10	2.71575E-10	5.90777E-11
202.0	4.57387E-10	1.73024E-10	2.72737E-10	5.97360E-11
203.0	4.58645E-10	1.74175E-10	2.73892E-10	6.03948E-11
204.0	4.59897E-10	1.75321E-10	2.75040E-10	6.10540E-11
205.0	4.61144E-10	1.76464E-10	2.76181E-10	6.17135E-11
206.0	4.62386E-10	1.77602E-10	2.77316E-10	6.23732E-11
207.0	4.63622E-10	1.78736E-10	2.78444E-10	6.30333E-11
208.0	4.64854E-10	1.79867E-10	2.79564E-10	6.36935E-11
209.0	4.66080E-10	1.80993E-10	2.80679E-10	6.43540E-11
210.0	4.67302E-10	1.82115E-10	2.81786E-10	6.50145E-11
211.0	4.68519E-10	1.83233E-10	2.82887E-10	6.56752E-11
212.0	4.69730E-10	1.84347E-10	2.83982E-10	6.63359E-11
213.0	4.70937E-10	1.85456E-10	2.85070E-10	6.69967E-11
214.0	4.72139E-10	1.86562E-10	2.86152E-10	6.76574E-11
215.0	4.73337E-10	1.87664E-10	2.87227E-10	6.83181E-11
216.0	4.74530E-10	1.88761E-10	2.88297E-10	6.89788E-11
217.0	4.75718E-10	1.89854E-10	2.89359E-10	6.96393E-11
218.0	4.76901E-10	1.90944E-10	2.90416E-10	7.02997E-11
219.0	4.78080E-10	1.92029E-10	2.91467E-10	7.09599E-11
220.0	4.79254E-10	1.93110E-10	2.92511E-10	7.16199E-11
221.0	4.80424E-10	1.94187E-10	2.93550E-10	7.22797E-11
222.0	4.81590E-10	1.95260E-10	2.94582E-10	7.29392E-11
223.0	4.82751E-10	1.96328E-10	2.95609E-10	7.35984E-11
224.0	4.83908E-10	1.97393E-10	2.96630E-10	7.42573E-11
225.0	4.85060E-10	1.98454E-10	2.97644E-10	7.49158E-11
226.0	4.86208E-10	1.99510E-10	2.98654E-10	7.55740E-11

227.0	4.87352E-10	2.00563E-10	2.99657E-10	7.62318E-11
228.0	4.88492E-10	2.01611E-10	3.00655E-10	7.68891E-11
229.0	4.89627E-10	2.02655E-10	3.01647E-10	7.75459E-11
230.0	4.90759E-10	2.03695E-10	3.02633E-10	7.82023E-11
231.0	4.91886E-10	2.04732E-10	3.03614E-10	7.88582E-11
232.0	4.93010E-10	2.05764E-10	3.04590E-10	7.95135E-11
233.0	4.94129E-10	2.06792E-10	3.05560E-10	8.01683E-11
234.0	4.95244E-10	2.07816E-10	3.06525E-10	8.08225E-11
235.0	4.96355E-10	2.08836E-10	3.07484E-10	8.14760E-11
236.0	4.97463E-10	2.09852E-10	3.08438E-10	8.21290E-11
237.0	4.98566E-10	2.10864E-10	3.09387E-10	8.27813E-11
238.0	4.99666E-10	2.11871E-10	3.10331E-10	8.34329E-11
239.0	5.00762E-10	2.12875E-10	3.11269E-10	8.40839E-11
240.0	5.01854E-10	2.13875E-10	3.12202E-10	8.47341E-11
241.0	5.02942E-10	2.14871E-10	3.13131E-10	8.53836E-11
242.0	5.04027E-10	2.15863E-10	3.14054E-10	8.60323E-11
243.0	5.05107E-10	2.16851E-10	3.14972E-10	8.66803E-11
244.0	5.06184E-10	2.17835E-10	3.15885E-10	8.73275E-11
245.0	5.07258E-10	2.18815E-10	3.16794E-10	8.79738E-11
246.0	5.08328E-10	2.19791E-10	3.17697E-10	8.86194E-11
247.0	5.09394E-10	2.20764E-10	3.18596E-10	8.92641E-11
248.0	5.10457E-10	2.21732E-10	3.19490E-10	8.99079E-11
249.0	5.11516E-10	2.22696E-10	3.20379E-10	9.05509E-11
250.0	5.12571E-10	2.23657E-10	3.21264E-10	9.11929E-11
251.0	5.13623E-10	2.24613E-10	3.22143E-10	9.18341E-11
252.0	5.14672E-10	2.25566E-10	3.23019E-10	9.24743E-11
253.0	5.15717E-10	2.26515E-10	3.23889E-10	9.31136E-11
254.0	5.16759E-10	2.27460E-10	3.24755E-10	9.37519E-11
255.0	5.17797E-10	2.28401E-10	3.25617E-10	9.43893E-11
256.0	5.18832E-10	2.29338E-10	3.26474E-10	9.50256E-11
257.0	5.19863E-10	2.30272E-10	3.27327E-10	9.56610E-11
258.0	5.20891E-10	2.31201E-10	3.28175E-10	9.62954E-11
259.0	5.21916E-10	2.32127E-10	3.29019E-10	9.69287E-11
260.0	5.22938E-10	2.33049E-10	3.29859E-10	9.75610E-11
261.0	5.23956E-10	2.33968E-10	3.30694E-10	9.81922E-11
262.0	5.24971E-10	2.34882E-10	3.31525E-10	9.88224E-11
263.0	5.25983E-10	2.35793E-10	3.32352E-10	9.94515E-11
264.0	5.26991E-10	2.36700E-10	3.33175E-10	1.00080E-10
265.0	5.27997E-10	2.37604E-10	3.33994E-10	1.00706E-10
266.0	5.28999E-10	2.38503E-10	3.34808E-10	1.01332E-10
267.0	5.29998E-10	2.39399E-10	3.35619E-10	1.01957E-10
268.0	5.30994E-10	2.40291E-10	3.36425E-10	1.02581E-10
269.0	5.31987E-10	2.41180E-10	3.37227E-10	1.03203E-10
270.0	5.32976E-10	2.42065E-10	3.38026E-10	1.03824E-10
271.0	5.33963E-10	2.42946E-10	3.38820E-10	1.04444E-10
272.0	5.34947E-10	2.43824E-10	3.39611E-10	1.05063E-10
273.0	5.35927E-10	2.44698E-10	3.40398E-10	1.05681E-10
274.0	5.36905E-10	2.45569E-10	3.41181E-10	1.06298E-10
275.0	5.37879E-10	2.46436E-10	3.41960E-10	1.06913E-10
276.0	5.38850E-10	2.47299E-10	3.42735E-10	1.07527E-10

277.0	5.39819E-10	2.48159E-10	3.43507E-10	1.08140E-10
278.0	5.40784E-10	2.49015E-10	3.44274E-10	1.08752E-10
279.0	5.41747E-10	2.49868E-10	3.45039E-10	1.09363E-10
280.0	5.42707E-10	2.50717E-10	3.45799E-10	1.09972E-10
281.0	5.43663E-10	2.51562E-10	3.46556E-10	1.10580E-10
282.0	5.44617E-10	2.52405E-10	3.47309E-10	1.11187E-10
283.0	5.45568E-10	2.53243E-10	3.48059E-10	1.11793E-10
284.0	5.46516E-10	2.54078E-10	3.48805E-10	1.12397E-10
285.0	5.47462E-10	2.54910E-10	3.49548E-10	1.13000E-10
286.0	5.48404E-10	2.55739E-10	3.50287E-10	1.13602E-10
287.0	5.49344E-10	2.56564E-10	3.51023E-10	1.14202E-10
288.0	5.50280E-10	2.57385E-10	3.51755E-10	1.14802E-10
289.0	5.51214E-10	2.58203E-10	3.52484E-10	1.15400E-10
290.0	5.52145E-10	2.59018E-10	3.53210E-10	1.15996E-10
291.0	5.53074E-10	2.59829E-10	3.53932E-10	1.16592E-10
292.0	5.54000E-10	2.60637E-10	3.54651E-10	1.17186E-10
293.0	5.54923E-10	2.61442E-10	3.55367E-10	1.17778E-10
294.0	5.55843E-10	2.62243E-10	3.56079E-10	1.18370E-10
295.0	5.56760E-10	2.63041E-10	3.56789E-10	1.18960E-10
296.0	5.57675E-10	2.63836E-10	3.57495E-10	1.19549E-10
297.0	5.58587E-10	2.64628E-10	3.58197E-10	1.20137E-10
298.0	5.59497E-10	2.65416E-10	3.58897E-10	1.20723E-10
299.0	5.60404E-10	2.66201E-10	3.59594E-10	1.21308E-10
300.0	5.61308E-10	2.66983E-10	3.60287E-10	1.21891E-10
301.0	5.62209E-10	2.67761E-10	3.60977E-10	1.22473E-10
302.0	5.63108E-10	2.68537E-10	3.61665E-10	1.23054E-10
303.0	5.64005E-10	2.69309E-10	3.62349E-10	1.23634E-10
304.0	5.64898E-10	2.70078E-10	3.63030E-10	1.24212E-10
305.0	5.65789E-10	2.70843E-10	3.63708E-10	1.24789E-10
306.0	5.66678E-10	2.71606E-10	3.64384E-10	1.25364E-10
307.0	5.67564E-10	2.72366E-10	3.65056E-10	1.25939E-10
308.0	5.68447E-10	2.73122E-10	3.65726E-10	1.26511E-10
309.0	5.69328E-10	2.73875E-10	3.66392E-10	1.27083E-10
310.0	5.70207E-10	2.74625E-10	3.67056E-10	1.27653E-10
311.0	5.71083E-10	2.75373E-10	3.67717E-10	1.28222E-10
312.0	5.71956E-10	2.76117E-10	3.68375E-10	1.28789E-10
313.0	5.72827E-10	2.76858E-10	3.69030E-10	1.29355E-10
314.0	5.73695E-10	2.77596E-10	3.69682E-10	1.29920E-10
315.0	5.74561E-10	2.78330E-10	3.70332E-10	1.30483E-10
316.0	5.75425E-10	2.79062E-10	3.70979E-10	1.31045E-10
317.0	5.76286E-10	2.79791E-10	3.71623E-10	1.31606E-10
318.0	5.77144E-10	2.80517E-10	3.72265E-10	1.32165E-10
319.0	5.78000E-10	2.81240E-10	3.72904E-10	1.32723E-10
320.0	5.78854E-10	2.81960E-10	3.73540E-10	1.33280E-10
321.0	5.79706E-10	2.82677E-10	3.74173E-10	1.33835E-10
322.0	5.80554E-10	2.83391E-10	3.74804E-10	1.34389E-10
323.0	5.81401E-10	2.84103E-10	3.75433E-10	1.34941E-10
324.0	5.82245E-10	2.84811E-10	3.76059E-10	1.35492E-10
325.0	5.83087E-10	2.85516E-10	3.76682E-10	1.36042E-10
326.0	5.83927E-10	2.86219E-10	3.77303E-10	1.36590E-10

327.0	5.84764E-10	2.86919E-10	3.77921E-10	1.37137E-10
328.0	5.85598E-10	2.87616E-10	3.78537E-10	1.37683E-10
329.0	5.86431E-10	2.88310E-10	3.79150E-10	1.38227E-10
330.0	5.87261E-10	2.89001E-10	3.79761E-10	1.38770E-10
331.0	5.88089E-10	2.89689E-10	3.80369E-10	1.39311E-10
332.0	5.88914E-10	2.90375E-10	3.80975E-10	1.39851E-10
333.0	5.89738E-10	2.91058E-10	3.81578E-10	1.40390E-10
334.0	5.90559E-10	2.91738E-10	3.82180E-10	1.40928E-10
335.0	5.91377E-10	2.92415E-10	3.82778E-10	1.41464E-10
336.0	5.92194E-10	2.93090E-10	3.83375E-10	1.41998E-10
337.0	5.93008E-10	2.93762E-10	3.83969E-10	1.42532E-10
338.0	5.93820E-10	2.94431E-10	3.84561E-10	1.43064E-10
339.0	5.94630E-10	2.95097E-10	3.85150E-10	1.43594E-10
340.0	5.95437E-10	2.95761E-10	3.85737E-10	1.44124E-10
341.0	5.96242E-10	2.96422E-10	3.86322E-10	1.44651E-10
342.0	5.97045E-10	2.97081E-10	3.86905E-10	1.45178E-10
343.0	5.97846E-10	2.97736E-10	3.87485E-10	1.45703E-10
344.0	5.98645E-10	2.98390E-10	3.88064E-10	1.46227E-10
345.0	5.99441E-10	2.99040E-10	3.88640E-10	1.46750E-10
346.0	6.00236E-10	2.99688E-10	3.89214E-10	1.47271E-10
347.0	6.01028E-10	3.00333E-10	3.89785E-10	1.47791E-10
348.0	6.01818E-10	3.00976E-10	3.90355E-10	1.48309E-10
349.0	6.02606E-10	3.01616E-10	3.90922E-10	1.48826E-10
350.0	6.03392E-10	3.02254E-10	3.91488E-10	1.49342E-10
351.0	6.04175E-10	3.02889E-10	3.92051E-10	1.49856E-10
352.0	6.04957E-10	3.03522E-10	3.92612E-10	1.50369E-10
353.0	6.05736E-10	3.04152E-10	3.93171E-10	1.50881E-10
354.0	6.06513E-10	3.04779E-10	3.93728E-10	1.51392E-10
355.0	6.07288E-10	3.05404E-10	3.94283E-10	1.51901E-10
356.0	6.08061E-10	3.06027E-10	3.94836E-10	1.52409E-10
357.0	6.08832E-10	3.06647E-10	3.95387E-10	1.52915E-10
358.0	6.09601E-10	3.07265E-10	3.95936E-10	1.53420E-10
359.0	6.10368E-10	3.07880E-10	3.96482E-10	1.53924E-10
360.0	6.11133E-10	3.08492E-10	3.97027E-10	1.54426E-10
361.0	6.11896E-10	3.09103E-10	3.97570E-10	1.54928E-10
362.0	6.12656E-10	3.09711E-10	3.98111E-10	1.55427E-10
363.0	6.13415E-10	3.10316E-10	3.98651E-10	1.55926E-10
364.0	6.14172E-10	3.10919E-10	3.99188E-10	1.56423E-10
365.0	6.14926E-10	3.11520E-10	3.99723E-10	1.56919E-10
366.0	6.15679E-10	3.12118E-10	4.00257E-10	1.57414E-10
367.0	6.16430E-10	3.12714E-10	4.00788E-10	1.57907E-10
368.0	6.17178E-10	3.13308E-10	4.01318E-10	1.58399E-10
369.0	6.17925E-10	3.13900E-10	4.01846E-10	1.58890E-10
370.0	6.18669E-10	3.14489E-10	4.02372E-10	1.59379E-10
371.0	6.19412E-10	3.15075E-10	4.02896E-10	1.59867E-10
372.0	6.20153E-10	3.15660E-10	4.03418E-10	1.60354E-10
373.0	6.20892E-10	3.16242E-10	4.03939E-10	1.60839E-10
374.0	6.21628E-10	3.16822E-10	4.04458E-10	1.61324E-10
375.0	6.22363E-10	3.17399E-10	4.04975E-10	1.61807E-10
376.0	6.23096E-10	3.17975E-10	4.05490E-10	1.62288E-10

377.0	6.23827E-10	3.18548E-10	4.06003E-10	1.62769E-10
378.0	6.24556E-10	3.19119E-10	4.06515E-10	1.63248E-10
379.0	6.25283E-10	3.19688E-10	4.07025E-10	1.63726E-10
380.0	6.26009E-10	3.20254E-10	4.07533E-10	1.64202E-10
381.0	6.26732E-10	3.20818E-10	4.08040E-10	1.64677E-10
382.0	6.27454E-10	3.21381E-10	4.08545E-10	1.65152E-10
383.0	6.28173E-10	3.21940E-10	4.09048E-10	1.65624E-10
384.0	6.28891E-10	3.22498E-10	4.09550E-10	1.66096E-10
385.0	6.29607E-10	3.23054E-10	4.10050E-10	1.66566E-10
386.0	6.30321E-10	3.23607E-10	4.10548E-10	1.67035E-10
387.0	6.31033E-10	3.24159E-10	4.11045E-10	1.67503E-10
388.0	6.31743E-10	3.24708E-10	4.11540E-10	1.67969E-10
389.0	6.32451E-10	3.25255E-10	4.12033E-10	1.68435E-10
390.0	6.33158E-10	3.25800E-10	4.12525E-10	1.68899E-10
391.0	6.33863E-10	3.26343E-10	4.13015E-10	1.69362E-10
392.0	6.34566E-10	3.26884E-10	4.13504E-10	1.69823E-10
393.0	6.35267E-10	3.27423E-10	4.13991E-10	1.70284E-10
394.0	6.35966E-10	3.27960E-10	4.14477E-10	1.70743E-10
395.0	6.36663E-10	3.28494E-10	4.14961E-10	1.71201E-10
396.0	6.37359E-10	3.29027E-10	4.15443E-10	1.71657E-10
397.0	6.38053E-10	3.29558E-10	4.15924E-10	1.72113E-10
398.0	6.38745E-10	3.30086E-10	4.16404E-10	1.72567E-10
399.0	6.39435E-10	3.30613E-10	4.16882E-10	1.73020E-10
400.0	6.40124E-10	3.31137E-10	4.17358E-10	1.73472E-10
401.0	6.40811E-10	3.31660E-10	4.17833E-10	1.73922E-10
402.0	6.41496E-10	3.32181E-10	4.18306E-10	1.74372E-10
403.0	6.42179E-10	3.32699E-10	4.18778E-10	1.74820E-10
404.0	6.42861E-10	3.33216E-10	4.19249E-10	1.75267E-10
405.0	6.43540E-10	3.33731E-10	4.19718E-10	1.75713E-10
406.0	6.44219E-10	3.34244E-10	4.20186E-10	1.76158E-10
407.0	6.44895E-10	3.34755E-10	4.20652E-10	1.76601E-10
408.0	6.45570E-10	3.35264E-10	4.21117E-10	1.77043E-10
409.0	6.46242E-10	3.35771E-10	4.21580E-10	1.77485E-10
410.0	6.46914E-10	3.36276E-10	4.22042E-10	1.77924E-10
411.0	6.47583E-10	3.36779E-10	4.22503E-10	1.78363E-10
412.0	6.48251E-10	3.37281E-10	4.22962E-10	1.78801E-10
413.0	6.48917E-10	3.37780E-10	4.23420E-10	1.79237E-10
414.0	6.49582E-10	3.38278E-10	4.23876E-10	1.79673E-10
415.0	6.50244E-10	3.38774E-10	4.24331E-10	1.80107E-10
416.0	6.50905E-10	3.39268E-10	4.24785E-10	1.80540E-10
417.0	6.51565E-10	3.39760E-10	4.25237E-10	1.80971E-10
418.0	6.52223E-10	3.40250E-10	4.25688E-10	1.81402E-10
419.0	6.52879E-10	3.40739E-10	4.26138E-10	1.81832E-10
420.0	6.53533E-10	3.41225E-10	4.26587E-10	1.82260E-10
421.0	6.54186E-10	3.41710E-10	4.27034E-10	1.82687E-10
422.0	6.54837E-10	3.42193E-10	4.27480E-10	1.83113E-10
423.0	6.55487E-10	3.42675E-10	4.27924E-10	1.83538E-10
424.0	6.56135E-10	3.43154E-10	4.28367E-10	1.83962E-10
425.0	6.56781E-10	3.43632E-10	4.28809E-10	1.84385E-10
426.0	6.57426E-10	3.44108E-10	4.29250E-10	1.84806E-10

427.0	6.58069E-10	3.44582E-10	4.29690E-10	1.85227E-10
428.0	6.58710E-10	3.45055E-10	4.30128E-10	1.85646E-10
429.0	6.59350E-10	3.45526E-10	4.30565E-10	1.86065E-10
430.0	6.59988E-10	3.45995E-10	4.31001E-10	1.86482E-10
431.0	6.60625E-10	3.46462E-10	4.31435E-10	1.86898E-10
432.0	6.61260E-10	3.46928E-10	4.31868E-10	1.87313E-10
433.0	6.61894E-10	3.47392E-10	4.32300E-10	1.87727E-10
434.0	6.62526E-10	3.47854E-10	4.32731E-10	1.88139E-10
435.0	6.63156E-10	3.48315E-10	4.33161E-10	1.88551E-10
436.0	6.63785E-10	3.48773E-10	4.33589E-10	1.88962E-10
437.0	6.64413E-10	3.49231E-10	4.34017E-10	1.89371E-10
438.0	6.65039E-10	3.49686E-10	4.34443E-10	1.89779E-10
439.0	6.65663E-10	3.50140E-10	4.34868E-10	1.90187E-10
440.0	6.66286E-10	3.50593E-10	4.35291E-10	1.90593E-10
441.0	6.66907E-10	3.51043E-10	4.35714E-10	1.90998E-10
442.0	6.67527E-10	3.51492E-10	4.36135E-10	1.91402E-10
443.0	6.68145E-10	3.51940E-10	4.36556E-10	1.91805E-10
444.0	6.68761E-10	3.52386E-10	4.36975E-10	1.92207E-10
445.0	6.69377E-10	3.52830E-10	4.37393E-10	1.92608E-10
446.0	6.69990E-10	3.53272E-10	4.37810E-10	1.93008E-10
447.0	6.70602E-10	3.53713E-10	4.38226E-10	1.93407E-10
448.0	6.71213E-10	3.54153E-10	4.38640E-10	1.93805E-10
449.0	6.71822E-10	3.54591E-10	4.39054E-10	1.94202E-10
450.0	6.72430E-10	3.55027E-10	4.39467E-10	1.94597E-10
451.0	6.73036E-10	3.55462E-10	4.39878E-10	1.94992E-10
452.0	6.73641E-10	3.55895E-10	4.40288E-10	1.95386E-10
453.0	6.74245E-10	3.56327E-10	4.40697E-10	1.95778E-10
454.0	6.74846E-10	3.56757E-10	4.41106E-10	1.96170E-10
455.0	6.75447E-10	3.57185E-10	4.41513E-10	1.96560E-10
456.0	6.76046E-10	3.57612E-10	4.41919E-10	1.96950E-10
457.0	6.76643E-10	3.58038E-10	4.42324E-10	1.97338E-10
458.0	6.77239E-10	3.58462E-10	4.42728E-10	1.97726E-10
459.0	6.77834E-10	3.58885E-10	4.43131E-10	1.98112E-10
460.0	6.78427E-10	3.59306E-10	4.43533E-10	1.98498E-10
461.0	6.79019E-10	3.59725E-10	4.43933E-10	1.98882E-10
462.0	6.79610E-10	3.60143E-10	4.44333E-10	1.99266E-10
463.0	6.80199E-10	3.60560E-10	4.44732E-10	1.99648E-10
464.0	6.80786E-10	3.60975E-10	4.45130E-10	2.00030E-10
465.0	6.81372E-10	3.61389E-10	4.45526E-10	2.00410E-10
466.0	6.81957E-10	3.61801E-10	4.45922E-10	2.00790E-10
467.0	6.82540E-10	3.62212E-10	4.46317E-10	2.01168E-10
468.0	6.83122E-10	3.62621E-10	4.46711E-10	2.01546E-10
469.0	6.83703E-10	3.63029E-10	4.47104E-10	2.01922E-10
470.0	6.84282E-10	3.63436E-10	4.47495E-10	2.02298E-10
471.0	6.84860E-10	3.63841E-10	4.47886E-10	2.02673E-10
472.0	6.85437E-10	3.64245E-10	4.48276E-10	2.03046E-10
473.0	6.86012E-10	3.64647E-10	4.48665E-10	2.03419E-10
474.0	6.86586E-10	3.65048E-10	4.49053E-10	2.03791E-10
475.0	6.87158E-10	3.65447E-10	4.49440E-10	2.04161E-10
476.0	6.87729E-10	3.65846E-10	4.49826E-10	2.04531E-10

477.0	6.88299E-10	3.66242E-10	4.50211E-10	2.04900E-10
478.0	6.88867E-10	3.66638E-10	4.50595E-10	2.05268E-10
479.0	6.89434E-10	3.67032E-10	4.50979E-10	2.05635E-10
480.0	6.90000E-10	3.67424E-10	4.51361E-10	2.06001E-10
481.0	6.90565E-10	3.67816E-10	4.51742E-10	2.06366E-10
482.0	6.91128E-10	3.68206E-10	4.52123E-10	2.06730E-10
483.0	6.91689E-10	3.68594E-10	4.52502E-10	2.07093E-10
484.0	6.92250E-10	3.68981E-10	4.52881E-10	2.07455E-10
485.0	6.92809E-10	3.69367E-10	4.53259E-10	2.07817E-10
486.0	6.93367E-10	3.69752E-10	4.53636E-10	2.08177E-10
487.0	6.93923E-10	3.70135E-10	4.54011E-10	2.08536E-10
488.0	6.94479E-10	3.70517E-10	4.54387E-10	2.08895E-10
489.0	6.95033E-10	3.70898E-10	4.54761E-10	2.09253E-10
490.0	6.95585E-10	3.71277E-10	4.55134E-10	2.09609E-10
491.0	6.96137E-10	3.71656E-10	4.55506E-10	2.09965E-10
492.0	6.96687E-10	3.72032E-10	4.55878E-10	2.10320E-10
493.0	6.97236E-10	3.72408E-10	4.56249E-10	2.10674E-10
494.0	6.97783E-10	3.72782E-10	4.56618E-10	2.11027E-10
495.0	6.98330E-10	3.73155E-10	4.56987E-10	2.11379E-10
496.0	6.98875E-10	3.73527E-10	4.57356E-10	2.11731E-10
497.0	6.99419E-10	3.73897E-10	4.57723E-10	2.12081E-10
498.0	6.99961E-10	3.74267E-10	4.58089E-10	2.12430E-10
499.0	7.00503E-10	3.74635E-10	4.58455E-10	2.12779E-10
500.0	7.01043E-10	3.75001E-10	4.58819E-10	2.13127E-10

T (K)	2--->3	2--->4	3--->4	3--->5	4--->5
5.0	3.58339E-35	9.37646E-69	9.30889E-44	9.15490E-86	4.47422E-52
6.0	5.81251E-31	5.85388E-59	3.86504E-38	3.76180E-73	4.60052E-45
7.0	5.91103E-28	5.81256E-52	3.99216E-34	3.85515E-64	4.69419E-40
8.0	1.06349E-25	1.02759E-46	4.09372E-31	2.20950E-57	2.67819E-36
9.0	6.02947E-24	1.23865E-42	8.99846E-29	3.98933E-52	2.23418E-33
10.0	1.52342E-22	2.27888E-39	6.73007E-27	6.40302E-48	4.85236E-31
11.0	2.13877E-21	1.06873E-36	2.29816E-25	1.76791E-44	3.96098E-29
12.0	1.93276E-20	1.79825E-34	4.35898E-24	1.30396E-41	1.55226E-27
13.0	1.24471E-19	1.37547E-32	5.25977E-23	3.48429E-39	3.45957E-26
14.0	6.14329E-19	5.66328E-31	4.44853E-22	4.19295E-37	4.94906E-25
15.0	2.45088E-18	1.42076E-29	2.83138E-21	2.66555E-35	4.96592E-24
16.0	8.22592E-18	2.38326E-28	1.43041E-20	1.00900E-33	3.73550E-23
17.0	2.39470E-17	2.86997E-27	5.97447E-20	2.49215E-32	2.21648E-22
18.0	6.19209E-17	2.62204E-26	2.12954E-19	4.31268E-31	1.07926E-21
19.0	1.44904E-16	1.89847E-25	6.64181E-19	5.53105E-30	4.44937E-21
20.0	3.11519E-16	1.12804E-24	1.84926E-18	5.49884E-29	1.59231E-20
21.0	6.22755E-16	5.65803E-24	4.67151E-18	4.39475E-28	5.04767E-20
22.0	1.16918E-15	2.45169E-23	1.08499E-17	2.90883E-27	1.44103E-19
23.0	2.07841E-15	9.35429E-23	2.34238E-17	1.63417E-26	3.75578E-19
24.0	3.52232E-15	3.19307E-22	4.74370E-17	7.95358E-26	9.03894E-19
25.0	5.72342E-15	9.88233E-22	9.08108E-17	3.41180E-25	2.02803E-18
26.0	8.96016E-15	2.80459E-21	1.65399E-16	1.30882E-24	4.27641E-18
27.0	1.35706E-14	7.36917E-21	2.88206E-16	4.54606E-24	8.53344E-18
28.0	1.99544E-14	1.80754E-20	4.82732E-16	1.44498E-23	1.62096E-17

29.0	2.85732E-14	4.16837E-20	7.80392E-16	4.24167E-23	2.94599E-17
30.0	3.99489E-14	9.09345E-20	1.22197E-15	1.15907E-22	5.14538E-17
31.0	5.46606E-14	1.88671E-19	1.85904E-15	2.96880E-22	8.66960E-17
32.0	7.33403E-14	3.74052E-19	2.75528E-15	7.17100E-22	1.41395E-16
33.0	9.66669E-14	7.11546E-19	3.98770E-15	1.64216E-21	2.23876E-16
34.0	1.25360E-13	1.30346E-18	5.64763E-15	3.58203E-21	3.45026E-16
35.0	1.60171E-13	2.30684E-18	7.84152E-15	7.47352E-21	5.18761E-16
36.0	2.01879E-13	3.95553E-18	1.06916E-14	1.49695E-20	7.62515E-16
37.0	2.51279E-13	6.58826E-18	1.43359E-14	2.88810E-20	1.09772E-15
38.0	3.09176E-13	1.06834E-17	1.89289E-14	5.38294E-20	1.55026E-15
39.0	3.76379E-13	1.69008E-17	2.46406E-14	9.71807E-20	2.15095E-15
40.0	4.53689E-13	2.61321E-17	3.16567E-14	1.70345E-19	2.93592E-15
41.0	5.41899E-13	3.95576E-17	4.01777E-14	2.90540E-19	3.94698E-15
42.0	6.41783E-13	5.87124E-17	5.04181E-14	4.83117E-19	5.23196E-15
43.0	7.54090E-13	8.55598E-17	6.26052E-14	7.84583E-19	6.84490E-15
44.0	8.79543E-13	1.22572E-16	7.69784E-14	1.24642E-18	8.84630E-15
45.0	1.01883E-12	1.72816E-16	9.37875E-14	1.93982E-18	1.13032E-14
46.0	1.17261E-12	2.40049E-16	1.13292E-13	2.96153E-18	1.42893E-14
47.0	1.34150E-12	3.28814E-16	1.35757E-13	4.44076E-18	1.78848E-14
48.0	1.52607E-12	4.44542E-16	1.61458E-13	6.54747E-18	2.21765E-14
49.0	1.72685E-12	5.93656E-16	1.90671E-13	9.50194E-18	2.72574E-14
50.0	1.94433E-12	7.83674E-16	2.23679E-13	1.35858E-17	3.32269E-14
51.0	2.17895E-12	1.02331E-15	2.60764E-13	1.91547E-17	4.01901E-14
52.0	2.43111E-12	1.32259E-15	3.02210E-13	2.66520E-17	4.82576E-14
53.0	2.70117E-12	1.69292E-15	3.48301E-13	3.66246E-17	5.75456E-14
54.0	2.98942E-12	2.14723E-15	3.99316E-13	4.97402E-17	6.81749E-14
55.0	3.29613E-12	2.70000E-15	4.55535E-13	6.68052E-17	8.02707E-14
56.0	3.62153E-12	3.36738E-15	5.17230E-13	8.87851E-17	9.39623E-14
57.0	3.96579E-12	4.16728E-15	5.84668E-13	1.16826E-16	1.09383E-13
58.0	4.32904E-12	5.11938E-15	6.58112E-13	1.52275E-16	1.26667E-13
59.0	4.71139E-12	6.24524E-15	7.37816E-13	1.96706E-16	1.45955E-13
60.0	5.11290E-12	7.56830E-15	8.24025E-13	2.51943E-16	1.67387E-13
61.0	5.53359E-12	9.11395E-15	9.16977E-13	3.20085E-16	1.91104E-13
62.0	5.97345E-12	1.09095E-14	1.01690E-12	4.03531E-16	2.17250E-13
63.0	6.43244E-12	1.29844E-14	1.12401E-12	5.05004E-16	2.45969E-13
64.0	6.91050E-12	1.53698E-14	1.23852E-12	6.27580E-16	2.77406E-13
65.0	7.40750E-12	1.80990E-14	1.36063E-12	7.74714E-16	3.11704E-13
66.0	7.92334E-12	2.12072E-14	1.49051E-12	9.50261E-16	3.49007E-13
67.0	8.45786E-12	2.47316E-14	1.62835E-12	1.15850E-15	3.89458E-13
68.0	9.01087E-12	2.87111E-14	1.77432E-12	1.40418E-15	4.33196E-13
69.0	9.58219E-12	3.31867E-14	1.92855E-12	1.69249E-15	4.80361E-13
70.0	1.01716E-11	3.82009E-14	2.09120E-12	2.02915E-15	5.31090E-13
71.0	1.07789E-11	4.37981E-14	2.26239E-12	2.42038E-15	5.85519E-13
72.0	1.14037E-11	5.00243E-14	2.44225E-12	2.87294E-15	6.43777E-13
73.0	1.20459E-11	5.69270E-14	2.63088E-12	3.39415E-15	7.05995E-13
74.0	1.27051E-11	6.45552E-14	2.82837E-12	3.99189E-15	7.72297E-13
75.0	1.33811E-11	7.29594E-14	3.03482E-12	4.67464E-15	8.42806E-13
76.0	1.40735E-11	8.21913E-14	3.25029E-12	5.45147E-15	9.17639E-13
77.0	1.47821E-11	9.23039E-14	3.47485E-12	6.33206E-15	9.96912E-13
78.0	1.55064E-11	1.03351E-13	3.70855E-12	7.32672E-15	1.08074E-12

79.0	1.62463E-11	1.15388E-13	3.95144E-12	8.44636E-15	1.16921E-12
80.0	1.70012E-11	1.28470E-13	4.20355E-12	9.70256E-15	1.26245E-12
81.0	1.77710E-11	1.42655E-13	4.46491E-12	1.11075E-14	1.36054E-12
82.0	1.85552E-11	1.57999E-13	4.73553E-12	1.26740E-14	1.46358E-12
83.0	1.93536E-11	1.74561E-13	5.01542E-12	1.44155E-14	1.57166E-12
84.0	2.01656E-11	1.92399E-13	5.30457E-12	1.63462E-14	1.68486E-12
85.0	2.09911E-11	2.11572E-13	5.60298E-12	1.84806E-14	1.80326E-12
86.0	2.18296E-11	2.32138E-13	5.91062E-12	2.08342E-14	1.92693E-12
87.0	2.26807E-11	2.54157E-13	6.22748E-12	2.34229E-14	2.05595E-12
88.0	2.35442E-11	2.77688E-13	6.55351E-12	2.62633E-14	2.19038E-12
89.0	2.44196E-11	3.02790E-13	6.88869E-12	2.93724E-14	2.33028E-12
90.0	2.53067E-11	3.29523E-13	7.23295E-12	3.27680E-14	2.47571E-12
91.0	2.62049E-11	3.57944E-13	7.58626E-12	3.64682E-14	2.62672E-12
92.0	2.71141E-11	3.88112E-13	7.94855E-12	4.04920E-14	2.78336E-12
93.0	2.80338E-11	4.20085E-13	8.31976E-12	4.48587E-14	2.94567E-12
94.0	2.89637E-11	4.53921E-13	8.69981E-12	4.95879E-14	3.11368E-12
95.0	2.99035E-11	4.89676E-13	9.08864E-12	5.47002E-14	3.28744E-12
96.0	3.08528E-11	5.27407E-13	9.48617E-12	6.02161E-14	3.46697E-12
97.0	3.18112E-11	5.67169E-13	9.89231E-12	6.61570E-14	3.65230E-12
98.0	3.27786E-11	6.09016E-13	1.03070E-11	7.25444E-14	3.84344E-12
99.0	3.37544E-11	6.53002E-13	1.07301E-11	7.94004E-14	4.04043E-12
100.0	3.47384E-11	6.99180E-13	1.11615E-11	8.67473E-14	4.24327E-12
101.0	3.57303E-11	7.47602E-13	1.16012E-11	9.46079E-14	4.45197E-12
102.0	3.67297E-11	7.98318E-13	1.20490E-11	1.03005E-13	4.66654E-12
103.0	3.77364E-11	8.51377E-13	1.25048E-11	1.11963E-13	4.88698E-12
104.0	3.87501E-11	9.06830E-13	1.29686E-11	1.21504E-13	5.11330E-12
105.0	3.97704E-11	9.64721E-13	1.34402E-11	1.31652E-13	5.34548E-12
106.0	4.07970E-11	1.02510E-12	1.39195E-11	1.42432E-13	5.58352E-12
107.0	4.18297E-11	1.08801E-12	1.44064E-11	1.53868E-13	5.82741E-12
108.0	4.28682E-11	1.15349E-12	1.49008E-11	1.65984E-13	6.07714E-12
109.0	4.39122E-11	1.22158E-12	1.54025E-11	1.78804E-13	6.33269E-12
110.0	4.49615E-11	1.29234E-12	1.59115E-11	1.92354E-13	6.59405E-12
111.0	4.60157E-11	1.36579E-12	1.64276E-11	2.06658E-13	6.86118E-12
112.0	4.70746E-11	1.44197E-12	1.69507E-11	2.21740E-13	7.13407E-12
113.0	4.81380E-11	1.52092E-12	1.74807E-11	2.37626E-13	7.41269E-12
114.0	4.92056E-11	1.60268E-12	1.80174E-11	2.54341E-13	7.69701E-12
115.0	5.02771E-11	1.68727E-12	1.85608E-11	2.71908E-13	7.98699E-12
116.0	5.13523E-11	1.77474E-12	1.91106E-11	2.90354E-13	8.28262E-12
117.0	5.24311E-11	1.86511E-12	1.96669E-11	3.09702E-13	8.58384E-12
118.0	5.35131E-11	1.95841E-12	2.02295E-11	3.29977E-13	8.89062E-12
119.0	5.45981E-11	2.05467E-12	2.07981E-11	3.51203E-13	9.20293E-12
120.0	5.56860E-11	2.15391E-12	2.13728E-11	3.73406E-13	9.52071E-12
121.0	5.67765E-11	2.25617E-12	2.19534E-11	3.96608E-13	9.84393E-12
122.0	5.78693E-11	2.36145E-12	2.25397E-11	4.20835E-13	1.01725E-11
123.0	5.89644E-11	2.46980E-12	2.31317E-11	4.46109E-13	1.05065E-11
124.0	6.00615E-11	2.58121E-12	2.37292E-11	4.72455E-13	1.08458E-11
125.0	6.11604E-11	2.69573E-12	2.43322E-11	4.99896E-13	1.11903E-11
126.0	6.22609E-11	2.81335E-12	2.49403E-11	5.28455E-13	1.15400E-11
127.0	6.33628E-11	2.93411E-12	2.55537E-11	5.58154E-13	1.18948E-11
128.0	6.44661E-11	3.05801E-12	2.61721E-11	5.89016E-13	1.22547E-11

129.0	6.55704E-11	3.18506E-12	2.67953E-11	6.21065E-13	1.26197E-11
130.0	6.66757E-11	3.31529E-12	2.74234E-11	6.54320E-13	1.29897E-11
131.0	6.77817E-11	3.44870E-12	2.80562E-11	6.88805E-13	1.33646E-11
132.0	6.88883E-11	3.58530E-12	2.86935E-11	7.24540E-13	1.37443E-11
133.0	6.99954E-11	3.72509E-12	2.93352E-11	7.61546E-13	1.41289E-11
134.0	7.11028E-11	3.86810E-12	2.99813E-11	7.99844E-13	1.45182E-11
135.0	7.22104E-11	4.01431E-12	3.06316E-11	8.39454E-13	1.49122E-11
136.0	7.33180E-11	4.16374E-12	3.12859E-11	8.80396E-13	1.53109E-11
137.0	7.44254E-11	4.31639E-12	3.19443E-11	9.22689E-13	1.57141E-11
138.0	7.55327E-11	4.47226E-12	3.26066E-11	9.66352E-13	1.61218E-11
139.0	7.66395E-11	4.63136E-12	3.32726E-11	1.01140E-12	1.65340E-11
140.0	7.77458E-11	4.79368E-12	3.39422E-11	1.05786E-12	1.69506E-11
141.0	7.88516E-11	4.95922E-12	3.46154E-11	1.10574E-12	1.73715E-11
142.0	7.99565E-11	5.12798E-12	3.52921E-11	1.15507E-12	1.77967E-11
143.0	8.10607E-11	5.29995E-12	3.59720E-11	1.20585E-12	1.82261E-11
144.0	8.21638E-11	5.47514E-12	3.66553E-11	1.25811E-12	1.86596E-11
145.0	8.32659E-11	5.65353E-12	3.73416E-11	1.31186E-12	1.90972E-11
146.0	8.43668E-11	5.83513E-12	3.80310E-11	1.36712E-12	1.95389E-11
147.0	8.54665E-11	6.01992E-12	3.87233E-11	1.42389E-12	1.99844E-11
148.0	8.65647E-11	6.20789E-12	3.94184E-11	1.48220E-12	2.04339E-11
149.0	8.76615E-11	6.39904E-12	4.01163E-11	1.54207E-12	2.08872E-11
150.0	8.87568E-11	6.59335E-12	4.08168E-11	1.60349E-12	2.13443E-11
151.0	8.98504E-11	6.79082E-12	4.15199E-11	1.66650E-12	2.18050E-11
152.0	9.09423E-11	6.99144E-12	4.22254E-11	1.73109E-12	2.22694E-11
153.0	9.20323E-11	7.19518E-12	4.29332E-11	1.79728E-12	2.27374E-11
154.0	9.31205E-11	7.40205E-12	4.36434E-11	1.86509E-12	2.32089E-11
155.0	9.42068E-11	7.61202E-12	4.43557E-11	1.93452E-12	2.36838E-11
156.0	9.52909E-11	7.82508E-12	4.50701E-11	2.00559E-12	2.41621E-11
157.0	9.63730E-11	8.04121E-12	4.57866E-11	2.07830E-12	2.46438E-11
158.0	9.74529E-11	8.26040E-12	4.65050E-11	2.15267E-12	2.51287E-11
159.0	9.85306E-11	8.48263E-12	4.72252E-11	2.22871E-12	2.56168E-11
160.0	9.96060E-11	8.70789E-12	4.79471E-11	2.30642E-12	2.61081E-11
161.0	1.00679E-10	8.93615E-12	4.86708E-11	2.38581E-12	2.66024E-11
162.0	1.01750E-10	9.16741E-12	4.93960E-11	2.46689E-12	2.70998E-11
163.0	1.02818E-10	9.40163E-12	5.01228E-11	2.54966E-12	2.76001E-11
164.0	1.03883E-10	9.63880E-12	5.08511E-11	2.63415E-12	2.81033E-11
165.0	1.04946E-10	9.87891E-12	5.15807E-11	2.72034E-12	2.86094E-11
166.0	1.06006E-10	1.01219E-11	5.23116E-11	2.80826E-12	2.91182E-11
167.0	1.07064E-10	1.03678E-11	5.30437E-11	2.89789E-12	2.96298E-11
168.0	1.08119E-10	1.06166E-11	5.37770E-11	2.98926E-12	3.01440E-11
169.0	1.09171E-10	1.08682E-11	5.45114E-11	3.08236E-12	3.06608E-11
170.0	1.10221E-10	1.11226E-11	5.52469E-11	3.17719E-12	3.11802E-11
171.0	1.11267E-10	1.13799E-11	5.59832E-11	3.27377E-12	3.17021E-11
172.0	1.12311E-10	1.16399E-11	5.67205E-11	3.37210E-12	3.22264E-11
173.0	1.13351E-10	1.19027E-11	5.74586E-11	3.47217E-12	3.27531E-11
174.0	1.14389E-10	1.21681E-11	5.81974E-11	3.57400E-12	3.32821E-11
175.0	1.15424E-10	1.24364E-11	5.89370E-11	3.67758E-12	3.38134E-11
176.0	1.16455E-10	1.27072E-11	5.96771E-11	3.78292E-12	3.43470E-11
177.0	1.17484E-10	1.29808E-11	6.04179E-11	3.89001E-12	3.48826E-11
178.0	1.18509E-10	1.32570E-11	6.11592E-11	3.99887E-12	3.54204E-11

179.0	1.19532E-10	1.35357E-11	6.19009E-11	4.10948E-12	3.59603E-11
180.0	1.20551E-10	1.38171E-11	6.26431E-11	4.22186E-12	3.65021E-11
181.0	1.21567E-10	1.41010E-11	6.33856E-11	4.33599E-12	3.70459E-11
182.0	1.22580E-10	1.43875E-11	6.41284E-11	4.45189E-12	3.75917E-11
183.0	1.23590E-10	1.46764E-11	6.48715E-11	4.56954E-12	3.81392E-11
184.0	1.24596E-10	1.49679E-11	6.56147E-11	4.68895E-12	3.86886E-11
185.0	1.25599E-10	1.52618E-11	6.63581E-11	4.81012E-12	3.92397E-11
186.0	1.26599E-10	1.55581E-11	6.71016E-11	4.93305E-12	3.97925E-11
187.0	1.27596E-10	1.58568E-11	6.78451E-11	5.05773E-12	4.03470E-11
188.0	1.28589E-10	1.61579E-11	6.85887E-11	5.18416E-12	4.09031E-11
189.0	1.29579E-10	1.64613E-11	6.93322E-11	5.31234E-12	4.14607E-11
190.0	1.30566E-10	1.67671E-11	7.00756E-11	5.44227E-12	4.20199E-11
191.0	1.31549E-10	1.70751E-11	7.08188E-11	5.57393E-12	4.25805E-11
192.0	1.32529E-10	1.73854E-11	7.15619E-11	5.70734E-12	4.31426E-11
193.0	1.33505E-10	1.76980E-11	7.23048E-11	5.84248E-12	4.37060E-11
194.0	1.34478E-10	1.80128E-11	7.30474E-11	5.97936E-12	4.42708E-11
195.0	1.35448E-10	1.83297E-11	7.37897E-11	6.11796E-12	4.48369E-11
196.0	1.36415E-10	1.86488E-11	7.45316E-11	6.25828E-12	4.54042E-11
197.0	1.37378E-10	1.89701E-11	7.52732E-11	6.40032E-12	4.59727E-11
198.0	1.38337E-10	1.92934E-11	7.60143E-11	6.54407E-12	4.65424E-11
199.0	1.39293E-10	1.96188E-11	7.67550E-11	6.68952E-12	4.71132E-11
200.0	1.40246E-10	1.99463E-11	7.74952E-11	6.83668E-12	4.76850E-11
201.0	1.41195E-10	2.02758E-11	7.82349E-11	6.98553E-12	4.82580E-11
202.0	1.42141E-10	2.06073E-11	7.89740E-11	7.13607E-12	4.88319E-11
203.0	1.43083E-10	2.09407E-11	7.97125E-11	7.28830E-12	4.94068E-11
204.0	1.44022E-10	2.12761E-11	8.04504E-11	7.44220E-12	4.99826E-11
205.0	1.44958E-10	2.16134E-11	8.11876E-11	7.59776E-12	5.05593E-11
206.0	1.45890E-10	2.19526E-11	8.19241E-11	7.75499E-12	5.11368E-11
207.0	1.46819E-10	2.22937E-11	8.26599E-11	7.91388E-12	5.17151E-11
208.0	1.47744E-10	2.26365E-11	8.33950E-11	8.07441E-12	5.22943E-11
209.0	1.48666E-10	2.29812E-11	8.41292E-11	8.23658E-12	5.28741E-11
210.0	1.49584E-10	2.33277E-11	8.48627E-11	8.40038E-12	5.34547E-11
211.0	1.50499E-10	2.36759E-11	8.55953E-11	8.56580E-12	5.40359E-11
212.0	1.51410E-10	2.40259E-11	8.63271E-11	8.73284E-12	5.46177E-11
213.0	1.52318E-10	2.43775E-11	8.70579E-11	8.90149E-12	5.52002E-11
214.0	1.53223E-10	2.47309E-11	8.77879E-11	9.07173E-12	5.57832E-11
215.0	1.54124E-10	2.50858E-11	8.85169E-11	9.24357E-12	5.63668E-11
216.0	1.55022E-10	2.54424E-11	8.92450E-11	9.41698E-12	5.69508E-11
217.0	1.55916E-10	2.58006E-11	8.99720E-11	9.59197E-12	5.75353E-11
218.0	1.56807E-10	2.61604E-11	9.06981E-11	9.76852E-12	5.81203E-11
219.0	1.57695E-10	2.65217E-11	9.14231E-11	9.94662E-12	5.87056E-11
220.0	1.58579E-10	2.68846E-11	9.21471E-11	1.01263E-11	5.92914E-11
221.0	1.59459E-10	2.72489E-11	9.28700E-11	1.03074E-11	5.98774E-11
222.0	1.60337E-10	2.76147E-11	9.35918E-11	1.04901E-11	6.04638E-11
223.0	1.61211E-10	2.79819E-11	9.43124E-11	1.06744E-11	6.10505E-11
224.0	1.62081E-10	2.83506E-11	9.50320E-11	1.08601E-11	6.16374E-11
225.0	1.62949E-10	2.87207E-11	9.57504E-11	1.10473E-11	6.22246E-11
226.0	1.63812E-10	2.90921E-11	9.64676E-11	1.12360E-11	6.28120E-11
227.0	1.64673E-10	2.94649E-11	9.71836E-11	1.14262E-11	6.33995E-11
228.0	1.65530E-10	2.98390E-11	9.78985E-11	1.16178E-11	6.39872E-11

229.0	1.66384E-10	3.02144E-11	9.86121E-11	1.18109E-11	6.45750E-11
230.0	1.67235E-10	3.05910E-11	9.93244E-11	1.20055E-11	6.51629E-11
231.0	1.68082E-10	3.09690E-11	1.00036E-10	1.22014E-11	6.57509E-11
232.0	1.68926E-10	3.13481E-11	1.00745E-10	1.23988E-11	6.63390E-11
233.0	1.69767E-10	3.17285E-11	1.01454E-10	1.25976E-11	6.69270E-11
234.0	1.70604E-10	3.21100E-11	1.02161E-10	1.27978E-11	6.75150E-11
235.0	1.71438E-10	3.24927E-11	1.02867E-10	1.29994E-11	6.81031E-11
236.0	1.72269E-10	3.28765E-11	1.03572E-10	1.32024E-11	6.86910E-11
237.0	1.73097E-10	3.32615E-11	1.04275E-10	1.34068E-11	6.92789E-11
238.0	1.73921E-10	3.36475E-11	1.04977E-10	1.36124E-11	6.98667E-11
239.0	1.74742E-10	3.40346E-11	1.05677E-10	1.38195E-11	7.04544E-11
240.0	1.75560E-10	3.44227E-11	1.06377E-10	1.40279E-11	7.10420E-11
241.0	1.76375E-10	3.48119E-11	1.07074E-10	1.42376E-11	7.16294E-11
242.0	1.77187E-10	3.52021E-11	1.07771E-10	1.44486E-11	7.22166E-11
243.0	1.77995E-10	3.55932E-11	1.08466E-10	1.46609E-11	7.28036E-11
244.0	1.78800E-10	3.59853E-11	1.09159E-10	1.48745E-11	7.33904E-11
245.0	1.79602E-10	3.63783E-11	1.09852E-10	1.50894E-11	7.39769E-11
246.0	1.80401E-10	3.67723E-11	1.10542E-10	1.53055E-11	7.45632E-11
247.0	1.81197E-10	3.71671E-11	1.11232E-10	1.55229E-11	7.51492E-11
248.0	1.81990E-10	3.75629E-11	1.11919E-10	1.57415E-11	7.57349E-11
249.0	1.82779E-10	3.79594E-11	1.12606E-10	1.59614E-11	7.63203E-11
250.0	1.83566E-10	3.83569E-11	1.13291E-10	1.61825E-11	7.69053E-11
251.0	1.84349E-10	3.87551E-11	1.13974E-10	1.64048E-11	7.74900E-11
252.0	1.85130E-10	3.91541E-11	1.14656E-10	1.66283E-11	7.80744E-11
253.0	1.85907E-10	3.95539E-11	1.15336E-10	1.68530E-11	7.86583E-11
254.0	1.86681E-10	3.99545E-11	1.16015E-10	1.70788E-11	7.92418E-11
255.0	1.87453E-10	4.03557E-11	1.16693E-10	1.73058E-11	7.98250E-11
256.0	1.88221E-10	4.07577E-11	1.17368E-10	1.75340E-11	8.04076E-11
257.0	1.88986E-10	4.11604E-11	1.18043E-10	1.77633E-11	8.09899E-11
258.0	1.89749E-10	4.15638E-11	1.18716E-10	1.79938E-11	8.15716E-11
259.0	1.90508E-10	4.19679E-11	1.19387E-10	1.82253E-11	8.21529E-11
260.0	1.91264E-10	4.23725E-11	1.20057E-10	1.84580E-11	8.27337E-11
261.0	1.92018E-10	4.27779E-11	1.20725E-10	1.86917E-11	8.33140E-11
262.0	1.92768E-10	4.31838E-11	1.21392E-10	1.89265E-11	8.38937E-11
263.0	1.93516E-10	4.35903E-11	1.22057E-10	1.91624E-11	8.44729E-11
264.0	1.94261E-10	4.39973E-11	1.22720E-10	1.93994E-11	8.50516E-11
265.0	1.95003E-10	4.44050E-11	1.23382E-10	1.96374E-11	8.56296E-11
266.0	1.95742E-10	4.48131E-11	1.24043E-10	1.98765E-11	8.62072E-11
267.0	1.96478E-10	4.52218E-11	1.24702E-10	2.01165E-11	8.67841E-11
268.0	1.97211E-10	4.56310E-11	1.25359E-10	2.03576E-11	8.73604E-11
269.0	1.97941E-10	4.60406E-11	1.26015E-10	2.05997E-11	8.79361E-11
270.0	1.98669E-10	4.64508E-11	1.26669E-10	2.08427E-11	8.85111E-11
271.0	1.99394E-10	4.68614E-11	1.27322E-10	2.10868E-11	8.90855E-11
272.0	2.00116E-10	4.72724E-11	1.27973E-10	2.13318E-11	8.96593E-11
273.0	2.00835E-10	4.76838E-11	1.28622E-10	2.15778E-11	9.02324E-11
274.0	2.01552E-10	4.80957E-11	1.29270E-10	2.18247E-11	9.08048E-11
275.0	2.02266E-10	4.85079E-11	1.29916E-10	2.20725E-11	9.13766E-11
276.0	2.02977E-10	4.89205E-11	1.30561E-10	2.23213E-11	9.19476E-11
277.0	2.03685E-10	4.93335E-11	1.31204E-10	2.25710E-11	9.25179E-11
278.0	2.04391E-10	4.97468E-11	1.31846E-10	2.28215E-11	9.30875E-11

279.0	2.05094E-10	5.01605E-11	1.32486E-10	2.30730E-11	9.36564E-11
280.0	2.05794E-10	5.05744E-11	1.33124E-10	2.33253E-11	9.42246E-11
281.0	2.06491E-10	5.09887E-11	1.33761E-10	2.35786E-11	9.47920E-11
282.0	2.07186E-10	5.14032E-11	1.34396E-10	2.38326E-11	9.53586E-11
283.0	2.07879E-10	5.18181E-11	1.35030E-10	2.40875E-11	9.59245E-11
284.0	2.08569E-10	5.22331E-11	1.35662E-10	2.43433E-11	9.64896E-11
285.0	2.09256E-10	5.26484E-11	1.36292E-10	2.45998E-11	9.70540E-11
286.0	2.09940E-10	5.30640E-11	1.36921E-10	2.48572E-11	9.76175E-11
287.0	2.10622E-10	5.34797E-11	1.37548E-10	2.51154E-11	9.81803E-11
288.0	2.11302E-10	5.38957E-11	1.38174E-10	2.53743E-11	9.87422E-11
289.0	2.11978E-10	5.43119E-11	1.38798E-10	2.56341E-11	9.93033E-11
290.0	2.12653E-10	5.47282E-11	1.39420E-10	2.58946E-11	9.98636E-11
291.0	2.13325E-10	5.51447E-11	1.40041E-10	2.61559E-11	1.00423E-10
292.0	2.13994E-10	5.55613E-11	1.40660E-10	2.64179E-11	1.00982E-10
293.0	2.14661E-10	5.59781E-11	1.41278E-10	2.66807E-11	1.01540E-10
294.0	2.15325E-10	5.63950E-11	1.41894E-10	2.69441E-11	1.02096E-10
295.0	2.15987E-10	5.68120E-11	1.42509E-10	2.72084E-11	1.02653E-10
296.0	2.16646E-10	5.72291E-11	1.43121E-10	2.74733E-11	1.03208E-10
297.0	2.17303E-10	5.76464E-11	1.43733E-10	2.77389E-11	1.03762E-10
298.0	2.17958E-10	5.80636E-11	1.44343E-10	2.80052E-11	1.04316E-10
299.0	2.18610E-10	5.84810E-11	1.44951E-10	2.82721E-11	1.04868E-10
300.0	2.19260E-10	5.88984E-11	1.45557E-10	2.85398E-11	1.05420E-10
301.0	2.19907E-10	5.93159E-11	1.46162E-10	2.88080E-11	1.05971E-10
302.0	2.20552E-10	5.97333E-11	1.46766E-10	2.90770E-11	1.06521E-10
303.0	2.21195E-10	6.01508E-11	1.47368E-10	2.93465E-11	1.07070E-10
304.0	2.21835E-10	6.05684E-11	1.47968E-10	2.96167E-11	1.07618E-10
305.0	2.22473E-10	6.09859E-11	1.48567E-10	2.98876E-11	1.08165E-10
306.0	2.23109E-10	6.14034E-11	1.49164E-10	3.01590E-11	1.08711E-10
307.0	2.23742E-10	6.18209E-11	1.49760E-10	3.04310E-11	1.09257E-10
308.0	2.24373E-10	6.22383E-11	1.50354E-10	3.07036E-11	1.09801E-10
309.0	2.25002E-10	6.26557E-11	1.50946E-10	3.09768E-11	1.10344E-10
310.0	2.25629E-10	6.30731E-11	1.51537E-10	3.12505E-11	1.10887E-10
311.0	2.26253E-10	6.34904E-11	1.52127E-10	3.15248E-11	1.11429E-10
312.0	2.26875E-10	6.39076E-11	1.52714E-10	3.17997E-11	1.11969E-10
313.0	2.27495E-10	6.43248E-11	1.53301E-10	3.20751E-11	1.12509E-10
314.0	2.28112E-10	6.47418E-11	1.53886E-10	3.23510E-11	1.13048E-10
315.0	2.28728E-10	6.51588E-11	1.54469E-10	3.26274E-11	1.13586E-10
316.0	2.29341E-10	6.55756E-11	1.55050E-10	3.29044E-11	1.14122E-10
317.0	2.29952E-10	6.59923E-11	1.55631E-10	3.31819E-11	1.14658E-10
318.0	2.30561E-10	6.64089E-11	1.56209E-10	3.34598E-11	1.15193E-10
319.0	2.31168E-10	6.68254E-11	1.56786E-10	3.37382E-11	1.15727E-10
320.0	2.31772E-10	6.72417E-11	1.57362E-10	3.40172E-11	1.16260E-10
321.0	2.32375E-10	6.76579E-11	1.57936E-10	3.42966E-11	1.16792E-10
322.0	2.32975E-10	6.80739E-11	1.58508E-10	3.45764E-11	1.17323E-10
323.0	2.33574E-10	6.84897E-11	1.59079E-10	3.48567E-11	1.17853E-10
324.0	2.34170E-10	6.89054E-11	1.59649E-10	3.51374E-11	1.18382E-10
325.0	2.34764E-10	6.93208E-11	1.60217E-10	3.54186E-11	1.18910E-10
326.0	2.35356E-10	6.97361E-11	1.60783E-10	3.57002E-11	1.19437E-10
327.0	2.35946E-10	7.01512E-11	1.61348E-10	3.59822E-11	1.19963E-10
328.0	2.36534E-10	7.05660E-11	1.61912E-10	3.62646E-11	1.20488E-10

329.0	2.37120E-10	7.09806E-11	1.62474E-10	3.65474E-11	1.21012E-10
330.0	2.37704E-10	7.13951E-11	1.63034E-10	3.68306E-11	1.21536E-10
331.0	2.38286E-10	7.18092E-11	1.63594E-10	3.71142E-11	1.22058E-10
332.0	2.38866E-10	7.22232E-11	1.64151E-10	3.73982E-11	1.22579E-10
333.0	2.39444E-10	7.26369E-11	1.64707E-10	3.76825E-11	1.23099E-10
334.0	2.40019E-10	7.30503E-11	1.65262E-10	3.79672E-11	1.23618E-10
335.0	2.40593E-10	7.34635E-11	1.65815E-10	3.82522E-11	1.24136E-10
336.0	2.41166E-10	7.38764E-11	1.66367E-10	3.85376E-11	1.24653E-10
337.0	2.41736E-10	7.42890E-11	1.66917E-10	3.88233E-11	1.25169E-10
338.0	2.42304E-10	7.47014E-11	1.67465E-10	3.91093E-11	1.25684E-10
339.0	2.42870E-10	7.51134E-11	1.68013E-10	3.93957E-11	1.26198E-10
340.0	2.43435E-10	7.55252E-11	1.68559E-10	3.96824E-11	1.26711E-10
341.0	2.43997E-10	7.59366E-11	1.69103E-10	3.99694E-11	1.27223E-10
342.0	2.44558E-10	7.63478E-11	1.69646E-10	4.02566E-11	1.27734E-10
343.0	2.45116E-10	7.67586E-11	1.70188E-10	4.05442E-11	1.28244E-10
344.0	2.45673E-10	7.71691E-11	1.70728E-10	4.08320E-11	1.28753E-10
345.0	2.46228E-10	7.75793E-11	1.71266E-10	4.11201E-11	1.29261E-10
346.0	2.46782E-10	7.79892E-11	1.71804E-10	4.14085E-11	1.29767E-10
347.0	2.47333E-10	7.83987E-11	1.72339E-10	4.16972E-11	1.30273E-10
348.0	2.47883E-10	7.88079E-11	1.72874E-10	4.19861E-11	1.30778E-10
349.0	2.48430E-10	7.92167E-11	1.73407E-10	4.22752E-11	1.31282E-10
350.0	2.48976E-10	7.96252E-11	1.73938E-10	4.25646E-11	1.31785E-10
351.0	2.49521E-10	8.00333E-11	1.74468E-10	4.28542E-11	1.32287E-10
352.0	2.50063E-10	8.04411E-11	1.74997E-10	4.31440E-11	1.32787E-10
353.0	2.50604E-10	8.08484E-11	1.75525E-10	4.34341E-11	1.33287E-10
354.0	2.51143E-10	8.12554E-11	1.76051E-10	4.37244E-11	1.33786E-10
355.0	2.51680E-10	8.16620E-11	1.76575E-10	4.40148E-11	1.34283E-10
356.0	2.52215E-10	8.20683E-11	1.77099E-10	4.43055E-11	1.34780E-10
357.0	2.52749E-10	8.24741E-11	1.77620E-10	4.45964E-11	1.35276E-10
358.0	2.53281E-10	8.28796E-11	1.78141E-10	4.48874E-11	1.35770E-10
359.0	2.53811E-10	8.32846E-11	1.78660E-10	4.51786E-11	1.36264E-10
360.0	2.54340E-10	8.36892E-11	1.79178E-10	4.54700E-11	1.36756E-10
361.0	2.54867E-10	8.40935E-11	1.79694E-10	4.57616E-11	1.37248E-10
362.0	2.55392E-10	8.44973E-11	1.80209E-10	4.60533E-11	1.37738E-10
363.0	2.55916E-10	8.49007E-11	1.80723E-10	4.63452E-11	1.38228E-10
364.0	2.56438E-10	8.53036E-11	1.81235E-10	4.66372E-11	1.38716E-10
365.0	2.56958E-10	8.57062E-11	1.81746E-10	4.69293E-11	1.39204E-10
366.0	2.57477E-10	8.61083E-11	1.82256E-10	4.72216E-11	1.39690E-10
367.0	2.57994E-10	8.65099E-11	1.82764E-10	4.75140E-11	1.40176E-10
368.0	2.58510E-10	8.69112E-11	1.83271E-10	4.78066E-11	1.40660E-10
369.0	2.59023E-10	8.73120E-11	1.83777E-10	4.80992E-11	1.41143E-10
370.0	2.59536E-10	8.77123E-11	1.84281E-10	4.83920E-11	1.41626E-10
371.0	2.60046E-10	8.81122E-11	1.84784E-10	4.86849E-11	1.42107E-10
372.0	2.60556E-10	8.85116E-11	1.85286E-10	4.89778E-11	1.42587E-10
373.0	2.61063E-10	8.89106E-11	1.85787E-10	4.92709E-11	1.43066E-10
374.0	2.61569E-10	8.93091E-11	1.86286E-10	4.95640E-11	1.43545E-10
375.0	2.62074E-10	8.97071E-11	1.86784E-10	4.98573E-11	1.44022E-10
376.0	2.62576E-10	9.01047E-11	1.87280E-10	5.01506E-11	1.44498E-10
377.0	2.63078E-10	9.05018E-11	1.87775E-10	5.04440E-11	1.44973E-10
378.0	2.63578E-10	9.08984E-11	1.88269E-10	5.07374E-11	1.45447E-10

379.0	2.64076E-10	9.12945E-11	1.88762E-10	5.10309E-11	1.45921E-10
380.0	2.64573E-10	9.16902E-11	1.89254E-10	5.13245E-11	1.46393E-10
381.0	2.65068E-10	9.20853E-11	1.89744E-10	5.16181E-11	1.46864E-10
382.0	2.65562E-10	9.24800E-11	1.90233E-10	5.19117E-11	1.47334E-10
383.0	2.66054E-10	9.28742E-11	1.90720E-10	5.22054E-11	1.47803E-10
384.0	2.66545E-10	9.32678E-11	1.91207E-10	5.24991E-11	1.48271E-10
385.0	2.67035E-10	9.36610E-11	1.91692E-10	5.27929E-11	1.48738E-10
386.0	2.67523E-10	9.40537E-11	1.92176E-10	5.30866E-11	1.49205E-10
387.0	2.68009E-10	9.44459E-11	1.92658E-10	5.33804E-11	1.49670E-10
388.0	2.68495E-10	9.48375E-11	1.93140E-10	5.36742E-11	1.50134E-10
389.0	2.68978E-10	9.52287E-11	1.93620E-10	5.39681E-11	1.50597E-10
390.0	2.69461E-10	9.56193E-11	1.94099E-10	5.42619E-11	1.51059E-10
391.0	2.69941E-10	9.60094E-11	1.94577E-10	5.45557E-11	1.51520E-10
392.0	2.70421E-10	9.63990E-11	1.95053E-10	5.48495E-11	1.51980E-10
393.0	2.70899E-10	9.67881E-11	1.95529E-10	5.51433E-11	1.52439E-10
394.0	2.71376E-10	9.71766E-11	1.96003E-10	5.54371E-11	1.52897E-10
395.0	2.71851E-10	9.75647E-11	1.96476E-10	5.57308E-11	1.53354E-10
396.0	2.72325E-10	9.79522E-11	1.96947E-10	5.60246E-11	1.53810E-10
397.0	2.72797E-10	9.83391E-11	1.97418E-10	5.63183E-11	1.54266E-10
398.0	2.73269E-10	9.87256E-11	1.97887E-10	5.66120E-11	1.54720E-10
399.0	2.73738E-10	9.91115E-11	1.98355E-10	5.69056E-11	1.55173E-10
400.0	2.74207E-10	9.94968E-11	1.98822E-10	5.71992E-11	1.55625E-10
401.0	2.74674E-10	9.98816E-11	1.99288E-10	5.74928E-11	1.56076E-10
402.0	2.75140E-10	1.00266E-10	1.99752E-10	5.77863E-11	1.56526E-10
403.0	2.75605E-10	1.00650E-10	2.00216E-10	5.80797E-11	1.56976E-10
404.0	2.76068E-10	1.01033E-10	2.00678E-10	5.83731E-11	1.57424E-10
405.0	2.76530E-10	1.01415E-10	2.01139E-10	5.86664E-11	1.57871E-10
406.0	2.76990E-10	1.01798E-10	2.01599E-10	5.89597E-11	1.58317E-10
407.0	2.77450E-10	1.02179E-10	2.02058E-10	5.92529E-11	1.58763E-10
408.0	2.77908E-10	1.02560E-10	2.02515E-10	5.95460E-11	1.59207E-10
409.0	2.78365E-10	1.02940E-10	2.02972E-10	5.98390E-11	1.59650E-10
410.0	2.78820E-10	1.03320E-10	2.03427E-10	6.01320E-11	1.60093E-10
411.0	2.79274E-10	1.03700E-10	2.03881E-10	6.04248E-11	1.60534E-10
412.0	2.79727E-10	1.04078E-10	2.04334E-10	6.07176E-11	1.60974E-10
413.0	2.80179E-10	1.04457E-10	2.04786E-10	6.10102E-11	1.61414E-10
414.0	2.80630E-10	1.04834E-10	2.05237E-10	6.13028E-11	1.61852E-10
415.0	2.81079E-10	1.05211E-10	2.05687E-10	6.15953E-11	1.62290E-10
416.0	2.81527E-10	1.05588E-10	2.06135E-10	6.18877E-11	1.62727E-10
417.0	2.81974E-10	1.05964E-10	2.06583E-10	6.21799E-11	1.63162E-10
418.0	2.82420E-10	1.06339E-10	2.07029E-10	6.24721E-11	1.63597E-10
419.0	2.82864E-10	1.06714E-10	2.07474E-10	6.27641E-11	1.64031E-10
420.0	2.83307E-10	1.07088E-10	2.07918E-10	6.30560E-11	1.64463E-10
421.0	2.83749E-10	1.07462E-10	2.08361E-10	6.33478E-11	1.64895E-10
422.0	2.84190E-10	1.07835E-10	2.08803E-10	6.36394E-11	1.65326E-10
423.0	2.84630E-10	1.08207E-10	2.09244E-10	6.39309E-11	1.65756E-10
424.0	2.85068E-10	1.08579E-10	2.09684E-10	6.42223E-11	1.66185E-10
425.0	2.85506E-10	1.08950E-10	2.10122E-10	6.45136E-11	1.66613E-10
426.0	2.85942E-10	1.09321E-10	2.10560E-10	6.48047E-11	1.67040E-10
427.0	2.86377E-10	1.09691E-10	2.10997E-10	6.50956E-11	1.67466E-10
428.0	2.86811E-10	1.10061E-10	2.11432E-10	6.53865E-11	1.67891E-10

429.0	2.87243E-10	1.10430E-10	2.11866E-10	6.56771E-11	1.68315E-10
430.0	2.87675E-10	1.10798E-10	2.12300E-10	6.59676E-11	1.68739E-10
431.0	2.88106E-10	1.11166E-10	2.12732E-10	6.62580E-11	1.69161E-10
432.0	2.88535E-10	1.11533E-10	2.13163E-10	6.65482E-11	1.69583E-10
433.0	2.88963E-10	1.11900E-10	2.13594E-10	6.68382E-11	1.70003E-10
434.0	2.89390E-10	1.12266E-10	2.14023E-10	6.71281E-11	1.70423E-10
435.0	2.89816E-10	1.12631E-10	2.14451E-10	6.74178E-11	1.70841E-10
436.0	2.90241E-10	1.12996E-10	2.14878E-10	6.77073E-11	1.71259E-10
437.0	2.90665E-10	1.13361E-10	2.15304E-10	6.79966E-11	1.71676E-10
438.0	2.91088E-10	1.13724E-10	2.15729E-10	6.82858E-11	1.72092E-10
439.0	2.91509E-10	1.14087E-10	2.16153E-10	6.85748E-11	1.72507E-10
440.0	2.91930E-10	1.14450E-10	2.16576E-10	6.88636E-11	1.72921E-10
441.0	2.92350E-10	1.14812E-10	2.16998E-10	6.91522E-11	1.73334E-10
442.0	2.92768E-10	1.15173E-10	2.17419E-10	6.94406E-11	1.73746E-10
443.0	2.93185E-10	1.15534E-10	2.17839E-10	6.97289E-11	1.74158E-10
444.0	2.93602E-10	1.15894E-10	2.18258E-10	7.00169E-11	1.74568E-10
445.0	2.94017E-10	1.16254E-10	2.18676E-10	7.03048E-11	1.74978E-10
446.0	2.94431E-10	1.16612E-10	2.19092E-10	7.05924E-11	1.75386E-10
447.0	2.94844E-10	1.16971E-10	2.19508E-10	7.08798E-11	1.75794E-10
448.0	2.95257E-10	1.17329E-10	2.19923E-10	7.11671E-11	1.76201E-10
449.0	2.95668E-10	1.17686E-10	2.20337E-10	7.14541E-11	1.76607E-10
450.0	2.96078E-10	1.18042E-10	2.20750E-10	7.17409E-11	1.77012E-10
451.0	2.96487E-10	1.18398E-10	2.21162E-10	7.20275E-11	1.77416E-10
452.0	2.96895E-10	1.18754E-10	2.21573E-10	7.23139E-11	1.77819E-10
453.0	2.97302E-10	1.19108E-10	2.21983E-10	7.26001E-11	1.78221E-10
454.0	2.97708E-10	1.19462E-10	2.22392E-10	7.28860E-11	1.78623E-10
455.0	2.98114E-10	1.19816E-10	2.22800E-10	7.31718E-11	1.79023E-10
456.0	2.98518E-10	1.20169E-10	2.23208E-10	7.34573E-11	1.79423E-10
457.0	2.98921E-10	1.20521E-10	2.23614E-10	7.37426E-11	1.79822E-10
458.0	2.99323E-10	1.20873E-10	2.24019E-10	7.40276E-11	1.80220E-10
459.0	2.99724E-10	1.21224E-10	2.24423E-10	7.43124E-11	1.80617E-10
460.0	3.00124E-10	1.21575E-10	2.24827E-10	7.45970E-11	1.81013E-10
461.0	3.00524E-10	1.21925E-10	2.25229E-10	7.48814E-11	1.81408E-10
462.0	3.00922E-10	1.22274E-10	2.25630E-10	7.51655E-11	1.81803E-10
463.0	3.01319E-10	1.22623E-10	2.26031E-10	7.54493E-11	1.82196E-10
464.0	3.01716E-10	1.22971E-10	2.26430E-10	7.57330E-11	1.82589E-10
465.0	3.02111E-10	1.23318E-10	2.26829E-10	7.60163E-11	1.82981E-10
466.0	3.02506E-10	1.23665E-10	2.27227E-10	7.62995E-11	1.83372E-10
467.0	3.02899E-10	1.24012E-10	2.27624E-10	7.65824E-11	1.83762E-10
468.0	3.03292E-10	1.24357E-10	2.28019E-10	7.68650E-11	1.84151E-10
469.0	3.03684E-10	1.24702E-10	2.28414E-10	7.71474E-11	1.84539E-10
470.0	3.04074E-10	1.25047E-10	2.28808E-10	7.74295E-11	1.84927E-10
471.0	3.04464E-10	1.25391E-10	2.29201E-10	7.77114E-11	1.85314E-10
472.0	3.04853E-10	1.25734E-10	2.29594E-10	7.79930E-11	1.85699E-10
473.0	3.05241E-10	1.26077E-10	2.29985E-10	7.82743E-11	1.86084E-10
474.0	3.05628E-10	1.26419E-10	2.30375E-10	7.85554E-11	1.86468E-10
475.0	3.06015E-10	1.26760E-10	2.30765E-10	7.88362E-11	1.86852E-10
476.0	3.06400E-10	1.27101E-10	2.31153E-10	7.91168E-11	1.87234E-10
477.0	3.06784E-10	1.27442E-10	2.31541E-10	7.93971E-11	1.87616E-10
478.0	3.07168E-10	1.27781E-10	2.31928E-10	7.96771E-11	1.87996E-10

479.0	3.07551E-10	1.28120E-10	2.32314E-10	7.99569E-11	1.88376E-10
480.0	3.07933E-10	1.28459E-10	2.32699E-10	8.02363E-11	1.88755E-10
481.0	3.08313E-10	1.28797E-10	2.33083E-10	8.05155E-11	1.89134E-10
482.0	3.08694E-10	1.29134E-10	2.33466E-10	8.07945E-11	1.89511E-10
483.0	3.09073E-10	1.29471E-10	2.33849E-10	8.10731E-11	1.89888E-10
484.0	3.09451E-10	1.29807E-10	2.34230E-10	8.13515E-11	1.90263E-10
485.0	3.09829E-10	1.30142E-10	2.34611E-10	8.16296E-11	1.90638E-10
486.0	3.10205E-10	1.30477E-10	2.34991E-10	8.19074E-11	1.91012E-10
487.0	3.10581E-10	1.30811E-10	2.35370E-10	8.21849E-11	1.91386E-10
488.0	3.10956E-10	1.31145E-10	2.35748E-10	8.24621E-11	1.91758E-10
489.0	3.11330E-10	1.31478E-10	2.36125E-10	8.27391E-11	1.92130E-10
490.0	3.11703E-10	1.31810E-10	2.36502E-10	8.30157E-11	1.92500E-10
491.0	3.12076E-10	1.32142E-10	2.36877E-10	8.32921E-11	1.92870E-10
492.0	3.12447E-10	1.32474E-10	2.37252E-10	8.35681E-11	1.93240E-10
493.0	3.12818E-10	1.32804E-10	2.37626E-10	8.38439E-11	1.93608E-10
494.0	3.13188E-10	1.33134E-10	2.37999E-10	8.41194E-11	1.93975E-10
495.0	3.13557E-10	1.33464E-10	2.38371E-10	8.43946E-11	1.94342E-10
496.0	3.13926E-10	1.33793E-10	2.38742E-10	8.46695E-11	1.94708E-10
497.0	3.14293E-10	1.34121E-10	2.39113E-10	8.49441E-11	1.95073E-10
498.0	3.14660E-10	1.34448E-10	2.39483E-10	8.52183E-11	1.95438E-10
499.0	3.15026E-10	1.34775E-10	2.39851E-10	8.54923E-11	1.95801E-10
500.0	3.15391E-10	1.35102E-10	2.40219E-10	8.57660E-11	1.96164E-10

Δj = -1 and Δj = -2

T (K)	1--->0	2--->0	2--->1	3--->1
5.0	3.34011E-10	2.31883E-10	4.02768E-10	3.28526E-10
6.0	3.31791E-10	2.24143E-10	4.03948E-10	3.23458E-10
7.0	3.29711E-10	2.18370E-10	4.06274E-10	3.20245E-10
8.0	3.27190E-10	2.13674E-10	4.08880E-10	3.17949E-10
9.0	3.24296E-10	2.09693E-10	4.11517E-10	3.16226E-10
10.0	3.21207E-10	2.06247E-10	4.14100E-10	3.14920E-10
11.0	3.18068E-10	2.03232E-10	4.16595E-10	3.13941E-10
12.0	3.14977E-10	2.00573E-10	4.18988E-10	3.13233E-10
13.0	3.11993E-10	1.98217E-10	4.21273E-10	3.12751E-10
14.0	3.09146E-10	1.96119E-10	4.23455E-10	3.12460E-10
15.0	3.06449E-10	1.94245E-10	4.25542E-10	3.12335E-10
16.0	3.03905E-10	1.92567E-10	4.27543E-10	3.12352E-10
17.0	3.01510E-10	1.91060E-10	4.29468E-10	3.12492E-10
18.0	2.99258E-10	1.89703E-10	4.31325E-10	3.12738E-10
19.0	2.97141E-10	1.88478E-10	4.33123E-10	3.13074E-10
20.0	2.95150E-10	1.87370E-10	4.34865E-10	3.13485E-10
21.0	2.93276E-10	1.86362E-10	4.36555E-10	3.13959E-10
22.0	2.91510E-10	1.85444E-10	4.38196E-10	3.14483E-10
23.0	2.89844E-10	1.84602E-10	4.39785E-10	3.15043E-10
24.0	2.88269E-10	1.83828E-10	4.41324E-10	3.15631E-10
25.0	2.86779E-10	1.83112E-10	4.42811E-10	3.16235E-10
26.0	2.85367E-10	1.82445E-10	4.44243E-10	3.16847E-10
27.0	2.84027E-10	1.81822E-10	4.45620E-10	3.17458E-10
28.0	2.82752E-10	1.81234E-10	4.46940E-10	3.18063E-10
29.0	2.81538E-10	1.80678E-10	4.48201E-10	3.18654E-10

30.0	2.80380E-10	1.80147E-10	4.49403E-10	3.19228E-10
31.0	2.79274E-10	1.79639E-10	4.50545E-10	3.19779E-10
32.0	2.78216E-10	1.79149E-10	4.51627E-10	3.20305E-10
33.0	2.77202E-10	1.78675E-10	4.52649E-10	3.20802E-10
34.0	2.76228E-10	1.78214E-10	4.53611E-10	3.21269E-10
35.0	2.75292E-10	1.77764E-10	4.54516E-10	3.21705E-10
36.0	2.74392E-10	1.77323E-10	4.55363E-10	3.22108E-10
37.0	2.73524E-10	1.76890E-10	4.56154E-10	3.22477E-10
38.0	2.72687E-10	1.76463E-10	4.56891E-10	3.22813E-10
39.0	2.71877E-10	1.76042E-10	4.57575E-10	3.23116E-10
40.0	2.71095E-10	1.75626E-10	4.58209E-10	3.23385E-10
41.0	2.70337E-10	1.75215E-10	4.58794E-10	3.23622E-10
42.0	2.69603E-10	1.74807E-10	4.59332E-10	3.23827E-10
43.0	2.68890E-10	1.74402E-10	4.59826E-10	3.24002E-10
44.0	2.68198E-10	1.74001E-10	4.60277E-10	3.24146E-10
45.0	2.67525E-10	1.73603E-10	4.60687E-10	3.24262E-10
46.0	2.66871E-10	1.73208E-10	4.61059E-10	3.24350E-10
47.0	2.66234E-10	1.72815E-10	4.61394E-10	3.24411E-10
48.0	2.65614E-10	1.72426E-10	4.61693E-10	3.24446E-10
49.0	2.65009E-10	1.72039E-10	4.61960E-10	3.24458E-10
50.0	2.64420E-10	1.71656E-10	4.62195E-10	3.24446E-10
51.0	2.63845E-10	1.71275E-10	4.62401E-10	3.24412E-10
52.0	2.63283E-10	1.70898E-10	4.62578E-10	3.24357E-10
53.0	2.62735E-10	1.70524E-10	4.62728E-10	3.24282E-10
54.0	2.62199E-10	1.70153E-10	4.62853E-10	3.24188E-10
55.0	2.61676E-10	1.69786E-10	4.62955E-10	3.24077E-10
56.0	2.61164E-10	1.69421E-10	4.63033E-10	3.23948E-10
57.0	2.60663E-10	1.69061E-10	4.63091E-10	3.23805E-10
58.0	2.60174E-10	1.68704E-10	4.63128E-10	3.23646E-10
59.0	2.59695E-10	1.68351E-10	4.63146E-10	3.23473E-10
60.0	2.59227E-10	1.68001E-10	4.63146E-10	3.23287E-10
61.0	2.58768E-10	1.67656E-10	4.63128E-10	3.23089E-10
62.0	2.58320E-10	1.67314E-10	4.63095E-10	3.22879E-10
63.0	2.57881E-10	1.66976E-10	4.63046E-10	3.22658E-10
64.0	2.57451E-10	1.66642E-10	4.62983E-10	3.22427E-10
65.0	2.57031E-10	1.66313E-10	4.62906E-10	3.22187E-10
66.0	2.56619E-10	1.65987E-10	4.62815E-10	3.21938E-10
67.0	2.56216E-10	1.65665E-10	4.62713E-10	3.21681E-10
68.0	2.55822E-10	1.65348E-10	4.62598E-10	3.21416E-10
69.0	2.55436E-10	1.65034E-10	4.62473E-10	3.21143E-10
70.0	2.55058E-10	1.64725E-10	4.62337E-10	3.20865E-10
71.0	2.54689E-10	1.64420E-10	4.62191E-10	3.20580E-10
72.0	2.54327E-10	1.64118E-10	4.62035E-10	3.20289E-10
73.0	2.53973E-10	1.63821E-10	4.61870E-10	3.19993E-10
74.0	2.53627E-10	1.63529E-10	4.61697E-10	3.19692E-10
75.0	2.53288E-10	1.63240E-10	4.61516E-10	3.19387E-10
76.0	2.52957E-10	1.62955E-10	4.61326E-10	3.19077E-10
77.0	2.52633E-10	1.62674E-10	4.61130E-10	3.18764E-10
78.0	2.52317E-10	1.62397E-10	4.60926E-10	3.18447E-10
79.0	2.52007E-10	1.62124E-10	4.60716E-10	3.18126E-10

80.0	2.51705E-10	1.61855E-10	4.60499E-10	3.17803E-10
81.0	2.51409E-10	1.61590E-10	4.60277E-10	3.17478E-10
82.0	2.51120E-10	1.61329E-10	4.60049E-10	3.17149E-10
83.0	2.50838E-10	1.61072E-10	4.59815E-10	3.16819E-10
84.0	2.50563E-10	1.60818E-10	4.59576E-10	3.16487E-10
85.0	2.50294E-10	1.60568E-10	4.59332E-10	3.16152E-10
86.0	2.50031E-10	1.60322E-10	4.59084E-10	3.15817E-10
87.0	2.49775E-10	1.60079E-10	4.58831E-10	3.15479E-10
88.0	2.49525E-10	1.59840E-10	4.58574E-10	3.15141E-10
89.0	2.49282E-10	1.59604E-10	4.58313E-10	3.14802E-10
90.0	2.49044E-10	1.59372E-10	4.58049E-10	3.14461E-10
91.0	2.48813E-10	1.59144E-10	4.57780E-10	3.14120E-10
92.0	2.48587E-10	1.58919E-10	4.57509E-10	3.13778E-10
93.0	2.48367E-10	1.58697E-10	4.57234E-10	3.13436E-10
94.0	2.48153E-10	1.58478E-10	4.56956E-10	3.13094E-10
95.0	2.47945E-10	1.58263E-10	4.56676E-10	3.12751E-10
96.0	2.47742E-10	1.58050E-10	4.56393E-10	3.12407E-10
97.0	2.47545E-10	1.57841E-10	4.56107E-10	3.12064E-10
98.0	2.47353E-10	1.57635E-10	4.55819E-10	3.11721E-10
99.0	2.47166E-10	1.57432E-10	4.55529E-10	3.11378E-10
100.0	2.46985E-10	1.57232E-10	4.55236E-10	3.11035E-10
101.0	2.46809E-10	1.57035E-10	4.54942E-10	3.10692E-10
102.0	2.46639E-10	1.56841E-10	4.54646E-10	3.10350E-10
103.0	2.46473E-10	1.56649E-10	4.54348E-10	3.10008E-10
104.0	2.46312E-10	1.56461E-10	4.54049E-10	3.09666E-10
105.0	2.46157E-10	1.56275E-10	4.53748E-10	3.09325E-10
106.0	2.46006E-10	1.56091E-10	4.53446E-10	3.08985E-10
107.0	2.45860E-10	1.55911E-10	4.53143E-10	3.08645E-10
108.0	2.45719E-10	1.55733E-10	4.52839E-10	3.08305E-10
109.0	2.45582E-10	1.55557E-10	4.52534E-10	3.07967E-10
110.0	2.45450E-10	1.55384E-10	4.52228E-10	3.07629E-10
111.0	2.45322E-10	1.55213E-10	4.51921E-10	3.07292E-10
112.0	2.45199E-10	1.55045E-10	4.51613E-10	3.06956E-10
113.0	2.45081E-10	1.54879E-10	4.51305E-10	3.06620E-10
114.0	2.44966E-10	1.54716E-10	4.50996E-10	3.06286E-10
115.0	2.44856E-10	1.54554E-10	4.50687E-10	3.05952E-10
116.0	2.44750E-10	1.54395E-10	4.50377E-10	3.05619E-10
117.0	2.44649E-10	1.54238E-10	4.50067E-10	3.05288E-10
118.0	2.44551E-10	1.54083E-10	4.49757E-10	3.04957E-10
119.0	2.44457E-10	1.53931E-10	4.49447E-10	3.04627E-10
120.0	2.44368E-10	1.53780E-10	4.49136E-10	3.04298E-10
121.0	2.44282E-10	1.53631E-10	4.48826E-10	3.03971E-10
122.0	2.44200E-10	1.53485E-10	4.48516E-10	3.03644E-10
123.0	2.44122E-10	1.53340E-10	4.48206E-10	3.03319E-10
124.0	2.44048E-10	1.53197E-10	4.47896E-10	3.02994E-10
125.0	2.43977E-10	1.53056E-10	4.47586E-10	3.02671E-10
126.0	2.43910E-10	1.52917E-10	4.47277E-10	3.02349E-10
127.0	2.43846E-10	1.52780E-10	4.46968E-10	3.02028E-10
128.0	2.43787E-10	1.52644E-10	4.46659E-10	3.01708E-10
129.0	2.43730E-10	1.52510E-10	4.46351E-10	3.01389E-10

130.0	2.43677E-10	1.52378E-10	4.46043E-10	3.01071E-10
131.0	2.43627E-10	1.52247E-10	4.45736E-10	3.00755E-10
132.0	2.43581E-10	1.52119E-10	4.45430E-10	3.00440E-10
133.0	2.43538E-10	1.51991E-10	4.45124E-10	3.00125E-10
134.0	2.43498E-10	1.51866E-10	4.44819E-10	2.99812E-10
135.0	2.43461E-10	1.51741E-10	4.44515E-10	2.99501E-10
136.0	2.43427E-10	1.51619E-10	4.44211E-10	2.99190E-10
137.0	2.43397E-10	1.51497E-10	4.43908E-10	2.98881E-10
138.0	2.43369E-10	1.51378E-10	4.43607E-10	2.98573E-10
139.0	2.43345E-10	1.51259E-10	4.43306E-10	2.98266E-10
140.0	2.43323E-10	1.51142E-10	4.43006E-10	2.97960E-10
141.0	2.43304E-10	1.51027E-10	4.42706E-10	2.97656E-10
142.0	2.43288E-10	1.50912E-10	4.42408E-10	2.97352E-10
143.0	2.43275E-10	1.50799E-10	4.42111E-10	2.97050E-10
144.0	2.43265E-10	1.50688E-10	4.41815E-10	2.96749E-10
145.0	2.43257E-10	1.50577E-10	4.41520E-10	2.96450E-10
146.0	2.43252E-10	1.50468E-10	4.41226E-10	2.96151E-10
147.0	2.43250E-10	1.50360E-10	4.40934E-10	2.95854E-10
148.0	2.43250E-10	1.50253E-10	4.40642E-10	2.95558E-10
149.0	2.43253E-10	1.50147E-10	4.40352E-10	2.95263E-10
150.0	2.43258E-10	1.50043E-10	4.40062E-10	2.94969E-10
151.0	2.43266E-10	1.49939E-10	4.39774E-10	2.94677E-10
152.0	2.43276E-10	1.49837E-10	4.39488E-10	2.94386E-10
153.0	2.43289E-10	1.49736E-10	4.39202E-10	2.94096E-10
154.0	2.43304E-10	1.49636E-10	4.38918E-10	2.93807E-10
155.0	2.43321E-10	1.49537E-10	4.38635E-10	2.93519E-10
156.0	2.43341E-10	1.49439E-10	4.38353E-10	2.93233E-10
157.0	2.43363E-10	1.49341E-10	4.38073E-10	2.92948E-10
158.0	2.43387E-10	1.49245E-10	4.37794E-10	2.92664E-10
159.0	2.43413E-10	1.49150E-10	4.37516E-10	2.92381E-10
160.0	2.43441E-10	1.49056E-10	4.37240E-10	2.92099E-10
161.0	2.43472E-10	1.48963E-10	4.36965E-10	2.91819E-10
162.0	2.43504E-10	1.48870E-10	4.36692E-10	2.91540E-10
163.0	2.43539E-10	1.48779E-10	4.36420E-10	2.91261E-10
164.0	2.43576E-10	1.48688E-10	4.36149E-10	2.90985E-10
165.0	2.43614E-10	1.48598E-10	4.35880E-10	2.90709E-10
166.0	2.43655E-10	1.48509E-10	4.35612E-10	2.90434E-10
167.0	2.43697E-10	1.48421E-10	4.35346E-10	2.90161E-10
168.0	2.43742E-10	1.48334E-10	4.35081E-10	2.89889E-10
169.0	2.43788E-10	1.48247E-10	4.34818E-10	2.89617E-10
170.0	2.43836E-10	1.48162E-10	4.34556E-10	2.89347E-10
171.0	2.43886E-10	1.48077E-10	4.34296E-10	2.89079E-10
172.0	2.43938E-10	1.47993E-10	4.34037E-10	2.88811E-10
173.0	2.43991E-10	1.47909E-10	4.33779E-10	2.88544E-10
174.0	2.44047E-10	1.47826E-10	4.33523E-10	2.88279E-10
175.0	2.44103E-10	1.47744E-10	4.33269E-10	2.88015E-10
176.0	2.44162E-10	1.47663E-10	4.33016E-10	2.87752E-10
177.0	2.44222E-10	1.47582E-10	4.32764E-10	2.87490E-10
178.0	2.44284E-10	1.47502E-10	4.32514E-10	2.87229E-10
179.0	2.44347E-10	1.47423E-10	4.32266E-10	2.86969E-10

180.0	2.44412E-10	1.47344E-10	4.32019E-10	2.86710E-10
181.0	2.44479E-10	1.47266E-10	4.31773E-10	2.86453E-10
182.0	2.44547E-10	1.47189E-10	4.31530E-10	2.86196E-10
183.0	2.44616E-10	1.47112E-10	4.31287E-10	2.85941E-10
184.0	2.44687E-10	1.47036E-10	4.31046E-10	2.85686E-10
185.0	2.44760E-10	1.46960E-10	4.30807E-10	2.85433E-10
186.0	2.44833E-10	1.46885E-10	4.30569E-10	2.85181E-10
187.0	2.44909E-10	1.46811E-10	4.30333E-10	2.84930E-10
188.0	2.44985E-10	1.46737E-10	4.30098E-10	2.84680E-10
189.0	2.45063E-10	1.46663E-10	4.29865E-10	2.84431E-10
190.0	2.45142E-10	1.46591E-10	4.29633E-10	2.84183E-10
191.0	2.45223E-10	1.46518E-10	4.29403E-10	2.83936E-10
192.0	2.45305E-10	1.46447E-10	4.29174E-10	2.83691E-10
193.0	2.45388E-10	1.46375E-10	4.28947E-10	2.83446E-10
194.0	2.45472E-10	1.46305E-10	4.28721E-10	2.83202E-10
195.0	2.45558E-10	1.46235E-10	4.28497E-10	2.82960E-10
196.0	2.45644E-10	1.46165E-10	4.28274E-10	2.82718E-10
197.0	2.45732E-10	1.46096E-10	4.28053E-10	2.82478E-10
198.0	2.45821E-10	1.46027E-10	4.27834E-10	2.82238E-10
199.0	2.45911E-10	1.45959E-10	4.27616E-10	2.82000E-10
200.0	2.46003E-10	1.45891E-10	4.27399E-10	2.81762E-10
201.0	2.46095E-10	1.45823E-10	4.27184E-10	2.81526E-10
202.0	2.46189E-10	1.45756E-10	4.26970E-10	2.81290E-10
203.0	2.46283E-10	1.45690E-10	4.26758E-10	2.81056E-10
204.0	2.46379E-10	1.45624E-10	4.26547E-10	2.80822E-10
205.0	2.46476E-10	1.45558E-10	4.26338E-10	2.80590E-10
206.0	2.46573E-10	1.45493E-10	4.26130E-10	2.80358E-10
207.0	2.46672E-10	1.45428E-10	4.25924E-10	2.80128E-10
208.0	2.46771E-10	1.45364E-10	4.25719E-10	2.79898E-10
209.0	2.46872E-10	1.45300E-10	4.25516E-10	2.79669E-10
210.0	2.46973E-10	1.45236E-10	4.25314E-10	2.79442E-10
211.0	2.47076E-10	1.45173E-10	4.25113E-10	2.79215E-10
212.0	2.47179E-10	1.45110E-10	4.24914E-10	2.78989E-10
213.0	2.47283E-10	1.45047E-10	4.24717E-10	2.78765E-10
214.0	2.47388E-10	1.44985E-10	4.24521E-10	2.78541E-10
215.0	2.47494E-10	1.44923E-10	4.24326E-10	2.78318E-10
216.0	2.47601E-10	1.44862E-10	4.24133E-10	2.78096E-10
217.0	2.47709E-10	1.44801E-10	4.23941E-10	2.77875E-10
218.0	2.47817E-10	1.44740E-10	4.23751E-10	2.77655E-10
219.0	2.47926E-10	1.44680E-10	4.23562E-10	2.77436E-10
220.0	2.48036E-10	1.44620E-10	4.23374E-10	2.77218E-10
221.0	2.48147E-10	1.44560E-10	4.23188E-10	2.77001E-10
222.0	2.48258E-10	1.44501E-10	4.23003E-10	2.76785E-10
223.0	2.48371E-10	1.44442E-10	4.22819E-10	2.76569E-10
224.0	2.48484E-10	1.44383E-10	4.22637E-10	2.76355E-10
225.0	2.48597E-10	1.44325E-10	4.22457E-10	2.76141E-10
226.0	2.48711E-10	1.44266E-10	4.22277E-10	2.75928E-10
227.0	2.48826E-10	1.44209E-10	4.22099E-10	2.75717E-10
228.0	2.48942E-10	1.44151E-10	4.21923E-10	2.75506E-10
229.0	2.49058E-10	1.44094E-10	4.21747E-10	2.75296E-10

230.0	2.49175E-10	1.44037E-10	4.21573E-10	2.75086E-10
231.0	2.49293E-10	1.43980E-10	4.21401E-10	2.74878E-10
232.0	2.49411E-10	1.43924E-10	4.21230E-10	2.74671E-10
233.0	2.49530E-10	1.43868E-10	4.21060E-10	2.74464E-10
234.0	2.49649E-10	1.43812E-10	4.20891E-10	2.74259E-10
235.0	2.49769E-10	1.43756E-10	4.20724E-10	2.74054E-10
236.0	2.49890E-10	1.43701E-10	4.20558E-10	2.73850E-10
237.0	2.50011E-10	1.43646E-10	4.20393E-10	2.73647E-10
238.0	2.50133E-10	1.43591E-10	4.20229E-10	2.73445E-10
239.0	2.50255E-10	1.43537E-10	4.20067E-10	2.73243E-10
240.0	2.50377E-10	1.43483E-10	4.19906E-10	2.73043E-10
241.0	2.50501E-10	1.43429E-10	4.19747E-10	2.72843E-10
242.0	2.50624E-10	1.43375E-10	4.19588E-10	2.72644E-10
243.0	2.50748E-10	1.43321E-10	4.19431E-10	2.72446E-10
244.0	2.50873E-10	1.43268E-10	4.19276E-10	2.72249E-10
245.0	2.50998E-10	1.43215E-10	4.19121E-10	2.72052E-10
246.0	2.51124E-10	1.43162E-10	4.18968E-10	2.71857E-10
247.0	2.51250E-10	1.43110E-10	4.18815E-10	2.71662E-10
248.0	2.51376E-10	1.43058E-10	4.18665E-10	2.71468E-10
249.0	2.51503E-10	1.43005E-10	4.18515E-10	2.71275E-10
250.0	2.51630E-10	1.42954E-10	4.18366E-10	2.71083E-10
251.0	2.51758E-10	1.42902E-10	4.18219E-10	2.70891E-10
252.0	2.51886E-10	1.42851E-10	4.18073E-10	2.70700E-10
253.0	2.52014E-10	1.42799E-10	4.17928E-10	2.70510E-10
254.0	2.52143E-10	1.42748E-10	4.17784E-10	2.70321E-10
255.0	2.52272E-10	1.42698E-10	4.17642E-10	2.70133E-10
256.0	2.52401E-10	1.42647E-10	4.17500E-10	2.69945E-10
257.0	2.52531E-10	1.42597E-10	4.17360E-10	2.69758E-10
258.0	2.52661E-10	1.42547E-10	4.17221E-10	2.69572E-10
259.0	2.52792E-10	1.42497E-10	4.17083E-10	2.69387E-10
260.0	2.52923E-10	1.42447E-10	4.16946E-10	2.69202E-10
261.0	2.53054E-10	1.42398E-10	4.16811E-10	2.69018E-10
262.0	2.53185E-10	1.42348E-10	4.16676E-10	2.68835E-10
263.0	2.53317E-10	1.42299E-10	4.16543E-10	2.68653E-10
264.0	2.53449E-10	1.42250E-10	4.16411E-10	2.68472E-10
265.0	2.53581E-10	1.42201E-10	4.16280E-10	2.68291E-10
266.0	2.53714E-10	1.42153E-10	4.16149E-10	2.68111E-10
267.0	2.53847E-10	1.42104E-10	4.16021E-10	2.67932E-10
268.0	2.53980E-10	1.42056E-10	4.15893E-10	2.67753E-10
269.0	2.54113E-10	1.42008E-10	4.15766E-10	2.67575E-10
270.0	2.54246E-10	1.41960E-10	4.15640E-10	2.67398E-10
271.0	2.54380E-10	1.41913E-10	4.15516E-10	2.67222E-10
272.0	2.54514E-10	1.41865E-10	4.15392E-10	2.67046E-10
273.0	2.54648E-10	1.41818E-10	4.15269E-10	2.66871E-10
274.0	2.54783E-10	1.41771E-10	4.15148E-10	2.66697E-10
275.0	2.54917E-10	1.41724E-10	4.15028E-10	2.66523E-10
276.0	2.55052E-10	1.41677E-10	4.14908E-10	2.66351E-10
277.0	2.55187E-10	1.41631E-10	4.14790E-10	2.66179E-10
278.0	2.55322E-10	1.41584E-10	4.14673E-10	2.66007E-10
279.0	2.55458E-10	1.41538E-10	4.14556E-10	2.65837E-10

280.0	2.55593E-10	1.41492E-10	4.14441E-10	2.65667E-10
281.0	2.55729E-10	1.41446E-10	4.14327E-10	2.65497E-10
282.0	2.55865E-10	1.41400E-10	4.14214E-10	2.65329E-10
283.0	2.56000E-10	1.41355E-10	4.14101E-10	2.65161E-10
284.0	2.56137E-10	1.41309E-10	4.13990E-10	2.64994E-10
285.0	2.56273E-10	1.41264E-10	4.13880E-10	2.64827E-10
286.0	2.56409E-10	1.41219E-10	4.13770E-10	2.64661E-10
287.0	2.56546E-10	1.41174E-10	4.13662E-10	2.64496E-10
288.0	2.56682E-10	1.41129E-10	4.13555E-10	2.64331E-10
289.0	2.56819E-10	1.41085E-10	4.13448E-10	2.64168E-10
290.0	2.56956E-10	1.41040E-10	4.13343E-10	2.64004E-10
291.0	2.57093E-10	1.40996E-10	4.13238E-10	2.63842E-10
292.0	2.57230E-10	1.40952E-10	4.13135E-10	2.63680E-10
293.0	2.57367E-10	1.40908E-10	4.13032E-10	2.63519E-10
294.0	2.57504E-10	1.40864E-10	4.12930E-10	2.63358E-10
295.0	2.57641E-10	1.40820E-10	4.12830E-10	2.63198E-10
296.0	2.57778E-10	1.40776E-10	4.12730E-10	2.63039E-10
297.0	2.57916E-10	1.40733E-10	4.12631E-10	2.62880E-10
298.0	2.58053E-10	1.40690E-10	4.12533E-10	2.62722E-10
299.0	2.58191E-10	1.40647E-10	4.12436E-10	2.62565E-10
300.0	2.58328E-10	1.40604E-10	4.12340E-10	2.62408E-10
301.0	2.58466E-10	1.40561E-10	4.12244E-10	2.62252E-10
302.0	2.58603E-10	1.40518E-10	4.12150E-10	2.62096E-10
303.0	2.58741E-10	1.40475E-10	4.12056E-10	2.61942E-10
304.0	2.58879E-10	1.40433E-10	4.11964E-10	2.61787E-10
305.0	2.59017E-10	1.40391E-10	4.11872E-10	2.61634E-10
306.0	2.59154E-10	1.40348E-10	4.11781E-10	2.61481E-10
307.0	2.59292E-10	1.40306E-10	4.11691E-10	2.61328E-10
308.0	2.59430E-10	1.40265E-10	4.11602E-10	2.61177E-10
309.0	2.59568E-10	1.40223E-10	4.11513E-10	2.61025E-10
310.0	2.59706E-10	1.40181E-10	4.11426E-10	2.60875E-10
311.0	2.59843E-10	1.40140E-10	4.11339E-10	2.60725E-10
312.0	2.59981E-10	1.40098E-10	4.11253E-10	2.60576E-10
313.0	2.60119E-10	1.40057E-10	4.11168E-10	2.60427E-10
314.0	2.60257E-10	1.40016E-10	4.11084E-10	2.60279E-10
315.0	2.60395E-10	1.39975E-10	4.11000E-10	2.60131E-10
316.0	2.60532E-10	1.39934E-10	4.10918E-10	2.59984E-10
317.0	2.60670E-10	1.39893E-10	4.10836E-10	2.59838E-10
318.0	2.60808E-10	1.39853E-10	4.10755E-10	2.59692E-10
319.0	2.60946E-10	1.39812E-10	4.10675E-10	2.59547E-10
320.0	2.61083E-10	1.39772E-10	4.10596E-10	2.59402E-10
321.0	2.61221E-10	1.39732E-10	4.10517E-10	2.59258E-10
322.0	2.61358E-10	1.39692E-10	4.10439E-10	2.59114E-10
323.0	2.61496E-10	1.39652E-10	4.10362E-10	2.58971E-10
324.0	2.61633E-10	1.39612E-10	4.10286E-10	2.58829E-10
325.0	2.61771E-10	1.39572E-10	4.10211E-10	2.58687E-10
326.0	2.61908E-10	1.39532E-10	4.10136E-10	2.58546E-10
327.0	2.62046E-10	1.39493E-10	4.10062E-10	2.58405E-10
328.0	2.62183E-10	1.39454E-10	4.09989E-10	2.58265E-10
329.0	2.62320E-10	1.39414E-10	4.09916E-10	2.58125E-10

330.0	2.62457E-10	1.39375E-10	4.09845E-10	2.57986E-10
331.0	2.62594E-10	1.39336E-10	4.09774E-10	2.57848E-10
332.0	2.62731E-10	1.39297E-10	4.09703E-10	2.57710E-10
333.0	2.62868E-10	1.39258E-10	4.09634E-10	2.57572E-10
334.0	2.63005E-10	1.39220E-10	4.09565E-10	2.57436E-10
335.0	2.63142E-10	1.39181E-10	4.09497E-10	2.57299E-10
336.0	2.63279E-10	1.39143E-10	4.09430E-10	2.57163E-10
337.0	2.63415E-10	1.39104E-10	4.09363E-10	2.57028E-10
338.0	2.63552E-10	1.39066E-10	4.09297E-10	2.56893E-10
339.0	2.63688E-10	1.39028E-10	4.09232E-10	2.56759E-10
340.0	2.63825E-10	1.38990E-10	4.09168E-10	2.56626E-10
341.0	2.63961E-10	1.38952E-10	4.09104E-10	2.56492E-10
342.0	2.64097E-10	1.38914E-10	4.09041E-10	2.56360E-10
343.0	2.64233E-10	1.38877E-10	4.08979E-10	2.56228E-10
344.0	2.64369E-10	1.38839E-10	4.08917E-10	2.56096E-10
345.0	2.64505E-10	1.38802E-10	4.08856E-10	2.55965E-10
346.0	2.64641E-10	1.38765E-10	4.08795E-10	2.55834E-10
347.0	2.64776E-10	1.38727E-10	4.08736E-10	2.55704E-10
348.0	2.64912E-10	1.38690E-10	4.08677E-10	2.55575E-10
349.0	2.65047E-10	1.38653E-10	4.08618E-10	2.55446E-10
350.0	2.65183E-10	1.38616E-10	4.08561E-10	2.55317E-10
351.0	2.65318E-10	1.38580E-10	4.08504E-10	2.55189E-10
352.0	2.65453E-10	1.38543E-10	4.08447E-10	2.55062E-10
353.0	2.65588E-10	1.38506E-10	4.08392E-10	2.54935E-10
354.0	2.65723E-10	1.38470E-10	4.08337E-10	2.54808E-10
355.0	2.65857E-10	1.38433E-10	4.08282E-10	2.54682E-10
356.0	2.65992E-10	1.38397E-10	4.08228E-10	2.54556E-10
357.0	2.66126E-10	1.38361E-10	4.08175E-10	2.54431E-10
358.0	2.66261E-10	1.38325E-10	4.08123E-10	2.54307E-10
359.0	2.66395E-10	1.38289E-10	4.08071E-10	2.54183E-10
360.0	2.66529E-10	1.38253E-10	4.08020E-10	2.54059E-10
361.0	2.66663E-10	1.38217E-10	4.07969E-10	2.53936E-10
362.0	2.66797E-10	1.38182E-10	4.07919E-10	2.53813E-10
363.0	2.66930E-10	1.38146E-10	4.07869E-10	2.53691E-10
364.0	2.67064E-10	1.38111E-10	4.07821E-10	2.53569E-10
365.0	2.67197E-10	1.38075E-10	4.07772E-10	2.53448E-10
366.0	2.67330E-10	1.38040E-10	4.07725E-10	2.53327E-10
367.0	2.67463E-10	1.38005E-10	4.07678E-10	2.53207E-10
368.0	2.67596E-10	1.37970E-10	4.07631E-10	2.53087E-10
369.0	2.67729E-10	1.37935E-10	4.07585E-10	2.52967E-10
370.0	2.67862E-10	1.37900E-10	4.07540E-10	2.52848E-10
371.0	2.67994E-10	1.37865E-10	4.07495E-10	2.52730E-10
372.0	2.68127E-10	1.37831E-10	4.07451E-10	2.52612E-10
373.0	2.68259E-10	1.37796E-10	4.07408E-10	2.52494E-10
374.0	2.68391E-10	1.37762E-10	4.07365E-10	2.52377E-10
375.0	2.68523E-10	1.37727E-10	4.07323E-10	2.52260E-10
376.0	2.68654E-10	1.37693E-10	4.07281E-10	2.52144E-10
377.0	2.68786E-10	1.37659E-10	4.07239E-10	2.52028E-10
378.0	2.68917E-10	1.37625E-10	4.07199E-10	2.51913E-10
379.0	2.69048E-10	1.37591E-10	4.07159E-10	2.51798E-10

380.0	2.69179E-10	1.37557E-10	4.07119E-10	2.51684E-10
381.0	2.69310E-10	1.37523E-10	4.07080E-10	2.51570E-10
382.0	2.69441E-10	1.37489E-10	4.07041E-10	2.51456E-10
383.0	2.69572E-10	1.37456E-10	4.07004E-10	2.51343E-10
384.0	2.69702E-10	1.37422E-10	4.06966E-10	2.51230E-10
385.0	2.69832E-10	1.37389E-10	4.06929E-10	2.51118E-10
386.0	2.69962E-10	1.37356E-10	4.06893E-10	2.51006E-10
387.0	2.70092E-10	1.37322E-10	4.06857E-10	2.50894E-10
388.0	2.70222E-10	1.37289E-10	4.06822E-10	2.50783E-10
389.0	2.70351E-10	1.37256E-10	4.06787E-10	2.50673E-10
390.0	2.70481E-10	1.37223E-10	4.06753E-10	2.50562E-10
391.0	2.70610E-10	1.37190E-10	4.06719E-10	2.50453E-10
392.0	2.70739E-10	1.37157E-10	4.06686E-10	2.50343E-10
393.0	2.70868E-10	1.37125E-10	4.06653E-10	2.50234E-10
394.0	2.70996E-10	1.37092E-10	4.06621E-10	2.50126E-10
395.0	2.71125E-10	1.37060E-10	4.06589E-10	2.50017E-10
396.0	2.71253E-10	1.37027E-10	4.06558E-10	2.49910E-10
397.0	2.71381E-10	1.36995E-10	4.06527E-10	2.49802E-10
398.0	2.71509E-10	1.36963E-10	4.06497E-10	2.49695E-10
399.0	2.71637E-10	1.36930E-10	4.06467E-10	2.49589E-10
400.0	2.71765E-10	1.36898E-10	4.06438E-10	2.49483E-10
401.0	2.71892E-10	1.36866E-10	4.06409E-10	2.49377E-10
402.0	2.72019E-10	1.36834E-10	4.06381E-10	2.49272E-10
403.0	2.72146E-10	1.36803E-10	4.06353E-10	2.49167E-10
404.0	2.72273E-10	1.36771E-10	4.06326E-10	2.49062E-10
405.0	2.72400E-10	1.36739E-10	4.06299E-10	2.48958E-10
406.0	2.72527E-10	1.36708E-10	4.06273E-10	2.48854E-10
407.0	2.72653E-10	1.36676E-10	4.06247E-10	2.48751E-10
408.0	2.72779E-10	1.36645E-10	4.06222E-10	2.48648E-10
409.0	2.72905E-10	1.36613E-10	4.06197E-10	2.48545E-10
410.0	2.73031E-10	1.36582E-10	4.06172E-10	2.48443E-10
411.0	2.73156E-10	1.36551E-10	4.06148E-10	2.48341E-10
412.0	2.73282E-10	1.36520E-10	4.06125E-10	2.48240E-10
413.0	2.73407E-10	1.36489E-10	4.06101E-10	2.48139E-10
414.0	2.73532E-10	1.36458E-10	4.06079E-10	2.48038E-10
415.0	2.73657E-10	1.36427E-10	4.06056E-10	2.47938E-10
416.0	2.73782E-10	1.36396E-10	4.06035E-10	2.47838E-10
417.0	2.73906E-10	1.36366E-10	4.06013E-10	2.47738E-10
418.0	2.74030E-10	1.36335E-10	4.05992E-10	2.47639E-10
419.0	2.74154E-10	1.36305E-10	4.05972E-10	2.47540E-10
420.0	2.74278E-10	1.36274E-10	4.05952E-10	2.47441E-10
421.0	2.74402E-10	1.36244E-10	4.05932E-10	2.47343E-10
422.0	2.74525E-10	1.36214E-10	4.05913E-10	2.47246E-10
423.0	2.74649E-10	1.36183E-10	4.05895E-10	2.47148E-10
424.0	2.74772E-10	1.36153E-10	4.05876E-10	2.47051E-10
425.0	2.74895E-10	1.36123E-10	4.05858E-10	2.46954E-10
426.0	2.75018E-10	1.36093E-10	4.05841E-10	2.46858E-10
427.0	2.75140E-10	1.36063E-10	4.05824E-10	2.46762E-10
428.0	2.75263E-10	1.36034E-10	4.05807E-10	2.46666E-10
429.0	2.75385E-10	1.36004E-10	4.05791E-10	2.46571E-10

430.0	2.75507E-10	1.35974E-10	4.05775E-10	2.46476E-10
431.0	2.75628E-10	1.35945E-10	4.05760E-10	2.46382E-10
432.0	2.75750E-10	1.35915E-10	4.05745E-10	2.46287E-10
433.0	2.75871E-10	1.35886E-10	4.05730E-10	2.46194E-10
434.0	2.75993E-10	1.35857E-10	4.05716E-10	2.46100E-10
435.0	2.76114E-10	1.35827E-10	4.05702E-10	2.46007E-10
436.0	2.76235E-10	1.35798E-10	4.05689E-10	2.45914E-10
437.0	2.76355E-10	1.35769E-10	4.05676E-10	2.45821E-10
438.0	2.76476E-10	1.35740E-10	4.05663E-10	2.45729E-10
439.0	2.76596E-10	1.35711E-10	4.05651E-10	2.45637E-10
440.0	2.76716E-10	1.35682E-10	4.05639E-10	2.45546E-10
441.0	2.76836E-10	1.35653E-10	4.05628E-10	2.45455E-10
442.0	2.76955E-10	1.35625E-10	4.05617E-10	2.45364E-10
443.0	2.77075E-10	1.35596E-10	4.05606E-10	2.45273E-10
444.0	2.77194E-10	1.35567E-10	4.05596E-10	2.45183E-10
445.0	2.77313E-10	1.35539E-10	4.05586E-10	2.45093E-10
446.0	2.77432E-10	1.35510E-10	4.05576E-10	2.45004E-10
447.0	2.77551E-10	1.35482E-10	4.05567E-10	2.44914E-10
448.0	2.77669E-10	1.35454E-10	4.05558E-10	2.44825E-10
449.0	2.77788E-10	1.35426E-10	4.05550E-10	2.44737E-10
450.0	2.77906E-10	1.35397E-10	4.05541E-10	2.44649E-10
451.0	2.78024E-10	1.35369E-10	4.05534E-10	2.44561E-10
452.0	2.78141E-10	1.35341E-10	4.05526E-10	2.44473E-10
453.0	2.78259E-10	1.35313E-10	4.05519E-10	2.44386E-10
454.0	2.78376E-10	1.35286E-10	4.05513E-10	2.44299E-10
455.0	2.78493E-10	1.35258E-10	4.05506E-10	2.44212E-10
456.0	2.78610E-10	1.35230E-10	4.05500E-10	2.44126E-10
457.0	2.78727E-10	1.35202E-10	4.05495E-10	2.44039E-10
458.0	2.78844E-10	1.35175E-10	4.05489E-10	2.43954E-10
459.0	2.78960E-10	1.35147E-10	4.05485E-10	2.43868E-10
460.0	2.79076E-10	1.35120E-10	4.05480E-10	2.43783E-10
461.0	2.79192E-10	1.35092E-10	4.05476E-10	2.43698E-10
462.0	2.79308E-10	1.35065E-10	4.05472E-10	2.43614E-10
463.0	2.79424E-10	1.35038E-10	4.05468E-10	2.43529E-10
464.0	2.79539E-10	1.35011E-10	4.05465E-10	2.43445E-10
465.0	2.79654E-10	1.34984E-10	4.05462E-10	2.43362E-10
466.0	2.79769E-10	1.34957E-10	4.05459E-10	2.43278E-10
467.0	2.79884E-10	1.34930E-10	4.05457E-10	2.43195E-10
468.0	2.79999E-10	1.34903E-10	4.05455E-10	2.43113E-10
469.0	2.80113E-10	1.34876E-10	4.05454E-10	2.43030E-10
470.0	2.80227E-10	1.34849E-10	4.05452E-10	2.42948E-10
471.0	2.80341E-10	1.34822E-10	4.05452E-10	2.42866E-10
472.0	2.80455E-10	1.34796E-10	4.05451E-10	2.42784E-10
473.0	2.80569E-10	1.34769E-10	4.05451E-10	2.42703E-10
474.0	2.80682E-10	1.34743E-10	4.05451E-10	2.42622E-10
475.0	2.80795E-10	1.34716E-10	4.05451E-10	2.42541E-10
476.0	2.80909E-10	1.34690E-10	4.05451E-10	2.42461E-10
477.0	2.81021E-10	1.34664E-10	4.05452E-10	2.42381E-10
478.0	2.81134E-10	1.34637E-10	4.05454E-10	2.42301E-10
479.0	2.81247E-10	1.34611E-10	4.05455E-10	2.42221E-10

480.0	2.81359E-10	1.34585E-10	4.05457E-10	2.42142E-10
481.0	2.81471E-10	1.34559E-10	4.05459E-10	2.42063E-10
482.0	2.81583E-10	1.34533E-10	4.05462E-10	2.41984E-10
483.0	2.81695E-10	1.34507E-10	4.05464E-10	2.41905E-10
484.0	2.81806E-10	1.34481E-10	4.05467E-10	2.41827E-10
485.0	2.81918E-10	1.34455E-10	4.05471E-10	2.41749E-10
486.0	2.82029E-10	1.34430E-10	4.05474E-10	2.41671E-10
487.0	2.82140E-10	1.34404E-10	4.05478E-10	2.41594E-10
488.0	2.82251E-10	1.34378E-10	4.05482E-10	2.41517E-10
489.0	2.82361E-10	1.34353E-10	4.05487E-10	2.41440E-10
490.0	2.82472E-10	1.34327E-10	4.05492E-10	2.41363E-10
491.0	2.82582E-10	1.34302E-10	4.05497E-10	2.41287E-10
492.0	2.82692E-10	1.34276E-10	4.05502E-10	2.41211E-10
493.0	2.82802E-10	1.34251E-10	4.05508E-10	2.41135E-10
494.0	2.82911E-10	1.34226E-10	4.05514E-10	2.41059E-10
495.0	2.83021E-10	1.34201E-10	4.05520E-10	2.40984E-10
496.0	2.83130E-10	1.34175E-10	4.05526E-10	2.40909E-10
497.0	2.83239E-10	1.34150E-10	4.05533E-10	2.40834E-10
498.0	2.83348E-10	1.34125E-10	4.05540E-10	2.40759E-10
499.0	2.83457E-10	1.34100E-10	4.05547E-10	2.40685E-10
500.0	2.83565E-10	1.34075E-10	4.05555E-10	2.40611E-10

T (K)	3--->2	4--->2	4--->3
5.0	4.77887E-10	4.05237E-10	3.75768E-10
6.0	4.76125E-10	3.91622E-10	3.76301E-10
7.0	4.74963E-10	3.81226E-10	3.77163E-10
8.0	4.73727E-10	3.72873E-10	3.78000E-10
9.0	4.72376E-10	3.65999E-10	3.78778E-10
10.0	4.70992E-10	3.60273E-10	3.79529E-10
11.0	4.69652E-10	3.55472E-10	3.80282E-10
12.0	4.68410E-10	3.51429E-10	3.81050E-10
13.0	4.67295E-10	3.48017E-10	3.81840E-10
14.0	4.66324E-10	3.45131E-10	3.82652E-10
15.0	4.65500E-10	3.42691E-10	3.83483E-10
16.0	4.64825E-10	3.40628E-10	3.84330E-10
17.0	4.64292E-10	3.38890E-10	3.85190E-10
18.0	4.63892E-10	3.37430E-10	3.86060E-10
19.0	4.63617E-10	3.36212E-10	3.86937E-10
20.0	4.63453E-10	3.35205E-10	3.87819E-10
21.0	4.63388E-10	3.34382E-10	3.88704E-10
22.0	4.63409E-10	3.33721E-10	3.89592E-10
23.0	4.63502E-10	3.33201E-10	3.90478E-10
24.0	4.63654E-10	3.32806E-10	3.91363E-10
25.0	4.63854E-10	3.32520E-10	3.92245E-10
26.0	4.64091E-10	3.32329E-10	3.93121E-10
27.0	4.64354E-10	3.32223E-10	3.93990E-10
28.0	4.64635E-10	3.32189E-10	3.94851E-10
29.0	4.64925E-10	3.32219E-10	3.95701E-10
30.0	4.65218E-10	3.32304E-10	3.96539E-10
31.0	4.65508E-10	3.32435E-10	3.97364E-10

32.0	4.65790E-10	3.32606E-10	3.98175E-10
33.0	4.66060E-10	3.32811E-10	3.98971E-10
34.0	4.66316E-10	3.33045E-10	3.99750E-10
35.0	4.66554E-10	3.33301E-10	4.00511E-10
36.0	4.66772E-10	3.33576E-10	4.01255E-10
37.0	4.66970E-10	3.33866E-10	4.01980E-10
38.0	4.67146E-10	3.34167E-10	4.02687E-10
39.0	4.67300E-10	3.34477E-10	4.03374E-10
40.0	4.67432E-10	3.34792E-10	4.04042E-10
41.0	4.67541E-10	3.35110E-10	4.04691E-10
42.0	4.67628E-10	3.35430E-10	4.05321E-10
43.0	4.67693E-10	3.35749E-10	4.05931E-10
44.0	4.67737E-10	3.36065E-10	4.06523E-10
45.0	4.67761E-10	3.36378E-10	4.07096E-10
46.0	4.67765E-10	3.36687E-10	4.07650E-10
47.0	4.67750E-10	3.36990E-10	4.08187E-10
48.0	4.67716E-10	3.37286E-10	4.08706E-10
49.0	4.67665E-10	3.37575E-10	4.09208E-10
50.0	4.67598E-10	3.37856E-10	4.09693E-10
51.0	4.67515E-10	3.38129E-10	4.10162E-10
52.0	4.67417E-10	3.38394E-10	4.10615E-10
53.0	4.67305E-10	3.38649E-10	4.11053E-10
54.0	4.67180E-10	3.38896E-10	4.11476E-10
55.0	4.67043E-10	3.39133E-10	4.11885E-10
56.0	4.66893E-10	3.39361E-10	4.12280E-10
57.0	4.66733E-10	3.39579E-10	4.12662E-10
58.0	4.66563E-10	3.39788E-10	4.13031E-10
59.0	4.66382E-10	3.39988E-10	4.13387E-10
60.0	4.66193E-10	3.40178E-10	4.13732E-10
61.0	4.65995E-10	3.40359E-10	4.14065E-10
62.0	4.65790E-10	3.40531E-10	4.14386E-10
63.0	4.65577E-10	3.40693E-10	4.14697E-10
64.0	4.65357E-10	3.40847E-10	4.14998E-10
65.0	4.65131E-10	3.40992E-10	4.15288E-10
66.0	4.64899E-10	3.41129E-10	4.15569E-10
67.0	4.64661E-10	3.41257E-10	4.15841E-10
68.0	4.64418E-10	3.41377E-10	4.16104E-10
69.0	4.64170E-10	3.41489E-10	4.16358E-10
70.0	4.63918E-10	3.41593E-10	4.16604E-10
71.0	4.63662E-10	3.41689E-10	4.16842E-10
72.0	4.63402E-10	3.41778E-10	4.17072E-10
73.0	4.63139E-10	3.41859E-10	4.17294E-10
74.0	4.62872E-10	3.41933E-10	4.17510E-10
75.0	4.62602E-10	3.42001E-10	4.17718E-10
76.0	4.62329E-10	3.42061E-10	4.17920E-10
77.0	4.62054E-10	3.42115E-10	4.18115E-10
78.0	4.61777E-10	3.42163E-10	4.18304E-10
79.0	4.61497E-10	3.42204E-10	4.18486E-10
80.0	4.61215E-10	3.42240E-10	4.18663E-10
81.0	4.60932E-10	3.42269E-10	4.18834E-10

82.0	4.60646E-10	3.42293E-10	4.19000E-10
83.0	4.60360E-10	3.42311E-10	4.19160E-10
84.0	4.60072E-10	3.42324E-10	4.19314E-10
85.0	4.59782E-10	3.42332E-10	4.19464E-10
86.0	4.59492E-10	3.42335E-10	4.19609E-10
87.0	4.59200E-10	3.42332E-10	4.19749E-10
88.0	4.58908E-10	3.42325E-10	4.19884E-10
89.0	4.58615E-10	3.42313E-10	4.20015E-10
90.0	4.58321E-10	3.42297E-10	4.20141E-10
91.0	4.58026E-10	3.42277E-10	4.20263E-10
92.0	4.57731E-10	3.42252E-10	4.20381E-10
93.0	4.57436E-10	3.42223E-10	4.20494E-10
94.0	4.57140E-10	3.42190E-10	4.20604E-10
95.0	4.56843E-10	3.42153E-10	4.20710E-10
96.0	4.56547E-10	3.42112E-10	4.20811E-10
97.0	4.56250E-10	3.42068E-10	4.20910E-10
98.0	4.55953E-10	3.42020E-10	4.21004E-10
99.0	4.55656E-10	3.41969E-10	4.21095E-10
100.0	4.55359E-10	3.41914E-10	4.21182E-10
101.0	4.55062E-10	3.41856E-10	4.21266E-10
102.0	4.54765E-10	3.41795E-10	4.21347E-10
103.0	4.54468E-10	3.41731E-10	4.21424E-10
104.0	4.54172E-10	3.41664E-10	4.21498E-10
105.0	4.53875E-10	3.41595E-10	4.21569E-10
106.0	4.53579E-10	3.41522E-10	4.21637E-10
107.0	4.53283E-10	3.41447E-10	4.21702E-10
108.0	4.52987E-10	3.41369E-10	4.21764E-10
109.0	4.52692E-10	3.41288E-10	4.21823E-10
110.0	4.52397E-10	3.41205E-10	4.21879E-10
111.0	4.52102E-10	3.41119E-10	4.21932E-10
112.0	4.51808E-10	3.41032E-10	4.21983E-10
113.0	4.51514E-10	3.40942E-10	4.22031E-10
114.0	4.51221E-10	3.40849E-10	4.22076E-10
115.0	4.50928E-10	3.40755E-10	4.22119E-10
116.0	4.50636E-10	3.40658E-10	4.22159E-10
117.0	4.50344E-10	3.40560E-10	4.22197E-10
118.0	4.50053E-10	3.40459E-10	4.22232E-10
119.0	4.49763E-10	3.40357E-10	4.22264E-10
120.0	4.49473E-10	3.40252E-10	4.22295E-10
121.0	4.49183E-10	3.40146E-10	4.22323E-10
122.0	4.48895E-10	3.40039E-10	4.22349E-10
123.0	4.48607E-10	3.39929E-10	4.22372E-10
124.0	4.48319E-10	3.39818E-10	4.22393E-10
125.0	4.48033E-10	3.39705E-10	4.22412E-10
126.0	4.47747E-10	3.39591E-10	4.22429E-10
127.0	4.47462E-10	3.39475E-10	4.22444E-10
128.0	4.47177E-10	3.39357E-10	4.22457E-10
129.0	4.46894E-10	3.39239E-10	4.22468E-10
130.0	4.46611E-10	3.39118E-10	4.22477E-10
131.0	4.46328E-10	3.38997E-10	4.22484E-10

132.0	4.46047E-10	3.38874E-10	4.22489E-10
133.0	4.45767E-10	3.38750E-10	4.22492E-10
134.0	4.45487E-10	3.38625E-10	4.22493E-10
135.0	4.45208E-10	3.38498E-10	4.22492E-10
136.0	4.44930E-10	3.38370E-10	4.22490E-10
137.0	4.44653E-10	3.38242E-10	4.22486E-10
138.0	4.44376E-10	3.38112E-10	4.22480E-10
139.0	4.44101E-10	3.37981E-10	4.22472E-10
140.0	4.43826E-10	3.37849E-10	4.22463E-10
141.0	4.43553E-10	3.37715E-10	4.22452E-10
142.0	4.43280E-10	3.37581E-10	4.22440E-10
143.0	4.43008E-10	3.37446E-10	4.22426E-10
144.0	4.42737E-10	3.37311E-10	4.22410E-10
145.0	4.42467E-10	3.37174E-10	4.22393E-10
146.0	4.42198E-10	3.37036E-10	4.22375E-10
147.0	4.41930E-10	3.36898E-10	4.22355E-10
148.0	4.41663E-10	3.36758E-10	4.22333E-10
149.0	4.41396E-10	3.36618E-10	4.22310E-10
150.0	4.41131E-10	3.36477E-10	4.22286E-10
151.0	4.40867E-10	3.36336E-10	4.22261E-10
152.0	4.40604E-10	3.36193E-10	4.22234E-10
153.0	4.40341E-10	3.36050E-10	4.22206E-10
154.0	4.40080E-10	3.35907E-10	4.22176E-10
155.0	4.39820E-10	3.35762E-10	4.22145E-10
156.0	4.39560E-10	3.35617E-10	4.22113E-10
157.0	4.39302E-10	3.35472E-10	4.22080E-10
158.0	4.39045E-10	3.35325E-10	4.22046E-10
159.0	4.38788E-10	3.35179E-10	4.22010E-10
160.0	4.38533E-10	3.35031E-10	4.21974E-10
161.0	4.38279E-10	3.34884E-10	4.21936E-10
162.0	4.38026E-10	3.34735E-10	4.21897E-10
163.0	4.37774E-10	3.34586E-10	4.21857E-10
164.0	4.37522E-10	3.34437E-10	4.21817E-10
165.0	4.37272E-10	3.34287E-10	4.21775E-10
166.0	4.37023E-10	3.34137E-10	4.21732E-10
167.0	4.36775E-10	3.33986E-10	4.21688E-10
168.0	4.36528E-10	3.33835E-10	4.21643E-10
169.0	4.36282E-10	3.33684E-10	4.21597E-10
170.0	4.36038E-10	3.33532E-10	4.21551E-10
171.0	4.35794E-10	3.33380E-10	4.21503E-10
172.0	4.35551E-10	3.33227E-10	4.21455E-10
173.0	4.35310E-10	3.33074E-10	4.21405E-10
174.0	4.35069E-10	3.32921E-10	4.21355E-10
175.0	4.34830E-10	3.32767E-10	4.21305E-10
176.0	4.34591E-10	3.32613E-10	4.21253E-10
177.0	4.34354E-10	3.32459E-10	4.21200E-10
178.0	4.34118E-10	3.32305E-10	4.21147E-10
179.0	4.33882E-10	3.32150E-10	4.21093E-10
180.0	4.33648E-10	3.31995E-10	4.21039E-10
181.0	4.33415E-10	3.31840E-10	4.20983E-10

182.0	4.33183E-10	3.31684E-10	4.20927E-10
183.0	4.32952E-10	3.31529E-10	4.20871E-10
184.0	4.32723E-10	3.31373E-10	4.20813E-10
185.0	4.32494E-10	3.31217E-10	4.20755E-10
186.0	4.32266E-10	3.31061E-10	4.20697E-10
187.0	4.32040E-10	3.30904E-10	4.20638E-10
188.0	4.31815E-10	3.30748E-10	4.20578E-10
189.0	4.31590E-10	3.30591E-10	4.20518E-10
190.0	4.31367E-10	3.30434E-10	4.20457E-10
191.0	4.31145E-10	3.30277E-10	4.20395E-10
192.0	4.30924E-10	3.30120E-10	4.20333E-10
193.0	4.30704E-10	3.29963E-10	4.20271E-10
194.0	4.30485E-10	3.29806E-10	4.20208E-10
195.0	4.30267E-10	3.29648E-10	4.20145E-10
196.0	4.30051E-10	3.29491E-10	4.20081E-10
197.0	4.29835E-10	3.29333E-10	4.20017E-10
198.0	4.29621E-10	3.29176E-10	4.19952E-10
199.0	4.29407E-10	3.29018E-10	4.19887E-10
200.0	4.29195E-10	3.28861E-10	4.19822E-10
201.0	4.28984E-10	3.28703E-10	4.19756E-10
202.0	4.28773E-10	3.28545E-10	4.19690E-10
203.0	4.28564E-10	3.28387E-10	4.19623E-10
204.0	4.28356E-10	3.28229E-10	4.19556E-10
205.0	4.28150E-10	3.28072E-10	4.19489E-10
206.0	4.27944E-10	3.27914E-10	4.19421E-10
207.0	4.27739E-10	3.27756E-10	4.19353E-10
208.0	4.27535E-10	3.27598E-10	4.19285E-10
209.0	4.27333E-10	3.27440E-10	4.19217E-10
210.0	4.27131E-10	3.27283E-10	4.19148E-10
211.0	4.26931E-10	3.27125E-10	4.19079E-10
212.0	4.26732E-10	3.26967E-10	4.19010E-10
213.0	4.26533E-10	3.26809E-10	4.18940E-10
214.0	4.26336E-10	3.26652E-10	4.18871E-10
215.0	4.26140E-10	3.26494E-10	4.18801E-10
216.0	4.25945E-10	3.26337E-10	4.18731E-10
217.0	4.25751E-10	3.26179E-10	4.18660E-10
218.0	4.25558E-10	3.26022E-10	4.18590E-10
219.0	4.25366E-10	3.25865E-10	4.18519E-10
220.0	4.25176E-10	3.25707E-10	4.18448E-10
221.0	4.24986E-10	3.25550E-10	4.18377E-10
222.0	4.24797E-10	3.25393E-10	4.18306E-10
223.0	4.24610E-10	3.25236E-10	4.18235E-10
224.0	4.24423E-10	3.25080E-10	4.18163E-10
225.0	4.24238E-10	3.24923E-10	4.18092E-10
226.0	4.24053E-10	3.24766E-10	4.18020E-10
227.0	4.23870E-10	3.24610E-10	4.17949E-10
228.0	4.23687E-10	3.24453E-10	4.17877E-10
229.0	4.23506E-10	3.24297E-10	4.17805E-10
230.0	4.23326E-10	3.24141E-10	4.17733E-10
231.0	4.23147E-10	3.23985E-10	4.17661E-10

232.0	4.22969E-10	3.23829E-10	4.17589E-10
233.0	4.22791E-10	3.23674E-10	4.17517E-10
234.0	4.22615E-10	3.23518E-10	4.17445E-10
235.0	4.22440E-10	3.23363E-10	4.17372E-10
236.0	4.22266E-10	3.23208E-10	4.17300E-10
237.0	4.22093E-10	3.23053E-10	4.17228E-10
238.0	4.21921E-10	3.22898E-10	4.17156E-10
239.0	4.21750E-10	3.22743E-10	4.17084E-10
240.0	4.21580E-10	3.22589E-10	4.17011E-10
241.0	4.21411E-10	3.22434E-10	4.16939E-10
242.0	4.21243E-10	3.22280E-10	4.16867E-10
243.0	4.21076E-10	3.22126E-10	4.16795E-10
244.0	4.20911E-10	3.21972E-10	4.16723E-10
245.0	4.20746E-10	3.21819E-10	4.16651E-10
246.0	4.20582E-10	3.21665E-10	4.16579E-10
247.0	4.20419E-10	3.21512E-10	4.16507E-10
248.0	4.20257E-10	3.21359E-10	4.16435E-10
249.0	4.20096E-10	3.21206E-10	4.16363E-10
250.0	4.19936E-10	3.21053E-10	4.16291E-10
251.0	4.19777E-10	3.20901E-10	4.16219E-10
252.0	4.19619E-10	3.20749E-10	4.16148E-10
253.0	4.19462E-10	3.20597E-10	4.16076E-10
254.0	4.19306E-10	3.20445E-10	4.16005E-10
255.0	4.19151E-10	3.20293E-10	4.15933E-10
256.0	4.18997E-10	3.20142E-10	4.15862E-10
257.0	4.18844E-10	3.19991E-10	4.15791E-10
258.0	4.18692E-10	3.19840E-10	4.15720E-10
259.0	4.18541E-10	3.19689E-10	4.15649E-10
260.0	4.18391E-10	3.19539E-10	4.15578E-10
261.0	4.18241E-10	3.19389E-10	4.15508E-10
262.0	4.18093E-10	3.19239E-10	4.15437E-10
263.0	4.17946E-10	3.19089E-10	4.15367E-10
264.0	4.17800E-10	3.18939E-10	4.15297E-10
265.0	4.17654E-10	3.18790E-10	4.15227E-10
266.0	4.17510E-10	3.18641E-10	4.15157E-10
267.0	4.17366E-10	3.18492E-10	4.15087E-10
268.0	4.17223E-10	3.18344E-10	4.15017E-10
269.0	4.17082E-10	3.18196E-10	4.14948E-10
270.0	4.16941E-10	3.18048E-10	4.14879E-10
271.0	4.16801E-10	3.17900E-10	4.14810E-10
272.0	4.16662E-10	3.17752E-10	4.14741E-10
273.0	4.16524E-10	3.17605E-10	4.14672E-10
274.0	4.16387E-10	3.17458E-10	4.14604E-10
275.0	4.16251E-10	3.17311E-10	4.14535E-10
276.0	4.16116E-10	3.17165E-10	4.14467E-10
277.0	4.15982E-10	3.17019E-10	4.14399E-10
278.0	4.15848E-10	3.16873E-10	4.14331E-10
279.0	4.15716E-10	3.16727E-10	4.14264E-10
280.0	4.15584E-10	3.16582E-10	4.14197E-10
281.0	4.15453E-10	3.16436E-10	4.14129E-10

282.0	4.15324E-10	3.16292E-10	4.14063E-10
283.0	4.15195E-10	3.16147E-10	4.13996E-10
284.0	4.15067E-10	3.16003E-10	4.13929E-10
285.0	4.14939E-10	3.15858E-10	4.13863E-10
286.0	4.14813E-10	3.15715E-10	4.13797E-10
287.0	4.14688E-10	3.15571E-10	4.13731E-10
288.0	4.14563E-10	3.15428E-10	4.13666E-10
289.0	4.14440E-10	3.15285E-10	4.13600E-10
290.0	4.14317E-10	3.15142E-10	4.13535E-10
291.0	4.14195E-10	3.15000E-10	4.13470E-10
292.0	4.14074E-10	3.14858E-10	4.13406E-10
293.0	4.13954E-10	3.14716E-10	4.13341E-10
294.0	4.13834E-10	3.14574E-10	4.13277E-10
295.0	4.13716E-10	3.14433E-10	4.13213E-10
296.0	4.13598E-10	3.14292E-10	4.13150E-10
297.0	4.13481E-10	3.14151E-10	4.13086E-10
298.0	4.13365E-10	3.14011E-10	4.13023E-10
299.0	4.13250E-10	3.13871E-10	4.12960E-10
300.0	4.13136E-10	3.13731E-10	4.12897E-10
301.0	4.13022E-10	3.13591E-10	4.12835E-10
302.0	4.12910E-10	3.13452E-10	4.12773E-10
303.0	4.12798E-10	3.13313E-10	4.12711E-10
304.0	4.12687E-10	3.13174E-10	4.12650E-10
305.0	4.12577E-10	3.13036E-10	4.12588E-10
306.0	4.12467E-10	3.12898E-10	4.12527E-10
307.0	4.12359E-10	3.12760E-10	4.12466E-10
308.0	4.12251E-10	3.12622E-10	4.12406E-10
309.0	4.12144E-10	3.12485E-10	4.12346E-10
310.0	4.12038E-10	3.12348E-10	4.12286E-10
311.0	4.11933E-10	3.12211E-10	4.12226E-10
312.0	4.11828E-10	3.12075E-10	4.12167E-10
313.0	4.11724E-10	3.11939E-10	4.12107E-10
314.0	4.11621E-10	3.11803E-10	4.12049E-10
315.0	4.11519E-10	3.11668E-10	4.11990E-10
316.0	4.11418E-10	3.11533E-10	4.11932E-10
317.0	4.11317E-10	3.11398E-10	4.11874E-10
318.0	4.11217E-10	3.11263E-10	4.11816E-10
319.0	4.11118E-10	3.11129E-10	4.11759E-10
320.0	4.11020E-10	3.10995E-10	4.11701E-10
321.0	4.10922E-10	3.10861E-10	4.11645E-10
322.0	4.10826E-10	3.10728E-10	4.11588E-10
323.0	4.10730E-10	3.10595E-10	4.11532E-10
324.0	4.10634E-10	3.10462E-10	4.11476E-10
325.0	4.10540E-10	3.10329E-10	4.11420E-10
326.0	4.10446E-10	3.10197E-10	4.11365E-10
327.0	4.10353E-10	3.10065E-10	4.11310E-10
328.0	4.10261E-10	3.09933E-10	4.11255E-10
329.0	4.10170E-10	3.09802E-10	4.11200E-10
330.0	4.10079E-10	3.09671E-10	4.11146E-10
331.0	4.09989E-10	3.09540E-10	4.11092E-10

332.0	4.09900E-10	3.09410E-10	4.11038E-10
333.0	4.09811E-10	3.09280E-10	4.10985E-10
334.0	4.09723E-10	3.09150E-10	4.10932E-10
335.0	4.09636E-10	3.09020E-10	4.10879E-10
336.0	4.09550E-10	3.08891E-10	4.10827E-10
337.0	4.09464E-10	3.08762E-10	4.10775E-10
338.0	4.09379E-10	3.08634E-10	4.10723E-10
339.0	4.09295E-10	3.08505E-10	4.10671E-10
340.0	4.09212E-10	3.08377E-10	4.10620E-10
341.0	4.09129E-10	3.08249E-10	4.10569E-10
342.0	4.09047E-10	3.08122E-10	4.10519E-10
343.0	4.08965E-10	3.07995E-10	4.10468E-10
344.0	4.08885E-10	3.07868E-10	4.10418E-10
345.0	4.08805E-10	3.07741E-10	4.10369E-10
346.0	4.08725E-10	3.07615E-10	4.10319E-10
347.0	4.08647E-10	3.07489E-10	4.10270E-10
348.0	4.08569E-10	3.07364E-10	4.10221E-10
349.0	4.08491E-10	3.07238E-10	4.10173E-10
350.0	4.08415E-10	3.07113E-10	4.10124E-10
351.0	4.08339E-10	3.06988E-10	4.10077E-10
352.0	4.08264E-10	3.06864E-10	4.10029E-10
353.0	4.08189E-10	3.06740E-10	4.09982E-10
354.0	4.08115E-10	3.06616E-10	4.09935E-10
355.0	4.08042E-10	3.06492E-10	4.09888E-10
356.0	4.07970E-10	3.06369E-10	4.09842E-10
357.0	4.07898E-10	3.06246E-10	4.09796E-10
358.0	4.07826E-10	3.06123E-10	4.09750E-10
359.0	4.07756E-10	3.06001E-10	4.09704E-10
360.0	4.07686E-10	3.05879E-10	4.09659E-10
361.0	4.07617E-10	3.05757E-10	4.09614E-10
362.0	4.07548E-10	3.05635E-10	4.09570E-10
363.0	4.07480E-10	3.05514E-10	4.09525E-10
364.0	4.07413E-10	3.05393E-10	4.09482E-10
365.0	4.07346E-10	3.05272E-10	4.09438E-10
366.0	4.07280E-10	3.05152E-10	4.09395E-10
367.0	4.07215E-10	3.05032E-10	4.09352E-10
368.0	4.07150E-10	3.04912E-10	4.09309E-10
369.0	4.07086E-10	3.04793E-10	4.09266E-10
370.0	4.07022E-10	3.04673E-10	4.09224E-10
371.0	4.06959E-10	3.04554E-10	4.09182E-10
372.0	4.06897E-10	3.04436E-10	4.09141E-10
373.0	4.06835E-10	3.04318E-10	4.09100E-10
374.0	4.06774E-10	3.04199E-10	4.09059E-10
375.0	4.06713E-10	3.04082E-10	4.09018E-10
376.0	4.06653E-10	3.03964E-10	4.08978E-10
377.0	4.06594E-10	3.03847E-10	4.08938E-10
378.0	4.06536E-10	3.03730E-10	4.08898E-10
379.0	4.06477E-10	3.03614E-10	4.08859E-10
380.0	4.06420E-10	3.03497E-10	4.08819E-10
381.0	4.06363E-10	3.03381E-10	4.08781E-10

382.0	4.06307E-10	3.03265E-10	4.08742E-10
383.0	4.06251E-10	3.03150E-10	4.08704E-10
384.0	4.06196E-10	3.03035E-10	4.08666E-10
385.0	4.06141E-10	3.02920E-10	4.08628E-10
386.0	4.06087E-10	3.02805E-10	4.08591E-10
387.0	4.06034E-10	3.02691E-10	4.08554E-10
388.0	4.05981E-10	3.02577E-10	4.08517E-10
389.0	4.05929E-10	3.02463E-10	4.08481E-10
390.0	4.05877E-10	3.02349E-10	4.08444E-10
391.0	4.05826E-10	3.02236E-10	4.08408E-10
392.0	4.05776E-10	3.02123E-10	4.08373E-10
393.0	4.05726E-10	3.02011E-10	4.08338E-10
394.0	4.05676E-10	3.01898E-10	4.08303E-10
395.0	4.05627E-10	3.01786E-10	4.08268E-10
396.0	4.05579E-10	3.01674E-10	4.08233E-10
397.0	4.05531E-10	3.01563E-10	4.08199E-10
398.0	4.05484E-10	3.01451E-10	4.08165E-10
399.0	4.05438E-10	3.01340E-10	4.08132E-10
400.0	4.05391E-10	3.01230E-10	4.08098E-10
401.0	4.05346E-10	3.01119E-10	4.08065E-10
402.0	4.05301E-10	3.01009E-10	4.08033E-10
403.0	4.05256E-10	3.00899E-10	4.08000E-10
404.0	4.05212E-10	3.00789E-10	4.07968E-10
405.0	4.05169E-10	3.00680E-10	4.07936E-10
406.0	4.05126E-10	3.00571E-10	4.07905E-10
407.0	4.05084E-10	3.00462E-10	4.07873E-10
408.0	4.05042E-10	3.00353E-10	4.07842E-10
409.0	4.05000E-10	3.00245E-10	4.07812E-10
410.0	4.04960E-10	3.00137E-10	4.07781E-10
411.0	4.04919E-10	3.00029E-10	4.07751E-10
412.0	4.04879E-10	2.99922E-10	4.07721E-10
413.0	4.04840E-10	2.99815E-10	4.07691E-10
414.0	4.04801E-10	2.99708E-10	4.07662E-10
415.0	4.04763E-10	2.99601E-10	4.07633E-10
416.0	4.04725E-10	2.99494E-10	4.07604E-10
417.0	4.04688E-10	2.99388E-10	4.07576E-10
418.0	4.04651E-10	2.99282E-10	4.07547E-10
419.0	4.04615E-10	2.99177E-10	4.07519E-10
420.0	4.04579E-10	2.99071E-10	4.07492E-10
421.0	4.04544E-10	2.98966E-10	4.07464E-10
422.0	4.04509E-10	2.98861E-10	4.07437E-10
423.0	4.04475E-10	2.98757E-10	4.07410E-10
424.0	4.04441E-10	2.98652E-10	4.07383E-10
425.0	4.04408E-10	2.98548E-10	4.07357E-10
426.0	4.04375E-10	2.98444E-10	4.07331E-10
427.0	4.04342E-10	2.98341E-10	4.07305E-10
428.0	4.04311E-10	2.98237E-10	4.07280E-10
429.0	4.04279E-10	2.98134E-10	4.07254E-10
430.0	4.04248E-10	2.98031E-10	4.07229E-10
431.0	4.04218E-10	2.97929E-10	4.07204E-10

432.0	4.04188E-10	2.97826E-10	4.07180E-10
433.0	4.04158E-10	2.97724E-10	4.07155E-10
434.0	4.04129E-10	2.97622E-10	4.07131E-10
435.0	4.04100E-10	2.97521E-10	4.07108E-10
436.0	4.04072E-10	2.97420E-10	4.07084E-10
437.0	4.04044E-10	2.97319E-10	4.07061E-10
438.0	4.04017E-10	2.97218E-10	4.07038E-10
439.0	4.03990E-10	2.97117E-10	4.07015E-10
440.0	4.03964E-10	2.97017E-10	4.06993E-10
441.0	4.03938E-10	2.96917E-10	4.06970E-10
442.0	4.03912E-10	2.96817E-10	4.06948E-10
443.0	4.03887E-10	2.96717E-10	4.06927E-10
444.0	4.03863E-10	2.96618E-10	4.06905E-10
445.0	4.03839E-10	2.96519E-10	4.06884E-10
446.0	4.03815E-10	2.96420E-10	4.06863E-10
447.0	4.03792E-10	2.96321E-10	4.06842E-10
448.0	4.03769E-10	2.96223E-10	4.06822E-10
449.0	4.03746E-10	2.96125E-10	4.06801E-10
450.0	4.03724E-10	2.96027E-10	4.06781E-10
451.0	4.03703E-10	2.95929E-10	4.06761E-10
452.0	4.03681E-10	2.95832E-10	4.06742E-10
453.0	4.03661E-10	2.95734E-10	4.06722E-10
454.0	4.03640E-10	2.95637E-10	4.06703E-10
455.0	4.03620E-10	2.95541E-10	4.06685E-10
456.0	4.03601E-10	2.95444E-10	4.06666E-10
457.0	4.03582E-10	2.95348E-10	4.06648E-10
458.0	4.03563E-10	2.95252E-10	4.06629E-10
459.0	4.03545E-10	2.95156E-10	4.06611E-10
460.0	4.03527E-10	2.95060E-10	4.06594E-10
461.0	4.03509E-10	2.94965E-10	4.06576E-10
462.0	4.03492E-10	2.94870E-10	4.06559E-10
463.0	4.03475E-10	2.94775E-10	4.06542E-10
464.0	4.03459E-10	2.94680E-10	4.06525E-10
465.0	4.03443E-10	2.94586E-10	4.06509E-10
466.0	4.03428E-10	2.94492E-10	4.06492E-10
467.0	4.03412E-10	2.94398E-10	4.06476E-10
468.0	4.03398E-10	2.94304E-10	4.06460E-10
469.0	4.03383E-10	2.94210E-10	4.06445E-10
470.0	4.03369E-10	2.94117E-10	4.06429E-10
471.0	4.03356E-10	2.94024E-10	4.06414E-10
472.0	4.03343E-10	2.93931E-10	4.06399E-10
473.0	4.03330E-10	2.93838E-10	4.06384E-10
474.0	4.03317E-10	2.93746E-10	4.06370E-10
475.0	4.03305E-10	2.93654E-10	4.06355E-10
476.0	4.03293E-10	2.93562E-10	4.06341E-10
477.0	4.03282E-10	2.93470E-10	4.06327E-10
478.0	4.03271E-10	2.93379E-10	4.06314E-10
479.0	4.03260E-10	2.93287E-10	4.06300E-10
480.0	4.03250E-10	2.93196E-10	4.06287E-10
481.0	4.03240E-10	2.93105E-10	4.06274E-10

482.0	4.03231E-10	2.93014E-10	4.06261E-10
483.0	4.03221E-10	2.92924E-10	4.06248E-10
484.0	4.03213E-10	2.92834E-10	4.06236E-10
485.0	4.03204E-10	2.92744E-10	4.06223E-10
486.0	4.03196E-10	2.92654E-10	4.06211E-10
487.0	4.03188E-10	2.92564E-10	4.06199E-10
488.0	4.03181E-10	2.92475E-10	4.06188E-10
489.0	4.03174E-10	2.92385E-10	4.06176E-10
490.0	4.03167E-10	2.92296E-10	4.06165E-10
491.0	4.03160E-10	2.92208E-10	4.06154E-10
492.0	4.03154E-10	2.92119E-10	4.06143E-10
493.0	4.03149E-10	2.92031E-10	4.06132E-10
494.0	4.03143E-10	2.91942E-10	4.06122E-10
495.0	4.03138E-10	2.91854E-10	4.06111E-10
496.0	4.03133E-10	2.91767E-10	4.06101E-10
497.0	4.03129E-10	2.91679E-10	4.06091E-10
498.0	4.03125E-10	2.91592E-10	4.06082E-10
499.0	4.03121E-10	2.91504E-10	4.06072E-10
500.0	4.03118E-10	2.91417E-10	4.06063E-10
